# Changes in serum creatinine in patients with active rheumatoid arthritis treated with tofacitinib: results from clinical trials

**DOI:** 10.1186/ar4673

**Published:** 2014-07-25

**Authors:** John D Isaacs, Andrea Zuckerman, Sriram Krishnaswami, Chudy Nduaka, Shuping Lan, Matthew M Hutmacher, Mary G Boy, Ken Kowalski, Sujatha Menon, Richard Riese

**Affiliations:** The National Institute for Health Research and Newcastle Biomedical Research Centre based at Newcastle Hospitals Foundation Trust and Newcastle University, Framlington Place, Newcastle upon Tyne, NE2 4HH UK; Pfizer Inc, 558 Eastern Point Road, Groton, CT 06340 USA; Ann Arbor Pharmacometrics Group (A2PG), 110 Miller Avenue, Ann Arbor, MI 48104 USA

## Abstract

**Introduction:**

Small increases in mean serum creatinine (SCr) were observed in studies of rheumatoid arthritis patients during tofacitinib treatment. These SCr changes were investigated and potential mechanisms explored.

**Methods:**

SCr values and renal adverse event data were pooled from five Phase 3 and two long-term extension (LTE) studies. Dose-response relationships and association with inflammation (C-reactive protein (CRP)) were explored using Phase 2 data and confirmed with Phase 3 data.

**Results:**

In Phase 3, least squares mean SCr differences from placebo at Month 3 were 0.02 and 0.04 mg/dl for tofacitinib 5 and 10 mg twice daily (BID) (*P* <0.05), respectively. During Months 0 to 3, confirmed SCr ≥33% increases over baseline were reported in 17 (1.4%; 5 mg BID) and 23 (1.9%; 10 mg BID) patients. Generally, elevations plateaued and remained within normal limits throughout Phase 3 and LTE studies. Exposure-response modeling demonstrated small, reversible effects of tofacitinib on mean SCr, and significant (*P* <0.05) effects of CRP on model parameters. Phase 3 data confirmed that patients with higher baseline CRP or greater CRP decreases following tofacitinib treatment had the largest increases in SCr. Across Phase 3 and LTE studies, 22 tofacitinib-treated patients had clinical acute renal failure (ARF), predominantly in the setting of concurrent serious illness.

**Conclusions:**

Tofacitinib treatment was associated with small, reversible mean increases in SCr that plateaued early. The mechanism behind these SCr changes remains unknown, but may involve effects of tofacitinib on inflammation. ARF occurred infrequently, was associated with concurrent serious illness, and was unrelated to prior SCr increases.

**Electronic supplementary material:**

The online version of this article (doi:10.1186/ar4673) contains supplementary material, which is available to authorized users.

## Introduction

Tofacitinib is an oral Janus kinase (JAK) inhibitor for the treatment of rheumatoid arthritis (RA). It is a selective inhibitor of the JAK family and blocks intracellular signaling of multiple key cytokines involved in the inflammatory cascade [1].

Six Phase 3 studies of tofacitinib in several RA patient populations have been completed. Tofacitinib dosed 5 and 10 mg twice daily (BID), as monotherapy or in combination with nonbiologic disease-modifying anti-rheumatic drugs (DMARDs), was efficacious, providing benefits in the signs and symptoms of RA, structural preservation, and in physical function and patient-reported outcomes. The safety profile of tofacitinib was consistent across the studies [2–7].

During the tofacitinib RA clinical development program, increases in mean serum creatinine (SCr) were observed in patients, despite lack of nephrotoxicity in preclinical and healthy volunteer studies [8–10]. Although SCr is widely used as an indicator of glomerular filtration rate (GFR) and therefore renal function, a number of factors may influence its generation and clearance for example, glomerular filtration of creatinine, muscle mass and turnover [11–13].

The objectives of this report are to investigate the clinical significance of these changes in SCr in the RA population and to address potential mechanisms.

## Methods

SCr values and renal adverse event (AE) data were pooled from five Phase 3 studies (in DMARD-inadequate responders) and two ongoing long-term extension (LTE) studies investigating tofacitinib (CP-690,550; Pfizer Inc, Groton, CT, USA) in RA. Evaluation of exposure-response relationships and covariate analyses were performed on five Phase 2 studies using nonlinear mixed-effects models. Phase 3 data were used to verify the hypothesis generated from exposure-response models, with emphasis on the relationship between changes in SCr and C-reactive protein (CRP), and creatine kinase (CK; small changes in CK were observed during Phase 3 studies) and CRP.

### Clinical studies

Tofacitinib 1, 3, 5, 10, 15, and 30 mg BID or 20 mg once daily (QD; A3921025 only) was investigated in five Phase 2 randomized, double-blind, placebo-controlled, multicenter studies (index studies): A3921019 (NCT00147498), A3921035 (NCT00550446), and A3921040 (NCT00687193) were tofacitinib monotherapy studies; A3921025 (NCT00413660) and A3921039 (NCT00603512) included background methotrexate (MTX). A3921035 included a monotherapy adalimumab arm as an active control. Details of individual study designs have been published previously [14–18].

The Phase 3 studies were double-blind, placebo-controlled, global studies (index studies): ORAL Step (A3921032; NCT00960440), ORAL Scan (A3921044; NCT00847613), ORAL Solo (A3921045; NCT00814307), ORAL Sync (A3921046; NCT00856544), and ORAL Standard (A3921064; NCT00853385). Tofacitinib 5 or 10 mg BID was investigated alone or in combination with nonbiologic DMARDs, mainly MTX, in several populations of RA patients who had previously demonstrated an inadequate response to at least one nonbiologic or biologic DMARD. ORAL Standard included an adalimumab plus MTX arm as an active control. In studies of ≥6 months’ duration, placebo patients who did not achieve a decrease in tender and swollen joints of at least 20% were advanced to tofacitinib at Month 3. All remaining placebo patients advanced to tofacitinib at Month 6. In the shorter studies, all placebo patients were advanced to tofacitinib at Month 3. Details of individual study designs have been published previously [2–6].

Patients from the Phase 2 and Phase 3 index studies could participate in one of two open-label, LTE studies. A3921024 (NCT00413699) included patients outside Japan. A3921041 (NCT00661661) was a multicenter study of Japanese patients from the Phase 2 (A3921039 or A3921040) and Phase 3 (ORAL Scan) studies. Upon LTE study entry, patients from Phase 2 received 5 mg BID and patients from Phase 3 received 10 mg BID, with the exception of Chinese and Japanese patients in Phase 3 studies who received 5 mg BID. Dose adjustments, as well as adjustments of concomitant medications, were permitted in the LTE studies [19]. LTE data collection and analyses are still ongoing; therefore, some values may change for the final analyses.

Full inclusion and exclusion criteria can be found in the individual study publications. Briefly, across Phases 2 and 3, patients were aged ≥18 years (≥20 years in Japan) with a diagnosis of moderate to severe active RA for ≥6 months and met American College of Rheumatology 1987 revised classification criteria [20]. Exclusion criteria relevant to the current analyses included: GFR ≤60 ml/min (the Cockcroft-Gault calculation) for A3921019, GFR ≤50 ml/min for the remaining Phase 2 studies, and GFR ≤40 ml/min for the five Phase 3 studies; or current or recent history of uncontrolled clinically significant renal disease.

Data presented in this study were taken from five Phase 2, five Phase 3 and two LTE studies. All studies were conducted in compliance with the Declaration of Helsinki, International Conference on Harmonisation Good Clinical Practice guidelines, and local country regulations. The final protocols, any amendments, and informed consent documentation were reviewed and approved by the Institutional Review Board and the Independent Ethics Committee of the investigational centers - see Additional files 1, 2, 3, 4, 5, 6, 7, 8, 9, 10, 11, and 12. All patients provided written informed consent.

### SCr monitoring and discontinuation

For the Phase 2 studies, baseline values were those taken on Day 0 or Week 0 prior to the first study dose. For the Phase 3 studies, calculated baseline values were the average of the screening and baseline values. For the LTE studies, baseline values were those of the Phase 2 or Phase 3 study for patients enrolling within 7 (A3921041) or 14 (A3921024) days of participation; if enrollment was >7 or >14 days after participation, baseline was the start of the LTE study prior to the first LTE study dose. In Phase 2 studies other than A3921019, patients were withdrawn for SCr increases >50% relative to baseline. In the Phase 3 and A3921041 LTE studies, patients discontinued therapy if two sequential increases in SCr >50% over baseline values were reported. Patients with SCr ≥33% at the end of treatment (EoT) or discontinuation were followed until SCr levels were within 10% of the baseline/screening value. In the A3921024 LTE study, for any SCr increase >50% over baseline values, adjustments in concomitant medications and/or dose of study drug were permitted within a 90-day period from the initial >50% SCr increase. If the >50% increase persisted for >90 days, the patient was withdrawn. However, patients were to be withdrawn if they had two sequential increases in SCr >100% over baseline values, irrespective of the 90-day period (protocol-specified). To assess smaller increases in SCr than those requiring discontinuation, SCr changes of ≥33% were analyzed throughout the index study, as well as at the EoT or discontinuation.

### Shift analyses

To examine the pattern of longer-term changes, patients in Phase 3 who continued into the LTE studies were classified into four categories based on their change from baseline in SCr at the EoT in the Phase 3 studies: ≤10%, >10 to <33%, ≥33 to ≤50%, and >50%. Patients in these four cohorts were followed in the LTE studies and reclassified into the same categories, based on the maximum percentage increase category achieved in two consecutive SCr measurements. Mean changes in SCr from baseline to the EoT in the Phase 3 and to the last observed visit (LOV) in the LTE studies were calculated for each of the four cohorts.

### Exposure-response analyses

The SCr data were described using a nonlinear mixed-effects model with multiplicative inter-individual random effects and an additive residual random effect (measurement error) to characterize the relationship between tofacitinib exposure and SCr levels. The structural form of the model was an indirect response model [21] comprising the following parameters: baseline (*R*_*0*_), SCr elimination rate constant (*K*_*out*_), maximum inhibitory drug effect (*E*_*max*_), and the dose that achieves 50% of the maximum drug effect (*ED*_*50*_). No inference was made with respect to whether tofacitinib influences the clearance or production of SCr despite the functional form of the model. An early dose-ranging Phase 2 study (A3921019) assessed SCr reversibility; patients with RA received tofacitinib 5, 15 or 30 mg BID for six weeks, followed by a six-week washout period, and SCr levels were monitored.

To explore a possible relationship between inflammation and changes in SCr, the influence of baseline CRP was estimated on four model parameters: *R*_*0*_, *K*_*out*_, *E*_*max*_, and *ED*_*50*_. Likelihood ratio tests were applied to evaluate improvement in goodness-of-fit when baseline CRP was added to the model. All analyses were performed using the first-order conditional-estimation method as implemented in the NONMEM software (v6, and v7 once available; ICON Plc, Dublin, Ireland).

Data from Phase 3 studies, not used in the modeling analysis, were analyzed descriptively by comparing the mean changes in SCr based on quartiles of baseline CRP, as well as quartiles of change from baseline in CRP at Week 12. Small increases in CK were observed in the Phase 3 clinical program; therefore, a similar analysis was performed for changes in CK as a function of CRP and for changes in SCr as a function of changes in CK.

### Adverse event analysis

AE data from Phase 3 and LTE studies were classified using the narrow SMQ (Standardized MedDRA (Medical Dictionary for Regulatory Activities) Queries) term of acute renal failure (ARF) and was further narrowed to include all patients whose AE met one of the following criteria: coded to the narrow SMQ of ARF (including preferred terms of acute phosphate nephropathy, acute prerenal failure, anuria, azotemia, continuous hemodiafiltration, dialysis, hemodialysis, nephropathy toxic, oliguria, peritoneal dialysis, renal failure, renal impairment); required permanent or temporary discontinuation of the study drug; or required a dose reduction of the study drug. This included laboratory investigations (for example, increased blood creatinine) as well as clinical renal and urinary disorders (for example, proteinuria, nephritis, renal impairment, ARF) [22]. Patients with clinical ARF were defined by the MedDRA terms: renal failure, azotemia, or renal impairment [22].

Patient data were then reviewed (nonblinded) in order to ascertain a potential etiology (or etiologies) for the renal AE. Data reviewed included demographics, investigator-reported term for the event, day of onset, and day of resolution or last evaluation - all relative to the start of the study drug, SCr, creatinine clearance (the Cockcroft-Gault calculation), maximum SCr value achieved, tofacitinib dose, concomitant medications, past medical history, associated or concurrent events, action taken, outcome of the event, and investigator and Pfizer case assessments. The focus of the concomitant medication review was on those medications with known associations with changes in renal function such as angiotensin-converting enzyme (ACE) inhibitors or angiotensin receptor blockers (ARBs), nonsteroidal anti-inflammatory drugs (NSAIDs) (both selective and nonselective cyclooxygenase inhibitors), diuretics, and nephrotoxic agents.

## Results

Across Phase 3, 3,315 patients received ≥1 dose of study drug (tofacitinib, placebo, or adalimumab) for durations that ranged up to 474 days. In the LTE studies, as of March 29, 2011, 3,227 patients received ≥1 dose of tofacitinib; of those, 2,019 received tofacitinib with background DMARDs and 1,208 received tofacitinib monotherapy. The Phase 2 model dataset held 1,475 patients, with 10,343 SCr observations for placebo and tofacitinib treatments and with a tofacitinib 1 to 30 mg BID dose range up to 24 weeks in duration.

In Phase 2 and 3 studies, patients had a mean age of 50.6 to 53.4 and 52.2 to 53.2 years, respectively; most patients were female and were Caucasian (with the exception of A3921039 and A3921040 where all patients were Japanese).

### Changes in SCr

**Figure 1:**
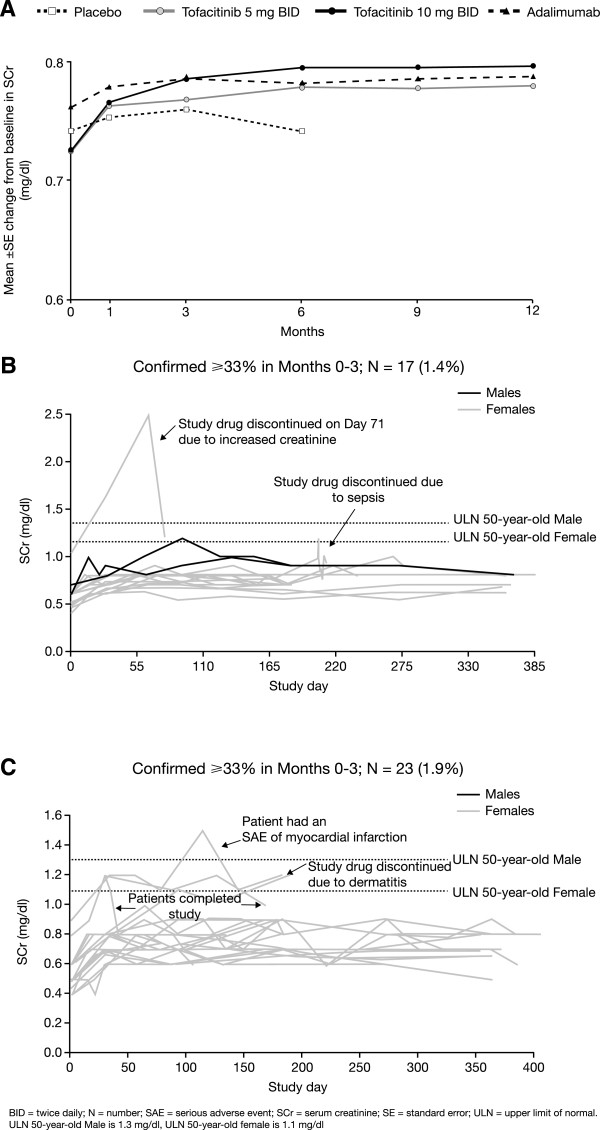
**SCr changes in Phase 3 studies. (A)** Mean (±SE) change from baseline in SCr (mg/dl) across the Phase 3 program (0 to 12 months). **(B)** SCr outliers during tofacitinib 5 mg BID treatment. **(C)** SCr outliers during tofacitinib 10 mg BID treatment.

Tofacitinib treatment in pooled Phase 3 studies over 12 months resulted in mean increases from baseline in SCr levels (Figure 1A). Increases occurred predominantly within the first three months. The least squares (LS) mean SCr increases at Month 3 were 0.07 and 0.08 mg/dl for 5 and 10 mg BID tofacitinib doses, respectively, compared with 0.04 mg/dl in the placebo and 0.06 mg/dl in the adalimumab groups. The differences (upper 95% confidence intervals) between the tofacitinib groups and the placebo group in LS mean increases were estimated to be 0.02 (0.04) and 0.04 (0.05) mg/dl for the 5 and 10 mg BID groups, respectively.

In the tofacitinib 5 mg BID group, 17/1,220 (1.4%) patients had a confirmed SCr increase of ≥33% from baseline in Months 0 to 3. Of these, two had a SCr measurement above the reference range (upper limit of normal: 1.1 and 1.3 mg/dl for females and males, respectively). One patient discontinued due to increased SCr with subsequent return to baseline. The second patient discontinued due to an episode of sepsis (Figure 1B). In the tofacitinib 10 mg BID group, 23/1,217 (1.9%) patients had a confirmed SCr increase ≥33% from baseline in Months 0 to 3. Four patients of these 23 patients had SCr values above the reference range: one patient experienced a serious adverse event (SAE) of myocardial infarction, but recovered, continued tofacitinib treatment, and completed the trial; one discontinued due to an AE of dermatitis; and in two patients the creatinine returned to within normal limits with continued tofacitinib treatment and they completed the trial (Figure 1C). Patients with two or more SCr increases ≥33% over baseline generally displayed stable or reduced levels over the remainder of the Phase 3 studies, despite continuation of therapy. A total of 16 and 12 tofacitinib-treated patients permanently discontinued due to SCr increases >50% over baseline in the Phase 3 and LTE (as of September 29, 2011) studies, respectively.

### Shift analyses

Patients demonstrating rises in SCr at the end of the index study showed no further increase in SCr during the LTE study (Tables 1 and 2). As shown in Table 1, the majority of patients who exhibited a >10% increase in SCr at the EoT in the Phase 3 studies either stayed in the same category or were in a lower category in the LTE studies. Similarly, patients who exhibited a >10% increase in SCr at the EoT in Phase 3 had very similar changes at both the EoT in Phase 3 and LOV in the LTE (Table 2). Together, these data indicate that patients with larger changes (>10%) in SCr in the Phase 3 studies did not demonstrate a further increase in SCr levels in the LTE studies.

**Table 1:** **SCr levels in the LTE and Phase 3 studies: number (percentage) of patients with maximum confirmed increase in SCr in the LTE studies versus increase at EoT in the Phase 3 studies**

SCr increase at EoT in Phase 3 (%)	LTE study: maximum percentage confirmed increase in creatinine (n = 1,651)				
	≤10%	>10 to <33%	≥33 to ≤50%	>50%	Total
≤10%	634 (69.8)	264 (29.0)	9 (1.0)	2 (0.2)	909 (100.0)
>10 to <33%	208 (31.9)	395 (60.5)	43 (6.6)	7 (1.1)	653 (100.0)
≥33 to ≤50%	24 (29.3)	28 (34.2)	24 (29.3)	6 (7.3)	82 (100.0)
>50%	0 (0.0)	2 (28.6)	2 (28.6)	3 (42.9)	7 (100.0)

EoT, end of treatment; LTE, long-term extension; SCr, serum creatinine.

**Table 2:** **SCr levels in the LTE and Phase 3 studies: stabilization of SCr levels in the LTE studies**

SCr increase at EoT in Phase 3 (%)	Mean (%) increase from baseline at EoT in index study, mg/dl	Mean (%) increase from baseline at last observed visit in LTE, mg/dl
≤10% (n = 909)	0.00 (0.3)	0.05 (7.5)
>10 to <33% (n = 653)	0.13 (18.6)	0.13 (18.5)
≥33 to ≤50% (n = 82)	0.25 (38.2)	0.24 (36.5)
>50% (n = 7)	0.32 (56.0)	0.26 (44.9)

EoT, end of treatment; LTE, long-term extension; SCr, serum creatinine.

### Exposure-response analyses

Analyses of Phase 2 data suggested a dose response in mean SCr. The final model estimated an *ED*_*50*_ of 0.88 mg suggesting that doses of tofacitinib 5 mg BID and greater are in excess of the *ED*_*80*_ (3.5 mg). Therefore, similar mean SCr increases were predicted for the 5 and 10 mg BID doses in Phase 3 trials.

**Figure 2:**
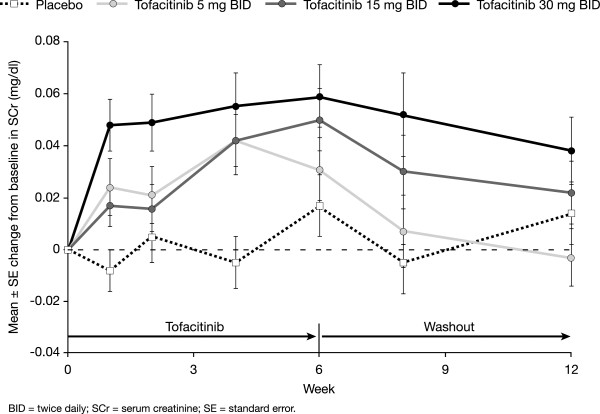
**SCr reversibility in a six-week Phase 2 study (A3921019) of tofacitinib.**

SCr levels at Month 12 in Phase 3 and in LTE studies were consistent with predictions at Month 6 from the Phase 2 double-blind data, suggesting that there was no evidence of a progressive increase in SCr levels with long-term treatment. In the A3921019 study, SCr increases reached steady-state levels in a typical patient by approximately six weeks and reversed in patients on 5 mg BID two to six weeks after stopping tofacitinib (Figure 2); the 15 mg and 30 mg BID doses were not carried forward into Phase 3 studies. In both Phase 3 and LTE studies, SCr changes were similar between patients receiving tofacitinib plus background DMARDs and those on tofacitinib monotherapy.

**Figure 3:**
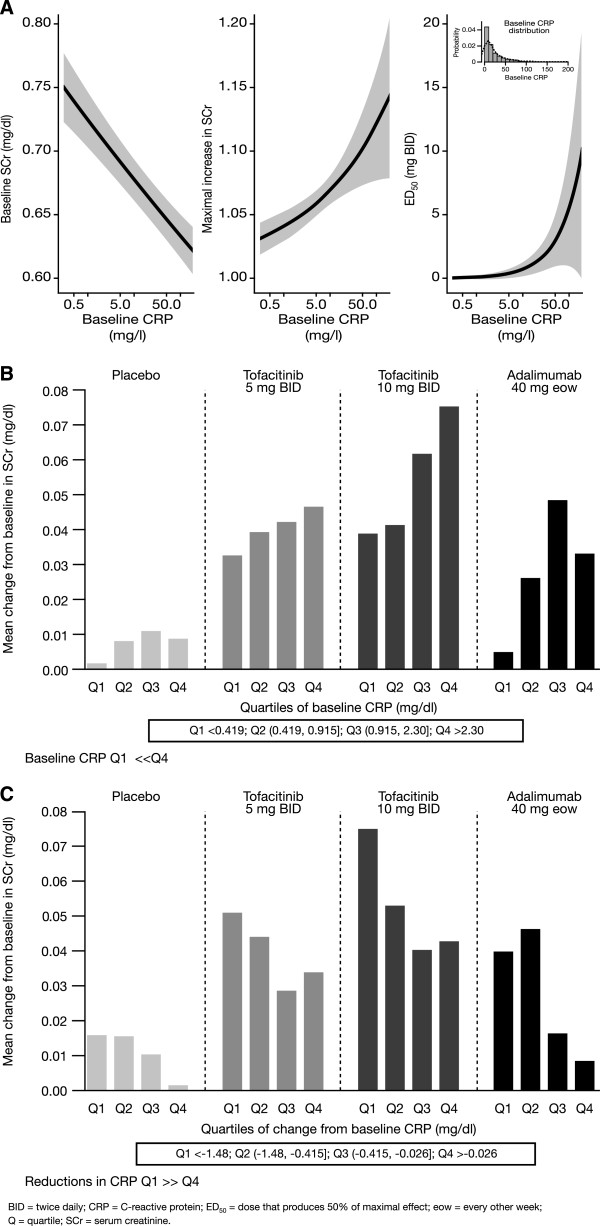
**Relationships between SCr and CRP using Phase 2 modeling data and Phase 3 data. (A)** Relationships between SCr model parameters and baseline CRP. **(B)** SCr increases at Month 3 by baseline CRP; baseline CRP Q1 < < Q4. **(C)** SCr increases at Month 3 by change in CRP at Month 3; reductions in CRP Q1 > > Q4.

Likelihood ratio tests indicated statistically significant (*P* <0.05) effects of baseline CRP on baseline, *E*_*max*_, and *ED*_*50*_. Lower baseline SCr values, greater maximum effects of tofacitinib on SCr (greater *E*_*max*_), and lower potencies (higher *ED*_*50*_) were associated with greater baseline CRP values (Figure 3A). The onset rate of the SCr changes did not appear to be associated with baseline CRP (that is, effects of baseline CRP on *K*_*out*_ were not significant).

**Figure 4:**
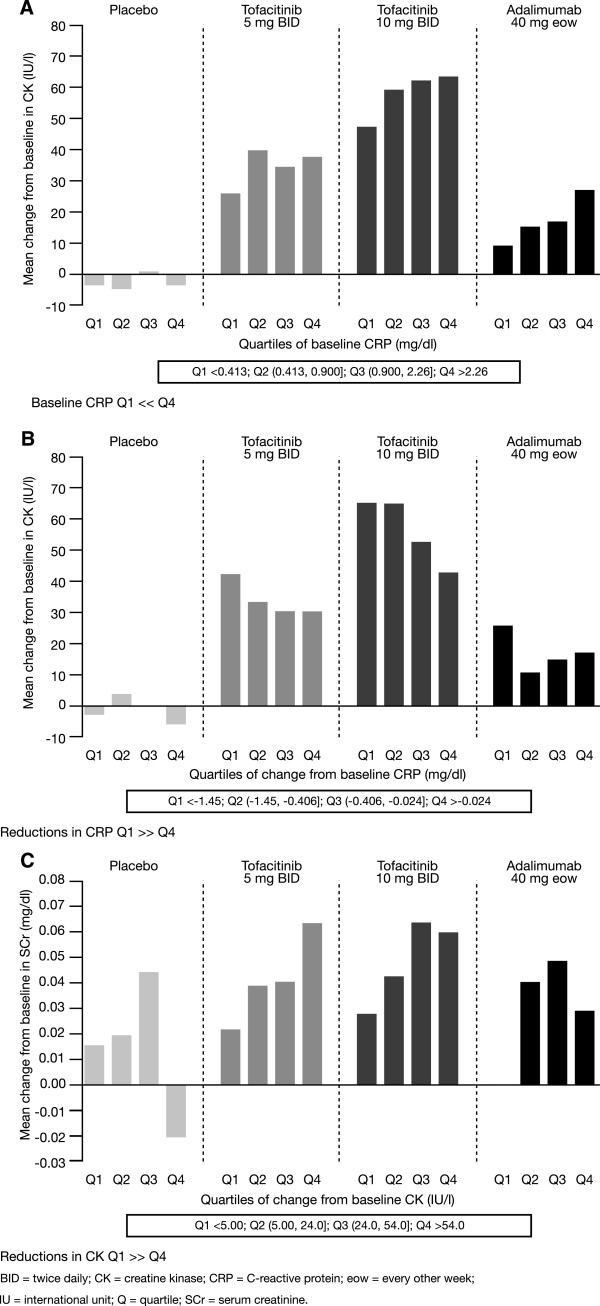
**Relationships between CK and SCr and CRP using Phase 3 data. (A)** CK increases at Week 12 by baseline CRP; baseline CRP Q1 < < Q4. **(B)** CK increases at Month 3 by change in CRP at Month 3; reductions in CRP Q1 > > Q4. **(C)** SCr increases at Month 3 by change in CK at Month 3; reductions in CK Q1 > > Q4.

From the Phase 3 data, it appeared that the higher the burden of inflammation, as assessed by baseline CRP, the greater the increase observed in SCr (Figure 3B). A similar trend was observed with patients treated with adalimumab, although the magnitude of SCr increase was lower than that of tofacitinib. Moreover, patients who showed the greatest reductions in CRP (quartile 1) following tofacitinib treatment appeared to have the highest increases in SCr compared with those who showed smaller reductions in CRP (quartile 4; Figure 3C). This trend was seen across all treatment arms, although the magnitude of increase differed between tofacitinib, adalimumab, and placebo. Similar relationships were seen when CK was analyzed either by quartiles of baseline CRP or change from baseline CRP at Week 12 (Figure 4A and B). There was a trend toward greater increases in SCr in those patients who had greater increases in CK (Figure 4C).

### Adverse event analysis

**Figure 5:**
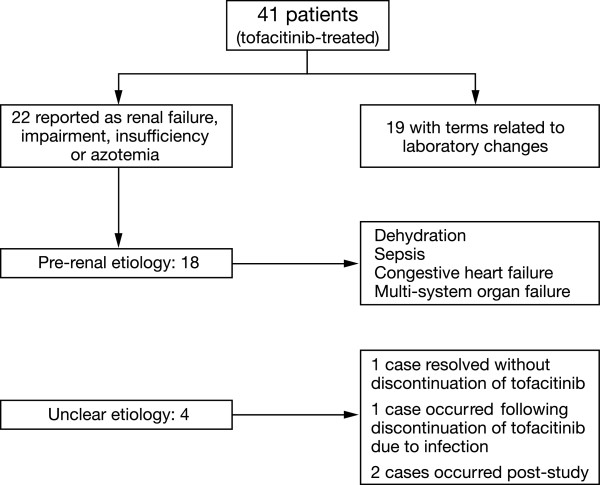
**Evaluation of renal events.** Includes all patients coded to the narrow SMQ of ARF or required permanent discontinuation, temporary discontinuation or dose reduction of the study drug. ARF, acute renal failure; SMQ, Standardized Medical Dictionary for Regulatory Activities Queries.

Of 3,030 tofacitinib-treated patients participating in Phase 3 studies, 0.3 to 0.7% experienced AEs reported as ARF. In the LTE studies, 2.9% and 1.0% of patients in the tofacitinib 5 and 10 mg BID groups, respectively, experienced AEs reported as ARF. Across the Phase 3 and LTE studies, analysis of the 41 ARF AEs identified 22 tofacitinib-treated patients as having clinical ARF (Figure 5). Of these, 18 patients had a likely prerenal cause such as dehydration, sepsis, congestive heart failure, or multi-system organ failure; four patients had an unclear etiology, two of which were post-study: of these, one patient experienced postoperative ARF following surgery for metastatic adenocarcinoma six weeks post-study, and the other after hip surgery eight weeks post-study (Additional file 13: Table S1). Of these 22 patients, six died, 13 resolved, two had ongoing cases of ARF at the time of the final visit in the LTE studies, and one improved. One patient receiving placebo died, with sepsis as a likely prerenal cause (Additional file 13: Table S1). Concomitant medications, including ACE inhibitors, ARBs, NSAIDs, and diuretics, were frequently used by patients with ARF, consistent with use in patients with RA. Resolution of ARF generally occurred with therapy of the prerenal contributing factors and temporary or permanent discontinuation of tofacitinib. Similar resolution occurred in those with laboratory test result increases of SCr.

## Discussion

Plasma or serum creatinine and its renal clearance are the most widely used indicators of GFR and thus renal function and increases in SCr in drug studies are often considered surrogates for impairment of renal function and clinical toxicity [11–13]. As SCr is derived from muscle creatine, factors that impact SCr levels include muscle mass and muscle turnover (recovery from starvation, muscle disease), medications, dietary protein, and creatine levels in addition to glomerular filtration and tubular transport [11–13]. Since these parameters themselves can vary longitudinally the relationship between SCr and GFR can change over time [12]. This is apparent in patients with RA, for example, where reduced muscle mass, immobilization or reduced activity, and inflammation, can further complicate the interpretation of SCr, such that SCr levels are often considered to be a relatively poor indicator of GFR in this population [11, 13].

No effect of tofacitinib on SCr or measured GFR was seen in healthy volunteers. Additionally, a study of tofacitinib co-administered with metformin, to assess possible effects on tubular secretion by organic cationic transporters (OCTs), reported no changes in metformin systemic exposure or renal clearance in healthy volunteers, and thus did not support altered tubular secretion as a mechanism for tofacitinib-associated changes in SCr (A3921143; NCT01405118 [23]). Nonetheless, since the study was not performed in patients with RA, and given that inflammation affects the expression of transporters and drug-metabolizing enzymes [24], the possibility of RA interfering with OCT expression, which is then corrected by tofacitinib, cannot be excluded.

Data on the natural course of SCr changes in patients with RA and in response to DMARDs other than MTX or calcineurin inhibitors are limited. In a MTX study, renal clearance of MTX at six months decreased by a mean (SD) of 23.8 (40.3) ml/min (*P* = 0.014) and creatinine clearance (standard formula) decreased by a mean (SD) of 8.6 ml/min (17.2) (*P* = 0.033) [25]. In a 24-week study of tacrolimus (a calcineurin inhibitor) in patients with RA, SCr elevations ≥40% occurred in 8.7% (6/69), 18.8% (12/64), 28.1% (18/64), and 7% (5/71) of patients receiving tacrolimus 1 mg, 3 mg, and 5 mg, and placebo QD, respectively [26]. Measured GFRs were significantly higher at one year for tofacitinib compared to cyclosporine A in a study of 331 kidney transplant recipients, but extrapolation to RA is difficult given differences in the patient population and other factors affecting renal function in patients posttransplantation [27]. In the tofacitinib RA program, small increases in SCr were seen with adalimumab but of lesser magnitude than with tofacitinib. To our knowledge, longitudinal SCr levels in programs of biologic DMARDs have not been described in detail.

Interestingly, increases in SCr have been reported in patients with RA treated with other JAK inhibitors in development. In a 12-week, Phase 2b, dose-ranging study of baricitinib (LY3009104), a JAK1/JAK2 inhibitor, in combination with nonbiologic DMARDs, increases in SCr of 0.09 to 0.11 mg/dl (7.96 and 9.72 μmol/l) following two doses of baricitinib (versus 0.01 mg/dl (0.88 μmol/l) for placebo) were observed [28]. In a 12-week monotherapy study of the investigational JAK3 selective inhibitor, VX-509, increases in SCr across the dose range were reported (0.04 (3.54 μmol/l) versus -0.01 mg/dl (-0.88 μmol/l) for placebo) [29]. The JAK signaling pathway is utilized by receptors for interleukins (ILs), interferons (IFNs), and hormones such as growth hormone and erythropoietin to mediate intracellular signaling [30]. Inhibition of JAK1 and/or JAK3 by tofacitinib blocks signaling through the common gamma chain-containing receptors for several cytokines, including IL-2, -4, -7, -9, -15, and -21, as well as through receptors for cytokines such as IL-6, IFN-gamma, and type 1 IFN [1]. However, the mechanism for increases in SCr with JAK inhibitors is not understood. JAK 1, 2, and 3 have been identified in kidney cells and tissue, but their role is not defined [31, 32]. The available data do not suggest a deleterious role for JAK inhibition in renal injury models; JAK inhibitor AG-490 was beneficial in several renal injury models [33], and JAK inhibitor NC1153 was not nephrotoxic in direct contrast to cyclosporine A in the low-salt rat model [34]. Tofacitinib was not nephrotoxic in standard nonclinical toxicity studies. No effects on SCr or urea nitrogen were observed, and no adverse histologic findings were detected in studies of up to six months in rats, and nine months in monkeys [9, 10]. Systemic exposures, based on area under the plasma concentration versus time curve for unbound tofacitinib, were up to 188 times in rats and six times in monkeys compared to that in patients with RA at a dose of 5 mg BID.

Given that small dose-dependent increases in mean CK were observed with tofacitinib treatment (and to a lesser degree with adalimumab) in the Phase 3 clinical program, we examined the relationship of CRP, as a marker of inflammation, with both SCr and CK. Serum CK has been shown to be lower in patients with RA versus healthy volunteers and to correlate with the level of inflammation [35]. Generally, mean CK tended to plateau after six months of tofacitinib treatment, stayed within the normal reference range and was not associated with clinical myopathy or changes in markers of muscle catabolism (lactate dehydrogenase and myoglobin). Exploratory analyses revealed an association between high baseline CRP levels and low baseline SCr in this RA population. Furthermore, the higher the CRP, the greater the increases seen in SCr upon treatment with tofacitinib; similar results were observed following adalimumab treatment, but of a lesser magnitude. A similar relationship was observed between CK and CRP and patients with greater CK increases also displayed greater SCr changes.

Taken together, these data suggest that the changes in SCr observed in the present studies may be related at least in part to an effect of JAK inhibition on the inflammatory burden of RA, possibly secondary to changes in creatinine generation. A recently completed measured GFR study in patients with RA receiving tofacitinib or placebo will provide further information on tofacitinib’s effect on the kidney (A3921152; NCT01484561).

Clinical manifestations of ARF in the Phase 3 and LTE studies showed no clear dose association and did not occur as a result of continuing SCr increases over time, but occurred acutely, with etiologies consistent with the causes of ARF in the general population (mostly prerenal - congestive heart failure, sepsis, or dehydration) [36]. Concomitant medications were consistent with those used in patients with RA. Resolution of ARF occurred in most patients, usually following treatment of contributing factors and temporary or permanent discontinuation of study drug or concomitant medications.

## Conclusions

Data summarized here suggest that tofacitinib has a small and reversible effect on mean SCr in patients with RA. These changes plateaued and did not appear to be associated with ARF or progressive worsening of renal function in the LTE studies. ARF occurred infrequently with tofacitinib treatment, was observed in the setting of concurrent illnesses associated with ARF in the general population, and generally reversed with appropriate management including discontinuation of study treatment or concomitant medications. The mechanism behind the SCr changes in RA with tofacitinib is presently unknown, and undergoing further investigation; however, exploratory analyses suggest a possible link between tofacitinib-induced changes in inflammation, SCr, and CK.

## Authors’ information

John D Isaacs, PhD, FRCP: The National Institute for Health Research and Newcastle Biomedical Research Centre based at Newcastle Hospitals Foundation Trust and Newcastle University, Newcastle upon Tyne, UK; Andrea Zuckerman, MD, Sriram Krishnaswami, PhD, Chudy Nduaka, DVM, PhD, Shuping Lan, MS, MPH, Mary G Boy, VMD, Sujatha Menon, PhD, Richard Riese, MD, PhD: Pfizer Inc, Groton, Connecticut, USA; Matt M Hutmacher, MS, Ken Kowalski, MS: Ann Arbor Pharmacometrics Group (A2PG), Ann Arbor, Michigan, USA.

## Electronic supplementary material

Additional file 1: **List of Investigators and Corresponding Ethics Committees or Institutional Review Boards for the Phase 2 A3921019 study.** (DOC 166 KB)

Additional file 2: **List of Investigators and Corresponding Ethics Committees or Institutional Review Boards for the LTE A3921024 study.** (DOC 1 MB)

Additional file 3: **List of Investigators and Corresponding Ethics Committees or Institutional Review Boards for the Phase 2 A3921025 study.** (DOC 268 KB)

Additional file 4: **List of Investigators and Corresponding Ethics Committees or Institutional Review Boards for the Phase 3 A3921032 study.** (DOC 320 KB)

Additional file 5: **List of Investigators and Corresponding Ethics Committees or Institutional Review Boards for the Phase 2 A3921035 study.** (DOC 187 KB)

Additional file 6: **List of Investigators and Corresponding Ethics Committees or Institutional Review Boards for the Phase 2 A3921039 study.** (DOC 70 KB)

Additional file 7: **List of Investigators and Corresponding Ethics Committees or Institutional Review Boards for the Phase 2 A3921040 study.** (DOC 168 KB)

Additional file 8: **List of Investigators and Corresponding Ethics Committees or Institutional Review Boards for the LTE A3921041 study.** (DOC 152 KB)

Additional file 9: **List of Investigators and Corresponding Ethics Committees or Institutional Review Boards for the Phase 3 A3921044 study.** (DOC 424 KB)

Additional file 10: **List of Investigators and Corresponding Ethics Committees or Institutional Review Boards for the Phase 3 A3921045 study.** (DOC 328 KB)

Additional file 11: **List of Investigators and Corresponding Ethics Committees or Institutional Review Boards for the Phase 3 A3921046 study.** (DOC 362 KB)

Additional file 12: **List of Investigators and Corresponding Ethics Committees or Institutional Review Boards for the Phase 3 A3921064 study.** (DOC 417 KB)

Additional file 13: Table S1: Acute renal failure cases. (DOC 54 KB)

## Abbreviations

ACE: angiotensin-converting enzyme
AE: adverse event
ARBs: angiotensin receptor blockers
ARF: acute renal failure
BID: twice daily
CK: creatine kinase
CRP: C-reactive protein
DMARDs: disease-modifying anti-rheumatic drugs
*ED*_*50*_: dose that achieves 50% of the maximum drug effect
*E*_*max*_: maximum inhibitory drug effect
EoT: end of treatment
GFR: glomerular filtration rate
IFNs: interferons
ILs: interleukins
JAK: Janus kinase
*K*_*out*_: SCr elimination rate constant
LOV: last observed visit
LS: least squares
LTE: long-term extension
MedDRA: Medical Dictionary for Regulatory Activities
MTX: methotrexate
NSAIDs: non-steroidal anti-inflammatory drugs
OCTs: organic cationic transporters
QD: once daily
*R*_*0*_: baseline
RA: rheumatoid arthritis
SAE: serious adverse event
SCr: serum creatinine
SMQ: Standardized Medical Dictionary for Regulatory Activities Queries.
