# Supplementary material for: Changes in serum creatinine in patients with active rheumatoid arthritis treated with tofacitinib: results from clinical trials
Source: Arthritis Res Ther. 2014 Jul 25;16(4):R158. doi: 10.1186/ar4673 (PMC4220634; doi:10.1186/ar4673)
Supplement: Supplementary file 1 — Additional file 1: List of Investigators and Corresponding Ethics Committees or Institutional Review Boards for the Phase 2 A3921019 study. (DOC 166 KB) [file 13075_2013_4378_MOESM1_ESM.doc]

# A4 LIST OF INVESTIGATORS AND CORRESPONDING ETHICS COMMITTEES OR INSTITUTIONAL REVIEW BOARDS

## Austria

**Coordinating Investigators:**

<None Entered>

| **Center** | **Principal Investigator** | **Co-Investigator(s)** | **Sub-Investigator(s)** | **Address(es)** | **Institutional Review Board or Ethics Committee Address(es)** |
| --- | --- | --- | --- | --- | --- |
|  |  |  |  |  |  |
| 1091 | Dr. Omid Zamani |  | Dr. Joerg Dietmar Rieger | Rheuma-Ordination Favoriten  Quellenstrasse 181  Wien, A-1100  AUSTRIA | Ethikkommission der Medizinischen Universitaet Graz  LKH-Universitätsklinikum - Eingangsgebäude  Auenbruggerplatz 2, 3.OG  Graz, A-8036  AUSTRIA |

## Belgium

**Coordinating Investigators:**

<None Entered>

| **Center** | **Principal Investigator** | **Co-Investigator(s)** | **Sub-Investigator(s)** | **Address(es)** | **Institutional Review Board or Ethics Committee Address(es)** |
| --- | --- | --- | --- | --- | --- |
|  |  |  |  |  |  |
| 1073 | Dr. Filip Van den Bosch |  | Dr. Mieke Devinck  Dr. Vanessa Smith | Universitair Ziekenhuis Gent  Reumatologie  De Pintelaan 185  Gent, 9000  BELGIUM | Ethisch Comité UZ Gent  2 P4  De Pintelaan 185  Gent, 9000  BELGIUM |

## Brazil

**Coordinating Investigators:**

<None Entered>

| **Center** | **Principal Investigator** | **Co-Investigator(s)** | **Sub-Investigator(s)** | **Address(es)** | **Institutional Review Board or Ethics Committee Address(es)** |
| --- | --- | --- | --- | --- | --- |
|  |  |  |  |  |  |
| 1039 | Dr. Cristiano A. Zerbini |  | Dr. Wagner Ikehara  Dr. Maria J. Nunes  Dr. Luiza Helena Ribeiro  Dr. Mariana G. Waisberg  Dr. Raissa Gomes Silva | Hospital Heliópolis - PAM  Serviço de Reumatologia  Rua Almirante Delamare, 1534  São Paulo, SP 04230-000  BRAZIL | Comite de Etica em Pesquisa do Complexo Hospitalar Heliopolis  Rua Conego Xavier, 276  São Paulo, SP 04231-030  BRAZIL |
|  |  |  |  |  |  |
| 1040 | Sebastiao C. Radominski |  | Andreas Funke  Maicon N. Loureiro  Alexandre G. Tavares  David Cezar Titton | Centro de Estudos do Hospital de Clínicas da UFPR  Rua Padre Camargo, 241  Alto da Glória  Curitiba, PR 80060-240  BRAZIL | Comitê de Ética em Pesquisa do Hospital de Clínicas da Universidade Federal do Paraná - HC UFPR  Rua General Carneiro, 181  Curitiba, PR 80060-900  BRAZIL |
|  |  |  |  |  |  |
| 1044 | Dr. Flora M. Marcolino |  | Edna S. Silva | SECONCI  Avenida Francisco Matarazzo, 74  São Paulo, SP 05001-000  BRAZIL | Comitê de Ética em Pesquisa do SECONCI-SP  Av. Francisco Matarazzo, 74  São Paulo, SP 05001-000  BRAZIL |
|  |  |  |  |  |  |
| 1064 | Dr. Antonio C. Ximenes |  | Rafael Navarrete Fernandez  Fabia M.G.P. Oliveira  Marcelo Pimenta | Hospital Geral de Goiânia Dr. Alberto Rassi  Departamento de Reumatologia  Av. Anhanguera, 6479  Goiânia, GO 74043-110  BRAZIL  Hospital Geral de Goiânia Dr. Alberto Rassi  Rua 9 B nº 129 - Sala: 701  Setor Oeste  Goiânia, GO 74110-120  BRAZIL | Comitê de Ética em Pesquisa Humana e Animal (CEPHA-HGG)  Hospital Geral de Goiânia Dr. Alberto Rassi  Av. Anhanguera, 6479  Goiânia, GO 74043-110  BRAZIL |

## Canada

**Coordinating Investigators:**

<None Entered>

| **Center** | **Principal Investigator** | **Co-Investigator(s)** | **Sub-Investigator(s)** | **Address(es)** | **Institutional Review Board or Ethics Committee Address(es)** |
| --- | --- | --- | --- | --- | --- |
|  |  |  |  |  |  |
| 1049 | Dr. Majed Khraishi |  | Dr. Michelle Young  Dr. Boyd Goodyear | St. Clare's Mercy Hospital  154 Lemarchand Road  Saint John's, NL A1C 5B8  CANADA | Human Investigations Committee  Human Investigations Committee  Room 1755 Health Sciences Centre  300 Prince Phillip Drive  St. John's, NL A1B 3V6  CANADA |
|  |  |  |  |  |  |
| 1050 | Milton F. Baker |  | Dr. J. Paul de Champlain | Office of Dr. Milton F. Baker  Suite 218  3930 Shelbourne Street  Victoria, BC V8P 5P6  CANADA | IRB Services  Suite 300  372 Hollandview Trail  Aurora, ON L4G 0A5  CANADA |
|  |  |  |  |  |  |
| 1053 | Dr. H. Niall Jones |  | Dr. Dalton E. Sholter  Dr. Alexander Yan | Rheumatology Research Associates Group  10839 124th Street  Edmonton , AB T5M 0H4  CANADA | College of Physicians and Surgeons  900 Manulife Place  10180-101st Street  Edmonton, AB T5J 4P8  CANADA |
|  |  |  |  |  |  |
| 1056 * | Dr. Janet Pope |  | Dr. Nicole le Riche | St Joseph's Health Care  268 Grosvenor Street  London, ON N6A 4V2  CANADA | University of Western Ontario  Office of Research Ethics  Room 00045  Dental Sciences Bldg.  London, ON N6A 5C1  CANADA |
|  |  |  |  |  |  |
| 1057 | Dr. Arthur Bookman |  |  | UHN-Toronto Western Hospital  EC 8-015  399 Bathurst Street  Toronto, ON M5T 2S8  CANADA | University Health Network Research Ethics Board  Hydro Building  Room 8-18  700 University Avenue, 8th Floor South  Toronto, ON M5G 1X6  CANADA |

## Germany

**Coordinating Investigators:**

<None Entered>

| **Center** | **Principal Investigator** | **Co-Investigator(s)** | **Sub-Investigator(s)** | **Address(es)** | **Institutional Review Board or Ethics Committee Address(es)** |
| --- | --- | --- | --- | --- | --- |
|  |  |  |  |  |  |
| 1048 | Prof. Dr. Hubert Nuesslein |  | Dr. Leonore Unger  Dr. Eva Wagner | Krankenhaus Friedrichstadt  1. Med. Klinik  Friedrichstrasse 41  Dresden, 01067  GERMANY | Ethikkommission bei der Saechsischen Landesaerztekammer  Schuetzenhoehe 16  Dresden, 01099  GERMANY |
|  |  |  |  |  |  |
| 1065 | Dr. Wolfgang Bolten |  | Dr. Elisabeth Waldorf-Bolten | Klaus Miehlke Klinik  Leibnitzstrasse 23  Wiesbaden, 65191  GERMANY | Ethikkommission bei der Saechsischen Landesaerztekammer  Schuetzenhoehe 16  Dresden, 01099  GERMANY |
|  |  |  |  |  |  |
| 1066 | Prof. Dr. Gerd-Ruediger Burmester |  | Dr. Jaqueline Detert | Universitaetsklinikum Charite  Med. Univ-Klinik u. Poliklinik III  Schumannstrasse 20-21  Berlin, 10098  GERMANY | Ethikkommission bei der Saechsischen Landesaerztekammer  Schuetzenhoehe 16  Dresden, 01099  GERMANY |
|  |  |  |  |  |  |
| 1067 * | Prof. Dr. Herbert Kellner |  |  | Arztpraxis Prof. Kellner  Romanstr. 9  Muenchen, 80639  GERMANY | Ethikkommission bei der Saechsischen Landesaerztekammer  Schuetzenhoehe 16  Dresden, 01099  GERMANY |
|  |  |  |  |  |  |
| 1068 | Dr. Juergen Wollenhaupt |  | Dr. Daniela Everding  Dr. Stefanie Haas  Dr. Bettina Velden  Dr. Wolfgang Winter | Allg. Krankenhaus Eilbek  Abt. Rheumatologie  Friedrichsberger Strasse 60  Hamburg, 22081  GERMANY | Ethikkommission bei der Saechsischen Landesaerztekammer  Schuetzenhoehe 16  Dresden, 01099  GERMANY |
|  |  |  |  |  |  |
| 1069 | Dr. med. Thilo Klopsch |  |  | Privat-Praxis, Rheumatologie  Wilhelm-Kuelz-Str. 15  Neubrandenburg, 17033  GERMANY | Ethikkommission bei der Saechsischen Landesaerztekammer  Schuetzenhoehe 16  Dresden, 01099  GERMANY |
|  |  |  |  |  |  |
| 1070 | Dr. Ulrich von Hinueber |  | Dr. Winfried Demary | Rheumatologische Gemeinschaftspraxis  Bahnhofsallee 3-4  Hildesheim, 31134  GERMANY | Ethikkommission bei der Saechsischen Landesaerztekammer  Schuetzenhoehe 16  Dresden, 01099  GERMANY |
|  |  |  |  |  |  |
| 1071 | Prof. Dr. Holm Haentzschel |  | Dr. Matthias Pierer  Dr. Ulf Wagner | Universitaet Leipzig, Zentrum fuer Innere Medizin  Med. Klinik und Poliklinik IV  Liebigstr. 22  Leipzig, 04103  GERMANY | Ethikkommission bei der Saechsischen Landesaerztekammer  Schuetzenhoehe 16  Dresden, 01099  GERMANY |

## Italy

**Coordinating Investigators:**

<None Entered>

| **Center** | **Principal Investigator** | **Co-Investigator(s)** | **Sub-Investigator(s)** | **Address(es)** | **Institutional Review Board or Ethics Committee Address(es)** |
| --- | --- | --- | --- | --- | --- |
|  |  |  |  |  |  |
| 1076 | Dr. CarloMaurizio Montecucco |  | Eleonora Bruschi  Dr. Roberto Caporali  Oscar Epis  Dr. Paola Rossi | Unita' Operativa di Reumatologia - I.R.C.C.S. Policlinico San Matteo  Viale Golgi, 19  Pavia, 27100  ITALY | Comitato di Bioetica I.R.C.C.S. Policlinico S.Matteo  V.le Golgi,19  pavia, 27100  ITALY |
|  |  |  |  |  |  |
| 1077 | Prof. Maurizio Cutolo |  | Maria Elena Secchi  Alberto Sulli | U.O. Clinica Reumatologica - Dipartimento di Medicina Interna  Università degli Studi di Genova  Viale Benedetto XV, 6  Genova, 16132  ITALY | Comitato Etico Dimi  Azienda Ospedaliera Ospedale S.Martino e Cliniche Universitarie Convenzionate  Universita' degli Studi di Genova  Viale Benedetto XV, 6  Genova, 16132  ITALY |
|  |  |  |  |  |  |
| 1079 | Prof. Marco Matucci Cerinic |  | Dr. Claudia Marinotti  Dr. Francesca Nacci | Universita' degli Studi Di Firenze Dipartimento di Medicina e Chirurgia  Azienda Ospedaliero Universitaria di Careggi S.O.D. Medicina Interna I e II  Scuola di Specializzazione in Reumatologia- Villa Monna Tessa  Viale Pieraccini 18  Firenze, 50139  ITALY | Comitato Etico per la Sperimentazione Clinica dei Farmaci- Azienda Ospedaliera Careggi  V.le Pieraccini,17  Firenze, 50139  ITALY |

## Mexico

**Coordinating Investigators:**

<None Entered>

| **Center** | **Principal Investigator** | **Co-Investigator(s)** | **Sub-Investigator(s)** | **Address(es)** | **Institutional Review Board or Ethics Committee Address(es)** |
| --- | --- | --- | --- | --- | --- |
|  |  |  |  |  |  |
| 1031 | Dr. Ignacio Garcia-De La Torre |  | Dr. Claudia Palafox  Dr. Miguel Trujillo  Dr. Vera Valladares | Centro de Estudios de Investigación Básica y Clínica SC.  Justo Sierra No. 2821-4  Col. Vallarta Norte  Guadalajara, Jalisco 44690  MEXICO | Comité de Etica Independiente del Centro de Estudios de Investigación Básica y Clínica SC  Justo Sierra No. 2821-4  Col. Vallarta Norte  Guadalajara, Jalisco 44690  MEXICO |
|  |  |  |  |  |  |
| 1032 | Dr. Virginia Pascual |  | Dr. Marina Rull-Gabayet | Instituto Nacional de Ciencias Medicas y Nutricion Dr Salvador Zubiran  Departamento de Inmunologia y Reumatologia  Vasco de Quiroga No 15  Tlalpan Seccion 16, DF 14000  MEXICO | Comité Institucional de Investigación Biomédica y Estudios en Humanos  Instituto Nacional de Ciencias Médicas y Nutrición Dr. Salvador Zubirán  Vasco de Quiroga #15  Tlalpan  México, DF 14000  MEXICO |
|  |  |  |  |  |  |
| 1033 | Dr. Ruben Burgos-Vargas |  | Dr. Virginia Alvarado-Romano  Adriana Calzada  Dr. Leticia Lino | Hospital General de México  Servicio de Reumatología  Dr. Balmis No. 148  Col. Doctores  México, D.F. 06726  MEXICO | Comisión de Etica  Hospital General de México  Dr. Balmis No. 148  Col. Doctores  México, D.F. 06726  MEXICO  Comisión de Investigación  Hospital General de México  Dr. Balmis No. 148  Col. Doctores  México, D.F. 06726  MEXICO |
|  |  |  |  |  |  |
| 1034 | Dr. Carlos Abud-Mendoza |  | Dr. Enrique Cuevas-Orta  Dr. Ricardo Moreno-Valdes  Dr. Gregorio Navarro-Cano  Dr. Martin Saldana-Barnad | Hospital Central Dr. Ignacio Morones Prieto  Unidad Regional de Reumatología y Osteoporosis  Av. Venustiano Carranza No. 2395  Col. Zona Universitaria  San Luis Potosí , San Luis Potosí 78240  MEXICO | Comité de Investigación y Ética del Hospital Central "Dr. Ignacio Morones Prieto"  Enseñanza e Investigación  Av. Venustiano Carranza No. 2395  San Luis Potosi, San Luis Potosi  MEXICO |
|  |  |  |  |  |  |
| 1035 | Dr. Guillermo F. Huerta-Yanez |  | Dr. Mario A. Chavez-Lopez  Dr. Victor A. Gallaga-Gutierrez | Hospital Miguel Hidalgo  Galeana Sur 465  Colonia Obraje  Aguascalientes, Aguascalientes 20230  MEXICO | Comité de Enseñanza, Investigación, Capacitación, Etica y Admisión  Galeana Sur 465  Colonia Obraje  Aguascalientes, Aguascalientes 20230  MEXICO |

## Slovakia

**Coordinating Investigators:**

Dr. Karim Benhatchi

Kvetoslava Greguskova

| **Center** | **Principal Investigator** | **Co-Investigator(s)** | **Sub-Investigator(s)** | **Address(es)** | **Institutional Review Board or Ethics Committee Address(es)** |
| --- | --- | --- | --- | --- | --- |
|  |  |  |  |  |  |
| 1086 | Dr. Jozef Lukac |  | Dr. Olga Lukacova | Narodny ustav reumatickych chorob  ul.I.Krasku 4  Piestany, 921 01  SLOVAKIA | Eticka komisia pri Narodnom ustave reumatickych chorob  ul.I.Krasku 4  Piestany, 921 01  SLOVAKIA |
|  |  |  |  |  |  |
| 1087 | Dr. Anna Sabova |  |  | Reumatologicka ambulancia  ul.M.R.Stefanika 187/177b  Piestany, 921 01  SLOVAKIA | Eticka komisia pri Presovskom samospravnom kraji  Presovsky samospravny kraj, odbor zdravotnictva  nam.Mieru 2,  Presov, 080 01  SLOVAKIA |
|  |  |  |  |  |  |
| 1088 | Pavol Polak |  |  | Nestatna reumatologicka ambulancia  ul. V.Spanyola 43  Zilina, 012 07  SLOVAKIA | Eticka komisia pri Zilinskom samospravnom kraji  Zilinsky samospravny kraj, odbor zdravotnictva  Komenskeho ul.48  Zilina, 011 09  SLOVAKIA |
|  |  |  |  |  |  |
| 1089 | Zelmira Macejova |  | Dr. Maria Oetterova | Fakultna nemocnica L.Pasteura, pracovisko tr.SNP, I.interna klinika  trieda SNP 1  Kosice, 040 11  SLOVAKIA | Eticka komisia Fakultna nemocnica L.Pasteura  Fakultna nemocnica L.Pasteura  Rastislavova 43  Kosice, 040 00  SLOVAKIA |

## Spain

**Coordinating Investigators:**

<None Entered>

| **Center** | **Principal Investigator** | **Co-Investigator(s)** | **Sub-Investigator(s)** | **Address(es)** | **Institutional Review Board or Ethics Committee Address(es)** |
| --- | --- | --- | --- | --- | --- |
|  |  |  |  |  |  |
| 1058 | Dr. Juan Gomez Reino |  | Juan Amarelo Ramos  Juan Garcia Meijide  Myriam Liz  Dr. Antonio Mera Varela  Susana Trincado Lopez | Hospital Clinico Universitario Santiago de Compostela  Servico de Reumatologia  C/ Choupana, s/n  Santiago de Compostela, La Coruña 15706  SPAIN | Servicio Galego de Saude (SERGAS)  Ethics Committee of Clinic Investigation  Edificio Administrativo San Lazaro, s/n  Santiago de Compostela, La Coruña 15703  SPAIN |
|  |  |  |  |  |  |
| 1059 | Dr. Jesus Tornero Molina |  | Rosa del Castillo  Manuel Fernandez Prada  Dr. Jose Antonio Piqueras  Dr. Javier Vidal Fuentes  Sara Zarzoso Hernandez | Hospital General Universitario de Guadalajara  Servicio de Reumatologia  Avda. Donantes de Sangre, s/n  Guadalajara, Guadalajara 19002  SPAIN | Hospital General Universitario de Guadalajara  Ethics Committee of Clinic Investigation  Avda Donantes de Sangre, S/N  Guadalajara, 19002  SPAIN |
|  |  |  |  |  |  |
| 1060 | Dr. JORDI CARBONELL |  | Juan Maymo  Isabel Padro  CAROLINA PEREZ  SILVIA SANCHEZ | HOSPITAL DEL MAR  SERVICIO DE REUMATOLOGIA  PASSEIG MARITIM, 25-29  BARCELONA, BARCELONA 08003  SPAIN | Instituto Municipal de Asistencia Sanitaria  Ethics Committee of Clinic Investigation  C/ Dr. Aiguader, 80  Barcelona, 08003  SPAIN |
|  |  |  |  |  |  |
| 1061 | Dr. Juan Sanchez Burson |  | Rocio Lara Aliaga | Hospital Universitario de Valme  Servico de Reumatologia  Ctra de Cadiz Km 548.9  Sevilla, Sevilla 41014  SPAIN | Comite Autonomico de Ensayos Clinicos de Andalucia  Ethics Committee of Clinic Investigation  Avda. de la Innovación, s/n  Edificio Arena, 1  Sevilla, Sevilla 41020  SPAIN |
|  |  |  |  |  |  |
| 1062 * | Dr. JOSE VALVERDE |  | Francisco Javier Narvaez Garcia | HOSPITAL UNIVERSITARIO DE BELLVITGE  SERVICIO DE REUMATOLOGIA  C/ FEIXA LLARGA, S/N  L´HOSPITALET DE LLOBREGAT, BARCELONA 08907  SPAIN | Ciutat Sanitaria i Universitaria de Bellvitge  Ethics Committee of Clinical Investigation  C/ Feixa Llarga, s/n  Secretaría de Docencia e Investigación  08907 L'Hospitalet de Llobregat (Barcelona)  SPAIN |
|  |  |  |  |  |  |
| 1063 | Dr. EMILIO MARTIN MOLA |  | Dr. Pilar Aguado  ALEJANDRO BALSA CRIADO  Miguel Bernad  Carmen De Aysa  Dr. Eugenio De Miguel  Carlos Perez de Ayala  Mª Carmen Rodriguez  Mª Encarnacion Roncal | HOSPITAL UNIVERSITARIO LA PAZ  SERVICIO DE REUMATOLOGIA  PASEO DE LA CASTELLANA, 261  MADRID, MADRID 28046  SPAIN | Hospital Universitario La Paz  Ethics Committee of Clinic Investigation  Pº de la Castellana, 261  Escuela de enfermeria, Planta 4º, Despacho 424  Madrid, 28046  SPAIN |

## United States

**Coordinating Investigators:**

<None Entered>

| **Center** | **Principal Investigator** | **Co-Investigator(s)** | **Sub-Investigator(s)** | **Address(es)** | **Institutional Review Board or Ethics Committee Address(es)** |
| --- | --- | --- | --- | --- | --- |
|  |  |  |  |  |  |
| 1001 | Dr. Eugene Patrick Boling |  | Ms. Patricia E. DesLauriers  Dr. Eric T. Lee  Dr. Mohamed Bassam Sebai | Boling Clinical Trails (BCT)  Suite 302  510 North 13th Avenue  Upland, CA 91786  UNITED STATES  Inland Rheumatology and Osteoporosis Medical Group  Suite 204  548 North 13th Avenue  Upland, CA 91786  UNITED STATES  Inland Rheumatology and Osteporosis Medical Group  Suite 306  548 North 13th Ave  Upland, CA 91786  UNITED STATES | WIRB  P O Box 12029  3535 7th Avenue Southwest  Olympia, WA 98502  UNITED STATES |
|  |  |  |  |  |  |
| 1002 | Dr. Stephen Allan Bookbinder |  | Mr. Arthur Elkins | Ocala Rheumatology Research Center  Suite 102  3210 Southwest 33rd Road  Ocala, FL 34474-7455  UNITED STATES | WIRB  P O Box 12029  3535 7th Avenue Southwest  Olympia, WA 98502  UNITED STATES |
|  |  |  |  |  |  |
| 1003 | Dr. Jane Herron Box |  | Dr. John Franklyn Babich  Dr. Patrick N. Box  Dr. Ashrito Kumar Dayal  Dr. William Bryant Gruhn | Arthritis Clinic & Carolina Bone & Joint, PA  Suite 100  10430 Park Road  Charlotte, NC 28210  UNITED STATES | WIRB  P O Box 12029  3535 7th Avenue Southwest  Olympia, WA 98502  UNITED STATES |
|  |  |  |  |  |  |
| 1005 | Dr. Ronald L. Collins |  | Dr. Kathleen Patricia Flint | Columbia Arthritis Center  1711 St. Julian Place  Columbia, SC 29204  UNITED STATES | WIRB  P O Box 12029  3535 7th Avenue Southwest  Olympia, WA 98502  UNITED STATES |
|  |  |  |  |  |  |
| 1006 | Dr. Andres Quiceno  Dr. John Joseph Cush (Previous PI) |  | Dr. Kathryn Dao | Presbyterian Hospital of Dallas, Arthritis Consultation Center  8200 Walnut Hill Lane  Dallas, TX 75231-4496  UNITED STATES | Presbyterian Hospital of Dallas  Institutional Review Board  8200 Walnut Hill Avenue  Dallas, TX 75231  UNITED STATES  WIRB  P O Box 12029  3535 7th Avenue Southwest  Olympia, WA 98502  UNITED STATES |
|  |  |  |  |  |  |
| 1007 | Dr. Robert Emil Ettlinger |  | Dr. George Howard Krick  Teresa A. Unkrur | Tacoma Center for Arthritis Research, PS  1901 South Cedar #204  1901 South Cedar #201  Tacoma, WA 98405  UNITED STATES | Western Institutional Review Board  PO Box 12029  3535 Seventh Avenue, Southwest  Olympia, WA 98508  UNITED STATES |
|  |  |  |  |  |  |
| 1010 | Dr. Alan Jan Kivitz  Dr. Shelly P. Kafka (Previous PI) |  | Ms. Marissa L. Dudeck  Ms. Leslie A. Krug  Dr. Frederick Timothy Murphy  Ms. Debra L. Rentz  Ms. Sharon K. Ritchey  Dr. Marianne L. Shaw  Dr. Vicki Marlene Sommer  Mr. Michael Joseph Zumer  Ms. Angela Marie Zumer-Braatz | Altoona Center for Clinical Research  1125 Old Route 220 North  Ducansville, PA 16635  UNITED STATES  Altoona Center for Clinical Research  336 Bloomfield Street  Johnstown, PA 15904  UNITED STATES | WIRB  P O Box 12029  3535 7th Avenue Southwest  Olympia, WA 98502  UNITED STATES |
|  |  |  |  |  |  |
| 1011 | Dr. Reynold Michael Karr Jr. |  | Ms. Diana L. Lachman  Mr. Aaron G. Misiuk  Dr. Andrew Seunghan Sohn | Reynold M. Karr, Jr., MD  Physician's Pharmaceutical Study Services  3128 Norton  Everett, WA 98201  UNITED STATES | WIRB  P O Box 12029  3535 7th Avenue Southwest  Olympia, WA 98502  UNITED STATES |
|  |  |  |  |  |  |
| 1012 | Dr. Clarence W. Legerton III |  | Dr. William Milnes Edwards  Dr. Gary Eliot Fink  Dr. Gregory William Niemer | Low Country Research  2860 Tricom Street  Charleston, SC 29406  UNITED STATES | Western Institutional Review Board  PO Box 12029  3535 Seventh Avenue, Southwest  Olympia, WA 98508  UNITED STATES |
|  |  |  |  |  |  |
| 1014 | Dr. Jeffrey Edward Poiley |  |  | Arthritis Associates  324 East Par Avenue  Orlando, FL 32804  UNITED STATES | WIRB  P O Box 12029  3535 7th Avenue Southwest  Olympia, WA 98502  UNITED STATES |
|  |  |  |  |  |  |
| 1017 | Dr. Jaime A. Pachon  Dr. Eric Andrew Sheldon (Previous PI) |  | Adriana Acosta  Ms. Ivonne G. Arean  Ms. Isabel Mercedes Pino  Julio Regalado Jr.  Dr. Howard I. Schwartz  Ms. Carleen Demshok Parlato | Arthritis and Rheumatic Care Center  Suite 201  7500 SW 87 Avenue  Miami, FL 33173  UNITED STATES  Miami Research Associates  Suite 202  7500 SW 87th Avenue  Miami, FL 33173  UNITED STATES | Western Institutional Review Board  PO Box 12029  3535 Seventh Avenue, Southwest  Olympia, WA 98508  UNITED STATES |
|  |  |  |  |  |  |
| 1018 | Dr. Joel Charles Silverfield |  | Dr. Rafael Antonio Blasini  Dr. Michael Claude Burnette  Dr. Harris Hugh McIlwain | Tampa Medical Group, PA  Suite 303 and 201  4700 North Habana Avenue  Tampa, FL 33614  UNITED STATES | WIRB  P O Box 12029  3535 7th Avenue Southwest  Olympia, WA 98502  UNITED STATES |
|  |  |  |  |  |  |
| 1020 | Dr. S. Bobo Tanner |  | Dr. Valerie S. Chen  Dr. David Daniel Hagaman  Dr. Eva Kathryn Miller  Dr. John Joseph Murray  Dr. John Michael Norvell  Christy Sparkman | Vanderbilt Asthma Sinus Allergy Program  Suite 120  2611 West End Avenue  Nashville, TN 37203  UNITED STATES | Vanderbilt University Institutional Review Board  D-3232 MCN  1161 21st Ave. South  Nashville, TN 37232-2598  UNITED STATES  WIRB  P O Box 12029  3535 7th Avenue Southwest  Olympia, WA 98502  UNITED STATES |
|  |  |  |  |  |  |
| 1021 | Dr. James Matthew Trice |  |  | Arthritis, Osteoporosis, and Musculoskeletal Disease Center  280 Pleasant Street  Concord, NH 03301  UNITED STATES | Concord Hospital Human Investigation Committee  Concord Hospital Human Investigation Committee  Concord Hospital  250 Pleasant Street  Concord, NH 03301  UNITED STATES  WIRB  P O Box 12029  3535 7th Avenue Southwest  Olympia, WA 98502  UNITED STATES |
|  |  |  |  |  |  |
| 1023 | Dr. Sanford Mayer Wolfe |  |  | STAT Research, Inc.  Suite 544  111 West First Street  Dayton, OH 45402  UNITED STATES | WIRB  P O Box 12029  3535 7th Avenue Southwest  Olympia, WA 98502  UNITED STATES |
|  |  |  |  |  |  |
| 1024 | Dr. Farrukh Zaidi |  | Margaret S. Allen  Ms. Lynne E. Merriam  Dr. Greg Marc Silver | Clinical Research of West Florida, Inc.  2147 Northeast Coachman Road  Clearwater, FL 33765  UNITED STATES  Excel Medical Imaging  5626 Gulf Drive  New Port Richey, FL 34652  UNITED STATES  Florida Arthritis and Osteoarthritis Center  8029 Washington Street  Port Richey, FL 34668  UNITED STATES | WIRB  P O Box 12029  3535 7th Avenue Southwest  Olympia, WA 98502  UNITED STATES |
|  |  |  |  |  |  |
| 1026 | Dr. Mark William Niemer |  | Ms. Leanne Johnson-Meeter  Dr. Steven Rock | Medical Associates Clinic  1000 Langworthy St  Dubuque, IA 52001-7313  UNITED STATES  Medical Associates Clinic  1500 Associates Drive  Dubuque, IA 52002  UNITED STATES | WIRB  P O Box 12029  3535 7th Avenue Southwest  Olympia, WA 98502  UNITED STATES |
|  |  |  |  |  |  |
| 1028 | Dr. Dayton Dennis Payne Jr. |  | Dr. Carlos A. De La Garza  Dr. Michael C. DeSantis  Dr. John Keith Earl  Dr. Robert Frederick Glenn  Dr. James Robinson Hodges  Dr. Baxter Columbus Leonard  Debora S. Runfola  Dr. William Cecil Thompson III | Crown Health Care  Medical Arts Clinic  24 2nd Avenue NE  Hickory, NC 28601  UNITED STATES  Hickory Family Practice  52 12th Avenue NE  Hickory, NC 28601  UNITED STATES  Piedmont Rheumatology, PA  230 18th Street Circle SE  Hickory, NC 28602  UNITED STATES  Unifour Medical Research Associate  1036 2nd Street, NE  Hickory, NC 28601  UNITED STATES | WIRB  P O Box 12029  3535 7th Avenue Southwest  Olympia, WA 98502  UNITED STATES |
|  |  |  |  |  |  |
| 1042 | Dr. Prem Chatur Chatpar |  | Dr. Sunil D. Aggarwal  Thomas Cipolla  Dr. Inna Livitz  Ms. Barbara Scandariato | Prem C. Chatpar, MD  524 Old Country Road  Plainview, NY 11803  UNITED STATES | WIRB  P O Box 12029  3535 7th Avenue Southwest  Olympia, WA 98502  UNITED STATES |
