# Supplementary material for: Changes in serum creatinine in patients with active rheumatoid arthritis treated with tofacitinib: results from clinical trials
Source: Arthritis Res Ther. 2014 Jul 25;16(4):R158. doi: 10.1186/ar4673 (PMC4220634; doi:10.1186/ar4673)
Supplement: Supplementary file 2 — Additional file 2: List of Investigators and Corresponding Ethics Committees or Institutional Review Boards for the LTE A3921024 study. (DOC 1 MB) [file 13075_2013_4378_MOESM2_ESM.doc]

# 16.1.4.1 LIST OF INVESTIGATORS AND CORRESPONDING ETHICS COMMITTEES OR INSTITUTIONAL REVIEW BOARDS

## Argentina

**Coordinating Investigators:**

<None Entered>

| **Center** | **Principal Investigator** | **Co-Investigator(s)** | **Sub-Investigator(s)** | **Address(es)** | **Institutional Review Board or Ethics Committee Address(es)** |
| --- | --- | --- | --- | --- | --- |
|  |  |  |  |  |  |
| 1080 | Dr. Guillermo A. Tate |  | Paula Bruni  Dr. Mariana Caubet  Natalia Caviglia  Dr. Mariel Susana Cutri  Dr. Claudia Andrea Helling  Dr. Eduardo Fabian Mysler  Dr. Anastasia Secco  Dr. Maria Natalia Tamborenea  Patricio Tate | OMI - Organizacion Medica de Investigacion  Uruguay 725, P.B.  Buenos Aires, C1015ABO  ARGENTINA | Comite Independiente de Etica (CIE) para Ensayos en Farmacologia Clinica  Fundación de Estudios Farmacologicos y de Medicamentos (FEFyM)  J.E. Uriburu 774- Piso 1°  Buenos Aires, C1027AAP  ARGENTINA  OMI Comite de Docencia e Investigacion  Uruguay 725, PB  Buenos Aires, C1013AAR  ARGENTINA |
|  |  |  |  |  |  |
| 1081 | Dr. Oscar Luis Rillo |  | Dr. Cecilia Elena Borlenghi  Dr. Luciana Casalla  Dr. Maria Alejandra Cusa  Dr. Roxana Dora De Salvo  Dra. Ana Maria Freuler  Dr. Alejandro Martinez Munoz  Dr. Silvia Papasidero  Maria Cristina Rillo Pratto  Dr. Lorena Salinas | Centro Médico Dra. De Salvo  Av. Cabildo 1548, 1A  Buenos Aires, C1426ABP  ARGENTINA  Hospital Sirio Libanes  Campana 4658, Second Floor  Buenos Aires, C1419AHN  ARGENTINA | Comite de Docencia e Investigacion del Hospital Sirio Libanes  Campana 4658  Buenos Aires, C1419AHN  ARGENTINA  Comite de Etica en Investigacion del Hospital Sirio Libanes  Campana 4658  Buenos Aires, C1419AHN  ARGENTINA  Comite Independiente de Etica (CIE) para Ensayos en Farmacologia Clinica  Fundación de Estudios Farmacologicos y de Medicamentos (FEFyM)  J.E. Uriburu 774- Piso 1°  Buenos Aires, C1027AAP  ARGENTINA |
|  |  |  |  |  |  |
| 1082 | Dr. Osvaldo Daniel Messina |  | Dr Edgardo Enrique Alvites Guerrero  Dr. Fabiana Geliberti  Dr. Maria Jose Gentile  Raquel Isabel Ramenzoni  Dr. Silvina Edith Suarez | Investigaciones Reumatologicas y Osteologicas (IRO)  J.E. Uriburu 1170, 1 Piso  Buenos Aires, C1114AAH  ARGENTINA | Comite de Docencia e Investigacion C.I.R.O.  Larrea 1106 - 4°B  Capital Federal, Buenos Aires C1117ABH  ARGENTINA  Comite de Etica para ensayos en farmacologia clinica "Investigaciones Reumatologicas y Osteologicas"  11 de Septiembre 2231, 7 A  Buenos Aires, C1428AII  ARGENTINA  Comite Independiente de Etica (CIE) para Ensayos en Farmacologia Clinica  Fundación de Estudios Farmacologicos y de Medicamentos (FEFyM)  J.E. Uriburu 774- Piso 1°  Buenos Aires, C1027AAP  ARGENTINA |
|  |  |  |  |  |  |
| 1083 | Dr. Mariano Ricardo Ghirlanda |  | Dr. Pablo De Caso  Dra. Ana Maria Freuler  Dr. Sebastian Rossi Lopez  Dr. David Gustavo Starosiliz | Saint Dennis Medical Group S.A.  Av. Rivadavia 2206 6 A  Buenos Aires, C1034ACO  ARGENTINA | Comite de Investigacion y Etica de Saint Dennis Medical Group S. A.  Av. Rivadavia 2206- 6°A  Buenos Aires, C1034ACO  ARGENTINA  Comite Independiente de Etica (CIE) para Ensayos en Farmacologia Clinica  Fundación de Estudios Farmacologicos y de Medicamentos (FEFyM)  J.E. Uriburu 774- Piso 1°  Buenos Aires, C1027AAP  ARGENTINA |
|  |  |  |  |  |  |
| 1614 | Dr. Gustavo Citera |  | Dr. Maria de los Angeles Correa  Dr. Fernando Martin Dal Pra  Dr. Maria V. Lencina  Dr. Jose A. Maldonado Cocco  Dr. Rodolfo S. Perez Alamino  Dr. Marcos Gabriel Rosemffet  Dr. Silvina L. Serra  Fernando Andres Sommerfleck  Dr Christian Waimann | Consultorios Reumatológicos Pampa  La Pampa 1548 1°A  Buenos Aires, C1428DZF  ARGENTINA | Comite Independiente de Etica (CIE) para Ensayos en Farmacologia Clinica  Fundación de Estudios Farmacologicos y de Medicamentos (FEFyM)  J.E. Uriburu 774- Piso 1°  Buenos Aires, C1027AAP  ARGENTINA |
|  |  |  |  |  |  |

## Australia

**Coordinating Investigators:**

<None Entered>

| **Center** | **Principal Investigator** | **Co-Investigator(s)** | **Sub-Investigator(s)** | **Address(es)** | **Institutional Review Board or Ethics Committee Address(es)** |
| --- | --- | --- | --- | --- | --- |
|  |  |  |  |  |  |
| 1262 | Assoc. Prof. Peter Thomas Nash |  | Dr. Frances Anne Johnson  Dr. David William Nicholls  Ms. Dale Shergold  Ms. Jan C. Smith  Dr. Susan L. Thackwray  Dr. Avgeania Louisa Voight | Rheumatology Research Unit Sunshine Coast  9-10 Maroochy Waters Shopping Centre  Denna Street  Maroochydore, QLD 4558  AUSTRALIA | Bellberry Limited Human Research Ethics Committee  229 Greenhill Road  Dulwich, SA 5065  AUSTRALIA  Redcliffe-Caboolture Ethics Committee  Unit 1, Ground Floor, Redcliffe Hospital  Anzac Avenue  Redcliffe, Qld 4020  AUSTRALIA |
|  |  |  |  |  |  |
| 1263 | Dr. Maureen Rischmueller |  | Dr. Rachel Black  Dr. Simon Patrick Burnet  Fin Cai  Jien Ni Cheng  Dr. Sarah Downie-Doyle  Dr. Scott Graf  Dr. Catherine Hill  Dr. Veera Katikireddi  Suman Murthy  Dr. Jem Ninan  Dr. Kimberly Hui Jin Ting  Dr. Samuel Lawrence Whittle | The Queen Elizabeth Hospital, Department of Rheumatology  28 Woodville Road  Woodville, SA 5011  AUSTRALIA | Central Northern Adelaide Health Service  Ethics of Human Research Committee  The Queen Elizabeth Hospital  28 Woodville Road  Woodville, SA 5011  AUSTRALIA |
|  |  |  |  |  |  |
| 1264 | Dr. Stephen Hall |  | Dr. Vivienne Beckett  Dr Jill Bell  Dr Malcolm Clark  Dr Jennifer Mary Davey  Ms Stephanie Dolejs  Dr. Marie Feletar  Dr. Andrew Gibson  Gail Grant  Dr. James McDonald  Dr Nicole McKay  Dr Louise Murdoch  Dr. Janene Richards | Emeritus Research  291 Wattletree Road  Malvern East, VIC 3145  AUSTRALIA | Cabrini Human Research Ethics Committee  183 Wattletree Rd,  Malvern, VIC , Australia 3144  AUSTRALIA |
|  |  |  |  |  |  |
| 1271 | Dr. David H. Bossingham |  | Karen Cooke  Dr. Joshua P. Hanson  Kathryn Leonard | Rheumatology Research  5th Floor Block B Cairns Base Hospital  The Esplanade  Cairns, QLD 4870  AUSTRALIA | Cairns and Hinterland Health Service District Human Research Ethics Committee  4th Floor, Block A  Cairns Base Hospital  Cairns, QLD 4870  AUSTRALIA |
|  |  |  |  |  |  |
| 1272 | A/Prof. Leslie Schrieber |  | Dr. Mark Arnold  Ms. Lyndall Henderson | Pharmacy Department  Level 1  Acute Services Building  New Royal North Shore Hopital  St. Leonards, NSW 2065  AUSTRALIA  Pharmacy Department (W)  Royal North Shore Hospital  St. Leonards, NSW 2065  AUSTRALIA  Royal North Shore Hospital  Level 7, Clinical Administration 7C  Acute Services Building (NEW RNSH BUILDING)  St Leonards, NSW 2065  AUSTRALIA | Northern Sydney / Central Coast Area Health Service HREC  Human Research Ethics Committee, Royal North Shore Hospital  Pacific Hwy  St Leonards, NSW 2065  AUSTRALIA |
|  |  |  |  |  |  |
| 1273 | Dr. Nicola Cook  Madelynn Chan (Previous PI) |  | Ms. Maureen Blore  Dr. Shereen Suyin Ch'ng  Madelynn Chan  Priya Chowalloor  Margaret Crowe  Dr. Ariadna Cuiesdean  Dr Andreea Harsanyi  Hope Kifuso  Yuen Leow  Andrew Lim  Christa Makin  Krista Makin  Dr. Shereen Paramalingam  Ms. Margaret Rogers  Swagat Shrestha  Hasib Sidiqi  Dr. Andrew Taylor  Dr. Ai Phuong Tran | Goatcher Clinical Research Unit Royal Perth Hospital Shenton Park Campus  6 Selby Street  Shenton Park, WA 6008  AUSTRALIA | Royal Perth Hospital Ethics Committee,  Royal Perth Hospital  Wellington Street Campus  Wellington Street  Perth, WA 6000  AUSTRALIA |
|  |  |  |  |  |  |
| 1577 | Dr. Paul A. Bird |  | Sharon Dunkley  Wendy Gellatley  Deanne Jenkin  Dr. Fredrick F. Joshua  Dr. Louis E. McGuigan | Optimus Clinical Research Pvt. Ltd.  Suite 4, Level 1  19 Kensington Street  Kogarah,  AUSTRALIA | Bellberry Limited Human Research Ethics Committee  229 Greenhill Road  Dulwich, SA 5065  AUSTRALIA |
|  |  |  |  |  |  |

## Austria

**Coordinating Investigators:**

<None Entered>

| **Center** | **Principal Investigator** | **Co-Investigator(s)** | **Sub-Investigator(s)** | **Address(es)** | **Institutional Review Board or Ethics Committee Address(es)** |
| --- | --- | --- | --- | --- | --- |
|  |  |  |  |  |  |
| 1041 | Dr. Joerg Dietmar Rieger |  | Elke Boettcher  Dr. Omid Zamani | Rheuma-Ordination Favoriten  Quellenstrasse 181  Wien, A-1100  AUSTRIA | Ethikkommission der Stadt Wien  Geschäftsführung: Gesundheitswesen und Soziales (MA 15), 3. Stock, Zimmer 304 bis 306  Neutorgasse 15  Wien, A-1013  AUSTRIA  Ethikkommission der Stadt Wien  Geschaeftsfuehrung: Gesundheitsdienst der Stadt Wien (MA 15),TownTown  Thomas-Klestil-Platz 8  Wien, A-1030  AUSTRIA  Ethikkommission der Stadt Wien  Geschaeftsfuehrung: Gesundheitsdienst der Stadt Wien (MA 15), TownTown  Thomas-Klestil-Platz 8  Wien, A-1030  AUSTRIA |
|  |  |  |  |  |  |
| 1588 | Prim. Univ. Doz. Dr. Ludwig Erlacher |  | Dr. Sonja Brandstaetter  Lilla Hatos-Agyi  Dr. Angelika Kraus  Dr. Monika Mustak-Blagusz  Katharina Schmied | Kaiser Franz Josef Spital  Kundratstrasse 3  Wien, 1100  AUSTRIA  Sozialmedizinisches Zentrum Sued - Kaiser Franz Josef Spital  2. Medizinische Abteilung  Abteilung fuer Rheumatologie und Osteologie sowie Akutgeriatrie  Kundratstrasse 3  Wien, A-1100  AUSTRIA | Ethikkommission der Stadt Wien  gemaess KAG, AMG und MPG  Town Town  Thomas-Klestil-Platz 8  Wien, A-1030  AUSTRIA |
|  |  |  |  |  |  |
| 1589 | Univ. Prof. Dr. Clemens Scheinecker |  | Dr. Michael Markus Bonelli  Dr. Miriam Gaertner  Prof. Dr. Klaus Peter Machold | Medizinische Universitaet Wien / AKH  Universitaetsklinik fuer Innere Medizin III  Klinische Abteilung fuer Rheumatologie  Waehringer Guertel 18-20  Wien, A-1090  AUSTRIA | Ethikkommission der Stadt Wien  gemaess KAG, AMG und MPG  Town Town  Thomas-Klestil-Platz 8  Wien, A-1030  AUSTRIA  Ethikkommission der Stadt Wien  Geschaeftsfuehrung: Gesundheitsdienst der Stadt Wien (MA 15), TownTown  Thomas-Klestil-Platz 8  Wien, A-1030  AUSTRIA |
|  |  |  |  |  |  |

## Belgium

**Coordinating Investigators:**

<None Entered>

| **Center** | **Principal Investigator** | **Co-Investigator(s)** | **Sub-Investigator(s)** | **Address(es)** | **Institutional Review Board or Ethics Committee Address(es)** |
| --- | --- | --- | --- | --- | --- |
|  |  |  |  |  |  |
| 1046 | Dr. Filip Eduard Jeanne Van den Bosch |  | Dr. Philippe Carron  Dr. Vanessa Smith  Dr. Ruth Wittoek | Universitair Ziekenhuis Gent - Reumatologie  De Pintelaan 185  Gent, 9000  BELGIUM | Universitair Ziekenhuis Gent, Ethisch Comite  De Pintelaan 185  Gent, 9000  BELGIUM |
|  |  |  |  |  |  |
| 1599 | Prof. Jean-Yves Reginster |  | Dr. Marie-Paule Lecart  Dr. Nathalie Sarlet | University of Liege  Quai Godefroid Kurth 45 (9th floor)  Liege, 4020  BELGIUM | Ethisch Comite Universitair Ziekenhuis Gent  De Pintelaan 185  Gent, 9000  BELGIUM |
|  |  |  |  |  |  |
| 1600 | Dr. Klaas Vandevyvere |  | Dr. Anne Durnez | Az Groeninge Kortrijk  Burg Vercruysselaan 5  Kortrijk, 8500  BELGIUM | Ethisch Comite Universitair Ziekenhuis Gent  De Pintelaan 185  Gent, 9000  BELGIUM |
|  |  |  |  |  |  |
| 1602 * | Prof. Piet P. M. M. Geusens |  | Dr. Hubert T. Berghs  Dr. Marleen Y. Coppens  Dr. Anna Sileghem  Dr. Inge Van Tilt  Dr. Johan L.M. Vanhoof  Dr. Pascale Volders | Associatie Reumatologie  Bretheistraat 149  Genk, 3600  BELGIUM  Associatie Reumatologie  Casterstraat 42  Hasselt, 3500  BELGIUM | Ethisch Comite Universitair Ziekenhuis Gent  De Pintelaan 185  Gent, 9000  BELGIUM |
|  |  |  |  |  |  |

## Bosnia And Herzegovina

**Coordinating Investigators:**

<None Entered>

| **Center** | **Principal Investigator** | **Co-Investigator(s)** | **Sub-Investigator(s)** | **Address(es)** | **Institutional Review Board or Ethics Committee Address(es)** |
| --- | --- | --- | --- | --- | --- |
|  |  |  |  |  |  |
| 1462 | Assist.Prof. Sekib Sokolovic |  | Dr. Nirvana Sabanovic Bajramovic | University Clinical Center Sarajevo  Bolnicka 25  Sarajevo, 71000  BOSNIA AND HERZEGOVINA | Ethics Committee  Clinical Center University of Sarajevo  Bolnicka 25  Sarajevo, 71000  BOSNIA AND HERZEGOVINA |
|  |  |  |  |  |  |

## Brazil

**Coordinating Investigators:**

<None Entered>

| **Center** | **Principal Investigator** | **Co-Investigator(s)** | **Sub-Investigator(s)** | **Address(es)** | **Institutional Review Board or Ethics Committee Address(es)** |
| --- | --- | --- | --- | --- | --- |
|  |  |  |  |  |  |
| 1042 | Dr. Cristiano Augusto de Freitas Zerbini |  | Dr. Bruno Cesar Cavalcanti dos Anjos  Dr. Marta Eliane de Carvalho Bastos  Lina Oliveira de Carvalho  Dr. Andre Consalter  Jessica Cale Duarte  Ana Beatriz Andreo Garcia  Dr. Wagner Ikehara  Dr. Maria Jose Nunes  Dr. Luiz Carlos Latorre  Dr. Andrea Barranjard Vannucci Lomonte  Dr. Silvia Caroline Santana Moura  Dr. Lenise Brandao Pieruccetti  Dr. Luiza Helena Coutinho Ribeiro  Dr. Raissa Gomes da Silva  Dr. Mariana G. Waisberg | CEPIC - Centro Paulista De Investigacao  Department Of Pathology  Rua Moreira e Costa, 342  Clinica E Servicos Medicos Ltda  Sao Paulo, SP 04266-010  BRAZIL | Comite de Etica em Pesquisa em Seres Humanos do Hospital Heliopolis  Rua Conego Xavier 276  Sao Paulo, SP 04231-030  BRAZIL |
|  |  |  |  |  |  |
| 1043 | Dr. Antonio Carlos Ximenes |  | Aline Assaf Branco  Dr. Rafael Navarrete Fernandez  Camila Guimaraes  Dr. Fabia M.G.P. Oliveira  Dr. Marcelo Pimenta  Dr. Bruno Nazeozeno Ribeiro | CIP - Centro Internacional de Pesquisa  Rua 9 B, 129 - 3rd floor - Setor Oeste  Goiania, GO 74110-120  BRAZIL  CIP - Centro Internacional de Pesquisas  Rua 9 B, 129 - 3 andar  Setor Oeste  Goiania, GO 74110-120  BRAZIL  Hospital Geral de Goiânia Doutor Alberto Rassi  Rua 9 B, 129 - Sala: 303  Setor Oeste  Goiânia, GO 74110-120  BRAZIL | Comite de Etica em Pesquisa do Hospital Alberto Rassi - HGG  Av. Anhanguera, 6479 - Setor Oeste  Goiania, GO 74110-010  BRAZIL |
|  |  |  |  |  |  |
| 1044 | Dr. Flora Maria D´Andrea Marcolino |  | Dr. Eduardo S. Meirelles | Hospital das Clinicas da Faculdade de Medicina da Universidade de Sao Paulo (HC-FMUSP)  I.O.T - Instituto de Ortopedia e Traumatologia  Rua Dr. Ovidio Pires de Campos, 333  Centro de Pesquisa - 1 andar  SAO PAULO, SP 05403-010  BRAZIL | Comissao de Etica para Analise de Projetos de Pesquisa (CAPPesq)  Hospital das Clinicas e da Faculdade de Medicina da Universidade de Sao Paulo  Rua Dr. Ovidio Pires de Campos, 225, sala 505  Cerqueira Cesar  Sao Paulo, SP 05403-010  BRAZIL |
|  |  |  |  |  |  |
| 1045 | Dr. Sebastiao Cezar Radominski |  | Dr. Vivian B. Coginotti  Dr. Sinara da Silva Freitas  Dr. Andreas Funke  Dr. Alexandre Grein Tavares  Dr. Maicon N. Loureiro  Patricia Martin  Dr. Lucila Stange Rezende  Dr. David Cezar Titton | Centro de Estudos em Terapias Inovadoras  Avenida Agostinho Leao Junior, 306  Curitiba, Paraná 80030-110  BRAZIL | Comite de Etica em Pesquisa em Seres Humanos do HC-UFPR  Rua General Carneiro, 181  Curitiba, PR 80060-900  BRAZIL |
|  |  |  |  |  |  |
| 1148 | Dr. Joao Carlos Tavares Brenol |  | Dr. Claiton Viegas Brenol  Rafael Mendonça da Silva Chakr  Andrese Gasparin  Dr. Odirlei Andre Monticielo  Dr. Tamara M. Mucenic  Penelope Esther Palominos  Dr. Ricardo Machado Xavier | Hospital de Clinicas de Porto Alegre  Servico de Reumatologia - 6Âº andar - sala 645A  Rua Ramiro Barcelos, 2350  Porto Alegre, RS 90035 903  BRAZIL  Hospital de Clinicas de Porto Alegre  Servico de Reumatologia - 6º andar - sala 645A  Rua Ramiro Barcelos, 2350  Porto Alegre, RS 90035-903  BRAZIL | Comite de Etica em Pesquisa do Hospital de Clinicas de Porto Alegre - HCPA  Rua Ramiro Barcelos, 2350  Sala 2200 B - Bom Fim  Porto Alegre, RS 90035-903  BRAZIL |
|  |  |  |  |  |  |
| 1185 | Dr. Jussara de Almeida Lima Kochen |  |  | Hospital Alemao Oswaldo Cruz  Rua Joao Juliao, 331  Paraiso  Sao Paulo, SP 01323-903  BRAZIL | Comite de Etica em Pesquisa do Hospital Alemao Oswaldo Cruz  Rua Joao Juliao, 331 - 14º andar  Bela Vista  Sao Paulo, SP 01323-903  BRAZIL |
|  |  |  |  |  |  |
| 1440 | Dr. Luciana Teixeira Pinto |  | Camila Cristhine Bucchi  Dr. Marise Lazaretti Castro  Diogo Souza Domiciano  Patricia Muszkat  Dr. Camila Albero Schiavon  Jeane Jeong Hoon Yang | IMA Brasil - Instituto de Medicina Avancada  Praca Americo Jacomino, 55  Vila Madalena  Sao Paulo, SP 05437-010  BRAZIL | Comite de Etica em Pesquisa do Instituto de Infectologia Emilio Ribas  Av. Dr. Arnaldo, 165  Sao Paulo, SP 01246-900  BRAZIL |
|  |  |  |  |  |  |
| 1441 | Dr. Mauro Waldemar Keiserman |  | Dr. Melissa Claudia Bisi  Livia Martins Soares Garcia  Dr. Briele Keiserman  Dr. Tatiana Karenini Muller  Mercedes Picarelli  Dr. Deise Marcela Piovesan  Dr. Aline Defaveri do Prado  Dr. Aline de Souza Streck  Caroline Z. Xavier de Freitas | Hospital Sao Lucas da PUCRS  Av. Ipiranga, 6690 - 4 andar  Porto Alegre, RS 90610-000  BRAZIL | Comite de Etica em Pesquisa da Pontificia Universidade Catolica do Rio Grande do Sul  Av. ipiranga 6690-Conj.314 -3 andar  Jardim Botanico  Porto Alegre, RS 90610-000  BRAZIL |
|  |  |  |  |  |  |
| 1442 | Dr. Ana Claudia Cauceglia Melazzi |  | Dr. Carolina A. Cabizuca  Dr. Tonia L. Cunha  Dr. Alessandra Saldanha Matheus Fernandes Da Costa  Dr. Adriana Danowski  Dr. Maria De Fatima Dias De Castro  Dr. Joselita Maria Franca de Siqueira  Dr. Juliana Branco Dias  Dr. Luiz Henrique de Gregorio  Dr. Angela Frazao Linhares Hahn  Daniel Barretto Kendler  Dr. Paulo Gustavo Sampaio Lacativa  Adriana Barroso Lambert  Dr. Flavia S. Lessa  Izabela Medeiros de Almeida Marques  Ricardo Montes  Dr. Tarso Lameri Sant anna Mosci  Paula Paiva  Paula Paiva  Dr. Renata Alexandra Calixto Pinheiro  Dr. Luis Augusto Tavares Russo  Dr. Pedro Moitrel Schwarts  Dr. Priscila Geller Wolff | CCBR Brasil  Centro de Pesquisas e Analises Clinicas Ltda  Rua Mena Barreto, 33  Rio de Janeiro, RJ 22271-100  BRAZIL  CCBR Brasil Centro de Pesquisas e Analises Clinicas Ltda  Mena Barreto, 33  Rio de Janeiro, RJ 22271-100  BRAZIL | Comite de Etica em Pesquisa em Seres Humanos do Hospital Pro-Cardiaco Pronto Socorro Cardiologico  Rua Paulo Barretto, 86  Rio de Janeiro, RJ 22280-010  BRAZIL |
|  |  |  |  |  |  |

## Bulgaria

**Coordinating Investigators:**

<None Entered>

| **Center** | **Principal Investigator** | **Co-Investigator(s)** | **Sub-Investigator(s)** | **Address(es)** | **Institutional Review Board or Ethics Committee Address(es)** |
| --- | --- | --- | --- | --- | --- |
|  |  |  |  |  |  |
| 1130 | Prof. Zlatimir Kolarov |  | Dr. Penka Bekyarova  Dr. Daniela Dimitrova  Dr. Mariana Ivanova Goycheva  Silviya Marincheva  Dr. Lubomir Marinov Marintchev  Dr. Simeon Monov  Assoc. Prof. Veneta Paskaleva-Peytcheva  Dr. Tzvetanka Petranova  Prof. Iordan I. Sheytanov  Dr. Ivan Y. Sheytanov  Dr. Tsvetelina Dimitrova Yoneva | Clinic of Rheumatology Multiprofile Hospital for Active treatment Sveti Ivan Rilski  13, Urvich Str.  Sofia, 1612  BULGARIA | Ethics Committee for Multicenter Trials  Pl. Sveta Nedelya 5  Sofia, 1000  BULGARIA |
|  |  |  |  |  |  |
| 1132 | Dr. Daniela Yaneva Bichovska |  | Dr. Ivan Bichovski  Emilia F. Fileva-Veleva  Dr. Mario B. Markov | DKTs "Sveta Anna" Sofia,  Konsultativen kabinet po Revmatologia  ul. Dimitar Mollov 1  Diagnostic Consultative Center "Sveta Anna"  Sofia, 1709  BULGARIA | Ethics Committee at MHAT "Sveta Anna"  Ethics Committee  1 Dimitar Mollov Str.  Sofia, 1709  BULGARIA  Ethics Committee for Multicenter Trials  Pl. Sveta Nedelya 5  Sofia, 1000  BULGARIA |
|  |  |  |  |  |  |
| 1133 | Dr. Boycho Oparanov |  | Dr. Borislava Angelova Ilchova  Dr. Raycho Raychev  Dr. Ignat Zhutev | Clinic of Cardiology and Rheumatology, Military Medical Academy  3, Georgi Sofiiski Str.  Sofia 1606,  BULGARIA  Clinic of Cardiology and Rheumatology, Military Medical Academy 3  Georgi Sofiiski Str.  Sofia, 1606  BULGARIA  MBAL na Voennomeditsinska Akademia - Sofia, Klinika po Revmatologia i Kardiologia  MMA-HAT Sofia  Ul. Georgi Sofiyski 3  Sofia, 1606  BULGARIA | Ethics Committee  3, Georgi Sofiiski  Sofia, 1606  BULGARIA  Ethics Committee for Multicenter Trials  Pl. Sveta Nedelya 5  Sofia, 1000  BULGARIA |
|  |  |  |  |  |  |
| 1134 | Assoc. Prof. Snezhanka Tisheva (Previous PI)  Prof. Kiril Yablanski |  | Dr. Virzhiniya Yordanova | Revmatologichen kabinet, DKTs Sv. Pantaleimon OOD  Ul. Trite bora 24, Zh.k. Druzhba  Pleven, 5800  BULGARIA | Ethics Committee for Multicenter Trials  Pl. Sveta Nedelya 5  Sofia, 1000  BULGARIA  Etichna komisiya pri DKTs Sv. Pantaleimon OOD  DKTs Sv. Pantaleimon OOD  Ul. Trite bora 24, Zh.k. Druzhba  Pleven, 5800  BULGARIA |
|  |  |  |  |  |  |
| 1448 | Dr. Ivan Goranov |  | Dr. Rumen Dachev  Dr. Katya Kuchmova | Revmatologichno Otdelenie, MBAL - Plovdiv  bul. Bulgaria 234  Plovdiv, 4000  BULGARIA | Ethics Committee for Multicenter Trials  Pl. Sveta Nedelya 5  Sofia, 1000  BULGARIA  Komisiya po etika pri MBAL-Plovdiv/Ethics Committee at MHAT-Plovdiv  MBAL-Plovdiv  MHAT-Plovdiv  bul. ¿Bulgaria¿ 234  Plovdiv, 4000  BULGARIA |
|  |  |  |  |  |  |
| 1449 | Prof. Anastas Batalov |  | Dr. Rositsa Karalilova  Aleksander Marinkov  Dr. Aneta Nikolova  Dr. Dimitar Penev  Ginka Poneva | MBAL "Kaspela" Plovdiv, Otdelenie po revmatologia  MHAT "Kaspela" Plovdiv  ul. Sofiya  64  Plovdiv, 4002  BULGARIA | Ethic Committee for Multicenter Trials / Etichna  pl. "Sveta Nedeliya" 5  Sofia, 1000  BULGARIA |
|  |  |  |  |  |  |
| 1533 | Nikolay G. Nikolov |  | Dr. Todor Hinov  Hristina P. Kostadinova  Plamen Kulikov  Dr. Borislav Nikolov | DKTs Akta Medika EOOD  Konsultativen kabinet po revmatologia  ul. Nikola Petkov 60  Sevlievo, 5400  BULGARIA | Ethics Committee for Multicenter Trials  Pl. Sveta Nedelya 5  Sofia, 1000  BULGARIA  Komisiya po etika pri DKTs"Akta Medika"/ Ethics Committee at DCC "Akta Medika"  DKTs"Akta Medika" EOOD  Diagnostic Consultative Center "Akta Medika" EOOD  ul. "Nikola Petkov" 60  Sevlievo, 5400  BULGARIA |
|  |  |  |  |  |  |
| 1621 * | Dr. Nadezhda G. Yordanova  Dr. Nadezhda G. Yordanova (Previous PI)  Svetla Kopcheva (Previous PI) |  | Dr. Varbitsa Hergeldzhieva  Dr. Penka Kamenova  Dr. Emil Parashkevov | MBAL-Ruse, AD, IV Terapevtichno i kardiologichno otdelenie  MHAT Ruse  2 ul. Nezavisimost  Ruse, 7002  BULGARIA | Ethics Committee for Multicenter Trials  Pl. Sveta Nedelya 5  Sofia, 1000  BULGARIA  Komisiya po etika pri MBAL-Ruse, AD  MBAL-Ruse, AD  MHAT Ruse  2 ul. Nezavisimost  Ruse, 7002  BULGARIA |
|  |  |  |  |  |  |
| 1622 | Svetla Kopcheva |  | Dr. Vanya Gorcheva  Dr. Dimitar Kazakov  Daniela Purcheva | MOBAL "D-r Stefan Cherkezov" AD, Revmokardiologichno otdelenie s intenziven sektor MHAT  "Dr. Stefan Cherkezov" AD ul. Nish 1  Veliko Tarnovo, 5000  BULGARIA | Ethics Committee for Multicenter Trials  26, Yanko Sakazov  Sofia, 1504  BULGARIA |
|  |  |  |  |  |  |
| 1670 | Rumen M. Stoilov |  | Dr. Penka Bekyarova  Dr. Kameliya Ivanova Garbeva-Popova  Dr. Mariana Ivanova Goycheva  Reneta Kroitorova-Dimitrova  Dr. Natalia Marinova  Dr. Valentina Reshkova | MBAL Sveti Ivan Rilski Sofia; Klinika po Revmatologia  MHAT Sveti Ivan Rilski  13, Urvich St.  Sofia, 1612  BULGARIA | Ethics Committee for Multicenter Trials  Pl. Sveta Nedelya 5  Sofia, 1000  BULGARIA |
|  |  |  |  |  |  |

## Canada

**Coordinating Investigators:**

<None Entered>

| **Center** | **Principal Investigator** | **Co-Investigator(s)** | **Sub-Investigator(s)** | **Address(es)** | **Institutional Review Board or Ethics Committee Address(es)** |
| --- | --- | --- | --- | --- | --- |
|  |  |  |  |  |  |
| 1391 | Dr. Alfred Augusto Cividino |  | Dr. Raja Bobba  Dr. Pauline Boulos  Dr. Mark P. Matsos  Dr. Janet Miron  Dr. Louise Sloat | Cividino Medicine Professional Corporation  187 Hughson Street South  Hamilton, ON L8N 2B6  CANADA  MAC Research Inc.  187 Hughson Street South  Hamilton, ON L8N 2B6  CANADA | IRB Services  Suite 300  372 Hollandview Trail  Aurora, ON L4G 0A5  CANADA |
|  |  |  |  |  |  |
| 1392 | Dr. J. Carter Thorne |  | Dr. Edward Ng Tung Hing  Dr. Jennifer Reist  Dr. Nooshin Samadi | The Arthritis Program Research Group Inc.  43 Lundy's Lane  Newmarket, ON L3Y 3R7  CANADA | IRB Services  Suite 300  372 Hollandview Trail  Aurora, ON L4G 0A5  CANADA |
|  |  |  |  |  |  |
| 1394 | Dr. Majed Khraishi |  | Karen Doyle  Dr. Ian Landells  Tina Pretty-Haines | Nexus Clinical Research  Suite 102  120 Stavenger Drive  St. John's, NL A1A 5E8  CANADA | IRB Services  Suite 300  372 Hollandview Trail  Aurora, ON L4G 0A5  CANADA |
|  |  |  |  |  |  |
| 1395 | Dr. Boulos Haraoui |  | Dr. Denis Choquette  Raymonde Gregoire  Jolaine L'Archeveque  Dr. Jean-Pierre Raynauld | Institut de Rhumatologie de Montreal  1551 Ontario Est  Montreal, QC H2L 1S6  CANADA | IRB Services  Suite 300  372 Hollandview Trail  Aurora, ON L4G 0A5  CANADA |
|  |  |  |  |  |  |
| 1396 | Dr. Milton F. Baker |  | Dr. Christopher Atkins  Dr. Leeanna Bulinckx  Dr. Linda Ross | PerCuro Clinical Research Ltd.  200 - 1105 Pandora Avenue  Victoria, BC V8V 3P9  CANADA | IRB Services  Suite 300  372 Hollandview Trail  Aurora, ON L4G 0A5  CANADA |
|  |  |  |  |  |  |
| 1397 | Dr. Louis Bessette |  | Dr. Marie-Claire Banville  Dr. Jacques Brown  Chantal Jacques  Evelyne Lejeune  Dr. Louise Morin  Anne Perron | Groupe de Recherche en Rhumatologie et Maladies Osseuses  Suite 101  1200 Avenue de Germain-des-Pres  Sainte-Foy, QC G1V 3M7  CANADA | IRB Services  Suite 300  372 Hollandview Trail  Aurora, ON L4G 0A5  CANADA |
|  |  |  |  |  |  |
| 1398 | Dr. Henry Niall Jones |  | Dr. Dalton Ernest Sholter  Dr. Alexander Yan | Rheumatology Research Associates Ltd.  10839 124 Street  Edmonton, AB T5M 0H4  CANADA | College of Physicians and Surgeons of Alberta - Research Ethics Review Committee  2700  10020 - 100 Street NW  Edmonton, AB T5J 0N3  CANADA |
|  |  |  |  |  |  |
| 1399 | Dr. Andre Damien Beaulieu |  | Dr. Pierre Lachance  Ms. Johanne Rioux  Dr. Rene Therrien | Centre de Traitement Specialise en Rhumatologie (C.T.S.R.)  Suite 360  3165, chemin St-Louis  Quebec, QC G1W 4R4  CANADA | IRB Services  Suite 300  372 Hollandview Trail  Aurora, ON L4G 0A5  CANADA |
|  |  |  |  |  |  |
| 1401 | Dr. Andrew Chow |  | Dr. Ami Mody  Dr. Elaine Soucy | Credit Valley Rheumatology  Suite 209  2300 Eglinton Avenue West  Mississauga, ON L5M 2V8  CANADA | IRB Services  Suite 300  372 Hollandview Trail  Aurora, ON L4G 0A5  CANADA |
|  |  |  |  |  |  |
| 1402 | Dr. Alice V. Klinkhoff |  | Dr. Kenneth Blocka  Dr. Andrew Chalmers | Dr. Alice Klinkhoff Inc.  895 West 10th Avenue,2ND FLOOR  Vancouver, BC V5Z 1L7  CANADA  The Arthritis Research Centre of Canada  895 West 10th Avenue  Vancouver, BC V5Z 1L7  CANADA | IRB Services  Suite 300  372 Hollandview Trail  Aurora, ON L4G 0A5  CANADA |
|  |  |  |  |  |  |
| 1403 | Dr. Bindu Nair |  | Dr. Regina Taylor-Gjevre | Royal University Hospital  Division of Rhuematology  103 Hospital Drive  55 Building  Saskatoon, SK S7H-2Z1  CANADA | University of Saskatchewan BioMedical Research Ethics Board (Bio-REB)  Research Ethics Office, University of Saskatchewan  NRC - Plant Biotechnology Research Institute  1607 - 110 Gymnasium Place  Saskatoon, SK S7N 4J8  CANADA |
|  |  |  |  |  |  |
| 1404 | Dr. Janet E. Pope |  | Lindsey Carroll  Dr. Sherry Rohekar  Dr. Gina Rohekar | St Joseph's Health Centre  268 Grosvenor Street  Monsignor Roney Building Level D2  PO Box 5777 Stn B  London, ON N6A 4V2  CANADA | Office of Research Ethics, University of Western Ontario  Room 4180  Support Services Building  1393 Western Road  London, ON N6A 5C1  CANADA  University of Western Ontario Health Sciences Research Ethics Board  Room 5150  Support Services Building  London, ON N6A 3K7  CANADA |
|  |  |  |  |  |  |
| 1406 | Dr. Jean-Luc Tremblay |  | Dr. Michele Dessureault  Dr. Julie Drouin  Dr. Clode Lessard  Frederic Morin  Louise Rouleau  Ms. Carmen Trudel | Centre de Recherche Musculo-Squelettique  1119 Ste Marguerite  Trois-Rivieres, QC G8Z 1Y2  CANADA | IRB Services  Suite 300  372 Hollandview Trail  Aurora, ON L4G 0A5  CANADA |
|  |  |  |  |  |  |
| 1542 | Dr. Timothy G. McCarthy |  | Dr. Cory Baillie  Ms. Shelley Shepherd  Janna Tweed | Manitoba Clinic  790 Sherbrook Street  Winnipeg, MB R3A 1M3  CANADA | IRB Services  Suite 300  372 Hollandview Trail  Aurora, ON L4G 0A5  CANADA |
|  |  |  |  |  |  |
| 1543 | Dr. Robert J. McKendry |  | Dr. Gunnar R. Kraag  Ms. Paula Patterson | Rheumatology Research Associates  Suite 412  1919 Riverside Drive  Ottawa, ON K1H 1A2  CANADA | IRB Services  Suite 300  372 Hollandview Trail  Aurora, ON L4G 0A5  CANADA |
|  |  |  |  |  |  |
| 1544 | Dr. William G. Bensen |  | Dr. Fernando Anthony Bianchi  Dr. Melissa Deamude | Dr. William G. Bensen Medicine Professional Corporation  Suite 203  25 Charlton Avenue East  Hamilton, ON L8N 1Y2  CANADA  Office of Dr. Fernando Bianchi  Suite 304  25 Charlton Avenue East  Hamilton, ON L8N 1Y2  CANADA | IRB Services  Suite 300  372 Hollandview Trail  Aurora, ON L4G 0A5  CANADA |
|  |  |  |  |  |  |
| 1545 | Dr. Mary Ann Fitzcharles |  | Dr. Martin A. Cohen  Dr. Michael R. Starr | West Island Rheumatology Research Associates  Suite 209  269 St. Jean Boulevard  Pointe-Claire, QC H9R 3J1  CANADA | IRB Services  Suite 300  372 Hollandview Trail  Aurora, ON L4G 0A5  CANADA |
|  |  |  |  |  |  |
| 1584 | Dr. Rafat Faraawi |  | Dr. Kelly Roth  Marzenna Zygmunt | KW Musculoskeletal Research Inc.  Suite 201  564 Belmont Avenue West  Kitchener, ON N2M 5N6  CANADA | IRB Services  Suite 300  372 Hollandview Trail  Aurora, ON L4G 0A5  CANADA |
|  |  |  |  |  |  |
| 1585 | Dr. Jude F. Rodrigues |  | Ms. Mary Ann Rodrigues | Clinical Research and Arthritis Centre  Suite 160  1720 Howard Avenue  Windsor, ON N8X 5A6  CANADA | IRB Services  Suite 300  372 Hollandview Trail  Aurora, ON L4G 0A5  CANADA |
|  |  |  |  |  |  |
| 1586 | Dr. Anna Maria Jaroszynska |  | Dr. Grzegorz Jaroszynski | Burlington Rheumatology and Osteoporosis Clinic  Suite 23  1960 Appleby Line  Burlington, ON L7L 0B7  CANADA | IRB Services  Suite 300  372 Hollandview Trail  Aurora, ON L4G 0A5  CANADA |
|  |  |  |  |  |  |
| 1587 | Dr. Saeed Raza Shaikh |  | Marcie Bak | Niagara Peninsula Arthritis Centre Inc.  Suite 5  2 Lakeshore Road  St. Catharines, ON L2N 7E4  CANADA | IRB Services  Suite 300  372 Hollandview Trail  Aurora, ON L4G 0A5  CANADA |
|  |  |  |  |  |  |

## Chile

**Coordinating Investigators:**

<None Entered>

| **Center** | **Principal Investigator** | **Co-Investigator(s)** | **Sub-Investigator(s)** | **Address(es)** | **Institutional Review Board or Ethics Committee Address(es)** |
| --- | --- | --- | --- | --- | --- |
|  |  |  |  |  |  |
| 1066 | Dr. Pedro Claudio Miranda-Cabezas |  | Dr. Jorge Saavedra Munoz  Dr. Cecilia Paz Trejo Rojas | Centro de Estudios Reumatologicos  Avenida Salvador 960  Providencia  Santiago, RM 7501126  CHILE  Office of Dr. Pedro Miranda  Oficina 25  Avda. Bernardo O'Higgins 240  Santiago,  CHILE | Comite Etico Cientifico  Servicio de Salud Metropolitano Oriente  Av. Salvador 364  Santiago,  CHILE |
|  |  |  |  |  |  |
| 1090 | Dr. Renato Jimenez Calabresse |  | Dr. Cecilia Georgi  Dr. Ricardo Jerez  Dr. Luis Roca | Estudios Clinicos V Region  Alvarez 1806  Vina del Mar, V Region 2570017  CHILE | Comite Etico Cientifico del Servicio de Salud Vina-Quillota  Calle Limache 1307  Esquina Penablanca, Piso 2  Vina del Mar, V Region  CHILE |
|  |  |  |  |  |  |
| 1145 | Dr. Marta Ofelia Aliste Silva |  | Dr. Maria Francisca Sabugo  Dr. Lilian Soto  Dr. Pamela Wurmann Kiblinsky | Consulta Privada Dra. Marta Aliste  Guardia Vieja 255, Oficina 1409  Providencia  Santiago, RM 7510186  CHILE  Consulta Privada Dra. Marta Aliste  Guardia Vieja 255, Oficina 1409  Providencia  Santiago, RM 7510186  CHILE | Comite Etico Cientifico  Servicio de Salud Metropolitano Oriente  Av. Salvador 364  Santiago,  CHILE |
|  |  |  |  |  |  |
| 1173 | Dr. Francisco Ballesteros |  | Dr. Maria Eugenia Alvarez  Dr. Carlos Fuentealba  Carola Martínez  Dr. Paula Pozo | Centro de Diagnostico y Tratamiento San Borja Arriaran  Seccion Reumatologia  Amazonas 619  Santiago, RM 8360156  CHILE | Comite Etico Cientifico  Servicio de Salud Metropolitano Central  Santa Rosa 1234, Pabellon Errazuriz piso 2  Santiago, RM  CHILE |
|  |  |  |  |  |  |
| 1174 | Dr. Gloria Holuigue |  | Juan Carlos Carvajal  Christian Zenteno | Clínica Santa Maria  Seccion Reumatologia  Fernando Manterola 0540  Providencia, Santiago, RM 7530206  CHILE | Comite de Etica  Clinica Santa Maria  Avenida Santa Maria 0410  Santiago,  CHILE  Comite Etico Cientifico, Servicio de Salud Metropolitano Oriente  Avenida Salvador 364  Providencia  Santiago, RM 7500922  CHILE |
|  |  |  |  |  |  |
| 1527 | Dr. Ana Maria Flores |  | Dr. Diego Carvajal  Dr. Miguel A. Valenzuela | Hospital Regional de Rancagua  Alameda #611  VI Region  Rancagua, 2841959  CHILE | Comite Etico Cientifico  Servicio de Salud Metropolitano Oriente  Avenida Salvador 364  Providencia, Santiago RM 7500922  CHILE |
|  |  |  |  |  |  |
| 1591 | Dr. Lucia Ponce |  | Dr. Alfredo Danin  Dr. Victoria Espinosa  Dr. Carlos Martinez  Dr. Pablo Riedemann | Consulta Privada Dra. Lucia Ponce  Diego Portales 516  Temuco, IX Region 4790928  CHILE | Comite de Evaluacion Etica Cientifica  Servicio de Salud Araucania Sur  Andres Bello 636  Temuco, IX Region 4791301  CHILE |
|  |  |  |  |  |  |
| 1671 * | Dr. Veronica Aguirre |  | Maria Aburto  Jose Ponce de Leon | Hospital Base Valdivia, Servicio de Neurología  Avenida Simpson 850  Valdivia, Region XIV 5090145  CHILE | Comite Etica de Investigacion Servicio de Salud  Vicente Perez Rosalez 560  Oficina 307  Valdivia, Region XIV 5110537  CHILE |
|  |  |  |  |  |  |

## China

**Coordinating Investigators:**

<None Entered>

| **Center** | **Principal Investigator** | **Co-Investigator(s)** | **Sub-Investigator(s)** | **Address(es)** | **Institutional Review Board or Ethics Committee Address(es)** |
| --- | --- | --- | --- | --- | --- |
|  |  |  |  |  |  |
| 1316 | Dr. ZhanGuo LI |  | Yuan An  Tian Liu  Sisi Pan | Peking University People's Hospital/Rheumatology and Immunology Department  No.11, Xizhimen South Street  Xicheng District  Beijing, 100044  CHINA | Ethics Committee of Peking University People's Hospital  No.11, Xizhimen South Street  Xicheng District  Beijing, 100044  CHINA |
|  |  |  |  |  |  |
| 1317 | Dr. Feng Huang |  | Yan Li  Hua Ma  Hong ZHANG | PLA General Hospital  28 Fu Xing Road HaiDian Qu  Beijing, 100853  CHINA | Ethics Committee of Peking University People's Hospital  No.11, Xizhimen South Street  Xicheng District  Beijing, 100044  CHINA |
|  |  |  |  |  |  |
| 1319 | Yi ZHENG |  | Xin DONG  Haiyun Li  Xuhua Shi  Xiaohong Wen  Yongfeng Zhang | Beijing Chaoyang Hospital, Capital University of Medical Science  No.8 Baijiazhuang Road  Chaoyang District  Beijing, 100020  CHINA | Ethics Committee of Peking University People's Hospital  No.11, Xizhimen South Street  Xicheng District  Beijing, 100044  CHINA |
|  |  |  |  |  |  |
| 1320 | Dr. Chunde Bao |  | Qing Dai  Dr. Hui Du  Dr. Qiang Guo | Rheumatology and Immunology Dept., Renji Hospital Shanghai Jiao Tong University School of Medicine  NO. 145 Middle Shandong Road  Shanghai, 200001  CHINA | Ethics Committee of Renji Hospital Shanghai Jiao Tong University School of Medicine  Ethics Committee  No. 1630 Dongfang Road  Shanghai, 200127  CHINA |
|  |  |  |  |  |  |
| 1321 | Dongbao Zhao |  | Ji Lianmei  Dr. Yeqing Shi  Deshao YE  Ju Zhang | Shanghai Changhai Hospital  No. 168, Changhai Road  Shanghai, Shanghai 200433  CHINA | Ethics Committee of Peking University People's Hospital  No.11, Xizhimen South Street  Xicheng District  Beijing, 100044  CHINA |
|  |  |  |  |  |  |
| 1322 | Prof. Dr. Huji Xu |  | Dr. Ting Li  Li Lin  Jing ZHAO  Ling Zhou | Shanghai Changzheng Hospital  No 415 Fengyang Road  Shanghai, 200003  CHINA | Ethics Committee of Peking University People's Hospital  No.11, Xizhimen South Street  Xicheng District  Beijing, 100044  CHINA |
|  |  |  |  |  |  |
| 1323 | Prof. Dr. JieRuo GU |  | Jianlin Huang  Ou Jin  Zetao Liao  Yunfeng Pan  Yuqiong Wu | The Third Affiliated Hospital of Sun Yat-Sen University  No.600, Tianhe Road  Shipaigangding Tianhe District  Guangzhou, 510630  CHINA | Ec Of The Third Affiliated Hospital Of Sun Yat-Sen University No600 Tianhe Road Guangdong  Guangzhou, 510630  CHINA |
|  |  |  |  |  |  |
| 1324 | Yi Tao |  | Chenghui Huang  Zeying Lin  Ziguan Ye  Yingyu Zeng | The Second Affiliated Hospital of Guangzhou Medical College  No. 250, Changgang East Road, Haizhu District  Guangzhou, Guangdong 510260  CHINA | Ethics Committee of Peking University People's Hospital  No.11, Xizhimen South Street  Xicheng District  Beijing, 100044  CHINA |
|  |  |  |  |  |  |
| 1326 | Xingfu Li |  | Huaxiang Liu  Xiuhua WANG | QiLu Hospital of Shandong University  107 Western Culture Road  Jinan, Shandong 250012  CHINA | Ethic Committee of QiLu Hospital of Shandong University  107 Western Culture Road  Jinan, Shandong 250012  CHINA |
|  |  |  |  |  |  |
| 1327 | Houheng Su |  | Guangwen LI  Hong MA  Qian Xing | Qingdao Municipal Hospital  No. 1, Jiaozhou Road  Qingdao, Shandong 266011  CHINA | Ethics Committee of Peking University People's Hospital  No.11, Xizhimen South Street  Xicheng District  Beijing, 100044  CHINA |
|  |  |  |  |  |  |
| 1328 | Professor Ping Zhu |  | Lina Chen  Nan Leng  Lu Wang  Ronghua Xie | Xijing Hospital, The Fourth Military Medical University  No. 15 Changlexi Road  Xi'an, Shanxi 710032  CHINA | Ethics Committee of Peking University People's Hospital  No.11, Xizhimen South Street  Xicheng District  Beijing, 100044  CHINA |
|  |  |  |  |  |  |
| 1329 | Dr. JianHua XU |  | Jing Cai  Shanyu Chen  Li Lian  Shuang Liu  Hui Xiao | An Hui Medical University 1st Hospital  Rheumatology Department  No. 218, Jixi Road  Hefei, 230022  CHINA | Ethics Committee of Peking University People's Hospital  No.11, Xizhimen South Street  Xicheng District  Beijing, 100044  CHINA |
|  |  |  |  |  |  |
| 1330 | Dr. XiangPei LI |  | Xiaomei Li  Yan Ma  Min Zhang | Anhui Province Hospital  No.17 Lujiang Road  Hefei, 230001  CHINA | Ethics Committee of Peking University People's Hospital  No.11, Xizhimen South Street  Xicheng District  Beijing, 100044  CHINA |
|  |  |  |  |  |  |
| 1331 | Huaxiang Wu |  | Wenjia Sun | The Second Affiliated Hospital of Zhejiang University School of Medicine  no. 88, jiefang road,  Hangzhou, Zhejiang 310009  CHINA | Ethics Committee of Peking University People's Hospital  No.11, Xizhimen South Street  Xicheng District  Beijing, 100044  CHINA |
|  |  |  |  |  |  |
| 1332 | Miaojia Zhang |  | Yu Ding  Yanyan Wang  Guiqin Yuan | Jiangsu Province Hospital  No.300 Guangzhou Rd  Gu Lou District  Nanjing, 210029  CHINA | Ethics Committee of Peking University People's Hospital  No.11, Xizhimen South Street  Xicheng District  Beijing, 100044  CHINA |
|  |  |  |  |  |  |
| 1333 | Zhiwei Chen |  | Yingsu Deng  Jian Wu  Keqin Zeng | The First Affiliated Hospital of Soochow University  Department of Rheumatology  No188 Shizi street  Suzhou, 215006  CHINA | EC of the First Affiliated Hospital of Soochow University  Department of Rheumatology,No.188 Shizi Street,  Suzhou, Jiangsu 215006  CHINA |
|  |  |  |  |  |  |
| 1334 | Shaoxian Hu |  | Dr. PeiGen He  Xiaomei Lei  Wei Tu  Fei Yu | Tongji Hospital, Tongji Medical College, Huazhong University of Science and Technology  No.1095, Jiefang Road  Wuhan, Hubei 430030  CHINA | Ethics Committee of Peking University People's Hospital  No.11, Xizhimen South Street  Xicheng District  Beijing, 100044  CHINA |
|  |  |  |  |  |  |
| 1335 | Professor Xiaoxia Zuo |  | Yisha Li  Sijia Liu  Hui Luo  Yanli Xie  Hongjun Zhao | Xiangya Hospital of Centre-south University  No. 87 Xiangya Road  Changsha, Hunan 410008  CHINA | Ethics Committee of Peking University People's Hospital  No.11, Xizhimen South Street  Xicheng District  Beijing, 100044  CHINA |
|  |  |  |  |  |  |
| 1336 | Dr. Yi Liu |  | Hui Lin  Chunyu Tan  Honghu Tang | Si Chuan Huaxi Hospital  Rheumatology Department  No.37, Wainanguoxuexiang  Chengdu, 610041  CHINA | Ethics Committee of Peking University People's Hospital  No.11, Xizhimen South Street  Xicheng District  Beijing, 100044  CHINA |
|  |  |  |  |  |  |
| 1337 | Dr. Lu GONG |  | Yongyu Liu  Wenwen Sun  Wei Wei  Na Zhang | Department of Infectious Diseases & Immunology, Tianjin Medical University General Hospital  No. 154, Anshan Road, Heping District  Tianjin, 300052  CHINA | Ethics Committee of Peking University People's Hospital  No.11, Xizhimen South Street  Xicheng District  Beijing, 100044  CHINA |
|  |  |  |  |  |  |

## Colombia

**Coordinating Investigators:**

<None Entered>

| **Center** | **Principal Investigator** | **Co-Investigator(s)** | **Sub-Investigator(s)** | **Address(es)** | **Institutional Review Board or Ethics Committee Address(es)** |
| --- | --- | --- | --- | --- | --- |
|  |  |  |  |  |  |
| 1350 | Dr. Edwin Antonio Jauregui (Previous PI)  Dr. Maria Concepcion Maldonado-Lopez |  | Dr. Maria Claudia Diaz  Dr. Aura Maria Dominguez  Andres Alfonso Gonzalez Romero  Dr. Edwin Antonio Jauregui  Dr. Jhon Jairo Medina  Dr. Yenny Soraida Valero | Riesgo De Fractura S.A  Carrera 12 No. 98-38  Bogota, Cundinamarca 0000  COLOMBIA | Comite de Etica de la Investigacion Riesgo de Fractura S.A.  Comite de Etica de la investigacion Riesgo de Fractura S.A  Carerra 13 N° 97-25  Bogota D.C., Cundinamarca 0000  COLOMBIA |
|  |  |  |  |  |  |
| 1355 | Dr. William Jose Otero Escalante |  | Dr. Carlos Andres Calderon Cordero  Dr. Marcial Martinez  Dr. Genaro Alexis Paredes Portillo  Silvia Constanza Plata Vanegas  Dr. Gerardo Ramirez MD  Dr. Fabian Alberto Ramirez Rubio  Dr. Elsa Reyes Sanmiguel  Dr. Hernan Roberto Vera Quinche | SERVIMED E.U.  Calle 51 No. 34-17 Consultorio 205-208  Centro Comercial Cabecera I Etapa  Bucaramanga, Santander 0000  COLOMBIA | Comite de ética en Investigación de Servimed E.U  Calle 51 No. 34-17 Consultorio 205-208  Centro Comercial cabecera I Etapa  Bucaramanga, Santander 0000  COLOMBIA |
|  |  |  |  |  |  |
| 1521 | Dr. Juan Jose Jaller Raad |  | Dr. Javier Cuartas  Dr. Ariel Herrera Perez  Dr. Anubys Uriel Maiguel Noriega  Javier Enrique Rueda Hernandez  Dr. Victor Andres Ulloque Lopez | Centro de Reumatologia y Ortopedia  Carrera 49C No. 82-120  Barranquilla, Atlantico 0000  COLOMBIA | Comité de etica independiente centro de reumatologia y ortopedia  Cr. 49C No. 82-120  Barranquilla, Atlantico 0000  COLOMBIA |
|  |  |  |  |  |  |
| 1583 | Dr. Patricia Julieta Velez-Sanchez |  | Dr. Luisa Fernanda Amador Rodriguez  Dr. Ana Milena Arbelaez Solera  Dr. Carolina Maria Munoz  Dr. Andres F. Prasca Vengoechea  Dr. Juan Carlos Salazar  Dr. Edgardo David Tobias | Centro Integral de Reumatologia e Inmunologia S.A.S. CIREI S.A.S  CIREI Carrera 12 No 97-44  Cundinamarca  Bogota, 0000  COLOMBIA | Comite de etica de la investigación-Riesgo de Fractura S.A  Carrera 13 No. 97-25  Bogota, Cundinamarca 0000  COLOMBIA |
|  |  |  |  |  |  |

## Costa Rica

**Coordinating Investigators:**

<None Entered>

| **Center** | **Principal Investigator** | **Co-Investigator(s)** | **Sub-Investigator(s)** | **Address(es)** | **Institutional Review Board or Ethics Committee Address(es)** |
| --- | --- | --- | --- | --- | --- |
|  |  |  |  |  |  |
| 1435 | Dr. Raul Alpizar |  | Dr. Victor Cisneros-Perez  Dr. Allan Soto-Calvo | Centro de Reumatología y Osteoporosis, Cartago  de la esquina noroeste del Convento de los Padres Capuchinos  75 metros al este  Cartago, 00000  COSTA RICA | UCIMED  Comite Etico Cientifico de la Universidad de Ciencias Medicas  400 mts Oeste del Ministerio de Agricultura y Ganaderia  Sabana Oeste  San Jose,  COSTA RICA |
|  |  |  |  |  |  |
| 1437 | Dr. Daniel Alfaro-Vargas |  | Raul Sotomayor MD | Hospital Cima San Jose  De Multiplaza Escazu, 600 mts este, carretera Propero Fernandez, continuag a PriceMart  Smart. Escazu  San Jose,  COSTA RICA  Laboratorio CIM (Centro de Investigaciones Medicas)  Frente a la entrada de emergencias del Hospital Nacional de Ninos  San Jose,  COSTA RICA | UCIMED  Comite Etico Cientifico de la Universidad de Ciencias Medicas  400 mts Oeste del Ministerio de Agricultura y Ganaderia  Sabana Oeste  San Jose,  COSTA RICA |
|  |  |  |  |  |  |
| 1582 | Dr. Jose F. Diaz-Coto |  | Dina Arrieta  Dr. Pablo Monge-Zeledon  Dr. Alvaro Montero-Vega | Oficina Privada Del Colegio deMedicos  Frente al Colegio la Salle  50 mts este y 400 mts sur  San Jose,  COSTA RICA | INSTITUTO COSTARRICENSE DE INVESTIGACIONES CLÍNICAS  Urbanizacion Los Arboles, Frente al CENARE  La Uruca, San Jose  COSTA RICA |
|  |  |  |  |  |  |

## Croatia

**Coordinating Investigators:**

Dr. Marko Baresic

Doc.dr. Jasminka Milas Ahic

Dr. Mislav Radic

| **Center** | **Principal Investigator** | **Co-Investigator(s)** | **Sub-Investigator(s)** | **Address(es)** | **Institutional Review Board or Ethics Committee Address(es)** |
| --- | --- | --- | --- | --- | --- |
|  |  |  |  |  |  |
| 1147 | Prof.dr Bozidar Curkovic |  | Dr. Kristina Kovac Durmis  Dr. Iva Popovic  Dr Iva Zagar | University Hospital Center "Zagreb"  Kispaticeva 12  Zagreb, 10000  CROATIA | Central Ethics Committee  Ksaver 200a  Zagreb, 10000  CROATIA |
|  |  |  |  |  |  |
| 1187 | Assoc. Prof. Dusanka Martinovic Kaliterna |  | Dr. Mislav Radic | University Hospital Split, Department for Internal Medicine, Division of Clinical Rheumatology  Spinciceva1  Split, 21000  CROATIA | CENTRAL ETHICS COMMITTEE  KSAVER  200A  ZAGREB, 10000  CROATIA |
|  |  |  |  |  |  |
| 1458 | Dr. Ksenija Mastrovic Radoncic |  | Dr. Maja P. Paar Puhovski | Institute for physical medicine, rehabilitation and rheumatology, General Hospital "Sveti duh"  Sveti Duh 64  Zagreb, 10000  CROATIA | CENTRAL ETHICS COMMITTEE  KSAVER  200A  ZAGREB, 10000  CROATIA |
|  |  |  |  |  |  |
| 1461 | Prof.dr Nada Cikes |  | Dr. Branimir Anic  Dr. Marko Baresic  Dr. Mislav Cerovec  Dr. Mirna Sentic | University Hospital Center Zagreb  Kispaticeva 12  Zagreb, 10000  CROATIA | Central Ethics Committee,Agency for Medicinal Products and Medical Devices  Ksaverska cesta 4  Zagreb, 10000  CROATIA |
|  |  |  |  |  |  |
| 1463 | Dr. Visnja Prus |  | Dr. Drazen Bedekovic  Doc.dr. Jasminka Milas Ahic | University Hospital Center Osijek  Deaprtment of Internal Medicine  JHuttlera 4  Osijek, 31000  CROATIA | Central Ethics Committee  Ksaver 200A  Zagreb, 10000  CROATIA  Central Ethics Committee,Agency for Medicinal Products and Medical Devices  Ksaverska cesta 4  Zagreb, 10000  CROATIA |
|  |  |  |  |  |  |

## Czech Republic

**Coordinating Investigators:**

<None Entered>

| **Center** | **Principal Investigator** | **Co-Investigator(s)** | **Sub-Investigator(s)** | **Address(es)** | **Institutional Review Board or Ethics Committee Address(es)** |
| --- | --- | --- | --- | --- | --- |
|  |  |  |  |  |  |
| 1122 | Dr. Sarka Forejtova |  | Dr. Katerina Jarosova  Dr. Olga Ruzickova  Dr. Dana Tegzova  Prof. Jiri Vencovsky | Institute of Rheumatology  Na Slupi 4  Praha 2, 128 50  CZECH REPUBLIC  Revmatologicky ustav  Radiodiagnosticke oddeleni  Na Slupi 4  Praha 2, 12850  CZECH REPUBLIC | Eticka komise  Revmatologicky ustav  Na Slupi 4  Praha 2, 128 50  CZECH REPUBLIC  Eticka komise IKEM a FTNsP  Videnska 800  Praha 4 Krc, 140 59  CZECH REPUBLIC |
|  |  |  |  |  |  |
| 1123 | Dr. Jan Rosa |  | Dr. Petr Kasalicky  Dr. Simona Skacelova | DC Mediscan  Sustova 1930  Praha 11 - Chodov, 148 00  CZECH REPUBLIC  Nemocnice na Frantisku  Radiodiagnosticke oddeleni  Na Frantisku 847/8  Praha 1, 11000  CZECH REPUBLIC | Eticka komise IKEM a FTNsP  Videnska 800  Praha 4 Krc, 140 59  CZECH REPUBLIC |
|  |  |  |  |  |  |
| 1124 | Dr. Sevda Augustinova |  | Dr. Jan Augustin  Dr. Vera Vlasakova | MEDIPONT Plus, s.r.o.  Matice Skolske 17  Ceske Budejovice, 370 01  CZECH REPUBLIC | Eticka komise IKEM a FTNsP  Videnska 800  Praha 4 Krc, 140 59  CZECH REPUBLIC |
|  |  |  |  |  |  |
| 1125 | Dr. Petr Vitek |  |  | Nemocnice Atlas, a.s.  Radiodiagnosticke oddeleni  tr. T. Bati 5135  Zlin, 760 01  CZECH REPUBLIC  PV-Medical s.r.o.  Revmatologicka ambulance  Stefanikova 477  Zlin, 760 01  CZECH REPUBLIC | Eticka komise IKEM a FTNsP  Videnska 800  Praha 4 Krc, 140 59  CZECH REPUBLIC |
|  |  |  |  |  |  |
| 1126 | Dr. Petr Kopsa |  | Dr. Marie Sedlackova  Dr. Lenka Zouharova | Fakultni Thomayerova nemocnice s poliklinikou  Revmatologicke a rehabilitacni oddeleni  Videnska 800  Praha 4, 140 59  CZECH REPUBLIC | Eticka komise IKEM a FTNsP  Videnska 800  Praha 4 Krc, 140 59  CZECH REPUBLIC |
|  |  |  |  |  |  |
| 1127 | Dr. Leona Prochazkova |  | Dr. Jana Bohmova  Dr. Petr Nemec  Dr. Vlastimil Racek | Fakultni nemocnice u sv. Anny v Brne, II. Interni klinika  Pekarska 53  Brno, 656 91  CZECH REPUBLIC | Eticka komise Fakultni nemocnice u sv. Anny v Brne  Pekarska 53  Brno, 656 91  CZECH REPUBLIC  Eticka komise IKEM a FTNsP  Videnska 800  Praha 4 Krc, 140 59  CZECH REPUBLIC |
|  |  |  |  |  |  |
| 1274 | Dr. Zdenek Dvorak |  | Dr. Martina Vaneckova | ARTHROMED, s. r. o. Revmatologicka ambulance  Rokycanova 2798  Pardubice, 530 02  CZECH REPUBLIC  CCBR Czech, a.s.  Trida miru 2800  Pardubice, 530 02  CZECH REPUBLIC | Eticka komise IKEM a FTNsP  Videnska 800  Praha 4 Krc, 140 59  CZECH REPUBLIC |
|  |  |  |  |  |  |
| 1275 | Dr. Libor Novosad |  |  | L.K.N. Arthrocentrum, s.r.o.  Revmatologicka ambulance  Na Valech 1  Hlucin, 748 01  CZECH REPUBLIC | Eticka komise IKEM a FTNsP  Videnska 800  Praha 4 Krc, 140 59  CZECH REPUBLIC |
|  |  |  |  |  |  |
| 1276 | Dr. Zuzana Stejfova |  |  | Nuselska poliklinika  LEKARNA  Taborska 57  Praha 4, 14000  CZECH REPUBLIC  Revmatologicka ambulance  Nuselska poliklinika  Taborska 57  Praha 4, 140 00  CZECH REPUBLIC | Eticka komise IKEM a FTNsP  Videnska 800  Praha 4 Krc, 140 59  CZECH REPUBLIC |
|  |  |  |  |  |  |
| 1277 | Dr. Zuzana Urbanova |  |  | Revmatologicka ambulance  Petra Rezka 1090/3  Praha 4, 14000  CZECH REPUBLIC | Eticka komise IKEM a FTNsP  Videnska 800  Praha 4 Krc, 140 59  CZECH REPUBLIC |
|  |  |  |  |  |  |
| 1278 | Dr. Jana Kopackova |  |  | ARTMEDI UPD s r.o.  Ceskoslovenske armady 164  Hostivice, 253 01  CZECH REPUBLIC  Nemocnice na Frantisku  Radiodiagnosticke oddeleni  Palackeho 720/5  Praha 1, 11000  CZECH REPUBLIC | Eticka komise IKEM a FTNsP  Videnska 800  Praha 4 Krc, 140 59  CZECH REPUBLIC |
|  |  |  |  |  |  |
| 1279 | Dr. Zdenka Mosterova |  | Dr. Erik Moster | Revmacentrum MUDr. Mostera, s.r.o.  Mosnova 8  Brno - Zidenice, 615 00  CZECH REPUBLIC  X-MEDICA s.r.o.  Jugoslavska 11  Brno, 61300  CZECH REPUBLIC | Eticka komise IKEM a FTNsP  Videnska 800  Praha 4 Krc, 140 59  CZECH REPUBLIC |
|  |  |  |  |  |  |
| 1314 | Dr. Zdenek Fojtik |  | Dr. Libor Cervinek  Dr. Monika Obrovska | FN Brno, Interni hematoonkologicka klinika  Revmatologicka ambulance  Jihlavska 20  Brno, 625 00  CZECH REPUBLIC | Eticka komise FN Brno  Jihlavska 20  Brno, 625 00  CZECH REPUBLIC  Eticka komise IKEM a FTNsP  Videnska 800  Praha 4 Krc, 140 59  CZECH REPUBLIC |
|  |  |  |  |  |  |
| 1315 | Dr. Helena Stehlikova |  |  | Ordinace revmatologa  Pod Holym vrchem 349  Ceska Lipa, 470 01  CZECH REPUBLIC | Eticka komise IKEM a FTNsP  Videnska 800  Praha 4 Krc, 140 59  CZECH REPUBLIC |
|  |  |  |  |  |  |
| 1603 | Dr. Petr Bradna |  | Dr. Michal Kodeda  Dr. Tomas Soukup  Dr. Jan Toms | Fakultni nemocnice Hradec Kralove  2. Interni klinika - revmatologie  Sokolska 581  Hradec Kralove, 50005  CZECH REPUBLIC  Fakultni nemocnice Hradec Kralove  Radiologicka klinika  Sokolska 581  Hradec Kralove, 50005  CZECH REPUBLIC | Eticka komise Fakultni nemocnice Hradec Kralove  Sokolska 581  Hradec Kralove, 500 05  CZECH REPUBLIC  Eticka komise IKEM a FTNsP  Videnska 800  Praha 4 Krc, 140 59  CZECH REPUBLIC |
|  |  |  |  |  |  |
| 1605 | Dr. Miroslava Rosypalova |  |  | Mephacentrum  Rentgenologicke pracoviste  Opavska 962  Ostrava - Poruba, 70800  CZECH REPUBLIC  Revmatologicka ambulance  Panelova 6116  Ostrava - Poruba, 70800  CZECH REPUBLIC | Eticka komise IKEM a FTNsP  Videnska 800  Praha 4 Krc, 140 59  CZECH REPUBLIC |
|  |  |  |  |  |  |

## Denmark

**Coordinating Investigators:**

<None Entered>

| **Center** | **Principal Investigator** | **Co-Investigator(s)** | **Sub-Investigator(s)** | **Address(es)** | **Institutional Review Board or Ethics Committee Address(es)** |
| --- | --- | --- | --- | --- | --- |
|  |  |  |  |  |  |
| 1347 | Dr. Bente Danneskiold-Samsoe |  | Dr. Henning Bliddal  Dr. Henrik Gudbergsen  Dr. Anja Falk Riecke  Dr. Birgit Falk Riecke | Frederiksberg Hospital  Parker Instituttet  Nordre Fasanvej 57  Frederiksberg, 2000  DENMARK | De Videnskabsetiske Komitéer for Region Hovedstaden  Regionsgaarden  Kongens Vaenge 2  Hilleroed, 3400  DENMARK |
|  |  |  |  |  |  |

## Dominican Republic

**Coordinating Investigators:**

<None Entered>

| **Center** | **Principal Investigator** | **Co-Investigator(s)** | **Sub-Investigator(s)** | **Address(es)** | **Institutional Review Board or Ethics Committee Address(es)** |
| --- | --- | --- | --- | --- | --- |
|  |  |  |  |  |  |
| 1226 | Dr. Patricia Alvarez-Felix |  | Rafael Alba-Feriz  Iraquel Cordero  Dr. Roberto Munoz-Louis  Miriam Nova  Cinthia Rivas | Centro Médico Bellas Artes  Calle Abelardo Rodriguez Urdaneta# 11  Suite 205. Gazcue  Santo Domingo, Santo Domingo 0  DOMINICAN REPUBLIC  Centro Médico Bellas Artes  Suite 205, Gascue  Calle Abelardo Rodriguez Urdaneta# 11  Santo Domingo, 00000  DOMINICAN REPUBLIC  Patricia Alvarez Site  Centro Médico Bellas Artes  Suite 205. Gazcue  Calle Abelardo Rodriguez Urdaneta# 11  Santo Domingo, Santo Domingo 00000  DOMINICAN REPUBLIC | CONABIOS  Avenida Bolivar N° 902; La Julia  Santo Domingo, Santo Domingo  DOMINICAN REPUBLIC |
|  |  |  |  |  |  |

## Finland

**Coordinating Investigators:**

<None Entered>

| **Center** | **Principal Investigator** | **Co-Investigator(s)** | **Sub-Investigator(s)** | **Address(es)** | **Institutional Review Board or Ethics Committee Address(es)** |
| --- | --- | --- | --- | --- | --- |
|  |  |  |  |  |  |
| 1248 | Dr. Kari Eklund (Previous PI)  Leena H. Paimela |  | Dr. Kari Eklund  Riitta Koivuniemi | Helsingin Reumakeskus Oy  Bulevardi 22 A  Helsinki, 00120  FINLAND | Helsingin ja Uudenmaan sairaanhoitopiiri  Sisatautien eettinen toimikunta  Biomedicum Helsinki 2 C  PL 705  Helsinki, HUS 00029  FINLAND |
|  |  |  |  |  |  |
| 1249 | Dr. Pentti Jarvinen |  | Sirkka Koskinen  Timo Valiviita | Kiljavan Laaketutkimus Oy  Donnerinkatu 5  Hyvinkaa, 05800  FINLAND | Helsingin ja Uudenmaan sairaanhoitopiiri  Sisatautien eettinen toimikunta  Biomedicum Helsinki 2 C  PL 705  Helsinki, HUS 00029  FINLAND |
|  |  |  |  |  |  |
| 1390 | Pia Isomaki |  | Krista Karstila  Vappu Rantalaiho  Susanna Sihvonen | Tampere University Hospital  TAYS/Reumakeskus/Finn-Medi 1  Biokatu 6  Tampere, 33520  FINLAND | Helsingin ja Uudenmaan sairaanhoitopiiri  Sisatautien eettinen toimikunta  Biomedicum Helsinki 2 C  PL 705  HUS, 00029  FINLAND |
|  |  |  |  |  |  |

## France

**Coordinating Investigators:**

<None Entered>

| **Center** | **Principal Investigator** | **Co-Investigator(s)** | **Sub-Investigator(s)** | **Address(es)** | **Institutional Review Board or Ethics Committee Address(es)** |
| --- | --- | --- | --- | --- | --- |
|  |  |  |  |  |  |
| 1635 | Pr. Francis Berenbaum |  | Julien Champey  Severine Neveu | Hôpital Saint-Antoine  Service de Rhumatologie  184 Rue du Faubourg Saint-Antoine  Paris, 75012  FRANCE | CPP Ile de France V  Hopital Saint-Antoine  184 rue du faubourg Saint-Antoine  Paris, 75012  FRANCE |
|  |  |  |  |  |  |
| 1637 | Dr. Claude-Laurent Benhamou (Previous PI)  Dr. Sylvie Loiseau-Peres |  | Dr. Eric Lespessailles | CHR Orleans, Hopital Porte Madeleine  IPROS SERVICE RHUMATOLOGIE  1 Rue Porte Madeleine  Orleans, 45000  FRANCE | CPP Ile de France V  Hopital Saint-Antoine  184 rue du faubourg Saint-Antoine  Paris, 75012  FRANCE |
|  |  |  |  |  |  |
| 1638 | Prof. Patrice Fardellone |  | Dr. Florence Millot | Hopital Nord  SERVICE DE RHUMATOLOGIE  1, Place Victor Pauchet  Amiens, 80054  FRANCE | CPP Ile de France V  Hopital Saint-Antoine  184 rue du faubourg Saint-Antoine  Paris, 75012  FRANCE |
|  |  |  |  |  |  |
| 1639 | Dr. Minh Nguyen |  | Dr. Christophe Hudry | Universite Paris Descartes - Hopital Cochin  27, Rue du Faubourg Saint Jacques  Paris, 75014  FRANCE | CPP Ile de France V  Hopital Saint-Antoine  184 rue du faubourg Saint-Antoine  Paris, 75012  FRANCE |
|  |  |  |  |  |  |

## Germany

**Coordinating Investigators:**

<None Entered>

| **Center** | **Principal Investigator** | **Co-Investigator(s)** | **Sub-Investigator(s)** | **Address(es)** | **Institutional Review Board or Ethics Committee Address(es)** |
| --- | --- | --- | --- | --- | --- |
|  |  |  |  |  |  |
| 1151 | Dr. med. Rieke Alten |  | Dr. med. Stefan Bieneck  Svetlana Djacenko  Anna Hadjisofokli  Dr. med. Christoph Pohl  Olaf Schroeder | Schlosspark-Klinik Innere Medizin II, Rheumatologie  Heubnerweg 2  Berlin, 14059  GERMANY | Ethikkommission der Saechsischen Landesaerztekammer  Schuetzenhoehe 16  Dresden, 01099  GERMANY |
|  |  |  |  |  |  |
| 1152 | Prof. Dr. med. Juergen Wollenhaupt |  | Dr. med. Michael Alexander Argirov  Dr. med. Andrea Binda  Dr. Nicole Boettcher  Dr. med Andrea Everding  Dr. Kathrin Fiege  Dr. med. Ines Fitschen  Dr. Priscillia Wilma Lau-Tchambo  Dr. med. Ulrike Schnoor  Alexander von Alt-Stutterheim  Dr. med. Wolfgang Winter | Schoen Klinik Hamburg - Eilbek, Abt. fuer Rheumatologie  Dehnhaide 120  Hamburg, 22081  GERMANY | Ethikkommission bei der Saechsischen Landesaerztekammer  Schuetzenhoehe 16  Dresden, 01099  GERMANY |
|  |  |  |  |  |  |
| 1157 | Prof. Dr. med. Christoph Baerwald |  | Dr. Sybille Arnold  Prof. em. Dr. med. Holm Haentzschel  Dr. Martin Hecker  Marco Krasselt  Dr. Olga Malysheva  Dr. Matthias Pierer  Prof. Dr. med. Ulf Wagner | Universitaetsklinikum Leipzig AoeR, Department fuer Innere Medizin  Sektion Rheumatologie / Gerontologie, Studienambulanz Rheumatologie  Liebigstr. 20  Leipzig, 04103  GERMANY | Ethikkommission der Saechsischen Landesaerztekammer  Schuetzenhoehe 16  Dresden, 01099  GERMANY |
|  |  |  |  |  |  |
| 1158 | Prof. Dr. med. Gerd-Ruediger Burmester |  | Dr. med. Tobias Alexander  Hans Bastian  Dr. med. Rene Dziurla  Dr. med. Eugen Feist  Claudia Kedor  Dr. Cornelia D. Spies  Jan Zernicke | Charite Campus Mitte, Medizinische Klinik mit Schwerpunkt Rheumatologie und Klinische Immunologie  Abteilung Neue Therapien  Chariteplatz 1  Berlin, 10117  GERMANY | Ethikkommission der Saechsischen Landesaerztekammer  Schuetzenhoehe 16  Dresden, 01099  GERMANY |
|  |  |  |  |  |  |
| 1170 | Dr. Leonore Unger |  | Dr.med. Anke Doering  Dr.med. Markus Enderlein  Dr.med. Marten Kayser  Sylvia Link  Dr. Eva-Maria Wagner | Krankenhaus Friedrichstadt, I. Medizinische Klinik  Friedrichstrasse 41  Dresden, 01067  GERMANY | Ethikkommission der Saechsischen Landesaerztekammer  Schuetzenhoehe 16  Dresden, 01099  GERMANY |
|  |  |  |  |  |  |
| 1269 | Prof. Dr. med. Hubert Nuesslein |  |  | Arztpraxis, Internist - Rheumatologie  Kontumazgarten 4  Nuernberg, 90429  GERMANY | Ethikkommission der Saechsischen Landesaerztekammer  Schuetzenhoehe 16  Dresden, 01099  GERMANY |
|  |  |  |  |  |  |
| 1311 | Prof. Dr. med. Hendrik Schulze-Koops |  | Dr. med. Mathias Gruenke  Dr. med. Matthias Witt | Klinikum der Universitaet Muenchen, Campus Innenstadt, Rheuma-Einheit  Pettenkoferstr. 8a  Muenchen, 80336  GERMANY | Ethikkommission der Saechsischen Landesaerztekammer  Schuetzenhoehe 16  Dresden, 01099  GERMANY |
|  |  |  |  |  |  |
| 1421 | Dr. Ulrich Schoo |  | Dr. Georg Huebner | Schwerpunktpraxis fuer Rheumatologie  Sprickmannstr. 36  Rheine, 48431  GERMANY | Ethikkommission der Saechsischen Landesaerztekammer  Schuetzenhoehe 16  Dresden, 01099  GERMANY |
|  |  |  |  |  |  |
| 1422 | Prof. Dr. Juergen Braun |  | Dr. Friedrich Dybowski  Dr. Claas Fendler  Dr. med. Frank Heldmann  Dr. med. Uta Kiltz  Dr. Ertan Saracbasi-Zender | St. Josefs-Krankenhaus, Rheumazentrum Ruhrgebiet  Landgrafenstr. 15  Herne, 44652  GERMANY | Ethikkommission der Saechsischen Landesaerztekammer  Schuetzenhoehe 16  Dresden, 01099  GERMANY |
|  |  |  |  |  |  |
| 1424 | Prof. Dr. Hans-Peter Tony |  | Dr. Martin Feuchtenberger  Dr. Ottar Gadeholt  Dr. Stefan Kleinert  Yvonne Kochler  Dr. med. Silke Osiek  Dr. Eva Ostermeier  Dr. Petra Roll  Eva Christina Scharbatke  Dr. med. Marc Schmalzing  Dr. med. Sebastian Matthias Hermann Schuh | Universitaetsklinikum Wuerzburg  Medizinische Klinik II, Rheumatologie/Immunologie, A3.-1.923  Oberduerrbacherstr. 6  Wuerzburg, 97080  GERMANY | Ethikkommission der Saechsischen Landesaerztekammer  Schuetzenhoehe 16  Dresden, 01099  GERMANY |
|  |  |  |  |  |  |
| 1425 | Dr. Siegfried Wassenberg |  | Peter Fretter  Dr. med. Markus Jost  Dr. Dorothea Longerich-Scheuss  Dr. Georg W. Raspe  Dr. med. Ralf Weier | Evangelisches Fachkrankenhaus Ratingen gGmbH  Rosenstr. 2  Ratingen, 40882  GERMANY | Ethikkommission der Saechsischen Landesaerztekammer  Schuetzenhoehe 16  Dresden, 01099  GERMANY |
|  |  |  |  |  |  |
| 1443 | Prof. Dr. med. Harald Burkhardt |  | Dr. med. Frank Behrens  Dr. med. Michaela Kohm  Dr. med. Nicola Reuschling | J.W.-Goethe-Universitaetsklinik, Medizinische Klinik II, Abteilung fuer Rheumatologie  Theodor-Stern-Kai 7  Frankfurt am Main, 60590  GERMANY | Ethikkommission der Saechsischen Landesaerztekammer  Schuetzenhoehe 16  Dresden, 01099  GERMANY |
|  |  |  |  |  |  |
| 1444 | Dr. med. Reiner Kurthen |  | Tom Kurthen | Privat-Praxis, Rheumatologie (P515)  Karlsgraben 15  Aachen, 52064  GERMANY | Ethikkommission der Saechsischen Landesaerztekammer  Schuetzenhoehe 16  Dresden, 01099  GERMANY |
|  |  |  |  |  |  |
| 1523 | Dr. Juergen Rech |  | Stephanie Finzel  Dr. med. Axel Hueber  Dr. Annja Patricia Charlotte Reisch  Dr. med. Monika Ronneberger  Dr. Jochen Wacker | Universitaetsklinikum Erlangen Medizinische Klinik 3  Department of Medicine 3, Rheumatology and Clinical Immunology  Ulmenweg 18  Studienambulanz  Erlangen, 91054  GERMANY | Ethikkommission der Saechsischen Landesaerztekammer  Schuetzenhoehe 16  Dresden, 01099  GERMANY |
|  |  |  |  |  |  |
| 1525 | PD Dr. Andrea Rubbert-Roth |  | Prof. Efim Benenson  Marco Herling  Dr Stefan Kraemer  Dr. Kseniya Maizus  Dr. Thomas Rath  Jasemine Saech  Dr. Roland Ullrich | Universitaetsklinikum Koeln Klinik I fuer Innere Medizin, Haus 16, 1. OG, Raum 1.008  Kerpener Str. 62  Koeln, 50937  GERMANY | Ethikkommission der Saechsischen Landesaerztekammer  Schuetzenhoehe 16  Dresden, 01099  GERMANY |
|  |  |  |  |  |  |
| 1526 | Dr. med. Sylke Wagner |  | Dr. med. Thomas Linde | Schwerpunktpraxis Rheumatologie  FAE Innere Medizin / Rheumatologie  Ludwig-Wucherer-Strasse 10,  Halle, 06108  GERMANY | Ethikkommission der Saechsischen Landesaerztekammer  Schuetzenhoehe 16  Dresden, 01099  GERMANY |
|  |  |  |  |  |  |
| 1530 | Dr. Helmut Soerensen |  | Dr. Bianka Andermann | Ambulantes Rheumazentrum Dr. Soerensen  Argentinische Allee 42  Berlin, 14163  GERMANY | Ethikkommission der Saechsischen Landesaerztekammer  Schuetzenhoehe 16  Dresden, 01099  GERMANY |
|  |  |  |  |  |  |

## Greece

**Coordinating Investigators:**

<None Entered>

| **Center** | **Principal Investigator** | **Co-Investigator(s)** | **Sub-Investigator(s)** | **Address(es)** | **Institutional Review Board or Ethics Committee Address(es)** |
| --- | --- | --- | --- | --- | --- |
|  |  |  |  |  |  |
| 1139 | Prof. Loukas Settas |  | Dr. Paschalis Dalkidis  Dr. Julia Grammatikopoulou  Dr. Andreas Karagiannakidis  Dr. Savvas Kofidis  Markos Kostopoulos  Dr. Charalampos Maskalidis  Nikolaos Tsakonas  Garyfallia Vegoudaki | General CLinic Euromedica "KYANOYS STAVROS"  Rheumatology Department  Kesanli 12  Thessaloniki, 54 636  GREECE  General Hospital Euromedica "KYANOUS STAVROS"  1, Vizyis Vyzantos Street  12, Kesanli Street  Thessaloniki, 54636  GREECE | National Ethics Committee - Ministry of Health  284 Mesogion Avenue  Athens, 15562  GREECE |
|  |  |  |  |  |  |

## Hungary

**Coordinating Investigators:**

<None Entered>

| **Center** | **Principal Investigator** | **Co-Investigator(s)** | **Sub-Investigator(s)** | **Address(es)** | **Institutional Review Board or Ethics Committee Address(es)** |
| --- | --- | --- | --- | --- | --- |
|  |  |  |  |  |  |
| 1142 | Dr. Eleni Kanakaridu (Previous PI)  Dr. Istvan Szombati  Dr. Istvan Szombati (Previous PI) |  | Dr. Eleni Kanakaridu  Dr. Karoly Nagy | Synexus Magyarorszag Kft.  Becsi u. 61  Budapest, H1036  HUNGARY | Egeszsegugyi Tudomanyos Tanacs Klinikai Farmakologiai Etikai Bizottsaga  Farmakologiai Etikai Bizottsaga  Arany J. u. 6-8.  Budapest, H-1051  HUNGARY |
|  |  |  |  |  |  |
| 1616 | Dr. Laszlo Samson |  | Dr. Beata Baksay  Dr. Judit Biro  Dr. Attila Kovacs | MAV Korhaz es Rendelointezet, Reumatologiai szakrendeles  Verseghy F. u. 6-8.  Szolnok, 5000  HUNGARY | Egeszsegugyi Tudomanyos Tanacs Klinikai Farmakologiai Etikai Bizottsaga  Farmakologiai Etikai Bizottsaga  Arany J. u. 6-8.  Budapest, H-1051  HUNGARY |
|  |  |  |  |  |  |
| 1618 | Dr. Gabriella Sulyok |  | Dr. Katalin Bezzegh  Dr. Barbara Buday  Dr. Eniko Kulcsar  Dr. Botond Literati-Nagy  Dr. Jozsef Pauer | Drug Research Center Kft. Reumatologiai Szakrendeles  Ady Endre u. 12.  Balatonfured, 8230  HUNGARY | Egeszsegugyi Tudomanyos Tanacs Klinikai Farmakologiai Etikai Bizottsaga  Farmakologiai Etikai Bizottsaga  Arany J. u. 6-8.  Budapest, H-1051  HUNGARY |
|  |  |  |  |  |  |
| 1632 | Dr. Regina Cseuz |  | Dr. Janos Bartalos  Dr. Marta Megyaszai | Revita Reumatologiai Rendelo  Margit krt. 50-52.  Budapest, 1027  HUNGARY | Egeszsegugyi Tudomanyos Tanacs Klinikai Farmakologiai Etikai Bizottsaga  Farmakologiai Etikai Bizottsaga  Arany J. u. 6-8.  Budapest, H-1051  HUNGARY |
|  |  |  |  |  |  |
| 1659 | Dr. Edit Vereckei |  | Greta Sterba | ORSZAGOS REUMATOLOGIAI ES FIZIOTERAPIAS INTEZET  II REUMATOLOGIAI OSZTALY  FRANKEL LEO U 25 29  Budapest, 1023  HUNGARY | Egeszsegugyi Tudomanyos Tanacs Klinikai Farmakologiai Etikai Bizottsaga  Farmakologiai Etikai Bizottsaga  Arany J. u. 6-8.  Budapest, H-1051  HUNGARY |
|  |  |  |  |  |  |

## India

**Coordinating Investigators:**

<None Entered>

| **Center** | **Principal Investigator** | **Co-Investigator(s)** | **Sub-Investigator(s)** | **Address(es)** | **Institutional Review Board or Ethics Committee Address(es)** |
| --- | --- | --- | --- | --- | --- |
|  |  |  |  |  |  |
| 1376 | Dr. Vineeta Shobha |  | Deepu Chandrappa Boregowda  Ms. Malavi Golla  Mr. Saseedaran Paulty  Dr. Cecil Ross  Savitha Sebastian  Ms. Ramya Singh  Dr. Soumya Umesh | St. John's Medical College Hospital  Sarjapur Road  Bangalore, Karnataka 560 034  INDIA | Institutional Ethical Review Board  St. John's Medical College & Hospital  Sarjapur Road  Bangalore, Karnataka 560 034  INDIA  Institutional Ethical Review Board  St. John's Medical College & Hospital  Sarjapur Road,  Bangalore, Karnataka 56003  INDIA |
|  |  |  |  |  |  |
| 1378 * | Dr. Chandrashekara Srikantiah |  | Dr. Jalaja Ganganna  Dr. Sneha Ramachandra Kulkarni  Mr. Sasi Kumar  Dr. Renuka Panchagnula  Mr. Amarnath Reddy Posini | Chanre Rheumatology & Immunology Center & Research  4th Block, 3rd Stage, Basaveswaranagar  #149, 15th Main NHCL,  Water Tank road,  Bangalore, Karnataka 560 079  INDIA  Chanre Rheumatology & Immunology Center & Research  Chanre Diagnostic Laboratory  #123, 13th Cross  Malleshwaram  Margosa Road,  Bangalore, Karnataka 560003  INDIA | Institutional Ethics Committee, Chanre Rheumatology & Immunology Center & Research  #149, 15th Main, NHCL Water Tank Road  4th Block, 3rd Stage, Basaveswaranagar  Bangalore, Karnataka 560 003  INDIA |
|  |  |  |  |  |  |
| 1379 | Dr. Shrikant Wagh |  | Dr. Ashish Goyal  Ms. Dhanshri Jadhav  Dr. Priscilla Joshi  Ms. Dipika Rao  Ms. Smeeta Salunkhe  Dr. Nuzhat Shaikh  Dr. Ratnamala Swami  Dr. Beenu Varghese  Dr. Pratibha Walde | Jehangir Clinical Development Centre Pvt. Ltd.  Jehangir Hospital  32, Sassoon Road  Pune, Maharashtra 411 001  INDIA | Ethics Committee  Jehangir Clinical Development Centre Pvt. Ltd.  Jehangir Hospital Premises  32  Sassoon Road  Pune, Maharashtra 411 001  INDIA  Jehangir Clinical Development Centre IRB  Institute and Jehangir Development Centre Ethics Committee  Jehangir Hospital Premises,  32, Sassoon Road  Pune, Maharashtra 411 001  INDIA |
|  |  |  |  |  |  |
| 1381 | Dr. Sarath Chandra Mouli Veeravalli |  | Dr. Kiran Kumar Narisepally  Dr. Ravi Kumar Neela  Ms. Rajeshwari Neela  Dr. Sreenivas Reddy Pesaru  Dr. Namrata Sridhar  Dr. Hima Bindu Vasamreddy | Krishna Institute of Medical Sciences Ltd  1-8-31/1  Minister Road  Secunderabad, Andhra Pradesh 500 003  INDIA | Institutional Ethics Committee  Krishna Institute of Medical Sciences  1-8-31/1,  Minister Road  Secunderabad, Andhra Pradesh 500003  INDIA |
|  |  |  |  |  |  |
| 1382 | Dr. Arvind K. Chopra |  | Dr. Pankaj Ajmire  Dr. Sharon Arthur  Mr. Sushil Dighe  Ms. Rubina Kazi  Dr. Vaijayanti Vardhan Lagu-Joshi  Dr. Meenakshi Manerikar  Ms. Sagai Moses  Naisar Nahar  Dr. Pradeep Naik  Dr. Sheetal S. Salvi  Dr. Zaheda Shaikh  Dr. Anupama Sonees | Arthritis Research and Care Foundation  Centre for Rheumatic Diseases  No. 11, Hermes Elegance  1988, Convent Street Camp  Pune, Maharashtra 411 001  INDIA  Center for Rheumatic Diseases  No. 11, Hermes Elegance  1988, Convent Street  Camp  Pune, Maharashtra 411 001  INDIA | CRD Ethics Committee  11, Hermes Elegance  1988, Convent Street Camp  Pune, Maharashtra 411 001  INDIA |
|  |  |  |  |  |  |
| 1384 | Dr. Nimish Sharma (Previous PI)  Dr. Reena I. Sharma  Dr. Reena I. Sharma (Previous PI)  Dr. Vikram I Shah (Previous PI) |  | Dr. Govind Agrawal  Dr. Pankaj Doshi  Dr. Nafisa S. Kathiwala  Ms. Devi Patel  Mr. Kiran Patel  Ms. Usha Shah  Dr. Vikram I Shah  Vishnu Sharma  Dr. Nimish Sharma | Shalby Hospitals  Opp. Karnavati Club  S.G. Road  P.O. Ambawadi Vistar  Ahmedabad, Gujarat 380 015  INDIA  Shalby Hospitals Ltd.  Sarkhej Gandhinagar Highway  Opp. Karnavati Club  Ahmedabad, Gujarat 380054  INDIA | Ethics Committee Shalby Hospitals  Shalby Hospitals  Opp. Karnavati Club,  S. G. Highway,  Ahmedabad, Gujarat 380 015  INDIA |
|  |  |  |  |  |  |
| 1385 * | Dr. Prabha Adhikari |  | Dr. Susan Dsouza  Dr. Deepak R. Madi  Dr. Basavaprabhu Nagabhushana  Dr. John Thomas Ramapuram  Dr. Satish B. Rao  Dr. Ashok K. Shenoy | Kasturba Medical College Hospital  Attavar  Mangalore, Karnataka 575 001  INDIA | Manipal University Ethics Cimmittee  Madhav Nagar  Manipal, karnataka 576 104  INDIA |
|  |  |  |  |  |  |
| 1388 | Dr. Jugal Kishore Kadel |  | Mr. Shaik Aziz  Ms. Shameen Begum  Dr. Manik Dixit  Dr. Sindhu Joshi  Dr. Vikas Kale  Dr. Srinivas Kulkarni  Ms. Madhuri Mahajan  Govindaiah Pulgam  Ms Vasantha Sarathi  Ms. Alahari Subbalakshmi | Mahavir Hospital & Research Centre  10-1-1, Bhagwan Mahavir Marg  A.C. Guards  Hyderabad, Andhra Pradesh 500 004  INDIA | Institutional Ethics Committee for Biomedical Research  Mahavir Hospital and Research Centre  10-1-1  Bhagwan Mahavir Marg  A C Guards  Hyderabad, Andhra Pradesh 500 004  INDIA |
|  |  |  |  |  |  |
| 1439 | Dr. Manoj Kumar Honnakere Venkataiya |  | Dr. Mahesh Basavanna  Dr. Ravi Krishnegowda  Dr. Siddarahalli Somashekara | Department of Orthopedics,Shirdi Sai Hospital  No.519, 2nd Main, Netravathi Street,  Devasandra,  New BEL Road,  Bangalore, Karnataka 560 054  INDIA | CLINICOM  Committee for Evaluation of Protocol for Clinical Research  "Bhooma", No.7, 17 A Cross,  Malleswaram West  Bangalore, Karnataka 560 055  INDIA  CLINICOM Committee for Evaluation of Protocols for Clinical  Research "Bhooma",  No. 7, 17 A Cross  Malleswaram West  Bangalore, 560 055  INDIA |
|  |  |  |  |  |  |
| 1653 | Dr. Sapan Pandya |  | Dr. Surendra Sitasharan Dwivedi  Dr. Shalini Pandya  Dr. Rakesh Solanki | Rheumatic Diseases Clinic  4th Floor, Vedanta Institute of Medical Sciences  Near Samved Hospital  Navrangpura  Ahmedabad, Gujarat 380009  INDIA | Ethics Committee of (CIMS)Care Institute of Medical Sciences  CIMS Hospital  Nr. Shakun Mall, Off.  Science City Road  Sola  Ahmedabad, Gujarat 380060  INDIA |
|  |  |  |  |  |  |

## Ireland

**Coordinating Investigators:**

<None Entered>

| **Center** | **Principal Investigator** | **Co-Investigator(s)** | **Sub-Investigator(s)** | **Address(es)** | **Institutional Review Board or Ethics Committee Address(es)** |
| --- | --- | --- | --- | --- | --- |
|  |  |  |  |  |  |
| 1553 | Prof. Douglas J. Veale |  | Dr Emese Balogh  Leonard Harty  Dr João A Madruga Dias  Carl Orr | St. Vincent's University Hospital  Department of Rheumatology  4 Herbert Avenue  Elm Park  Dublin,  IRELAND | Clinical Research Ethics Committee of the Cork Teaching Hospitals  Lancaster Hall  6 Little Hanover Street  Cork,  IRELAND |
|  |  |  |  |  |  |

## Italy

**Coordinating Investigators:**

<None Entered>

| **Center** | **Principal Investigator** | **Co-Investigator(s)** | **Sub-Investigator(s)** | **Address(es)** | **Institutional Review Board or Ethics Committee Address(es)** |
| --- | --- | --- | --- | --- | --- |
|  |  |  |  |  |  |
| 1059 | Prof. Marco Matucci Cerinic |  | Dr. Francesca Bartoli  Dr. Silvia Bellando Randone  Dr. Cosimo Bruni  Dr. Laura Cometi  Dr. Francesca Nacci  Dr. Francesca Peruzzi  Daniela Pollakova  Dr. Danilo Squatrito | SOD, Medicina Interna 1 e Reumatologia  Villa Monna Tessa  Viale Pieraccini, 18  Firenze, 50139  ITALY | Comitato Etico Area Vasta Centro  Azienda Ospedaliera-Universitaria Careggi di Firenze  Largo Brambilla,3  Firenze, 50134  ITALY |
|  |  |  |  |  |  |
| 1162 | Prof. Maurizio Cutolo |  | Maria Elena Secchi  Alberto Sulli | U.O. Clinica Reumatologica  Dipartimento di Medicina Interna  Universita' degli Studi di Genova  Viale Benedetto XV, 6  Genova, 16132  ITALY | Comitato Etico Azienda Ospedaliera Universitaria San Martino  Largo Rosanna Benzi 10  Genova, 16132  ITALY |
|  |  |  |  |  |  |

## Korea, Republic Of

**Coordinating Investigators:**

<None Entered>

| **Center** | **Principal Investigator** | **Co-Investigator(s)** | **Sub-Investigator(s)** | **Address(es)** | **Institutional Review Board or Ethics Committee Address(es)** |
| --- | --- | --- | --- | --- | --- |
|  |  |  |  |  |  |
| 1136 | Dr. Eun Bong Lee |  | Byoong Yong Choi  Dr. In Ah Choi  Chul Kim  Dr. Hye Won Kim  Jin Hyun Kim  Joonwan Kim  Jaeki Koh  Eun Young Lee  Kiwon Moon  Hyejin Oh  Jin Kyun Park  Hee Jung Ryu  Dr. Yeong-Wook Song  Ran Song  Ji Ae Yang  Myeong Jae Yoon | Seoul National University Hospital  Rheumatology, Internal Medicine  101 Daehang-ro  28 Yeongeon-Dong Jongno-gu  Seoul, 110-744  KOREA, REPUBLIC OF | IRB of Seoul National University Hospital  28 Yeongeon-dong, Jongno-gu  Seoul, 110-744  KOREA, REPUBLIC OF |
|  |  |  |  |  |  |
| 1137 | Dae-Hyun Yoo |  | Dr. Sang-Cheol Bae  So Young Bang  Soo Kyung Cho  Chan-Bum Choi  Young Bin Joo  Jae-Bum Jun  Kyong Hee Jung  Il Kim  Hee-Sun Kim  Jin-Ju Kim  Eun Mi Kim  Young Sam Kim  Tae-Hwan Kim  Kyeong A. Lee  Dr. So Yeon Park  Jeong Ha Park  Song-Ree Park  Yoon-Kyoung Sung | Hanyang University Hospital, Department of Rheumatology  17 Haengdang-dong, Seongdong-gu  Seoul, Republic Of Korea 133-792  KOREA, REPUBLIC OF | IRB of Hanyang University Hospital  Institutional Review Board  17, Haengdang-Dong, Seongdong-Gu  Seoul, 133-792  KOREA, REPUBLIC OF |
|  |  |  |  |  |  |
| 1232 | Soo-Kon Lee |  | Sang-Youn Jung  Dr. Yoon Kang  YouJae Kim  Kwang Hoon Lee  Jin-su Park  Dr. Yong-Beom Park  Hee-Jin Park | Yonsei University College of Medicine, Severance Hospital, Rheumatology, Internal Medicine  134 Shinchon-dong, Seodaemun-gu  Seoul, 120-752  KOREA, REPUBLIC OF | IRB of Severance Hospital  134 Shinchon-dong, Seodaemun-gu  Seoul, 120-752  KOREA, REPUBLIC OF |
|  |  |  |  |  |  |
| 1233 | Won Park |  | Jung Ran Choi  Kowoon Joo  Kyong Hee Jung  Dr. Seong Ryul Kwon  Mie Jin Lim  Chang Gi Moon  Ji Yeol Yoon | Inha University Hospital, Medicine/Rheumatology  7-206, 3-Ga, Sinheung-Dong, Jung-Gu  Incheon, 400-711  KOREA, REPUBLIC OF | IRB of Inha University Hospital  7-206, 3-Ga, Sinheung-Dong, Jung-Gu  Incheon, 400-711  KOREA, REPUBLIC OF |
|  |  |  |  |  |  |
| 1234 | Sung-Hwan Park |  | Ji Hyeon Ju  Dr. Ho-Youn Kim  Seung-Ki Kwok  Kyung-Su Park  Ho Sung Yoon | The Catholic University of Korea, Seoul St. Mary's Hospital Rheumatology, Internal Medicine  505, Banpo-dong, Seocho-gu  Seocho-gu  Seoul, 137-701  KOREA, REPUBLIC OF | The Catholic University of Korea  Seoul St. Mary's Hospital IRB  505 Banpo-dong Seocho-gu  Seoul, 137-701  KOREA, REPUBLIC OF |
|  |  |  |  |  |  |
| 1235 | Dr. Bin Yoo |  | Dr. Seokchan Hong  Dr. Yong Gil Kim  Yoo Jae Kim  Dr. Bon San Koo  Dr. Chang-Keun Lee  Seung Geun Lee  Dr. Min Wook So | Asan Medical Center  88, Olympic-ro 43-gil  Songpa-gu  Seoul, 442-723  KOREA, REPUBLIC OF  Asan Medical Center Rheumatology, Internal Medicine  86, Asanbyeongwon-gil, Songpa-gu  Seoul, 138-736  KOREA, REPUBLIC OF | IRB of Asan Medical Center  86, Asanbyeongwon-gil, Songpa-gu  Seoul, 138-736  KOREA, REPUBLIC OF |
|  |  |  |  |  |  |
| 1236 * | Dr. Eun-Mi Koh (Previous PI)  Hoon-Suk Cha |  | Joong kyong Ahn  Jiwon Hwang  Dr. Jaejoon Lee  Ji Min Oh | Samsung Medical Center, Division of Rheumatology, Department of Medicine  50 Ilwon-dong, Gangnam-Gu  Seoul, 135-710  KOREA, REPUBLIC OF | IRB of Samsung Medical Center  Institutional Review Board  50 Ilwon-Dong, Gangnam-Gu  Seoul, 135-710  KOREA, REPUBLIC OF |
|  |  |  |  |  |  |
| 1237 | Dong Hyuk Sheen  Seung Cheol Shim (Previous PI) |  | Mi Kyoung Lim  Hyo Park | Eulji University Hospital Internal Medicine Rheumatology  1306 Dunsandong, Seogu  Daejeon, 302-799  KOREA, REPUBLIC OF | IRB of Eulji University Hospital  1306 Dunsandong, Seogu  Daejeon, 302-799  KOREA, REPUBLIC OF |
|  |  |  |  |  |  |
| 1265 | Dr. Shin-Seok Lee |  | Sung-Ji Lee  Jeong-Won Lee  Kyung-Eun Lee  Dong Jin Park  Seong-Rye Seo | Chonnam National University Hospital  8, Hak-dong, Song-gu  Gwangju, 501-757  KOREA, REPUBLIC OF | IRB of Chonnam National University Hospital  8, Hak-dong, Dong-gu  Gwangju, 501-757  KOREA, REPUBLIC OF |
|  |  |  |  |  |  |
| 1511 | Jungsik Song  Min-Chan Park (Previous PI) |  | Soo-Jin Chung  Yong-Jin Kwon | Gangnam Severance Hospital/Rheumatology  712 Eonjuro, Gangnam-gu  Seoul, 135-720  KOREA, REPUBLIC OF | IRB of Gangnam Severance Hospital  712 Eonjuro, Gangnam-gu  Seoul, 135-720  KOREA, REPUBLIC OF |
|  |  |  |  |  |  |
| 1512 | Prof. Jung-Yoon Choe |  | Hyun-Young Jung  Ji Hun Kim  Dr. Seong-Kyu Kim  Hwa-Jeong Lee  Dr. Sung-Hoon Park | Daegu Catholic Univ Hosp Medical Center Department of Rheumatology  3056-6 Daemyung-4 dong  Namgu  Daegu, 705-718  KOREA, REPUBLIC OF | IRB of Daegu Catholic University Medical Center  #202, Geumgang Villa, 991-8, Daemyung-10 dong, Nam-gu  Daegu, 705-812  KOREA, REPUBLIC OF |
|  |  |  |  |  |  |
| 1513 | Dr. Won-Tae Chung |  | Sang Yeob Lee  Sung Won Lee | Dong-A University Hospital/ Department of Pharmacy  1, Dongdaeshin-dong, 3-ga, Seo-gu  Busan, 602-715  KOREA, REPUBLIC OF | IRB of Dong-A University Hospital  1, Dongdaesin-dong, 3-ga,Seo-Gu  Busan, 602-715  KOREA, REPUBLIC OF |
|  |  |  |  |  |  |
| 1568 | Dr. Sang-Heon Lee |  | Somi Kim  Dr. Hae-Rim Kim  Dr. Ho-Youn Kim  Jung Hwa Lee | Konkuk University Medical Center, Department of Rheumatology  4-12 Hwayang-dong Gwangjin-gu  Seoul, 143-729  KOREA, REPUBLIC OF | Institutional Review Board, Konkuk University Medical Center  4-12 Hwayang-dong Gwangjin-gu  Seoul, 143-729  KOREA, REPUBLIC OF |
|  |  |  |  |  |  |

## Malaysia

**Coordinating Investigators:**

<None Entered>

| **Center** | **Principal Investigator** | **Co-Investigator(s)** | **Sub-Investigator(s)** | **Address(es)** | **Institutional Review Board or Ethics Committee Address(es)** |
| --- | --- | --- | --- | --- | --- |
|  |  |  |  |  |  |
| 1285 | Dr Heselynn Hussein (Previous PI)  Nor Shuhaila Shahril |  | Dr. Fazirah Abdullah  Dr. Hui Jen Ding  Dr Heselynn Hussein  Dr Eashwary Mageswaren  Dr. Liza Mohd Isa  Ahmad Zaidi Othman  Dr. Shamala Rajalingam | Hospital Putrajaya  Presint 7  Federal Government Administration Centre  Department of Medicine  Putrajaya, Wilayah Persekutuan 62250  MALAYSIA | Medical Research & Ethics Committee  Ministry of Health, c/o NIH Secretariat, Institute for Health Management  Bangsar  Kuala Lumpur, 59000  MALAYSIA |
|  |  |  |  |  |  |
| 1286 | Dr. Suk Chyn Gun |  | Chee Ken Cheah  Dr. Beryl Agnes D'souza  Dr. C.Gandhi K.Chembalingam Pillay  Dr. Ai Lee Lim  Dr. Asmah Mohd  Dr. Liza Mohd Isa  Dr. Nadiah Mohd Noor  Dr. Shalinie Ramanujam | Hospital Tuanku Ja'afar  Department of Medicine  Jalan Rasah  Seremban, 70300  MALAYSIA | Medical Research & Ethics Committee  Ministry of Health, c/o NIH Secretariat, Institute for Health Management  Bangsar  Kuala Lumpur, 59000  MALAYSIA |
|  |  |  |  |  |  |
| 1287 | Dr Swan Sim Yeap |  | Dr. Raveendran Ramachandran | Sime Darby Medical Centre Subang Jaya Sdn Bhd  No. 1, Jalan SS12/1A  Subang Jaya  Selangor, 47500  MALAYSIA | Independent Ethics Committee  Sime Darby Medical Centre Subang Jaya Sdn Bhd  No. 1, Jalan SS12/1A  Subang Jaya, Selangor 47500  MALAYSIA |
|  |  |  |  |  |  |
| 1288 | Dr. Azmillah Rosman |  | Dr. Hilmi Abdullah  Dr. Ramani Arumugam  Dr. Hwee Cheng Chong  Dr. Asmahan Mohamed Ismail  Dr. Ing Soo Lau  Dr. Habiba Mohd Yusoof  Dr. Mollyza Mohd Zain  Dr. Yew Chong Ong  MD Kuan Woon Pang  Sheeren Ch'ng Suyin | Hospital Selayang  Faculty of Medicine  Lebuhraya Selayang-Kepong  Level 11  Batu Caves, 68100  MALAYSIA | Medical Research & Ethics Committee  Ministry of Health, c/o NIH Secretariat, Institute for Health Management  Bangsar  Kuala Lumpur, 59000  MALAYSIA |
|  |  |  |  |  |  |
| 1307 | Dr. Cheng Lay Teh |  | Dr. Wai Hoong Chan  Dr. Yaw Kiet Cheong  Dr. Yin Yin Angela Chia  Dr. Jun Lee  Dr. Jin Shyan Wong | Sarawak General Hospital  Department Of Medicine Jalan Hospital  Jalan Tun Ahmad Zaidi Adruce  Kuching, 93586  MALAYSIA | Medical Research & Ethics Committee  Ministry of Health, c/o NIH Secretariat, Institute for Health Management  Bangsar  Kuala Lumpur, 59000  MALAYSIA |
|  |  |  |  |  |  |
| 1308 | Dr. Rachel Joshua Thundyil  Dr. Yun Yin Chong (Previous PI) |  | Dr. Yun Yin Chong  Dr. Anna Farazilah Mohammad Salleh  Dr. Giri Shan Rajahram  Dr Teck Huat Wong | Queen Elizabeth Hospital  Rheumatology Clinic (Room 2) Medical Clinic  Level 2, New Clinic Block  Kota Kinabalu  Sabah, 88586  MALAYSIA | Medical Ethics & Research Committee  Institute for Health Management  Jalan Rumah Sakit  Bangsar, Kuala Lumpur 59000  MALAYSIA |
|  |  |  |  |  |  |
| 1309 | Dr. Sook Khuan Chow |  | Dr. Suresh V. Nainan George  Dr. Surendren Thuraisingham  Dr. Amir Azlan Zain | Sunway Medical Centre  Pharmacy Department  No. 5, Jalan Lagoon Selatan  Bandar Sunway  Petaling Jaya, Selangor Darul Ehsan 46150  MALAYSIA | Medical Research & Ethics Committee  Ministry of Health, c/o NIH Secretariat, Institute for Health Management  Jalan Rumah Sakit  Bangsar  Kuala Lumpur, 59000  MALAYSIA  Sunway Medical Centre Independent Research Ethics Committee (SREC)  Clinical Research Centre  Sunway Medical Centre  No. 5, Jalan Lagoon Selatan  Bandar Sunway, Petaling Jaya, Selangor Darul Ehsan 46150  MALAYSIA |
|  |  |  |  |  |  |

## Mexico

**Coordinating Investigators:**

Maria Dolores Alonso-Martinez (Previous Coordinating Investigator)

| **Center** | **Principal Investigator** | **Co-Investigator(s)** | **Sub-Investigator(s)** | **Address(es)** | **Institutional Review Board or Ethics Committee Address(es)** |
| --- | --- | --- | --- | --- | --- |
|  |  |  |  |  |  |
| 1055 | Dr. Virginia Pascual-Ramos |  | Lucia Comellas - Kirkerup  Mario Cesar Ocampo-Torres  Dr. Marina Rull-Gabayet  Tania Sanchez-Hernandez  Karina Santana  Dr. Karina Santana De Anda  Yefte Efrain Silva-Lopez  Dr. Pablo Villasenor-Ovies | Instituto Nacional de Ciencias Medicas y Nutricion Salvador Zubiran  Departamento de Inmunologia y Reumatologia  Vasco de Quiroga 15  Col Seccion XVI Tlalpan  Mexico, DF 14000  MEXICO | Instituto Nacional de Ciencias Medicas y Nutricion Salvador Zubiran  Comite Institucional de Investigacion Biomedica en Humanos  VASCO DE QUIROGA 15  COL SECCION XVI TLALPAN  Mexico, DF 14000  MEXICO |
|  |  |  |  |  |  |
| 1056 * | Dr. Ruben Burgos-Vargas |  | Dr. Conrado Garcia-Garcia | Hospital General de México  Servicio de Reumatología  Dr. Balmis No. 148  Col. Doctores  Mexico, D.F. 06726  MEXICO | Comisión de Etica  Hospital General de México  Dr. Balmis No. 148  Col. Doctores  México, D.F. 06726  MEXICO  Comisión de Investigación  Hospital General de México  Dr. Balmis No. 148  Col. Doctores  México, D.F. 06726  MEXICO |
|  |  |  |  |  |  |
| 1057 | Dr. Mario H. Cardiel-Rios |  | Maria Dolores Alonso-Martinez  Dr. Hilda Leticia Avila-Martinez  Dr. Gabriela Calderon-Izazaga  Rosa M. Larios-Garcia  Rosa Janete Ramirez-Tapia  Cinthya Salazar-Carbajal  Raquel Sanchez-Hernandez | Star Medica Centro de Investigacion Clinica de Morelia  SC Virrey de Mendoza 1998-502/416 Felix Ireta  Morelia, MICHOACAN 58070  MEXICO | Star Medica  Comité de la Calidad de la Atencion Medica, Credenciales, Ensenanza,  Investigacion, Capacitacion y Etica, Evaluacion del Expediente Clinico  Virrey de Mendoza 2000  Felix Ireta  Morelia, Michoacan 58070  MEXICO |
|  |  |  |  |  |  |
| 1181 | Dr. Daniel Xavier Xibille-Friedmann |  | Dr. Mariana Alvarez-Fuentes  Donaji Domiguez  Sara Eugenia Hernandez-Gongora  Javier Arturo Linares-Garcia | Fundacion El Hospitalito  Av. Lazaro Cardenas 104  Col. Jiquilpan Cuernavaca  Morelos, 62170  MEXICO  INOVAMED Hospital  Cuauhtemoc 203-109  Colonia Lomas de la Selva  Cuernavaca, Morelos 62270  MEXICO | Comité de Ética del Hospital INOVAMED  Cuauhtemoc 305  Colonia Lomas de la Selva  Cuernavaca, Morelos 62270  MEXICO |
|  |  |  |  |  |  |
| 1183 | Dr. Manuel Robles-San Roman |  | Maria Eugenia Davalos-Zugasti  Dr. Maria Consuelo Medina-Puente  Dr. Miguel Angel Torres-Rodriguez | Centro Medico Toluca  Av. Benito Juarez Norte 135-B Consultorio 308 Torre II  Col Barrio de San Mateo  Metepec, Estado de Mexico 52140  MEXICO | Comité de Bioética e Investigación  Facultad de Medicina de la Universidad Autónoma del Estado de México  Paseo Tollocan Esquina Jesús Carranza  Colonia Moderna de la Cruz  Toluca, Estado de México 50120  MEXICO |
|  |  |  |  |  |  |
| 1188 | Dr. Reyna Manuela Bustamante-Gonzalez |  | Dalia de Leon-Murillo  Dr. Norma Alicia Martinez-Trejo  Antonio Sanchez-Gonzalez | Centro de Investigacion Clinica Especializada, SC  Tlatetilpa No. 24  Colonia Barrio San Lucas  Coyoacan, Mexico D.F. 04030  MEXICO | Comite Bioetico para la Investigacion Clinica S.C.  Puebla 422  Despacho 4  Col. Roma Sur  MEXICO, DISTRITO FEDERAL 06700  MEXICO |
|  |  |  |  |  |  |
| 1572 | Dr. Cesar Francisco Pacheco-Tena |  | Dr. Edgardo Munoz-Esteves  Dr. Hugo Parra-Ruiz  Dra. Adelfia Urenda-Quezada  Dr. Omar Villarreal Dominguez | Hospital Christus Muguerza del Parque  Calle 14 1610 A Colonia Centro,  Chihuahua, Chihuahua 31000  MEXICO | Comite de Etica e Investigacion del Hospital Christus Muguerza del Parque  Calle Dr. Pedro Leal Rodriguez y de la Llave  Chihuahua, Chihuahua 31000  MEXICO |
|  |  |  |  |  |  |
| 1573 | Dr. Mario A. Garza-Elizondo |  | Dr. Jorge Antonio Esquivel-Valerio  Dr. Diana Elsa Flores-Alvarado  Dr. Jacqueline Rodriguez-Amado  Dr. Cassandra Michelle Skinner-Taylor  Dra. Brenda Roxana Vazquez-Fuentes | Hospital Universitario Jose Eleuterio Gonzalez  Gonzalitos 235 Norte  Colonia Mitras Centro  Monterrey, Nuevo Leon 64020  MEXICO | Comite de Etica Facultad de Medicina de la UANL y Hospital Universitario Dr. Jose Eleuterio Gonzalez  Av. Francisco I Madero Pte s/n y Dr. E Aguirre Pequeno  Col. Mitras Centro  Monterrey, Nuevo Leon 64460  MEXICO |
|  |  |  |  |  |  |
| 1574 | Dr. Diego Cesar-Ricardo Ramos-Remus |  | Dionisio Castillo  Dr. Sergio Duran-Barragan  Dr. Adriana Sanchez-Ortiz | Unidad de Investigacion en Enfermedades Cronico Degenerativas  Colomos 2292,  COLONIA PROVIDENCIA  Guadalajara, Jalisco 44620  MEXICO | Comite de Bioetica de la Unidad de Investigacion en Enfermedades Cronico-Degenerativas  Colomos 2292  Col. Providencia  Guadalajara, Jalisco 44620  MEXICO |
|  |  |  |  |  |  |
| 1575 | Dr. Jesus Ernesto Santana-Sahagun (Previous PI)  Dr. Maria Cristina Saldate-Alonso |  | Carla Yolanda Corti-Saldate  Francis B. Gabbai-Laval  Alma Guadalupe Lopez-Beltran  Dr. Marisela Lopez-Vazquez  Dr. Jesus Ernesto Santana-Sahagun | Centro de Investigacion del Noroeste S.C.  Blvd. Sanchez Taboada 9250-28 Zona Rio  Tijuana, Baja California 22010  MEXICO | Comision de Investigacion y Etica Centro Medico Nova  Avenida Guadalupe Victoria 9308 Zona Rio  Tijuana, Baja California 22010  MEXICO |
|  |  |  |  |  |  |
| 1623 | Dr. Rafael Horacio Cornejo-Ballesteros |  | Dr. Marco Antonio Escobedo-Madrigal  Benigno Figueroa-Nunez | Clinica de Enfermedades Cronicas y de Procedimientos Especiales (CECYPE)  Fr. Bernardino de SahagÃºn 101  Fraccionamiento Mirador de Punhuato  Morelia, 58249  MEXICO | COMISION DE ETICA DEL HOSPITAL GENERAL DE MEXICO  DR. BALMIS # 148 COLONIA DOCTORES  MEXICO CITY, D.F. 06726  MEXICO  Comision de Etica e Investigacion CECYPE  Comision de Etica e Investigacion  Fray Bernardino de Sahagun 101  Fraccionamiento Mirador del Punhuato  Morelia, Michoacan 58249  MEXICO |
|  |  |  |  |  |  |
| 1625 | Dr. Isaura Maria Rodriguez-Torres |  | Dr. Carlos De la Cueva-Rodriguez | Unidad de Enfermedades Reumaticas y Cronico Degenerativas SC  Matamoros 798 West Torreon  Torreon, Coahuila 27000  MEXICO | Comite de Bioetica de la Facultad de Medicina UA de C  Morelos 900 Oriente  Colonia Centro  Torreon, Coahuila 27000  MEXICO |
|  |  |  |  |  |  |
| 1626 | Dr. Jose Arturo Covarrubias-Cobos |  | Dr. Francisco Avila-Zapata  Hugo Israel Segovia-Escalante | Centro Medico de las Americas  Consultorio 115  Calle 54 365 x 33 Avenida Perez Ponce, Centro  Merida, Yucatan 97000  MEXICO | Comite de Etica del Centro Medico de las Americas  Calle 54 365 x 33A  Avenida Perez Ponce  Merida, Yucatan 97000  MEXICO  Unidad de Atencion Medica e Investigacion en Salud S.C.  Calle 15 491  Colonia Altabrisa  Merida, Yucatan 97134  MEXICO |
|  |  |  |  |  |  |
| 1627 | Dr. Carlos Abud-Mendoza |  | Dr. Enrique Cuevas-Orta  Dr. Ricardo Moreno-Valdes  Dr. Martin Saldana-Barnad  Dra. Eva Nina Santillan-Guerrero | Hospital Central Dr. Ignacio Morones Prieto Unidad Regional de Reumatologia y Osteoporosis  Avenida Venustiano Carranza 2395  Zona Universitaria  San Luis Potosi, 78240  MEXICO | Comite de Investigacion y Etica  Hospital Central Dr. Ignacio Morones Prieto  Avenida Venustiano Carrranza 2395  Zona Universitaria  San Luis Potosi, SLP78240  MEXICO |
|  |  |  |  |  |  |
| 1628 | Dr. Maria de Lourdes C. Sanchez-Gonzalez |  | Dr. Leonor Adriana Barile-Fabris  Dr. Beatriz Alicia De Cortina-Camou | Hospital Angeles del Pedregal  Periférico Sur #3697. Consultorio 570. Col. Héroes de Padierna  Consultorio Sotano 33  Mexico, 10700  MEXICO | Comite de Bioetica del Instituto de Ciencias Biomedicas Angeles  Camino a Santa Teresa 1055  Torre Especialidades piso 14  Heroes de Padierna  Mexico, DF 10700  MEXICO |
|  |  |  |  |  |  |
| 1629 | Dr. Roman Cardona-Cabrera |  | Maria Isabel Segura-Esquivel | Centro Reumatologico de Queretaro  Heraclio Cabrera 52  Colonia Ensueno  Queretaro, Queretaro 76178  MEXICO | Comite Bioetico para la Investigacion Clinica S.C.  Puebla 422  Despacho 4  Col. Roma Sur  MEXICO, DISTRITO FEDERAL 06700  MEXICO |
|  |  |  |  |  |  |
| 1642 | Dr. Hilario Ernesto Avila - Armengol |  | Yunuen Elizabeth Amezcua-Heredia  Hugo Antonio Ascencio Hernandez  MD Myrla Yaneth Avendaño-Avalos  Dr. Victoria del Carmen Padilla-Rios | Instituto Jaliscience de Investigación Clínica SA de CV  Clinica de Osteoporosis y Reumatologia  Av. De La Paz 1917 y 1919  Colonia Americana  Guadalajara, 44160  MEXICO | Comité de Etica del Instituto Jalisciense de Investigacion Clinica S.A de C.V.  Calle Penintenciaria # 20  Col Centro  Guadalajara, Jalisco 44100  MEXICO |
|  |  |  |  |  |  |
| 1654 | Fedra Consuelo Irazoque-Palazuelos |  | Dr. Lilia Andrade Ortega  Dr. Illiana G. Leyva  Dr. Nancy Guadalupe Rodriguez-Inzunza  Natalia Vera  Natalia Paz Vera Olguin  Dr. Rosario del Carmen Zavala-Cueva | Centro de Investigacion y Tratamiento Reumatologico S.C.  General Cano 130  Colonia San Miguel Chapultepec  Del. Miguel Hidalgo  Mexico, D.F. 11850  MEXICO  Hospital Angeles Mocel  Gelati 29, Consultorio 302, Colonia San Miguel Chapultepec  Mexico City, 11850  MEXICO | Comite Bioetico para la Investigacion Clinica S.C.  Puebla 422  Despacho 4  Col. Roma Sur  MEXICO, DISTRITO FEDERAL 06700  MEXICO |
|  |  |  |  |  |  |
| 1655 | Dr. Juan Cruz Rizo-Rodriguez |  | Victor Manuel Dimas-Pecina  Nestor Saul Hernandez-Milan  Bertha Lavin-Agoitia  Daniela Trevino | Centro de Alta Especialidad en Reumatologia e Investigacion del Potosi S.C. Consultorio 600 6to Piso  Hospital Angeles Centro Medico del Potosi  Antonio Aguilar 155  Colonia Burocratas del Estado  San Luis Potosi, 78200  MEXICO | Comite Bioetico para la Investigacion Clinica S. C.  Puebla 422-4  Colonia Roma Sur, Delegacion Cuauhtemoc  Mexico, D.F. 06700  MEXICO |
|  |  |  |  |  |  |

## New Zealand

**Coordinating Investigators:**

<None Entered>

| **Center** | **Principal Investigator** | **Co-Investigator(s)** | **Sub-Investigator(s)** | **Address(es)** | **Institutional Review Board or Ethics Committee Address(es)** |
| --- | --- | --- | --- | --- | --- |
|  |  |  |  |  |  |
| 1593 | Dr. Alan Doube |  | Dr. Trisha Holmes  Mrs. Joanna Schollum  Dr. Kamal Kishor Solanki  Dr. Douglas Harvie Nash White | Waikato Hospital, Rheumatology  Pembroke Street  Hamilton, 3204  NEW ZEALAND | Multi-region Ethics Committee  Ministry of Health  133 Molesworth Street  Wellington, 6145  NEW ZEALAND  Multi-Region Ethics Committee  Ministry of Health  133 Molesworth Street  Wellington, 6145  NEW ZEALAND |
|  |  |  |  |  |  |
| 1594 | Dr. Daniel Wai Tho Ching |  | Dr. Christine Joan Auton  Dr. Clare Mary Davenport  Dr. Christine Anne Gloag  Dr. Matthew John Hills | Timaru Rheumatology Studies  28 Carlisle Place  Timaru, 7910  NEW ZEALAND | Multi-region Ethics Committee  Ministry of Health  133 Molesworth Street  Wellington, 6145  NEW ZEALAND |
|  |  |  |  |  |  |
| 1596 | Dr. Nigel Leslie Gilchrist |  | Dr. Peter W. Moller  Dr. Marina Dawn Sew Hoy | The Canterbury Geriatric Medical Research Trust, c/- The Princess Margaret Hospital  Cashmere Road  Christchurch, 8022  NEW ZEALAND | Multi-region Ethics Committee  Ministry of Health  133 Molesworth Street  Wellington, 6145  NEW ZEALAND |
|  |  |  |  |  |  |

## Peru

**Coordinating Investigators:**

<None Entered>

| **Center** | **Principal Investigator** | **Co-Investigator(s)** | **Sub-Investigator(s)** | **Address(es)** | **Institutional Review Board or Ethics Committee Address(es)** |
| --- | --- | --- | --- | --- | --- |
|  |  |  |  |  |  |
| 1416 * | Dr. Felix J. Romero |  | Dr. Jorge Mario Alarcon Piana  Dr. Fernando Atencia  Dr. Federico E. Elguera | Instituto Peruano del Hueso y la Articulacion SAC-Privado-Lima/Centro de Investigacion IPHAR  Av. Petit Thouars 3954  San Isidro  Lima, L-27  PERU | Comite Institucional de Etica de la Universidad Peruana Cayetano Heredia  Av. Honorio Delgado 430.  Urb. Ingenieria. San Martin de Porres.  Lima, Lima L31  PERU |
|  |  |  |  |  |  |
| 1417 | Dr. Ariel Reynaldo Salinas |  | Dr. Graciela Victoria Alonso | Hospital Nacional IV Alberto Sabogal Sologuren  Unidad de Investigacion, ESSALUD. Red Asistencial Sabogal  Jr. Colina 1081  Bellavista  Callao, C-02  PERU | Comite De Bioetica De La Red Asistencial Sabogal - Essalud  Jr. Colina 1081  Bellavista, Callao Callao 02  PERU |
|  |  |  |  |  |  |
| 1529 * | Dr. Risto Perich |  | Dr. Janet Grisel Huaman  Dr. David Leon  Dr. Mariela Medina | Centro Medico Corpac/Investigaciones en Reumatologia  Av. Gonzales Olaechea 165 Urbanizacion Corpac  San Isidro  Lima, Lima L-27  PERU | Comite Institucional de Etica de la Universidad Peruana Cayetano Heredia  Av. Honorio Delgado 430.  Urb. Ingenieria. San Martin de Porres.  Lima, Lima L31  PERU |
|  |  |  |  |  |  |

## Philippines

**Coordinating Investigators:**

<None Entered>

| **Center** | **Principal Investigator** | **Co-Investigator(s)** | **Sub-Investigator(s)** | **Address(es)** | **Institutional Review Board or Ethics Committee Address(es)** |
| --- | --- | --- | --- | --- | --- |
|  |  |  |  |  |  |
| 1408 | Dr. Sandra T.G.V. Navarra |  | Dr. Ginger Alden I. Cabasan  Dr. Maria Cristina C. Tolin  Dr. Eugene A. Uy | Hospital Research Center, University of Santo Tomas Hospital  Ground Floor, Clinical Division Building  Espana Street  Manila, 1008  PHILIPPINES  University of Santo Tomas Hospital  Hospital Research Center Clinical Division Bldg.  6th Floor  Espana  Manila, Phillipines 1008  PHILIPPINES | Institutional Review Board  Rm 426, 4th Floor, Clinical Division Building  University of Santo Tomas Hospital  Espana Blvd.,  Manila, 1008  PHILIPPINES |
|  |  |  |  |  |  |
| 1409 | Dr. Emmanuel C. Perez |  | Dr. Andrei Rhoneil M. Rodriguez | University of Perpetual Help  Rizal Dalta Medical Center Research Room  7th Floor  Medical Arts Building Alabang-Zapote Road  Las Piñas City, 1742  PHILIPPINES | Institutional Ethics Review Board  7th Floor, Medical Arts Building  University of Perpetual Help System Dalta  Alabang - Zapote Road  Las Piñas City, Philippines 1742  PHILIPPINES |
|  |  |  |  |  |  |
| 1410 | Dr. Auxencio Lorenz A. Lucero Jr. |  | Dr. Jenny Rubio Bicol  Franco Onswald C. Ruberica | 1. De La Salle University Health Sciences Campus Clinical Epidemiology Unit  3rd Floor The Angelo King Medical Research Center  Congressional Road  Dasmarinas, Cavite 4114  PHILIPPINES | De La Salle Health Sciences Insitute Independent Ethics Committee  Room 6301  De La Salle Angelo King Medical Research Center  Congressional Avenue  Dasmari¿as, Cavite, Philippines 4114  PHILIPPINES |
|  |  |  |  |  |  |
| 1411 | Dr. Edgar B. Ramiterre |  | Maria Aurora Narisma | Brokenshire Integrated Health Ministries,  Inc. Brokenshire Memorial Hospital  Room 237  Madapo,  PHILIPPINES  Section of Rheumatology  Department of Internal Medicine  Davao Medical Center  Bajada  Davao City, 8000  PHILIPPINES  Southern Philippines Medical Center (SPMC) (Davao Medical Center (DMC))  Department of Internal Medicine  Jp Laurel Avenue  Bajada  Davao, 8000  PHILIPPINES | Ethics Committee  Southern Philippines Medical Center  Bajada, Davao City, 8000  PHILIPPINES |
|  |  |  |  |  |  |
| 1412 | Dr. Allan E. Lanzon |  | Rosalia Manaloto | Mary Mediatrix Medical Center  3rd Floor HB Calleja Bldg.  J.P. Laurel Highway  Lipa City, Batangas 4217  PHILIPPINES | Institutional Review Board  Mary Mediatrix Medical Center  J.P. Laurel Highway  Lipa City, Batangas 4217  PHILIPPINES  Mary Mediatrix Medical Center JP  Narcisa Sonia Comia,MD  Laurel Highway,  Lipa City, Batangas  PHILIPPINES |
|  |  |  |  |  |  |
| 1413 | Dr. Harold Michael P. Gomez |  | Dr. Gemalyn F. Pineda-Gueco | Angeles University Foundation Medical Center  3rd floor Infection Control and Research Room  Angeles, Pampanga 2009  PHILIPPINES | Angeles University Foundation  Anita S. Sanchez, MD  Laurel Highway,  Lipa City, Batangas  PHILIPPINES  Institutional Review Board  College of Medicine  3rd Floor San Agustin Building  Angeles University Foundation Medical Center  McArthur Highway  Angeles City, Pampanga 2009  PHILIPPINES |
|  |  |  |  |  |  |
| 1414 | Josephine Abao Lim |  | Dr. Ronald Eullaran | Chong Hua Medical Arts Building  Room 102  J. Llorente Extension  Cebu City, 6000  PHILIPPINES | Institutional Review Board  Chong Hua Hospital  Fuente Osmeña Blvd.  Cebu City, Philippines 6000  PHILIPPINES |
|  |  |  |  |  |  |

## Poland

**Coordinating Investigators:**

Dr. Robert Rupinski

| **Center** | **Principal Investigator** | **Co-Investigator(s)** | **Sub-Investigator(s)** | **Address(es)** | **Institutional Review Board or Ethics Committee Address(es)** |
| --- | --- | --- | --- | --- | --- |
|  |  |  |  |  |  |
| 1060 | Prof. Janusz Badurski |  | Dr. Med. Stefan Daniluk  Dr. Anna Maria Jarmoc  Dr. Elzbieta Zofia Jeziernicka  Dr. Nonna Anna Nowak | NZOZ i Chorob Kostno-Stawowych J. Badurski Sp.J ul  Stoleczna 7  Bialystok, 15- 879  POLAND | Komisja Bioetyczna przy Okregowej Izbie Lekarskiej  ul. Swietojanska 7  Bialystok, 15-082  POLAND |
|  |  |  |  |  |  |
| 1061 | Dr. Wieslawa Porawska |  | Dr. Kamilla Klama  Dr. Wlodzimierz Piotrowski | Centrum Badan Klinicznych s.c. Wieslawa Porawska, Lukasz Porawski  Ul. Sniadeckich 7/2  Poznan, 60-773  POLAND | Komisja Bioetyczna przy Okregowej Izbie Lekarskiej  ul. Swietojanska 7  Bialystok, 15-082  POLAND |
|  |  |  |  |  |  |
| 1063 | Dr. Artur Racewicz |  | Dr. Helena Denisiuk  Dr. Malgorzata Fiedorczyk  Dr. Sylwia Kalinko  Dr. Krystyna Kuc  Dr. Dorota Laszcz  Dr. Anna Madrzak  Dr. Sylwia Izabela Raczynska  Dr. Jerzy Supronik | NZOZ "Osteo-Medic"s.c. Artur Racewicz, Jerzy Supronik  ul. Wiejska 81  Bialystok, 15-354  POLAND  ZDROWIE OSTEO-MEDIC s. c.  Lidia i Artur Racewicz, Agnieszka i Jerzy Supronik  ul. Wiejska 81  Bialystok, 15-354  POLAND | Komisja Bioetyczna przy Okregowej Izbie Lekarskiej  ul. Swietojanska 7  Bialystok, 15-082  POLAND |
|  |  |  |  |  |  |
| 1064 | Dr. Jaroslaw Marcinkiewicz |  | Dr. Iwonna Lajborek-Czyz  Dr. Ewa Wojtowicz  Dr. Romana Zak | Wojewodzki Zespol Reumatologiczny im. dr Jadwigi Titz-Kosko  ul. Grunwaldzka 1/3  Sopot, Poland 81-759  POLAND | Komisja Bioetyczna przy Okregowej Izbie Lekarskiej  ul. Swietojanska 7  Bialystok, 15-082  POLAND |
|  |  |  |  |  |  |
| 1160 | Dr. Zofia Ruzga |  | Dr. Radoslaw Janiak  Ewa Jazwinska-Tarnawska  Ewa Krecipro-Nizinska  Dr. Anna Sidorowicz-Bialynicka  Dr. Renata Wojtala | SYNEXUS Polska Sp. z.o.o.  ul. Swobodna 8a  Wroclaw, 50-088  POLAND | Komisja Bioetyczna przy Okregowej Izbie Lekarskiej  ul. Swietojanska 7  Bialystok, 15-082  POLAND |
|  |  |  |  |  |  |
| 1161 | Dr. Ines Pokrzywnicka-Gajek |  | Dr. Ewa Czernecka  Dr. Dorota Knychas  Dr. Andrzej Sawicki  Dr. Alina Walczak  Dr. Barbara Zalewska | Lecznica Specjalistow, Centrum Medyczne "Osteomed" NZOZ  Al. Krakowska 110/114  Warszawa, 02-256  POLAND  SYNEXUS SCM Sp. z o.o.  Oddzial w Warszawie  Leszno 12  Warszawa, 01-192  POLAND | Komisja Bioetyczna przy Okregowej Izbie Lekarskiej  ul. Swietojanska 7  Bialystok, 15-082  POLAND |
|  |  |  |  |  |  |
| 1349 | Dr. Jerzy Klimczak (Previous PI)  Dr. Joanna Badowska |  | Dr. Jerzy Klimczak  Dr. Malgorzata Klimczak  Dr. Anna Wlodarczak | Niepubliczny Specjalistyczny Zaklad Opieki Zdrowotnej Medicus  ul. Bielska 37  Cieszyn, 43-400  POLAND | Komisja Bioetyczna przy Okregowej Izbie Lekarskiej  ul. Swietojanska 7  Bialystok, 15-082  POLAND |
|  |  |  |  |  |  |
| 1429 | Prof. Pawel Hrycaj |  | Dr. Justyna Cal-Kocikowska  Dr. Katarzyna Cebrowska  Dr. Lidia Fornalska  Dr. Elzbieta Gigiel  Dr. Lukasz Hordecki  Dr. Kajetan Kisiel  Dr. Michal Moskal  Dr. Ewa Stachura  Dr. Dorota Zapolska-Pytlik | Szpital im. Teodora Dunina  Oddzial Reumatologiczny  Ul. Szpitalna 7  Koscian, 64-000  POLAND | Komisja Bioetyczna przy Okregowej Izbie Lekarskiej  ul. Swietojanska 7  Bialystok, 15-082  POLAND |
|  |  |  |  |  |  |
| 1445 | Slawomir Jeka |  | Dr. Jolanta Augustynowicz-Koziell  Dr. Radoslaw Brukiewa  Dr. Dominik Chraniuk  Dorota Wisniewska  Dr. Rafal Wojciechowski | NZOZ "NASZ LEKARZ"  Praktyka Grupowa Lekarzy Rodzinnych z Przychodnia Specjalistyczna  ul. Szczytna 20  Torun, 87-100  POLAND | Komisja Bioetyczna przy Okregowej Izbie Lekarskiej  ul. Swietojanska 7  Bialystok, 15-082  POLAND |
|  |  |  |  |  |  |
| 1446 | Anetta Pawlus (Previous PI)  Dr. Elzbieta Langer-Bieda |  | Dr. Przemyslaw Borowy  Ass. Prof. Edward Czerwinski  Dr. Jolanta Osieleniec | Krakowskie Centrum Medyczne NZOZ  ul. Kopernika 32  Krakow, 31-501  POLAND | Komisja Bioetyczna przy Okregowej Izbie Lekarskiej  ul. Swietojanska 7  Bialystok, 15-082  POLAND |
|  |  |  |  |  |  |
| 1508 | Dr. Maria Rell-Bakalarska |  | Dr. Janusz Jaworski  Dr. Ewa Klimczak  Dr. Robert Rupinski  Dr. Iwona Slowinska  Dr. Ewa Walewska  Dr. Malgorzata Wieteska  Agnieszka Zielinska | Rheuma Medicus - Specjalistyczne Centrum Reumatologii i Osteoporozy  Ul. Pruszkowska 6  Warszawa, 02-118  POLAND | Komisja Bioetyczna przy Okregowej Izbie Lekarskiej  ul. Swietojanska 7  Bialystok, 15-082  POLAND |
|  |  |  |  |  |  |

## Puerto Rico

**Coordinating Investigators:**

<None Entered>

| **Center** | **Principal Investigator** | **Co-Investigator(s)** | **Sub-Investigator(s)** | **Address(es)** | **Institutional Review Board or Ethics Committee Address(es)** |
| --- | --- | --- | --- | --- | --- |
|  |  |  |  |  |  |
| 1202 | Dr. Oscar Soto-Raices |  | Dr. Marelli Colon-Emeric | San Juan Arthritis & Research Center  El Mon te Mall Suite 2010  652 Avenue Munoz Rivera  San Juan, 00918  PUERTO RICO | Quorum Institutional Review Board  Suite 1000  1601 Fifth Avenue  Seattle, WA 98101  UNITED STATES  Quorum Review, Inc.  Suite 1000  1601 Fifth Avenue  Seattle, WA 98101  UNITED STATES |
|  |  |  |  |  |  |
| 1551 | Dr. Roberto Leon Perez |  | Dr. Elizabeth A. Barranco Santana | Edificio Parra  Oficina 505  2225 Ponce Bypass  Ponce, 00717  PUERTO RICO  Ponce School of Medicine  CAIMED Center  280 Monterrey Street  Ponce, 00716  PUERTO RICO | Quorum Review Institutional Review Board Incorporated  Suite 1000  1601 Fifth Avenue  Seattle, WA 98101  UNITED STATES |
|  |  |  |  |  |  |

## Romania

**Coordinating Investigators:**

<None Entered>

| **Center** | **Principal Investigator** | **Co-Investigator(s)** | **Sub-Investigator(s)** | **Address(es)** | **Institutional Review Board or Ethics Committee Address(es)** |
| --- | --- | --- | --- | --- | --- |
|  |  |  |  |  |  |
| 1154 | Prof. Dr. Rodica Marieta Chirieac |  | Dr. Codrina Irena Mihaela Ancuta  Dr Codruta Iulia Belibou  Dr. Iulia Georgiana Nita  Dr. Mihaela Simona Stoica | Spitalul de Recuperare Iasi  Str Pantelimon Halipa 14  Iasi, 700661  ROMANIA | Academia de Stiinte Medicale,Comisia Nationala de Etica pentru Studiul Clinic al Medicamentului  Str. Av. Sanatescu nr. 48,  Sector 1,  Bucuresti, 011478  ROMANIA |
|  |  |  |  |  |  |
| 1156 | Prof. Dr. Ruxandra Maria Ionescu |  | Dr. Andreea Ileana Borangiu  Dr Maria Laura Groseanu  Dr. Maria Laura Isac  Dr. Daniela Opris | Spitalul Clinic "Sf. Maria"  B-dul Ion Mihalache 37-39  Bucuresti, 011172  ROMANIA | Ministerul Sanatatii Comisia Nationala de Etica pentru Studiul Clinic al Medicamentului  Str Av Sanatescu nr 48  Bucuresti, sector 1 011478  ROMANIA |
|  |  |  |  |  |  |

## Russian Federation

**Coordinating Investigators:**

Polina Pukhtinskaya

| **Center** | **Principal Investigator** | **Co-Investigator(s)** | **Sub-Investigator(s)** | **Address(es)** | **Institutional Review Board or Ethics Committee Address(es)** |
| --- | --- | --- | --- | --- | --- |
|  |  |  |  |  |  |
| 1430 | Dr. Olga U. Stetsiouk |  | Dr. Irina V. Andreeva  Dr. Natalya Yu. Khozyainova  Tatyana V. Korolyova  Nadezhda V. Moskaleva | State Educational Institution of Higher professional education Smolensk State Medical Academy  Clinical Research Centre of diagnostic medicine and drugs  28 ulitsa Krupskoy  Smolensk, 214019  RUSSIAN FEDERATION | Independent EC of State Educational Institution of  High Professional Education  Smolensk State Medical Academy  28 ulitsa Krupskoy, 214019  27 prospect Gagarina  Smolensk, 214018  RUSSIAN FEDERATION  Independent Ethics Committee of State Educational Institution of High Professional Education  "Smolensk State Medical Academy of Federal Agency of Healthcare and Social Development"  28, ul. Krupskoj, 214019  27, pr. Gagarina  Smolensk, 214018  RUSSIAN FEDERATION  Independent Ethics Committee of State Educational Institution of High Professional Education  Smolensk State Medical Academy  28 ulitsa Krupskoy, 214019  27 prospect Gagarina  Smolensk, 214018  RUSSIAN FEDERATION |
|  |  |  |  |  |  |
| 1432 | Prof. Irina Mihailovna Marusenko |  | Dr. Yanina A. Avdeeva  Dr. Svetlana N. Kondrichina  Dr. Nina V. Koryakova  Dr. Irina I. Polskaya  Dr. Natalia N. Vezikova | GUZ Republican Hospital V.A.Baranov  3 ulitsa Pirogova  Petrozavodsk, 185019  RUSSIAN FEDERATION  Republican Hospital n. a. V.A.Baranov  Pirogova str., 3  Petrozavodsk, 185019  RUSSIAN FEDERATION | Ethics Committee at the Federal Service on Surveillance in Healthcare and Social Development  building 1, 4 Slavyanskaya ploshchad¿  Moscow, 109074  RUSSIAN FEDERATION  Ethics Committee at the Federal Service on Surveillance in Healthcare and Social Development  8, str. 2, Petrovskij bulvar  Moscow, 127051  RUSSIAN FEDERATION  Ethics Committee at the Republican Hospital n.a. V.A. Baranov  Pirogova str., 3  Petrozavodsk, 185019  RUSSIAN FEDERATION  Ethics Council at the Ministry of Healthcare of Russian Federation  3, Rakhmanovskij per.  Moscow, 127994  RUSSIAN FEDERATION |
|  |  |  |  |  |  |
| 1434 | Prof. Grigory P. Aroutyunov |  | Dr. Ekaterina Yu. Ilyina  Dr. Elena A. Kolesnikova  Dr. Karine A. Lytkina  Dr. Alexandr V. Rozanov | Russian State Medical University Moscow Faculty City Clinical Hospital #4  Department of Therapy  25 Pavlovskaya ulitsa  building 13  Moscow, 115093  RUSSIAN FEDERATION | Ethics Committee at the Federal Service on Surveillance in Healthcare and Social Development  building 1, 4 Slavyanskaya ploshchad¿  Moscow, 109074  RUSSIAN FEDERATION  Ethics Committee at the Federal Service on Surveillance in Healthcare and Social Development  8, str. 2, Petrovskij bulvar  Moscow, 127051  RUSSIAN FEDERATION  Ethics Council at the Ministry of Healthcare and Social Development of Russian Federation  3, Rakhmanovskij per.  Moscow, 127994  RUSSIAN FEDERATION  Local Ethics Committee at the City Clinical Hospital #4  building 13  25 Pavlovskaya ulitsa  Moscow, 115093  RUSSIAN FEDERATION |
|  |  |  |  |  |  |
| 1609 | Dr. Galina Usova |  | Dr. Elena Usova | Clinical Hospital #122 named after L.G. Sokolov of the Federal Medical-Biological Agency  4 Prospect Kultury  St. Petersburg, 194291  RUSSIAN FEDERATION | Ethics Committee at the Federal Service on Surveillance in Healthcare and Social Development  8, str. 2, Petrovskij bulvar  Moscow, 127051  RUSSIAN FEDERATION  Ethics Council at the Ministry of Healthcare of Russian Federation  3, Rakhmanovskij per.  Moscow, 127994  RUSSIAN FEDERATION  Local Ethics Committee at the L.G. Sokolov Clinical Hospital #122  4 Prospect Kultury  Saint-Petersburg, 194291  RUSSIAN FEDERATION |
|  |  |  |  |  |  |
| 1610 | Dr. Elena N. Ushakova |  | Veronika Finozhenok  Mariya Kosacheva  Anna Kozitsina  Dina Rogova  Alexander M. Shulman | State Health Institution City Hospital # 25, City Rheumatology Center of St. Petersburg  30 Bolshaya Podyatcheskaya Ulitsa  St. Petersburg, 190068  RUSSIAN FEDERATION | Ethics Committee at the Federal Service on Surveillance in Healthcare and Social Development  8, str. 2, Petrovskij bulvar  Moscow, 127051  RUSSIAN FEDERATION  Ethics Council at the Ministry of Healthcare of Russian Federation  3, Rakhmanovskij per.  Moscow, 127994  RUSSIAN FEDERATION  Local Ethics Committee at the State Health Institution City Hospital # 25  City Rheumatology Center of St. Petersburg  30 Bolshaya Podyatcheskaya Ulitsa  St. Petersburg, 190068  RUSSIAN FEDERATION |
|  |  |  |  |  |  |
| 1611 | Valeria Esip |  | Natalia N. Ivanova  Dr. Natalia Kirichek  Dr. Olga Shmanovskaya | St. Petersburg State Institution of Healthcare Consultative-diagnostic Center #85  89/3 Prospect Veteranov  St. Petersburg, 198260  RUSSIAN FEDERATION | Ethics Committee at the Federal Service on Surveillance in Healthcare and Social Development  8, str. 2, Petrovskij bulvar  Moscow, 127051  RUSSIAN FEDERATION  Ethics Council at the Ministry of Healthcare of Russian Federation  3, Rakhmanovskij per.  Moscow, 127994  RUSSIAN FEDERATION  Local Ethics Committee  at the St. Petersburg State Institution of Healthcare Consultative-diagnostic Center #85  89/3 Prospect Veteranov  St. Petersburg, 198260  RUSSIAN FEDERATION |
|  |  |  |  |  |  |
| 1612 | Dr. Andrey M. Sarana |  | Dr. Alina S. Agafina  Dr. Anna Anisenkova  Dr. Kamila Budagova | State Healthcare Institution City Hospital # 40 of Administrative Health Resort District  lit. B,9 Borisova ulitsa, Sestroretsk  St.Petersburg, 197706  RUSSIAN FEDERATION | Ethics Committee at the City Hospital # 40 Kurortnogo Administrativnogo Rajona  Borisova ulitsa, 9, lit. B, Sestroretsk  St.Petersburg, 197706  RUSSIAN FEDERATION  Ethics Committee at the Federal Service on Surveillance in Healthcare and Social Development  8, str. 2, Petrovskij bulvar  Moscow, 127051  RUSSIAN FEDERATION  Ethics Council at the Ministry of Healthcare of Russian Federation  3, Rakhmanovskij per.  Moscow, 127994  RUSSIAN FEDERATION |
|  |  |  |  |  |  |
| 1613 | Alexey L. Maslyanskiy |  | Marina Andrianova  Dr. Olga S. Bobina  Dr. Irina L. Dzhalalova  Dr. Irina L. Dzhalalova  Olga Kostikova  Dr. Natalia Kunitskaya | Almazov Federal Heart, Blood and Endocrinology Centre  2 Ulitsa Akkuratova  Saint-Petersburg, 197341  RUSSIAN FEDERATION | Ethics Committee at the Federal Service on Surveillance in Healthcare and Social Development  8, str. 2, Petrovskij bulvar  Moscow, 127051  RUSSIAN FEDERATION  Ethics Council at the Ministry of Healthcare of Russian Federation  3, Rakhmanovskij per.  Moscow, 127994  RUSSIAN FEDERATION  Local Ethics Committee at the Almazov Federal  15 Prospect Parkhomenko  Saint-Petersburg, 194156  RUSSIAN FEDERATION |
|  |  |  |  |  |  |
| 1646 | Dr. Andrey E. Dorokhov |  | Yulia Barinova  Dr. Valentina Chirkova  Dr. Irina L. Demidova  Natalia Dianova  Dr. Larisa Filippova  Dr. Elena Fogel  Dr. Daria Giganova  Dr. Elena S. Karpova  Marina Kateneva  Svetlana Novikova  Dr. Svetlana Tryankina  Dr. Natalia Zyablova | State Healthcare Institution Regional Clinical Hospital  1, Lyapidevskogo str.,  Barnaul, 656024  RUSSIAN FEDERATION | Local Ethics Committee of State Healthcare  Institution "Regional Clinical Hospital"  Lapidevskogo Street, 1  Barnaul, 656024  RUSSIAN FEDERATION |
|  |  |  |  |  |  |
| 1647 | Dr. Larisa V. Eliseeva |  | Dr. Evgenia Bukharova  Dr. Ksenia Gunderina  Dr. Natalia Kurkina  Dr. Olga Savelieva  Dr. Anna Shatuta  Dr. Olga Sibireva  Dr. Nadezhda V. Stroganova-Markova | Regional State Budgetary Healthcare Institution of Tomsk Regional Clinical Hospital  96,Ivana Chernykh str.  Tomsk, 634063  RUSSIAN FEDERATION | Ethics Council at the Ministry of Healthcare of Russian Federation  Department of State Regulation of Medicines Circulation  3, Rakhmanovskij per.  Moscow, 127994  RUSSIAN FEDERATION  Local Ethics Committee of Regional State Healthcare Institution of Tomsk Regional Clinical Hospital  96, Ivana Chernykh str.  Tomsk, 634063  RUSSIAN FEDERATION |
|  |  |  |  |  |  |
| 1648 | Prof. Tatiana A. Raskina |  | Dr. Julia V. Averkieva  Dr. Viacheslav Fanaskov  Dr. Marina Koroleva  Dr. Marina Letaeva  Dr. Olga S. Malyshenko  Oxana Pirogova  Dr. Svetlana A. Polyakova  Dr. Elena S. Shaf  Dr. Elena Shatina  Dr. Tatiana S. Sokolova | Kemerovo Regional Veterans Hospital  Rheumatology Regional center  50 Let Oktyabrya 10 Kemerovo  Kemerovo, 650099  RUSSIAN FEDERATION | Ethics Council at the Ministry of Healthcare of Russian Federation  Department of State Regulation of Medicines Circulation  3, Rakhmanovskij per.  Moscow, 127994  RUSSIAN FEDERATION  Local Ethics Committee of State Healthcare Institution Regional Clinical Hospital for War Veterans  50 let Oktaybrya str.  10, Kemerovo, 650099  RUSSIAN FEDERATION |
|  |  |  |  |  |  |
| 1649 | Dr. Elena Vladimirovna Zonova |  | Dr. Anna Akimova  Tatyana Dyakova  Anton Gutov  Dr. Dmitry Khabarov  Svetlana Lapsina  Dr. Tatyana Mikhailova  Dmitry Orlov  Nina Rakitina | "Consultative and Diagnostic Rheumatology Center Zdorovye Systavy", LLC  333 Romanova str  Novosibirsk, 630091  RUSSIAN FEDERATION | Ethics Council at the Ministry of Healthcare and Social Development of Russian Federation  Department of State Regulation of Medicines Circulation  3, Rakhmanovskij per.  Moscow, 127994  RUSSIAN FEDERATION  Local Ethics Committee of State Institution  Scientific Research Institute of Clinical and Experimental Lymphology of the Siberian Branch of RAMS  6, Arbuzova str.  Novosibirsk, 630117  RUSSIAN FEDERATION |
|  |  |  |  |  |  |
| 1650 | Dr. Nadezhda Vladimirovna Izmozherova |  | Dr. Maria I. Fominykh  Dr. Elena Gavrilova  Dr. Nina A. Getmanova  Dr. Olga Kolmakova  Dr. Olga O. Novoselova  Dr. Artem A. Popov  Dr. Elena A. Safianik  Dr. Natalia V. Tagiltseva | State Educational Institution of Higher Professional Education  Ural State Medical Academy of Federal Healthcare Agency and Social Development  General medicine department N 2 based on Municipal Institution Central City Clinical Hospital No 6  34, Seraphimy Deryabinoy str.  Ekaterinburg, 620149  RUSSIAN FEDERATION | Local Ethics Committee at Ural State Medical Academy  3 ulitsa Repina  Ekaterinburg, 620109  RUSSIAN FEDERATION |
|  |  |  |  |  |  |
| 1651 | Prof. Olga Mikhailovna Lesnyak |  | Dr. Tatiana Y. Bashkirtseva  Dr. Elena Kalinina  Dr. Luiza N. Kamkina  Dr. Natalia N. Krokhina  Dr. Anastasia M. Sukhanova  Dr. Varvara Tikhonova | State Healthcare Institution  Sverdlovsk Regional Clinical Hospital ¿ 1  185, Volgogradskaya str.  Ekaterinburg, 620102  RUSSIAN FEDERATION | Local Ethics Committee of State Healthcare  Institution "Sverdlovsk Regional Clinical Hospital No. 1"  185, Volgogradskaya str.  Ekaterinburg, 620102  RUSSIAN FEDERATION |
|  |  |  |  |  |  |
| 1663 | Dr. Evgeny Lvovich Nasonov (Previous PI)  Dr. Marina L. Stanislav |  | Anna Ilyina  Dr. Natalia G. Ionichenok  Dr. Yulia Korsakova  Dr. Elena Y. Panasyuk  Anna Pchelintseva  Dr. Alla P. Zhornyak | Rheumatology Research Institute of Russian Academy of Medical Sciences  34A Kashirskoe shosse  Moscow, 115522  RUSSIAN FEDERATION | Ethics Committee at Research Institute of Rheumatology  34 A Kashirskoe shosse  Moscow, 115522  RUSSIAN FEDERATION |
|  |  |  |  |  |  |

## Slovakia

**Coordinating Investigators:**

<None Entered>

| **Center** | **Principal Investigator** | **Co-Investigator(s)** | **Sub-Investigator(s)** | **Address(es)** | **Institutional Review Board or Ethics Committee Address(es)** |
| --- | --- | --- | --- | --- | --- |
|  |  |  |  |  |  |
| 1072 | Dr. Jozef Lukac |  | Dr. Olga Lukacova | Narodny ustav reumatickych chorob  ul.Ivana Krasku 4  Piestany, 921 01  SLOVAKIA | Eticka komisia pri Narodnom ustave reumatickych chorob  ul.I.Krasku 4  Piestany, 921 01  SLOVAKIA |
|  |  |  |  |  |  |
| 1073 * | Zelmira Macejova |  |  | Fakultna nemocnica L.Pasteura, I.interna klinika  trieda SNP 1  Kosice, 040 11  SLOVAKIA | Eticka komisia  Fakultna nemocnica L.Pasteura  Rastislavova 43  Kosice, 040 00  SLOVAKIA  Eticka komisia  pri Narodnom ustave reumatickych chorob  ul. I. Krasku 4  Piestany, 921 01  SLOVAKIA |
|  |  |  |  |  |  |
| 1075 | Dr. Pavol Polak |  | Frantisek Michalicka  Dr. Jan Remsik | NsP Zilina  Nestatna reumatologicka ambulancia  ul. Vojtecha Spanyola 43  Zilina, 012 07  SLOVAKIA | Eticka komisia pri Zilinskom samospravnom kraji  Zilinsky samospravny kraj, odbor zdravotnictva  Komenskeho ul.48  Zilina, 011 09  SLOVAKIA  Eticka komisia, Narodny ustav reumatickych chorob  Nabrezie I. Krasku 4  Piestany, 921 12  SLOVAKIA |
|  |  |  |  |  |  |
| 1076 * | Dr. Anna Sabova |  |  | Reumatologicka ambulancia  ul.M.R.Stefanika 187/177b  Vranov nad Toplou, 093 27  SLOVAKIA | Eticka komisia  Presovsky samospravny kraj, odbor zdravotnictva  nam.Mieru 2,  Presov, 080 01  SLOVAKIA  Eticka komisia  pri Narodnom ustave reumatickych chorob  ul. I. Krasku 4  Piestany, 921 01  SLOVAKIA |
|  |  |  |  |  |  |
| 1077 | Dr. Ivana Revayova |  |  | NOVAPHARM s.r.o -Zeleznicna NaP, Reumatologicka ambulancia  Sancova 110  Bratislava, 832 99  SLOVAKIA | Eticka komisia  pri Narodnom ustave reumatickych chorob  ul. I. Krasku 4  Piestany, 921 01  SLOVAKIA  Eticka komisia Novapharm, s.r.o.  Zeleznicna nemocnica a poliklinika  Sancova 110  Bratislava 3, 832 99  SLOVAKIA |
|  |  |  |  |  |  |
| 1338 | Dr. Peter Belica |  |  | Ecclesia, s.r.o Reumatologicka ambulancia  SNP 42/A  Nove Zamky, 94001  SLOVAKIA | Eticka komisia  Nitriansky samospravny kraj  Stefanikova tr.69  Nitra, 949 01  SLOVAKIA  Eticka komisia, Narodny ustav reumatickych chorob  Nabrezie I. Krasku 4  Piestany, 921 12  SLOVAKIA |
|  |  |  |  |  |  |
| 1339 | Dr. Agnesa Szolnokiova |  | Dr. Gizela Szolnokiova | AAGS, s.r.o. , nestatne zdravotnicke zariadenie,Dept. of Rheumatology  Reumatologicka ambulancia  Velkoblahovska 10  Dunajska Streda, 92901  SLOVAKIA | Eticka komisia Trnavskeho samospravneho kraja  P.O.BOX 128, Starohajska 10  Trnava, 917 01  SLOVAKIA  Eticka komisia, Narodny ustav reumatickych chorob  Nabrezie I. Krasku 4  Piestany, 921 12  SLOVAKIA |
|  |  |  |  |  |  |
| 1340 * | Dr. Maria Kolkusova |  | Dr. Lubomira Simova | Reumatologicka Ambulancia  Nemocnicna 986  Povazska Dystrica, 017 01  SLOVAKIA | Eticka komisia Tren¿ianskeho samosprávneho kraja  K Dolnej stanici 7282/20A  Tren¿ín, Slovakia 911 01  SLOVAKIA  Eticka komisia, Narodny ustav reumatickych chorob  Nabrezie I. Krasku 4  Piestany, 921 12  SLOVAKIA |
|  |  |  |  |  |  |
| 1341 | MUDr. Danica Telepkova |  |  | Interna a reumatologicka ambulancia  MUDr. Danica Telepkova, s.r.o.  Strojarenska 13  Kosice, 040 01  SLOVAKIA | Eticka komisia Kosickeho samospravneho kraja  Namestie Maratonu mieru 1  Kosice, 042 66  SLOVAKIA  Eticka komisia, Narodny ustav reumatickych chorob  Nabrezie I. Krasku 4  Piestany, 921 12  SLOVAKIA |
|  |  |  |  |  |  |
| 1342 | Dr. Zuzana Bubanova |  | Dr. Eva Kokavcova | Reumatologicka ambulancia, Poliklinika Ruzinov  Ruzinovska 6  Bratislava, 82606  SLOVAKIA | Eticka komisia  Urad samospravneho kraja  Sabinovska 16  P.O.Box 106  Bratislava, 820 05  SLOVAKIA  Eticka komisia, Narodny ustav reumatickych chorob  Nabrezie I.  Krasku 4  Piestany, 921 12  SLOVAKIA |
|  |  |  |  |  |  |
| 1343 | Dr. Zuzana Cizmarikova |  |  | Reumatologicka ambulancia, MUDr. Zuzana Cizmarikova, s.r.o.  Karpatska 3273/11  Poprad, 058 01  SLOVAKIA | Eticka komisia Presovskeho samospravneho kraja  Namestie mieru 2  Presov, 080 01  SLOVAKIA  Eticka komisia, Narodny ustav reumatickych chorob  Nabrezie I. Krasku 4  Piestany, 921 12  SLOVAKIA |
|  |  |  |  |  |  |
| 1344 | Dr. Viola Husarova |  |  | Reumatologicka ambulancia, REUMEX, s.r.o.  Zeleznicna 686/23  Rimavska Sobota, 979 01  SLOVAKIA | Eticka komisia, Narodny ustav reumatickych chorob  Nabrezie I. Krasku 4  Piestany, 921 12  SLOVAKIA  Nezavisla Eticka komisia Banskobystrickeho samospravneho kraja  Namestie SNP 23  Banska Bystrica, 974 01  SLOVAKIA |
|  |  |  |  |  |  |
| 1346 | Dr. Milan Krpciar |  |  | Reumatologicka ambulancia, Nestatne zdravotnicke zariadenie  Sotinska 1588  Senica, 905 01  SLOVAKIA | Eticka komisia Trnavskeho samospravneho kraja  P.O.BOX 128, Starohajska 10  Trnava, 917 01  SLOVAKIA  Eticka komisia, Narodny ustav reumatickych chorob  Nabrezie I. Krasku 4  Piestany, 921 12  SLOVAKIA |
|  |  |  |  |  |  |

## Spain

**Coordinating Investigators:**

<None Entered>

| **Center** | **Principal Investigator** | **Co-Investigator(s)** | **Sub-Investigator(s)** | **Address(es)** | **Institutional Review Board or Ethics Committee Address(es)** |
| --- | --- | --- | --- | --- | --- |
|  |  |  |  |  |  |
| 1035 | Dr. Jordi Carbonell Abello |  | Miriam Almirall Bernabe  Joan Calvet Fontova  Elisa Docampo Martinez  Ma Pilar Lisbona Perez  Juan Maymo | HOSPITAL DEL MAR  SERVICIO DE REUMATOLOGIA  PASSEIG MARITIM 25  BARCELONA, BARCELONA 08003  SPAIN | Comite Autonomico de Ensayos Clinicos de Andalucia  ETHICS COMMITTEE OF CLINIC INVESTIGATION  CONSEJERIA DE SALUD  AVDA. INNOVACION S/N  EDIFICIO ARENA 1  SEVILLA, SEVILLA 41020  SPAIN |
|  |  |  |  |  |  |
| 1036 | Juan Gomez-Reino Carnota |  | Dr. Juan Garcia Meijide  Myriam Liz Graña  Jose Ramon Maneiro Fernandez  Alejandra Rama Serans  Eva Salgado Perez | HOSPITAL CLINICO UNIVERSITARIO DE SANTIAGO  SERVICIO DE REUMATOLOGIA  Pº DE LA CHOUPANA S/N  SANTIAGO DE COMPOSTELA, A CORUÑA 15706  SPAIN | Comite Autonomico de Ensayos Clinicos de Andalucia  ETHICS COMMITTEE OF CLINIC INVESTIGATION  CONSEJERIA DE SALUD  AVDA. INNOVACION S/N  EDIFICIO ARENA 1  SEVILLA, SEVILLA 41020  SPAIN  Comite Etico de Investigacion Clinica de Galicia  SUBDIRECCION GENERAL DE FARMACIA Y PRODUCTOS SANITARIOS  CONSELLERIA DE SANIDADE  C/ SAN LAZARO, S/N  SANTIAGO DE COMPOSTELA, A CORUÑA 15703  SPAIN |
|  |  |  |  |  |  |
| 1038 | Dr. Emilio Martin Mola |  | Dr. Alejandro Balsa Criado  Miguel Bernad Pineda  Gema Bonilla Hernan  Sara Garcia Carazo  Leticia Lojo Oliveira  Maria Leticia Lojo-Oliveira  Dr. Carlos Perez De Ayala | Hospital Universitario De La Paz  Paseo de la Castellana, 261  UNIDAD DE CORONARIA, 1Âº PLANTA  Madrid, 28046  SPAIN | Comite Autonomico de Ensayos Clinicos de Andalucia  ETHICS COMMITTEE OF CLINIC INVESTIGATION  CONSEJERIA DE SALUD  AVDA. INNOVACION S/N  EDIFICIO ARENA 1  SEVILLA, SEVILLA 41020  SPAIN  Hospital Universitario La Paz  Secretaria Tecnica CEIC  Hospital General ¿ Planta 8ª  Paseo de la Castellana, 261  Madrid, 28046  SPAIN |
|  |  |  |  |  |  |
| 1039 | Juan Miguel Sanchez Burson |  | Natalia Cid Boza  Paz Gonzalez Moreno  Raquel Hernandez Sanchez | HOSPITAL EL TOMILLAR  CTRA. DOS HERMANAS-ALCALA  DOS HERMANAS, SEVILLA 41700  SPAIN  HOSPITAL NUESTRA SEÑORA DE VALME  SERVICIO DE REUMATOLOGIA  CTRA. CADIZ-BELLAVISTA KM. 548,9  SEVILLA, SEVILLA 41014  SPAIN | Comite Autonomico de Ensayos Clinicos de Andalucia  ETHICS COMMITTEE OF CLINIC INVESTIGATION  CONSEJERIA DE SALUD  AVDA. INNOVACION S/N  EDIFICIO ARENA 1  SEVILLA, SEVILLA 41020  SPAIN  Hospital Nuestra Señora de Valme  Comite Etico de Investigacion Clinica  Ctra de Cadiz-Bellavista, km. 548,9  Sevilla, 41014  SPAIN |
|  |  |  |  |  |  |
| 1292 | Dr. Francisco Javier Blanco Garcia |  | Dr. Elena Alonso Blanco Morales  Dr. Maria Del Carmen Bejerano Herreria  Jesus Carlos Fernandez Lopez  Mercedes Freire Gonzalez  Jose de Jesus Eduardo Gomez Bañuelos  Derikah Tatiana Gonzalez Perez  Ana Lois  Dr. Natividad Oreiro Villar | Complexo Hospitalario Universitario de A Coruna LABORATORIO DE INVESTIGACION  LABORATORIO DE INVESTIGACION EDIFICIO ANEXO AL HOSPITAL MATERNO INFANTIL  EDIFICIO ANEXO AL HOSPITAL MATERNO INFANTIL C/. XUBIAS DE ARRIBA, 84  LA CORUNA, 15006  SPAIN | Comite Autonomico de Ensayos Clinicos de Andalucia  ETHICS COMMITTEE OF CLINIC INVESTIGATION  CONSEJERIA DE SALUD  AVDA. INNOVACION S/N  EDIFICIO ARENA 1  SEVILLA, SEVILLA 41020  SPAIN  Comite Etico de Investigacion Clinica de Galicia  SUBDIRECCION GENERAL DE FARMACIA Y PRODUCTOS SANITARIOS  CONSELLERIA DE SANIDADE  C/ SAN LAZARO, S/N  SANTIAGO DE COMPOSTELA, A CORUÑA 15703  SPAIN |
|  |  |  |  |  |  |
| 1293 | Dr. Eduardo Ucar Angulo  Jose Miguel Aranburu Albizuri (Previous PI) |  | Olaia Begona Fernandez-Berrizbeitia  Maria Esther Ruiz Lucea | Hospital de Basurto  Servicio De Reumatologia  Avda. Montevideo, 18  Bilbao, Vizcaya 48013  SPAIN | Comite Autonomico de Ensayos Clinicos de Andalucia  ETHICS COMMITTEE OF CLINIC INVESTIGATION  CONSEJERIA DE SALUD  AVDA. INNOVACION S/N  EDIFICIO ARENA 1  SEVILLA, SEVILLA 41020  SPAIN  Comite Etico de Investigacion Clinica. Comunidad Autonoma del Pais Vasco (CEIC-E)  Direccion de Farmacia  C/ Donostia-San Sebastian, nº 1  Vitoria, Alava 01010  SPAIN  Hospital de Basurto  Comite Etico de Investigacion Clinica  Avda. Montevideo, 18  Bilbao, Vizcaya 48013  SPAIN |
|  |  |  |  |  |  |
| 1294 | Dr. Alberto Alonso Ruiz |  | Marcelo Calabozo Raluy  Fernando Perez Ruiz | Hospital Universitario Cruces  Servicio de Reumatologia  Plaza de Cruces s/n  BARAKALDO, VIZCAYA 48903  SPAIN | Comite Autonomico de Ensayos Clinicos de Andalucia  ETHICS COMMITTEE OF CLINIC INVESTIGATION  CONSEJERIA DE SALUD  AVDA. INNOVACION S/N  EDIFICIO ARENA 1  SEVILLA, SEVILLA 41020  SPAIN  Comite Etico de Investigacion Clinica. Comunidad Autonoma del Pais Vasco (CEIC-E)  Direccion de Farmacia  C/ Donostia-San Sebastian, nº 1  Vitoria, Alava 01010  SPAIN  Hospital de Cruces  Comite Etico de Investigacion Clinica  U. Epidemiologia Clinica. Pabellon de Investigacion (detras del Pabellon de Administracion)  Plaza de Cruces nº 12  Barakaldo, Vizcaya 48903  SPAIN |
|  |  |  |  |  |  |
| 1295 | Eugenio Chamizo Carmona |  | Juan Jose Aznar Sanchez  Adela Gallego Flores  Noemi Patricia Garrido Puñal | HOSPITAL DE MERIDA  CONSULTAS EXTERNAS 3ª PLANTA. SERVICIO DE REUMATOLOGIA  C/MIGUEL SERVET S/N  MERIDA, BADAJOZ 06800  SPAIN | Comite Autonomico de Ensayos Clinicos de Andalucia  ETHICS COMMITTEE OF CLINIC INVESTIGATION  CONSEJERIA DE SALUD  AVDA. INNOVACION S/N  EDIFICIO ARENA 1  SEVILLA, 41020  SPAIN  Hospital Infanta Cristina  Comite Etico de Investigacion Clinica de Badajoz  6ª Planta  Avda. de Elvas, s/n  Badajoz, Badajoz 06080  SPAIN |
|  |  |  |  |  |  |
| 1298 | Dr. Juan Garcia Meijide (Previous PI)  Manuel Pombo Suarez |  | Myriam Liz Graña  Fatima Nercellas Colmeiro | HOSPITAL NUESTRA SENORA DE LA ESPERANZA  AVENIDA DE LAS BURGAS 2  SANTIAGO DE COMPOSTELA, 15705  SPAIN | Comite Autonomico de Ensayos Clinicos de Andalucia  ETHICS COMMITTEE OF CLINIC INVESTIGATION  CONSEJERIA DE SALUD  AVDA. INNOVACION S/N  EDIFICIO ARENA 1  SEVILLA, 41020  SPAIN  Comite Etico de Investigacion Clinica de Galicia  SUBDIRECCION GENERAL DE FARMACIA Y PRODUCTOS SANITARIOS  CONSELLERIA DE SANIDADE  C/ SAN LAZARO, S/N  SANTIAGO DE COMPOSTELA, A CORUÑA 15703  SPAIN |
|  |  |  |  |  |  |
| 1299 | Dr. Antonio Fernandez Nebro |  | Maria Angeles Belmonte Lopez  Enrique Calero Secall  Virginia Coret Cagigal  Maria Victoria Irigoyen Oyarzabal  Francisco Gabriel Jimenez Nunez  Maria America Lopez Lasanta  Antonio Ponce Vargas  Manuel Rodriguez Perez  Inmaculada Urena Garnica | HOSPITAL CIVIL. HOSPITAL REGIONAL UNIVERSITARIO CARLOS HAYA  PABELLON 7, 2ª PLANTA, SERVICIO DE REUMATOLOGIA  PLAZA DEL HOSPITAL CIVIL S/N  MALAGA, MALAGA 29009  SPAIN | Comite Autonomico de Ensayos Clinicos de Andalucia  ETHICS COMMITTEE OF CLINIC INVESTIGATION  CONSEJERIA DE SALUD  AVDA. INNOVACION S/N  EDIFICIO ARENA 1  SEVILLA, 41020  SPAIN  Hospital Universitario Carlos Haya  Comite Etico de Investigacion Clinica  7ª Planta Pabellon A  Avda. Carlos Haya, s/n  Malaga, 29010  SPAIN |
|  |  |  |  |  |  |
| 1300 * | Alicia Garcia Lopez |  | Maria del Pilar Maiquez Asuero  Juan Bautista Povedano Gomez | HOSPITAL UNIVERSITARIO VIRGEN DEL ROCIO  CENTRO DE DIAGNOSTICO Y TRATAMIENTO. SERVICIO DE REUMATOLOGIA, 3ª PLANTA  AVDA. MANUEL SIUROT, S/N  SEVILLA, SEVILLA 41013  SPAIN | Comite Autonomico de Ensayos Clinicos de Andalucia  ETHICS COMMITTEE OF CLINIC INVESTIGATION  CONSEJERIA DE SALUD  AVDA. INNOVACION S/N  EDIFICIO ARENA 1  SEVILLA, SEVILLA 41020  SPAIN |
|  |  |  |  |  |  |
| 1301 | Isabel Mateo Bernardo |  | Alexia de Juanes Montmeterme  Javier Garcia Gonzalez  Rosa Maria Gonzalez Crespo  Beatriz Joven Ibañez | Hospital 12 de Octubre  Departamento de Reumatologia - Edificio nuevo de Consultas externas - Planta 5º  Avenida de Cordoba SN  Madrid, Madrid 28041  SPAIN | Comite Autonomico de Ensayos Clinicos de Andalucia  ETHICS COMMITTEE OF CLINIC INVESTIGATION  CONSEJERIA DE SALUD  AVDA. INNOVACION S/N  EDIFICIO ARENA 1  SEVILLA, 41020  SPAIN  NSTITUTO DE INVESTIGACION HOSPITAL 12 de Octubre  María Ugalde Díez  Area de Gestion de Proyectos - Unidad Administrativa CEIC  Centro de Actividades Ambulatorias, Bloque D - Planta 6ª  Avda. de Cordoba, s/n  Madrid, 28041  SPAIN |
|  |  |  |  |  |  |
| 1303 | Desamparados Ybanez Garcia |  | Maria Teresa Contreras Martinez  Begona Laiz Marro  Mª Luisa Muñoz Guillen  Rosa Negueroles Albuixech  Dr. Jose Luis Valero Sanz | Hospital Universitari i Politecnic La Fe de Valencia  Servicio de Reumatologia. Torre B. 6ª Planta.  Bulevar Sur, s/n  Valencia, Valencia 46026  SPAIN | Comite Autonomico de Ensayos Clinicos de Andalucia  ETHICS COMMITTEE OF CLINIC INVESTIGATION  CONSEJERIA DE SALUD  AVDA. INNOVACION S/N  EDIFICIO ARENA 1  SEVILLA, SEVILLA 41020  SPAIN |
|  |  |  |  |  |  |
| 1304 | Dr. Federico Navarro Sarabia |  | Dr. Paula Cejas Caceres  Maria Fernandez Alba  Maria Dolores Garcia Armario  Blanca Estela Hernandez Cruz  Manuel Maqueda Lopez  Virginia Moreira  Maria Victoria Navarro Compan  Dolores Ruiz Montesinos | Hospital Universitario Virgen Macarena  Servicio de Reumatologia  Avenida Dr Fedriani, 3, Semisotano  Sevilla, 41009  SPAIN | Comite Autonomico de Ensayos Clinicos de Andalucia  ETHICS COMMITTEE OF CLINIC INVESTIGATION  CONSEJERIA DE SALUD  AVDA. INNOVACION S/N  EDIFICIO ARENA 1  SEVILLA, 41020  SPAIN  Hospital Universitario Virgen de la Macarena  Comite Etico de Investigacion Clinica  Avda. Doctor Fedriani, 3  Sevilla, 41009  SPAIN |
|  |  |  |  |  |  |
| 1576 | Dr. Ricardo Blanco Alonso |  | Mario Agudo Bilbao  Ines Margarita Ayerbe Cintra  Dr. Alfonso Corrales Martinez  Dr. Miguel Angel Gonzalez-Gay Mantecon  Cristina Martinez Dubois  Dr. Victor Manuel Martinez Taboada  Maria Enriqueta Peiro Callizo  Dr. Jose Luis Peña Sagredo | Hospital Universitario Marques de Valdecilla (HUMV)  CONSULTAS DE REUMATOLOGIA - EDIFICIO DE CONSULTAS  VALDECILLA SUR  SEGUNDA PLANT Avenida Valdecilla No 25  SANTANDER, CANTABRIA 39008  SPAIN | Comite Autonomico de Ensayos Clinicos de Andalucia  ETHICS COMMITTEE OF CLINIC INVESTIGATION  CONSEJERIA DE SALUD  AVDA. INNOVACION S/N  EDIFICIO ARENA 1  SEVILLA, SEVILLA 41020  SPAIN  Hospital Marques de Valdecilla  Comite Etico de Investigacion Clinica de Cantabria  Fundacion Marques de Valdecilla, 5ª Planta. Escuela Universitaria de Enfermeria  Avda. de Valdecilla, s/n, Santander 39008  SPAIN |
|  |  |  |  |  |  |

## Sweden

**Coordinating Investigators:**

Thomas Mandl

| **Center** | **Principal Investigator** | **Co-Investigator(s)** | **Sub-Investigator(s)** | **Address(es)** | **Institutional Review Board or Ethics Committee Address(es)** |
| --- | --- | --- | --- | --- | --- |
|  |  |  |  |  |  |
| 1078 | Dr. Soren Transo |  | Dr. Britt-Mari Wigert | Lanssjukhuset Ryhov, Ortoped- och reumatologkliniken  Jonkoping, 551 85  SWEDEN  Sjukhusapoteket Jonkoping  Lanssjukhuset Ryhov  Jonkoping, 55185  SWEDEN | Regionala Etikprovningsnamnden i Linkoping  c/o Halsouniversitetets kansli, Linkopings Universitet  Linkoping, 581 83  SWEDEN |
|  |  |  |  |  |  |
| 1079 | Dr. Solbritt Rantapaa-Dahlqvist (Previous PI)  Dr. Stefan Engstrand |  | Dr. Gerd-Marie Alenius  Lena Innala | Norrlands Universitetssjukhus  Reumatologiska kliniken  Umea, 901 85  SWEDEN | Regionala Etikprovningsnamnden i Linkoping  c/o Halsouniversitetets kansli, Linkopings Universitet  Linkoping, 581 83  SWEDEN |
|  |  |  |  |  |  |
| 1313 | Ann Olofsson Sahlqvist (Previous PI)  Lilian Vasaitis |  | Johan Back  Eva Baecklund  Dan Henrohn | Akademiska University Hospital  Reumatologiska kliniken  Sjukhusvagen  Uppsala, 75185  SWEDEN | Regionala etikprovningsnamnden i Linkoping  c/o Halsouniversitetets kansli  Sandbacksgatan 7  Linkoping, 58183  SWEDEN |
|  |  |  |  |  |  |
| 1519 | Dr. Jorgen Lysholm |  | Tomas Husmark | FALU LASARETT MEDICINKLINIKEN  Falun, 791 82  SWEDEN | Regionala etikprovningsnamnden i Linkoping  C O HALSOUNIVERSITETETS KANSLI LINKOPINGS UNIVERSITET  Sandbacksgatan 7  Linkoping, 58183  SWEDEN |
|  |  |  |  |  |  |
| 1607 | John Svensson |  | Dr. Staffan Magnusson  Evangelia Roumpi | SUNDSVALLS SJUKHUS MEDICINKLINIKEN LASARETTSVÃ¿GEN 21  Sundsvall, 851 86  SWEDEN | Regionala etikprovningsnamnden i Linkoping  C/O Halsouniversitetets Kansli Linkopings Universitet  Sandbacksgatan 7  Linkoping, 58183  SWEDEN |
|  |  |  |  |  |  |
| 1608 | Elke Theander |  |  | Skanes Universitetssjukhus i Malmo  Reumamottagningen ing 25 plan 2  S Forstadsgatan 101  Malmo, 205 02  SWEDEN | Regionala etikprovningsnamnden i Linkoping  C/O Halsouniversitetets Kansli Linkopings Universitet  Sandbacksgatan 7  Linkoping, 58183  SWEDEN |
|  |  |  |  |  |  |

## Taiwan

**Coordinating Investigators:**

<None Entered>

| **Center** | **Principal Investigator** | **Co-Investigator(s)** | **Sub-Investigator(s)** | **Address(es)** | **Institutional Review Board or Ethics Committee Address(es)** |
| --- | --- | --- | --- | --- | --- |
|  |  |  |  |  |  |
| 1361 | Dr. Wen-Chan Tsai |  | Dr. Jia-Hua Ho  Dr. Tsan-Teng Ou  Dr. Chen-Ching Wu  Dr. Jeng-Hsien Yen | Kaohsiung Medical University,Chung-Ho Memorial Hospital  No. 100 Tzyou 1st Road  Kaohsiung, 807  TAIWAN | Kaohsiung Medical University Chung-Ho Memorial Hospital, Institutional Review Board  No. 100, Tzyou Ist Road  Kaohsiung, 807  TAIWAN |
|  |  |  |  |  |  |
| 1362 | Dr. Ping-Ning Hsu |  | Yun-Chen Toh | National Taiwan University Hospital  No.7  Chung-Shan South Road Taipei,  Taipei, 10018  TAIWAN | IRB Services  Suite 300  372 Hollandview Trail  Aurora, ON L4G 0A5  CANADA  Research Ethics Committee of National Taiwan University Hospital  No.7 Chung-Shan South Road  Taipei, 100  TAIWAN |
|  |  |  |  |  |  |
| 1363 | Hsiao-yi Lin |  | Yi-Chun Lin  Shu-Hung Wang | Taipei Veterans General Hospital  No. 201 Shih-Pai Road Section 2  Beitou District  Taipei, 11217  TAIWAN | Taipei Veterans General Hospital, The Institutional Review Board  No. 201  Sec. 2, Shih-Pai Road  Taipei, 112  TAIWAN |
|  |  |  |  |  |  |
| 1365 | Dr. Ming-Fei Liu |  | Dr. Meng-Yu Weng | National Cheng Kung University Hospital  No. 138, Sheng-Li Road  Tainan, 704  TAIWAN | Human Experiment and Ethics Committee National Cheng Kung University Hospital  138 Sheng Li Road  Tainan, 704  TAIWAN  National Cheng Kung University Hospital, Institutional Review Board  No. 138, Sheng-Li Road  Tainan, 704  TAIWAN |
|  |  |  |  |  |  |
| 1366 | Dr. Der-Yuan Chen  Dr. Joung-liang Lan (Previous PI) |  | Hsin-Hua Chen  Yi-Hsing Chen  Dr. Hsiu-Cheng Chou  Dr. Tsu-yi Hsieh  Dr. Chia-Wei Hsieh  Wen-Nan Huang  Chen Yi-Ming | Taichung Veterans General Hospital  No. 160, Section. 3, Taichung Port Road, Situn District  Taichung, 40705  TAIWAN | The Institutional Review Board of Taichung Veterans General Hospital  No. 160, Section. 3, Taichung Port Road., Situn District  Taichung City, 407  TAIWAN |
|  |  |  |  |  |  |
| 1514 | Dr. Shue-Fen Luo |  | Huei-Huang Ho  Miss Hsiu-Feng Hsia  Chang-Fu Kuo  Lieh-bang Liou | Chang Gung Medical Foundation-Linkou Branch  Department of Internal Medicine, Division of Allergy, Immunology and Rheumatology  5, Fu-Shin Street  Kwei-Shan, 333  TAIWAN | Chang Gung Medical Foundation, Institutional Review Board  No. 199 Tung Hwa North Road  Taipei, 10507  TAIWAN |
|  |  |  |  |  |  |
| 1515 | Dr. Tien-tsai Cheng |  | Ying-Chou Chen  Dr. Han-Ming Lai  Ya-Chi Ou  Shan-Fu Yu | Chang Gung Medical Foudation Kaohsiung Branch  No 123, Ta-Pei Road, Niao Sung Hsiang  Kaohsiung, 833  TAIWAN  Chang Gung Medical Foundation, Kaohsiung Branch  123 Ta-Pei Road  Niao-Sung Hsiang  Kaohsiung, 833  TAIWAN  Chang Gung Medical Foundation-Kaohsiung Branch, Department of Pharmacy  No.123, Dapi Rd., Niaosong Shiang  Kaohsiung County, 83301  TAIWAN  Chang Gung Medical Foundation-Kaohsiung Branch/Division of Allergy, Immunology and Rheumatology  123, Ta-Pei Road  Niao Sung Hsiang, Kaohsiung County 833  TAIWAN  Chang Gung Memorial Hospital Kaohsiung branch  Division of Allergy, Immunology and Rheumatology  No. 123, Ta-Pei Road  Niao Sung Hsiang, Kaohsiung County 833  TAIWAN | Chang Gung Medical Foundation, Institutional Review Board  No. 199 Tung Hwa North Road  Taipei, 10507  TAIWAN |
|  |  |  |  |  |  |
| 1517 | Dr. Che-Chun Su |  | Ms. Jenny Chiang  Dr. Ying-Ming Chiu | Changhua Christian Hospital  135 Nanhsiao Street  Changhua, Changhua 500  TAIWAN | Changhua Christian Hospital, Institutional Review Board  No. 135, Nanhsiao Street  Changhua, 500  TAIWAN |
|  |  |  |  |  |  |
| 1518 | Dr. Jui-Cheng Tseng |  | Ling-Jung Yen | Kaohsiung Veterans General Hospital  386 Ta-Chung First Road  Kaohsiung, 813  TAIWAN | Kaohsiung Veterans General Hospital, Institutional Review Board  386 Ta-Chung First Road  Kaohsiung, 813  TAIWAN |
|  |  |  |  |  |  |

## Thailand

**Coordinating Investigators:**

<None Entered>

| **Center** | **Principal Investigator** | **Co-Investigator(s)** | **Sub-Investigator(s)** | **Address(es)** | **Institutional Review Board or Ethics Committee Address(es)** |
| --- | --- | --- | --- | --- | --- |
|  |  |  |  |  |  |
| 1453 | Prof. Worawit Louthrenoo |  | Assist. Prof. Nuntana Kasitanon  Suparaporn Wangkaew | Division of Rheumatology, Department of Internal Medicine, Faculty of Medicine,  Chiang Mai University, 110 Intavaroros Road,  Amphoe Mueang, Chiang Mai 50200  THAILAND | Research Ethics Committee 1  Research Ethics Committee 1, Faculty of Medicine, Chiang Mai University  110 Intavaroros Road  Amphoe Muang, Chiang Mai 50200  THAILAND |
|  |  |  |  |  |  |
| 1454 | Dr. Sungchai Angthararak |  | Kittiwan Choojitarom MD  Dr. Tasanee Kitumnuaypong  Dr. Neerawan Krajaechan | Rheumatology Unit, Department of Internal Medicine, Rajavithi Hospital  2 Rajavithi Road  Bangkok, Thailand 10400  THAILAND | Ethics Committees on Researches Involving Human Subjects, Rajavithi Hospital  2 Rajavithi Road  Bangkok, 10400  THAILAND  Ministry of Public Health  Public Health Academic Office  Tiwanon Road  Amphoe Muang  Nonthaburi, 11000  THAILAND |
|  |  |  |  |  |  |
| 1455 | Assoc.Prof Siraphop Suwannaroj |  | Assist.Prof Chingching Foocharoen  Assist.Prof. Ajanee Mahakkanukrauh  Prof. Ratanavadee Nanagara | Allergy Immunology Rheumatology Division,Department of Medicine, Faculty of Medicine  Khon Kaen University,  123 Mirtapap Road, Amphur Muang,  Khon Kaen, Thailand 40002  THAILAND | The Khon Kaen University Ethics Committee for Human Research  Faculty of Medicine, Khon Kaen University  Dean Office 6 floor  123 Mitraphap Road  Khon Kaen, 40200  THAILAND |
|  |  |  |  |  |  |
| 1456 | Dr. Paijit Asavatanabodee |  | Sumapa Chaiamnuay  Chokchai Kittiyanpanya  Pongthorn Narongroeknawin  Suphawan Phukongchai  Tarinee Rojsakulkit | Rheumatology Unit, Department of Internal Medicine, Phramongkutklao Hospital  315 Rajavithi Rd, Rajathevee  Phayathai, Bangkok 10400  THAILAND | Institutional Review Board Royal Thai Army Medical Department  317 Rajavithi Road  Rajathevee  Bangkok, 10400  THAILAND |
|  |  |  |  |  |  |
| 1624 | Dr. Wanruchada Katchamart |  | Dr. Praveena Chiowchanwisawakit  Dr. Somboon Intalapaporn  Dr. Chayawee Muangchan | Siriraj Hospital, Mahidol University  Division of Rheumatology, Department of Medicine, Faculty of Medicine  2 Prannok Road  Bangkoknoi  Bangkok, 10700  THAILAND | Siriraj Institutional Review Board  2 Prannok Road  Bangkoknoi  Bangkok, Bangkok 10700  THAILAND |
|  |  |  |  |  |  |

## Turkey

**Coordinating Investigators:**

<None Entered>

| **Center** | **Principal Investigator** | **Co-Investigator(s)** | **Sub-Investigator(s)** | **Address(es)** | **Institutional Review Board or Ethics Committee Address(es)** |
| --- | --- | --- | --- | --- | --- |
|  |  |  |  |  |  |
| 1052 | Prof. Dr. Nurullah Akkoc |  | Assoc. Prof. Dr. Servet Akar  Assoc. Prof. Dr. Ahmet Merih Birlik  Prof. Dr. Sedef Gidener | Dokuz Eylul University Medical Faculty Internal Diseases Department Rheumatology Division  Inciralti  Izmir, 35340  TURKEY | CEIC Parc de Salut Mar  Institut de Recerca Hospital del Mar  C/ Dr. Aiguader 88  Barcelona, 08003  SPAIN  Dokuz Eylul Universitesi Tip Fakultesi Klinik Arastirmalar Etik Kurulu  Dokuz Eylul Universitesi Dekanlik Binasi  Kat 1  Izmir, Inciralti 35340  TURKEY  Ilac Klinik Arastirmalar Etik Danisma Kurulu  Sogutozu Mahallesi 2176. Sokak No:5  Cankaya, Ankara 06520  TURKEY  Izmir No.3 Clinical Research Ethics Committee  Dokuz Eylul University Medical Faculty  Inciralti  Izmir, 35340  TURKEY |
|  |  |  |  |  |  |
| 1053 | Prof. Dr. Gokhan Keser |  | Assoc. Prof. Dr. Kenan Aksu  Prof. Dr. Isik Tuglular | Ege University Medical Faculty Internal Diseases Department Rheumatology Division  Bornova  Izmir, 35100  TURKEY |  |
|  |  |  |  |  |  |
| 1054 | Prof. Dr. Sedat Kiraz |  | Assoc. Prof. Dr. Sule Apras Bilgen  Prof. Dr. Alper Iskit  Spec. Dr. Omer Karadag | Hacettepe University Faculty of Medicine  Department of Rheumotology  Sihhiye  Ankara, 06100  TURKEY  Hacettepe University Medical Faculty Internal Diseases Department Rheumatology Division  Sihhiye  Ankara, 06100  TURKEY |  |
|  |  |  |  |  |  |

## Ukraine

**Coordinating Investigators:**

<None Entered>

| **Center** | **Principal Investigator** | **Co-Investigator(s)** | **Sub-Investigator(s)** | **Address(es)** | **Institutional Review Board or Ethics Committee Address(es)** |
| --- | --- | --- | --- | --- | --- |
|  |  |  |  |  |  |
| 1163 | Vira Iosypivna Tseluyko |  | Dr. Ol'ha Victorivna Radchenko  Dr. Viktoriya Victorivna Yarosh | Municipal Establishment of Health Care  "Kharkiv City Clinical Hospital #8", Dept. of Rheumatology  Kharkiv Medical Academy of Postgraduate Education  Chair of Cardiology & Functional Diagnostic  266g, Saltivske shosse  Kharkiv, 61178  UKRAINE | Committee for Ethics Issues of of ME of Health Care "Kharkiv City Clinical Hospital #8"  266g, Saltivske Shosse  Kharkiv, 61178  UKRAINE |
|  |  |  |  |  |  |
| 1164 | Prof. Vadym A. Vizir |  | Dr. Olexandr E. Berezyn  Dr. Igor V. Zaika | City Hospital #7, Department of Internal Diseases #2 of Zaporizhzhia State Medical University  9 Lunacharskogo Street  Zaporizhzhia, 69118  UKRAINE | Central Committee for Ethics Issues of Ministry of Health Care of Ukraine  5, Narodnogo opolchennya Str.  Kyiv, 03680  UKRAINE  Committee for Ethics Issues of Zaporizhzhia Clinical Hospital #7  9 Lunacharskogo Street  Zaporizhzhia, 69118  UKRAINE |
|  |  |  |  |  |  |
| 1165 | Prof. Oleg N. Nadashkevich |  | Dr. Halyna M. Hrytsenko  Oksana Z. Stefyuk | Municipal City Clinical Hospital #4, Policlinic Care, Family Medicine and Dermatology  Venereology Department of Lviv National Medical University n.a. Danylo Galytskyy.  3 Sventsitskogo Street  Lviv, 79011  UKRAINE | Central Committee for Ethics Issues of Ministry of Health Care of Ukraine  5, Narodnogo opolchennya Street  Kyiv, 03680  UKRAINE  Committee for Ethics Issues of Municipal City Clinical Hospital #4  3 Sventsitskogo Str  Lviv, 79011  UKRAINE |
|  |  |  |  |  |  |
| 1166 | Prof. Mykola A. Stanislavchuk |  | Dr. Nabil Sh. Ali  Dr. Inna I. Andrushko  Dr. Olena Oleksandrivna Savytska | Vinnitsa Regional Clinical Hospital n.a. Pirogov  Department of Internal Medicine #1 of Vinnitsa National Medical University n.a. Pirogov  46 Pirogova Street  Vinnitsa, 21018  UKRAINE  Vinnitsa Regional Clinical Hospital n.a. Pirogov  Department of Faculty Therapy of Vinnitsa National Medical University n.a. Pirogov  46 Pirogova Street  Vinnitsa, 21018  UKRAINE | Committee for Ethics Issues of Vinnitsa Regional Clinical  Hospital n.a. Pirogov  46 Pirogova Street  Vinnitsa, 21018  UKRAINE |
|  |  |  |  |  |  |
| 1167 | Prof. Vladyslav V. Povoroznyuk |  | Dr. Halyna S. Dubetska  Dr. Nataliia I. Dzerovych  Dr. Nataliia V. Grygorieva  Dr. Tetyana A. Karasevska  Dr. Tetyana V. Orlyk | State Establishment «Institute of Gerontology of NAMS of Ukraine n.a. D.F. Chebotareva»,  Department of Clinical Physiology and Pathology of Musculoskeletal System  67 Vyshgorodska Street  Kyiv, 04114  UKRAINE | Committee for Ethics Issues Of Clinical trials of Institute of Gerontology of AMS of Ukraine  67, Vyshgorodska Street  Kyiv, 04114  UKRAINE |
|  |  |  |  |  |  |
| 1179 | Prof. Anatoliy S. Svintsitskyy |  | Dr. Nataliia I. Khomchenkova  Dr. Nataliya P. Kozak | State Institution Republican Clinical Hospital of the Ministry of Health of Ukraine  Department of Internal Medicine #3 of National Medical University n.a. O.O. Bogomolets  9 Yuriya Kotsyubynskogo Street  Kyiv, 04053  UKRAINE | Committee for Ethics Issues of SI "Republican Clinical Hospital of the MoH of Ukraine¿  9 Yuriya Kotsyubynskogo Street  Kyiv, 04053  UKRAINE |
|  |  |  |  |  |  |
| 1522 | Prof. Andriy V. Petrov |  | Dr. Ganna A. Alekseeva  Volodymyr O. Biloglazov  Dr. Galyna M. Koshukova | CRI "Clinical Territorial Medical Association "University Clinic", Department of Rheumatology  SI "Crimean State Medical University n.a. S.I.  Georgievskiy", Department of Internal Medicine#2  69 Kyivska Street  Simferopol, Crimea 95017  UKRAINE | Committee for Ethics Issues of Crimean Republican Institution "Clinical Hospital n.a. M.O. Semashko"  69, Kyivska Street  Simferopol, Crimea 95017  UKRAINE |
|  |  |  |  |  |  |
| 1662 | Dr. Victoriia V. Vasylets |  | Dr. Natalia V. Krasiluk  Dr. Natalya S. Tikhonchuk | Municipal Establishment City Clinical Hospital #9 n.a. O.I. Minakov, Department of Rheumatology  9 Pastera Street  Odesa, 65026  UKRAINE | Committee for Ethics Issues of Municipal Establishment City Clinical Hospital #9 n.a. O.I. Minakov  3 Valikhovskyi Lane  Odesa, 65082  UKRAINE |
|  |  |  |  |  |  |

## United Kingdom

**Coordinating Investigators:**

<None Entered>

| **Center** | **Principal Investigator** | **Co-Investigator(s)** | **Sub-Investigator(s)** | **Address(es)** | **Institutional Review Board or Ethics Committee Address(es)** |
| --- | --- | --- | --- | --- | --- |
|  |  |  |  |  |  |
| 1369 | Prof. John D. Isaacs |  | Dr. Gillian M. Bell  Dorothy Carmen  Dr. Wan Fai Ng  Dr. Martin Rynne  Linda Smith  Dr. Evin Sowden  John K. Wilson | Royal Victoria Infirmary  Clinical Research Facility  6th Floor, Leazes Wing  Newcastle Upon Tyne, NE1 4LP  UNITED KINGDOM | West midlands Research Ethics Commitee - Edgbaston  West Midlands - Edgbaston  The Old Chapel  Royal Standard Place  Nottingham, NG1 6FS  UNITED KINGDOM |
|  |  |  |  |  |  |
| 1371 | Dr. Emmanuel George |  | Dr. Priyanka Chandratre  Peter Chapman  Dr. Yee Ho Chiu  Sarah Gibson  Nicola Jeffries  Deepti Kapur  Madhu Mahindrakar  Dr. Pippa McCaffrey  Dr. Sangita Sathyamurthy  Dr. Vipin Tayal | Arrowe Park Hopsital  Department of Rheumatology  Arrowe Park Road  Wirral, Merseyside CH49 5PE  UNITED KINGDOM | West midlands Research Ethics Commitee - Edgbaston  West Midlands - Edgbaston  The Old Chapel  Royal Standard Place  Nottingham, NG1 6FS  UNITED KINGDOM |
|  |  |  |  |  |  |
| 1374 | Dr. Karen May Jane Douglas  Dr. Karen May Jane Douglas (Previous PI)  Dr. Theodoros Dimitroulas (Previous PI) |  | Dr. Theodoros Dimitroulas  Dr. George Hirsch  Prof. George D. Kitas  Stephanie Mole  Shirley Ann O¿Hare  Dr. Tracey Toms  Elizabeth Wells | The Dudley Group of Hospitals (DGOH) NHS Foundation Trust - Russells Hall Hospital  Department of Rheumatology  Esk House  Dudley, DY1 2HQ  UNITED KINGDOM | West midlands Research Ethics Commitee - Edgbaston  West Midlands - Edgbaston  The Old Chapel  Royal Standard Place  Nottingham, NG1 6FS  UNITED KINGDOM |
|  |  |  |  |  |  |
| 1375 | Dr. Thomas P. Sheeran |  | Dr. Abdul Baker  Dr. Sangeetha Basker  Deborah Lloyd  Dr. Feyal Malik  Jacqueline McPeake  Dr. Diarmuid Mulherin  Dr. Thomas Price  Samantha Roskell  Dr. Sofia Tosounidou  Dr Srivinasan Venkatachalam | Cannock Chase Hospital/Mid Staffordshire General Hospitals NHS Trust  Brunswick Road  Cannock, Mid Staffordshire WS11 2XY  UNITED KINGDOM | West midlands Research Ethics Commitee - Edgbaston  West Midlands - Edgbaston  The Old Chapel  Royal Standard Place  Nottingham, NG1 6FS  UNITED KINGDOM |
|  |  |  |  |  |  |

## United States

**Coordinating Investigators:**

<None Entered>

| **Center** | **Principal Investigator** | **Co-Investigator(s)** | **Sub-Investigator(s)** | **Address(es)** | **Institutional Review Board or Ethics Committee Address(es)** |
| --- | --- | --- | --- | --- | --- |
|  |  |  |  |  |  |
| 1002 | Dr. Sanford Mayer Wolfe |  |  | STAT Research, Inc.  Suite 230  West Medical Plaza  One Elizabeth Place  Dayton, OH 45417  UNITED STATES  The Office of Dr. Sanford M. Wolfe, D.O. - STAT Research, Inc.  West Medical Plaza - Suite 230  One Elizabeth Place  Dayton, OH 45417  UNITED STATES | Quorum Institutional Review Board  Suite 1000  1601 Fifth Avenue  Seattle, WA 98101  UNITED STATES |
|  |  |  |  |  |  |
| 1003 | Dr. Melody D. St. John |  | Laura J. Larrison  Dr. James W. Logan | Mercy Clinical Hot Springs Communities  100 McGowan Court  Hot Springs, AR 71913  UNITED STATES | Quorum Review, Inc  Suite 800  1501 Fourth Avenue  Seattle, WA 98101  UNITED STATES |
|  |  |  |  |  |  |
| 1004 | Dr. Jeffrey Edward Poiley |  |  | The Office of Jeffrey E. Poiley, MD  324 East Par Avenue  Orlando, FL 32804  UNITED STATES | Quorum Institutional Review Board  Suite 1000  1601 Fifth Avenue  Seattle, WA 98101  UNITED STATES  Quorum Review, Inc.  Suite 1000  1601 Fifth Avenue  Seattle, WA 98101  UNITED STATES |
|  |  |  |  |  |  |
| 1005 | Dr. Stephen Allan Bookbinder |  | Christy M. Anstead  Arthur Elkins | Ocala Rheumatology Research Center  Suite 102  3210 Southwest 33rd Road  Ocala, FL 34474  UNITED STATES | Quorum Review IRB  Suite 1000  1601 Fifth Avenue  Seattle, WA 98101  UNITED STATES  Quorum Review, Inc.  Suite 1000  1601 Fifth Avenue  Seattle, WA 98101  UNITED STATES |
|  |  |  |  |  |  |
| 1006 | Dr. Stanley Bruce Cohen |  | Dr. Jean Ann Clark  Roy Mitchell Fleischmann  Thomas David Geppert  Mohammed Imran Iqbal  Robert Neil Jenkins  Dr. Talat Jehan Kheshgi  Zoran Kurepa  Dr. Sharad Lakhanpal  Dr. Andrea Suzanne Martin  Dr. Catalina Orozco  Dr. Richard Leo Stern  Dayna S. Swan-Flanders  Dr. Jack Bernstein Vine  Andrea S. Wheeler | Metroplex Clinical Research Center  8144 Walnut Hill Lane  Suite 810  Dallas, TX 75231  UNITED STATES  Radiant Research-Dallas  Suite 441  5939 Harry Hines Blvd.  Dallas, TX 75235  UNITED STATES | Quorum Review, Inc.  Suite 1000  1601 Fifth Avenue  Seattle, WA 98101  UNITED STATES |
|  |  |  |  |  |  |
| 1008 | Dr. Jaime Alberto Pachon |  | Ms Adriana Acosta  Dr. Jorge Tomas Caso  Dr. Margarita Rosa Garces  Dr. Olga Kromo  Isabel Mercedes Pino  Dr. Howard I. Schwartz  Dr. Eric Andrew Sheldon | Arthritis & Rheumatic Care Center  Suite 501  6141 Sunset Drive  Miami, FL 33143  UNITED STATES  Miami Research Associates  Suite 301  6141 Sunset Drive  Miami, FL 33143  UNITED STATES | Quorum Review IRB  Suite 1000  1601 Fifth Avenue  Seattle, WA 98101  UNITED STATES |
|  |  |  |  |  |  |
| 1009 | Dr. Mark William Niemer |  | George J. Casey  Dr. Steven M. Rock | Medical Associates Clinic, PC  1500 Associates Drive  Dubuque, IA 52002  UNITED STATES | Quorum Review, Inc.  Suite 1000  1601 Fifth Avenue  Seattle, WA 98101  UNITED STATES |
|  |  |  |  |  |  |
| 1010 | Dr. Joel Charles Silverfield |  | Dr. Michael Claude Burnette  Dr. Laura McIlwain Cruse  Dr. Bernard F. Germain  Dr. Harris Hugh McIlwain  Dr. Kimberly McIlwain Smith  Brenda L. Ware | Bernard Germain, MD  13801 Bruce B Downs Blvd,  Tampa, FL 33613  UNITED STATES  Burnette & Silverfield, MDS, PLC  Suite 303  4700 North Habana Avenue  Tampa, FL 33614  UNITED STATES  Tampa Medical Group, P.A.  Suite 406  13801 North Bruce B. Downs Boulevard  Tampa, FL 33613  UNITED STATES | Quorum Review Inc.  Suite 1000  1601 5th Avenue  Seattle, WA 98101  UNITED STATES  Quorum Review IRB Incorporated  Suite 1000  1601 Fifth Avenue  Seattle, WA 98101  UNITED STATES |
|  |  |  |  |  |  |
| 1011 | Dr. Simpson Bobo Tanner IV |  | Cindy Kate Anderson  Dr. John Michael Fahrenholz  Nanalane P. Frey | Vanderbilt University Medical Center  Suite 120  2611 West End Avenue  Nashville, TN 37203  UNITED STATES | Vanderbilt Institutional Review Board  1313 21st Avenue South  504 Oxford House  Nashville, TN 37232  UNITED STATES |
|  |  |  |  |  |  |
| 1012 | Dr. Frederick Dietz |  | Dr. Robin Renee Hovis  Kathryn M. Lemonds-Johnson  Dr. Frank A. Ventimiglia | Rockford Health Physicians  2300 North Rockton Avenue  Rockford, IL 61103-3692  UNITED STATES | Quorum Review, Inc.  Suite 1000  1601 Fifth Avenue  Seattle, WA 98101  UNITED STATES |
|  |  |  |  |  |  |
| 1013 | Dr. Edward Joel Fudman |  | Dr. Stephanie Ann Booth  Dr. Brian Sam Sayers | Austin Rheumatology Research  Suite 702  1301 West 38th Street  Austin, TX 78705  UNITED STATES  Austin Rheumatology Research  Suite 110  1301 West 38th Street  Austin, TX 78705  UNITED STATES | Quorum Review, Inc.  Suite 800  1501 Fourth Avenue  Seattle, WA 98101  UNITED STATES |
|  |  |  |  |  |  |
| 1014 * | Dr. Ami Charise Milton |  | Tricia A. Eveleigh  Deborah L. Schu  Jennifer A. Sohl | Internist Associates of Central New York  Suite 200  739 Irving Avenue  Syracuse, NY 13210  UNITED STATES | Quorum Review, Inc.  Suite 1000  1601 Fifth Avenue  Seattle, WA 98101  UNITED STATES |
|  |  |  |  |  |  |
| 1016 | Dr. Antony C. Hou |  | Patricia E. DesLauriers  Tina J. Escobedo  Dr. Eric Cheng-Jer Lee  Dr. Mohamed Bassam Sebai | Inland Rheumatology Clinical Trials  Suite 306 548 North 13th Ave  Upland, CA 91786  UNITED STATES  Inland Rheumatology Clinical Trials, Inc.  1238 East Arrow Highway  Upland, CA 91786  UNITED STATES | Quorum Review, Inc.  Suite 1000  1601 Fifth Avenue  Seattle, WA 98101  UNITED STATES |
|  |  |  |  |  |  |
| 1017 | Dr. Robert Emil Ettlinger |  | Dr. George Howard Krick  Neil F. Moody Jr.  Teresa A. Unkrur | Tacoma Center for Arthritis Research, PS  Suite 204  1901 South Cedar Street  Tacoma, WA 98405  UNITED STATES  Tacoma Center of Arthritis Research, PS  Suite 108  1901 South Cedar  Tacoma, WA 98405  UNITED STATES  The Office of Robert E. Ettlinger, MD  Tacoma Center for Arthritis Research, PS  1901 South Cedar, #108  Tacoma, WA 98405  UNITED STATES | Quorum Review, Inc.  Suite 1000  1601 Fifth Avenue  Seattle, WA 98101  UNITED STATES |
|  |  |  |  |  |  |
| 1018 | Dr. Joel Marc Kremer |  | Christine J. Barr  Dr. Ludovico Frank Cavaliere  Justine S. Feder-Lailer  Haley Garrett  Dr. Neal Steven Greenstein  Dr. Dorota L. Hausner-Sypek  Jessica L. Johnson  Mari V. Kaymakcian  Justine V. Kehn  Kathleen A. Kessler  Iris B. Klein  Dr. Victoria M. Michaels  Teresa M. Michaels  Rhonda L. Murphy  Dr. Norman Reid Romanoff  Dr. Harbrinder S. Sandhu  Jennifer Schreiner  Dr. Lee Schulman Shapiro  Nicole L. Shultes  Dr. Aixa E. Toledo-Garcia | The Center for Rheumatology, LLP  Suite 101  1367 Washington Avenue  Albany, NY 12206  UNITED STATES | Quorum Review IRB  Suite 1000  1601 Fifth Avenue  Seattle, WA 98101  UNITED STATES  Quorum Review, Inc.  Suite 1000  1601 Fifth Avenue  Seattle, WA 98101  UNITED STATES |
|  |  |  |  |  |  |
| 1020 | Dr. John Joseph Cush |  | Dr. Kathryn H. Dao  Leilani D. Law | Baylor Research Institute  9900 North Central Expressway  Arthritis Care and Research Center  Suite 550  Dallas, TX 75231  UNITED STATES  Presbyterian Hospital of Dallas, Arthritis Consultation Center  8200 Walnut Hill Lane  Dallas, TX 75231-4496  UNITED STATES | Baylor Research Institute Institutional Review Board  White/Blue/Red  Suite 501  3310 Live Oak  Dallas, TX 75204  UNITED STATES |
|  |  |  |  |  |  |
| 1024 | Dr. Jeffrey Louis Kaine |  | Dr. Yoel Drucker  Jill R. Garrett | Lovelace Scientific Resources  Suite C  411 Commercial Court  Venice, FL 34292  UNITED STATES  Lovelace Scientific Resources, Inc.  Suite 560  5741 Bee Ridge Road  Sarasota, FL 34233  UNITED STATES  Sarasota Arthritis Research Center  Suite 101  1945 Versailles Street  Sarasota, FL 34239  UNITED STATES  Venice Arthritis Center  Suite D  411 Commercial Court  Venice, FL 34292  UNITED STATES | Quorum Review, Inc.  Suite 1000  1601 Fifth Avenue  Seattle, WA 98101  UNITED STATES |
|  |  |  |  |  |  |
| 1026 | Dr. Richard Roy Olson |  | Dr. David James Dansdill  Tami M. Kucia  Dr. Matthew L. Mundwiler | Rockford Orthopedic Associates  5875 East Riverside Blvd  Rockford, IL 61114  UNITED STATES | Quorum Institutional Review Board  Suite 1000  1601 Fifth Avenue  Seattle, WA 98101  UNITED STATES  Quorum Review, Inc.  Suite 1000  1601 Fifth Avenue  Seattle, WA 98101  UNITED STATES |
|  |  |  |  |  |  |
| 1027 | Dr. Robert Michael Griffin Jr. |  | Dr. Michael Allen Borofsky  Dr. Brent William Calhoon  Dr. Jane Crosby  Dr. Brian Anthony DelVecchio  Amy B. Keefe  Dr. Saurin Mrugank Mehta  Dr. Peter Daniel Nicholas Jr.  Jennifer C. Renninger  Dr. Nancy Jane Walker  Dr. Jerome Stephen Weisberg | Clinical Research Center of Reading, LLP  401 Buttonwood Street  West Reading, PA 19611  UNITED STATES  Clinical Research Center of Reading, LLP  2760 Century Boulevard  Wyomissing, PA 19610  UNITED STATES | Quorum Review IRB  Suite 1000  1601 Fifth Avenue  Seattle, WA 98101  UNITED STATES |
|  |  |  |  |  |  |
| 1028 | Dr. Carol Lynn Danning |  | Janet L. Bahr  Sharon I. Barnhart  Nancy J. Davidson  Dr. Guy Peter Fiocco  Dr. Jack M. Lockhart  Lynette D. Storlie  Dr. Peter Arndt Valen  Diane J. Webster | Gundersen Clinic, Ltd  1836 South Avenue  LaCrosse  Onalaska, WI 54601  UNITED STATES  Gundersen Clinic, Ltd  Mail Stop NC3-001  3111 Gundersen Drive  Onalaska, WI 54650  UNITED STATES  Gundersen Clinic, Ltd  3111 Gundersen Drive  Onalaska, WI 54650  UNITED STATES | Gundersen Clinic, Ltd Human Subjects Committee  1836 South Avenue  La Crosse, WI 54601  UNITED STATES |
|  |  |  |  |  |  |
| 1029 | Dr. Dayton Dennis Payne Jr. |  | Dr. Ronald David Caldwell Jr.  Christopher G. Dowd  Dr. John Keith Earl  Kimberly Granger  Dr. Jill Anne McClory  Dr. Crystal L. Olson | PMG Research Hickory, LLC  Suite 202  1781 Tate Boulevard Southeast  Hickory, NC 28601  UNITED STATES  PMG Research Hickory, LLC  225 18th Street Southeast  Hickory, NC 28602  UNITED STATES  PMG Research Hickory, LLC  Suite 202  1781 Tate Boulevard Southeast  Hickory, NC 28602  UNITED STATES | Quorum Institutional Review Board  Suite 1000  1601 Fifth Avenue  Seattle, WA 98101  UNITED STATES  Quorum Review, Inc.  Suite 1000  1601 Fifth Avenue  Seattle, WA 98101  UNITED STATES |
|  |  |  |  |  |  |
| 1030 | Dr. Pietro Vito Rocca |  |  | Delaware Arthritis and Osteoporosis Center  Suite 101  537 Stanton-Christiana Road  Newark, DE 19713  UNITED STATES | Quorum Institutional Review Board  Suite 1000  1601 Fifth Avenue  Seattle, WA 98101  UNITED STATES |
|  |  |  |  |  |  |
| 1031 | Dr. Geneva Louise Hill |  | DeEtte M. Burton  Dr. Josette J. Johnson  Dr. Jeffrey Geldert Lawson | Palmetto Clinical at Piedmont Arthritis  Suite 400  3 St. Francis Dr  Greenville, SC 29601  UNITED STATES  Palmetto Clinical Trial Services, LLC  611 NE Main Street  Simpsonville, SC 29681  UNITED STATES  Piedmont Arthritis Clinic, PA  Suite 400  3 Saint Francis Drive  Greenville, SC 29601  UNITED STATES | Quorum Review, Inc.  Suite 1000  1601 Fifth Avenue  Seattle, WA 98101  UNITED STATES |
|  |  |  |  |  |  |
| 1032 | Dr. Nathan Wei |  | Theresa M. Gillis | Arthritis Treatment Center  71 Thomas Johnson Drive  Frederick, MD 21702  UNITED STATES  The Arthritis and Osteoporosis Center of Maryland  71 Thomas Johnson Drive  Frederick, MD 21702  UNITED STATES | Quorum Institutional Review Board  Suite 1000  1601 Fifth Avenue  Seattle, WA 98101  UNITED STATES  Quorum Review, Inc.  Suite 1000  1601 Fifth Avenue  Seattle, WA 98101  UNITED STATES |
|  |  |  |  |  |  |
| 1040 | Dr. Emily Jane Herron Box |  | Dr. John Franklyn Babich  Dr. Patrick N. Box  Dr. Ashrito Kumar Dayal | Box Arthritis & Rheumatology of the Carolinas, PLLC  Suite 100  10502 Park Road  Charlotte, NC 28210  UNITED STATES | Quorum Review IRB  Suite 1000  1601 Fifth Avenue  Seattle, WA 98101  UNITED STATES |
|  |  |  |  |  |  |
| 1092 | Dr. Paul Andrew Dura |  | Jennifer L. Bubel  Kristin J. Contro  Aspen L. D'Angelo  Dr. Thomas Joseph Oven  Susan A. Wenzinger | Regional Rheumatology Associates  Suite 302  161 Riverside Drive  Binghamton, NY 13905  UNITED STATES | Quorum Review, Inc.  Suite 1000  1601 Fifth Avenue  Seattle, WA 98101  UNITED STATES |
|  |  |  |  |  |  |
| 1094 | Dr. Mark Christopher Genovese |  | Dr. Eliza Farmer Chakravarty  Dr. Lorinda Susan Chung  Dr. Scott T. Kawamoto  Dr. William Hewitt Robinson  Dr. Andrew Lee Rozelle  Dr. Laura F. Su  Dr. Paul J. Utz | Stanford Health Services  Medical Specialty Clinics  300 Pasteur Dr. A175  Stanford, CA 94305  UNITED STATES  Stanford Health Services GCRC  HG130  300 Pasteur Drive  Stanford, CA 94305  UNITED STATES  Stanford Investigational Pharmacy  Drug Shipment  H0301  300 Pasteur Drive  Stanford, CA 94305  UNITED STATES  Stanford University School of Medicine  Suite 203  Division of Immunology and Rheumatology  1000 Welch Road  Palo Alto, CA 94304  UNITED STATES | Stanford University Administrative Panel on Human Subjects in Medical Research  1215 Welch Road  Module A  Stanford, CT 94305-5401  UNITED STATES |
|  |  |  |  |  |  |
| 1095 | Dr. Steen Erik Mortensen |  |  | Via Christi Clinic, P.A.  Rheumatology Department  3311 East Murdock  Wichita, KS 67208  UNITED STATES | Quorum Institutional Review Board  Suite 1000  1601 Fifth Avenue  Seattle, WA 98101  UNITED STATES |
|  |  |  |  |  |  |
| 1096 | Dr. Cummins Lue |  | Dr. James Howard Abraham III  Dr. Laura Ballard Trigg  Dr. Robert M. Brewer  Dr. Stephen D. Holt  Dr. Richard William Houk  Dr. Lisa Ann Jarvis Lowery  Dr. S. Michael Jones  Dr. Margaret A. West | Little Rock Diagnostic Clinic, P.A.  1001 Lile Drive  Little Rock, AR 72205  UNITED STATES | Quorum Review Inc.  Suite 1000  1601 Fifth Avenue  Seattle, WA 98101  UNITED STATES  Quorum Review IRB Incorporated  Suite 1000  1601 Fifth Avenue  Seattle, WA 98101  UNITED STATES |
|  |  |  |  |  |  |
| 1098 | Dr. Michael Steven Brooks |  | Dr. Shahin Bagheri  Dr. Steven Eyanson  Beth Pierce  Tracy Lynn Stepanek  Diane Welsh | Physicians Clinic of Iowa  Suite PCI  202 Tenth Street SE  Cedar Rapids, IA 52403  UNITED STATES | Quorum Review, Inc.  Suite 1000  1601 Fifth Avenue  Seattle, WA 98101  UNITED STATES |
|  |  |  |  |  |  |
| 1100 | Dr. Patrick Thomas Schuette |  | Dr. William Joseph Arnold  Dr. Erin L. Arnold  Dr. Alfonso Eduardo Bello  Dr. Susan B. Broy  Dr. Gerald Marc Eisenberg  Dr. Ami K. Kothari  Dr. Mary Lynn Moran  Dr. Amanda K. Myers  Dr. John L. Skosey | Illinois Bone and Joint Institute, LLC  9000 Waukegan Road  Morton Grove, IL 60053  UNITED STATES | Quorum Review, Inc.  Suite 1000  1601 Fifth Avenue  Seattle, WA 98101  UNITED STATES |
|  |  |  |  |  |  |
| 1101 | Dr. Richard James Misischia (Previous PI)  Dr. Robert J. Capps |  | Dr. Marcin T. Gornisiewicz  Carla P. Hoskins  Dr. Jay Henderson Warrick  Sharon Y. West  Dr. Donna M. Winn  Dr. John Frederick Wolfe | Rheumatology Consultants, PLLC  Suite 200 - Colony Park  4707 Papermill Drive  Knoxville, TN 37909-1907  UNITED STATES | Quorum Review Institutional Review Board Incorporated  Suite 1000  1601 Fifth Avenue  Seattle, WA 98101  UNITED STATES |
|  |  |  |  |  |  |
| 1105 | Dr. Luis Rolan Espinoza |  | Dr. Raquel S. Cuchacovich | Louisana State University Health Sciences Center  Department of Rheumatology  3700 St. Charles Ave. Room 431  New Orleans, LA 70115  UNITED STATES  Louisiana State University Health Sciences Center  Department of Rheumatology  Suite 890  2820 Napoleon Avenue  New Orleans, LA 70115  UNITED STATES  Louisiana State University Health Sciences Center  Department of Rheumatology  4th Floor  3700 St. Charles Avenue  New Orleans, LA 70115  UNITED STATES | LSU Health Sciences Center  New Orleans  Institutional Review Board  433 Bolivar Street  New Orleans, LA 70112  UNITED STATES |
|  |  |  |  |  |  |
| 1106 | Dr. Basit A. Malik |  | Nathalie Brink  Dr. Dennis Kurt Buth  Cynthia L. James  Dr. Gregory F. Lakin  Vicky C. Rockhill  Dr. Neal B. Secrist  Cassaundra Shultz  Kimberly L. Talbot | Professional Research Network of Kansas  Suite 400  345 Riverview  Wichita, KS 67203  UNITED STATES | Quorum Review Institutional Review Board Incorporated  Suite 1000  1601 Fifth Avenue  Seattle, WA 98101  UNITED STATES |
|  |  |  |  |  |  |
| 1107 * | Dr. Stephen Anthony Ruhlman |  | Dr. Arnold Leslie Katz  Dr. John Albert Mallory  Dr. Lawrence David Riffel  Dr. Bruce Herschel Short | Pinnacle Medical Research  Suite 101  10601 Quivira Road  Overland Park, KS 66215  UNITED STATES | Quorum Review, Inc.  Suite 1000  1601 Fifth Avenue  Seattle, WA 98101  UNITED STATES |
|  |  |  |  |  |  |
| 1108 | Dr. Philip Judson Mease |  | Dr. Catherine Jennifer Bakewell  Kori Anne Dewing  Nicole Marie Furfaro  Jane Park  Lyne Ann Schaefer-Alfonse  Christy Vath  Susan Lucille Williams-Judge | Investigational Drug Service  747 Broadway  Seattle, WA 98122  UNITED STATES  Seattle Rheumatology Associates  Suite 600  601 Broadway  Seattle, WA 98122  UNITED STATES  Swedish Clinical Research  601 BROADWAY  Suite 600  Seattle, WA 98122  UNITED STATES  Swedish Medical Center  747 Broadway  Seattle, WA 98122  UNITED STATES | Western Institutional Review Board (WIRB)  3535 Seventh Avenue Southwest  Olympia, WA 98502  UNITED STATES |
|  |  |  |  |  |  |
| 1109 | Dr. Alan Jan Kivitz |  | Angela Marie-Zumer Braatz  Dr. Lori Ann Lavelle  Dr. Frederick Timothy Murphy  Dr. Marianne L. Shaw  Tamara L. Smith  Michael Joseph Zumer | Altoona Center for Clinical Research  175 Meadowbrook Lane  Duncansville, PA 16635  UNITED STATES | Quorum Review, Inc.  Suite 1000  1601 Fifth Avenue  Seattle, WA 98101  UNITED STATES |
|  |  |  |  |  |  |
| 1112 | Dr. William C. Gough III |  | Dr. Kristin Marie Gowin  Audrey W. Little  Dr. Christopher G. Meyer  Dr. Ellison Leon Smith  Dr. Jill Scott Vargo  Corinne H. Yelton | Asheville Rheumatology & Osteoporosis Research Associates, P.A.  Suite 304  445 Biltmore Center  Asheville, NC 28801  UNITED STATES  Asheville Rheumatology & Osteoporosis Research Associates, P.A.  Suite 304  445 Biltmore Center  Asheville, NC 28801  UNITED STATES  Asheville Rheumatology & Osteoporosis Research Associates, PA  Suite 200  4 Vanderbilt Park Drive  Asheville, NC 28803  UNITED STATES  Asheville Rheumatology and Osteoporosis Research Associates  Suite 304  445 Biltmore Center  Asheville, NC 28801  UNITED STATES | Quorum Review, Inc.  Suite 1000  1601 Fifth Avenue  Seattle, WA 98101  UNITED STATES |
|  |  |  |  |  |  |
| 1113 | Dr. Michael James Fairfax |  | Beth A. Elliott  W. Richard Horn  Amanda L. Lewis  Courtney G. McDaniel  Dr. Charles S. Mitchell  Courtney K. Ramirez  Peter L. Trethewey | ArthroCare, Arthritis Care & Research P.C.  Suite 200  3921 East Baseline Road  Gilbert, AZ 85234  UNITED STATES | Quorum Review, Inc.  Suite 1000  1601 Fifth Avenue  Seattle, WA 98101  UNITED STATES |
|  |  |  |  |  |  |
| 1114 | Dr. Jeffrey Robert Curtis |  | Martha L. Sanderson | The University of Alabama at Birmingham  Arthritis Clinical Intervention Program - SRC 076  1717 Sixth Avenue South  Birmingham, AL 35294  UNITED STATES | Western Institutional Review Board  3535 Seventh Avenue Southwest  Olympia, WA 98502  UNITED STATES |
|  |  |  |  |  |  |
| 1117 * | Dr. Kyle Woodrow Strader |  | Dr. Douglas Garland Freeman Jr.  Dr. Louie E. Tsiktsiris | N.C. Arthritis and Allergy Care Center, P.A.  3831 Merton Drive  Raleigh, NC 27609  UNITED STATES | Quorum Review, Inc.  Suite 1000  1601 Fifth Avenue  Seattle, WA 98101  UNITED STATES |
|  |  |  |  |  |  |
| 1120 | Dr. Stephen Michael Lindsey |  | Lisa Casteigne Alleman  Dr. Bobby J. Dupre  Sharon Rockhold Holder  Kristina K. Rau  Dr. Sean E. Shannon | Ochsner Clinic Foundation - Baton Rouge  2nd Floor  9001 Summa Avenue  Baton Rouge, LA 70809  UNITED STATES  Ochsner Clinic Foundation - Baton Rouge  Department of Clinical Research - 3rd Floor  9001 Summa Avenue  Baton Rouge, LA 70809  UNITED STATES | Ochsner Institutional Review Board  Brent House - Room 505  1514 Jefferson Highway  New Orleans, LA 70121  UNITED STATES |
|  |  |  |  |  |  |
| 1153 | Dr. David Hilton Sikes |  | Donna Day  Dr. Mark Sol Eisner  Dr. Natalie Ann Faith  Michelle L. Meyer  Dr. Nathan Arnold Meyer  Dr. Ernesto Rodriguez  Marokhaya Samb  Dr. Amarilis Torres | Florida Medical Clinic, P.A.  Clinical Research Division  38135 Market Square  Zephyrhills, FL 33542  UNITED STATES | Quorum Institutional Review Board  Ethics Committee - CRGH  Concord Repatriation General Hospital - Building 75  Hospital Road  Concord, NSW 2139  AUSTRALIA  Quorum IRB  Suite 1000  1601 Fifth Avenue  Seattle, WA 98101  UNITED STATES  Quorum Review IRB Incorporated  Suite 1000  1601 Fifth Avenue  Seattle, WA 98101  UNITED STATES  Quorum Review, Inc.  Suite 1000  1601 Fifth Avenue  Seattle, WA 98101  UNITED STATES |
|  |  |  |  |  |  |
| 1171 | Dr. Atul Kumar Singhal |  | Gina Agnew  Julia A. Dilliard  Doris C. Harvey  Dr. John M. Joseph  Kristen Lois Lyons  Steven I. Macofsky  Melissa Nicho  Yolanda Perez  Dr. Guillermo A. Quiceno  Sandra Jean Rodriguez | Southwest Rheumatology Research, LLC  Suite 615  18601 LBJ Freeway  Mesquite, TX 75150  UNITED STATES | Quorum Review Institutional Review Board Incorporated  Suite 1000  1601 Fifth Avenue  Seattle, WA 98101  UNITED STATES |
|  |  |  |  |  |  |
| 1172 * | Dr. Debra R. Michel |  | Dr. Santina Carminati Taddei | Clinical Research Advantage, Inc./  East Valley Rheumatology & Osteoporosis, PC  3921 East Baseline Road, Suite 108  Gilbert, AZ 85234  UNITED STATES | Quorum Review, Inc.  Suite 1000  1601 Fifth Avenue  Seattle, WA 98101  UNITED STATES |
|  |  |  |  |  |  |
| 1190 | Dr. Seppo E. Rapo |  | Dr. John Franklin Berry  Dr. David J. Brown  Janet Callaghan  Susan G. Cannon  Dr. Benjamin Dichter Gordon  Dr. Kathleen A. Kerrigan  Jennifer B. Mahannah  Dr. Herbert O. Mathewson  Dr. James Arthur McCarthy | Clinical Research Center of Cape Cod, Inc.  134 Ansel Hallet Road  West Yarmouth, MA 02673  UNITED STATES  Clinical Research Center of Cape Cod, Inc.  131 Attucks Lane  Hyannis, MA 02601  UNITED STATES | Quorum Review, Inc.  Suite 1000  1601 Fifth Avenue  Seattle, WA 98101  UNITED STATES |
|  |  |  |  |  |  |
| 1191 | Dr. Charles L. Ludivico |  | Susan Marshall Durkin  Nancy Katherine McFadden  Maxine R. Paden  Erica L. Rau  Dr. Allen Jeffrey Samuels  Dr. Ranju Singh | East Penn Rheumatology Associates, PC  Suite 402 and 501  701 Ostrum Street  Bethlehem, PA 18015  UNITED STATES | Quorum Review IRB  Suite 1000  1601 Fifth Avenue  Seattle, WA 98101  UNITED STATES |
|  |  |  |  |  |  |
| 1192 | Dr. Shelly Pearl Kafka |  | Dr. Laura J. Stavrakis | Mountain State Clinical Research  Suite 303A  300 Davisson Run Road  Clarksburg, WV 26301  UNITED STATES  Mountain State Clinical Research  395 Emily Drive  Clarksburg, WV 26301  UNITED STATES  Mountain State Clinical Research  Suite 104  300 Davisson Run Road  Clarksburg, WV 26301  UNITED STATES  United Hospital Center Clinical Trials Office  395 Emily Road  Clarksburg, WV 26301  UNITED STATES | Quorum Review, Inc.  Suite 1000  1601 Fifth Avenue  Seattle, WA 98101  UNITED STATES |
|  |  |  |  |  |  |
| 1193 | Dr. Melvin Albert Churchill Jr. |  | Dr. Rick Charles Chatwell  Dr. Julie A. Collier  Heather A. Janes-Sorensen  Dr. Lisa Kastanek  Anne Robinson Lorenz  Dr. Kristina Ann Tyndall  Dr. Robert Michael Valente | Arthritis Center of Nebraska  3901 Pine Lake Road  LINCOLN, NE 68516  UNITED STATES  Physician Research Collaboration, LLC  Suite 120  3901 Pine Lake Road  Lincoln, NE 68516  UNITED STATES | Quorum Review IRB  Suite 1000  1601 Fifth Street  Seattle, WA 98101  UNITED STATES |
|  |  |  |  |  |  |
| 1194 | Dr. Steven Dell Mathews |  | Dr. Darlene Maria Bartilucci  Dr. Alicia D. Campbell  Dr. Ramon B. Castello  Dr. Erin Gautier Doty  Dr. Susan Neims Greco  Dr. Keith R. Holden  Dr. Jeffry Alan Jacqmein  Dr. Michael Jay Koren  Dr. Alpa Mahendra Patel  Dawn Marie Robison  Dr. Neil Sager  Dr. Carolyn Minh Tran  Dr. Francis P. Valenzuela | Jacksonville Center for Clinical Research  Suite 1  4085 University Boulevard South  Jacksonville, FL 32216  UNITED STATES | Quorum Institutional Review Board  Suite 1000  1601 Fifth Avenue  Seattle, WA 98101  UNITED STATES |
|  |  |  |  |  |  |
| 1196 | Dr. Neil J. Gonter |  | Alexandru F. Kimel  Dr. Ralph E. Marcus | Rheumatology Associates of North Jersey  1415 Queen Anne Road  Teaneck, NJ 07666  UNITED STATES | Quorum Review, Inc.  Suite 1000  1601 Fifth Avenue  Seattle, WA 98101  UNITED STATES |
|  |  |  |  |  |  |
| 1197 | Dr. Nicholas A. Patrone |  |  | Boice-Willis Clinic  Suite 320  901 North Winstead Avenue  Rocky Mount, NC 27804  UNITED STATES | Quorum Institutional Review Board  Suite 1000  1601 Fifth Avenue  Seattle, WA 98101  UNITED STATES  Quorum Review, Inc.  Suite 1000  1601 Fifth Avenue  Seattle, WA 98101  UNITED STATES |
|  |  |  |  |  |  |
| 1198 | Dr. John Joseph Condemi |  | Terry F. Arnold  Jill S. Bernhardt  Dr. Peter Michael Grace Deane  Dr. Anatole K. Kleiner  Dr. Emmanuel Adolphus Quaidoo  Erika Spiroff | AAIR Research Center  Suite 305  300 Meridian Centre  Rochester, NY 14618  UNITED STATES | Quorum Review, Inc.  Suite 1000  1601 Fifth Avenue  Seattle, WA 98101  UNITED STATES |
|  |  |  |  |  |  |
| 1199 * | Dr. Luis A. Toro |  | Jill R. Garrett | Lovelace Scientific Resources  Suite 560  5741 Bee Ridge Road  Sarasota, FL 34233  UNITED STATES  The Arthritis Specialty Centre  Suite 550  5741 Bee Ridge Road  Sarasota, FL 34233  UNITED STATES | Quorum Review, Inc.  Suite 1000  1601 Fifth Avenue  Seattle, WA 98101  UNITED STATES |
|  |  |  |  |  |  |
| 1201 | Dr. Bridget Tyrell Walsh |  | Dr. Laurie Ann Bergstrom  Michelle K. Cornett  Dr. Michael Joseph Maricic  Dr. Sabina R. Mian  Dr. Deborah Jane Power  Dr. Berchman Austin Vaz | Catalina Pointe Clinical Research  Suite 100  7520 North Oracle Road  Tucson, AZ 85704  UNITED STATES | Quorum Review, Inc.  Suite 1000  1601 Fifth Avenue  Seattle, WA 98101  UNITED STATES |
|  |  |  |  |  |  |
| 1203 | Dr. Jeffrey Stewart Neal |  | Dr. Kelly K. Cole  Dr. Rita M. Egan  Dr. Paul M. Goldfarb Jr. | Bluegrass Community Reaserch, Inc.  330 Waller Avenue  Lexington, KY 40504  UNITED STATES | Quorum Institutional Review Board  Suite 1000  1601 Fifth Avenue  Seattle, WA 98101  UNITED STATES  Quorum Review, Inc.  Suite 1000  1601 Fifth Avenue  Seattle, WA 98101  UNITED STATES |
|  |  |  |  |  |  |
| 1204 | Dr. Jody Kay Hargrove |  | Dr. Paul Harvey Waytz  Dr. David Charles Zoschke | Arthritis and Rheumatology Consultants, P.A.  7250 France Avenue South  Suite 215  Edina, MN 55435  UNITED STATES | Quorum Review, Inc.  Suite 1000  1601 Fifth Avenue  Seattle, WA 98101  UNITED STATES |
|  |  |  |  |  |  |
| 1206 | Dr. Raymond Edward Jackson |  | Dr. Inocencio A. Cuesta  Dr. Eric John Knipple | Premier Imaging Center, PLC  Suite 100  31500 Telegraph Road  Bingham Farms, MI 48025  UNITED STATES  QUEST Research Institiute  Suite 230  31000 Telegraph  Bingham Farms, MI 48025  UNITED STATES | Quorum Review, Inc.  Suite 1000  1601 Fifth Avenue  Seattle, WA 98101  UNITED STATES |
|  |  |  |  |  |  |
| 1208 | Dr. Alan Lawrence Brodsky |  | Dr. Pooja Banerjee  Stacey L. Cupit  Stacey L. Cupit | Arthritis Care and Diagnostic Center, P.A.  8440 Walnut Hill Lane  Suite 340  Dallas, TX 75231  UNITED STATES | Quorum Review, Inc.  Suite 1000  1601 Fifth Avenue  Seattle, WA 98101  UNITED STATES |
|  |  |  |  |  |  |
| 1209 | Dr. Paul L. Katzenstein |  | Sherry L. Booz  Dr. Mark Stephens Box | Kansas City Internal Medicine  Suite 350  20 Northeast St. Luke's Boulevard  Lee's Summit, MO 64086  UNITED STATES | Quorum Review, Inc.  Suite 1000  1601 Fifth Avenue  Seattle, WA 98101  UNITED STATES |
|  |  |  |  |  |  |
| 1210 * | Dr. Alvin Francis Wells |  | Mary J. Haning  Nicole M. Jodat  Kristen L. Ribar  Nicole T. Rivecca | Medical Diagnostic Imaging  3111 West Rawson Avenue  Franklin, WI 53132  UNITED STATES  Rheumatology and Immunotherapy Center  Suite 101  200 East Ryan Road  Oak Creek, WI 53154  UNITED STATES | Quorum Review, Inc.  Suite 1000  1601 Fifth Avenue  Seattle, WA 98101  UNITED STATES |
|  |  |  |  |  |  |
| 1211 | Dr. Karen Sue Kolba |  | Beverley Duarte | Pacific Arthritis Center Medical Group  607 East Plaza Drive  Suite A  Santa Maria, CA 93454  UNITED STATES | Quorum Review, Inc.  Suite 1000  1601 Fifth Avenue  Seattle, WA 98101  UNITED STATES |
|  |  |  |  |  |  |
| 1213 | Dr. David William Bouda  Dr. Suresh Kumar Reddy Pasya (Previous PI)  Dr. Talha Shamim (Previous PI) |  | Dr. Krishan Ariyarathna  Melanie Budine  Dr. Alfred David Felber  Kylie M. Hutsell  Shannon R. Stafford | Heartland Clinic Research, Inc.  2201 North 90th Street  Suite 125-126  Omaha, NE 68134  UNITED STATES | Quorum Review, Inc.  Suite 1000  1601 Fifth Avenue  Seattle, WA 98101  UNITED STATES |
|  |  |  |  |  |  |
| 1214 | Dr. Erdal Diri |  | Jerane A. Forsberg  Kristen A. Hedgepeth  Kristen A. Schoen | Trinity Health Center - Medical Arts  400 Burdick Expressway East  Minot, ND 58701  UNITED STATES | Trinity IRB  Institutional Review Board  One Burdick Expressway West  Minot, ND 58701  UNITED STATES |
|  |  |  |  |  |  |
| 1215 | Dr. Michael Eugene Sayers |  | Dr. Michael Roger Baker  Dr. Martha Leigh D'Ambrosio  Dr. Melissa T. Hocate  Dr. Megan C. MacNeil  Bonnie L. Miranda  Pamela G. Saufley  Deborah Ann Spoerl | Arthritis Associates and Osteoporosis Center of Colorado Springs  Suite 200  215 Parkside Drive  Colorado Springs, CO 80910  UNITED STATES | Quorum Review, Inc.  Suite 1000  1601 Fifth Avenue  Seattle, WA 98101  UNITED STATES |
|  |  |  |  |  |  |
| 1216 | Dr. Charles Allen Birbara |  | Dr. Sheela Kumar  Dr. Nassif F. Maalouli  Dr. Basheer Rahmoun | Clinical Pharmacalogy Study Group  25 Oak Ave.  Worcester, MA 01605  UNITED STATES  Clinical Pharmacology Study Group  26 Queen Street  Worcester, MA 01610  UNITED STATES | Quorum Review, Inc.  Suite 1000  1601 Fifth Avenue  Seattle, WA 98101  UNITED STATES |
|  |  |  |  |  |  |
| 1217 | Dr. Selden Longley III |  | Dr. Meghavi Sheth Kosboth  Dr. Thomas Mark Lloyd Sr. | Southeastern Arthritis Center  Suite 8  4343 West Newberry Road  Gainesville, FL 32607  UNITED STATES  Southeastern Imaging & Diagnostics  Imaging Only  Suite 5  4343 West Newberry Road  Gainesville, FL 32607  UNITED STATES  Southeastern Integrated Medical, PL  d/b/a Florida Medical Research Institute  Suite 17  4343 West Newberry Road  Gainesville, FL 32607  UNITED STATES | Quorum Review, Inc.  Suite 1000  1601 Fifth Avenue  Seattle, WA 98101  UNITED STATES |
|  |  |  |  |  |  |
| 1218 | Dr. Herbert Stuart Block Baraf |  | Dr. Ashley D. Beall  Dr. Paul John DeMarco  Dr. Emma G. DiIorio  Dr. Alan Ken Matsumoto  Dr. Guada R. Respicio  Dr. Robert Lawrence Rosenberg  Dr. Evan Lloyd Siegel | Center for Rheumatology and Bone Research  Suite 306  2730 University Boulevard West  Wheaton, MD 20902  UNITED STATES | Quorum Review, Inc.  Suite 1000  1601 Fifth Avenue  Seattle, WA 98101  UNITED STATES |
|  |  |  |  |  |  |
| 1219 | Dr. Haydon Anthony Moorman  Dr. James D. Taborn (Previous PI) |  | Dr. Douglas G. Campbell  Dr. Andrew Daugavietis  Deborah Hotchkiss  Kimberly A. Kendall  Lynn M. Perez  Dr. Stacey E. Watson | Borgess Internal Medicine  Suite 124  1717 Shaffer Street  Kalamazoo, MI 49048  UNITED STATES  Borgess Research Institute  Suites 003 and 004  1717 Shaffer Street  Kalamazoo, MI 49048  UNITED STATES  Borgess Rheumatology  2490 S. 11th Street  Kalamazoo, MI 49009  UNITED STATES | Quorum Review  Review  Suite 1000  1601 Fifth Avenue  Seattle, WA 98101  UNITED STATES |
|  |  |  |  |  |  |
| 1221 | Dr. Chokkalingam Siva |  | Dr. Shantanu Bishwal  Dr. Fernando X. Castro  Deanna K. Davenport  Dr. Kenneth G. Lawlor  Dr. Celso R. Velazquez | University of Missouri-Columbia  One Hospital Drive  M746 Heath Services Center  Columbia, MO 65212  UNITED STATES  University Physicians  Woodrail Clinic  Building 2, Suite 150  1000 West Nifong  Columbia, MO 65203  UNITED STATES | Health Sciences Institutional Review Board  University of Missouri - Columbia  190 Galena Hall  Columbia, MO 65212  UNITED STATES |
|  |  |  |  |  |  |
| 1222 | Dr. William Rodney Palmer |  | Dr. Michael Gray Feely  Dr. Magdalena Maria Fiksinski  Dr. Kristin Sue Lake  Dr. Marcus Hilton Snow | Westroads Medical Group  10170 Nicholas Street  Omaha, NE 68114  UNITED STATES | Quorum Institutional Review Board  Suite 1000  1601 Fifth Avenue  Seattle, WA 98101  UNITED STATES  Quorum Review, Inc.  Suite 1000  1601 Fifth Avenue  Seattle, WA 98101  UNITED STATES |
|  |  |  |  |  |  |
| 1223 | Dr. Ara Hagop Dikranian |  | Dr. Puja Chitkara  Dr. Michael Ira Keller  Timothy F. Lazarek  Jennifer Marconato  Dr. Smitha Chiniga Reddy | San Diego Arthritis Medical Clinic  Suite 300  3633 Camino Del Rio South  San Diego, CA 92108  UNITED STATES | Quorum Review, Inc.  Suite 1000  1601 Fifth Avenue  Seattle, WA 98101  UNITED STATES |
|  |  |  |  |  |  |
| 1225 | Dr. Dale George Halter |  | Bessie B. Ahrendt  Dr. Harvey I. Hyman  Dr. Frank Leo Lanza  Dr. Sean X. Tao  Ryan A. Valicek  Dr. Frank R. Wellbourne  Dr. Francis M. Williams | Houston Institute for Clinical Research  Suite 720  7777 Southwest Freeway  Houston, TX 77074  UNITED STATES | Quorum Review, Inc.  Suite 1000  1601 Fifth Avenue  Seattle, WA 98101  UNITED STATES |
|  |  |  |  |  |  |
| 1228 | Dr. James Craig VanDeWall |  | Dr. Ross Arthur Horsley  Josephine Raab  Dr. Victor Ramon Rodriguez | Southern Tier Arthritis and Rheumatism  415 North 8th Street  Olean, NY 14760  UNITED STATES | Quorum Institutional Review Board  Suite 1000  1601 Fifth Avenue  Seattle, WA 98101  UNITED STATES |
|  |  |  |  |  |  |
| 1231 | Dr. William Julius Shergy |  | Theresa Ann Causey  Dr. Kun Chen  Dr. Jesus Hernandez  Dr. Robert Edward Hunt  Dr. Robert Macon Phillips Jr. | Rheumatology Associates of North Alabama, P.C.  Suites 600 & 620  201 Sivley Road  Huntsville, AL 35801  UNITED STATES | Quorum Institutional Review Board  Suite 1000  1601 Fifth Avenue  Seattle, WA 98101  UNITED STATES  Quorum Review, Inc.  Suite 1000  1601 Fifth Avenue  Seattle, WA 98101  UNITED STATES |
|  |  |  |  |  |  |
| 1238 | Dr. Ira Francis Fenton |  | Dr. Neil B. Perlman  Dr. Carl M. Silberman  Cecelia Yvette Thurman | Deerbrook Medical Associates  Suite 116  565 Lakeview Parkway  Vernon Hills, IL 60061  UNITED STATES | Quorum Review, Inc.  Suite 1000  1601 Fifth Avenue  Seattle, WA 98101  UNITED STATES |
|  |  |  |  |  |  |
| 1240 | Dr. Beata Joanna Filip-Majewski |  | Katie Hartshorn  Dr. Leslie D. McCasland | NEA Baptist Clinic  4802 East Johnson Avenue  Jonesboro, AR 72401  UNITED STATES  NEA Baptist Clinic - Clinical Research Center  Suite C  416 East Washington Avenue  Jonesboro, AR 72401  UNITED STATES  Obsolete Address  311 East Matthews Avenue  Jonesboro, AR 72401  UNITED STATES | Quorum Review, Inc.  Suite 1000  1601 Fifth Avenue  Seattle, WA 98101  UNITED STATES |
|  |  |  |  |  |  |
| 1241 * | Dr. Moges Sisay |  | Dr. Richard Earnest Bell  Dr. Mujtaba F. Tapal | TSARR, LLC  (Tri-State Arthritis and Rheumatology Research, LLC)  3801 Bellemeade Avenue, Suite 320  Evansville, IN 47714  UNITED STATES | Quorum Review, Inc.  Suite 1000  1601 Fifth Avenue  Seattle, WA 98101  UNITED STATES |
|  |  |  |  |  |  |
| 1242 | Dr. Farrukh Zaidi |  | Dr. Lisa Mauri Cohen  Dr. M. Sami Mughni | Florida Arthritis & Osteoporosis Center  8029 Washington Street  Port Richey, FL 34668  UNITED STATES  Suncoast Clinical Research, Inc.  5604 Gulf Drive  New Port Richey, FL 34652  UNITED STATES | Quorum Institutional Review Board  Suite 1000  1601 Fifth Avenue  Seattle, WA 98101  UNITED STATES  Quorum Review, Inc.  Suite 1000  1601 Fifth Avenue  Seattle, WA 98101  UNITED STATES |
|  |  |  |  |  |  |
| 1243 | Dr. Steven Charles Kimmel |  | Dr. Alan Richard Alberts  Dr. Elias Halpert  Dr. Kevin Elliot Stone | West Broward Rheumatology Associates, Inc.  Suite 300  7431 North University Drive  Tamarac, FL 33321  UNITED STATES | Quorum Review, Inc.  Suite 1000  1601 Fifth Avenue  Seattle, WA 98101  UNITED STATES |
|  |  |  |  |  |  |
| 1246 | Dr. Anupama Savithri Bhat  Dr. Douglas Menzies Haselwood (Previous PI) |  | Dr. Chandan D.S. Cheema  Harold Jesse Ewing  Dr. Douglas Menzies Haselwood | Med Investigations  Administrative Office  Suite 260  6600 Mercy Court  Fair Oaks, CA 95628  UNITED STATES  Office of Anupama Bhat, MD  151 North Sunrise Avenue  Suite 1201  Roseville, CA 95661  UNITED STATES  Office of Douglas M. Haselwood, MD  Suite 1201  151 North Sunrise Avenue  Roseville, CA 95661  UNITED STATES  Office of Douglas Menzies Haselwood, MD  Suite A  6608 Mercy Court  Fair Oaks, CA 95628  UNITED STATES  Sierra Rheumatology  Suite 1201  151 North Sunrise Avenue  Roseville, CA 95661  UNITED STATES | Quorum Review, Inc.  Suite 1000  1601 Fifth Avenue  Seattle, WA 98101  UNITED STATES |
|  |  |  |  |  |  |
| 1251 | Dr. Geoffrey Stephen Gladstein |  | Jennifer Battaglia  Jennifer Battaglia  Laura Brothers  Janette Charles  Dr. Mirela Dumitrescu  Dr. Germano A. Guadagnoli  Mary Ellen Porrata  Milena Seara  Wiltora M. Stanley  Beth Carlson Tuohy  Margaret E. Ziegler | New England Research Associates, LLC  Suite 101  5520 Park Avenue  Trumbull, CT 06611  UNITED STATES | Quorum Review, Inc.  Suite 800  1501 Fourth Avenue  Seattle, WA 98101  UNITED STATES |
|  |  |  |  |  |  |
| 1252 | Dr. Michael Thomas Stack |  | Norma S. Anderson  Dr. Richard M. Knapek | Diagnostic Rheumatology and Research PC  6447 South East Street  Suite A  Indianapolis, IN 46227  UNITED STATES | Quorum Institutional Review Board  Suite 1000  1601 Fifth Avenue  Seattle, WA 98101  UNITED STATES  Quorum Review, Inc.  Suite 1000  1601 Fifth Avenue  Seattle, WA 98101  UNITED STATES |
|  |  |  |  |  |  |
| 1254 | Dr. Michael Wayne Grisanti |  | Dr. Karen Bir  Dr. Joseph Michael Grisanti  Dr. J. Cairn Marrale  Dr. Mary Margaret O'Neil  Dr. Ivan Sabio  Marcy Sheehan  Kathleen A. Ziomek | Buffalo Rheumatology  Suite 100  3055 Southwestern Boulevard  Orchard Park, NY 14127  UNITED STATES | Quorum Review, Inc.  Suite 1000  1601 Fifth Avenue  Seattle, WA 98101  UNITED STATES |
|  |  |  |  |  |  |
| 1255 | Dr. Jefrey Dale Lieberman |  | Nancy Ellen Green | Jeffrey D. Lieberman, MD, PC  2712 North Decatur Road  Decatur, GA 30333  UNITED STATES | Quorum Review, Inc.  Suite 1000  1601 Fifth Avenue  Seattle, WA 98101  UNITED STATES |
|  |  |  |  |  |  |
| 1260 | Dr. Stuart Michael Weisman |  | Denise M. Munro  Jean Petrecz  Jennifer R. Roland | Boulder Medical Center, PC  2750 Broadway Street  Boulder, CO 80304  UNITED STATES | Quorum Institutional Review Board  Suite 1000  1601 Fifth Avenue  Seattle, WA 98101  UNITED STATES  Quorum Review, Inc.  Suite 1000  1601 Fifth Avenue  Seattle, WA 98101  UNITED STATES |
|  |  |  |  |  |  |
| 1261 | Dr. Mohammed Yaseen Abubaker |  | Nancy Ellen Green | Marietta Rheumatology  Suite 100  670 North Avenue  Marietta, GA 30060  UNITED STATES | Quorum Review, Inc.  Suite 1000  1601 Fifth Avenue  Seattle, WA 98101  UNITED STATES |
|  |  |  |  |  |  |
| 1266 | Dr. Deborah Dyett Desir |  | Jennifer L. Becker  Dr. C. Roxana Ciubotaru  Grace M. Cortezzo  Anita B. DeAngelo  Ms. Penelope K. Dubin  Dr. Sonia Gordon-Dole  Dr. Victor Martin  Dr. Vivian C. Shih | Arthritis & Osteoporosis Center, PC  3018 Dixwell Avenue  Hamden, CT 06518  UNITED STATES | Quorum Review, Inc.  Suite 1000  1601 Fifth Avenue  Seattle, WA 98101  UNITED STATES |
|  |  |  |  |  |  |
| 1270 | Dr. Ruth Ditzian Kadanoff |  | Dr. Fariha Kausar  Dr. Rochella Abaygar Ostrowski  Dr. John Arthur Robinson II | Loyola University Medical Center  2160 South First Avenue  Maywood, IL 60153  UNITED STATES | Loyola University Medical Center, Committee for the Protection of Human Subjects  2160 South First Avenue  Maywood, IL 60153  UNITED STATES |
|  |  |  |  |  |  |
| 1281 | Dr. Andrew Keith Solomon (Previous PI)  Dr. Richard Alan Houdek Jimenez |  | Dr. Julie Lyn Carkin  Myla R. Morales-Tomas  Dr. Steven Scott Overman  Dr. Jeff Regan Peterson  Dr. Andrew Keith Solomon | The Seattle Arthritis Clinic  Suite 250  10330 Meridian Avenue North  Seattle, WA 98133  UNITED STATES | Quorum Review, Inc.  Suite 1000  1601 Fifth Avenue  Seattle, WA 98101  UNITED STATES |
|  |  |  |  |  |  |
| 1282 * | Dr. Richard Thomas Meehan |  | Linda Bannister  Dr. Marc D. Cohen | National Jewish Health  1400 Jackson Street  Denver, CO 80206  UNITED STATES | Quorum Review, Inc.  Suite 1000  1601 Fifth Avenue  Seattle, WA 98101  UNITED STATES |
|  |  |  |  |  |  |
| 1283 | Dr. John Irving Reed |  | Dr. John Thomas Hosey  Marcia S. Kirkpatrick  Candace LeBlanc  Dr. Julie V. Levengood  Dr. Alice Anne S. Williams  Dr. Robert Alan Yood | Fallon Clinic, Inc.  165 Mill Street  Leominster, MA 01453  UNITED STATES  Fallon Clinic, Inc.  640 Lincoln Street  640 Lincoln Street, MA 01605  UNITED STATES  Fallon Clinic, Inc. at  Worcester Medical Center  123 Summer Street  Worcester, MA 01608  UNITED STATES | Fallon Community Health Plan IRB  640 Lincoln Street  Worcester, MA 01605  UNITED STATES |
|  |  |  |  |  |  |
| 1284 | Dr. Michael Edmund Luggen |  | Laura Mary Alexander  Brandy Lynn Alexander  Laura Mary Alexander  Mary Margaret Bengel-Rogers  Dr. Kerrin D. Burte  Dr. Matthew Franklin Burton  Dr. Gregory Joseph DeLorenzo  Dr. David Greenblatt  Karie Kathern King  Peggy Ann Slattery | Cincinnati Rheumatic Disease Study Group, Inc.  311 Straight Street  Cincinnati, OH 45219  UNITED STATES | Quorum Institutional Review Board  Suite 1000  1601 Fifth Avenue  Seattle, WA 98101  UNITED STATES |
|  |  |  |  |  |  |
| 1310 | Dr. Arnaldo Torres |  | Ingrid Ferro-Spilde  Susan Fisher  Luis M. Garzon | BayCare Outpatient Imaging at Bardmoor  *Imaging Only*  8787 Bryan Dairy Road  Largo, FL 33777  UNITED STATES  DMI Research, Inc.  6699 90th Avenue North  Pinellas Park, FL 33782  UNITED STATES  St. Petersburg Arthritis Center  6711 38th Avenue North  St. Petersburg, FL 33710  UNITED STATES | Quorum Institutional Review Board  Suite 1000  1601 Fifth Avenue  Seattle, WA 98101  UNITED STATES  Quorum Review, Inc.  Suite 1000  1601 Fifth Avenue  Seattle, WA 98101  UNITED STATES |
|  |  |  |  |  |  |
| 1465 | Dr. William Patrick Knibbe |  | Michelle Hicks  Janeen Jenkins  Dr. James Edward Loveless | St. Luke's Clinic - Rheumatology  (Clinic Location)  Suite 100  600 West Robbins Road  Boise, ID 83702  UNITED STATES  St. Luke's Clinic - Rheumatology  (Secondary Clinic Location)  Suite 1234  520 South Eagle Road  Meridian, ID 83642  UNITED STATES  St. Luke's Intermountain Research Center  (Drug Shipment and Administration)  Suite 402  600 West Robbins Road  Boise, ID 83702  UNITED STATES | Quorum Review, Inc.  Suite 1000  1601 Fifth Avenue  Seattle, WA 98101  UNITED STATES |
|  |  |  |  |  |  |
| 1470 | Dr. William Stohl |  | Sandra Cardenas  Michael Elist  Dr. Reena Kapadia Heyer  Dr. Roshan Kotha  David Brian Kovacs  Adam Kreitenberg  Dr. Minh Chau Nguyen  Dr. Elizabeth Ortiz  Winnie Kate Pang  Dr. Anne Violet Quismorio  Dr. Shylaja Rachabattula  Rosy Rajbhandary  Hitasha Singh  Dr. Li Yang  Karen Cahit Yeter | Keck Medicine of USC  Department of Rheumatology - HMR 711  2011 Zonal Avenue - HMR 711  Los Angeles, CA 90033  UNITED STATES | University of Southern California Health Science Campus Institutional Review Board  LAC/USC Medical Center  General Hospital Suite 4700  1200 North State Street  Los Angeles, CA 90033  UNITED STATES |
|  |  |  |  |  |  |
| 1472 | Dr. Robert Greig Trapp |  | Belinda S. Brown  Monica Peregrin | The Arthritis Center  1200 Centre West Drive  Springfield, IL 62704  UNITED STATES | Quorum Review IRB Incorporated  Suite 1000  1601 Fifth Avenue  Seattle, WA 98101  UNITED STATES  Quorum Review, Inc.  Suite 1000  1601 Fifth Avenue  Seattle, WA 98101  UNITED STATES |
|  |  |  |  |  |  |
| 1473 | Dr. Charles Meredith King II |  | Dr. David Kevin Asa  Dr. George Alfred Housley Jr.  Dr. Amit Bhaskar Prasad  Brandon L. Young | North Mississippi Medical Clinics, Inc.  845 South Madison Street  Tupelo, MS 38801  UNITED STATES | North Mississippi Health Services Institutional Review Board  830 South Gloster Street  Tupelo, MS 38801  UNITED STATES |
|  |  |  |  |  |  |
| 1474 | Dr. Carl Patrick Griffin  Dr. Larry Grant Willis (Previous PI)  Dr. William Martin Schnitz (Previous PI) |  | Lindsey May Gillispie  Dr. Norman Kerr Imes  Dr. William Martin Schnitz | Lynn Health Science Institute  Suite 800  3555 Northwest 58th Street  Oklahoma City, OK 73112  UNITED STATES | Quorum Review IRB Incorporated  Suite 1000  1601 Fifth Avenue  Seattle, WA 98101  UNITED STATES  Quorum Review, Inc.  Suite 1000  1601 Fifth Avenue  Seattle, WA 98101  UNITED STATES |
|  |  |  |  |  |  |
| 1476 | Dr. Micha Abeles |  | Dr. Aryeh M. Abeles  Dr. Santhanam Lakshminarayanan  Kim Schwartz  Kim Striegel  Dr. John Vischio | University of Connecticut Health Center  263 Farmington Avenue  Farmington, CT 06030-5353  UNITED STATES | University of Connecticut Health Center Institutional Review Board  16 Munson Road  Farmington, CT 06030  UNITED STATES |
|  |  |  |  |  |  |
| 1478 | Prof. Arthur Francis Kavanaugh |  | Dr. Arnold Ceponis  Dr. Jeannie Huan Chao  Dr. Kenneth Carekin Kalunian  Dr. Susan Jung-Ah Lee | University of California  Center for Innovative Therapy  Suite# 2-078  9444 Medical Center Drive  LaJolla, CA 92037-0943  UNITED STATES  University of California San Diego  9320 Campus Point Drive  La Jolla, CA 92037  UNITED STATES  University of California San Diego Center for Innovative Therapy  Suite 2-078  9444 Medical Center Drive  La Jolla, CA 92093  UNITED STATES | Human Research Protections Program  MC0052  9500 Gilman Drive  La Jolla, CA 92093-0052  UNITED STATES  University of California San Diego Human Research Protections Program  9444 Medical Center Drive  East Campus Office Building (ECOB) First Floor  La Jolla, CA 92093  UNITED STATES |
|  |  |  |  |  |  |
| 1479 | Dr. Charles Henry Pritchard |  | Dr. David J. Chesner  Dr. Elana R. Eisner  Dr. Charles Michael Franklin  Dr. Dennis A. Jerdan  Dr. Mark Lopatin | Rheumatic Disease Associates, Ltd.  2360 Maryland Road  Willow Grove, PA 19090  UNITED STATES | Quorum Review IRB Incorporated  Suite 1000  1601 Fifth Avenue  Seattle, WA 98101  UNITED STATES  Quorum Review, Inc.  Suite 1000  1601 Fifth Avenue  Seattle, WA 98101  UNITED STATES |
|  |  |  |  |  |  |
| 1480 | Dr. James Michael Calmes |  | Dr. Naga S. Bushan  Dr. Jose Del Giudice  Dr. Jitendra Indrukumar Vasandani | Arthritis and Osteoporosis Associates, LLP  5220 80th Street  Lubbock, TX 79424  UNITED STATES | Quorum Review, Inc.  Suite 1000  1601 Fifth Avenue  Seattle, WA 98101  UNITED STATES |
|  |  |  |  |  |  |
| 1481 | Dr. Christina Marie Charles-Schoeman |  | Amber A. Bechtel  Dr. Daniel Eric Furst  Dr. Veena Kittane Ranganath  Maricela Rodriguez | UCLA Medical Center  Division of Rheumatology  1000 Veteran Ave, C-3259  Los Angeles, CA 90095  UNITED STATES  University of California Los Angeles  32-59 Rheumatology  1000 Veteran Avenue  Rehab Center  Los Angeles, CA 90095  UNITED STATES  University of California Los Angeles - David Geffen School of Medicine - Division of Rheumatology  1000 Veteran Avenue  Third Floor - Rehab Center  Los Angeles, CA 90095  UNITED STATES | Office for the Protection of Research Subjects  Suite 102  11000 Kinross Avenue  Los Angeles, CA 90095  UNITED STATES  Oklahoma Medical Research Foundation - Institutional Review Board  825 Northeast 13th Street  Oklahoma City, OK 73104  UNITED STATES  Quorum Review  Review  Suite 1000  1601 Fifth Avenue  Seattle, WA 98101  UNITED STATES |
|  |  |  |  |  |  |
| 1482 | Dr. Adrienne Hollander |  | Dr. Stephen L. Burnstein  Dr. James Paul Dwyer  Dr. Brian Lee Grimmett  Dr. Kenneth Howard Maurer  Dr. Michael Charles Schuster  Dr. Arielle S. Silver  Dr. Sheldon D. Solomon | Arthritis, Rheumatic & Back Disease Associates  Suite 101  2309 Evesham Road  Voorhees, NJ 08043  UNITED STATES | Quorum Review, Inc.  Suite 1000  1601 Fifth Avenue  Seattle, WA 98101  UNITED STATES |
|  |  |  |  |  |  |
| 1483 | Dr. David Andrew Bong (Previous PI)  Dr. Theresa M. Karplus |  | Dr. Esther C. Hwang  Dr. Uzma A. Khan  Dr. Marcia J. Sparling | The Vancouver Clinic, Inc., PS  700 Northeast 87th Avenue  Vancouver, WA 98664  UNITED STATES  The Vancouver Clinic, Inc., PS  700 Northeast 87th Avenue  Suite 260  Vancouver, WA 98664  UNITED STATES | Quorum Review, Inc.  Suite 1000  1601 Fifth Avenue  Seattle, WA 98101  UNITED STATES |
|  |  |  |  |  |  |
| 1484 | Dr. Joseph Eugene Huffstutter |  | Dr. William David Craig | Arthritis Associates, PLLC  1035 Executive Drive  Hixson, TN 37343  UNITED STATES | Quorum Review, Inc.  Suite 1000  1601 Fifth Avenue  Seattle, WA 98101  UNITED STATES |
|  |  |  |  |  |  |
| 1485 | Dr. Gary Edward Myerson |  | Anna F. Adams  Dr. Paul George Sutej | Arthritis and Rheumatology of Georgia  Suite 220  980 Johnson Ferry Road  Atlanta, GA 30342  UNITED STATES | Quorum Review IRB Incorporated  Suite 1000  1601 Fifth Avenue  Seattle, WA 98101  UNITED STATES  Quorum Review, Inc.  Suite 1000  1601 Fifth Avenue  Seattle, WA 98101  UNITED STATES |
|  |  |  |  |  |  |
| 1486 | Dr. William F. C. Rigby |  | Dr. Daniel Arthur Albert  Dr. Lin Anita Jeromin Brown  Cheryl L. Carlson  Gregory Challener  Dr. Richard C. Chou  Dr. Jeff S. Croteau  Dr. Lauren A. Dudley  Carey Field  Whitney F. Hilton  Dr. Jonathan D. Jones  Zsolt Kulcsar  Dr. John N. Mecchella  Dr. Alireza Meysami  Dr. Nicole M. Orzechowski  Irene Shyu  Dr. Robert L. Wortmann | Dartmouth-Hitchcock Medical Center  Rheumatology Clinic 5C  One Medical Center Drive  Lebanon, NH 03756  UNITED STATES | Dartmouth College  Committee for the Protection of Human Subjects  3rd Floor #6254  63 South Main Street  Hanover, NH 03755  UNITED STATES |
|  |  |  |  |  |  |
| 1488 * | Dr. Suthin Songcharoen |  | Courtney Stanford | Arthritis and Osteoporosis Treatment and Research Center  Suite 300  2550 Flowood Drive  Flowood, MS 39232  UNITED STATES | Quorum Review, Inc.  Suite 1000  1601 Fifth Avenue  Seattle, WA 98101  UNITED STATES |
|  |  |  |  |  |  |
| 1489 * | Dr. James Chad Byrd |  | Dr. Steven Richard Kaster  Dr. Peter Douglas Rutherford  Dr. Galen R. Sorom | Wenatchee Valley Medical Center  Clinical Research Department  820 North Chelan Avenue  Wenatchee, WA 98801  UNITED STATES | Quorum Review Inc.  Suite 1000  1601 Fifth Avenue  Seattle, WA 98101  UNITED STATES |
|  |  |  |  |  |  |
| 1490 | Dr. Philip A. Waller |  | Eduardo Mansur  Jorge Mendez  Dan Nguyen  Dr. Gineth Paola Pinto-Patarroyo  Barbara A. Slusher  Dr. Prashanth Reddy Sunkureddi  DeAnna Elena Wallace | Accurate Clinical Research, Inc.  12553 Gulf Freeway  Houston, TX 77034  UNITED STATES | Quorum Review IRB Incorporated  Suite 1000  1601 Fifth Avenue  Seattle, WA 98101  UNITED STATES  Quorum Review, Inc.  Suite 1000  1601 Fifth Avenue  Seattle, WA 98101  UNITED STATES |
|  |  |  |  |  |  |
| 1492 | Dr. David Ralph Mandel |  | Katy E. Eichas  Sue M. Morecki  Patricia L. Scott  Deborah K. Torres  Joana Zula | Office of David R. Mandel, MD, Inc.  Suite 106  6551 Wilson Mills Road  Mayfield Village, OH 44143  UNITED STATES | Quorum Review IRB Incorporated  Suite 1000  1601 Fifth Avenue  Seattle, WA 98101  UNITED STATES |
|  |  |  |  |  |  |
| 1493 | Dr. Craig Davis Scoville |  | Susan H. Hughes | Institute of Arthritis Research  2220 East 25th Street  Idaho Falls, ID 83404  UNITED STATES | Quorum Review IRB Incorporated  Suite 1000  1601 Fifth Avenue  Seattle, WA 98101  UNITED STATES  Quorum Review, Inc.  Suite 1000  1601 Fifth Avenue  Seattle, WA 98101  UNITED STATES |
|  |  |  |  |  |  |
| 1498 | Dr. Jeffrey Alan Alper |  | Alisha Krysten Nicole Walker | Jeffrey Alper MD Research  689 Ninth Street North  Naples, FL 34102  UNITED STATES | Quorum Review, Inc.  Suite 1000  1601 Fifth Avenue  Seattle, WA 98101  UNITED STATES |
|  |  |  |  |  |  |
| 1499 | Dr. Melanie Creech Barron  Dr. Rahul Keshav Patel (Previous PI) |  | Rosa Artola DO  Dr. Jeffrey Kyle Bacon  Susan N. Bitner  Sharon Natasha Cha  Dr. Michael Casey Coan  Linda Davis  Dr. Hugo Bittar Fonseca  Sunil T. Gupta DO  Dr. Cynthia Ann Jimenez-Williams  Dr. Kathryn Michele Lawson  Dr. Robert Larry Marshall  Dr. Deepti Patki  Sandra Powell CCRC  Dr. Bernard Ross Rubin  Dr. Mansi Shah  Dr. Kokila Thirumurthi  Dr. Beth Ann Valashinas | University of North Texas Health Science Center at Fort Worth  Department of Internal Medicine - Rheumatology  855 Montgomery Street  Fort Worth, TX 76107  UNITED STATES | University of North Texas Health Science Center at Fort Worth Institutional Review Board  3400 Camp Bowie Boulevard  Fort Worth, TX 76107  UNITED STATES |
|  |  |  |  |  |  |
| 1500 | Dr. Norman Brian Gaylis |  | Dr. Marcos E. Maldonado  Dr. Elana M. Oberstein  Julia P. Savloff | Arthritis and Rheumatic Disease Specialties  Suite 200  21097 Northeast 27th Court  Aventura, FL 33180  UNITED STATES | Quorum Review, Inc.  Suite 1000  1601 Fifth Avenue  Seattle, WA 98101  UNITED STATES |
|  |  |  |  |  |  |
| 1501 | Dr. Charles D. Radis  Dr. David Benjamin Talmadge (Previous PI) |  | Dr. Brian E. Daikh  Dr. Edward Fels  Dr. Stephanie D. Gartner-Fanburg  Dr. Brian J. Keroack  Dr. Marc Lawrence Miller | Rheumatology Associates  51 Sewell Street  Portland, ME 04102  UNITED STATES | Quorum Review IRB Incorporated  Suite 1000  1601 Fifth Avenue  Seattle, WA 98101  UNITED STATES  Quorum Review, Inc.  Suite 1000  1601 Fifth Avenue  Seattle, WA 98101  UNITED STATES |
|  |  |  |  |  |  |
| 1502 | Dr. Steven Jay Klein |  | Dawnetta S. Adams  Dr. Mary Patrice Howell | Klein and Associates, MD, PA  Suites C and D  921 Seton Drive  Cumberland, MD 21502  UNITED STATES | Quorum Review, Inc.  Suite 1000  1601 Fifth Avenue  Seattle, WA 98101  UNITED STATES |
|  |  |  |  |  |  |
| 1503 | Dr. Ralph Edward Bennett |  | Scott F. Brown  Dr. Paul H. Caldron  Shawna Carbonniere  Dr. Sheetal K. Chhaya  Dr. Saima Choham  Candace L. Coggin  Tiffany Anne Fotinos  Edith L. Gazo  Dana Hoffman  Miranda Isom  Dr. Romina Jajoo  Randy Jay  Maurisa O. Konya  Catherine Sue Lynn  Dr. Eric Alan Peters  Dora Quan  Dr. John Irwin Starr  Dr. Areena Swarup  Carol M. Swenson  Dr. John Robert Paul Tesser | Arizona Arthritis & Rheumatology Associates, P.C.  Suite 202  1500 South Dobson Road  PARADISE VALLEY, AZ 85202  UNITED STATES  Arizona Arthritis and Rheumatology Associates, PC  Suite 204  5601 West Eugie Avenue  Glendale, AZ 85304  UNITED STATES  Arizona Arthritis and Rheumatology Associates, PC  Suite 505  9305 West Thomas Road  Phoenix, AZ 85037  UNITED STATES  Arizona Arthritis and Rheumatology Research PLLC  Suite F-150  10599 North Tatum Boulevard  Paradise Valley, AZ 85253  UNITED STATES  Arizona Arthritis Research PLC  Suite 170  4550 E. Bell Road  Phoenix, AZ 85032-9385  UNITED STATES | Quorum Review, Inc.  Suite 1000  1601 Fifth Avenue  Seattle, WA 98101  UNITED STATES |
|  |  |  |  |  |  |
| 1504 | Dr. Jeffrey Sanders Ritter |  | Dayorky L. Carnero  Dr. Santiago M. deSolo  Luis A. Helago  Debbie Watson  Dr. Michael Alan Weitz | Center for Arthritis and Rheumatic Diseases  Suite #304  7190 Southwest 87 Avenue  Miami, FL 33173  UNITED STATES | Quorum Review Institutional Review Board Incorporated  Suite 1000  1601 Fifth Avenue  Seattle, WA 98101  UNITED STATES |
|  |  |  |  |  |  |
| 1505 * | Dr. Soumya M. Reddy |  | Dr. Svetlana Krasnokutsky  Dr. Euna Lee  Dr. Pamela B. Rosenthal  Dr. Jonathan Samuels  Dr. Jose Scher  Dr. Yusuf Yazici | New York University Hospital for Joint Diseases  Seligman Center for Advanced Therapeutics  246 East 20th Street  New York, NY 10003  UNITED STATES | New York University School of Medicine  Institutional Board of Research Associates  Building #VET 10 West  550 First Avenue  New York, NY 10016  UNITED STATES |
|  |  |  |  |  |  |
| 1531 * | Dr. Marcy Behar Bolster |  | Dr. Aleksandra Granath  Dr. Faye N. Hant  Dr. Corey M. Hatfield  Dr. Marina A. Pulini-Franks  Dr. Richard Michael Silver  Dr. Edwin Allan Smith  Dr. Martin A. Trojanowski | Medical University of South Carolina  Suite 912 - MSC 637  96 Jonathan Lucas Street  Charleston, SC 29425  UNITED STATES  Medical University of South Carolina - Rheumatology  5th Floor - Suite 503  135 Rutledge Avenue  Charleston, SC 29425  UNITED STATES  Medical University of South Carolina Investigational Drug Services  MH Room 161  169 Ashley Avenue  Charleston, SC 29425  UNITED STATES | Institutional Review Board for Human Research  Office of Research Integrity  Suite 601 - MSC 857  19 Hagood Avenue  Charleston, SC 29425  UNITED STATES |
|  |  |  |  |  |  |
| 1538 | Dr. Bruce Allan Miller  Dr. Joseph Stanley Habros (Previous PI) |  | Dr. Roberta L. Bruhn  Dr. Ana Chinchilla  Dr. Foster Timm McCarty III  Dr. Albert Q. Tejada  Jason T. Zatkovich | Radiant Research, Inc.  Suite 200  7555 E. Osborn Road  Scottsdale, AZ 85251  UNITED STATES | Quorum Review Institutional Review Board Incorporated  Suite 1000  1601 Fifth Avenue  Seattle, WA 98101  UNITED STATES |
|  |  |  |  |  |  |
| 1540 * | Dr. Angela M. McCain |  | Dr. Nkechinyere Emejuaiwe  Nga Kathy Nguyen | Texas Research Center, LP  Suite 245  16659 Southwest Freeway  Sugar Land, TX 77479  UNITED STATES | Quorum Review Institutional Review Board Incorporated  Suite 1000  1601 Fifth Avenue  Seattle, WA 98101  UNITED STATES |
|  |  |  |  |  |  |
| 1547 * | Dr. David Andrew McLain |  | Dr. Gary Louis Boyd  Dr. Raymond James Browne  Mary Elizabeth Helms  Rebecca Ann McNair  Dr. Christopher Walton Roney  Dr. James Sosnowchik  Dr. John Arthur Ward  Dr. Hayes Taylor Williams  Dr. Harold Andrew Wilson Jr. | Achieve Clinical Research, LLC  Suite 41  2017 Canyon Road  Birmingham, AL 35216  UNITED STATES  Birmingham Rheumatology  Suite 211  2022 Brookwood Medical Center Drive  Birmingham, AL 35209  UNITED STATES | Quorum Review Institutional Review Board Incorporated  Suite 1000  1601 Fifth Avenue  Seattle, WA 98101  UNITED STATES |
|  |  |  |  |  |  |
| 1548 | Dr. Wesley Tyra Mizutani |  | Dr. Joel C. Bartlett | Talbert Medical Group  19066 Magnolia Street  Huntington Beach, CA 92646  UNITED STATES | Quorum Review Institutional Review Board Incorporated  Suite 1000  1601 Fifth Avenue  Seattle, WA 98101  UNITED STATES |
|  |  |  |  |  |  |
| 1550 | Dr. Jacob Asher Aelion |  | Dr. Satish K. Odhav | Arthritis Clinic  Suite 400  371 North Parkway  Jackson, TN 38305  UNITED STATES | Quorum Review Institutional Review Board Incorporated  Suite 1000  1601 Fifth Avenue  Seattle, WA 98101  UNITED STATES |
|  |  |  |  |  |  |
| 1557 | Dr. John Patrick Lavery |  | Richard Grandjean  Doris C. Harvey  Mukesh Satodiya | Office of John P. Lavery, MD, PA  Suite 120  997 Raintree Circle  Allen, TX 75013  UNITED STATES | Quorum Review Institutional Review Board Incorporated  Suite 1000  1601 Fifth Avenue  Seattle, WA 98101  UNITED STATES |
|  |  |  |  |  |  |
| 1560 | Dr. Stanford Lee-Yu Peng |  | Dr. Jane Hoyt Buckner  Dr. Jeffrey Steven Carlin | Virginia Mason Medical Center  Mail Stop: X6-RHE  1100 Ninth Avenue  PO Box 900  Seattle, WA 98101  UNITED STATES  Virginia Mason Medical Center  Investigational Drug Service  MS:H3-PI  925 Seneca Street  Seattle, WA 98101  UNITED STATES | Quorum Review Institutional Review Board Incorporated  Suite 1000  1601 Fifth Avenue  Seattle, WA 98101  UNITED STATES |
|  |  |  |  |  |  |
| 1620 | Dr. Robert William Shurmur  Dr. Thomas F. Ignaczak (Previous PI) |  | Antonio P. Giannelli  Dr. Lakshmi Padma Kocharla  Dr. Daniela Renkiewicz | Associated Internal Medicine Specialists  Suite 302  2845 Capital Avenue Southwest  Battle Creek, MI 49015  UNITED STATES  Bronson Internal Medicine and Rheumatology  Suite 302  2845 Capital Avenue Southwest  Battle Creek, MI 49015  UNITED STATES | Quorum Review Institutional Review Board Incorporated  Suite 1000  1601 Fifth Avenue  Seattle, WA 98101  UNITED STATES |
|  |  |  |  |  |  |
| 1631 | Dr. Michael David Kohen |  | Dr. Carlos Javier Farach | Allergy, Asthma, Arthritis, & Lung  709 North Clyde Morris Boulevard  Daytona Beach, FL 32114  UNITED STATES  Millennium Research  Suite B2  1545 Hand Avenue  Ormond Beach, FL 32174  UNITED STATES | Quorum Review Institutional Review Board Incorporated  Suite 1000  1601 Fifth Avenue  Seattle, WA 98101  UNITED STATES |
|  |  |  |  |  |  |
| 1633 | Dr. Ewa Olech (Previous PI)  Dr. Joan Tenenbaum Merrill |  | Shulawn Akanmu  Dr. Eliza Farmer Chakravarty  Dr. Judith Ann James  Wede-Joy Punni  Dr. Aikaterini Thanou  Samera Vaseer | OKLAHOMA MEDICAL RESEARCH FOUNDATION OMRF  MS #22  825 N E 13TH STREET  Oklahoma City, OK 73104  UNITED STATES | Oklahoma Medical Research Foundation - Institutional Review Board  MS #9  825 NE 13th Street  Oklahoma City, OK 73104  UNITED STATES |
|  |  |  |  |  |  |
| 1643 | Dr. Robert Eugene Harrell Jr. |  | Dr. Stanley Charles Burns | Osborne Research Center, LLC  720 West Third Street  Little Rock, AR 72201  UNITED STATES | Quorum Review, Inc.  Suite 1000  1601 Fifth Avenue  Seattle, WA 98101  UNITED STATES |
|  |  |  |  |  |  |
