# Supplementary material for: Changes in serum creatinine in patients with active rheumatoid arthritis treated with tofacitinib: results from clinical trials
Source: Arthritis Res Ther. 2014 Jul 25;16(4):R158. doi: 10.1186/ar4673 (PMC4220634; doi:10.1186/ar4673)
Supplement: Supplementary file 3 — Additional file 3: List of Investigators and Corresponding Ethics Committees or Institutional Review Boards for the Phase 2 A3921025 study. (DOC 268 KB) [file 13075_2013_4378_MOESM3_ESM.doc]

# A4 LIST OF INVESTIGATORS AND CORRESPONDING ETHICS COMMITTEES OR INSTITUTIONAL REVIEW BOARDS

## Argentina

**Coordinating Investigators:**

<None Entered>

| **Center** | **Principal Investigator** | **Co-Investigator(s)** | **Sub-Investigator(s)** | **Address(es)** | **Institutional Review Board or Ethics Committee Address(es)** |
| --- | --- | --- | --- | --- | --- |
|  |  |  |  |  |  |
| 1056 | Dr. Osvaldo Daniel Messina |  | Dr. Fabiana Geliberti  Dr. Daniel A. Pate  Dr. Silvina Edith Suarez  Dr. Fabiana Geliberti  Dr. Daniel A. Pate  Raquel Isabel Ramenzoni  Dr. Silvina Edith Suarez | Centro de Investigaciones Reumatologicas y Osteologicas-C I R O  Larrea 1106- 4 B  Capital Federal, Buenos Aires (C1117ABH)  ARGENTINA | Comite Independiente de Etica para Ensayos en Farmacologia Clinica  Fundación de Estudios Farmacologicos y de Medicamentos (FEFyM)  J.E. Uriburu 774- Piso 1°  Buenos Aires, C1027AAP  ARGENTINA  Comite de Etica del Centro de Investigaciones Reumatologicas y Osteologicas  11 de Septiembre 2231 7° A  Buenos Aires, C1428AII  ARGENTINA |
|  |  |  |  |  |  |
| 1057 | Dr. Guillermo A. Tate |  | Dr. Mariel Susana Cutri  Dr. Eduardo Fabian Mysler  Paula Bruni  Dr. Mariana Caubet  Natalia Caviglia  Dr. Clauda Andrea Helling  Dr. Anastasia Secco  Dr. Maria Natalia Tamborenea | OMI - Organización Médica de Investigación  Uruguay 725, P.B.  Buenos Aires, C1013AAR  ARGENTINA | Comite Independiente de Etica para Ensayos en Farmacologia Clinica  Fundación de Estudios Farmacologicos y de Medicamentos (FEFyM)  Uriburu 774- Piso 1°  Buenos Aires, 1027  ARGENTINA  OMI Comite de Docencia e Investigacion  Uruguay 725, PB  Buenos Aires, C1013AAR  ARGENTINA |
|  |  |  |  |  |  |
| 1071 | Dr. Mariano Ricardo Ghirlanda |  | Dr. Sebastian Rossi Lopez  Dr. David Gustavo Starosiliz  Dr. Pablo De Caso  Dra. Ana Maria Freuler | Saint Dennis Medical Group S.A.  Av. Rivadavia 2206 6° A  Buenos Aires, C1034ACO  ARGENTINA | Comite Independiente de Etica para Ensayos en Farmacologia Clinica  Fundación de Estudios Farmacologicos y de Medicamentos (FEFyM)  J.E. Uriburu 774- Piso 1°  Buenos Aires, C1027AAP  ARGENTINA  Comite de Investigacion y Etica de Saint Dennis Medical Group S. A.  Av. Rivadavia 2206- 6°A  Buenos Aires, C1034ACO  ARGENTINA |
|  |  |  |  |  |  |
| 1072 | Dr. Oscar Luis Rillo |  | Dr. Luciana Casalla  Dra. Ana Maria Freuler  Dr. Lorena Salinas  Dr. Maria Alejandra Cusa  Dr. Silvia Papasidero  Maria Cristina Rillo Pratto | Centro Médico Dra. De Salvo  Av. Cabildo 1548, 1A  Buenos Aires, C1426ABP  ARGENTINA | Comite Independiente de Etica para Ensayos en Farmacologia Clinica  Fundación de Estudios Farmacologicos y de Medicamentos (FEFyM)  J.E. Uriburu 774- Piso 1°  Buenos Aires, C1027AAP  ARGENTINA  Comite Independiente de Etica para Ensayos en Farmacología Clínica  Av. Cabildo 1548- 1° Piso  Buenos Aires, C1426ABP  ARGENTINA |

## Brazil

**Coordinating Investigators:**

<None Entered>

| **Center** | **Principal Investigator** | **Co-Investigator(s)** | **Sub-Investigator(s)** | **Address(es)** | **Institutional Review Board or Ethics Committee Address(es)** |
| --- | --- | --- | --- | --- | --- |
|  |  |  |  |  |  |
| 1001 | Dr. Cristiano Augusto de Freitas Zerbini |  | Dr. Bruno César Cavalcanti dos Anjos  Dr. Wagner Ikehara  Dr. Andrea Barranjard Vannucci Lomonte  Dr. Silvia Caroline Santana Moura  Dr. Maria José Nunes  Dr. Luiza Helena Coutinho Ribeiro  Dr. Raissa Gomes Silva  Dr. Mariana G. Waisberg | Hospital Heliópolis - PAM  Serviço de Reumatologia  Rua Almirante Delamare, 1534  Sao Paulo, SP 04230-000  BRAZIL | Comite de Etica em Pesquisa do Complexo Hospitalar Heliopolis  Rua Conego Xavier, 276 - 10º andar  Sacomã  São Paulo, SP 04231-030  BRAZIL |
|  |  |  |  |  |  |
| 1002 | Dr. Antônio Carlos Ximenes |  | Dr. Rafael Navarrete Fernandez  Fabia M.G.P. Oliveira  Marcelo Pimenta  Dr. Bruno Nazeozeno Ribeiro | Hospital Geral de Goiânia Dr. Alberto Rassi  Departamento de Reumatologia  Av. Anhanguera, 6479  Setor Oeste  Goiânia, GO 74043-110  BRAZIL  Hospital Geral de Goiânia Dr. Alberto Rassi  Rua 9 B,129  Sala 303 - Setor Oeste  Goiania, GO 74110-120  BRAZIL | Comitê de Ética em Pesquisa Humana e Animal (CEPHA-HGG)  Hospital Geral de Goiânia Dr. Alberto Rassi  Av. Anhanguera, 6479  Setor Oeste  Goiânia, GO 74110-010  BRAZIL |
|  |  |  |  |  |  |
| 1003 | Dr. Flora M. D. Marcolino |  | Dr. Eduardo S. Meirelles | Hospital das Clínicas da Faculdade de Medicina da Universidade de São Paulo (HC-FMUSP)  I.O.T ¿ Instituto de Ortopedia e Traumatologia  Rua Dr. Ovídio Pires de Campos, 333  Sala A325 - 3º andar  São Paulo, SP 05403-010  BRAZIL | Comissão de Ética para Análise de Projetos de Pesquisa (CAPPesq)  Hospital das Clínicas e da Faculdade de Medicina da Universidade de São Paulo  Rua Dr. Ovidio Pires de Campos, 225 - 5o. andar - sala 505  Cerqueira Cesar  São Paulo, SP 05403-010  BRAZIL |
|  |  |  |  |  |  |
| 1004 | Sebastiao C. Radominski |  | Dr. Sinara da Silva Freitas  Andreas Funke  Maicon N. Loureiro  Dr. Lucila Stange Rezende  Alexandre G. Tavares  David Cezar Titton | Centro de Estudos em Terapias Inovadoras  Rua Padre Camargo, 241  Alto da Glória  Curitiba, PR 80060-240  BRAZIL  Hospital de Clínicas da UFPR  Rua General Carneiro, 181  Centro  Curitiba, PR 80060-900  BRAZIL | Comitê de Ética em Pesquisa em Seres Humanos do HC-UFPR  Rua General Carneiro, 181  Curitiba, PR 80060-900  BRAZIL |

## Bulgaria

**Coordinating Investigators:**

Rositsa Antova

Violeta Doneva

Spaska Velichkova

Evelina Yordanova

| **Center** | **Principal Investigator** | **Co-Investigator(s)** | **Sub-Investigator(s)** | **Address(es)** | **Institutional Review Board or Ethics Committee Address(es)** |
| --- | --- | --- | --- | --- | --- |
|  |  |  |  |  |  |
| 1103 | Prof. Zlatimir Kolarov |  | Dr. Penka Bekyarova  Dr. Daniela Dimitrova  Dr. Mariana Goycheva  Dr. Lubomir Marinov Marintchev  Dr. Simeon Monov  Assoc. Prof. Veneta Paskaleva-Peytcheva  Dr. Tzvetanka Petranova  Prof. Iordan Sheytanov  Dr. Ivan Sheytanov | Clinic of Rheumatology Multiprofile Hospital for Active treatment Sveti Ivan Rilski  13, Urvich Str.  Sofia, 1612  BULGARIA | Ethic Committee for Multicenter Trials  26, Yanko Sakazov  Sofia, 1504  BULGARIA  Ethics Committee at MHAT "Sveti Ivan Rilski"  Ethics Committee  15 Academician Ivan Geshov Blvd.  Sofia, 1431  BULGARIA |
|  |  |  |  |  |  |
| 1104 | Dr. Daniela Bichovska |  | Dr. Ivan Bichovski  Dr. Mario Markov | Clinic of Internal Diseases, Multiprofile Hospital for Active treatment Sveta Anna  1, Dimitar Mollov Str.  Sofia, 1709  BULGARIA | Ethic Committee for Multicenter Trials  26, Yanko Sakazov  Sofia, 1504  BULGARIA  Ethics Committee at MHAT "Sveta Anna"  Ethics Committee  1 Dimitar Mollov Str.  Sofia, 1709  BULGARIA |
|  |  |  |  |  |  |
| 1105 | Dr. Boycho Oparanov |  | Dr. Borislava Angelova Ilchova  Dr. Raycho Raychev  Dr. Ignat Zhutev | Clinic of Cardiology and Rheumatology, Military Medical Academy  3, Georgi Sofiiski Str.  Sofia 1606  BULGARIA | Ethic Committee for Multicenter Trials  26, Yanko Sakazov  Sofia, 1504  BULGARIA  Ethics Committee at Military Medical Academy  3, Georgi Sofiiski  Sofia, 1606  BULGARIA |

## Chile

**Coordinating Investigators:**

<None Entered>

| **Center** | **Principal Investigator** | **Co-Investigator(s)** | **Sub-Investigator(s)** | **Address(es)** | **Institutional Review Board or Ethics Committee Address(es)** |
| --- | --- | --- | --- | --- | --- |
|  |  |  |  |  |  |
| 1063 | Dr. Pedro Miranda |  | Dr. Jorge Saavedra  Dr. Cecilia Trejo | Oficina Privada Dr. Pedro Miranda  Avda. Bernardo O'Higgins 240, Oficina 25  Santiago, Santiago  CHILE | Comité Ético Científico  Servicio de Salud Metropolitano Oriente  Av. Salvador 364  Santiago  CHILE |
|  |  |  |  |  |  |
| 1069 | Dr. Oscar Neira |  | Dr. Patricia Foster  Dr. Sergio Palma  Dr. Maria José Villar | Hospital del Salvador  Reumatología  Av. Salvador 364  Providencia  Santiago, RM 7500922  CHILE | Comité Ético Científico  Servicio de Salud Metropolitano Oriente  Av. Salvador 364  Santiago  CHILE |
|  |  |  |  |  |  |
| 1089 | Dr. Renato Jimenez |  | Dr. Cecilia Georgi  Dr. Ricardo Jerez  Dr. Luis Roca | Hospital Gustavo Fricke  Alvarez 1532  Viña Del Mar, V Region 2570017  CHILE | Comite Etico Cientifico Hospital Dr. Gustavo Fricke  Alvarez 1532, 2do piso, oficina 3  Viña del Mar  CHILE |
|  |  |  |  |  |  |
| 1109 | Dr. Marta Aliste |  | Dr. Annaelise Goecke  Dr. Lilian Soto  Dr. Pamela Wurmann | Centro Médico Prosalud  Hernando de Aguirre 194, Oficina 21  Providencia, RM  CHILE | Comité Ético Científico  Servicio de Salud Metropolitano Oriente  Av. Salvador 364  Santiago  CHILE |

## Czech Republic

**Coordinating Investigators:**

<None Entered>

| **Center** | **Principal Investigator** | **Co-Investigator(s)** | **Sub-Investigator(s)** | **Address(es)** | **Institutional Review Board or Ethics Committee Address(es)** |
| --- | --- | --- | --- | --- | --- |
|  |  |  |  |  |  |
| 1097 | Dr. Sarka Forejtova |  | Dr. Dana Tegzova | Revmatologicky ustav  Na Slupi 4  Praha 2, 128 50  CZECH REPUBLIC | Eticka komise IKEM a FTNsP  Fakultni Thomayerova nemocnice s poliklinikou  Videnska 800  Praha 4 Krc, 140 59  CZECH REPUBLIC  Revmatologicky ustav  Eticka komise  Na Slupi 4  Praha 2, 128 50  CZECH REPUBLIC |
|  |  |  |  |  |  |
| 1098 | Dr. Jan Rosa |  | Dr. Petr Kasalicky | DC Mediscan  Sustova 1930  Praha 11 - Chodov, 148 00  CZECH REPUBLIC | Eticka komise IKEM a FTNsP  Fakultni Thomayerova nemocnice s poliklinikou  Videnska 800  Praha 4 Krc, 140 59  CZECH REPUBLIC |
|  |  |  |  |  |  |
| 1099 | Dr. Sevda Augustinova |  | Dr. Jan Augustin  Dr. Vera Vlasakova | MEDIPONT Plus, s.r.o.  Matice Skolske 17  Ceske Budejovice, 370 01  CZECH REPUBLIC | Eticka komise IKEM a FTNsP  Fakultni Thomayerova nemocnice s poliklinikou  Videnska 800  Praha 4 Krc, 140 59  CZECH REPUBLIC |
|  |  |  |  |  |  |
| 1100 | Dr. Petr Vitek |  |  | PV-Medical s.r.o.  Revmatologicka ambulance  Padelky I/3645  Zlin, 760 01  CZECH REPUBLIC | Eticka komise IKEM a FTNsP  Fakultni Thomayerova nemocnice s poliklinikou  Videnska 800  Praha 4 Krc, 140 59  CZECH REPUBLIC |
|  |  |  |  |  |  |
| 1101 | Dr. Petr Kopsa |  | Dr. Marie Sedlackova  Dr. Lenka Zouharova | Fakultni Thomayerova nemocnice s poliklinikou  Revmatologicke a rehabilitacni oddeleni  Videnska 800  Praha 4, 140 59  CZECH REPUBLIC | Eticka komise IKEM a FTNsP  Fakultni Thomayerova nemocnice s poliklinikou  Videnska 800  Praha 4 Krc, 140 59  CZECH REPUBLIC |
|  |  |  |  |  |  |
| 1102 | Dr. Leona Prochazkova |  | Dr. Jana Bohmova  Dr. Petr Nemec | Fakultni nemocnice u sv. Anny v Brne, II. Interni klinika  Pekarska 53  Brno, 656 91  CZECH REPUBLIC | Eticka komise Fakultni nemocnice u sv. Anny v Brne  Pekarska 53  Brno, 656 91  CZECH REPUBLIC  Eticka komise IKEM a FTNsP  Fakultni Thomayerova nemocnice s poliklinikou  Videnska 800  Praha 4 Krc, 140 59  CZECH REPUBLIC |

## Hungary

**Coordinating Investigators:**

<None Entered>

| **Center** | **Principal Investigator** | **Co-Investigator(s)** | **Sub-Investigator(s)** | **Address(es)** | **Institutional Review Board or Ethics Committee Address(es)** |
| --- | --- | --- | --- | --- | --- |
|  |  |  |  |  |  |
| 1093 | Dr. Eleonora Nemeth |  | Dr. Tibor Nemes | Selye Janos Korhaz es Rendelointezet  Szechenyi u. 2.  Komarom, H-2921  HUNGARY | Medical Research Council Ethics Committee for Clinical Pharmacology  Arany J. u. 6-8  Budapest, H-1051  HUNGARY |
|  |  |  |  |  |  |
| 1094 | Dr. Istvan Szombati |  | Dr. Eleni Kanakaridu  Dr. Karoly Nagy  Dr. Krisztina Szabo | Synexus Magyarorszag Kft.  Becsi u. 61.  Budapest, H-1036  HUNGARY | Medical Research Council Ethics Committee for Clinical Pharmacology  Arany J. u. 6-8  Budapest, H-1051  HUNGARY |
|  |  |  |  |  |  |
| 1095 | Dr. Attila Kovacs |  | Dr. Judit Biro  Dr. Marianna Czifra  Dr. Laszlo Samson  Dr. Maria Takacs | MAV Korhaz es Rendelointezet  Versehy F. u. 6-8.  Szolnok, H-5000  HUNGARY | Medical Research Council Ethics Committee for Clinical Pharmacology  Arany J. u. 6-8  Budapest, H-1051  HUNGARY |
|  |  |  |  |  |  |
| 1096 | Dr. Antal Insperger |  | Dr. Edit Drescher  Dr. Timea Kocsy  Dr. Judit Pulai  Dr. Tamas Szabo | Veszprem Megyei Onkormanyzat Csolnoky Ferenc Korhaz-Rendelointezet  Korhaz u. 1.  Veszprem, H-8200  HUNGARY | Medical Research Council Ethics Committee for Clinical Pharmacology  Arany J. u. 6-8  Budapest, H-1051  HUNGARY |

## Mexico

**Coordinating Investigators:**

<None Entered>

| **Center** | **Principal Investigator** | **Co-Investigator(s)** | **Sub-Investigator(s)** | **Address(es)** | **Institutional Review Board or Ethics Committee Address(es)** |
| --- | --- | --- | --- | --- | --- |
|  |  |  |  |  |  |
| 1058 | Dr. Virginia Pascual |  | Dr. Marina Rull-Gabayet | Instituto Nacional de Ciencias Medicas y Nutricion Salvador Zubiran  Departamento de Inmunologia y Reumatologia  Vasco de Quiroga 15  Col Seccion XVI Tlalpan  Mexico, DF 14000  MEXICO | Instituto Nacional de Ciencias Medicas y Nutrición Salvador Zubiran  Comite Institucional de Investigacion Biomedica en Humanos  VASCO DE QUIROGA 15  COL SECCION XVI TLALPAN  Mexico, DF 14000  MEXICO |
|  |  |  |  |  |  |
| 1060 | Dr. Mario H. Cardiel-Rios |  | Maria Dolores Alonso-Martinez  Rosa Janete Ramirez-Tapia | Star Medica  Centro de Investigacion Clinica de Morelia SC.  Virrey de Mendoza 1998-502/416  Felix Ireta  Morelia, Michoacan 58070  MEXICO | Star Medica  Comité de la Calidad de la Atencion Medica, Credenciales, Ensenanza,  Investigacion, Capacitacion y Etica, Evaluacion del Expediente Clinico  Virrey de Mendoza 2000  Felix Ireta  Morelia, Michoacan 58070  MEXICO |

## Poland

**Coordinating Investigators:**

<None Entered>

| **Center** | **Principal Investigator** | **Co-Investigator(s)** | **Sub-Investigator(s)** | **Address(es)** | **Institutional Review Board or Ethics Committee Address(es)** |
| --- | --- | --- | --- | --- | --- |
|  |  |  |  |  |  |
| 1053 | Prof. Janusz Badurski |  | Dr. Stefan Daniluk  Dr. Anna Jarmoc  Dr. Elzbieta Zofia Jeziernicka  Dr. Nonna Nowak | Niepubliczny Zaklad Opieki Zdrowotnej  Centrum Osteoporozy i Chorob Kostno-Stawowych  Ul. Warynskiego 6/2  Bialystok, 15-461  POLAND | Komisja Bioetyczna przy Okregowej Izbie Lekarskiej  ul. Swietojanska 7  Bialystok, 15-082  POLAND |
|  |  |  |  |  |  |
| 1054 | Dr. Wieslawa Porawska |  | Dr. Kamilla Klama  Dr. Wlodzimierz Piotrowski | Poznanski Osrodek Medyczny  'Novamed'  Ul. Sniadeckich 7/2  Poznan, 60-773  POLAND | Komisja Bioetyczna przy Okregowej Izbie Lekarskiej  ul. Swietojanska 7  Bialystok, 15-082  POLAND |
|  |  |  |  |  |  |
| 1055 | Dr. Artur Racewicz |  | Dr. Malgorzata Fiedorczyk  Dr. Sylwia Kalinko  Dr. Krystyna Kuc  Dr. Dorota Laszcz  Dr. Sylwia Izabela Raczynska  Dr. Jerzy Supronik | SP ZOZ Wojewodzki Szpital Zespolony im. Jedrzeja Sniadeckiego  Oddzial Chorob Wewnetrznych i Reumatologii  ul. M. Curie Sklodowskiej 25  Bialystok, 15-950  POLAND | Komisja Bioetyczna przy Okregowej Izbie Lekarskiej  ul. Swietojanska 7  Bialystok, 15-082  POLAND |
|  |  |  |  |  |  |
| 1061 * | Dr. Waclaw Palczynski |  | Dr. Elzbieta P. Pawlaczyk-Strugala  Dr. Cezary Strugala | Niepubliczny Zaklad Opieki Zdrowotnej, Grudziadzkie Centrum Pomocy  "Dobra Praktyka Lekarska"  ul. Chelminska 74  Grudziadz, 86-300  POLAND | Komisja Bioetyczna przy Okregowej Izbie Lekarskiej  ul. Swietojanska 7  Bialystok, 15-082  POLAND |
|  |  |  |  |  |  |
| 1062 | Dr. Jaroslaw Marcinkiewicz |  | Dr. Iwonna Lajborek-Czyz  Dr. Ewa Wojtowicz  Dr. Romana Zak | Wojewodzki Zespol Reumatologiczny im. dr J. Titz-Kosko  ul. Grunwaldzka 1/3  Sopot, 81-759  POLAND | Komisja Bioetyczna przy Okregowej Izbie Lekarskiej  ul. Swietojanska 7  Bialystok, 15-082  POLAND |
|  |  |  |  |  |  |
| 1113 | Dr. Zofia Ruzga |  | Dr. Radoslaw Janiak  Dr. Ewa Jazwinska-Tarnawska  Dr. Anna Sidorowicz-Bialynicka  Dr. Renata Wojtala | "SYNEXUS SCM" Sp. z o.o.  Swobodna 8a  Wroclaw, 50-088  POLAND | Komisja Bioetyczna przy Okregowej Izbie Lekarskiej  ul. Swietojanska 7  Bialystok, 15-082  POLAND |
|  |  |  |  |  |  |
| 1114 | Dr. Ines Pokrzywnicka-Gajek |  | Dr. Andrzej Sawicki  Dr. Barbara Zalewska | Lecznica Specjalistow, Centrum Medyczne "Osteomed" NZOZ  Al. Krakowska 110/114  Warszawa, 02-256  POLAND | Komisja Bioetyczna przy Okregowej Izbie Lekarskiej  ul. Swietojanska 7  Bialystok, 15-082  POLAND |

## Slovakia

**Coordinating Investigators:**

<None Entered>

| **Center** | **Principal Investigator** | **Co-Investigator(s)** | **Sub-Investigator(s)** | **Address(es)** | **Institutional Review Board or Ethics Committee Address(es)** |
| --- | --- | --- | --- | --- | --- |
|  |  |  |  |  |  |
| 1075 | Dr. Jozef Lukac |  | Dr. Olga Lukacova | Narodny ustav reumatickych chorob  ul.Ivana Krasku 4  Piestany, 921 01  SLOVAKIA | Eticka komisia pri Narodnom ustave reumatickych chorob  ul.I.Krasku 4  Piestany, 921 01  SLOVAKIA |
|  |  |  |  |  |  |
| 1078 | Pavol Polak |  |  | Nestatna reumatologicka ambulancia, NsP Zilina  ul. Vojtecha Spanyola 43  Zilina, 012 07  SLOVAKIA | Eticka komisia pri Zilinskom samospravnom kraji  Zilinsky samospravny kraj, odbor zdravotnictva  Komenskeho ul.48  Zilina, 011 09  SLOVAKIA |
|  |  |  |  |  |  |
| 1080 | Dr. Ivana Revayova |  |  | Reumatologicka ambulancia  Pribinova 25  Bratislava, 81109  SLOVAKIA | Eticka komisia Novapharm, s.r.o.  Zeleznicna nemocnica a poliklinika  Sancova 110  Bratislava 3, 832 99  SLOVAKIA |

## Spain

**Coordinating Investigators:**

<None Entered>

| **Center** | **Principal Investigator** | **Co-Investigator(s)** | **Sub-Investigator(s)** | **Address(es)** | **Institutional Review Board or Ethics Committee Address(es)** |
| --- | --- | --- | --- | --- | --- |
|  |  |  |  |  |  |
| 1035 | Dr. Jesus Tornero Molina |  | Rosa del Castillo  Manuel Fernandez Prada  Pilar Muñoz  Dr. Javier Vidal Fuentes | HOSPITAL GENERAL UNIVERSITARIO DE GUADALAJARA  SERVICIO DE REUMATOLOGIA  C/DONANTES DE SANGRE S/N  GUADALAJARA, GUADALAJARA 19002  SPAIN | Comite Autonomico de Ensayos Clinicos de Andalucia  ETHICS COMMITTEE OF CLINIC INVESTIGATION  CONSEJERIA DE SALUD  AVDA. INNOVACION S/N  EDIFICIO ARENA 1  SEVILLA, SEVILLA 41020  SPAIN |
|  |  |  |  |  |  |
| 1037 | Dr. Juan Gomez Reino |  | Juan Garcia Meijide  Myriam Liz  Alejandra Rama Serans | HOSPITAL CLINICO UNIVERSITARIO DE SANTIAGO  SERVICIO DE REUMATOLOGIA  Pº DE LA CHOUPANA S/N  SANTIAGO DE COMPOSTELA, A CORUÑA 15706  SPAIN | Comite Autonomico de Ensayos Clinicos de Andalucia  ETHICS COMMITTEE OF CLINIC INVESTIGATION  CONSEJERIA DE SALUD  AVDA. INNOVACION S/N  EDIFICIO ARENA 1  SEVILLA, SEVILLA 41020  SPAIN |
|  |  |  |  |  |  |
| 1039 | Dr. EMILIO MARTIN MOLA |  | ALEJANDRO BALSA CRIADO  Dr. Miguel Bernad Pineda  Dr. Carlos Perez De Ayala | HOSPITAL UNIVERSITARIO LA PAZ  SERVICIO DE REUMATOLOGIA  Pº DE LA CASTELLANA, 261  MADRID, MADRID 28046  SPAIN | Comite Autonomico de Ensayos Clinicos de Andalucia  ETHICS COMMITTEE OF CLINIC INVESTIGATION  CONSEJERIA DE SALUD  AVDA. INNOVACION S/N  EDIFICIO ARENA 1  SEVILLA, SEVILLA 41020  SPAIN |
|  |  |  |  |  |  |
| 1040 | Dr. Juan Sanchez Burson |  | Natalia Cid Boza  Paz Gonzalez Moreno  Raquel Hernandez Sanchez | HOSPITAL NUESTRA SEÑORA DE VALME  SERVICIO DE REUMATOLOGIA  CTRA. CADIZ-BELLAVISTA KM. 548,9  SEVILLA, SEVILLA 41014  SPAIN | Comite Autonomico de Ensayos Clinicos de Andalucia  ETHICS COMMITTEE OF CLINIC INVESTIGATION  CONSEJERIA DE SALUD  AVDA. INNOVACION S/N  EDIFICIO ARENA 1  SEVILLA, SEVILLA 41020  SPAIN |

## Sweden

**Coordinating Investigators:**

<None Entered>

| **Center** | **Principal Investigator** | **Co-Investigator(s)** | **Sub-Investigator(s)** | **Address(es)** | **Institutional Review Board or Ethics Committee Address(es)** |
| --- | --- | --- | --- | --- | --- |
|  |  |  |  |  |  |
| 1081 | Dr. Soren Transo |  | Dr. Britt-Mari Wigert | Lanssjukhuset Ryhov, Ortoped- och reumatologkliniken  Jonkoping, 551 85  SWEDEN | Regionala Etikprovningsnamnden i Linkoping  c/o Halsouniversitetets kansli, Linkopings Universitet  Linkoping, 581 83  SWEDEN |
|  |  |  |  |  |  |
| 1082 | Dr. Solbritt Rantapaa-Dahlqvist |  | Dr. Gerd-Marie Alenius  Dr. Stefan Engstrand | Norrlands Universitetssjukhus/Reumatologiska kliniken  Umea, 901 85  SWEDEN | Regionala Etikprovningsnamnden i Linkoping  c/o Halsouniversitetets kansli, Linkopings Universitet  Linkoping, 581 83  SWEDEN |

## Turkey

**Coordinating Investigators:**

<None Entered>

| **Center** | **Principal Investigator** | **Co-Investigator(s)** | **Sub-Investigator(s)** | **Address(es)** | **Institutional Review Board or Ethics Committee Address(es)** |
| --- | --- | --- | --- | --- | --- |
|  |  |  |  |  |  |
| 1049 | Prof. Dr. Vedat Hamuryudan |  | Dr. Gulen Hatemi  Assoc. Prof. Dr. Melike Melikoglu  Prof. Dr. Oner Suzer | Istanbul University Cerrahpasa Medical Faculty  Internal Diseases Department Rheumatology Division  Kocamustafapasa  Istanbul, 34098  TURKEY | Istanbul University Cerrahpasa Medical Faculty Ethics Committee  Istanbul University Cerrahpasa Medical Faculty Ethics Committee  Kocamustafapasa  Istanbul, 34098  TURKEY  Turkish Ministry of Health Central Ethics Committee  T.R. Ministry of Health Pharmaceutical General Directorate  Central Ethics Committee / Regulatory Authority  Cankiri Cad. 57  Diskapi ¿ Ulus  ANKARA , 06060  TURKEY |
|  |  |  |  |  |  |
| 1050 | Prof. Dr. Nurullah Akkoc |  | Assoc. Prof. Dr. Servet Akar  Assoc. Prof. Dr. Ahmet Merih Birlik  Prof. Dr. Sedef Gidener | 9 Eylul University Faculty of Medicine  Department of Rheumotology  Alsancak  Izmir, 35340  TURKEY | Dokuz Eylul University Medical Faculty Ethics Committee  Dokuz Eylul University Medical Faculty Ethics Committee  Inciralti  Izmir, 35340  TURKEY  Turkish Ministry of Health Central Ethics Committee  T.R. Ministry of Health Pharmaceutical General Directorate  Central Ethics Committee / Regulatory Authority  Cankiri Cad. 57  Diskapi ¿ Ulus  ANKARA , 06060  TURKEY |
|  |  |  |  |  |  |
| 1051 | Prof. Dr. Gokhan Keser |  | Assoc. Prof. Dr. Kenan Aksu  Prof. Dr. Isik Tuglular | Ege University Faculty of Medicine  Department of Rheumotology  Izmir, 35100  TURKEY | Ege University Medical Faculty Ethics Committee  Ege University Medical Faculty Ethics Committee  Bornova  Izmir, 35100  TURKEY  Turkish Ministry of Health Central Ethics Committee  T.R. Ministry of Health Pharmaceutical General Directorate  Central Ethics Committee / Regulatory Authority  Cankiri Cad. 57  Diskapi ¿ Ulus  ANKARA , 06060  TURKEY |
|  |  |  |  |  |  |
| 1052 | Prof. Dr. Sedat Kiraz |  | Assoc. Prof. Dr. Sule Apras Bilgen  Prof. Dr. Alper Iskit  Spec. Dr. Omer Karadag | Hacettepe University Faculty of Medicine  Department of Rheumotology  Sihhiye  Ankara, 06100  TURKEY | Hacettepe University Medical Faculty Ethics Committee  Hacettepe University Medical Faculty Ethics Committee  Sihhiye  Ankara, 06100  TURKEY  Turkish Ministry of Health Central Ethics Committee  T.R. Ministry of Health Pharmaceutical General Directorate  Central Ethics Committee / Regulatory Authority  Cankiri Cad. 57  Diskapi ¿ Ulus  ANKARA , 06060  TURKEY |

## United States

**Coordinating Investigators:**

<None Entered>

| **Center** | **Principal Investigator** | **Co-Investigator(s)** | **Sub-Investigator(s)** | **Address(es)** | **Institutional Review Board or Ethics Committee Address(es)** |
| --- | --- | --- | --- | --- | --- |
|  |  |  |  |  |  |
| 1005 * | Dr. Stuart S. Kassan |  | Dr. Judy Weiss | Colorado Arthritis Associates  Suite 314  4200 West Conejos Place  Denver, CO 80204  UNITED STATES | Quorum Institutional Review Board  Suite 1000  1601 Fifth Avenue  Seattle, WA 98101  UNITED STATES |
|  |  |  |  |  |  |
| 1006 | Dr. Frederick Dietz |  | Dr. Robin Renee Hovis  Dr. Frank A. Ventimiglia | Rockford Health Physicians  2300 North Rockton Avenue  Rockford, IL 61103-3692  UNITED STATES | Quorum Institutional Review Board  Suite 1000  1601 Fifth Avenue  Seattle, WA 98101  UNITED STATES |
|  |  |  |  |  |  |
| 1007 | Dr. Sanford Mayer Wolfe |  |  | STAT Research, Inc.  Suite 544  111 West First Street  Dayton, OH 45402  UNITED STATES | Quorum Institutional Review Board  Suite 1000  1601 Fifth Avenue  Seattle, WA 98101  UNITED STATES |
|  |  |  |  |  |  |
| 1008 | Dr. Melody St. John |  | Laura Larrison  Dr. James W. Logan | Heritage Physician Group  100 McGowan Court  Hot Springs, AR 71913  UNITED STATES | Quorum Institutional Review Board  Suite 1000  1601 Fifth Avenue  Seattle, WA 98101  UNITED STATES |
|  |  |  |  |  |  |
| 1009 | Dr. Jeffrey Edward Poiley |  |  | Jeffrey E. Poiley MD, PA  324 East Par Avenue  Orlando, FL 32804  UNITED STATES | Quorum Institutional Review Board  Suite 1000  1601 Fifth Avenue  Seattle, WA 98101  UNITED STATES |
|  |  |  |  |  |  |
| 1010 | Dr. Stephen Allan Bookbinder |  | Dr. Arthur Elkins | Ocala Rheumatology Research Center  Suite 102  3210 Southwest 33rd Road  Ocala, FL 34474  UNITED STATES | Quorum Institutional Review Board  Suite 1000  1601 Fifth Avenue  Seattle, WA 98101  UNITED STATES |
|  |  |  |  |  |  |
| 1011 | Dr. Stanley Bruce Cohen |  | Jean A. Clark  Dr. Roy Mitchell Fleischmann  Dr. Thomas David Geppert  Dr. Imran Iqbal  Dr. Robert Neil Jenkins  Dr. Sharad Lakhanpal  Dr. Richard L. Stern  Dr. Jack Bernstein Vine  Andrea S. Wheeler | Metroplex Clinical Research Centre - Dallas  Suite 441  5939 Harry Hines Boulevard  Dallas, TX 75235  UNITED STATES | Quorum Institutional Review Board  Suite 1000  1601 Fifth Avenue  Seattle, WA 98101  UNITED STATES |
|  |  |  |  |  |  |
| 1014 | Dr. Mark William Niemer |  | Mr. George J. Casey  Dr. Steven Rock | Medical Associates Clinic  1500 Associates Drive  Dubuque, IA 52002  UNITED STATES | Quorum Institutional Review Board  Suite 1000  1601 Fifth Avenue  Seattle, WA 98101  UNITED STATES |
|  |  |  |  |  |  |
| 1015 | Dr. Joel Charles Silverfield |  | Dr. Michael Claude Burnette  Dr. Harris Hugh McIlwain  Dr. Kimberly McIlwain Smith | Tampa Medical Group, PA  Suite 303  4700 North Habana Avenue  Tampa, FL 33614  UNITED STATES | Quorum Institutional Review Board  Suite 1000  1601 Fifth Avenue  Seattle, WA 98101  UNITED STATES |
|  |  |  |  |  |  |
| 1018 | Dr. Edward Joel Fudman |  | Dr. Stephanie Ann Booth  Dr. Brian Sam Sayers | Austin Rheumatology Research  Suite 110  1301 West 38th Street  Austin, TX 78705  UNITED STATES  Austin Rheumatology Research  Suite 702  1301 West 38th Street  Austin, TX 78705  UNITED STATES | Quorum Institutional Review Board  Suite 1000  1601 Fifth Avenue  Seattle, WA 98101  UNITED STATES |
|  |  |  |  |  |  |
| 1020 | Dr. Robert Michael Griffin Jr. |  | Dr. Michael Allen Borofsky  Brent William Calhoon  Dr. Peter Daniel Nicholas Jr.  Dr. Jerome Stephen Weisberg  Dr. Brian Del Vecchio | Clinical Research Center of Reading LLP  401 Buttonwood Street  West Reading, PA 19611-1124  UNITED STATES | Quorum Institutional Review Board  Suite 1000  1601 Fifth Avenue  Seattle, WA 98101  UNITED STATES |
|  |  |  |  |  |  |
| 1022 | Antony Hou |  | Tina Escobedo  Dr. Eric C. Lee  Dr. Mohamed Bassam Sebai  Ms. Patricia E. DesLauriers | Boling Clinical Trails (BCT)  Suite 302  510 North 13th Avenue  Upland, CA 91786  UNITED STATES  Inland Rheumatology and Osteoporosis Medical Group  Suite 306  548 North 13th Avenue  Upland, CA 91786  UNITED STATES | Quorum Institutional Review Board  Suite 1000  1601 Fifth Avenue  Seattle, WA 98101  UNITED STATES |
|  |  |  |  |  |  |
| 1023 | Dr. Robert Emil Ettlinger |  | Dr. George Howard Krick  Dr. Neil F. Moody Jr.  Teresa A. Unkrur | Tacoma Center for Arthritis Research, PS  Suite 201  1901 South Cedar Street  Tacoma, WA 98405  UNITED STATES  Tacoma Center for Arthritis Research, PS  Suite 204  1901 South Cedar Street  Tacoma, WA 98405-2308  UNITED STATES | Quorum Institutional Review Board  Suite 1000  1601 Fifth Avenue  Seattle, WA 98101  UNITED STATES |
|  |  |  |  |  |  |
| 1024 | Dr. Joel Marc Kremer |  | Christine Barr  Justine Feder-Lailer  Dr. Neal Steven Greenstein  Dr. Dorota Hausner-Sypek  Mari Kaymakcian  Teresa Michaels  Victoria Michaels  Dr. Norman Reid Romanoff  Jennifer Schreiner  Dr. Lee Shapiro  Ludovico Frank Cavaliere  Haley Garrett | Center for Rheumatology  Suite 101  1367 Washington Avenue  Albany, NY 12206-1043  UNITED STATES | Quorum Institutional Review Board  Suite 1000  1601 Fifth Avenue  Seattle, WA 98101  UNITED STATES |
|  |  |  |  |  |  |
| 1025 * | Dr. Robert A. Kimelheim |  | Dr. Mohan Gurubhagavatula | Arthritis and Rheumatic Disease Associates  Suite 24  8815 Germantown Avenue  Philladelphia, PA 19118  UNITED STATES | Quorum Institutional Review Board  Suite 1000  1601 Fifth Avenue  Seattle, WA 98101  UNITED STATES |
|  |  |  |  |  |  |
| 1028 | Dr. Richard Roy Olson |  | Dr. David James Dansdill  Tami Kucia | Rockford Orthopedic Associates  324 Roxbury Road  Rockford, IL 61107  UNITED STATES | Quorum Institutional Review Board  Suite 1000  1601 Fifth Avenue  Seattle, WA 98101  UNITED STATES |
|  |  |  |  |  |  |
| 1029 | Dr. Carol Lynn Danning |  | Dr. Janet Bahr  Sharon Barnhart  Nancy Davidson  Dr. Jack Lockhart  Diane Webster  Dr. Guy Peter Fiocco  Dr. Peter Arndt Valen | Gundersen Clinic, Ltd  Mail Stop NCI-005  3111 Gundersen Drive  Onalaska, WI 54650  UNITED STATES | Gundersen Clinic, Ltd Human Subjects Committee  1836 South Avenue  La Crosse, WI 54601  UNITED STATES |
|  |  |  |  |  |  |
| 1031 | Pietro Rocca |  |  | Delaware Arthritis and Osteoporosis Center  Suite 101  537 Stanton-Christiana Road  Newark, DE 19713  UNITED STATES | Quorum Institutional Review Board  Suite 1000  1601 Fifth Avenue  Seattle, WA 98101  UNITED STATES |
|  |  |  |  |  |  |
| 1032 | Dr. Geneva Louise Hill |  | Dr. Josette J. Johnson  Dr. Jeffrey Geldert Lawson | Piedmont Arthritis Clinic, PA  Suite 400  3 St. Francis Drive  Greenville, SC 29601  UNITED STATES | Quorum Institutional Review Board  Suite 1000  1601 Fifth Avenue  Seattle, WA 98101  UNITED STATES |
|  |  |  |  |  |  |
| 1033 | Dr. Nathan Wei |  |  | The Arthritis and Osteoporosis Center of Maryland  71 Thomas Johnson Drive  Frederick, MD 21702  UNITED STATES | Quorum Institutional Review Board  Suite 1000  1601 Fifth Avenue  Seattle, WA 98101  UNITED STATES |
|  |  |  |  |  |  |
| 1041 | Jane H. Box |  | Dr. John Franklyn Babich  Dr. Patrick N. Box  Dr. Ashrito Kumar Dayal  Dr. John Franklyn Babich  Dr. Patrick N. Box  Dr. Ashrito Kumar Dayal | Arthritis Clinic & Carolina Bone & Joint, PA  10460 Park Road  Charlotte, NC 28210  UNITED STATES | Quorum Institutional Review Board  Suite 1000  1601 Fifth Avenue  Seattle, WA 98101  UNITED STATES |
|  |  |  |  |  |  |
| 1090 | Dr. Paul A. Dura |  | Kristin J. Contro  Aspen L. D'Angelo  Dr. Thomas Joseph Oven  Susan Wenzinger  Jennifer L. Bubel | Regional Rheumatology Associates  Suite 302  161 Riverside Drive  Binghamton, NY 13905  UNITED STATES | Quorum Institutional Review Board  Suite 1000  1601 Fifth Avenue  Seattle, WA 98101  UNITED STATES |
|  |  |  |  |  |  |
| 1092 | Dr. Philip Judson Mease |  | Ms. Nicole M. Furfaro  Lyne Schaefer-Alfonse  Sue Williams-Judge  Kori A. Dewing | Investigational Drug Service  747 Broadway  Seattle, WA 98122  UNITED STATES  Seattle Rheumatology Associates  Suite 1000  1101 Madison  Seattle, WA 98104  UNITED STATES  Swedish Medical Center  747 Broadway  Seattle, WA 98122  UNITED STATES | Western Institutional Review Board  3535 Seventh Avenue, SW  Olympia, WA 98502  UNITED STATES |
|  |  |  |  |  |  |
| 1111 * | Dr. David Hilton Sikes |  | Dr. Mark Sol Eisner  Nathan A. Meyer  Dr. Amarilis Torres | Florida Medical Clinic  38135 Market Square  Zephyrhills, FL 33540  UNITED STATES | Quorum IRB  Suite 1000  1601 Fifth Avenue,  Seattle, WA 98101  UNITED STATES |
|  |  |  |  |  |  |
| 1112 | Dr. Atul K. Singhal |  | Ms. Julia A. Dilliard  Ms. Doris C. Harvey  Dr. Guillermo A. Quiceno | SouthWest Rheumatology, PA  Suite 614  8230 Walnut Hill Lane  Dallas, TX 75231  UNITED STATES  SouthWest Rheumatology, PA  Suite 615  18601 LBJ Freeway  Mesquite, TX 75150  UNITED STATES | Quorum IRB  Suite 1000  1601 Fifth Avenue,  Seattle, WA 98101  UNITED STATES |
|  |  |  |  |  |  |
| 1115 | Dr. Debra R. Michel |  | Dr. Santina Carminati Taddei | East Valley Rheumatology and Osteoporosis, PC  Suite 108  3921 East Baseline Road  Gilbert, AZ 85234  UNITED STATES | Quorum Institutional Review Board  Suite 1000  1601 Fifth Avenue  Seattle, WA 98101  UNITED STATES |
|  |  |  |  |  |  |
| 1116 | Dr. Sanjiv Kapil |  | Dr. Harry Nyanteh | Florida Arthritis Center, P.L.  Suite 1005  147 Parliament Loop  Lake Mary, FL 32746  UNITED STATES  Physicians Research Alliance  Suite 102A  609 North Charles Richard Beall Boulevard  Debary, FL 32713  UNITED STATES | Quorum Institutional Review Board  Suite 1000  1601 Fifth Avenue  Seattle, WA 98101  UNITED STATES |
|  |  |  |  |  |  |
| 1117 | Dr. Kyle Woodrow Strader |  | Dr. Douglas Garland Freeman Jr.  Dr. Louie E. Tsiktsiris | North Carolina Arthritis and Allergy Care Center  3831 Merton Drive  Raleigh, NC 27609  UNITED STATES | Quorum Institutional Review Board  Suite 1000  1601 Fifth Avenue  Seattle, WA 98101  UNITED STATES |
