# Supplementary material for: Changes in serum creatinine in patients with active rheumatoid arthritis treated with tofacitinib: results from clinical trials
Source: Arthritis Res Ther. 2014 Jul 25;16(4):R158. doi: 10.1186/ar4673 (PMC4220634; doi:10.1186/ar4673)
Supplement: Supplementary file 4 — Additional file 4: List of Investigators and Corresponding Ethics Committees or Institutional Review Boards for the Phase 3 A3921032 study. (DOC 320 KB) [file 13075_2013_4378_MOESM4_ESM.doc]

# 16.1.4 LIST OF INVESTIGATORS AND CORRESPONDING ETHICS COMMITTEES OR INSTITUTIONAL REVIEW BOARDS

## Australia

**Coordinating Investigators:**

<None Entered>

| **Center** | **Principal Investigator** | **Co-Investigator(s)** | **Sub-Investigator(s)** | **Address(es)** | **Institutional Review Board or Ethics Committee Address(es)** |
| --- | --- | --- | --- | --- | --- |
|  |  |  |  |  |  |
| 1106 | Dr. Paul A. Bird |  | Mary Clarke  Dr. Fredrick F. Joshua  Dr. Louis E. McGuigan  Dr. Ian J. Portek  Dr. Rajapakse Lalith Ratnayake | Combined Rheumatolgoy Practice (CRP)  Suite 4, Level 1  19 Kensington Street  Kogarah, NSW 2217  AUSTRALIA | Bellberry Ltd Human Research Ethics Committee  229 Greenhill Road  Dulwich, SA 5065  AUSTRALIA |
|  |  |  |  |  |  |
| 1108 | A/Prof. Russell R. Buchanan |  | Lorrane Gatt  Dr. Kim Le Marshall  Dr. John H. Moi | Austin Health - Heidelberg Repatriation Hospital  300 Waterdale Road  Heidelberg, VIC 3081  AUSTRALIA | Austin Health Human Research Ethics Committee  Henry Buck Building  Austin Hospital  145 Studley Road  Heidelberg, VIC 3084  AUSTRALIA |
|  |  |  |  |  |  |
| 1112 * | Dr. Michael Ahern |  | Malcolm D. Smith  Mihir Wechalekar | Repatriation General Hospital  Daws Rd  Daw Park, SA 5041  AUSTRALIA | Flinders Clinical Research Ethics Committee  Flinders Clinical Research Ethics Committee (FCREC)  Room 2A 221  Flinders Medical Centre  Bedford Park  South Australia, 5042  AUSTRALIA |
|  |  |  |  |  |  |

## Austria

**Coordinating Investigators:**

<None Entered>

| **Center** | **Principal Investigator** | **Co-Investigator(s)** | **Sub-Investigator(s)** | **Address(es)** | **Institutional Review Board or Ethics Committee Address(es)** |
| --- | --- | --- | --- | --- | --- |
|  |  |  |  |  |  |
| 1092 | Dr. Omid Zamani |  | Elke Böttcher  Dr. Joerg Dietmar Rieger | Rheuma Zentrum Favoriten  Quellenstrasse 181  Wien, A-1100  AUSTRIA | Ethikkommission der Stadt Wien  TownTown  Thomas-Klestil-Platz 8  Wien, A-1030  AUSTRIA |
|  |  |  |  |  |  |
| 1113 | Univ. Prof. Dr. Clemens Scheinecker |  | Dr. Michael Markus Bonelli  Prof. Dr. Klaus Peter Machold | Allgemeines Krankenhaus-Universitatskliniken  Wahringer Gurtel 18-20  Wien,  AUSTRIA  Universitätsklinik für Innere Medizin III  Abtleilung für Rheumatologie  AKH-Wien  Währinger Gürtel 18-20  Wien, 1090  AUSTRIA | Ethikkommission der Stadt Wien  TownTown  Thomas-Klestil-Platz 8  Wien, A-1030  AUSTRIA |
|  |  |  |  |  |  |
| 1117 | Prim. Univ. Doz. Dr. Ludwig Erlacher |  | Dr. Sonja Brandstaetter  Dr. Sonja Brandstaetter  Dr. Angelika Kraus  Dr. Monika Mustak-Blagusz | SMZ Sud Kaiser Franz Josef Spital  Kundratstrasse 3  Wien, 1100  AUSTRIA | Ethikkommission der Stadt Wien  TownTown  Thomas-Klestil-Platz 8  Wien, A-1030  AUSTRIA |
|  |  |  |  |  |  |

## Belgium

**Coordinating Investigators:**

<None Entered>

| **Center** | **Principal Investigator** | **Co-Investigator(s)** | **Sub-Investigator(s)** | **Address(es)** | **Institutional Review Board or Ethics Committee Address(es)** |
| --- | --- | --- | --- | --- | --- |
|  |  |  |  |  |  |
| 1094 * | Prof. Piet P. M. M. Geusens |  | Dr. Hubert Berghs  Dr. Marleen Coppens  Dr. Anne Sileghem  Dr. Johan L.M. Vanhoof  Dr. Pascale Volders | Biomedical Research Institute/ Department of Rheumatology  Bretheistraat 149  Genk, 3600  BELGIUM | Ethisch Comité  AZ Groeninge  Burgemeester Vercruysselaan 5  Kortrijk, 8500  BELGIUM |
|  |  |  |  |  |  |
| 1098 | Dr. Klaas Vandevyvere |  | Dr. Anne Durnez | AZ Groeninge Campus Sint Maarten  Burgemeester Vercruysselaan 5  Kortrijk,  BELGIUM | Ethisch Comite  Burgemeester Vercruysselaan 5  Kortrijk, 8500  BELGIUM |
|  |  |  |  |  |  |
| 1102 | Dr. Jan L. A. Lenaerts (Previous PI)  Dr. Luk Corluy |  | Dr. Christine Langenaken  Dr Jan Lenaerts  Dr. Paul Van Wanghe | Reuma Instituut Hasselt  Anne Frankplein 17  Hasselt, 3500  BELGIUM | Ethisch Comite  Burgemeester Vercruysselaan 5  Kortrijk, 8500  BELGIUM |
|  |  |  |  |  |  |
| 1123 | Prof. Jean-Yves Reginster |  | Dr. Angela Kvasz  Dr. Marie-Paule Lecart  Dr. Nathalie Sarlet | Centre Hospitalier Universitaire de Liège  Unité d'Exploration de l'Os et du Cartilage  CHU Centre Ville, Brulle (9th floor)  Quai Godefroid Kurth 45  Liège, 4020  BELGIUM | Ethisch Comite  Burgemeester Vercruysselaan 5  Kortrijk, 8500  BELGIUM |
|  |  |  |  |  |  |

## Brazil

**Coordinating Investigators:**

<None Entered>

| **Center** | **Principal Investigator** | **Co-Investigator(s)** | **Sub-Investigator(s)** | **Address(es)** | **Institutional Review Board or Ethics Committee Address(es)** |
| --- | --- | --- | --- | --- | --- |
|  |  |  |  |  |  |
| 1090 | Dr. Sebastiao C. Radominski |  | Dr. Vivian B. Coginotti  Dr. Sinara da Silva Freitas  Dr. Andreas Funke  Dr. Lucila Stange Rezende  Dr. David Cezar Titton | Centro de Estudos em Terapias Inovadoras  Rua Padre Camargo, 241  Alto da Gloria  Curitiba, PR 80060-240  BRAZIL | Comitê de Ética em Pesquisa em Seres Humanos do Hospital das Clínicas da UFPR  Rua General Carneiro 181  Curitiba, PR 80060-900  BRAZIL |
|  |  |  |  |  |  |
| 1091 | Dr. Antonio Carlos Ximenes |  | Dr. Rafael Navarrete Fernandez  Dr. Fabia M.G.P. Oliveira  Dr. Marcelo Pimenta  Dr. Bruno Nazeozeno Ribeiro | CIP - Centro Internacional de Pesquisas  Rua 9 B, 129 - 3 andar  Setor Oeste  Goiania, GO 74110-120  BRAZIL | Comite de Etica em Pesquisa do Hospital Geral de Goiania - CEPHGG  Hospital Geral de Goiania Dr. Alberto Rassi  Av. Anhanguera, 6479  Setor Oeste  Goiania, GO 74110-010  BRAZIL |
|  |  |  |  |  |  |
| 1130 | Dr. Mauro W. Keiserman |  | Melissa Claudia Bisi  Dr. Briele Keiserman  Dr. Tatiana Karenini Muller  Dr. Aline Defaveri do Prado | Hospital Sao Lucas da PUCRS  Servico de Reumatologia  Av. Ipiranga, 6690 - Conj. 220- 2 andar  Jardim Botanico  Porto Alegre, RS 90610-000  BRAZIL  Hospital Sao Lucas da PUCRS  Centro de Pesquisa Clinica  Avenida Ipiranga, 6690 - 4 andar  Porto Alegre, RS 90610-000  BRAZIL | Comite de Etica em Pesquisa da Pontificia Universidade Catolica do Rio Grande do Sul  Av. ipiranga 6690-Conj.314 -3 andar  Jardim Botanico  Porto Alegre, RS 90610-000  BRAZIL |
|  |  |  |  |  |  |
| 1137 | Dr. Cristiano Augusto de Freitas Zerbini |  | Dr. Marta E. C. Bastos  Lina Oliveira de Carvalho  Dr. Wagner Ikehara  Dr. Luiz Carlos Latorre  Dr. Andrea Barranjard Vannucci Lomonte  Dr. Silvia Caroline Santana Moura  Dr. Maria Jose Nunes  Dr. Raissa Gomes da Silva  Dr. Mariana G. Waisberg | CEPIC - Centro Paulista de Investigacao Clinica e Servicos Medicos Ltda  Rua Moreira e Costa 342  Sao Paulo, SP 04266-010  BRAZIL | Comite de Etica em Pesquisa do Complexo Hospitalar Heliopolis  Rua Conego Xavier, 276 - 10º andar  Sacoma  Sao Paulo, SP 04231-030  BRAZIL |
|  |  |  |  |  |  |

## Canada

**Coordinating Investigators:**

<None Entered>

| **Center** | **Principal Investigator** | **Co-Investigator(s)** | **Sub-Investigator(s)** | **Address(es)** | **Institutional Review Board or Ethics Committee Address(es)** |
| --- | --- | --- | --- | --- | --- |
|  |  |  |  |  |  |
| 1003 | Dr. Alfred Augusto Cividino |  | Dr. Raja Bobba  Dr. Pauline Boulos  Louise Sloat | MAC Research Inc.  187 Hughson Street South  Hamilton, ON L8N 2B6  CANADA | IRB Services  Suite 300  372 Hollandview Trail  Aurora, ON L4G 0A5  CANADA |
|  |  |  |  |  |  |
| 1007 | Dr. Timothy G. McCarthy |  | Dr. Cory Baillie  Janna Tweed | Manitoba Clinic  790 Sherbrook Street  Winnipeg, MB R3A 1M3  CANADA | IRB Services  Suite 300  372 Hollandview Trail  Aurora, ON L4G 0A5  CANADA |
|  |  |  |  |  |  |
| 1008 | Dr. Robert J. McKendry |  | Dr. Gunnar R. Kraag  Ms. Paula Patterson | Rheumatology Research Associates  Suite 412  1919 Riverside Drive  Ottawa, ON K1H 1A2  CANADA | IRB Services  Suite 300  372 Hollandview Trail  Aurora, ON L4G 0A5  CANADA |
|  |  |  |  |  |  |
| 1026 | Dr. William G. Bensen |  | Dr. Fernando Anthony Bianchi  Ms. Melissa Deamude | Dr. William G. Bensen Medicine Professional Corporation  Suite 203  25 Charlton Avenue East  Hamilton, ON L8N 1Y2  CANADA  Office of Dr. Fernando Bianchi  Suite 304  25 Chartlon Avenue East  Hamilton, ON L8N 1Y2  CANADA | IRB Services  Suite 300  372 Hollandview Trail  Aurora, ON L4G 0A5  CANADA |
|  |  |  |  |  |  |
| 1029 | Dr. Mary Ann Fitzcharles |  | Dr. Martin A. Cohen  Louisa Da Silva  Mrs. Grace Lavigne  Dr. Michael R. Starr | West Island Rheumatology Research Associates  Suite 209  269 St. Jean Boulevard  Pointe Claire, QC H9R 3J1  CANADA | IRB Services  Suite 300  372 Hollandview Trail  Aurora, ON L4G 0A5  CANADA |
|  |  |  |  |  |  |
| 1030 | Dr. Boulos Haraoui |  | Dr. Denis Choquette  Raymonde Gregoire  Jolaine L'Archevesque  Dr. Jean-Pierre Raynauld | Institut de Rhumatologie de Montreal  1551 Ontario Est  Montreal, QC H2L 1S6  CANADA | IRB Services  Suite 300  372 Hollandview Trail  Aurora, ON L4G 0A5  CANADA |
|  |  |  |  |  |  |

## France

**Coordinating Investigators:**

<None Entered>

| **Center** | **Principal Investigator** | **Co-Investigator(s)** | **Sub-Investigator(s)** | **Address(es)** | **Institutional Review Board or Ethics Committee Address(es)** |
| --- | --- | --- | --- | --- | --- |
|  |  |  |  |  |  |
| 1095 | Pr. Francis Berenbaum |  | Julien Champey  Mathilde Michon  Jeremie Sellam | Hôpital Saint-Antoine  184 rue du Faubourg Saint-Antoine  Paris,  FRANCE | Comité de Protection des Personnes - CPP ILE-DE-France V  Hôpital Saint-Antoine  184 rue du Faubourg Saint-Antoine  75012 PARIS,  FRANCE |
|  |  |  |  |  |  |
| 1101 | Roland Chapurlat |  | Dr. Elisabeth Confavreux  Dr. Florence Duvert | Hopital Edouard Herriot  Service de Rheumatologie  5 place d'Arsonval  Lyon,  FRANCE | Comité de Protection des Personnes - CPP ILE-DE-France V  Hôpital Saint-Antoine  184 rue du Faubourg Saint-Antoine  75012 PARIS,  FRANCE |
|  |  |  |  |  |  |
| 1107 | Dr. Claude-Laurent Benhamou |  | Anca Adriana Corondan  Eric Lespessailles  Sylvie Loiseau-Peres | Centre Hospitalier Régional d'Orléans Hôpital Porte Madeleine  IPROS  1 rue Porte Madeleine  Orleans, 45000  FRANCE  HOPITAL PORTE MADELEINE  Secteur de Rhumatologie - BP 2439  1 rue Porte Madeleine  ORLEANS CEDEX 1, 45032  FRANCE | Comité de Protection des Personnes - CPP ILE-DE-France V  Hôpital Saint-Antoine  184 rue du Faubourg Saint-Antoine  75012 PARIS,  FRANCE |
|  |  |  |  |  |  |
| 1120 | Prof. Patrice Fardellone |  | Franck Grados  Dr. Anissa Hacene  Isabelle Henry  Dr. Florence Millot  Thomas Ryckelynck | CHU Amiens, Service de Rhumatologie  1 Place Victor Pauchet  Amiens, 80054  FRANCE | Comité de Protection des Personnes - CPP ILE-DE-France V  Hôpital Saint-Antoine  184 rue du Faubourg Saint-Antoine  75012 PARIS,  FRANCE |
|  |  |  |  |  |  |
| 1134 | Dr Minh Nguyen |  | Pr. Maxime Dougados  Christophe Hudry  Dr. Sami Kolta  Ms Catherine Le Bourlout | Hopital Cochin  Rhumatologie - Hardy B4  27, rue du Faubourg Saint-Jacques  Paris, 75014  FRANCE |  |
|  |  |  |  |  |  |

## Germany

**Coordinating Investigators:**

Prof. Dr. med. Gerd-Ruediger Burmester

| **Center** | **Principal Investigator** | **Co-Investigator(s)** | **Sub-Investigator(s)** | **Address(es)** | **Institutional Review Board or Ethics Committee Address(es)** |
| --- | --- | --- | --- | --- | --- |
|  |  |  |  |  |  |
| 1064 | Prof. Dr. med. Gerd-Ruediger Burmester |  | Dr. med. Tobias Alexander  Dr. med. Karlfried Aupperle  Dr. med. Mike Oliver Becker  Dr. med. Eugen Feist  Dr. med. Cornelia Spies  Jan Zernicke | Charite Campus Mitte, Medizinische Klinik mit Schwerpunkt Rheumatologie und Klinische Immunologie  Abteilung Neue Therapien  Chariteplatz 1  Berlin, 10117  GERMANY | Landesamt fuer Gesundheit und Soziales (LAGeSo)  Landesamt fuer Gesundheit und Soziales (LaGeSo)  Geschaeftstelle der Ethikkommission Berlin  Fehrbelliner Platz 1  Berlin, 10707  GERMANY |
|  |  |  |  |  |  |
| 1065 | Prof. Dr. med. Harald Burkhardt |  | Dr. med. Frank Behrens  Dr. med. Michaela Koehm  Dr. med. Michaela Koehm  Dr. med. Nicola Reuschling | J.W.-Goethe-Universitaetsklinik, Medizinische Klinik II, Abteilung fuer Rheumatologie  Theodor-Stern-Kai 7  Frankfurt am Main, 60590  GERMANY | Landesamt fuer Gesundheit und Soziales (LAGeSo)  Landesamt fuer Gesundheit und Soziales (LaGeSo)  Geschaeftstelle der Ethikkommission Berlin  Fehrbelliner Platz 1  Berlin, 10707  GERMANY |
|  |  |  |  |  |  |
| 1066 | Dr. med. Juergen Rech |  | Matthias Englbrecht  Stephanie Finzel  Dr. med. Monika Ronneberger  Dr. med. Monika Ronneberger  Jochen Wacker | Studienambulanz, Medizinische Klinik 3, Universitaetsklinikum Erlangen  Ulmenweg 18  Erlangen, 91054  GERMANY | Landesamt fuer Gesundheit und Soziales (LAGeSo)  Landesamt fuer Gesundheit und Soziales (LaGeSo)  Geschaeftstelle der Ethikkommission Berlin  Fehrbelliner Platz 1  Berlin, 10707  GERMANY |
|  |  |  |  |  |  |
| 1067 | Prof. Dr. Hans-Peter Tony |  | Dr. Martin Feuchtenberger  Dr. Stefan Kleinert  Yvonne Kochler  Annette Kuhn  Dr. med. Silke Osiek  Dr. Eva Ostermeier  Gabriele Rothenbucher | Universitaetsklinikum Wuerzburg, Medizinische Klinik II, Rheumatologie/Immunologie, A3.-1.923  Oberduerrbacherstr. 6  Wuerzburg, 97080  GERMANY | Landesamt fuer Gesundheit und Soziales (LAGeSo)  Landesamt fuer Gesundheit und Soziales (LaGeSo)  Geschaeftstelle der Ethikkommission Berlin  Fehrbelliner Platz 1  Berlin, 10707  GERMANY |
|  |  |  |  |  |  |
| 1070 | Dr. Helmut Soerensen |  | Dr. Bianka Andermann  Anna-Elisabeth Thiele | Ambulantes Rheumazentrum Dr. Soerensen  Argentinische Allee 42  Berlin, 14163  GERMANY | Landesamt fuer Gesundheit und Soziales (LAGeSo)  Landesamt fuer Gesundheit und Soziales (LaGeSo)  Geschaeftstelle der Ethikkommission Berlin  Fehrbelliner Platz 1  Berlin, 10707  GERMANY |
|  |  |  |  |  |  |
| 1097 | PD Dr. Andrea Rubbert-Roth |  | Prof. Efim Benenson  Dr. Thomas Rath  Jasemine Saech | Universitaetsklinikum Koeln Klinik I fuer Innere Medizin, Haus 16, 1. OG, Raum 1.008  Kerpener Str. 62  Koeln, 50937  GERMANY | Landesamt fuer Gesundheit und Soziales (LAGeSo)  Landesamt fuer Gesundheit und Soziales (LaGeSo)  Geschaeftstelle der Ethikkommission Berlin  Fehrbelliner Platz 1  Berlin, 10707  GERMANY |
|  |  |  |  |  |  |
| 1124 | Dr. med. Sylke Wagner |  | Dr. med. Thomas Linde | FAE Innere Medizin / Rheumatologie  Ludwig-Wucherer-Str. 10  Halle, 06108  GERMANY | Landesamt fuer Gesundheit und Soziales (LAGeSo)  Landesamt fuer Gesundheit und Soziales (LaGeSo)  Geschaeftstelle der Ethikkommission Berlin  Fehrbelliner Platz 1  Berlin, 10707  GERMANY |
|  |  |  |  |  |  |
| 1126 | Dr. Ulrich Schoo |  | Dr. Georg Huebner | Schwerpunktpraxis fuer Rheumatologie  Sprickmannstr. 36  Rheine, 48431  GERMANY | Landesamt fuer Gesundheit und Soziales (LAGeSo)  Landesamt fuer Gesundheit und Soziales (LaGeSo)  Geschaeftstelle der Ethikkommission Berlin  Fehrbelliner Platz 1  Berlin, 10707  GERMANY |
|  |  |  |  |  |  |
| 1127 | Prof. Dr. med. Juergen Wollenhaupt |  | Dr. med. Andrea Binda  Dr. med. Andrea Everding  Dr. med. Ulrike Schnoor  Dr. med. Wolfgang Winter | Schoen Klinik Hamburg-Eilbek, Abt. Rheumatologie und Klin. Immunologie  Dehnhaide 120  Hamburg, 22081  GERMANY | Landesamt fuer Gesundheit und Soziales (LAGeSo)  Landesamt fuer Gesundheit und Soziales (LaGeSo)  Geschaeftstelle der Ethikkommission Berlin  Fehrbelliner Platz 1  Berlin, 10707  GERMANY |
|  |  |  |  |  |  |
| 1136 | Prof. Dr. med. Ulf Wagner |  | Dr. Sybille Arnold  Prof. Dr. med. Christoph Baerwald  Dr. Andreas Kupka  Dr. Olga Malysheva  Dr. Matthias Pierer  Dr. Susette Unger | Universitaetsklinikum Leipzig AoeR, Department fuer Innere Medizin  Sektion Rheumatologie/Gerontologie, Studienambulanz Rheumatologie  Liebigstr. 20  Leipzig, 04103  GERMANY | Landesamt fuer Gesundheit und Soziales (LAGeSo)  Landesamt fuer Gesundheit und Soziales (LaGeSo)  Geschaeftstelle der Ethikkommission Berlin  Fehrbelliner Platz 1  Berlin, 10707  GERMANY |
|  |  |  |  |  |  |

## Ireland

**Coordinating Investigators:**

<None Entered>

| **Center** | **Principal Investigator** | **Co-Investigator(s)** | **Sub-Investigator(s)** | **Address(es)** | **Institutional Review Board or Ethics Committee Address(es)** |
| --- | --- | --- | --- | --- | --- |
|  |  |  |  |  |  |
| 1118 | Dr. Douglas J. Veale |  | Dr. Oliver Fitzgerald  Leonard Harty  Anne-Barbara Mongey  Ronan Mullen  Aamir Saeed  Agnes Szentpetery | Department of Rheumatology  St. Vincent's University Hospital  Elm Park  Dublin, 4  IRELAND | Clinical Research Ethics Committee of the Cork Teaching Hospitals  Lancaster Hall  6 Little Hanover Street  Cork,  IRELAND |
|  |  |  |  |  |  |
| 1121 * | Alexander Fraser |  | Dr. Mumtaz Khan | St. Nessan¿s Hospital  Croom, Co. Limerick  IRELAND | Clinical Research Ethics Committee of the Cork Teaching Hospitals  Lancaster Hall  6 Little Hanover Street  Cork,  IRELAND |
|  |  |  |  |  |  |

## Italy

**Coordinating Investigators:**

<None Entered>

| **Center** | **Principal Investigator** | **Co-Investigator(s)** | **Sub-Investigator(s)** | **Address(es)** | **Institutional Review Board or Ethics Committee Address(es)** |
| --- | --- | --- | --- | --- | --- |
|  |  |  |  |  |  |
| 1099 | Marco Matucci Cerinic (Previous PI)  Prof. Marco Matucci Cerinic |  | Dr. Francesca Bartoli  Dr. Silvia Bellando Randone  Giulia Carnesecchi  Elena Corsi  Dr. Francesca Nacci  Francesca Peruzzi | Dipartimento di Biomedicina, Azienda Ospedaliera Universitaria Careggi  SOD Reumatolgia, Villa Monna Tessa  Viale Pieraccini 18  Firenze, 50139  ITALY | Comitato Etico per la Sperimentazione Clinica dei Medicinali  dell'Azienda Ospedaliero-Universitaria Careggi di Firenze  C.T.O.  Largo Palagi, 1  Firenze, 50139  ITALY |
|  |  |  |  |  |  |
| 1100 * | Walter Grassi |  | Alarico Ariani  Dr. Patrizia Blasetti  Glauco Gubinelli | Ospedale Carlo Urbani  Clinica Reumatologia  ASUR - Zona Territoriale 5  Via dei Colli 52  Jesi, (AN) 60035  ITALY | Comitato Etico  dell¿Azienda Sanitaria Unica Regionale Delle Marche Di Ancona  Via Caduti del Lavoro, 40  Ancona, 60131  ITALY |
|  |  |  |  |  |  |

## Korea, Republic Of

**Coordinating Investigators:**

<None Entered>

| **Center** | **Principal Investigator** | **Co-Investigator(s)** | **Sub-Investigator(s)** | **Address(es)** | **Institutional Review Board or Ethics Committee Address(es)** |
| --- | --- | --- | --- | --- | --- |
|  |  |  |  |  |  |
| 1109 | Jung-Yoon Choe |  | Hyun-Young Jung  Dr. Seong-Kyu Kim  Hwa-Jeong Lee  Dr. Sung-Hoon Park | Daegu Catholic University Medical Center  Department of Rheumatology  3056-6 Daemyung-4 dong, Nam-gu  Daegu, 705-718  KOREA, REPUBLIC OF | IRB of Daegu Catholic University Medical Center  #202, Geumgang Villa, 991-8, Daemyung-10 dong, Nam-gu  Daegu, 705-812  KOREA, REPUBLIC OF |
|  |  |  |  |  |  |
| 1110 | Min-Chan Park |  | Soo-Jin Chung  Mi Ra Jang  Yong-Jin Kwon | Gangnam Severance Hospital  Rheumatology  712 Eonjuro, Gangnam-gu  Seoul, 135-720  KOREA, REPUBLIC OF | IRB of Gangnam Severance Hospital  712 Eonjuro, Gangnam-gu  Seoul, 135-720  KOREA, REPUBLIC OF |
|  |  |  |  |  |  |
| 1111 | Won Tae Chung |  | Yong-min Jo  Sang Yeob Lee  Sung Won Lee | Dong-A University Medical Center  Department of Rheumatology  1, Dongdaesin-dong, 3-ga, Seo-gu  Busan, 602-715  KOREA, REPUBLIC OF | IRB of Dong-A University Hospital  1, Dongdaesin-dong, 3-ga, Seo-gu  Busan, 602-715  KOREA, REPUBLIC OF |
|  |  |  |  |  |  |

## Spain

**Coordinating Investigators:**

<None Entered>

| **Center** | **Principal Investigator** | **Co-Investigator(s)** | **Sub-Investigator(s)** | **Address(es)** | **Institutional Review Board or Ethics Committee Address(es)** |
| --- | --- | --- | --- | --- | --- |
|  |  |  |  |  |  |
| 1013 | Alberto Alonso Ruiz |  | Marcelo Calabozo Raluy  Fernando Perez Ruiz | HOSPITAL DE CRUCES  SERVICIO DE REUMATOLOGIA  PLAZA DE CRUCES, S/N  BARACALDO, VIZCAYA 48903  SPAIN | Hospital Universitario Doctor Peset  Ethics Committee of Clinic Investigation  C/ Gaspar Aguilar, 90  1ª Planta. Consultas Externas  Valencia, 46017  SPAIN |
|  |  |  |  |  |  |
| 1014 | Carlos Manuel Gonzalez-Fernandez |  | Dr. Luis Carreño Perez  Francisco Javier Lopez Longo  Indalecio Monteagudo Saenz  Maria Montoro Alvarez | HOSPITAL GENERAL UNIVERSITARIO GREGORIO MARAÑON  SERVICIO DE REUMATOLOGIA  C/. DOCTOR ESQUERDO, 46  MADRID, MADRID 28007  SPAIN | Hospital Universitario Doctor Peset  Ethics Committee of Clinic Investigation  C/ Gaspar Aguilar, 90  1ª Planta. Consultas Externas  Valencia, 46017  SPAIN |
|  |  |  |  |  |  |
| 1015 | Eugenio Chamizo Carmona |  | Juan Jose Aznar Sanchez  Adela Gallego Flores  Noemi Patricia Garrido Puñal | HOSPITAL DE MERIDA  CONSULTAS EXTERNAS 3ª PLANTA. SERVICIO DE REUMATOLOGIA  C/MIGUEL SERVET S/N  MERIDA, BADAJOZ 06800  SPAIN | Hospital Universitario Doctor Peset  Ethics Committee of Clinic Investigation  C/ Gaspar Aguilar, 90  1ª Planta. Consultas Externas  Valencia, 46017  SPAIN |
|  |  |  |  |  |  |
| 1016 | Jose Andres Roman Ivorra |  | Cristina Alcañiz Escandell  Inmaculada Concepcion Chalmeta Verdejo  Nagore Fernandez-Llanio Comella  Elia Valls Pascual | HOSPITAL UNIVERSITARIO DR. PESET  SERVICIO DE REUMATOLOGIA  CONSULTAS EXTERNAS 2ª PLANTA  AVDA. GASPAR AGUILAR, 90 (C/ JUAN DE GARAY)  VALENCIA, VALENCIA 46017  SPAIN | Hospital Universitario Doctor Peset  Ethics Committee of Clinic Investigation  C/ Gaspar Aguilar, 90  1ª Planta. Consultas Externas  Valencia, 46017  SPAIN |
|  |  |  |  |  |  |
| 1017 | Juan Garcia Meijide |  | Myriam Liz Graña  Manuel Pombo Suarez | HOSPITAL NUESTRA SEÑORA DE LA ESPERANZA  AVENIDA DE LAS BURGAS,2  SANTIAGO DE COMPOSTELA, A CORUÑA 15705  SPAIN | Hospital Universitario Doctor Peset  Ethics Committee of Clinic Investigation  C/ Gaspar Aguilar, 90  1ª Planta. Consultas Externas  Valencia, 46017  SPAIN |
|  |  |  |  |  |  |
| 1018 | Jose Miguel Aranburu Albizuri |  | Olaia Begoña Fernandez-Berrizbeitia  Jose Manuel Gorordo Olaizola  Ignacio Torre Salaberri  Eduardo Ucar Angulo | HOSPITAL DE BASURTO  SERVICIO DE REUMATOLOGIA  AV. MONTEVIDEO Nº 18  BILBAO, BIZKAIA 48013  SPAIN | Hospital Universitario Doctor Peset  Ethics Committee of Clinic Investigation  C/ Gaspar Aguilar, 90  1ª Planta. Consultas Externas  Valencia, 46017  SPAIN |
|  |  |  |  |  |  |
| 1041 | Dr. Ricardo Blanco Alonso |  | Mario Agudo Bilbao  Ines Margarita Ayerbe Cintra  Miguel Angel Gonzalez-Gay Mantecon  Cristina Martinez Dubois  Dr. Victor Manuel Martinez Taboada  Maria Enriqueta Peiro Callizo  Dr. Jose Luis Peña Sagredo | HOSPITAL UNIVERSITARIO MARQUES DE VALDECILLA  CONSULTAS DE REUMATOLOGIA - EDIFICIO DE CONSULTAS, VALDECILLA SUR, SEGUNDA PLANTA  AVDA. VALDECILLA Nº 25,  SANTANDER, CANTABRIA 39008  SPAIN | Hospital Universitario Doctor Peset  Ethics Committee of Clinic Investigation  C/ Gaspar Aguilar, 90  1ª Planta. Consultas Externas  Valencia, 46017  SPAIN |
|  |  |  |  |  |  |

## Taiwan

**Coordinating Investigators:**

<None Entered>

| **Center** | **Principal Investigator** | **Co-Investigator(s)** | **Sub-Investigator(s)** | **Address(es)** | **Institutional Review Board or Ethics Committee Address(es)** |
| --- | --- | --- | --- | --- | --- |
|  |  |  |  |  |  |
| 1049 | Dr. Shue-Fen Luo |  | Hsiu-Feng Hsia  Chang-Fu Kuo | Chang Gung Medical Foundation-Linkou Branch/Department of Internal Medicine  5, Fu-Shin Street  Kweishan, Taoyuan County 333  TAIWAN | Chang Gung Medical Foundation, Institutional Review Board  No. 199 Tung Hwa North Road  Taipei, 10507  TAIWAN |
|  |  |  |  |  |  |
| 1050 | Dr. Tien-tsai Cheng |  | Ying-Chou Chen  Dr. Han-Ming Lai  Ya-Chi Ou  Shan-Fu Yu | Chang Gung Medical Foundation-Kaohsiung Branch/Division of Allergy, Immunology and Rheumatology  123, Ta-Pei Road  Niao Sung Hsiang, Kaohsiung County 833  TAIWAN | Chang Gung Medical Foundation, Institutional Review Board  No. 199 Tung Hwa North Road  Taipei, 10507  TAIWAN |
|  |  |  |  |  |  |
| 1051 * | Dr. Hung-An Chen  Dr. Pei-Chih Chen (Previous PI) |  | Dr. Pei-Chih Chen  Dr. Yeong-Jang Lin | Chi-Mei Medical Center  2F, Clinical Research Department  No. 901, Chung Hwa Road  Yung Kang City  Tainan, 710  TAIWAN | Chi Mei Medical Center, Institutional Review Board  901 Junghua Road  Tainan, Taiwan 710  TAIWAN |
|  |  |  |  |  |  |
| 1074 | Dr. Che-Chun Su |  | Ms. Jenny Chiang  Dr. Ying-Ming Chiu | Changhua Christian Hospital  135 Nanhsiao Street  Changhua, Changhua 500  TAIWAN | Changhua Christian Hospital, Institutional Review Board  135 Nan Hsiao Street  Changhua, Taiwan 500  TAIWAN |
|  |  |  |  |  |  |
| 1075 | Dr. Jui-Cheng Tseng |  | Ling-Jung Yen | Kaohsiung Veterans General Hospital  386 Ta-Chung First Road  Kaohsiung, 813  TAIWAN | Kaohsiung Veterans General Hospital, Institutional Review Board  386 Ta-Chung First Road  Kaohsiung, 813  TAIWAN |
|  |  |  |  |  |  |

## United States

**Coordinating Investigators:**

<None Entered>

| **Center** | **Principal Investigator** | **Co-Investigator(s)** | **Sub-Investigator(s)** | **Address(es)** | **Institutional Review Board or Ethics Committee Address(es)** |
| --- | --- | --- | --- | --- | --- |
|  |  |  |  |  |  |
| 1001 | Dr. Jeffrey Ross Curtis |  | Linda G. Cowden  Martha L. Sanderson  Anita S. Turner | University of Alabama at Birmingham  Arthritis Clinical Intervention Program  1717 6th Avenue South, SRC 076  Birmingham, AL 35294  UNITED STATES | Western Institutional Review Board  3535 7th Avenue SW  Olympia, WA 98502  UNITED STATES |
|  |  |  |  |  |  |
| 1019 | Dr. Luis Rolan Espinoza |  | Dr. Raquel S. Cuchacovich  Dr. Vijay Karia  Dr. Tahir Khan  Dr. Suman Lata | Louisiana State University Health Sciences Center  Department of Rheumatology  Suite 890  2820 Napoleon Avenue  New Orleans, LA 70115  UNITED STATES | LSU Health Sciences Center  Institutional Review Board  433 Bolivar Street  New Orleans, LA 70112  UNITED STATES |
|  |  |  |  |  |  |
| 1021 | Dr. Robert Greig Trapp |  | Belinda S. Brown  Monica Peregrin | The Arthritis Center  1200 Centre West Drive  Springfield, IL 62704  UNITED STATES | Quorum Institutional Review Board  Suite 1000  1601 Fifth Avenue  Seattle, WA 98101  UNITED STATES |
|  |  |  |  |  |  |
| 1024 | Dr. William Patrick Knibbe |  | Michelle Hicks  Janeen Jenkins  Dr. James Edward Loveless | St. Luke's Clinic - Rheumatology  (Clinic Location)  Suite 100  600 West Robbins Road  Boise, ID 83702  UNITED STATES  St. Luke's Intermountain Research Center  (Drug Shipment/ Administration)  Suite 402  600 West Robbins Road  Boise, ID 83702  UNITED STATES | Quorum Institutional Review Board  Suite 1000  1601 Fifth Avenue  Seattle, WA 98101  UNITED STATES |
|  |  |  |  |  |  |
| 1031 | Dr. William Stohl |  | Sandra Cardenas  Dr. Reena Kapadia Heyer  Dr. Roshan Kotha  Dr. Minh Chau Nguyen  Dr. Elizabeth Ortiz  Dr. Anne Violet Quismorio  Dr. Shylaja Rachabattula  Dr. Tien-I Karleen Su  Dr. Darice Yang  Dr. Li Yang | University of Southern California Keck School of Medicine  Department of Rheumatology - HMR 711  2011 Zonal Avenue  Los Angeles, CA 90033  UNITED STATES  University of Southern California University Hospital  CTU  1500 San Pablo Street  Los Angeles, CA 90033  UNITED STATES | University of Southern California Health Science Campus Institutional Review Board  LAC/USC Medical Center  Intern's Residence Dorm 425  2020 Zonal Avenue  Los Angeles, CA 90033  UNITED STATES |
|  |  |  |  |  |  |
| 1038 | Dr. Charles Meredith King II |  | Dr. David Kevin Asa  Dr. George A. Housley Jr.  Dr. Amit Bhaskar Prasad  Brandon L. Young | North Mississippi Medical Clinics, Inc.  845 South Madison Street  Tupelo, MS 38801  UNITED STATES | North Mississippi Medical Center Institutional Review Board  830 South Gloster Street  Tupelo, MS 38801  UNITED STATES |
|  |  |  |  |  |  |
| 1042 | Dr. Micha Abeles |  | Dr. Aryeh M. Abeles  Dr. Santhanam Lakshminarayanan  Kim Schwartz  Kim Striegel  Dr. John Vischio | University of Connecticut Health Center  263 Famington Avenue  Farmington, CT 06030-5353  UNITED STATES | University of Connecticut Health Center Institutional Review Board  16 Munson Road  Farmington, CT 06030  UNITED STATES |
|  |  |  |  |  |  |
| 1044 | Dr. Joel Charles Silverfield |  | Dr. Michael Claude Burnette  Dr. Harris Hugh McIlwain  Dr. Kimberly McIlwain Smith | Tampa Medical Group, PA  Suite 303  4700 North Habana Avenue  Tampa, FL 33614  UNITED STATES | Quorum Institutional Review Board  Suite 1000  1601 Fifth Avenue  Seattle, WA 98101  UNITED STATES |
|  |  |  |  |  |  |
| 1046 | Dr. Robert Michael Griffin Jr. |  | Dr. Michael Allen Borofsky  Brent William Calhoon  Jane Crosby  Dr. Saurin Mrugank Mehta  Dr. Peter Daniel Nicholas Jr.  Dr. Nancy Jane Walker  Dr. Jerome Stephen Weisberg | Clinical Research Center of Reading, LLP  2760 Century Boulevard  Wyomissing, PA 19610  UNITED STATES | Quorum Institutional Review Board  Suite 1000  1601 Fifth Avenue  Seattle, WA 98101  UNITED STATES |
|  |  |  |  |  |  |
| 1048 | Dr. Charles Henry Pritchard |  | Dr. David J. Chesner  Dr. Elana R. Eisner  Dr. Charles Michael Franklin  Dr. Dennis A. Jerdan  Dr. Mark Lopatin | Rheumatic Disease Associates, Ltd.  2360 Maryland Road  Willow Grove, PA 19090  UNITED STATES | Quorum Institutional Review Board  Suite 1000  1601 Fifth Avenue  Seattle, WA 98101  UNITED STATES |
|  |  |  |  |  |  |
| 1052 | Dr. James Michael Calmes |  | Dr. Naga S. Bushan  Dr. Jose Del Giudice  Dr. Jitendra Indrukumar Vasandani | Arthritis and Osteoporosis Associates, LLP  5220 80th Street  Lubbock, TX 79424  UNITED STATES | Quorum Institutional Review Board  Suite 1000  1601 Fifth Avenue  Seattle, WA 98101  UNITED STATES |
|  |  |  |  |  |  |
| 1053 | Dr. Christina Marie Charles-Schoeman |  | Amber A. Bechtel  Dr. Daniel E. Furst  Dr. Harold Edward Paulus  Sharon J. Quigley  Dr. Veena Kittane Ranganath | University of California Los Angeles  3rd Floor  1000 Veteran Avenue  Los Angeles, CA 90095  UNITED STATES | Western Institutional Review Board  3535 Seventh Avenue Southwest  Olympia, WA 98502  UNITED STATES |
|  |  |  |  |  |  |
| 1054 | Dr. Adrienne Hollander |  | Dr. Stephen L. Burnstein  Dr. James Paul Dwyer  Dr. Brian Lee Grimmett  Dr. Kenneth Howard Maurer  Dr. Michael Charles Schuster  Dr. Arielle S. Silver  Dr. Sheldon D. Solomon | Arthritis, Rheumatic & Back Disease Associates  Suite 101  2309 Evesham Road  Voorhees, NJ 08043  UNITED STATES | Quorum Institutional Review Board  Suite 1000  1601 Fifth Avenue  Seattle, WA 98101  UNITED STATES |
|  |  |  |  |  |  |
| 1055 | Dr. David Andrew Bong (Previous PI)  Dr. Theresa M. Karplus |  | Dr. Esther C. Hwang  Dr. Uzma A. Khan  Dr. Marcia J. Sparling | The Vancouver Clinic, Inc., PS  700 Northeast 87th Avenue  Vancouver, WA 98664  UNITED STATES  The Vancouver Clinic, Inc., PS  2525 Northeast 139th Street  Vancouver, WA 98686  UNITED STATES  The Vancouver Clinic, Inc., PS (administrative only)  Suite 260B  501 Southeast 172nd Avenue  Vancouver, WA 98684  UNITED STATES | Quorum Institutional Review Board  Suite 1000  1601 Fifth Avenue  Seattle, WA 98101  UNITED STATES |
|  |  |  |  |  |  |
| 1056 | Dr. Gary Edward Myerson |  | Anna L. Adams  Anna L. Ferguson  Dr. Paul George Sutej | Arthritis and Rheumatology of Georgia  Suite 220  980 Johnson Ferry Road  Atlanta, GA 30342  UNITED STATES | Quorum Institutional Review Board  Suite 1000  1601 Fifth Avenue  Seattle, WA 98101  UNITED STATES |
|  |  |  |  |  |  |
| 1057 | Dr. Joseph Eugene Huffstutter |  | Dr. William David Craig | Arthritis Associates, PLLC  1035 Executive Drive  Hixson, TN 37343  UNITED STATES | Quorum Institutional Review Board  Suite 1000  1601 Fifth Avenue  Seattle, WA 98101  UNITED STATES |
|  |  |  |  |  |  |
| 1058 | Dr. William F. C. Rigby |  | Dr. Daniel Arthur Albert  Dr. Lin Anita Jeromin Brown  Whitney F. Hilton  Dr. Jonathan D. Jones  Dr. John N. Mecchella  Dr. Alireza Meysami  Dr. Nicole M. Orzechowski  Dr. Robert L. Wortmann  Dr. Moe T. Zan  Dr. Alicia J. Zbehlik | Dartmouth-Hitchcock Medical Center  One Medical Center Drive  Lebanon, NH 03756  UNITED STATES | Committee for the Protection of Human Subjects  Dartmouth College  3rd floor, #6254  63 South Main Street  Hanover, NH 03755  UNITED STATES |
|  |  |  |  |  |  |
| 1062 | Dr. Philip A. Waller |  | Barbara A. Slusher  Dr. Prashanth R. Sunkureddi  DeAnna Elena Wallace | Accurate Clinical Research, Inc.  12553 Gulf Freeway  Houston, TX 77034  UNITED STATES | Quorum Institutional Review Board  Suite 1000  1601 Fifth Avenue  Seattle, WA 98101  UNITED STATES |
|  |  |  |  |  |  |
| 1063 | Dr. Suthin N. Songcharoen |  | Allison Spell  Courtney Stanford | Arthritis and Osteoporosis Treatment and Research Center  Suite 300  2550 Flowood Drive  Flowood, MS 39232  UNITED STATES | Quorum Institutional Review Board  Suite 1000  1601 Fifth Avenue  Seattle, WA 98101  UNITED STATES |
|  |  |  |  |  |  |
| 1072 | Dr. Craig Davis Scoville |  | Susan H. Hughes | Institute of Arthritis Research  2220 East 25th Street  Idaho Falls, ID 83404  UNITED STATES | Quorum Institutional Review Board  Suite 1000  1601 Fifth Avenue  Seattle, WA 98101  UNITED STATES |
|  |  |  |  |  |  |
| 1077 * | Dr. Lourdes J. Feliciano |  | Dr. Aixa Rivera  Dr. Dinamarca Rodriquez | The Alliance for Research and Knowledge Corporation  1427 Fernandez Juncos Avenue  San Juan, PR 00910  UNITED STATES | Quorum Institutional Review Board  Suite 1000  1601 Fifth Avenue  Seattle, WA 98101  UNITED STATES |
|  |  |  |  |  |  |
| 1079 | Dr. Simpson Bobo Tanner IV |  | Cindy Kate Anderson  Dr. John Michael Fahrenholz  Shannon B. Flowers | Vanderbilt University Medical Center  Suite 120  2611 West End Avenue  Nashville, TN 37203  UNITED STATES | Vanderbilt Institutional Review Board  1313 21st Avenue South  504 Oxford House  Nashville, TN 37232  UNITED STATES |
|  |  |  |  |  |  |
| 1080 | Dr. Rahul Keshav Patel |  | Dr. Jeffrey Kyle Bacon  Dr. Melanie Creech Barron  Susan N. Bitner  Sharon Natasha Cha  Dr. Michael Casey Coan  Linda G. Davis  Dr. Hugo Bittar Fonseca  Cynthia Ann Jimenez-Williams  Dr. Kathryn Michele Lawson  Dr. Robert Larry Marshall  Dr. Bernard Ross Rubin  Dr. Mansi Shah  Dr. Kokila Thirumurthi  Dr. Beth Ann Valashinas | University of North Texas Health Science Center at Fort Worth  Department of Internal Medicine - Rheumatology  855 Montgomery Street  Fort Worth, TX 76107  UNITED STATES  University of North Texas Health Science Center at Fort Worth  3500 Camp Bowie Boulevard  Fort Worth, TX 76107  UNITED STATES | University of North Texas Health Science Center at Fort Worth Institutional Review Board  3400 Camp Bowie Boulevard  Fort Worth, TX 76107  UNITED STATES |
|  |  |  |  |  |  |
| 1082 * | Dr. Dianne Lynn Petrone |  | Susan K. Burdett  Cathy A. Fernandez  Dr. Alex M. Limanni  Dr. Priya K. Nair  Dr. Himanshu R. Patel  Dr. Marian Eleanor Sackler  Dr. Linda G. Teague  Dr. Christopher V. Tehlirian  Dr. John J. Willis  Bing Zheng | Arthritis Centers of Texas  Suite 300  712 North Washington  Dallas, TX 75246  UNITED STATES | Quorum Institutional Review Board  Suite 1000  1601 Fifth Avenue  Seattle, WA 98101  UNITED STATES |
|  |  |  |  |  |  |
| 1083 | Dr. Norman Brian Gaylis |  | Dr. Marcos E. Maldonado  Dr. Elana M. Oberstein  Dr. Julia P. Savloff | Arthritis and Rheumatic Disease Specialties  Suite 200  21097 Northeast 27th Court  Aventura, FL 33180  UNITED STATES | Quorum Institutional Review Board  Suite 1000  1601 Fifth Avenue  Seattle, WA 98101  UNITED STATES |
|  |  |  |  |  |  |
| 1084 | Dr. Nicholas A. Patrone |  | Jan Adams  Lisa J. Steed | Boice-Willis Clinic, PA  Suite 210  901 North Winstead Avenue  Rocky Mount, NC 27804  UNITED STATES | Quorum Institutional Review Board  Suite 1000  1601 Fifth Avenue  Seattle, WA 98101  UNITED STATES |
|  |  |  |  |  |  |
| 1085 | Dr. Charles D. Radis  Dr. David Benjamin Talmadge (Previous PI) |  | Dr. Brian E. Daikh  Dr. Edward Fels  Dr. Stephanie D. Gartner-Fanburg  Dr. Brian J. Keroack  Dr. Marc Lawrence Miller | Rheumatology Associates  51 Sewall Street  Portland, ME 04102  UNITED STATES | Quorum Institutional Review Board  Suite 1000  1601 Fifth Avenue  Seattle, WA 98101  UNITED STATES |
|  |  |  |  |  |  |
| 1086 | Dr. Steven Jay Klein |  | Dawnetta S. Adams  Dr. Mary P. Howell | Klein & Associates, M.D., P.A.  Suites C & D  921 Seton Drive  Cumberland, MD 21502  UNITED STATES | Quorum Institutional Review Board  Suite 1000  1601 Fifth Avenue  Seattle, WA 98101  UNITED STATES |
|  |  |  |  |  |  |
| 1125 | Dr. Charles Allen Birbara |  | Dr. Sheela Kumar  Dr. Nassif Maalouli  Dr. Basheer Rahmoun | Clinical Pharmacology Study Group  26 Queen Street  Worcester, MA 01610  UNITED STATES | Quorum Review Institutional Review Board Incorporated  Suite 1000  1601 Fifth Avenue  Seattle, WA 98101  UNITED STATES |
|  |  |  |  |  |  |
| 1128 | Dr. Richard James Misischia |  | Dr. Robert J. Capps  Dr. Marcin T. Gornisiewicz  Nickie L. Hurst  Cathy C. Rhodes  Dr. Jay Henderson Warrick  Dr. Donna M. Winn  Dr. John Frederick Wolfe | Rheumatology Consultants, PLLC  Suite 200, Colony Park  4707 Papermill Drive  Knoxville, TN 37909-1900  UNITED STATES | Quorum Review Institutional Review Board Incorporated  Suite 1000  1601 Fifth Avenue  Seattle, WA 98101  UNITED STATES |
|  |  |  |  |  |  |
| 1129 | Dr. Charles L. Ludivico |  | Nancy Katherine McFadden  Maxine R. Paden  Erica L. Rau  Dr. Allen Jeffrey Samuels  Dr. Ranju Singh | East Penn Rheumatology Associates, PC  Suite 501 and 601  701 Ostrum Street  Bethlehem, PA 18015-1153  UNITED STATES | Quorum Review Institutional Review Board Incorporated  Suite 1000  1601 Fifth Avenue  Seattle, WA 98101  UNITED STATES |
|  |  |  |  |  |  |
| 1131 | Dr. Michael Thomas Stack |  | Norma S. Anderson | Diagnostic Rheumatology and Research, PC  Suite B-1  1030 East County Line Road  Indianapolis, IN 46227  UNITED STATES | Quorum Review Institutional Review Board Incorporated  Suite 1000  1601 Fifth Avenue  Seattle, WA 98101  UNITED STATES |
|  |  |  |  |  |  |
| 1132 | Dr. Geoffrey Stephen Gladstein |  | Laura Brothers  Janette Charles  Jennifer Molnar  Dr. Stephen J. Moses  Mary Ellen Porrata  Milena Seara  Beth Carlson Tuohy  Margaret E. Ziegler | New England Research Associates, LLC  Suite 101  5520 Park Avenue  Trumbull, CT 06611  UNITED STATES | Quorum Review Institutional Review Board Incorporated  Suite 1000  1601 Fifth Avenue  Seattle, WA 98101  UNITED STATES |
|  |  |  |  |  |  |
| 1133 | Dr. Puja Chitkara |  | Dr. Ara Hagop Dikranian  Dr. Michael Ira Keller  Timothy F. Lazarek  Jennifer Marconato | San Diego Arthritis Medical Clinic  Suite 300  3633 Camino Del Rio South  San Diego, CA 92108  UNITED STATES | Quorum Review Institutional Review Board Incorporated  Suite 1000  1601 Fifth Avenue  Seattle, WA 98101  UNITED STATES |
|  |  |  |  |  |  |
| 1138 | Dr. Roy Mitchell Fleischmann |  | Jean Ann Clark  Dr. Stanley Bruce Cohen  Dr. Thomas David Geppert  Dr. Imran Iqbal  Dr. Robert Neil Jenkins  Dr. Talat Jehan Kheshgi  Dr. Zoran Kurepa  Dr. Sharad Lakhanpal  Andrea Suzanne Martin  Dr. Catalina Orozco  Dr. Richard Leo Stern  Dr. Jack Bernstein Vine | Metroplex Clinical Research Center  Suite 810  8144 Walnut Hill Lane  Dallas, TX 75231  UNITED STATES | Quorum Review Institutional Review Board Incorporated  Suite 1000  1601 Fifth Avenue  Seattle, WA 98101  UNITED STATES |
|  |  |  |  |  |  |
