# Supplementary material for: Changes in serum creatinine in patients with active rheumatoid arthritis treated with tofacitinib: results from clinical trials
Source: Arthritis Res Ther. 2014 Jul 25;16(4):R158. doi: 10.1186/ar4673 (PMC4220634; doi:10.1186/ar4673)
Supplement: Supplementary file 5 — Additional file 5: List of Investigators and Corresponding Ethics Committees or Institutional Review Boards for the Phase 2 A3921035 study. (DOC 187 KB) [file 13075_2013_4378_MOESM5_ESM.doc]

# A4 LIST OF INVESTIGATORS AND CORRESPONDING ETHICS COMMITTEES OR INSTITUTIONAL REVIEW BOARDS

## Brazil

**Coordinating Investigators:**

<None Entered>

| **Center** | **Principal Investigator** | **Co-Investigator(s)** | **Sub-Investigator(s)** | **Address(es)** | **Institutional Review Board or Ethics Committee Address(es)** |
| --- | --- | --- | --- | --- | --- |
|  |  |  |  |  |  |
| 1053 | Dr. Joao Carlos Tavares Brenol |  | Dr. Claiton Viegas Brenol  Dr. Odirlei A. Monticielo  Dr. Tamara M. Mucenic  Dr. Ricardo Machado Xavier | Hospital de Clinicas de Porto Alegre  Servico de Reumatologia  Rua Ramiro Barcelos, 2350- 6º andar - sala 645 A  Bom Fim  Porto Alegre, RS 90035-903  BRAZIL | Comite de Etica em Pesquisa do Hospital de Clinicas de Porto Alegre - HCPA  Rua Ramiro Barcelos, 2350  Sala 2200 B - Bom Fim  Porto Alegre, RS 90035-903  BRAZIL |
|  |  |  |  |  |  |
| 1096 | Dr. Jussara de Almeida Lima Kochen |  |  | Hospital Alemao Oswaldo Cruz  Instituto de Ciências ¿ Centro de Pesquisa Clínica  Rua Joao Juliao, 331  Sao Paulo, SP 01323-903  BRAZIL | Comite de Etica em Pesquisa do Hospital Alemao Oswaldo Cruz  Rua Joao Juliao, 331 - 14º andar  Bela Vista  Sao Paulo, SP 01323-903  BRAZIL |

## Bulgaria

**Coordinating Investigators:**

Rositsa Antova

Minka Geteva

Spaska Velichkova

| **Center** | **Principal Investigator** | **Co-Investigator(s)** | **Sub-Investigator(s)** | **Address(es)** | **Institutional Review Board or Ethics Committee Address(es)** |
| --- | --- | --- | --- | --- | --- |
|  |  |  |  |  |  |
| 1066 | Prof. Zlatimir Kolarov |  | Dr. Penka Bekyarova  Dr. Daniela Dimitrova  Dr. Kameliya Ivanova Garbeva-Popova  Dr. Mariana Goycheva  Dr. Lubomir Marinov Marintchev  Dr. Simeon Monov  Assoc. Prof. Veneta Paskaleva-Peytcheva  Dr. Tzvetanka Petranova  Prof. Iordan Sheytanov  Dr. Ivan Sheytanov  Dr. Tsvetelina Dimitrova Yoneva | Clinic of Rheumatology Multiprofile Hospital for Active treatment Sveti Ivan Rilski  13, Urvich Str.  Sofia, 1612  BULGARIA | Ethic Committee for Multicenter Trials  26, Yanko Sakazov  Sofia, 1504  BULGARIA  Ethics Committee at MHAT "Sveti Ivan Rilski"  Ethics Committee  15 Academician Ivan Geshov Blvd.  Sofia, 1431  BULGARIA |
|  |  |  |  |  |  |
| 1067 | Dr. Daniela Bichovska |  | Dr. Ivan Bichovski  Dr. Mario Markov | Clinic of Internal Diseases, Multiprofile Hospital for Active treatment Sveta Anna  1, Dimitar Mollov Str.  Sofia, 1709  BULGARIA | Ethic Committee for Multicenter Trials  26, Yanko Sakazov  Sofia, 1504  BULGARIA  Ethics Committee at MHAT "Sveta Anna"  Ethics Committee  1 Dimitar Mollov Str.  Sofia, 1709  BULGARIA |
|  |  |  |  |  |  |
| 1068 | Dr. Boycho Oparanov |  | Dr. Borislava Angelova Ilchova  Dr. Raycho Raychev  Dr. Ignat Zhutev | Clinic of Cardiology and Rheumatology, Military Medical Academy  3, Georgi Sofiiski Str.  Sofia 1606  BULGARIA | Ethic Committee for Multicenter Trials  26, Yanko Sakazov  Sofia, 1504  BULGARIA  Ethics Committee  3, Georgi Sofiiski  Sofia, 1606  BULGARIA |
|  |  |  |  |  |  |
| 1069 | Assoc. Prof. Snezhanka Tisheva |  | Dr. Nadja Stancheva  Dr. Kiril Yablanski  Dr. Virzhiniya Yordanova | University Multiprofile Hospital for Active Treatment "Dr. Georgi Stranski"  Clinic of Cardiology and Rheumatology  8A Georgi Kotchev Str.  Pleven, 5800  BULGARIA | Ethic Committee for Multicenter Trials  26, Yanko Sakazov  Sofia, 1504  BULGARIA  Ethics Committee at University Multiprofile Hospital for Active Treatment "D-r Georgi Stranski"  Ethics Committee  8A Georgi Kotchev Str.  Pleven, 5800  BULGARIA |

## Chile

**Coordinating Investigators:**

<None Entered>

| **Center** | **Principal Investigator** | **Co-Investigator(s)** | **Sub-Investigator(s)** | **Address(es)** | **Institutional Review Board or Ethics Committee Address(es)** |
| --- | --- | --- | --- | --- | --- |
|  |  |  |  |  |  |
| 1092 | Dr. Pedro Miranda |  | Dr. Jorge Saavedra  Dr. Cecilia Trejo | Office of Dr. Pedro Miranda  Avda Bernardo O'Higgins 240, oficina 145  Santiago, RM 8331030  CHILE | Comite Etico Cientifico  Servicio de Salud Metropolitano Oriente  Av. Salvador 364  Santiago  CHILE |
|  |  |  |  |  |  |
| 1093 | Dr. Francisco Ballesteros |  | Dr. Maria Eugenia Alvarez  Dr. Carlos Fuentealba  Dr. Carola Martinez  Dr. Paula Pozo | Centro de Diagnóstico y Tratamiento San Borja Arriarán  Sección Reumatología  Amazonas 619  Santiago, RM 8360156  CHILE | Comite Etico Cientifico  Servicio de Salud Metropolitano Central  Santa Rosa 1234, Pabellon Errazuriz piso 2  Santiago, RM  CHILE |
|  |  |  |  |  |  |
| 1094 | Dr. Gloria Holuigue |  | Dr. Viviana Maluje | Clínica Santa María, Sección Reumatología  Fernando Manterola 0540  Providencia, Santiago, RM 7530206  CHILE | Comite Etico Cientifico, Servicio de Salud Metropolitano Oriente  Avenida Salvador 364  Providencia  Santiago, RM 7500922  CHILE  Comite de Etica  Clinica Santa Maria  Avenida Santa Maria 0410  Santiago  CHILE |

## Croatia

**Coordinating Investigators:**

<None Entered>

| **Center** | **Principal Investigator** | **Co-Investigator(s)** | **Sub-Investigator(s)** | **Address(es)** | **Institutional Review Board or Ethics Committee Address(es)** |
| --- | --- | --- | --- | --- | --- |
|  |  |  |  |  |  |
| 1081 | Simeon Grazio |  | dr. Tomislav Nemcic  Dr. Hana Skala | University Hosp. Sestre Milosrdnice, Clinic for Rheumatology, Physical and Rehabilitation Medicine  Vinogradska 29  Zagreb, 10000  CROATIA | Central Ethics Committee  Agency for Medicinal Products and Medical Devices  Ksaverska c. 4  Zagreb, 10000  CROATIA |
|  |  |  |  |  |  |
| 1082 | Bozidar Curkovic |  | Assistant Prof Durda Babic Naglic  Dr. Iva Popovic  Dr Iva Zagar | University Hospital Center "Zagreb"  Kispaticeva 12  Zagreb, 10000  CROATIA | Central Ethics Committee  Agency for Medicinal Products and Medical Devices  Ksaverska c. 4  Zagreb, 10000  CROATIA |
|  |  |  |  |  |  |
| 1102 | Assistant Prof.dr. Dusanka Martinovic Kaliterna |  | Dr. Mislav Radic | University Hospital Split, Department for Internal Medicine, Division of Clinical Rheumatology  Spinciceva1  Split, 21000  CROATIA | Central Ethics Committee  Agency for Medicinal Products and Medical Devices  Ksaverska c. 4  Zagreb, 10000  CROATIA |

## Czech Republic

**Coordinating Investigators:**

<None Entered>

| **Center** | **Principal Investigator** | **Co-Investigator(s)** | **Sub-Investigator(s)** | **Address(es)** | **Institutional Review Board or Ethics Committee Address(es)** |
| --- | --- | --- | --- | --- | --- |
|  |  |  |  |  |  |
| 1056 | Dr. Jan Rosa |  |  | DC Mediscan  Sustova 1930  Praha 11 - Chodov, 148 00  CZECH REPUBLIC | Eticka komise IKEM a FTNsP  Videnska 800  Praha 4 Krc, 140 59  CZECH REPUBLIC |
|  |  |  |  |  |  |
| 1057 | Dr. Petr Vitek |  |  | PV-Medical s.r.o.  Revmatologicka ambulance  Padelky I/3645  Zlin, 760 01  CZECH REPUBLIC | Eticka komise IKEM a FTNsP  Fakultni Thomayerova nemocnice s poliklinikou  Videnska 800  Praha 4 Krc, 140 59  CZECH REPUBLIC |
|  |  |  |  |  |  |
| 1058 | Dr. Petr Nemec |  | Dr. Leona Prochazkova | Fakultni nemocnice u sv. Anny v Brne  II. Interni klinika  Pekarska 53  Brno, 656 91  CZECH REPUBLIC | Eticka komise Fakultni nemocnice u sv. Anny v Brne  Pekarska 53  Brno, 656 91  CZECH REPUBLIC  Eticka komise IKEM a FTNsP  Videnska 800  Praha 4 Krc, 140 59  CZECH REPUBLIC |
|  |  |  |  |  |  |
| 1059 | Dr. Petr Kopsa |  | Dr. Lenka Zouharova | Fakultni Thomayerova nemocnice s poliklinikou  Revmatologicke a rehabilitacni oddeleni  Videnska 800  Praha 4, 140 59  CZECH REPUBLIC | Eticka komise IKEM a FTNsP  Fakultni Thomayerova nemocnice s poliklinikou  Videnska 800  Praha 4 Krc, 140 59  CZECH REPUBLIC |
|  |  |  |  |  |  |
| 1060 | Dr. Sarka Forejtova |  | Dr. Ladislav Senolt  Dr. Dana Tegzova | Revmatologicky ustav  Na Slupi 4  Praha 2, 128 50  CZECH REPUBLIC | Eticka komise  Revmatologicky ustav  Na Slupi 4  Praha 2, 128 50  CZECH REPUBLIC  Eticka komise IKEM a FTNsP  Fakultni Thomayerova nemocnice s poliklinikou  Videnska 800  Praha 4 Krc, 140 59  CZECH REPUBLIC |

## Germany

**Coordinating Investigators:**

<None Entered>

| **Center** | **Principal Investigator** | **Co-Investigator(s)** | **Sub-Investigator(s)** | **Address(es)** | **Institutional Review Board or Ethics Committee Address(es)** |
| --- | --- | --- | --- | --- | --- |
|  |  |  |  |  |  |
| 1076 | Prof. Dr. med. Christoph Baerwald |  | OA Dr. Sybille Arnold  Prof. em. Dr. med. Holm Haentzschel  Dr. Martin Hecker  Dr. Olga Malysheva  Dr. Matthias Pierer  PD Dr. med. habil. Ulf Wagner  OA Dr. Wolfram Seidel | Klinik fuer Gastroenterologie und Rheumatologie, Sektion Rheumatologie  Liebigstr. 22  Leipzig, 04103  GERMANY | Ethikkommission der Saechsischen Landesaerztekammer  Schuetzenhoehe 16  Dresden, 01099  GERMANY |
|  |  |  |  |  |  |
| 1078 | Dr. Ulrich von Hinueber |  | Dr. Winfried Demary | Rheumatologische Gemeinschaftspraxis  Bahnhofsallee 3-4  Hildesheim, 31134  GERMANY | Ethikkommission der Saechsischen Landesaerztekammer  Schuetzenhoehe 16  Dresden, 01099  GERMANY |
|  |  |  |  |  |  |
| 1079 | Dr. Leonore Unger |  | Dr.med. Markus Enderlein  Dr.med. Marten Kayser  Dr.med. Eva-Maria Wagner | Krankenhaus Friedrichstadt, 1. Med. Klinik  Friedrichstrasse 41  Dresden, 01067  GERMANY | Ethikkommission der Saechsischen Landesaerztekammer  Schuetzenhoehe 16  Dresden, 01099  GERMANY |
|  |  |  |  |  |  |
| 1080 | Prof. Dr. med. Juergen Wollenhaupt |  | Dr. med. Andrea Binda  Dr. med. Andrea Everding  Dr. med. Ulrike Schnoor  Dr. med. Wolfgang Winter | Klinikum Eilbek, Abt. fuer Rheumatologie  Dehnhaide 120  Hamburg, 22081  GERMANY | Ethikkommission der Saechsischen Landesaerztekammer  Schuetzenhoehe 16  Dresden, 01099  GERMANY |

## Greece

**Coordinating Investigators:**

<None Entered>

| **Center** | **Principal Investigator** | **Co-Investigator(s)** | **Sub-Investigator(s)** | **Address(es)** | **Institutional Review Board or Ethics Committee Address(es)** |
| --- | --- | --- | --- | --- | --- |
|  |  |  |  |  |  |
| 1062 | Prof. Loukas Settas |  | Dr. Maria Efstathiou  Dr. Savvas Kofidis  Dr. Konstantinos Lazaridis  Dr. Charalampos Maskalidis  Dr. Dimitrios Zisopoulos | A.H.E.P.A. Hospital/First Internal Medicine Clinic  1 Stilponos Kyriakidi Street  Thessaloniki, 54 636  GREECE | National Ethics Committee  284 Mesogion Avenue  Athens, 15562  GREECE |
|  |  |  |  |  |  |
| 1063 | Assoc. Prof. Panayiotis G. Vlachoyiannopoulos |  | Dr. Vasiliki-Kalliopi K. Bournia  Dr. Menelaos Manoussakis | Laikon Hospital, Department of Pathophysiology  M.Asias  75  Goudi, Athens 11527  GREECE | National Ethics Committee  284 Mesogion Avenue  Athens, 15562  GREECE |

## Hungary

**Coordinating Investigators:**

<None Entered>

| **Center** | **Principal Investigator** | **Co-Investigator(s)** | **Sub-Investigator(s)** | **Address(es)** | **Institutional Review Board or Ethics Committee Address(es)** |
| --- | --- | --- | --- | --- | --- |
|  |  |  |  |  |  |
| 1044 | Dr. Attila Kovacs |  | Dr. Judit Biro  Dr. Marianna Czifra  Dr. Anita Kozma  Dr. Laszlo Samson | MAV Korhaz es Rendelointezet  Verseghy F. u. 6-8.  Szolnok, H-5000  HUNGARY | Medical Research Council Ethics Committee for Clinical Pharmacology  Arany J. u. 6-8  Budapest, H-1051  HUNGARY |

## Italy

**Coordinating Investigators:**

<None Entered>

| **Center** | **Principal Investigator** | **Co-Investigator(s)** | **Sub-Investigator(s)** | **Address(es)** | **Institutional Review Board or Ethics Committee Address(es)** |
| --- | --- | --- | --- | --- | --- |
|  |  |  |  |  |  |
| 1048 | Prof. Marco Matucci Cerinic |  | Dr. Francesca Bartoli  Dr. Silvia Bellando Randone  Dr. Francesca Nacci | SOD, Medicina Interna 1 e Reumatologia, Villa Monna Tessa  Viale Pieraccini, 18  Firenze, 50139  ITALY | Comitato Etico per la Sperimentazione Clinica dei Medicinali  dell'Azienda Ospedaliero Universitaria Careggi di Firenze  Viale Pieraccini, 28  Firenze, 50139  ITALY |
|  |  |  |  |  |  |
| 1073 | Prof. Maurizio Cutolo |  | Maria Elena Secchi | UO Clinica Reumatologica, Dipartimento di Medicina Interna, Universita' degli Studi di Genova  Viale Benedetto XV, 6  Genova, 16132  ITALY | Comitato Etico Azienda Ospedaliera Universitaria San Martino  Largo Rosanna Benzi 10  Genova, 16132  ITALY |

## Korea, Republic Of

**Coordinating Investigators:**

<None Entered>

| **Center** | **Principal Investigator** | **Co-Investigator(s)** | **Sub-Investigator(s)** | **Address(es)** | **Institutional Review Board or Ethics Committee Address(es)** |
| --- | --- | --- | --- | --- | --- |
|  |  |  |  |  |  |
| 1040 | Eun Bong Lee |  | Jin Hyun Kim  Eun Young Lee  Kiwon Moon  Ran Song  Yeong-Wook Song  Jaeki Koh  Hee Jung Ryu | Seoul National University Hospital, Department of Internal Medicine  28 Yeongeon-dong, Jongno-gu  Seoul, 110-744  KOREA, REPUBLIC OF | IRB of Seoul National University Hospital  IRB of Seoul National University Hospital  28 Yeongeon-dong, Jongno-gu  Seoul, 110-744  KOREA, REPUBLIC OF |
|  |  |  |  |  |  |
| 1041 | Dae-Hyun Yoo |  | Dr. Sang-Cheol Bae  So Young Bang  Soo Kyung Cho  Jae-Bum Jun  Hee-Sun Kim  Il Kim  Tae-Hwan Kim  Jeong Ha Park  Song-Ree Park  Yoon-Kyoung Sung  Tae-Jong Kim  Yun Jung Kim  Hye-Ryeon Yun | Hanyang University Hospital, Department of Rheumatology  17 Hengdang-dong, Seongdong-gu  Seoul, 133-792  KOREA, REPUBLIC OF | IRB of Hanyang University Hospital  Institutional Review Board  17, Haengdang-Dong, Seongdong-Gu  Seoul, 133-792  KOREA, REPUBLIC OF |

## Mexico

**Coordinating Investigators:**

<None Entered>

| **Center** | **Principal Investigator** | **Co-Investigator(s)** | **Sub-Investigator(s)** | **Address(es)** | **Institutional Review Board or Ethics Committee Address(es)** |
| --- | --- | --- | --- | --- | --- |
|  |  |  |  |  |  |
| 1039 | Dr. Reyna Manuela Bustamante-Gonzalez |  | Dr. Norma Alicia Martinez-Trejo | Comite Mexicano para la Prevencion de la Osteoporosis, A.C.  Insurgentes sur 299 Mezzanine  Col Hipodromo  Mexico, D.F. 06100  MEXICO | Comite de Etica del Comite Mexicano para la Prevencion de la Osteoporosis, A. C.  Insurgentes sur 299  Col Hipodromo  Mexico, D. F. 06100  MEXICO |
|  |  |  |  |  |  |
| 1046 | Dr. Jose Javier Orozco-Alcala |  | Dr. Maria Dolores Ochoa-Rodriguez | Private Office  Reforma 2491  Fraccionamiento Ladron de Guevara, Sector Hidalgo  Guadalajara, Jalisco 44650  MEXICO | Comite de Etica del Hospital San Javier  Av. Pablo Casals 640  Col. Prados Povidencia  Guadalajara, Jalisco 44670  MEXICO |
|  |  |  |  |  |  |
| 1047 | Dr. Manuel Robles-San Roman |  | Maria Eugenia Davalos-Zugasti  Dr. Maria Consuelo Medina-Puente  Dr. Miguel Angel Torres-Rodriguez | Centro Medico Toluca  Av. Benito Juarez Norte 135-B Consultorio 308 Torre II  Col Barrio de San Mateo  Metepec, Estado de Mexico 52140  MEXICO | Comité de Bioética e Investigación  Facultad de Medicina de la Universidad Autónoma del Estado de México  Paseo Tollocan Esquina Jesús Carranza  Colonia Moderna de la Cruz  Toluca, Estado de México 50120  MEXICO |
|  |  |  |  |  |  |
| 1055 | Dr. Daniel Xavier Xibille-Friedmann |  | Sara Eugenia Hernandez-Gongora  Dr. Mariana Alvarez-Fuentes | INOVAMED Hospital  Cuauhtemoc 203-109  Colonia Lomas de la Selva  Cuernavaca, Morelos 62270  MEXICO | Comité de Ética del Hospital INOVAMED  Cuauhtemoc 305  Colonia Lomas de la Selva  Cuernavaca, Morelos 62270  MEXICO |

## Romania

**Coordinating Investigators:**

<None Entered>

| **Center** | **Principal Investigator** | **Co-Investigator(s)** | **Sub-Investigator(s)** | **Address(es)** | **Institutional Review Board or Ethics Committee Address(es)** |
| --- | --- | --- | --- | --- | --- |
|  |  |  |  |  |  |
| 1086 | Ruxandra Maria Ionescu |  | Dr. Andreea Ileana Borangiu  Dr. Maria Laura Isac  Daniela Opris | Spitalul Clinic "Sf. Maria"  B-dul Ion Mihalache 37-39  Bucuresti, 011172  ROMANIA | Academia de Stiinte Medicale,Comisia Nationala de Etica pentru Studiul Clinic al Medicamentului  Str. Av. Sanatescu nr. 48,  Sector 1,  Bucuresti, 011478  ROMANIA |
|  |  |  |  |  |  |
| 1087 | Prof. Maria Suta |  | Dr. Victoria Cristina Duminica  Dr. Ana-Maria Mihaela Ramazan | Spitalul Clinic Judetean de Urgenta Constanta,Sectia de Reumatologie  Bd. Tomis Nr. 145  Constanta, Constanta 900591  ROMANIA | Academia de Stiinte Medicale,Comisia Nationala de Etica pentru Studiul Clinic al Medicamentului  Str. Av. Sanatescu nr. 48,  Sector 1,  Bucuresti, 011478  ROMANIA |
|  |  |  |  |  |  |
| 1088 | Prof. Dr. Rodica Marieta Chirieac |  | Dr. Codrina Ancuta  Dr. Iulia Georgiana Nita  Dr. Mihaela Simona Stoica | Spitalul de Recuperare Iasi  Str Pantelimon Halipa 14  Iasi, Iasi 700661  ROMANIA | Academia de Stiinte Medicale,Comisia Nationala de Etica pentru Studiul Clinic al Medicamentului  Str. Av. Sanatescu nr. 48,  Sector 1,  Bucuresti, 011478  ROMANIA |

## Slovakia

**Coordinating Investigators:**

<None Entered>

| **Center** | **Principal Investigator** | **Co-Investigator(s)** | **Sub-Investigator(s)** | **Address(es)** | **Institutional Review Board or Ethics Committee Address(es)** |
| --- | --- | --- | --- | --- | --- |
|  |  |  |  |  |  |
| 1089 | Dr Pavol Polak |  |  | Nestatna reumatologicka ambulancia, NsP Zilina  ul. Vojtecha Spanyola 43  Zilina, 012 07  SLOVAKIA | Eticka komisia pri Narodnom ustave reumatickych chorob  ul.I.Krasku 4  Piestany, 921 01  SLOVAKIA  Eticka komisia pri Zilinskom samospravnom kraji  Zilinsky samospravny kraj, odbor zdravotnictva  Komenskeho ul.48  Zilina, 011 09  SLOVAKIA |
|  |  |  |  |  |  |
| 1090 * | Dr. Ivan Rybar |  | Dr. Dagmar Micekova | Narodny ustav reumatickych chorob  Nabrezie Ivana Krasku 4  Piestany, 921 12  SLOVAKIA | Eticka komisia pri Narodnom ustave reumatickych chorob  ul.I.Krasku 4  Piestany, 921 01  SLOVAKIA |

## Ukraine

**Coordinating Investigators:**

<None Entered>

| **Center** | **Principal Investigator** | **Co-Investigator(s)** | **Sub-Investigator(s)** | **Address(es)** | **Institutional Review Board or Ethics Committee Address(es)** |
| --- | --- | --- | --- | --- | --- |
|  |  |  |  |  |  |
| 1065 | Vira Iosypivna Tseluyko |  | Dr. Ol'ha Victorivna Radchenko  Dr. Viktoriya Victorivna Yarosh | City Clinical Hospital # 8, Dept of Cardiology and Functional Diagnostics  266g, Saltivske Shosse  Kharkiv , 61000  UKRAINE | Central Committee for Ethics Issues of Ministry of Health of Ukraine  5, Narodnogo opolchennya Street  Kyiv, 03680  UKRAINE |
|  |  |  |  |  |  |
| 1075 | Mykola A. Stanislavchuk |  | Dr. Nabil Shakhid Ali  Dr. Inna I. Andrushko  Dr. Olena O. Savytska | Vinnitsa Regional Clinical Hospital n.a. Pirogov, Dept of Faculty Therapy of Vinnitsa NMU  46, Pirogova Street  Vinnitsa, 21018  UKRAINE | Bioethics Committee of Vinnitsa Regional Clinical Hospital n.a. Pirogov  46 Pirogova Street  Vinnitsa, 21018  UKRAINE  Central Committee for Ethics Issues of Ministry of Health of Ukraine  5, Narodnogo opolchennya Street  Kyiv, 03680  UKRAINE |
|  |  |  |  |  |  |
| 1083 | Prof. Vadym Vizir |  | Dr. Olexandr E. Berezyn  Olexandr V. Demidenko  Dr. Anton S. Sadomov  Dr. Igor V. Zaika | City Hospital #7, Department of Internal Diseases #2 of Zaporizhzhia State Medical University  9 Lunacharskogo Street  Zaporizhzhia, 69118  UKRAINE | Central Committee for Ethics Issues of Ministry of Health of Ukraine  5, Narodnogo opolchennya Street  Kyiv, 03680  UKRAINE |
|  |  |  |  |  |  |
| 1084 | Prof. Oleg N. Nadashkevich |  | Dr. Halyna M. Hrytsenko | City Clinical Hospital #4, Dept of Internal Medicine #2 and Dermatology, Venereology of Lviv NMU  3 Sventsitskogo Street  Lviv, 79011  UKRAINE | Central Committee for Ethics Issues of Ministry of Health of Ukraine  5, Narodnogo opolchennya Street  Kyiv, 03680  UKRAINE |
|  |  |  |  |  |  |
| 1091 | Vladyslav V. Povoroznyuk |  | Dr. Maryna A. Bystrytska  Dr. Nataliia I. Dzerovych  Dr. Nataliia V. Grygorieva  Dr. Tetyana V. Orlyk | Institute of Gerontology, Department of Clinical Physiology and Pathology of Musculoskeletal System  67 Vyshgorodska Street  Kyiv, 04114  UKRAINE | Central Committee for Ethics Issues of Ministry of Health of Ukraine  5, Narodnogo opolchennya Str.  Kyiv, 03680  UKRAINE  Committee for Ethics Issues Of Clinical trials of Institute of Gerontology of AMS of Ukraine  67, Vyshgorodska Street  Kyiv, 04114  UKRAINE |

## United States

**Coordinating Investigators:**

<None Entered>

| **Center** | **Principal Investigator** | **Co-Investigator(s)** | **Sub-Investigator(s)** | **Address(es)** | **Institutional Review Board or Ethics Committee Address(es)** |
| --- | --- | --- | --- | --- | --- |
|  |  |  |  |  |  |
| 1003 | Dr. Joel Charles Silverfield |  | Dr. Michael Claude Burnette  Dr. Harris Hugh McIlwain  Dr. Kimberly McIlwain Smith | Tampa Medical Group, PA  Suite 303  4700 North Habana Avenue  Tampa, FL 33614  UNITED STATES | Quorum Institutional Review Board  Suite 1000  1601 Fifth Avenue  Seattle, WA 98101  UNITED STATES |
|  |  |  |  |  |  |
| 1004 | Dr. Alan Jan Kivitz |  | Dr. Lori Ann Lavelle  Dr. Frederick Timothy Murphy  Dr. Marianne L. Shaw | Altoona Center for Clinical Research  1125 Old Route 220 North  Duncansville, PA 16635  UNITED STATES | Quorum Institutional Review Board  Suite 1000  1601 Fifth Avenue  Seattle, WA 98101  UNITED STATES |
|  |  |  |  |  |  |
| 1005 | Dr. Mark Christopher Genovese |  | Dr. Eliza F. Chakravarty  Dr. Lorinda Chung  William Hewitt Robinson  Dr. Andrew L. Rozelle  Dr. Laura F. Su  Dr. Paul Utz | Stanford Health Services  Medical Specialty Clinics  A175  300 Pasteur Drive  Stanford, CA 94305  UNITED STATES  Stanford Health Services GCRC  HG130  300 Pasteur Drive  Stanford, CA 94305  UNITED STATES  Stanford Investigational Pharmacy  Drug Shipment  H0301  300 Pasteur Drive  Stanford, CA 94305  UNITED STATES  Stanford University Medical Center - School of Medicine  Division of Immunology and Rheumatology - MC5755 #203  1000 Welch Road  Palo Alto, CA 94304  UNITED STATES | Stanford University Administrative Panel on Human Subjects in Medical Research  1215 Welch Road  Module A  Stanford, CT 94305-5401  UNITED STATES |
|  |  |  |  |  |  |
| 1006 | Dr. Sanford Mayer Wolfe |  |  | STAT Research, Inc.  Suite 230  One Elizabeth Place  Dayton, OH 45408  UNITED STATES | Quorum Institutional Review Board  Suite 1000  1601 Fifth Avenue  Seattle, WA 98101  UNITED STATES |
|  |  |  |  |  |  |
| 1007 | Dr. Richard Roy Olson |  | Dr. David James Dansdill  Tami Kucia | Rockford Orthopedic Associates  324 Roxbury Road  Rockford, IL 61107  UNITED STATES | Quorum Institutional Review Board  Suite 1000  1601 Fifth Avenue  Seattle, WA 98101  UNITED STATES |
|  |  |  |  |  |  |
| 1008 | Dr. Richard B. Lies |  | Dr. Steen Erik Mortensen | Wichita Clinic PA  Rheumatology Department  3311 East Murdock  Wichita, KS 67208  UNITED STATES | Quorum Institutional Review Board  Suite 1000  1601 Fifth Avenue  Seattle, WA 98101  UNITED STATES |
|  |  |  |  |  |  |
| 1009 | Dr. Cummins Lue |  | Dr. James Howard Abraham III  Dr. Robert M. Brewer  Dr. Steve Holt  Dr. Richard William Houk  Dr. S. Michael Jones  Dr. Lisa Ann Jarvis Lowery  Dr. Laura B. Trigg  Dr. Margaret A. West | Little Rock Diagnostic Clinic  10001 Lile Drive  Little Rock, AR 72205  UNITED STATES | Quorum Institutional Review Board  Suite 1000  1601 Fifth Avenue  Seattle, WA 98101  UNITED STATES |
|  |  |  |  |  |  |
| 1010 | Dr. Roy Mitchell Fleischmann |  | Jean A. Clark  Dr. Stanley Bruce Cohen  Dr. Thomas David Geppert  Dr. Imran Iqbal  Dr. Robert Neil Jenkins  Dr. Sharad Lakhanpal  Dr. Richard L. Stern  Dr. Jack Bernstein Vine  Andrea S. Wheeler | Metroplex Clinical Research Center  Suite 441  5939 Harry Hines Boulevard  Dallas, TX 75235  UNITED STATES | Quorum Institutional Review Board  Suite 1000  1601 Fifth Avenue  Seattle, WA 98101  UNITED STATES |
|  |  |  |  |  |  |
| 1012 | Michael Steven Brooks |  | Dr. Shahin Bagheri  Dr. Steven Eyanson | Physicians Clinic of Iowa  600 7th St SE  Cedar Rapids, IA 52401-2112  UNITED STATES | Quorum Institutional Review Board  Suite 1000  1601 Fifth Avenue  Seattle, WA 98101  UNITED STATES |
|  |  |  |  |  |  |
| 1014 | Dr. Patrick Thomas Schuette |  | Dr. Erin L. Arnold  Dr. William Joseph Arnold  Dr. Alfonso E. Bello  Dr. Susan B. Broy  Dr. Gerald Marc Eisenberg  Dr. Mary L. Moran  Dr. Amanda K. Myers  Dr. John L. Skosey | Illinois Bone and Joint Institute, LLC  9000 Waukegan Rd  Morton Grove, IL 60053  UNITED STATES | Quorum Institutional Review Board  Suite 1000  1601 Fifth Avenue  Seattle, WA 98101  UNITED STATES |
|  |  |  |  |  |  |
| 1015 | Dr. Richard James Misischia |  | Dr. Robert J. Capps  Dr. Marcin T. Gornisiewicz  Dr. Jay Henderson Warrick  Dr. Donna M. Winn  Dr. John Frederick Wolfe | Rheumatology Consultants, PLLC  Colony Park, Suite 200  4707 Papermill Drive  Knoxville, TN 37909-1600  UNITED STATES | Quorum Review Inc.  Suite 1000  1601 Fifth Avenue  Seattle, WA 98101  UNITED STATES |
|  |  |  |  |  |  |
| 1019 | Dr. Luis Rolan Espinoza |  | Dr. Raquel Cuchacovich | Louisiana State University Health Sciences Center  Department of Rheumatology  7th & 8th Floor, Suites 700 & 890  2820 Napoleon Avenue  New Orleans, LA 70115  UNITED STATES | LSUHSC IRB  433 Bolivar Street  New Orleans, LA 70112  UNITED STATES |
|  |  |  |  |  |  |
| 1020 | Antony Hou |  | Dr. Eric C. Lee  Dr. Mohamed Bassam Sebai  Ms. Patricia E. DesLauriers  Tina Escobedo | Inland Rheumatology and Osteoporosis Medical Group  Ship Drug to:  Suite 306  548 North 13th Avenue  Upland, CA 91786  UNITED STATES  Inland Rheumatology and Osteoporosis Medical Group  Suite 201  510 North 13th Avenue  Upland, CA 91786  UNITED STATES  Inland Rheumatology and Osteoporosis Medical Group  Suite 204  548 North 13th Avenue  Upland, CA 91786  UNITED STATES | Quorum Institutional Review Board  Suite 1000  1601 Fifth Avenue  Seattle, WA 98101  UNITED STATES |
|  |  |  |  |  |  |
| 1021 | Dr. Basit Malik |  | Dr. Dennis Kurt Buth  Dr. Gregory F. Lakin  Dr. Neal B. Secrist  Kimberly Talbot | Professional Research Network of Kansas  Suite 400  345 Riverview  Wichita, KS 67203  UNITED STATES | Quorum IRB  Suite 1000  1601 Fifth Avenue,  Seattle, WA 98101  UNITED STATES |
|  |  |  |  |  |  |
| 1024 | Dr. Sanjay Kishore Udani |  | Dr. Boris D. Ratiner  Dr. Steven York | Medicus Research  Office of Dr. Ratiner  18376 Clark Street  Tarzana, CA 91356  UNITED STATES  Medicus Research  Suite 240 & 335  18250 Roscoe Boulevard  Northridge, CA 91325  UNITED STATES  Office of Dr. York  Suite 260  18250 Roscoe Boulevard  Northridge, CA 91325  UNITED STATES | Quorum Institutional Review Board  Suite 1000  1601 Fifth Avenue  Seattle, WA 98101  UNITED STATES |
|  |  |  |  |  |  |
| 1028 | Dr. Michael James Fairfax |  | Courtney G. McDaniel  Dr. Charles S. Mitchell  Beth A. Elliott  Ms. Amanda L. Lewis  Mr. Peter L. Trethewey | ArthroCare, Arthritis Care & Research PC  Suite 11  7525 East Broadway Road  Mesa, AZ 85208  UNITED STATES | Quorum Institutional Review Board  Suite 1000  1601 Fifth Avenue  Seattle, WA 98101  UNITED STATES |
|  |  |  |  |  |  |
| 1033 | Dr. Dayton Dennis Payne Jr. |  | Dr. Ronald David Caldwell Jr.  Dr. John Keith Earl | Hickory Family Practice Associates  52 12th Avenue Northeast  Hickory, NC 28601  UNITED STATES  Piedmont Rheumatology  230 18th Street Circle Southeast  Hickory, NC 28602  UNITED STATES  Unifour Medical Research  1036 2nd Street, NE  Hickory, NC 28601  UNITED STATES | Quorum Institutional Review Board  Suite 1000  1601 Fifth Avenue  Seattle, WA 98101  UNITED STATES |
|  |  |  |  |  |  |
| 1038 | Dr. Jeffrey Edward Poiley |  |  | Jeffrey E. Poiley MD, PA  324 East Par Avenue  Orlando, FL 32804  UNITED STATES | Quorum Institutional Review Board  Suite 1000  1601 Fifth Avenue  Seattle, WA 98101  UNITED STATES |
|  |  |  |  |  |  |
| 1098 | Dr. Atul Kumar Singhal |  | Julia A. Dilliard  Ms. Doris C. Harvey | Southwest Rheumatology, PA  Suite 615  18601 LBJ Freeway  Mesquite, TX 75150  UNITED STATES | Quorum Institutional Review Board  Suite 1000  1601 Fifth Avenue  Seattle, WA 98101  UNITED STATES |
