# Supplementary material for: Changes in serum creatinine in patients with active rheumatoid arthritis treated with tofacitinib: results from clinical trials
Source: Arthritis Res Ther. 2014 Jul 25;16(4):R158. doi: 10.1186/ar4673 (PMC4220634; doi:10.1186/ar4673)
Supplement: Supplementary file 6 — Additional file 6: List of Investigators and Corresponding Ethics Committees or Institutional Review Boards for the Phase 2 A3921039 study. (DOC 70 KB) [file 13075_2013_4378_MOESM6_ESM.doc]

# 16.1.4.1 LIST OF INVESTIGATORS AND CORRESPONDING ETHICS COMMITTEES OR INSTITUTIONAL REVIEW BOARDS

## Japan

**Coordinating Investigators:**

<None Entered>

| **Center** | **Principal Investigator** | **Co-Investigator(s)** | **Sub-Investigator(s)** | **Address(es)** | **Institutional Review Board or Ethics Committee Address(es)** |
| --- | --- | --- | --- | --- | --- |
|  |  |  |  |  |  |
| 1001 | Dr. Ryutaro Matsumura |  |  | National Hospital Organization Chiba-East Hospital  673  Nitona-chou  Chuou-ku  Chiba, Chiba 260-8712  JAPAN | National Hospital Organization Chiba-East Hospital  673  Nitona-chou  Chuou-ku  Chiba, Chiba 260-8712  JAPAN |
|  |  |  |  |  |  |
| 1002 | Dr. Kazuhiko Yamamoto |  | Keishi Fujio  Noboru Hagino  Hiroko Kanda  Kimito Kawahata  Kanae Kubo  Mikako Mori  Akiko Okamoto  Hirofumi Shoda | The University of Tokyo Hospital  7-3-1  Hongo  Bunkyo-ku, Tokyo 113-8655  JAPAN | The University of Tokyo hospital IRB  7-3-1  Hongo  Bunkyo-ku, Tokyo 113-8655  JAPAN |
|  |  |  |  |  |  |
| 1003 | Dr. Shigeto Tohma |  | Tatsuoh Ikenaka  Toshihiro Matsui  Dr. Hisanori Nakayama  Kota Shimada | National Hospital Organization Sagamihara National Hospital  18-1  Sakuradai  Sagamihara, Kanagawa 228-8522  JAPAN | NATIONAL HOSPITAL ORGANIZATION SAGAMIHARA NATIONAL HOPITAL IRB  18-1,sakuradai,  sagamihara-shi,  Kanagawa, 228-8522  JAPAN |
|  |  |  |  |  |  |
| 1004 | Dr. Takeshi Kuroda (Previous PI)  Masaaki Nakano |  | Daisuke Kobayashi  Dr. Takeshi Kuroda  Shuichi Murakami  Yoko Wada | Niigata University Medical & Dental Hospital  1-754  Asahimatchidoori  Chuou-ku  Niigata, Niigata 951-8520  JAPAN | Niigata University Medical & Dental Hospital IRB  754  Ichibancho  Asahimachidoori  Niigata, Niigata 951-8520  JAPAN |
|  |  |  |  |  |  |
| 1005 | Dr. Shiro Ohshima |  | Yoshinori Harada  Masaru Ishii  Taeko Ishii  Yoshinori Katada  Jyunichi Kikuta  Masato Matsushita  Takio Narikawa  Yukihiko Saeki  Eriko Tanaka  Takao Yamanaka | National Hospital Organization Osaka Minami Medical Center  2-1  Kidohigashimachi  Kawachinagano, Osaka 586-8521  JAPAN | National Hospital Organization Osaka Minami Center  2-1  Kidohigashimachi  Kawachinagano,  Osaka, Japan 586-8521  JAPAN |
|  |  |  |  |  |  |
| 1006 | Yoshiya Tanaka |  | Shunsuke Fukuyo  Kentaro Hanami  Shigeru Iwata  Satoshi Kubo  Kazuhisa Nakano  Masao Nawata  Kazuyoshi Saito  Norifumi Sawamukai  Katsunori Suzuki  Kunihiro Yamaoka | University of Occupational and Environmental Health Hospital  1-1  Iseigaoka  Yahatanishi-ku, Kitakyusyu Fukuoka  JAPAN | University of Occupational and Environmental Health Hospital IRB  1-1  Iseigaoka  Yahata-Nishi-ku  Kita-Kyushu,, Fukuoka 807-8555  JAPAN |
|  |  |  |  |  |  |
| 1007 | Dr. Hisashi Yamanaka  Naoyuki Kamatani (Previous PI) |  | Dr. Takefumi Furuya  Dr. Masako Hara  Dr. Naomi Ichikawa  Dr. Katsunori Ikari  Dr. Tokiko Kanno  Dr. Yasushi Kawaguchi  Dr. Mariko Kitahama  Dr. Tsuyoshi Kobashigawa  Dr. Yumi Koseki  Dr. Shigeru Kotake  Dr. Shigeki Momohara  Dr. Ayako Nakajima  Dr. Yuki Nanke  Hiroshi Okamoto  Dr. Seiji Saito  Dr. Tadashi Sakurai  Dr. Eri Sato  Dr. Makoto Soejima  Dr. Atsuo Taniguchi  Dr. Chihiro Terai | Tokyo Women's Medical University, Institute of Rheumatology  10-22  Kawada-cho  Shinjyuku-ku, Tokyo 162-0054  JAPAN | Tokyo Women's Medical University Hospital IRB  8-1  Kawada-cho  Shinjyuku-ku, Tokyo 162-8666  JAPAN |
|  |  |  |  |  |  |
| 1008 | Dr. Nobuyuki Miyasaka |  | Dr. Masayoshi Harigai  Dr. Hitoshi Kohsaka  Dr. Ryuji Koike  Dr. Yukiko Komano  Dr. Tetsuo Kubota  Toshihiro Nanki  Dr. Yoshinori Nonomura  Dr. Fumihito Suzuki  Dr. Kazuki Takada  Dr. Michi Tanaka | Tokyo Medical And Dental University Hospital, Faculty of Medicine  1-5-45  Yushima  Bunkyo-ku, Tokyo 113-8519  JAPAN | Tokyo Medical And Dental University Hospital, Faculty of Medicine IRB  1-5-45  Yushima  Bunkyo-ku,, Tokyo 113-8519  JAPAN |
|  |  |  |  |  |  |
| 1009 | Tsutomu Takeuchi |  | Dr. Haruo Abe  Dr. Koichi Amano  Dr. Hideto Kameda  Hayato Nagasawa  Katsuya Suzuki  Kensei Tsusaka | Saitama Medical Center  1981  Tsujido-machi, Kamoda  Kawagoe-shi, Saitama 350-8550  JAPAN | Institutional Review Board (IRB) of Saitama Medical Center, Saitama Medical University  1981  Tsujido-machi  Kamoda  Kawagoe-shi, Saitama 350-8550  JAPAN |
|  |  |  |  |  |  |
| 1010 | Hajime Yamagata |  |  | National Hospital Organization MURAYAMA Medical Center  2-37-1  Gakuen  Musashimurayama-shi, Tokyo 208-0011  JAPAN | National Hospital Organization MURAYAMA Medical Center IRB  2-37-1  Gakuen  Musashimurayama-shi,, Tokyo 208-0011  JAPAN |
|  |  |  |  |  |  |
| 1011 | Shunsuke Mori |  | Kerstin Bashir  Isamu Chou | Kumamoto Saishunso National Hospital  2659  Suya  Koushi, Kumamoto 861-1196  JAPAN | Kumamoto Saishunso National Hospital IRB  2659  Suya,  Koushi-shi,, Kumamoto 861-1196  JAPAN |
|  |  |  |  |  |  |
| 1012 | Shuji Ohta |  | Midori Akatsu | Taga General Hospital  2-1-2  Kokubu-cho  Hitachi-shi, Ibaraki 316-0035  JAPAN | Taga General Hospital IRB  2-1-2,  Kokubu-cho,  Hitachi-shi,, Ibaraki 316-0035  JAPAN |
|  |  |  |  |  |  |
| 1013 | Hiroshi Tsuda |  | Katsura Houtatsu  Kwang Seok Yang | Juntendo Tokyo Koto Geriatric Medical Center  3-3-20  Shinsuna  Koto-ku, Tokyo 136-0075  JAPAN | Juntendo Tokyo Koto Geriatric Medical Center IRB  3-3-20  shinnsuna,  koutou-ku,, Tokyo 136-0075  JAPAN |
|  |  |  |  |  |  |
| 1014 | Hisaji Ohshima |  | Kumiko Akiya | National Hospital Organization Tokyo Medical Center  2-5-1  Higashigaoka  Meguro-ku, Tokyo 152-8902  JAPAN | National Hospital Organization Tokyo Medical Center IRB  2-5-1  Higashigaoka  Meguro-ku, Tokyo 152-8902  JAPAN |
|  |  |  |  |  |  |
| 1015 | Kenjiro Yamanaka |  | Yoshinori Kanai  Soichiro Nakano  Kaoru Sugimoto | Sasaki Foundation Kyoundo Hospital  1-8  Kandasurugadai  Chiyoda-ku, Tokyo 101-0062  JAPAN | Sasaki Foundation Kyoundo Hospital IRB  1-8  kandasurugadai,  chiyoda-ku,, Tokyo 101-0062  JAPAN |
|  |  |  |  |  |  |
| 1016 | Eiichi Suematsu |  | Yukio Esaki  Goh Hirata  Takashi Itokawa  Hisaaki Miyahara  Tomoya Miyamura  Masataka Nakamura  Koshiro Sonomoto  Hideyuki Watanabe  Masahiro Yamamoto | National Hospital Organization Kyushu Medical Center  1-8-1  Jigyohama  Chuo-ku  Fukuoka, Fukuoka 810-8563  JAPAN | National Hospital Organization Kyushu Medical Center IRB  1-8-1  Chigyohama  Chuo-ku, Fukuoka 810-8563  JAPAN |
|  |  |  |  |  |  |
| 1017 | Yasuhiko Munakata |  | Naoko Misu | Taihaku Sakura Hospital  1-12-26  Tomizawa  Taihaku-ku  Sendai, Miyagi 982-0032  JAPAN | NS Clinic Institutional Review Board  2-5  Shinmachi  Hachioji, Tokyo 192-0065  JAPAN |
|  |  |  |  |  |  |
| 1018 | Yoshinari Takasaki |  | Hirofumi Amano  Shouseki Lee  Ran Matsudaira  Masakazu Matsushita  Shinji Morimoto  Michihiro Ogasawara  Hitoshi Ogasawara  Kurisu Tada  Naoto Tamura  Ken Yamaji | Juntendo University  3-1-3  Hongo  Bunkyo-k, Tokyo 113-8431  JAPAN | Juntendo University Hospital IRB  Juntendo University Hospital  3-1-3  Hongo  Bunkyo-ku, Tokyo 113-8431  JAPAN |
|  |  |  |  |  |  |
| 1019 | Hirobumi Kondo |  | Kenta Hoshi  Hide Nagaba | Kitasato Institute Medical Center Hospital  6-100  Arai  Kitamoto, Saitama 364-8501  JAPAN | Kitasato Institute Medical Center Hospital  6-100  Arai¿  Kitamoto, Saitama 364-8501  JAPAN |
|  |  |  |  |  |  |
