# Supplementary material for: Changes in serum creatinine in patients with active rheumatoid arthritis treated with tofacitinib: results from clinical trials
Source: Arthritis Res Ther. 2014 Jul 25;16(4):R158. doi: 10.1186/ar4673 (PMC4220634; doi:10.1186/ar4673)
Supplement: Supplementary file 7 — Additional file 7: List of Investigators and Corresponding Ethics Committees or Institutional Review Boards for the Phase 2 A3921040 study. (DOC 168 KB) [file 13075_2013_4378_MOESM7_ESM.doc]

# 16.1.4 LIST OF INVESTIGATORS AND CORRESPONDING ETHICS COMMITTEES OR INSTITUTIONAL REVIEW BOARDS

## Japan

**Coordinating Investigators:**

<None Entered>

| **Center** | **Principal Investigator** | **Co-Investigator(s)** | **Sub-Investigator(s)** | **Address(es)** | **Institutional Review Board or Ethics Committee Address(es)** |
| --- | --- | --- | --- | --- | --- |
|  |  |  |  |  |  |
| 1001 | Dr. Ryutaro Matsumura |  |  | National Hospital Organization Chiba-East Hospital  673  Nitona-chou  Chuou-ku  Chiba, Chiba 260-8712  JAPAN | National Hospital Organization Chiba-East Hospital  673  Nitona-chou  Chuou-ku  Chiba, Chiba 260-8712  JAPAN |
|  |  |  |  |  |  |
| 1003 | Dr. Shigeto Tohma |  | Hiroshi Furukawa  Hidekazu Futami  Atsushi Hashimoto  Tatsuoh Ikenaka  Kanako Iwata  Toshihiro Matsui  Hisanori Nakayama  Yuko Okazaki  Kota Shimada  Hirokazu Takaoka | National Hospital Organization Sagamihara National Hospital  18-1  Sakuradai, Minami-ku  Sagamihara, Kanagawa 252-0392  JAPAN | National Hospital Organization Sagamihara National Hospital  18-1  Sakuradai, Minami-ku  Sagamihara, Kanagawa 252-0392  JAPAN |
|  |  |  |  |  |  |
| 1005 | Masato Matsushita |  | Yoshinori Harada  Taeko Ishii  Yoshinori Katada  Dr. Shiro Ohshima  Yukihiko Saeki  Eriko Tanaka | National Hospital Organization Osaka Minami Center  2-1  Kidohigashimachi  Kawachinagano, Osaka 586-8521  JAPAN | National Hospital Organization Osaka Minami Center  2-1  Kidohigashimachi  Kawachinagano,  Osaka, Japan 586-8521  JAPAN |
|  |  |  |  |  |  |
| 1006 | Dr. Yoshiya Tanaka |  | Shunsuke Fukuyo  Kentaro Hanami  Dr. Eri Hirakawa  Dr. Shintaro Hirata  Shigeru Iwata  Dr. Takayuki Katsuyama  Satoshi Kubo  Dr. Ippei Miyagawa  Kazuhisa Nakano  Masao Nawata  Kazuyoshi Saito  Dr. Kazuki Someya  Koshiro Sonomoto  Dr. Shizuyo Tsujimura  Kunihiro Yamaoka  Dr. Maiko Yoshikawa  Dr. Sonosuke Yukawa  Dr. Naoki Yunoue | University of Occupational and Environmental Health Hospital  1-1  Iseigaoka  Yahatanishi-ku  Kitakyusyu, Fukuoka 807-8555  JAPAN | University of Occupational and Environmental Health Hospital IRB  1-1  Iseigaoka  Yahata-Nishi-ku  Kita-Kyushu,, Fukuoka 807-8555  JAPAN |
|  |  |  |  |  |  |
| 1007 | Dr. Hisashi Yamanaka |  | Sayumi Baba  Dr. Chikako Fukasawa  Dr. Takefumi Furuya  Daisuke Hoshi  Dr. Naomi Ichikawa  Dr. Katsunori Ikari  Dr. Takuji Iwamoto  Dr. Tokiko Kanno  Dr. Mariko Kitahama  Dr. Tsuyoshi Kobashigawa  Dr. Shigeru Kotake  Dr. Yumi Kozeki  Dr. Shigeki Momohara  Dr. Ayako Nakajima  Dr. Yuki Nanke  Dr. Eri Sato  Yohei Seto  Kumi Shidara  Kae Takagi  Dr. Atsuo Taniguchi  Akiko Tochimoto  Dr. So Tsukahara  Wako Urano | Tokyo Women's Medical University, Institute of Rheumatology  10-22  Kawada-cho  Shinjyuku-ku, Tokyo 162-0054  JAPAN | Tokyo Women's Medical University Hospital IRB  8-1  Kawada-cho  Shinjyuku-ku, Tokyo 162-8666  JAPAN |
|  |  |  |  |  |  |
| 1008 | Nobuyuki Miyasaka |  | Dr. Masayoshi Harigai  Shinya Hirata  Hideyuki Iwai  Dr. Hitoshi Kohsaka  Dr. Ryuji Koike  Dr. Tetsuo Kubota  Fumitaka Mizoguchi  Toshihiro Nanki  Dr. Yoshinori Nonomura  Dr. Kazuki Takada  Dr. Michi Tanaka | Tokyo Medical And Dental University Hospital, Faculty of Medicine  1-5-45  Yushima  Bunkyo-ku, Tokyo 113-8519  JAPAN | Tokyo Medical And Dental University Hospital, Faculty of Medicine  1-5-45  Yushima  Bunkyo-ku,, Tokyo 113-8519  JAPAN |
|  |  |  |  |  |  |
| 1009 | Dr. Kouichi Amano |  | Dr. Hideto Kameda  Dr. Hayato Nagasawa  Dr. Katsuya Suzuki  Tsutomu Takeuchi  Dr. Kensei Tsusaka | Saitama Medical Center  1981  Kamoda  Kawagoe-shi, Saitama 350-8550  JAPAN | Saitama medical center IRB  1981  Kamoda, Kawagoe-shi  Saitama, Japan 350-8550  JAPAN |
|  |  |  |  |  |  |
| 1010 | Hajime Yamagata |  | Shinichiro Iwata  Hideaki Murakami  Yoshihiro Sasazaki | National Hospital Organization MURAYAMA Medical Center  2-37-1  Gakuen  Musashimurayama-shi, Tokyo 208-0011  JAPAN | National Hospital Organization MURAYAMA Medical Center  2-37-1  Gakuen  Musashimurayama-shi,, Tokyo 208-0011  JAPAN |
|  |  |  |  |  |  |
| 1011 * | Dr. Shunsuke Mori |  | Akihisa Yamashita  Kensuke Yonemura | Kumamoto Saishunso National Hospital  2659  Suya  Koushi, Kumamoto 861-1196  JAPAN | Kumamoto Saishunso National Hospital IRB  2659  Suya,  Koushi-shi,, Kumamoto 861-1196  JAPAN |
|  |  |  |  |  |  |
| 1016 | Eiichi Suematsu |  | Yukio Esaki  Goh Hirata  Akie Hirata  Yoshiro Horai  Hisaaki Miyahara  Tomoya Miyamura  Masataka Nakamura  Masahiro Yamamoto | National Hospital Organization Kyushu Medical Center  1-8-1  Jigyohama  Chuo-ku  Fukuoka, Fukuoka 810-8563  JAPAN | National Hospital Organization Kyushu Medical Center IRB  1-8-1  Jigyohama  Chuo-ku  Fukuoka, Fukuoka 810-8563  JAPAN |
|  |  |  |  |  |  |
| 1017 | Yasuhiko Munakata |  | Kazuyuki Honda  Hideyuki Saito  Shigeru Wakatsuki | Taihaku Sakura Hospital  1-12-26  Tomizawa  Taihaku-ku  Sendai, Miyagi 982-0032  JAPAN | NS Clinic Institutional Review Board  2-26-9  Myojincho  Hachioji, Tokyo 192-0046  JAPAN |
|  |  |  |  |  |  |
| 1019 | Dr. Hide Yoshida |  | Dr. Kenta Hoshi  Hirobumi Kondo  Takeo Kudo  Toshimichi Matsui | Kitasato Institute Medical Center Hospital  6-100  Arai  Kitamoto, Saitama 364-8501  JAPAN | Kitasato Institute Medical Center Hospital IRB  6-100  Arai  Kitamoto, Saitama 354-8501  JAPAN |
|  |  |  |  |  |  |
| 1021 | Katsumi Chiba |  |  | Fukusima Daiichi Hospital  16-2  Narude  Kitasawamata-aza  Fukusima, Fukusima 960-8251  JAPAN | fukushimadaiichi hospital IRB  16-2  Narude  Kitasawamata-aza  Fukushima, Fukushima 960-8251  JAPAN |
|  |  |  |  |  |  |
| 1022 | Yasushi Nawata |  | Dr. Shigekazu Takahashi  Dr. Shigeru Tanaka | Chiba-ken Saiseikai Narashino Hospital  1-1-1  Izumi-cho  Narashino, Chiba 275-8580  JAPAN | Chiba-ken Saiseikai Narashino Hospital IRB  1-1-1  Izumi-cho  Narashino, Chiba 275-8580  JAPAN |
|  |  |  |  |  |  |
| 1023 | Akihiro Yamaguchi |  | Noboru Hagino  Yukiko Iwasaki | Fukuhara Hospital  2-8-16  Kitazawa  Setagaya-ku, Tokyo 155-0031  JAPAN | Sone Clinic IRB  3-31-1  Shinjuku  Shinjuku-ku, Tokyo 160-0022  JAPAN |
|  |  |  |  |  |  |
| 1024 | Shigeru Honjyo |  | Mitsuhiro Kawano  Hirofumi Taki | Toyama-ken Saiseikai Takaoka Hospital  387-1  Futaduka  Takaoka, Toyama 933-8525  JAPAN | Toyama-ken Saiseikai Takaoka Hospital Institutional Review Board  387-1  Futaduka  Takaoka, Toyama 933-8525  JAPAN |
|  |  |  |  |  |  |
| 1025 | Yuji Yamanishi |  |  | Hiroshima Rheumatology Clinic  10-13  Teppo-cho  Naka-ku  Hiroshima, Hiroshima 730-0017  JAPAN | Sone Clinic IRB  3-31-1  Shinjuku  Shinjuku-ku, Tokyo 160-0022  JAPAN |
|  |  |  |  |  |  |
| 1026 | Yoshinobu Koyama |  | Kenji Fujii  Toshiyuki Ota | Aso Iizuka Hospital  3-83  Yoshio-machi  Iiduka, Fukuoka 820-8505  JAPAN | Aso Iizuka Hospital  3-83  Yoshio-machi  Iiduka, Fukuoka 820-8505  JAPAN |
|  |  |  |  |  |  |
| 1027 | Eisuke Shono |  |  | SHONO Rheumatism Clinic  1-10-27  Nishijin  Sawara-ku, Fukuoka 814-0002  JAPAN | Sone Clinic IRB  3-31-1  Shinjuku  Shinjuku-ku, Tokyo 160-0022  JAPAN |
|  |  |  |  |  |  |
| 1028 | Tomomi Tsuru |  | Takashi Etoh  Yasushi Inoue  Misato Nakagawa  Hitoshi Nakashima  Masanari Shiramoto | Medical Corporation Souseikai PS Clinic  Random Square 8F, 6-18  Tenyamachi  Kakata-ku  Fukuoka, Fukuoka 812-0025  JAPAN | Medical Co. LTA Kyushu clinical pharmacology reserch clinic IRB  2-13-16  jigyou  Chuuo-ku, Fukuoka 810-0064  JAPAN |
|  |  |  |  |  |  |
| 1029 | Kiyoshi Migita |  | Yasumori Izumi  Taiichiro Miyashita  Satoru Motokawa  Tadayoshi Ohno  Takafumi Torigoshi | National Hospital Organization Nagasaki Medical Center  2-1001-1  Kubara  Ohmura, Nagasaki 856-0835  JAPAN | National Hospital Organization Central Review Board  2-5-21  Higashigaoka  Meguro, Tokyo 152-0021  JAPAN |
|  |  |  |  |  |  |
| 1030 | Yukitaka Ueki |  | Nozomi Iwanaga  Hironobu Sato  Kaoru Terada  Satoshi Yamasaki | Sasebo Chuo Hospital  15  Yamato-cho  Sasebo, Nagasaki 857-1195  JAPAN | Sasebo Chuo Hospital IRB  Sasebo Chuo Hospital  15  Yamato-cho  Sasebo, Nagasaki 857-1195  JAPAN |
|  |  |  |  |  |  |
| 1031 | Motohiro Oribe |  |  | A Medical Corporation Oribe Rheumatism Internist Clinic  1-8-15  Higasiomichi  Oita, Oita 870-0823  JAPAN | Sone Clinic IRB  3-31-1  Shinjuku  Shinjuku-ku, Tokyo 160-0022  JAPAN |
|  |  |  |  |  |  |
| 1032 | Takao Sugiyama |  | Masaaki Furukawa  Arifumi Iwata  Makoto Sueishi  Toyohiko Sugimoto | Shimoshizu National Hospital  934-5  Shikawatashi  Yotsukaidou, Chiba 284-0003  JAPAN | National Hospital Organization Central Review Board  2-5-21  Higashigaoka  Meguro, Tokyo 152-0021  JAPAN |
|  |  |  |  |  |  |
| 1033 | Yojiro Kawabe |  | Koichiro Aratake  Fumiko Tanaka | National Hospital Organization Ureshino Medical Center  2436  Ooaza shimojyukuhei  Ureshino-machi  Ureshino-shi, Saga 843-0393  JAPAN | National Hospital Organization Central Review Board  2-5-21  Higashigaoka  Meguro, Tokyo 152-0021  JAPAN |
|  |  |  |  |  |  |
| 1035 | Shigenori Tamaki |  | Yumiko Asanuma  Motokazu Kai  Takeshi Nagakura  Kunikazu Ogawa  Tetsuya Sano  Masaru Tanaka  Ikuko Tanaka | National hospital Organization Mie Chuou Medical Center  2158-5  Myojin-Cho, Hisai  Tsu, Mie 514-1101  JAPAN | National Hospital Organization Central Review Board  2-5-21  Higashigaoka  Meguro, Tokyo 152-0021  JAPAN |
|  |  |  |  |  |  |
| 1036 | Masakazu Kondo |  |  | Kondo clinic for rheumatism and orthopaedics  3-10-11  Tenjin, Chuo-ku  Fukuoka, Fukuoka 810-0001  JAPAN | Haradoi Hospital  6-40-8  Aoba  Higashi-ku  Fukuoka, Fukuoka 813-0025  JAPAN |
|  |  |  |  |  |  |
| 1037 | Masaya Mukai |  | Makoto Kondo | Sapporo city general hospital  13-1-1  Kita11jonishi, Chuo-ku  Sapporo, Hokkaido 060-8604  JAPAN | Sapporo city general hospital IRB  13-1-1  Kita11jonishi  Chuo-ku  Sapporo, Hokkaido 060-8604  JAPAN |
|  |  |  |  |  |  |
| 1038 | Atsushi Kaneko |  | Yoshito Eto  Daihei Kida  Kiwamu Saito  Tomotaro Sato  Nobunori Takahashi  Kenichiro Tsuji  Masami Tsukamoto | National hospital Organization Nagoya Medical Center  4-1-1  Sannomaru, Naka-ku  Nagoya, Aichi 460-0001  JAPAN | National Hospital Organization Central Review Board  2-5-21  Higashigaoka  Meguro, Tokyo 152-0021  JAPAN |
|  |  |  |  |  |  |
| 1039 | Naoki Ishiguro |  | Koji Funahashi  Masatoshi Hayashi  Yasuhide Kanayama  Daizo Kato  Toshihisa Kojima  Hiroyuki Matsubara  Tomone Shioura | Nagoya University Hospital  65  Tsurumai-cho, Showa-ku  Nagoya, Aichi 466-8560  JAPAN | Nagoya University Hospital IRB  65  Tsurumai-cho  Showa-ku  Nagoya, Aichi 466-8560  JAPAN |
|  |  |  |  |  |  |
| 1041 | Tatsuya Atsumi |  | Yuichiro Fujieda  Tetsuya Horita  Hiroshi Kataoka  Masaru Kato  Takashi Kurita  Toshio Odani  Kotaro Ohtomo  Kenji Oku  Shinsuke Yasuda | Hokkaido University Hospital  5  Kita14jonishi, Kita-ku  Sapporo, Hokkaido 060-8648  JAPAN | Hokkaido University Hospital IRB  Kita 14, Nishi 5  Kita-ku  Sapporo, Hokkaido 060-8648  JAPAN |
|  |  |  |  |  |  |
| 1042 | Kazuhide Tanimura |  | Jun Fukae  Megumi Matsuhashi  Masato Shimizu | Hokkaido Medical Center for Rheumatic Diseases  3-1-45  1jyo, Kotoni, Nishi-ku  Sapporo, Hokkaido 063-0811  JAPAN | Hokkaido Medical Center for Rheumatic Diseases IRB  3-1-45  1jyo, Kotoni, Nishi-ku  Sapporo, Hokkaido, Japan 063-0811  JAPAN |
|  |  |  |  |  |  |
| 1043 | Kou Katayama |  | Takuya Ruike  Toshikazu Sato | Katayama Orthopedic Rheumatology Clinic  4-5-17  Toyooka13jo  Asahikawa, Hokkaido 078-8243  JAPAN | Toyooka Chuo Hospital IRB  Toyooka Chuo Hospital  7-2-1-5  Toyooka  Asahikawa-shi, Hokkaido 078-8237  JAPAN |
|  |  |  |  |  |  |
| 1044 | Takayuki Sumida |  | Daisuke Goto  Taichi Hayashi  Masanobu Horikoshi  Satoshi Ito  Yuya Kondo  Isao Matsumoto  Hiroshi Ogishima  Makoto Sugihara  Takeshi Suzuki  Youhei Takano  Hiroto Tsuboi  Naoto Umeda | Tsukuba University Hospital  2-1-1  Amakubo  Tsukuba, Ibaraki 305-8576  JAPAN | Tsukuba University Hospital IRB  2-1-1  Amakubo  Tsukuba, Ibaraki 305-8576  JAPAN |
|  |  |  |  |  |  |
| 1045 | Michishi Tsukano (Previous PI)  Mitsuru Sakaguchi |  | Syuichi Higashi  Toshio Kitamura  Hironori Kudo  Kunihiko Tomoda  Michishi Tsukano | Kumamoto Orthopaedic Hospital  1-15-7  Kuhonji  Kumamoto, Kumamoto 862-0976  JAPAN | Kumamoto Orthopaedic Hospital IRB  1-15-7  Kuhonji  Kumamoto, Kumamoto 862-0976  JAPAN |
|  |  |  |  |  |  |
| 1046 | Seizo Yamana |  | Mitsuhiro Iwahashi  Motoaki Kin  Keisuke Kobayashi  Rie Sasaki  Jiro Yamana | Higashihiroshima Memorial Hospital  2214  Yoshiyuki, Saijo-cho  Higashihiroshima, Hiroshima 739-0002  JAPAN | Higashihiroshima Memorial Hospital IRB  2214  Yoshiyuki  Saijo-cho  Higashihiroshima, Hiroshima 739-0002  JAPAN |
|  |  |  |  |  |  |
| 1047 | Daisuke Kawabata |  | Takao Fujii  Tsuneyo Mimori  Takaki Nojima  Koichiro Ohmura  Takashi Usui  Naoichiro Yukawa | Kyoto University Hospital  54  Shogoinkawahara-cho, Sakyo-ku  Kyoto, Kyoto 606-8507  JAPAN | Kyoto University Hospital IRB  54  Shogoinkawahara-cho  Sakyo-ku  Kyoto, Kyoto 606-8507  JAPAN |
|  |  |  |  |  |  |
| 1048 | Atsushi Kawakami |  | Katsumi Eguchi  Hiroaki Ida  Tomohiro Koga  Hideki Nakamura  Akitomo Okada  Tomoki Origuchi  Satoshi Yamasaki | Nagasaki University Hospital  1-7-1  Sakamoto  Nagasaki, Nagasaki 852-8501  JAPAN | Nagasaki University Hospital of Medicine and Dentistry IRB  1-7-1  Sakamoto  Nagasaki, Nagasaki 852-8501  JAPAN |
|  |  |  |  |  |  |
| 1049 | Hajime Sano |  | Naoto Azuma  Naoaki Hashimoto  Tsuyoshi Iwasaki  Masayasu Kitano  Kiyoshi Matsui  Mai Morimoto  Aki Nishioka  Mika Okabe  Masahiro Sekiguchi | The Hospital of Hyogo College of Medicine  1-1  Mukogawa-cho  Nishinomiya, Hyogo 663-8501  JAPAN | The Hospital of Hyogo College of Medicine IRB  Institutional Review Board  1-1 Mukogawa-cho  Nishinomiya, Hyogo 663-8501  JAPAN |
|  |  |  |  |  |  |
| 1050 | Hiroshi Inoue |  | Takeo Sakurai  Yasuyuki Tamura  Yoshihiro Yamashina | Inoue Hospital  55  Torimachi  Takasaki, Gunma 370-0053  JAPAN | Inoue Hospital IRB  Inoue Hospital  55  toorimachi  Takasaki, Gunma 370-0053  JAPAN |
|  |  |  |  |  |  |
| 1052 | Tatsuo Hirose |  | Toshiharu Kakimoto | Saitama city hospital  2460  Mimuro, Midori-ku  Saitama, Saitama 336-8522  JAPAN | Institutional Review Board of Keihin Chuo Clinic,Hisamitsu Clinic and Masabayashi Clinic  2-20-10,Hitotsuya  Adachi-ku, Tokyo 121-0075  JAPAN |
|  |  |  |  |  |  |
| 1053 | Junji Chiba |  | Koichiro Hayata  Yasuo Inoue  Katsuaki Kanbe  Atsushi Nakamura | Tokyo Women's Medical University Medical Center East  2-1-10  Nishiogu  Arakawa-ku, Tokyo 116-8567  JAPAN | Tokyo Women's Medical University Hospital IRB  8-1  Kawada-cho  Shinjyuku-ku, Tokyo 162-8666  JAPAN |
|  |  |  |  |  |  |
| 1054 | Kenshi Higami |  | Satomi Higami | Higami hospital  701  Kuzumoto-cho  Kashihara, Nara 634-0007  JAPAN | Hanna Hospital Institutional Review Board  1-1-31  Terakawa  Daito-shi  Osaka,  JAPAN |
|  |  |  |  |  |  |
| 1055 | Teruaki Nakano |  |  | St. Mary's Hospital  422  Tsubukuhonmachi  Kurume, Fukuoka 830-8543  JAPAN | Institutional Review Board of St. Mary's Hospital  422  Tsubukuhonmachi  Kurume-shi, Fukuoka  JAPAN |
|  |  |  |  |  |  |
| 1056 | Yasuhiko Hirabayashi |  | Sanae Shimura | Hikarigaoka Spellman Hospital  6-7-1  higashisendai  miyagino-ku  Sendai, Miyagi 983-0833  JAPAN | wakaba hospital IRB  wakaba hospital IRB  609  tomiya  Sakado, Saitama 350-0208  JAPAN |
|  |  |  |  |  |  |
| 1057 | Masato Yagita |  | Saori Hatachi  Akira Ohnishi | The Tazuke Kofukai Medical Research Institute Kitano Hospital  2-4-20  Ohgimachi, Kita-ku  Osaka, Osaka 530-8480  JAPAN | Kitano Hospital IRB  2-4-20  Ohgi-machi, Kita-ku  Osaka, Osaka 530-8480  JAPAN |
|  |  |  |  |  |  |
| 1058 | Yutaka Kawahito |  | Hidetaka Ishino  Masataka Kohno  Yasunori Tsubouchi  Aihiro Yamamoto | University Hospital, Kyoto Prefectural University of Medicine  465  Kajiicho  Hirokoji-Agaru, Kawaramachi-dori, kamigyo-ku  Kyoto, Kyoto 602-8566  JAPAN | University Hospital, Kyoto Prefectural University of Medicine¿IRB  465  Kajiicho, Hirokoji-Agaru,  Kawaramachi-dori, Kamigyo-ku  Kyoto, Kyoto 602-8566  JAPAN |
|  |  |  |  |  |  |
| 1059 | Takuya Sawabe |  | Hiroshi Miyagawa  Asako Oguma  Jyunji Otsuka  Soushi Takahashi | Hiroshima Red Cross Hospital & Atomic-bomb Survivors Hospital  1-9-6  Sendamati, Naka-ku  Hiroshima-city, Hiroshima 730-8619  JAPAN | Hiroshima Red Cross & Atomic-Bomb Surviors Hospital IRB  1-9-6  Senda-machi  Naka-ku  Hiroshima, Hiroshima 730-8619  JAPAN |
|  |  |  |  |  |  |
