# Supplementary material for: Changes in serum creatinine in patients with active rheumatoid arthritis treated with tofacitinib: results from clinical trials
Source: Arthritis Res Ther. 2014 Jul 25;16(4):R158. doi: 10.1186/ar4673 (PMC4220634; doi:10.1186/ar4673)
Supplement: Supplementary file 8 — Additional file 8: List of Investigators and Corresponding Ethics Committees or Institutional Review Boards for the LTE A3921041 study. (DOC 152 KB) [file 13075_2013_4378_MOESM8_ESM.doc]

# 16.1.4.1 LIST OF INVESTIGATORS AND CORRESPONDING ETHICS COMMITTEES OR INSTITUTIONAL REVIEW BOARDS

## Japan

**Coordinating Investigators:**

<None Entered>

| **Center** | **Principal Investigator** | **Co-Investigator(s)** | **Sub-Investigator(s)** | **Address(es)** | **Institutional Review Board or Ethics Committee Address(es)** |
| --- | --- | --- | --- | --- | --- |
|  |  |  |  |  |  |
| 1001 | Dr. Ryutaro Matsumura |  | Takuya Nakazawa  Tomo Suzuki | National Hospital Organization Chiba-East Hospital  673  Nitona-chou  Chuou-ku  Chiba, Chiba 260-8712  JAPAN | National Hospital Organization Chiba-East Hospital  673  Nitona-chou  Chuou-ku  Chiba, Chiba 260-8712  JAPAN |
|  |  |  |  |  |  |
| 1002 | Dr. Kazuhiko Yamamoto |  | Keishi Fujio  Noboru Hagino  Hiroko Kanda  Kimito Kawahata  Kanae Kubo  Hirofumi Shoda  Shuji Sumitomo | The University of Tokyo Hospital  7-3-1  Hongo  Bunkyo-ku, Tokyo 113-8655  JAPAN | The University of Tokyo hospital IRB  7-3-1  Hongo  Bunkyo-ku, Tokyo 113-8655  JAPAN |
|  |  |  |  |  |  |
| 1003 | Dr. Shigeto Tohma |  | Hiroshi Furukawa  Hidekazu Futami  Atsushi Hashimoto  Tatsuoh Ikenaka  Kanako Iwata  Toshihiro Matsui  Dr. Hisanori Nakayama  Yuko Okazaki  Kota Shimada  Hirokazu Takaoka | National Hospital Organization Sagamihara National Hospital  18-1  Sakuradai, Minami-ku  Sagamihara, Kanagawa 252-0392  JAPAN | National Hospital Organization Sagamihara National Hospital  18-1  Sakuradai, Minami-ku  Sagamihara, Kanagawa 252-0392  JAPAN |
|  |  |  |  |  |  |
| 1004 | Dr. Takeshi Kuroda (Previous PI)  Masaaki Nakano |  | Daisuke Kobayashi  Dr. Takeshi Kuroda  Shuichi Murakami  Takeshi Nakatsue  Hiroe Sato  Yoko Wada | Niigata University Medical & Dental Hospital  1-754  Asahimatchidoori  Chuou-ku  Niigata, Niigata 951-8520  JAPAN | Niigata University Medical & Dental Hospital IRB  754  Ichibancho  Asahimachidoori  Niigata, Niigata 951-8520  JAPAN |
|  |  |  |  |  |  |
| 1005 | Dr. Shiro Ohshima |  | Yoshinori Harada  Yoshinori Katada  Yuichi Maeda  Masato Matsushita  Yukihiko Saeki  Eriko Tanaka  Soichiro Tsuji  Akane Watanabe  Maiko Yoshimura | National Hospital Organization Osaka Minami Center  2-1  Kidohigashimachi  Kawachinagano, Osaka 586-8521  JAPAN | National Hospital Organization Osaka Minami Center  2-1  Kidohigashimachi  Kawachinagano,  Osaka, Japan 586-8521  JAPAN |
|  |  |  |  |  |  |
| 1006 | Kazuyoshi Saito  Yoshiya Tanaka (Previous PI) |  | Dr. Eri Hirakawa  Dr. Shintaro Hirata  Shigeru Iwata  Dr. Takayuki Katsuyama  Satoshi Kubo  Dr. Ippei Miyagawa  Kazuhisa Nakano  Shingo Nakayamada  Masao Nawata  Koshiro Sonomoto  Yoshiya Tanaka  Dr. Shizuyo Tsujimura  Kunihiro Yamaoka  Dr. Maiko Yoshikawa  Dr. Sounosuke Yukawa  Dr. Naoki Yunoue | University of Occupational and Environmental Health Hospital  1-1  Iseigaoka  Yahatanishi-ku  Kitakyusyu, Fukuoka 807-8555  JAPAN | University of Occupational and Environmental Health Hospital IRB  1-1  Iseigaoka  Yahata-Nishi-ku  Kita-Kyushu,, Fukuoka 807-8555  JAPAN |
|  |  |  |  |  |  |
| 1007 | Dr. Hisashi Yamanaka  Naoyuki Kamatani (Previous PI) |  | Sayumi Baba  Dr. Chikako Fukasawa  Dr. Takefumi Furuya  Dr. Masako Hara  Daisuke Hoshi  Dr. Naomi Ichikawa  Dr. Katsunori Ikari  Dr. Takuji Iwamoto  Dr. Tokiko Kanno  Dr. Yasushi Kawaguchi  Dr. Mariko Kitahama  Dr. Tsuyoshi Kobashigawa  Dr. Yumi Koseki  Dr. Shigeru Kotake  Dr. Shigeki Momohara  Dr. Ayako Nakajima  Dr. Yuki Nanke  Hiroshi Okamoto  Dr. Seiji Saito  Dr. Eri Sato  Yohei Seto  Kumi Shidara  Dr. Makoto Soejima  Kae Takagi  Dr. Atsuo Taniguchi  Dr. Chihiro Terai  Dr. So Tsukahara  Wako Urano | Tokyo Women's Medical University, Institute of Rheumatology  10-22  Kawada-cho  Shinjyuku-ku, Tokyo 162-0054  JAPAN | Tokyo Women's Medical University Hospital IRB  8-1  Kawada-cho  Shinjyuku-ku, Tokyo 162-8666  JAPAN |
|  |  |  |  |  |  |
| 1008 | Dr. Hitoshi Kohsaka  Dr. Nobuyuki Miyasaka (Previous PI) |  | Dr. Masayoshi Harigai  Shinya Hirata  Hideyuki Iwai  Dr. Ryuji Koike  Dr. Tetsuo Kubota  Yoshishige Miyabe  Toshihiro Nanki  Dr. Yoshinori Nonomura  Dr. Kazuki Takada  Dr. Michi Tanaka  Dr. Waka Yokoyama | Tokyo Medical And Dental University Hospital, Faculty of Medicine  1-5-45  Yushima  Bunkyo-ku, Tokyo 113-8519  JAPAN | Tokyo Medical And Dental University Hospital, Faculty of Medicine IRB  1-5-45  Yushima  Bunkyo-ku,, Tokyo 113-8519  JAPAN |
|  |  |  |  |  |  |
| 1009 | Dr. Koichi Amano  Tsutomu Takeuchi (Previous PI) |  | Hayato Nagasawa  Tsutomu Takeuchi | Saitama Medical Center  1981  Kamoda  Kawagoe-shi, Saitama 350-8550  JAPAN | Institutional Review Board (IRB) of Saitama Medical Center, Saitama Medical University  1981  Kamoda, Kawagoe-shi  Saitama, Japan 350-8550  JAPAN |
|  |  |  |  |  |  |
| 1010 | Hajime Yamagata  Hajime Yamagata (Previous PI)  Junichi Kaburaki (Previous PI) |  | Shinichiro Iwata  Hideaki Murakami  Hiroshi Usui | National Hospital Organization MURAYAMA Medical Center  2-37-1  Gakuen  Musashimurayama-shi, Tokyo 208-0011  JAPAN | National Hospital Organization MURAYAMA Medical Center IRB  2-37-1  Gakuen  Musashimurayama-shi,, Tokyo 208-0011  JAPAN |
|  |  |  |  |  |  |
| 1011 | Shunsuke Mori |  | Junji Hamamoto  Fumiya Imamura  Dr. Esaki Toshihiro  Akihisa Yamashita  Kensuke Yonemura | Kumamoto Saishunso National Hospital  2659  Suya  Koushi, Kumamoto 861-1196  JAPAN | Kumamoto Saishunso National Hospital IRB  2659  Suya,  Koushi-shi,, Kumamoto 861-1196  JAPAN |
|  |  |  |  |  |  |
| 1012 | Shuji Ohta |  | Soichi Hotta  Seiji Mogi | Taga General Hospital  2-1-2  Kokubu-cho  Hitachi-shi, Ibaraki 316-0035  JAPAN | Taga General Hospital IRB  2-1-2,  Kokubu-cho,  Hitachi-shi,, Ibaraki 316-0035  JAPAN |
|  |  |  |  |  |  |
| 1013 | Hiroshi Tsuda |  | Tomoko Watanabe  Kwang Seok Yang | Juntendo Tokyo Koto Geriatric Medical Center  3-3-20  Shinsuna  Koto-ku, Tokyo 136-0075  JAPAN | Juntendo Tokyo Koto Geriatric Medical Center IRB  3-3-20  shinnsuna,  koutou-ku,, Tokyo 136-0075  JAPAN |
|  |  |  |  |  |  |
| 1014 | Hisaji Ohshima |  | Kumiko Akiya | National Hospital Organization Tokyo Medical Center  2-5-1  Higashigaoka  Meguro-ku, Tokyo 152-8902  JAPAN | National Hospital Organization Tokyo Medical Center IRB  2-5-1  Higashigaoka  Meguro-ku, Tokyo 152-8902  JAPAN |
|  |  |  |  |  |  |
| 1015 | Kenjiro Yamanaka |  | Yoshinori Kanai  Soichiro Nakano | Sasaki Foundation Kyoundo Hospital  1-8  Kandasurugadai  Chiyoda-ku, Tokyo 101-0062  JAPAN | Sasaki Foundation Kyoundo Hospital IRB  1-8  kandasurugadai,  chiyoda-ku,, Tokyo 101-0062  JAPAN |
|  |  |  |  |  |  |
| 1016 | Eiichi Suematsu |  | Yukio Esaki  Goh Hirata  Yoshiro Horai  Motoko Ishida  Nobutaka Kaibara  Hisaaki Miyahara  Tomoya Miyamura  Masanobu Oishi  Masahiro Yamamoto | National Hospital Organization Kyushu Medical Center  1-8-1  Jigyohama  Chuo-ku  Fukuoka, Fukuoka 810-8563  JAPAN | National Hospital Organization Kyushu Medical Center IRB  1-8-1  Jigyohama  Chuo-ku  Fukuoka, Fukuoka 810-8563  JAPAN |
|  |  |  |  |  |  |
| 1017 | Yasuhiko Munakata (Previous PI)  Yukio Sato |  | Kazuyuki Honda  Naoko Misu  Naruhiko Takasawa  Shigeru Wakatsuki | Sendai Taihaku Hospital  1-12-26 Tomizawa Taihakuku Sendai-city  Miyagi, 982-0032  JAPAN | NS Clinic Institutional Review Board  2-26-9  Myojincho  Hachioji  Tokyo, Japan 192-0046  JAPAN |
|  |  |  |  |  |  |
| 1018 | Yoshinari Takasaki |  | Hirofumi Amano  Shouseki Lee  Ran Matsudaira  Masakazu Matsushita  Shinji Morimoto  Hitoshi Ogasawara  Michihiro Ogasawara  Kurisu Tada  Naoto Tamura  Ken Yamaji | Juntendo University Hospital  3-1-3  Hongo  Bunkyo-ku, Tokyo 113-8431  JAPAN | Juntendo University Hospital IRB  Juntendo University Hospital  3-1-3  Hongo  Bunkyo-ku, Tokyo 113-8431  JAPAN |
|  |  |  |  |  |  |
| 1019 | Hirobumi Kondo (Previous PI)  Toshimichi Matsui |  | Kenta Hoshi  Hirobumi Kondo  Takeo Kudo  Hide Nagaba  Hide Nagaba | Kitasato University Kitasato Institute Medical Center Hospital  6-100  Arai  Kitamoto, Saitama 364-8501  JAPAN | Kitasato University Kitasato Institute Medical Center Hospital IRB  6-100 Arai  Kitamoto, Saitama 364-8501  JAPAN |
|  |  |  |  |  |  |
| 1021 | Katsumi Chiba |  |  | Fukusima Daiichi Hospital  16-2  Narude  Kitasawamata-aza  Fukusima, Fukusima 960-8251  JAPAN | fukushimadaiichi hospital IRB  16-2  Narude  Kitasawamata-aza  Fukushima, Fukushima 960-8251  JAPAN |
|  |  |  |  |  |  |
| 1022 | Yasushi Nawata |  | Kazuhiro Hamasato  Shigekazu Takahashi | Chiba-ken Saiseikai Narashino Hospital  1-1-1  Izumi-cho  Narashino, Chiba 275-8580  JAPAN | Chiba-ken Saiseikai Narashino Hospital IRB  1-1-1  Izumi-cho  Narashino, Chiba 275-8580  JAPAN |
|  |  |  |  |  |  |
| 1023 | Akihiro Yamaguchi |  | Noboru Hagino  Yukiko Iwasaki | Fukuhara Hospital  2-8-16  Kitazawa  Setagaya-ku, Tokyo 155-0031  JAPAN | Sone Clinic IRB  3-31-1  Shinjuku  Shinjuku-ku, Tokyo 160-0022  JAPAN |
|  |  |  |  |  |  |
| 1025 | Yuji Yamanishi |  |  | Hiroshima Rheumatology Clinic  10-13  Teppo-cho  Naka-ku  Hiroshima, Hiroshima 730-0017  JAPAN | Sone Clinic IRB  sone clinic IRB  3-32-8  Shinjuku  Shinjuku-ku, Tokyo-to 160-0022  JAPAN |
|  |  |  |  |  |  |
| 1026 | Shuji Nagano  Toshiyuki Ota (Previous PI)  Yoshinobu Koyama (Previous PI) |  | Kenji Fujii  Toshiyuki Ota  Ayumi Uchino | Aso Iizuka Hospital  3-83  Yoshio-machi  Iiduka, Fukuoka 820-8505  JAPAN | Aso Iizuka Hospital  3-83  Yoshio-machi  Iiduka, Fukuoka 820-8505  JAPAN |
|  |  |  |  |  |  |
| 1027 | Eisuke Shono |  |  | SHONO Rheumatism Clinic  1-10-27  Nishijin  Sawara-ku, Fukuoka 814-0002  JAPAN | Sone Clinic IRB  sone clinic IRB  3-32-8  Shinjuku  Shinjuku-ku, Tokyo-to 160-0022  JAPAN |
|  |  |  |  |  |  |
| 1028 | Tomomi Tsuru |  | Takashi Etoh  Masayoshi Goto  Yasushi Inoue  Misato Nakagawa  Hitoshi Nakashima  Masanari Shiramoto  Kayo Taira | Medical Co.LTA PS Clinic  Random Square 8F/6-18  Tenyamachi  Hakata-ku  Fukuoka, Fukuoka 812-0025  JAPAN | Medical Co. LTA Kyushu clinical pharmacology reserch clinic IRB  2-13-16  jigyou  Chuuo-ku, Fukuoka 810-0064  JAPAN |
|  |  |  |  |  |  |
| 1029 | Kiyoshi Migita |  | Yasumori Izumi  Taiichiro Miyashita  Satoru Motokawa  Tadayoshi Ohno  Takafumi Torigoshi | National Hospital Organization Nagasaki Medical Center  2-1001-1  Kubara  Ohmura, Nagasaki 856-0835  JAPAN | National Hospital Organization Central Review Board  2-5-21  Higashigaoka  Meguro, Tokyo 152-0021  JAPAN |
|  |  |  |  |  |  |
| 1030 | Yukitaka Ueki |  | Kunihiro Ichinose  Nozomi Iwanaga  Hironobu Sato  Kaoru Terada  Satoshi Yamasaki | Sasebo Chuo Hospital  15  Yamato-cho  Sasebo, Nagasaki 857-1195  JAPAN | Sasebo Chuo Hospital IRB  Sasebo Chuo Hospital  15  Yamato-cho  Sasebo, Nagasaki 857-1195  JAPAN |
|  |  |  |  |  |  |
| 1031 | Motohiro Oribe |  |  | A Medical Corporation Oribe Rheumatism Internist Clinic  1-8-15  Higasiomichi  Oita, Oita 870-0823  JAPAN | Sone Clinic IRB  sone clinic IRB  3-32-8  Shinjuku  Shinjuku-ku, Tokyo-to 160-0022  JAPAN |
|  |  |  |  |  |  |
| 1032 | Dr. Takao Sugiyama |  | Masaaki Furukawa  Makoto Sueishi  Toyohiko Sugimoto | Shimoshizu National Hospital  934-5  Shikawatashi  Yotukaidou, Chiba 284-0003  JAPAN | National Hospital Organization Central Review Board  2-5-21  Higashigaoka  Meguro, Tokyo 152-0021  JAPAN |
|  |  |  |  |  |  |
| 1033 | Yojiro Kawabe |  | Koichiro Aratake  Fumiko Tanaka | Ureshino Medical Center  2436  Ooaza Shimojyukuhei  Ureshino-machi  Ureshino, Saga 843-0393  JAPAN | National Hospital Organization Central Review Board  2-5-21  Higashigaoka  Meguro, Tokyo 152-0021  JAPAN |
|  |  |  |  |  |  |
| 1035 | Motokazu Kai (Previous PI)  Shigenori Tamaki (Previous PI)  Takeshi Nagakura |  | Yumiko Asanuma  Motokazu Kai  Hideki Kotera  Yoshihiro Miura  Kunikazu Ogawa  Shigenori Tamaki  Ikuko Tanaka  Masaru Tanaka | National hospital Organization Mie Chuou Medical Center  2158-5  Myojin-Cho, Hisai  Tsu, Mie 514-1101  JAPAN | National Hospital Organization Central Review Board  2-5-21  Higashigaoka  Meguro, Tokyo 152-0021  JAPAN |
|  |  |  |  |  |  |
| 1036 | Masakazu Kondo |  |  | Kondo clinic for rheumatism and orthopaedics  3-10-11  Tenjin, Chuo-ku  Fukuoka, Fukuoka 810-0001  JAPAN | Haradoi Hospital IRB  6-40-8  Aoba  Higashi-ku  Fukuoka, Fukuoka 813-0025  JAPAN |
|  |  |  |  |  |  |
| 1037 | Masaya Mukai |  | Makoto Kondo | Sapporo city general hospital  13-1-1  Kita11jonishi, Chuo-ku  Sapporo, Hokkaido 060-8604  JAPAN | Sapporo city general hospital IRB  13-1-1  Kita11jonishi  Chuo-ku  Sapporo, Hokkaido 060-8604  JAPAN |
|  |  |  |  |  |  |
| 1038 | Atsushi Kaneko |  | Yoshito Eto  Hisato Ishikawa  Daihei Kida  Kiwamu Saito  Tomotaro Sato  Nobunori Takahashi | National hospital Organization Nagoya Medical Center  4-1-1  Sannomaru, Naka-ku  Nagoya, Aichi 460-0001  JAPAN | National Hospital Organization Central Review Board  2-5-21  Higashigaoka  Meguro, Tokyo 152-0021  JAPAN |
|  |  |  |  |  |  |
| 1039 | Naoki Ishiguro (Previous PI)  Toshihisa Kojima |  | Koji Funahashi  Masahiro Hanabayashi  Yosuke Hattori  Masatoshi Hayashi  Yasuhide Kanayama  Daizo Kato  Nobunori Takahashi | Nagoya University Hospital  65  Tsurumai-cho, Showa-ku  Nagoya, Aichi 466-8560  JAPAN | Nagoya University Hospital IRB  65  Tsurumai-cho  Showa-ku  Nagoya, Aichi 466-8560  JAPAN |
|  |  |  |  |  |  |
| 1041 | Tatsuya Atsumi |  | Toshiyuki Bohgaki  Yuichiro Fujieda  Tetsuya Horita  Yusaku Kanetsuka  Hiroshi Kataoka  Masaru Kato  Michihito Kono  Takashi Kurita  Ikuma Nakagawa  Atsushi Noguchi  Toshio Odani  Kenji Oku  Kotaro Otomo  Haruki Shida  Toshiyuki Watanabe  Shinsuke Yasuda | Hokkaido University Hospital  5  Kita14jonishi, Kita-ku  Sapporo, Hokkaido 060-8648  JAPAN | Hokkaido University Hospital IRB  Kita 14, Nishi 5  Kita-ku  Sapporo, Hokkaido 060-8648  JAPAN |
|  |  |  |  |  |  |
| 1042 | Kazuhide Tanimura |  | Jun Fukae  Masato Isobe  Megumi Matsuhashi  Masato Shimizu | Hokkaido Medical Center for Rheumatic Diseases  3-1-45  1jyo, Kotoni, Nishi-ku  Sapporo, Hokkaido 063-0811  JAPAN | Hokkaido Medical Center for Rheumatic Diseases IRB  3-1-45  Ichijo  Kotoni, Nishi-ku  Sapporo, Hokkaido 063-0811  JAPAN |
|  |  |  |  |  |  |
| 1043 | Kou Katayama |  | Toshikazu Sato | Katayama Orthopedic Rheumatology Clinic  4-5-17  Toyooka13jo  Asahikawa, Hokkaido 078-8243  JAPAN | Toyooka chuo hospital  2-1-5  Toyooka7jo  Asahikawa, Hokkaido 078-8237  JAPAN |
|  |  |  |  |  |  |
| 1044 | Takayuki Sumida |  | Taichi Hayashi  Masanobu Horikoshi  Yuya Kondo  Isao Matsumoto  Hiroshi Ogishima  Makoto Sugihara  Takeshi Suzuki  Hiroto Tsuboi | Tsukuba University Hospital  2-1-1  Amakubo  Tsukuba, Ibaraki 305-8576  JAPAN | Tsukuba University Hospital IRB  2-1-1  Amakubo  Tsukuba, Ibaraki 305-8576  JAPAN |
|  |  |  |  |  |  |
| 1045 | Michishi Tsukano  Michishi Tsukano (Previous PI)  Mitsuru Sakaguchi (Previous PI) |  | Toshio Kitamura  Hironori Kudo  Mitsuru Sakaguchi  Kunihiko Tomoda | Kumamoto Orthopaedic Hospital  1-15-7  Kuhonji  Kumamoto, Kumamoto 862-0976  JAPAN | Kumamoto Orthopaedic Hospital IRB  1-15-7  Kuhonji  Kumamoto, Kumamoto 862-0976  JAPAN |
|  |  |  |  |  |  |
| 1046 | Mitsuhiro Iwahashi  Seizo Yamana (Previous PI) |  | Teppei Hashimoto  Motoaki Kin  Keisuke Kobayashi  Rie Sasaki  Jiro Yamana  Seizo Yamana | Higashihiroshima Memorial Hospital  2214  Yoshiyuki, Saijo-cho  Higashihiroshima, Hiroshima 739-0002  JAPAN | Higashihiroshima Memorial Hospital IRB  2214  Yoshiyuki  Saijo-cho  Higashihiroshima, Hiroshima 739-0002  JAPAN |
|  |  |  |  |  |  |
| 1047 | Daisuke Kawabata (Previous PI)  Hajime Yoshifuji |  | Takao Fujii  Tsuneyo Mimori  Takaki Nojima  Koichiro Ohmura  Takashi Usui  Naoichiro Yukawa | Kyoto University Hospital  54  Shogoinkawahara-cho, Sakyo-ku  Kyoto, Kyoto 606-8507  JAPAN | Kyoto University Hospital Institutional Review Board  54  Shogoinkawahara-cho  Sakyo-ku  Kyoto, Kyoto 606-8507  JAPAN |
|  |  |  |  |  |  |
| 1048 | Atsushi Kawakami |  | Katsumi Eguchi  Yoshiro Horai  Kunihiro Ichinose  Naoki Iwamoto  Shinya Kawashiri  Tomohiro Koga  Hideki Nakamura  Yoshikazu Nakashima  Akitomo Okada  Tomoki Origuchi  Takahisa Suzuki  Mami Tamai  Satoshi Yamasaki | Nagasaki University Hospital of Medicine and Dentistry  1-7-1  Sakamoto  Nagasaki, Nagasaki 852-8501  JAPAN | Nagasaki University Hospital IRB  1-7-1  Sakamoto  Nagasaki, Nagasaki 852-8501  JAPAN |
|  |  |  |  |  |  |
| 1049 | Hajime Sano |  | Naoto Azuma  Naoaki Hashimoto  Tsuyoshi Iwasaki  Masayasu Kitano  Kiyoshi Matsui  Mai Morimoto  Aki Nishioka  Mika Okabe  Masahiro Sekiguchi | The Hospital of Hyogo College of Medicine  1-1  Mukogawa-cho  Nishinomiya, Hyogo 663-8501  JAPAN | The Hospital of Hyogo College of Medicine IRB  Institutional Review Board  1-1 Mukogawa-cho  Nishinomiya, Hyogo 663-8501  JAPAN |
|  |  |  |  |  |  |
| 1050 | Hiroshi Inoue |  | Takeo Sakurai  Yasuyuki Tamura  Yoshihiro Yamashina | Inoue Hospital  55  Torimachi  Takasaki, Gunma 370-0053  JAPAN | Inoue Hospital IRB  Inoue Hospital  55  toorimachi  Takasaki, Gunma 370-0053  JAPAN |
|  |  |  |  |  |  |
| 1052 | Tatsuo Hirose |  | Toshiharu Kakimoto | Saitama city hospital  2460  Mimuro, Midori-ku  Saitama, Saitama 336-8522  JAPAN | Keihin central clinic, Hisamitsu clinic and Masabayashi clinic IRB  2-20-10  Hitotsuya  Adachiku, Tokyo  JAPAN |
|  |  |  |  |  |  |
| 1053 | Junji Chiba |  | Koichiro Hayata  Yasuo Inoue  Katsuaki Kanbe  Atsushi Nakamura | Tokyo Women's Medical University Medical Center East  2-1-10  Nishiogu  Arakawa-ku, Tokyo 116-8567  JAPAN | Tokyo Women's Medical University Hospital IRB  8-1  Kawada-cho  Shinjyuku-ku, Tokyo 162-8666  JAPAN |
|  |  |  |  |  |  |
| 1054 | Kenshi Higami |  | Satomi Higami | Higami hospital  701  Kuzumoto-cho  Kashihara, Nara 634-0007  JAPAN | Hanna Hospital Institutional Review Board  1-1-31  Terakawa  Daito-shi  Osaka,  JAPAN |
|  |  |  |  |  |  |
| 1055 | Dr. Teruaki Nakano |  |  | St. Mary's Hospital  422  Tsubukuhonmachi  Kurume, Fukuoka 830-8543  JAPAN | Institutional Review Board of St. Mary's Hospital  422  Tsubukuhonmachi  Kurume-shi, Fukuoka  JAPAN |
|  |  |  |  |  |  |
| 1056 | Yasuhiko Hirabayashi |  | Sanae Shimura | Hikarigaoka Spellman Hospital  6-7-1  higashisendai  miyagino-ku  Sendai, Miyagi 983-0833  JAPAN | wakaba hospital IRB  wakaba hospital IRB  609  tomiya  Sakado, Saitama 350-0208  JAPAN |
|  |  |  |  |  |  |
| 1057 | Masato Yagita |  | Saori Hatachi  Yoshinobu Matsuura  Isao Murakami  Miwa Nisida | The Tazuke Kofukai Medical Research Institute Kitano Hospital  2-4-20  Ohgimachi, Kita-ku  Osaka, Osaka 530-8480  JAPAN | Kitano Hospital IRB  2-4-20  Ohgi-machi, Kita-ku  Osaka, Osaka 530-8480  JAPAN |
|  |  |  |  |  |  |
| 1058 | Yutaka Kawahito |  | Hidetaka Ishino  Masataka Kohno  Aihiro Yamamoto | University Hospital, Kyoto Prefectural University of Medicine  465  Kajiicho  Hirokoji-Agaru, Kawaramachi-dori, kamigyo-ku  Kyoto, Kyoto 602-8566  JAPAN | University Hospital, Kyoto Prefectural University of Medicine IRB  465  Kajiicho, Hirokoji-Agaru,  Kawaramachi-dori, Kamigyo-ku  Kyoto, Kyoto 602-8566  JAPAN |
|  |  |  |  |  |  |
| 1059 | Takuya Sawabe |  | Hiroshi Miyagawa  Asako Oguma  Jyunji Otsuka  Soushi Takahashi | Hiroshima Red Cross Hospital & Atomic-bomb Survivors Hospital  1-9-6  Sendamati, Naka-ku  Hiroshima-city, Hiroshima 730-8619  JAPAN | Hiroshima Red Cross & Atomic-Bomb Surviors Hospital IRB  1-9-6  Senda-machi  Naka-ku  Hiroshima, Hiroshima 730-8619  JAPAN |
|  |  |  |  |  |  |
| 1061 | Tsutomu Takeuchi |  | Yumiko Inoue  Yuko Kaneko  Jun Kikuchi  Yasushi Kondo  Masataka Kuwana  Hidekata Yasuoka | Keio University Hospital  35  Shinano-machi  Shinjuku-ku, Tokyo, Japan 160-8582  JAPAN | Keio University Hospital IRB  35  Shinano-machi  Shinjuku-ku, Tokyo 160-8582  JAPAN |
|  |  |  |  |  |  |
| 1062 | Dr Toshiaki Miyamoto |  | Rei Ito  Dr. Yuichiro Taguchi | Seirei Hamamatsu General Hospital  2-12-12  Naka-ku, Sumiyoshi  Hamamatsu, Shizuoka 430-8558  JAPAN | Hamamatsu Clinical Research Network Institutional Review Board  2-12-12  Naka-ku, Sumiyoshi  Hamamatsu, Shizuoka 430-8558  JAPAN |
|  |  |  |  |  |  |
