# Supplementary material for: Changes in serum creatinine in patients with active rheumatoid arthritis treated with tofacitinib: results from clinical trials
Source: Arthritis Res Ther. 2014 Jul 25;16(4):R158. doi: 10.1186/ar4673 (PMC4220634; doi:10.1186/ar4673)
Supplement: Supplementary file 9 — Additional file 9: List of Investigators and Corresponding Ethics Committees or Institutional Review Boards for the Phase 3 A3921044 study. (DOC 424 KB) [file 13075_2013_4378_MOESM9_ESM.doc]

# 16.1.4 LIST OF INVESTIGATORS AND CORRESPONDING ETHICS COMMITTEES OR INSTITUTIONAL REVIEW BOARDS

## Australia

**Coordinating Investigators:**

<None Entered>

| **Center** | **Principal Investigator** | **Co-Investigator(s)** | **Sub-Investigator(s)** | **Address(es)** | **Institutional Review Board or Ethics Committee Address(es)** |
| --- | --- | --- | --- | --- | --- |
|  |  |  |  |  |  |
| 1001 | Dr. David Nicholls (Previous PI)  Prof. Peter T. Nash  Prof. Peter T. Nash (Previous PI) |  | Dr. Frances Johnson  Dr. David Nicholls  Ms. Dale Shergold  Ms. Jan Smith  Dr. Susan Thackwray  Dr. Avgeania Voight | Rheumatology Research Unit Sunshine Coast  9-10 Maroochy Waters Shopping Centre  Denna Street  Maroochydore, QLD 4558  AUSTRALIA | Redcliffe-Caboolture Ethics Committee  Unit 1, Ground Floor, Redcliffe Hospital  Anzac Avenue  Redcliffe, Qld 4020  AUSTRALIA |
|  |  |  |  |  |  |
| 1002 | Dr. Maureen Rischmueller |  | Rachel Black  Dr. Simon Burnet  Dr. Sarah Downie-Doyle  Dr. Catherine Hill  Dr. Veera Katikireddi  Dr. Jem Ninan  Dr. Samuel Whittle | The Queen Elizabeth Hospital, Department of Rheumatology  28 Woodville Road  Woodville, SA 5011  AUSTRALIA | Central Northern Adelaide Health Service  Ethics of Human Research Committee  The Queen Elizabeth Hospital  28 Woodville Road  Woodville, SA 5011  AUSTRALIA |
|  |  |  |  |  |  |
| 1003 | Assoc. Prof. Stephen Hall |  | Dr. Vivienne Beckett  Dr Jill Bell  Dr Malcolm Clark  Dr Jennifer Davey  Dr. Marie Feletar  Dr. Andrew Gibson  Dr. James McDonald  Dr Nicole McKay  Dr Louise Murdoch  Dr. Veronique Sayag-Boukris | Emeritus Research  291 Wattletree Road  Malvern East, VIC 3145  AUSTRALIA | Cabrini Human Research Ethics Committee  183 Wattletree Road  Malvern, VIC 3144  AUSTRALIA |

## Brazil

**Coordinating Investigators:**

<None Entered>

| **Center** | **Principal Investigator** | **Co-Investigator(s)** | **Sub-Investigator(s)** | **Address(es)** | **Institutional Review Board or Ethics Committee Address(es)** |
| --- | --- | --- | --- | --- | --- |
|  |  |  |  |  |  |
| 1069 | Dr. Cristiano Augusto de Freitas Zerbini |  | Dr. Marta E. C. Bastos  Lina Oliveira de Carvalho  Dr. Wagner Ikehara  Dr. Luiz Carlos Latorre  Dr. Andrea Barranjard Vannucci Lomonte  Dr. Silvia Caroline Santana Moura  Dr. Maria Jose Nunes  Dr. Lenise B. Pieruccetti  Dr. Luiza Helena Coutinho Ribeiro  Dr. Raissa Gomes da Silva  Dr. Mariana G. Waisberg | CEPIC - Centro Paulista de Investigacao Clinica e Servicos Medicos Ltda  Rua Moreira e Costa 342  Sao Paulo, SP 04266-010  BRAZIL | Comite de Etica em Pesquisa em Seres Humanos do Hospital Heliopolis  Rua Conego Xavier 276  Sao Paulo, SP 04231-030  BRAZIL |
|  |  |  |  |  |  |
| 1070 | Dr. Antonio Carlos Ximenes |  | Dr. Rafael Navarrete Fernandez  Dr. Fabia M.G.P. Oliveira  Dr. Marcelo Pimenta  Dr. Bruno Nazeozeno Ribeiro | CIP - Centro Internacional de Pesquisas  Rua 9 B, 129 - 3 andar  Setor Oeste  Goiania, GO 74110-120  BRAZIL  CLINICA DE RAIOS X NABYH SALUM S/S - CLINICA SAO MATHEUS  Avenida República do Líbano, 1440  Goiania, GO 74115-030  BRAZIL | Comite de Etica em Pesquisa do Hospital Geral de Goiania - CEPHGG  Avenida Anhhaguera, 6479 - Setor Oeste  Goiania, GO 74110-010  BRAZIL |
|  |  |  |  |  |  |
| 1071 | Dr. Sebastiao C. Radominski |  | Dr. Vivian B. Coginotti  Dr. Sinara da Silva Freitas  Dr. Andreas Funke  Dr. Lucila Stange Rezende  Dr. Alexandre G. Tavares  Dr. David Cezar Titton | Centro de Estudos em Terapias Inovadoras-CETI  Rua Padre Camargo, 241  Alto da Glória  Curitiba, PR 80060-240  BRAZIL | Comitê de Ética em Pesquisa em Seres Humanos do Hospital das Clínicas da UFPR  Rua General Carneiro 181  Curitiba, PR 80060-900  BRAZIL |
|  |  |  |  |  |  |
| 1072 | Dr. Joao Carlos Tavares Brenol |  | Dr. Claiton Viegas Brenol  Dr. Odirlei A. Monticielo  Dr. Tamara M. Mucenic  Dr. Ricardo Machado Xavier | Hospital de Clinicas de Porto Alegre  Grupo de Pesquisa  Rua Ramiro Barcelos 2350 2 andar  sala 2202  Porto Alegre, RS 90035-903  BRAZIL  Hospital de Clinicas de Porto Alegre  Servico de Reumatologia  Rua Ramiro Barcelos, 2350- 6º andar - sala 645 A  Bom Fim  Porto Alegre, RS 90035-903  BRAZIL | Comite de Etica em Pesquisa em Seres Humanos do Hospital de Clinicas de Porto Alegre- HCPA  Rua Ramiro Barcelos 2350, 2 andar  Bom Fim  Porto Alegre, RS 90035-903  BRAZIL |
|  |  |  |  |  |  |
| 1073 | Dr. Ana Claudia Cauceglia Melazzi |  | Dr. Carolina A. Cabizuca  Dr. Alessandra S. M. F. Costa  Tonia L. Cunha  Dr. Adriana Danowski  Dr. Maria De Fatima Dias De Castro  Dr. Juliana Branco Dias  Dr. Luiz Henrique de Gregorio  Dr. Angela Frazao Linhares Hahn  Daniel Barretto Kendler  Dr. Paulo Gustavo Sampaio Lacativa  Dr. Flavia S. Lessa  Dr. Tarso Lameri Sant anna Mosci  Dr. Renata Alexandra Calixto Pinheiro  Dr. Luis Augusto Tavares Russo  Dr. Pedro Moitrel Schwarts  Dr. Priscila Geller Wolff | CCBR Brasil  Centro de Pesquisas e Analises Clinicas Ltda.  Rua Mena Barreto, 33  Rio de Janeiro, RJ 22271-100  BRAZIL | Comite de Etica em Pesquisa do Hospital Pro Cardiaco Pronto Socorro Cardiologico/PROCEP  Rua Dona Mariana, 219  Rio de Janeiro, RJ 22280-000  BRAZIL |
|  |  |  |  |  |  |
| 1074 | Dr. Mauro W. Keiserman |  | Melissa Claudia Bisi  Dr. Briele Keiserman  Dr. Tatiana Karenini Muller  Dr. Maria Mercedes Picarelli  Dr. Aline Defaveri do Prado  Caroline Z. Xavier de Freitas | Hospital Sao Lucas da PUCRS  Av. Ipiranga, 6690 - 4 andar  Porto Alegre, RS 90610-000  BRAZIL | Comite de Etica em Pesquisa da Pontificia Universidade Catolica do Rio Grande do Sul  Av. ipiranga 6690-Conj.314 -3 andar  Jardim Botanico  Porto Alegre, RS 90610-000  BRAZIL |
|  |  |  |  |  |  |
| 1075 | Dr. Luciana Teixeira Pinto |  | Camila Cristhine Bucchi  Dr. Marise Lazaretti Castro  Patricia Muszkat  Dr. Camila Albero Schiavon  Jeane Jeong Hoon Yang | Instituto de Pesquisa Clínica e Medicina Avançada  Praca Americo Jacomino, 55  Vila Madalena  Sao Paulo, SP 05437-010  BRAZIL | Comite de Etica em Pesquisa do Instituto de Infectologia Emilio Ribas  Av. Dr. Arnaldo, 165  Sao Paulo, SP 01246-900  BRAZIL |
|  |  |  |  |  |  |

## Bulgaria

**Coordinating Investigators:**

<None Entered>

| **Center** | **Principal Investigator** | **Co-Investigator(s)** | **Sub-Investigator(s)** | **Address(es)** | **Institutional Review Board or Ethics Committee Address(es)** |
| --- | --- | --- | --- | --- | --- |
|  |  |  |  |  |  |
| 1135 | Dr. Boycho Oparanov |  | Dr. Borislava Angelova Ilchova  Dr. Raycho Raychev  Dr. Ignat Zhutev | MBAL na Voennomeditsinska Akademia - Sofia, Klinika po Revmatologia i Kardiologia  MMA HAT Sofia  Ul. Georgi Sofiyski 3  Sofia, 1606  BULGARIA | Ethics Committee for Multicenter Trials  ul. "Damyan Gruev" 8  Sofia, 1303  BULGARIA  Komisiya po etika pri MBAL na Voennomeditsinska Akademia - Sofia/Ethics Commettee at MMA HAT-Sofia  MMA HAT-Sofia  Ul. Georgi Sofiyski  3  Sofia, 1606  BULGARIA |
|  |  |  |  |  |  |
| 1136 | Dr. Daniela Bichovska |  | Dr. Ivan Bichovski  Emilia F. Fileva-Veleva | DKTs "Sveta Anna" Sofia  Konsultativen Kabinet po Revmatologia  Diagnostic Consultative Center "Sveta Anna" Sofia  Ul. "Dimitar Mollov" 1  Sofia, 1709  BULGARIA | Ethics Committee for Multicenter Trials  ul. "Damyan Gruev" 8  Sofia, 1303  BULGARIA  Komisiya po etika pri DKTs"Sv. Anna"/ Ethics Committee at DCC "Sv. Anna"  DKTs "Sveta Anna"  Diagnostic Consultative Center "Sveta Anna"  Ul. Dimitar Mollov 1  Sofia, 1709  BULGARIA |
|  |  |  |  |  |  |
| 1141 | Anastas Batalov |  | Dr. Rositsa Karalilova  Dr. Aneta Nikolova  Dr. Dimitar Penev | MBAL "Kaspela" Plovdiv  Otdelenie po revmatologia  MHAT "Kaspela" Plovdiv  bul. "Dunav" 1  Plovdiv, 4002  BULGARIA | Ethics Committee for Multicenter Trials  ul. "Damyan Gruev" 8  Sofia, 1303  BULGARIA  Komisiya po etika kam MBAL "Kaspela"/ Ethics committee at MHAT "Kaspela"  MBAL 'Kaspela' Plovdiv  MHAT 'Kaspela' Plovdiv  Ul. Sofiya 64  Plovdiv, 4002  BULGARIA |
|  |  |  |  |  |  |
| 1177 | Dr. Ivan Goranov |  | Katya Kuchmova  Mariana Panayotova | MBAL - Plovdiv  Revmatologichno Otdelenie  MHAT - Plovdiv  bul. "Bulgaria" 234  Plovdiv, 4000  BULGARIA | Ethics Committee for Multicenter Trials  ul. "Damyan Gruev" 8  Sofia, 1303  BULGARIA  Komisiya po etika pri MBAL-Plovdiv/Ethics Committee at MHAT-Plovdiv  MBAL-Plovdiv  MHAT-Plovdiv  bul. ¿Bulgaria¿ 234  Plovdiv, 4000  BULGARIA |
|  |  |  |  |  |  |

## Canada

**Coordinating Investigators:**

Leeanna Bulinckx

Raymonde Gregoire

Judi Guthrie

Jolaine L'Archeveque

Dr. Alexander Yan

Karen Doyle (Previous Coordinating Investigator)

Evelyne Lejeune (Previous Coordinating Investigator)

| **Center** | **Principal Investigator** | **Co-Investigator(s)** | **Sub-Investigator(s)** | **Address(es)** | **Institutional Review Board or Ethics Committee Address(es)** |
| --- | --- | --- | --- | --- | --- |
|  |  |  |  |  |  |
| 1010 | Dr. Alfred A. Cividino |  | Dr. Raja Bobba  Dr. Pauline Boulos  Janet Miron  Louise Sloat | MAC Research Inc.  187 Hughson Street South  Hamilton, ON L8N 2B6  CANADA | IRB Services  Suite 300  372 Hollandview Trail  Aurora, ON L4G 0A5  CANADA |
|  |  |  |  |  |  |
| 1011 | Dr. J. Carter Thorne |  | Lois Derrick  Edward Ng Tung Hing  Jennifer Reist  Ms. Katalin Russell | The Arthritis Program Research Group Inc.  43 Lundy's Lane  Newmarket, ON L3Y 3R7  CANADA | IRB Services  Suite 300  372 Hollandview Trail  Aurora, ON L4G 0A5  CANADA |
|  |  |  |  |  |  |
| 1012 * | Dr. Edward C. Keystone |  | Dr. Vivian P. Bykerk  Dr. Peter Lee | Mount Sinai Hospital, The Rebecca MacDonald Centre for Arthritis and Autoimmune Disease  The Joseph and Wolf Lebovic Building, Room 2-006  60 Murray Street  Toronto, ON M5T 3L9  CANADA | Mount Sinai Hospital Research Ethics Board  600 University Avenue  Toronto, ON M5G 1X5  CANADA |
|  |  |  |  |  |  |
| 1015 | Dr. Majed Khraishi |  | Karen Doyle  Dr. Ian Landells  Dr. Ian Landells  Tina Pretty-Haines | Nexus Clinical Research  120 Stavanger Drive Suite 102  St. John's, NL A1A 5E8  CANADA | IRB Services  Suite 300  372 Hollandview Trail  Aurora, ON L4G 0A5  CANADA |
|  |  |  |  |  |  |
| 1016 | Dr. Boulos Haraoui |  | Dr. Denis Choquette  Dr. Jean-Pierre Raynauld | Institut de Rheumatologie de Montreal  1551 Ontario Est  Montreal, QC H2L 1S6  CANADA | IRB Services  Suite 300  372 Hollandview Trail  Aurora, ON L4G 0A5  CANADA |
|  |  |  |  |  |  |
| 1017 | Dr. Milton F. Baker |  | Dr. Christopher Atkins  Linda Ross | PerCuro Clinical Research Ltd.  200-1105 Pandora Avenue  Victoria, BC V8V 3P9  CANADA | IRB Services  Suite 300  372 Hollandview Trail  Aurora, ON L4G 0A5  CANADA |
|  |  |  |  |  |  |
| 1019 | Dr. Louis Bessette |  | Dr. Christine Banville  Dr. Marie-Claire Banville  Dr. Jacques Brown  Chantal Jacques  Evelyne Lejeune  Dr. Louise Morin | Groupe de recherche en rhumatologie et maladies osseuses  101-1200 Avenue de Germain-des-Pres  Quebec, QC G1V 3M7  CANADA | IRB Services  Suite 300  372 Hollandview Trail  Aurora, ON L4G 0A5  CANADA |
|  |  |  |  |  |  |
| 1020 | Dr. Henry Niall Jones |  | Dr. Shafiq Akbar  Dr. Dalton Ernest Sholter | Rheumatology Research Associates Ltd.  10839 124 Street  Edmonton, AB T5M 0H4  CANADA | College of Physicians and Surgeons of Alberta  2700 10020 100 Street  Edmonton, AB T5J 0N3  CANADA |
|  |  |  |  |  |  |

## Colombia

**Coordinating Investigators:**

<None Entered>

| **Center** | **Principal Investigator** | **Co-Investigator(s)** | **Sub-Investigator(s)** | **Address(es)** | **Institutional Review Board or Ethics Committee Address(es)** |
| --- | --- | --- | --- | --- | --- |
|  |  |  |  |  |  |
| 1057 | Dr. Edwin Antonio Jauregui MD (Previous PI)  Dr. Maria Concepcion Maldonado |  | Dr. Maria Claudia Diaz  Dr. Aura Maria Dominguez  Dr. Edwin Antonio Jauregui MD  Dr. Jhon Jairo Medina  Dr. Yenny Soraida Valero | Riesgo de Fractura S.A.  Carrera 12 # 98-38  Bogota, Cundinamarca  COLOMBIA | Comite de etica de la investigación-Riesgo de Fractura S.A  Cr. 12 No.98-38  Bogota, Cundinarca  COLOMBIA |
|  |  |  |  |  |  |
| 1130 | Dr. Javier Dario Marquez Hernandez MD |  | Dr. Luis Fernando Pinto  Dr. Carlos Jaime Velasquez Franco | Hospital Pablo Tobon Uribe  Calle 78B #69-240 Piso 9 Unidad de Investigaciones  Medellin, Antioquia 0  COLOMBIA | Comite de investigaciones y etica en investigaciones Pablo Tobon Uribe  Calle 78B No.69-240  Medellin, Antioquia 0000  COLOMBIA |
|  |  |  |  |  |  |
| 1132 | Dr. William Jose Otero Escalante MD |  | Dr. Marcial Martinez  Dr. Gerardo Ramirez MD  Hernan Roberto Vera Quinche | SERVIMED E.U  Calle 51# 34-17 Consultorio 208-208A Centro Comercial Cabecera. Etapa I  Bucaramanga, Santander  COLOMBIA | Comite de ética en Investigación de Servimed E.U  Calle 51 No. 34-17 Consultorio 208-208A Centro comercial  Cabecera etapa I  Bucaramanga, Santander 0000  COLOMBIA |
|  |  |  |  |  |  |
| 1191 | Dr. Juan Jose Jaller Raad |  | Dr. Javier Cuartas  Dr. Anubys Maiguel  Dr. Victor Andres Ulloque Lopez | Centro de Reumatologia y Ortopedia  Cra 49 C No 82-125  Barranquilla, Atlantico 0000  COLOMBIA | Comité de etica independiente centro de reumatologia y ortopedia  Cr. 49C No. 82-120  Barranquilla, Atlantico 0000  COLOMBIA |
|  |  |  |  |  |  |

## Czech Republic

**Coordinating Investigators:**

<None Entered>

| **Center** | **Principal Investigator** | **Co-Investigator(s)** | **Sub-Investigator(s)** | **Address(es)** | **Institutional Review Board or Ethics Committee Address(es)** |
| --- | --- | --- | --- | --- | --- |
|  |  |  |  |  |  |
| 1169 | Dr. Zdenek Dvorak |  | Lucie Koubova  Petra Stefanova  Dr. Martina Vaneckova | ARTHROMED, s. r. o. Revmatologicka ambulance  Rokycanova 2798  Pardubice, 530 02  CZECH REPUBLIC  CCBR Czech, a.s.  Trida miru 2800  Pardubice, 530 02  CZECH REPUBLIC | Eticka komise pri Fak. Thomayerove nemocnici a IKEM  Videnska 800  Praha 4, 140 59  CZECH REPUBLIC |
|  |  |  |  |  |  |
| 1170 | Dr. Jan Rosa |  | Dr. Petr Kasalicky  Alena Slechticka | DC Mediscan  Sustova 1930  Praha 11 - Chodov, 148 00  CZECH REPUBLIC  Nemocnice na Frantisku  Radiodiagnosticke oddeleni  Na Frantisku 847/8  Praha 1, 11000  CZECH REPUBLIC | Eticka komise pri Fak. Thomayerove nemocnici a IKEM  Videnska 800  Praha 4, 140 59  CZECH REPUBLIC |
|  |  |  |  |  |  |
| 1171 | Dr. Jana Kopackova |  | Dr. Karel Fara | ARTMEDI UPD s r.o.  Ceskoslovenske armady 164  Hostivice, 253 01  CZECH REPUBLIC  Nemocnice na Frantisku  Radiodiagnosticke oddeleni  Palackeho 720/5  Praha 1, 11000  CZECH REPUBLIC | Eticka komise pri Fak. Thomayerove nemocnici a IKEM  Videnska 800  Praha 4, 140 59  CZECH REPUBLIC |
|  |  |  |  |  |  |
| 1172 | Dr. Petr Vitek |  | Dr. Olga Januskova | Nemocnice Atlas, a.s.  Radiodiagnosticke oddeleni  tr. T. Bati 5135  Zlin, 760 01  CZECH REPUBLIC  PV-Medical s.r.o.  Revmatologicka ambulance  Padelky I/3645  Zlin, 760 01  CZECH REPUBLIC | Eticka komise pri Fak. Thomayerove nemocnici a IKEM  Videnska 800  Praha 4, 140 59  CZECH REPUBLIC |
|  |  |  |  |  |  |
| 1173 | Dr. Zdenka Mosterova |  | Dr. Rene Moster  Dr. Erik Moster | Revmacentrum MUDr. Mostera, s.r.o.  Mosnova 8  Brno - Zidenice, 615 00  CZECH REPUBLIC  X-MEDICA s.r.o.  Jugoslavska 11  Brno, 61300  CZECH REPUBLIC | Eticka komise pri Fak. Thomayerove nemocnici a IKEM  Videnska 800  Praha 4, 140 59  CZECH REPUBLIC |
|  |  |  |  |  |  |
| 1175 | Dr. Dana Tegzova |  | Dr. Sarka Forejtova  Dr. Katerina Jarosova  Prof. Jiri Vencovsky | Revmatologicky ustav  Na Slupi 4  Praha 2, 128 50  CZECH REPUBLIC  Revmatologicky ustav  Radiodiagnosticke oddeleni  Na Slupi 4  Praha 2, 128 50  CZECH REPUBLIC | Eticka komise pri Fak. Thomayerove nemocnici a IKEM  Videnska 800  Praha 4, 140 59  CZECH REPUBLIC  Revmatologicky ustav  Eticka komise  Na Slupi 4  Praha 2, 128 50  CZECH REPUBLIC |
|  |  |  |  |  |  |

## Greece

**Coordinating Investigators:**

<None Entered>

| **Center** | **Principal Investigator** | **Co-Investigator(s)** | **Sub-Investigator(s)** | **Address(es)** | **Institutional Review Board or Ethics Committee Address(es)** |
| --- | --- | --- | --- | --- | --- |
|  |  |  |  |  |  |
| 1030 | Prof. Loukas Settas |  | Dr. Paschalis Dalkidis  Julia Grammatikopoulou  Dr. Andreas Karagiannakidis  Markos Kostopoulos | A.H.E.P.A. Hospital/First Internal Medicine Clinic  1 Stilponos Kyriakidi Street  Thessaloniki, 54 636  GREECE | National Ethics Committee  284 Mesogion Avenue  Athens, 15562  GREECE |
|  |  |  |  |  |  |

## India

**Coordinating Investigators:**

<None Entered>

| **Center** | **Principal Investigator** | **Co-Investigator(s)** | **Sub-Investigator(s)** | **Address(es)** | **Institutional Review Board or Ethics Committee Address(es)** |
| --- | --- | --- | --- | --- | --- |
|  |  |  |  |  |  |
| 1124 | Dr. Vineeta Shobha |  | Mr. Shashidharan Bapulli  Mr. Azhar Basha  Deepu Chandrappa Boregowda  Ms. Malavi Golla  Ms. Jeromi Lakshmaiah  Ms. Swathi Mothkur  Mr. Saseedaran Papully  Dr. Cecil Ross  Ms. Ramya Singh  Dr. Soumya Umesh | St. John's Medical College Hospital, Department of Medicine  Sarjapur Road  Bangalore, Karnataka 560 034  INDIA | Institutional Ethical Review Board, St. John's Medical College  Sarjapur Road  Bangalore, Karnataka 560 034  INDIA |
|  |  |  |  |  |  |
| 1125 | Dr. Srikantiah Chandrashekara |  | Mr. Azam Basheer  Mr. Raghunandan Bommanna  Dr. Jalaja Ganganna  Dr. Sneha Ramachandra Kulkarni  Mr. Sasi Kumar  Mr. Sasi Kumar  Dr. Renuka Panchagnula  Mr. Amarnath Reddy Posini  Mr. Govindu Kallalli Setti  Dr. Radhika Sreeramappa  Ms. Shuchita Thawait | Chanre Rheumatology & Immunology Center & Research  #149, 15th Main NHCL,  Water Tank road,  4th Block,3rd Stage, Basaveswaranagar,  Bangalore, Karnataka 560 079  INDIA | Institutional Ethics Committee  #149, 15th Main, NHCL,  Water Tank Road, 4th Block, 3rd Stage  Basaveswaranagar  Bangalore, Karnataka 560 079  INDIA |
|  |  |  |  |  |  |
| 1126 | Dr. Shrikant Wagh |  | Dr. Ashish Goyal  Dr. Priscilla Joshi  Dr. Veena Kulkarni  Dr Raviraj Pisal  Dr. Nuzhat Shaikh  Dr. Ratnamala Swami  Dr. Beenu Varghese  Dr. Pratibha Walde | Jehangir Clinical Development Centre Pvt. Ltd.  Jehangir Hospital  32, Sassoon Road  Pune, Maharashtra 411 001  INDIA | Hirabai Cowasji Jehangir Medical Research Institute and Jehangir Development Centre Ethics Committee  Jehangir Hospital Premises  32, Sassoon Road  Pune, Maharashtra 411 001  INDIA |
|  |  |  |  |  |  |
| 1127 | Dr. Arvind K. Chopra |  | Dr. Sharon Arthur  Ms. Shama Kazi  Ms. Rubina Kazi  Dr. Vinaya Rajendrakumar Kunjeer  Dr. Vaijayanti Vardhan Lagu-Joshi  Dr. Pradeep Naik  Ms. Manjit Gurudas Saluja  Dr. Sheetal S. Salvi  Ms. Zaida Shaikh | Center for Rheumatic Diseases  No. 11, Hermes Elegance  1988, Convent Street  Camp  Pune, Maharashtra 411 001  INDIA | CRD Ethics Committee  11, Hermes Elegance  1988, Convent Street Camp  Pune, Maharashtra 411 001  INDIA |
|  |  |  |  |  |  |
| 1149 | Dr. Uppuluri Ramakrishna Rao |  | Ms. Shashikala Arava  Mr. Shaik Aziz  Mr. Sadat Baig  Dr. Firdaus Fatima  Mr. Ashok Kumar Guggilla  Dr. Datta Kumar  Dr. Maryam Younis Mohiuddin  Mr. Venkata Subrahamanya Sarma Valiveti  Mr. Gopalakrishnaiah Vempati | Sri Deepti Rheumatology Centre  6-2-45/8,  A.C.Guards,  Hyderabad, Andra Pradesh 500 004  INDIA | Ethics Committee  Sri Deepti Rheumatology  6-2-45/B,  A. C. Guards  Hyderabad, Andhra Pradesh 500 004  INDIA |
|  |  |  |  |  |  |
| 1150 | Dr. Sarath Chandra Mouli Veeravalli |  | Mr. Naveen Kumar Bairi  Ms. Kalavathi Kodi  Dr. Venkata Chalapathi Rao Mamidi  Mr. Kiran Kumar Narisepally  Dr. Ravi Kumar Neela  Ms. Rajeshwari Neela  Ms. Sunitha Rani Pagidipalli  Dr. Sreenivas Reddy Pesaru  Dr. Namrata Sridhar  Dr. Himabindu Vasamreddy | Department of Rheumatology  Krishna Institute of Medical Sciences  1-8-31/1, Minister Road  Secunderabad, Andhra Pradesh 500003  INDIA | Institutional Ethics Committee  Krishna Institute of Medical Sciences Ltd.  1-8-31/1, Minister Road  Secunderabad, Andra Pradesh 500003  INDIA |
|  |  |  |  |  |  |
| 1174 | Dr. Prabha Adhikari |  | Dr. Basavaprabhu Achappa  Ms. Beena Banz  Mr. Arindam Basu  Ms. Deepa Bhat  Dr. Keshava Bhat  Dr. Sydney D'souza  Dr. Susan D'souza  Dr. Vishal Garg  Ms. Manjula Girish  Ms Anupama Holla  Ms. Mary Jyothi  Dr. Ramachandra K. Kamath  Dr. Deepak R. Madi  Ms Suprabha Pai  Dr. John Thomas Ramapuram  Dr. Shalini Rao  Ms. Rajashree Rao  Dr. Satish B. Rao  Dr. Raghava Sharma  Dr. Pradeep Shenoy  Dr. Ashok K. Shenoy  Dr. Sonali Ullal  Dr. Tarun Varma | Manipal Acunova KMC Clinical Research Centre  5th Floor, MCODS Building  Attavar  Mangalore, Karnataka 575 001  INDIA | Manipal University Ethics Cimmittee  Madhav Nagar  Manipal, karnataka 576 104  INDIA |
|  |  |  |  |  |  |
| 1182 | Dr. Jugal Kishore Kadel |  | Mr. Shaik Aziz  Ms. Shameen Begum  Dr. Manik Dixit  Ms. Ratnakumari Gopu  Dr. Sindhu Joshi  Dr. Vikas Kale  Dr. Srinivas Kulkarni  Ms. Madhuri Mahajan  Mr. Ramya Nagabandi  Govindaiah Pulgam  Ms. Alahari Subbalakshmi  Mr. Mohammed Mateen Yazdani | Mahavir Hospital & Research Center  10-1-1, Bhagwan Mahavir Marg  A.C. Guards  Hyderabad, Andhra Pradesh 500 004  INDIA | Institutional Ethical Committee for Bio Medical Research  Bhagwan Mahavir Medical Research Center, Mahavir Hospital & Research Center,  10-1-1, Bhagwan Mahavir Marg,  A.C. Guards,  Hyderabad, Andhra Pradesh 500 004  INDIA |
|  |  |  |  |  |  |

## Japan

**Coordinating Investigators:**

<None Entered>

| **Center** | **Principal Investigator** | **Co-Investigator(s)** | **Sub-Investigator(s)** | **Address(es)** | **Institutional Review Board or Ethics Committee Address(es)** |
| --- | --- | --- | --- | --- | --- |
|  |  |  |  |  |  |
| 1108 | Dr. Yoshiya Tanaka |  | Shunsuke Fukuyo  Kentaro Hanami  Dr. Eri Hirakawa  Dr. Shintaro Hirata  Shigeru Iwata  Dr. Takayuki Katsuyama  Satoshi Kubo  Dr. Ippei Miyagawa  Kazuhisa Nakano  Masao Nawata  Kazuyoshi Saito  Dr. Kazuki Someya  Koshiro Sonomoto  Dr. Shizuyo Tsujimura  Kunihiro Yamaoka  Dr. Maiko Yoshikawa  Dr. Sonosuke Yukawa  Dr. Naoki Yunoue | University of Occupational and Environmental Health Hospital  1-1  Iseigaoka  Yahatanishi-ku  Kitakyusyu, Fukuoka  JAPAN | University of Occupational and Environmental Health Hospital IRB  1-1  Iseigaoka  Yahata-Nishi-ku  Kita-Kyushu,, Fukuoka 807-8555  JAPAN |
|  |  |  |  |  |  |
| 1109 | Dr. Hisashi Yamanaka |  | Sayumi Baba  Dr. Chikako Fukasawa  Dr. Takefumi Furuya  Daisuke Hoshi  Dr. Naomi Ichikawa  Dr. Katsunori Ikari  Dr. Takuji Iwamoto  Dr. Tokiko Kanno  Dr. Mariko Kitahama  Dr. Tsuyoshi Kobashigawa  Dr. Shigeru Kotake  Dr. Yumi Kozeki  Dr. Shigeki Momohara  Dr. Ayako Nakajima  Dr. Yuki Nanke  Dr. Eri Sato  Yohei Seto  Kumi Shidara  Kae Takagi  Dr. Atsuo Taniguchi  Akiko Tochimoto  Dr. So Tsukahara  Wako Urano | Tokyo Women's Medical University, Institute of Rheumatology  10-22  Kawada-cho  Shinjuku-ku, Tokyo 162-0054  JAPAN | Tokyo Women's Medical University Hospital IRB  8-1  Kawada-cho  Shinjyuku-ku, Tokyo 162-8666  JAPAN |
|  |  |  |  |  |  |
| 1110 | Dr. Kouichi Amano |  | Dr. Hideto Kameda  Dr. Tsuneo Kondo  Dr. Takahiko Kurasawa  Dr. Hayato Nagasawa  Eiko Nishi  Dr. Koji Nishimura  Dr. Ayumi Okuyama  Dr. Katsuya Suzuki  Dr. Hirofumi Takei  Tsutomu Takeuchi  Dr. Kensei Tsusaka | Saitama Medical Center  1981  Kamoda  Kawagoe-shi, Saitama 350-8550  JAPAN | Saitama medical center IRB  1981  Kamoda, Kawagoe-shi  Saitama, Japan 350-8550  JAPAN |
|  |  |  |  |  |  |
| 1111 | Nobuyuki Miyasaka |  | Dr. Masayoshi Harigai  Shinya Hirata  Hideyuki Iwai  Dr. Hitoshi Kohsaka  Dr. Ryuji Koike  Dr. Tetsuo Kubota  Fumitaka Mizoguchi  Toshihiro Nanki  Dr. Kazuki Takada  Dr. Akito Takamura  Dr. Michi Tanaka  Dr. Kaori Watanabe | Tokyo Medical And Dental University Hospital, Faculty of Medicine  1-5-45  Yushima  Bunkyo-ku, Tokyo 113-8519  JAPAN | Tokyo Medical And Dental University Hospital, Faculty of Medicine IRB  1-5-45  Yushima  Bunkyo-ku,, Tokyo 113-8519  JAPAN |
|  |  |  |  |  |  |
| 1113 | Dr. Shigeto Tohma |  | Hiroshi Furukawa  Hidekazu Futami  Atsushi Hashimoto  Tatsuoh Ikenaka  Kanako Iwata  Toshihiro Matsui  Hisanori Nakayama  Yuko Okazaki  Kota Shimada  Hirokazu Takaoka | National Hospital Organization Sagamihara National Hospital  18-1  Sakuradai, Minami-ku  Sagamihara, Kanagawa 252-0392  JAPAN | National Hospital Organization Central Review Board  2-5-21  Higashigaoka  Meguro, Tokyo 152-0021  JAPAN |
|  |  |  |  |  |  |
| 1116 | Dr. Shunsuke Mori |  | Junji Hamamoto  Tateki Segata  Akihisa Yamashita  Kensuke Yonemura | Kumamoto Saishunso National Hospital  2659  Suya  Koushi, Kumamoto 861-1196  JAPAN | National Hospital Organization Central Review Board  2-5-21  Higashigaoka  Meguro, Tokyo 152-0021  JAPAN |
|  |  |  |  |  |  |
| 1117 | Shuji Ohta |  | Seiji Mogi | Taga General Hospital  2-1-2  Kokubu-cho  Hitachi-shi, Ibaraki 316-0035  JAPAN | Taga General Hospital IRB  2-1-2,  Kokubu-cho,  Hitachi-shi,, Ibaraki 316-0035  JAPAN |
|  |  |  |  |  |  |
| 1119 | Yasuhiko Munakata |  | Hiroshi Fujii  Kazuyuki Honda  Naoko Misu  Hideyuki Saito  Shigeru Wakatsuki | Taihaku Sakura Hospital  1-12-26  Tomizawa  Taihaku-ku  Sendai, Miyagi 982-0032  JAPAN | NS Clinic Institutional Review Board  2-26-9  Myojincho  Hachioji  Tokyo, Japan 192-0046  JAPAN |
|  |  |  |  |  |  |
| 1120 | Yoshinari Takasaki |  | Dr. Hirofumi Amano  Dr. Shouseki Lee  Dr. Ran Matsudaira  Dr. Masakazu Matsushita  Dr. Shinji Morimoto  Dr. Michihiro Ogasawara  Dr. Hitoshi Ogasawara  Fumio Sekiya  Dr. Kurisu Tada  Dr. Naoto Tamura  Dr. Ken Yamaji | Juntendo University Hospital  3-1-3  Hongo  Bunkyo-ku, Tokyo 113-8431  JAPAN | Juntendo University Hospital IRB  Juntendo University Hospital  3-1-3  Hongo  Bunkyo-ku, Tokyo 113-8431  JAPAN |
|  |  |  |  |  |  |
| 1167 | Yojiro Kawabe |  | Koichiro Aratake  Fumiko Tanaka | National Hospital Organization Ureshino Medical Center  2436  Ooaza shimojyukuhei  Ureshino-machi  Ureshino-shi, Saga 843-0393  JAPAN | National Hospital Organization Central Review Board  2-5-21  Higashigaoka  Meguro, Tokyo 152-0021  JAPAN |
|  |  |  |  |  |  |
| 1168 | Kiyoshi Migita |  | Yasumori Izumi  Taiichiro Miyashita  Satoru Motokawa  Tadayoshi Ohno  Takafumi Torigoshi | National Hospital Organization Nagasaki Medical Center  2-1001-1  Kubara  Ohmura, Nagasaki 856-8562  JAPAN | National Hospital Organization Central Review Board  2-5-21  Higashigaoka  Meguro, Tokyo 152-0021  JAPAN |
|  |  |  |  |  |  |
| 1178 | Yukitaka Ueki |  | Nozomi Iwanaga  Hironobu Sato  Kaoru Terada  Satoshi Yamasaki | Sasebo Chuo Hospital  15  Yamato-cho  Sasebo, Nagasaki 857-1195  JAPAN | Sasebo Chuo Hospital IRB  Sasebo Chuo Hospital  15  Yamato-cho  Sasebo, Nagasaki 857-1195  JAPAN |
|  |  |  |  |  |  |
| 1185 | Mitsuhiro Iwahashi  Seizo Yamana (Previous PI) |  | Motoaki Kin  Keisuke Kobayashi  Rie Sasaki  Seizo Yamana  Jiro Yamana | Higashihiroshima Memorial Hospital  2214  Yoshiyuki, Saijo-cho  Higashihiroshima, Hiroshima 739-0002  JAPAN | Higashihiroshima Memorial Hospital IRB  2214  Yoshiyuki  Saijo-cho  Higashihiroshima, Hiroshima 739-0002  JAPAN |
|  |  |  |  |  |  |
| 1190 | Tsutomu Takeuchi |  | Keisuke Izumi  Yuko Kaneko  Noriko Kimura  Masataka Kuwana  Hidekata Yasuoka | Keio University Hospital  35  Shinano-machi  Shinjuku-ku, Tokyo, Japan 160-8582  JAPAN | Keio University Hospital IRB  35  Shinano-machi  Shinjuku-ku, Tokyo 160-8582  JAPAN |
|  |  |  |  |  |  |
| 1192 | Dr Toshiaki Miyamoto |  | Rei Ito  Dr. Yuichiro Taguchi | Seirei Hamamatsu General Hospital  2-12-12  Sumiyoshi  Hamamatsu, Shizuoka 430-8558  JAPAN  Seirei Hamamatsu General Hospital  2-12-12  Naka-ku, Sumiyoshi  Hamamatsu, Shizuoka 430-8558  JAPAN | Hamamatsu Clinical Research Network Institutional Review Board  2-12-12  Naka-ku, Sumiyoshi  Hamamatsu, Shizuoka 430-8558  JAPAN |
|  |  |  |  |  |  |

## Korea, Republic Of

**Coordinating Investigators:**

<None Entered>

| **Center** | **Principal Investigator** | **Co-Investigator(s)** | **Sub-Investigator(s)** | **Address(es)** | **Institutional Review Board or Ethics Committee Address(es)** |
| --- | --- | --- | --- | --- | --- |
|  |  |  |  |  |  |
| 1038 | Soo-Kon Lee |  | Sang-Youn Jung  Yoon Kang  Kwang Hoon Lee  Hyung Ah Park | Yonsei University College of Medicine, Severance Hospital, Rheumatology, Internal Medicine  134 Shinchon-dong, Seodaemun-gu  Seoul, 120-752  KOREA, REPUBLIC OF | IRB of Severance Hospital  134 Shinchon-dong, Seodaemun-gu  Seoul, 120-752  KOREA, REPUBLIC OF |
|  |  |  |  |  |  |
| 1039 | Yeong-Wook Song |  | Sunghae Chang  In Ah Choi  Churl Hyun Im  Chul Kim  Hye Won Kim  Jin Hyun Kim  Joonwan Kim  Eun Young Lee  Eun Bong Lee  Kichul Shin  Ran Song  Young Im Yoon | Seoul National University Hospital, Rheumatology, Internal Medicine  28 Yongon-dong, Chongno-gu  Seoul, 110-744  KOREA, REPUBLIC OF | IRB of Seoul National University Hospital  Institutional Review Board  28 Yongon-dong, Chongno-gu  Seoul, 110-744  KOREA, REPUBLIC OF |
|  |  |  |  |  |  |
| 1040 | Dr. Sang-Cheol Bae |  | So Young Bang  Soo Kyung Cho  Chan-Bum Choi  Jae-Bum Jun  Kyong Hee Jung  Kyong Hee Jung  Young Sam Kim  Eun Mi Kim  Jahee Kim  Eun Mi Kim  Tae-Hwan Kim  Kyeong A. Lee  So Yeon Park  So Yeon Park  Yoon-Kyoung Sung  Dae-Hyun Yoo | Hanyang University Hospital, Department of Rheumatology  17 Haengdang-dong, Seongdong-gu  Seoul, 133-792  KOREA, REPUBLIC OF | IRB of Hanyang University Hospital  Institutional Review Board  17, Haengdang-Dong, Seongdong-Gu  Seoul, 133-792  KOREA, REPUBLIC OF |
|  |  |  |  |  |  |
| 1041 | Won Park |  | Jung Ran Choi  Kowoon Joo  Seong Ryul Kwon  Mi Young Lee  Mie Jin Lim  Chang Gi Moon  Ji Yeol Yoon | Inha University Hospital, Medicine/Rheumatology  7-206, 3-Ga, Sinheung-Dong, Jung-Gu  Incheon, 400-711  KOREA, REPUBLIC OF | IRB of Inha University Hospital  7-206, 3-Ga, Sinheung-Dong, Jung-Gu  Incheon, 400-711  KOREA, REPUBLIC OF |
|  |  |  |  |  |  |
| 1042 | Sung-Hwan Park |  | Ji Hyeon Ju  Ji Hyeon Ju  Dr. Ho-Youn Kim  Dr. Ho-Youn Kim  Seung-Ki Kwok  Kyung-Su Park  Ho Sung Yoon | The Catholic University of Korea, Seoul St. Mary's Hospital/ Rheumatology, Internal Medicine  505, Banpo-dong, Seocho-gu  Seoul, 137-701  KOREA, REPUBLIC OF | The Catholic University of Korea, Seoul St. Mary's Hospital IRB  505 Banpo-dong, Seocho-gu  Seoul, 137-701  KOREA, REPUBLIC OF |
|  |  |  |  |  |  |
| 1047 | Bin Yoo |  | Yong Gil Kim  Dr. Bon San Koo  Seung Geun Lee  Chang-Keun Lee  Min Wook So | Asan Medical Center, Rheumatology, Internal Medicine  388-1, Pungnap-2dong, Songpa-gu  Seoul, 138-736  KOREA, REPUBLIC OF | IRB of Asan Medical Center  86 Asanbyeongwon-gil, Songpa-Gu  Seoul, 138-736  KOREA, REPUBLIC OF |
|  |  |  |  |  |  |
| 1048 | Dr. Eun-Mi Koh (Previous PI)  Hoon-Suk Cha |  | Joong kyong Ahn  Jiwon Hwang  Dr. Jaejoon Lee  Ji Min Oh | Samsung Medical Center, Division of Rheumatology, Department of Medicine  50 Ilwon-dong, Gangnam-Gu  Seoul, 135-710  KOREA, REPUBLIC OF | IRB of Samsung Medical Center  Institutional Review Board  50 Ilwon-Dong, Gangnam-Gu  Seoul, 135-710  KOREA, REPUBLIC OF |
|  |  |  |  |  |  |
| 1049 | Seung Cheol Shim |  | Mi Kyoung Lim  Dong Hyuk Sheen | Eulji University Hospital, Internal Medicine, Rheumatology  1306 Dunsandong, Seogu  Daejeon, 302-799  KOREA, REPUBLIC OF | IRB of Eulji University Hospital  1306 Dunsandong, Seogu  Daejeon, 302-799  KOREA, REPUBLIC OF |
|  |  |  |  |  |  |

## Mexico

**Coordinating Investigators:**

<None Entered>

| **Center** | **Principal Investigator** | **Co-Investigator(s)** | **Sub-Investigator(s)** | **Address(es)** | **Institutional Review Board or Ethics Committee Address(es)** |
| --- | --- | --- | --- | --- | --- |
|  |  |  |  |  |  |
| 1063 | Dr. Virginia Pascual-Ramos |  | Lucia Comellas - Kirkerup  Hilda Esther Fragoso-Loyo  Dr. Marina Rull-Gabayet  Dr. Pablo Villasenor-Ovies | Instituto Nacional de Ciencias Medicas y Nutricion Salvador Zubiran  Departamento de Inmunologia y Reumatologia  Vasco de Quiroga 15  Colonia Seccion XVI Delegacion Tlalpan  Mexico, DF 14000  MEXICO | Comité Institucional de Investigación Biomédica en Humanos  Instituto Nacional de Ciencias Medicas y Nutricion Dr Salvador Zubiran  Vasco de Quiroga No 15  Del. Tlalpan  Mexico, DF 14000  MEXICO |
|  |  |  |  |  |  |
| 1064 | Dr. Mario H. Cardiel-Rios |  | Dr. Hilda Leticia Avila-Martinez  Evelyn Cecilia Cervantes-Perez  Rosa M. Larios-Garcia  Javier Mota-Gonzalez  Raquel Sanchez-Hernandez | Star Medica. Centro de Investigacion Clinica de Morelia SC  Virrey de Mendoza 1998-502/416  Felix Ireta  Morelia, Michoacan 58070  MEXICO | Star Medica  Comite de la Calidad de la Atencion Medica, Credenciales, Ensenanza,  Investigacion, Capacitacion y Etica, Evaluacion del Expediente Clinico  Virrey de Mendoza 2000  Felix Ireta  Morelia, Michoacan 58070  MEXICO |
|  |  |  |  |  |  |

## Poland

**Coordinating Investigators:**

<None Entered>

| **Center** | **Principal Investigator** | **Co-Investigator(s)** | **Sub-Investigator(s)** | **Address(es)** | **Institutional Review Board or Ethics Committee Address(es)** |
| --- | --- | --- | --- | --- | --- |
|  |  |  |  |  |  |
| 1142 | Dr. Ines Pokrzywnicka-Gajek |  | Dr. Ewa Czernecka  Dr. Dorota Knychas  Dr. Andrzej Sawicki  Dr. Malgorzata Szymanska  Dr. Alina Walczak | Lecznica Specjalistow, Centrum Medyczne "Osteomed" NZOZ  Al. Krakowska 110/114  Warszawa, 02-256  POLAND | Komisja Bioetyczna Okregowej Izby Lekarskiej W Warszawie  ul. Pulawska 18  Warszawa, 02-512  POLAND |
|  |  |  |  |  |  |
| 1162 | Dr. Zofia Ruzga |  | Dr. Ewa Jazwinska-Tarnawska  Ewa Krecipro-Nizinska  Dr. Anna Sidorowicz-Bialynicka  Elzbieta Sonik | "SYNEXUS SCM" Sp. z o.o.  Swobodna 8a  Wroclaw, 50-088  POLAND | Komisja Bioetyczna Okregowej Izby Lekarskiej W Warszawie  ul. Pulawska 18  Warszawa, 02-512  POLAND |
|  |  |  |  |  |  |

## Taiwan

**Coordinating Investigators:**

<None Entered>

| **Center** | **Principal Investigator** | **Co-Investigator(s)** | **Sub-Investigator(s)** | **Address(es)** | **Institutional Review Board or Ethics Committee Address(es)** |
| --- | --- | --- | --- | --- | --- |
|  |  |  |  |  |  |
| 1078 | Dr. Wen-Chan Tsai |  | Jia-Hua Ho  Tsan-Teng Ou  Chen-Ching Wu  Jeng-Hsien Yen | Chung-Ho Memorial Hospital, Kaohsiung Medical University  No. 100 Tzyou 1st Road  Kaohsiung, 807  TAIWAN | Kaohsiung Medical University Chung-Ho Memorial Hospital, Institutional Review Board  No. 100, Tzyou 1st Road  Kaohsiung, Taiwan 807  TAIWAN |
|  |  |  |  |  |  |
| 1079 | Dr. Ping-Ning Hsu |  | Yun-Chen Toh | National Taiwan University Hospital  No. 7 Chung-Shan South Road  Taipei, 100  TAIWAN | Research Ethics Committee of National Taiwan University Hospital  No.7 Chung-Shan South Road  Taipei, 100  TAIWAN |
|  |  |  |  |  |  |
| 1080 | Dr. Hsiao-Yi Lin |  | Yi-Chun Lin | Taipei Veterans General Hospital  Division of allergy, Immunology & Rheumatology, Department of Medicine  No. 201, Section 2, Shih-Pai Road  Taipei, 112  TAIWAN | Taipei Veterans General Hospital, The Institutional Review Board  No. 201  Sec. 2, Shih-Pai Road  Taipei, 112  TAIWAN |
|  |  |  |  |  |  |
| 1102 | Lieh-bang Liou |  | Huei-Huang Ho  Wen-Pin Tsai | Chang Gung Medical Foundation-Linkou Branch  5  Fu-Shin Street  Kweishan, Taoyuan County, 333  TAIWAN | Chang Gung Medical Foundation, Institutional Review Board  No. 199 Tung Hwa North Road  Taipei, 10507  TAIWAN |
|  |  |  |  |  |  |
| 1103 | Dr. Ming-Fei Liu |  | Meng-Yu Weng | National Cheng Kung University Hospital  No. 138, Sheng-Li Road  Tainan, 704  TAIWAN | Human Experiment and Ethics Committee National Cheng Kung University Hospital  138 Sheng Li Road  Tainan, 704  TAIWAN |
|  |  |  |  |  |  |
| 1122 | Dr. Joung-liang Lan |  | Yi-Hsing Chen  Dr. Der-Yuan Chen  Hsin-Hua Chen  Hsiu-Cheng Chou  Dr. Tsu-yi Hsieh  Dr. Chia-Wei Hsieh  Wen-Nan Huang | Taichung Veterans General Hospital  No. 160, Section. 3, Taichung Port Road, Situn District  Taichung, 407  TAIWAN | The Institutional Review Board of Taichung Veterans General Hospital  No. 160, Section. 3, Taichung Port Road., Situn District  Taichung City, 407  TAIWAN |
|  |  |  |  |  |  |

## Ukraine

**Coordinating Investigators:**

<None Entered>

| **Center** | **Principal Investigator** | **Co-Investigator(s)** | **Sub-Investigator(s)** | **Address(es)** | **Institutional Review Board or Ethics Committee Address(es)** |
| --- | --- | --- | --- | --- | --- |
|  |  |  |  |  |  |
| 1153 | Vira Iosypivna Tseluyko |  | Dr. Ol'ha Victorivna Radchenko  Dr. Viktoriya Victorivna Yarosh | City Clinical Hospital # 8, Dept of Cardiology and Functional Diagnostics  266g, Saltivske Shosse  Kharkiv, 61178  UKRAINE | Central Ethics Committee Ministry of Health of Ukraine  5, Narodnogo Opolchennya Str.  Kyiv, 03680  UKRAINE  Committee for Ethics Issues of City Clinical Hospital #8  266g, Saltivske Shosse  Kharkiv, 61178  UKRAINE |
|  |  |  |  |  |  |
| 1154 | Prof. Mykola A. Stanislavchuk |  | Dr. Nabil Sh. Ali  Dr. Olena O. Savytska  Dr. Natalia V. Shcolina | Vinnitsa Regional Clinical Hospital n.a. Pirogov, Dept of Internal Medicine #1 of Vinnitsa NMU  46 Pirogova Street  Vinnitsa, 21018  UKRAINE | Bioethics Committee of Vinnitsa Regional Clinical Hospital n.a. Pirogov  46 Pirogova Street  Vinnitsa, 21018  UKRAINE  Central Ethics Committee Ministry of Health of Ukraine  5, Narodnogo Opolchennya Str.  Kyiv, 03680  UKRAINE |
|  |  |  |  |  |  |
| 1155 | Dr. Halyna M. Hrytsenko |  | Nataliya O. Smoley  Oksana Z. Stefyuk  Roksolana Tsurkan | Municipal City Clinical Hospital #4, Department of Rheumatology  3 Sventsitskogo Street  Lviv, 79011  UKRAINE | Central Ethics Committee Ministry of Health of Ukraine  5, Narodnogo Opolchennya Str.  Kyiv, 03680  UKRAINE  Committee for Ethics Issues of Municipal City Clinical Hospital #4  3 Sventsitskogo Str  Lviv, 79011  UKRAINE |
|  |  |  |  |  |  |
| 1156 | Prof. Vladyslav V. Povoroznyuk |  | Dr. Nataliia V. Grygorieva  Dr. Tetyana A. Karasevska  Dr. Tetyana V. Orlyk | Institute of Gerontology, Department of Clinical Physiology and Pathology of Musculoskeletal System  67 Vyshgorodska Street  Kyiv, 04114  UKRAINE | Central Ethics Committee Ministry of Health of Ukraine  5, Narodnogo Opolchennya Str.  Kyiv, 03680  UKRAINE  Committee for Ethics Issues of Institute of Gerontology  67, Vyshgorodska Street  Kyiv, 04114  UKRAINE |
|  |  |  |  |  |  |
| 1157 | Andriy Petrov |  | Dr. Ganna A. Alekseeva  Volodymyr Biloglazov  Dr. Galyna M. Koshukova | Republican Clinical Hospital  Dept of Internal Medicine #2 of SI "Crimean State Medical University n.a. S.I. Georgiyevskyj"  69, Kyivska Street  Simferopol, Crimea 95017  UKRAINE | Central Ethics Committee Ministry of Health of Ukraine  5, Narodnogo Opolchennya Str.  Kyiv, 03680  UKRAINE  Committee for Ethics Issues of Republican Clinical Hospital  69, Kyivska Street  Simferopol, Crimea 95017  UKRAINE |
|  |  |  |  |  |  |

## United States

**Coordinating Investigators:**

<None Entered>

| **Center** | **Principal Investigator** | **Co-Investigator(s)** | **Sub-Investigator(s)** | **Address(es)** | **Institutional Review Board or Ethics Committee Address(es)** |
| --- | --- | --- | --- | --- | --- |
|  |  |  |  |  |  |
| 1004 | Dr. Joel Charles Silverfield |  | Dr. Michael Claude Burnette  Dr. Harris Hugh McIlwain  Dr. Kimberly McIlwain Smith | Tampa Medical Group, PA  Suite 303  4700 North Habana Avenue  Tampa, FL 33614  UNITED STATES | Quorum Institutional Review Board  Suite 1000  1601 Fifth Avenue  Seattle, WA 98101  UNITED STATES |
|  |  |  |  |  |  |
| 1005 | Dr. Roy Mitchell Fleischmann |  | Jean A. Clark  Dr. Stanley Bruce Cohen  Dr. Thomas David Geppert  Dr. Imran Iqbal  Dr. Robert Neil Jenkins  Dr. Talat Jehan Kheshgi  Dr. Zoran Kurepa  Dr. Sharad Lakhanpal  Andrea S. Martin  Dr. Catalina Orozco  Dr. Richard L. Stern  Dayna S. Swan-Flanders  Dr. Jack Bernstein Vine  Andrea S. Wheeler | Metroplex Clinical Research Center  Suite 810  8144 Walnut Hill Lane  Dallas, TX 75231  UNITED STATES | Quorum Institutional Review Board  Suite 1000  1601 Fifth Avenue  Seattle, WA 98101  UNITED STATES |
|  |  |  |  |  |  |
| 1006 | Dr. Raymond Edward Jackson |  | Kara M. Bardram  Dr. Inocencia A. Cuesta | Premier Imaging Center  (Imaging Only)  Suite 010  31500 Telegraph  Bingham Farms, MI 48025  UNITED STATES  QUEST Research Institute  Suite 230  31000 Telegraph  Bingham Farms, MI 48025  UNITED STATES | Quorum Institutional Review Board  Suite 1000  1601 Fifth Avenue  Seattle, WA 98101  UNITED STATES |
|  |  |  |  |  |  |
| 1007 | Dr. Jeffrey Louis Kaine |  | Dr. Yoel Drucker  Dr. Daniel Small  Dr. Ronald I. Weitzner  Dr. Richard A. Yonker | Sarasota Arthritis Research Center  Suite 101  1945 Versailles Street  Sarasota, FL 34239  UNITED STATES | Quorum Institutional Review Board  Suite 1000  1601 Fifth Avenue  Seattle, WA 98101  UNITED STATES |
|  |  |  |  |  |  |
| 1021 | Dr. Jody Kay Hargrove |  | Dr. Hammad Bajwa  Dr. Vernon W. Berglund  Dr. Angela M. Dahle  Dr. Walter H. Dorman  Dr. Paul Harvey Waytz  Dr. David Charles Zoschke | Arthritis and Rheumatology Consultants, P.A.  Suite 215  7250 France Avenue South  Edina, MN 55435  UNITED STATES | Quorum Institutional Review Board  Suite 1000  1601 Fifth Avenue  Seattle, WA 98101  UNITED STATES |
|  |  |  |  |  |  |
| 1024 | Dr. Robert Michael Griffin Jr. |  | Dr. Michael Allen Borofsky  Brent William Calhoon  Jane Crosby  Dr. Saurin Mrugank Mehta  Dr. Peter Daniel Nicholas Jr.  Dr. Nancy Jane Walker  Dr. Jerome Stephen Weisberg | Clinical Research Center of Reading, LLC  2760 Century Boulevard  Wyomissing, PA 19610  UNITED STATES | Quorum Institutional Review Board  Suite 1000  1601 Fifth Avenue  Seattle, WA 98101  UNITED STATES |
|  |  |  |  |  |  |
| 1025 * | Dr. John Joseph Cush |  | Leilani D. Law | Baylor Research Institute  Arthritis Care and Research Center  Suite 550  9900 North Central Expressway  Dallas, TX 75231  UNITED STATES | Baylor Research Institute Institutional Review Board  White/Blue  Suite 501  3310 Live Oak  Dallas, TX 75204  UNITED STATES |
|  |  |  |  |  |  |
| 1046 | Dr. Patrick Thomas Schuette |  | Dr. Erin L. Arnold  Dr. William Joseph Arnold  Dr. Alfonso E. Bello  Dr. Gerald Marc Eisenberg  Dr. Ami K. Kothari  Dr. Mary L. Moran  Dr. Amanda K. Myers | Illinois Bone and Joint Institute, LLC  9000 Waukegan Road  Morton Grove, IL 60053  UNITED STATES | Quorum Institutional Review Board  Suite 1000  1601 Fifth Avenue  Seattle, WA 98101  UNITED STATES |
|  |  |  |  |  |  |
| 1050 | Dr. Alan Lawrence Brodsky |  | Dr. Pooja Banerjee  Stacey L. Cupit | Arthritis Care and Diagnostic Center  Suite 340  8440 Walnut Hill Lane  Dallas, TX 75231  UNITED STATES | Quorum Institutional Review Board  Suite 1000  1601 Fifth Avenue  Seattle, WA 98101  UNITED STATES |
|  |  |  |  |  |  |
| 1051 | Dr. Paul L. Katzenstein |  | Sherry L. Booz  Dr. Mark Stephens Box  Janet E. Harman | Kansas City Internal Medicine  Suite 350  20 Northeast St. Luke's Boulevard  Lee's Summit, MO 64086  UNITED STATES | Quorum Institutional Review Board  Suite 1000  1601 Fifth Avenue  Seattle, WA 98101  UNITED STATES |
|  |  |  |  |  |  |
| 1052 * | Dr. Steven D. Mathews |  | Dr. Darlene M. Bartilucci  Dr. Alicia D. Campbell  Dr. Ramon B. Castello  Dr. Susan Neims Greco  Dr. Keith R. Holden  Dr. Jeffry Alan Jacqmein  Dr. Michael Jay Koren  Dr. Alpa Mahendra Patel  Dawn M. Robison  Dr. Neil Sager  Dr. Carolyn M. Tran  Dr. Francis P. Valenzuela | Jacksonville Center for Clinical Research  Suite 1  4085 University Boulevard South  Jacksonville, FL 32216  UNITED STATES | Quorum Institutional Review Board  Suite 1000  1601 Fifth Avenue  Seattle, WA 98101  UNITED STATES |
|  |  |  |  |  |  |
| 1053 | Dr. Alvin Francis Wells |  | Mary J. Haning  Nicole Jodat  Kristen L. Ribar  Nicole T. Rivecca | Medical Diagnostic Imaging  X-Rays Only  3111 West Rawson Avenue  Franklin, WI 53132  UNITED STATES  Rheumatology and Immunotherapy Center  Suite 101  200 East Ryan Road  Oak Creek, WI 53154  UNITED STATES | Quorum Institutional Review Board  Suite 1000  1601 Fifth Avenue  Seattle, WA 98101  UNITED STATES |
|  |  |  |  |  |  |
| 1054 | Dr. Karen Sue Kolba |  | Susan Greene | Pacific Arthritis Center Medical Group  Suite A  607 East Plaza Drive  Santa Maria, CA 93454  UNITED STATES | Quorum Institutional Review Board  Suite 1000  1601 Fifth Avenue  Seattle, WA 98101  UNITED STATES |
|  |  |  |  |  |  |
| 1056 | Dr. Talha Shamim (Previous PI)  Suresh Kumar Reddy Pasya |  | Dr. Krishan Ariyarathna  Melanie Budine  Kylie M. Hutsell | Heartland Clinical Research, Inc.  2201 North 90th Street  Suite 125-126  Omaha, NE 68134  UNITED STATES | Quorum Institutional Review Board  Suite 1000  1601 Fifth Avenue  Seattle, WA 98101  UNITED STATES |
|  |  |  |  |  |  |
| 1059 * | Dr. Erdal Diri |  | Jerane A. Forsberg | Trinity Health Center - Medical Arts  400 Burdick Expressway East  Minot, ND 58701  UNITED STATES | Trinity IRB  Institutional Review Board  One Burdick Expressway West  Minot, ND 58701  UNITED STATES |
|  |  |  |  |  |  |
| 1060 | Dr. Michael Eugene Sayers |  | Dr. Michael Roger Baker  Dr. Martha Leigh D'Ambrosio  Dr. Melissa T. Hocate  Dr. Megan C. MacNeil  Bonnie L. Miranda  Pamela G. Saufley  Deborah Ann Spoerl | Arthritis Associates & Osteoporosis Center of Colorado Springs  Suite 200  215 Parkside Drive  Colorado Springs, CO 80910  UNITED STATES | Quorum Institutional Review Board  Suite 1000  1601 Fifth Avenue  Seattle, WA 98101  UNITED STATES |
|  |  |  |  |  |  |
| 1061 | Dr. Bridget Tyrell Walsh |  | Dr. Laurie Ann Bergstrom  Michelle K. Cornett  Dr. Michael Joseph Maricic  Dr. Sabina R. Mian  Dr. Deborah Jane Power | Catalina Pointe Clinical Research, Inc.  Suite 100  7520 North Oracle Road  Tucson, AZ 85704  UNITED STATES | Quorum Institutional Review Board  Suite 1000  1601 Fifth Avenue  Seattle, WA 98101  UNITED STATES |
|  |  |  |  |  |  |
| 1062 | Dr. Charles Allen Birbara |  | Joan Greaney  Dr. Sheela Kumar  Dr. Nassif Maalouli  Dr. Basheer Rahmoun | Clinical Pharmacology Study Group  26 Queen Street  Worcester, MA 01610  UNITED STATES | Quorum Institutional Review Board  Suite 1000  1601 Fifth Avenue  Seattle, WA 98101  UNITED STATES |
|  |  |  |  |  |  |
| 1076 | Dr. Selden Longley III |  | Dr. Meghavi Sheth Kosboth  Dr. Thomas Mark Lloyd Sr. | Southeastern Arthritis Center  Suite 8  4343 West Newberry Road  Gainesville, FL 32607  UNITED STATES  Southeastern Imaging & Diagnostics  Suite 5  4343 West Newberry Road  Gainesville, FL 32607  UNITED STATES  Southeastern Integrated Medical, PL d/b/a Florida Medical Research Institute  Suite 17  4343 West Newberry Road  Gainesville, FL 32607  UNITED STATES | Quorum Institutional Review Board  Suite 1000  1601 Fifth Avenue  Seattle, WA 98101  UNITED STATES |
|  |  |  |  |  |  |
| 1077 | Dr. Herbert Stuart Block Baraf |  | Dr. Ashley D. Beall  Dr. Paul J. DeMarco  Dr. Emma G. DiIorio  Dr. Alan Ken Matsumoto  Dr. Robert Lawrence Rosenberg  Dr. Evan Lloyd Siegel | The Center for Rheumatology and Bone Research  Suite 306  2730 University Boulevard West  Wheaton, MD 20902  UNITED STATES | Quorum Institutional Review Board  Suite 1000  1601 Fifth Avenue  Seattle, WA 98101  UNITED STATES |
|  |  |  |  |  |  |
| 1081 | Dr. Haydon Anthony Moorman  Dr. James D. Taborn (Previous PI) |  | Dr. Douglas G. Campbell  Dr. Andrew Daugavietis  Deborah Hotchkiss  Kimberly A. Kendall  Loria L. Ramos  Dr. Stacey E. Watson | Borgess Research Institute  Suite 003 and Suite 004  1717 Shaffer Street  Kalamazoo, MI 49048  UNITED STATES | Quorum Institutional Review Board  Suite 1000  1601 Fifth Avenue  Seattle, WA 98101  UNITED STATES |
|  |  |  |  |  |  |
| 1082 | Dr. Joel Marc Kremer |  | Christine J. Barr  Dr. Ludovico Frank Cavaliere  Justine S. Feder-Lailer  Dr. Neal Steven Greenstein  Dr. Dorota L. Hausner-Sypek  Jessica L. Johnson  Mari V. Kaymakcian  Justine V. Kehn  Kathleen A. Kessler  Iris B. Klein  Jessica A. Messemer  Dr. Victoria M. Michaels  Teresa M. Michaels  Rhonda L. Murphy  Dr. Norman Reid Romanoff  Dr. Harbrinder S. Sandhu  Dr. Lee Schulman Shapiro  Nicole L. Shultes  Dr. Aixa Toledo-Garcia | The Center for Rheumatology, LLP  Suite 101  1367 Washington Avenue  Albany, NY 12206  UNITED STATES | Quorum Institutional Review Board  Suite 1000  1601 Fifth Avenue  Seattle, WA 98101  UNITED STATES |
|  |  |  |  |  |  |
| 1083 | Dr. Geneva Louise Hill |  | Dr. Josette J. Johnson  Dr. Jeffrey Geldert Lawson | Piedmont Arthritis Clinic, PA  Suite 400  3 St. Francis Drive  Greenville, SC 29601  UNITED STATES | Quorum Institutional Review Board  Suite 1000  1601 Fifth Avenue  Seattle, WA 98101  UNITED STATES |
|  |  |  |  |  |  |
| 1084 | Dr. Chokkalingam Siva |  | Dr. Shantanu Bishwal  Dr. Fernando X. Castro  Deanna K. Davenport  Dr. Kenneth G. Lawlor  Dr. Celso R. Velazquez | University of Missouri-Columbia  M746 Health Sciences Center  1 Hospital Drive  Columbia, MO 65212  UNITED STATES  University Physicians  Woodrail Clinic  Building 2, Suite 150  1000 West Nifong  Columbia, MO 65203  UNITED STATES | Health Sciences Institutional Review Board  University of Missouri - Columbia  190 Galena Hall  Columbia, MO 65212  UNITED STATES |
|  |  |  |  |  |  |
| 1085 | Dr. William Rodney Palmer |  | Dr. Michael Gray Feely  Dr. Magdalena Maria Fiksinski  Dr. Kristin Sue Lake  Dr. Marcus Hilton Snow | Westroads Medical Group  10170 Nicholas Street  Omaha, NE 68114  UNITED STATES | Quorum Institutional Review Board  Suite 1000  1601 Fifth Avenue  Seattle, WA 98101  UNITED STATES |
|  |  |  |  |  |  |
| 1086 | Dr. Ara Hagop Dikranian |  | Dr. Puja Chitkara  Dr. Michael Ira Keller  Timothy F. Lazarek  Jennifer Marconato  Dr. Smitha Chiniga Reddy | San Diego Arthritis Medical Clinic  Suite 300  3633 Camino Del Rio South  San Diego, CA 92108  UNITED STATES | Quorum Institutional Review Board  Suite 1000  1601 Fifth Avenue  Seattle, WA 98101  UNITED STATES |
|  |  |  |  |  |  |
| 1087 | Dr. Sanford Mayer Wolfe |  | Dr. Chacko Alappatt  Rebecca J. Hanshew | STAT Research, Inc.  Suite 230  One Elizabeth Place, West Medical Plaza  Dayton, OH 45417  UNITED STATES | Quorum Institutional Review Board  Suite 1000  1601 Fifth Avenue  Seattle, WA 98101  UNITED STATES |
|  |  |  |  |  |  |
| 1088 | Dr. Kevin James Kempf |  | Dr. Everett H. Allen  Dr. Thomas A. Rennie Jr. | Radiant Research San Antonio Northeast  Suite 207  8527 Village Drive  San Antonio, TX 78217  UNITED STATES | Quorum Institutional Review Board  Suite 1000  1601 Fifth Avenue  Seattle, WA 98101  UNITED STATES |
|  |  |  |  |  |  |
| 1089 | Dr. Ami Charise Milton |  | Lori Ann Dana  Tricia A. Eveleigh  Dr. Ray Thomas Forbes  Deborah L. Schu  Jennifer A. Sohl | Internist Associates of Central New York  Suite 200  739 Irving Avenue  Syracuse, NY 13210  UNITED STATES | Quorum Institutional Review Board  Suite 1000  1601 Fifth Avenue  Seattle, WA 98101  UNITED STATES |
|  |  |  |  |  |  |
| 1090 | Dr. Dale George Halter |  | Bessie B. Ahrendt  Dr. Padma R. Chimata  Dr. Harvey I. Hyman  Dr. Frank Leo Lanza  Dr. Sean X. Tao  Dr. Frank R. Wellborne  Dr. Francis M. Williams | Houston Institute for Clinical Research  Suite 720  7777 Southwest Freeway  Houston, TX 77074  UNITED STATES | Quorum Institutional Review Board  Suite 1000  1601 Fifth Avenue  Seattle, WA 98101  UNITED STATES |
|  |  |  |  |  |  |
| 1092 | Dr. Shelly Pearl Kafka |  | Laura J. Stavrakis  Dr. Dominick R. Woofter | Mountain State Clinical Research  Suite 303A  300 Davisson Run Road  Clarksburg, WV 26301  UNITED STATES | Quorum Institutional Review Board  Suite 1000  1601 Fifth Avenue  Seattle, WA 98101  UNITED STATES |
|  |  |  |  |  |  |
| 1093 * | Dr. Larry W. Moreland |  | Dr. Marc C. Levesque  Dr. Douglas W. Lienesch | University of Pittsburgh  Division of Rheumatology & Clinical Immunology  Arthritis Clinical Intervention Program  3347 Forbes Avenue, 3rd Floor - Suite 303  Pittsburgh, PA 15213  UNITED STATES  University of Pittsburgh Medical Center  200 Lothrop Street  Pittsburgh, PA 15213  UNITED STATES | Quorum Institutional Review Board  Suite 1000  1601 Fifth Avenue  Seattle, WA 98101  UNITED STATES |
|  |  |  |  |  |  |
| 1094 * | Dr. Joan Marie Bathon |  | Dr. Clifton O. Bingham III  Dr. Jon T. Giles  Dr. Uzma Haque | Johns Hopkins University, JHAAC  Division of Rheumatology  Suite 1B.1  5501 Hopkins Bayview Circle  Baltimore, MD 21224-6821  UNITED STATES | The Johns Hopkins Medicine Institutional Review Board  Reed Hall, Suite B130  1620 McElderry Street  Baltimore, MD 21205-1911  UNITED STATES |
|  |  |  |  |  |  |
| 1098 | Dr. Mark William Niemer |  | George J. Casey  Julie C. Ivanov | Medical Associates Clinic, PC  1500 Associates Drive  Dubuque, IA 52002  UNITED STATES | Quorum Institutional Review Board  Suite 1000  1601 Fifth Avenue  Seattle, WA 98101  UNITED STATES |
|  |  |  |  |  |  |
| 1099 | Dr. Paul Andrew Dura |  | Kristin J. Contro  Aspen L. D'Angelo  Dr. Thomas Joseph Oven | Regional Rheumatology Associates  Suite 302  161 Riverside Drive  Binghamton, NY 13905  UNITED STATES | Quorum Institutional Review Board  Suite 1000  1601 Fifth Avenue  Seattle, WA 98101  UNITED STATES |
|  |  |  |  |  |  |
| 1101 | Dr. Richard James Misischia |  | Dr. Robert J. Capps  Dr. Marcin T. Gornisiewicz  Nickie L. Hurst  Cathy C. Rhodes  Dr. Jay Henderson Warrick  Dr. Donna M. Winn  Dr. John Frederick Wolfe | Rheumatology Consultants, PLLC  Suite 200  4707 Papermill Drive  Colony Park  Knoxville, TN 37909-1900  UNITED STATES | Quorum Institutional Review Board  Suite 1000  1601 Fifth Avenue  Seattle, WA 98101  UNITED STATES |
|  |  |  |  |  |  |
| 1133 | Dr. David Hilton Sikes |  | Dr. Mark Sol Eisner  Dr. Natalie A. Faith  Dr. Nathan A. Meyer  Michelle L. Meyer  Marokhaya Samb | Florida Medical Clinic, P.A.  Clinical Research Division  38135 Market Square  Zephyrhills, FL 33542  UNITED STATES | Quorum Institutional Review Board  Suite 1000  1601 Fifth Avenue  Seattle, WA 98101  UNITED STATES |
|  |  |  |  |  |  |
| 1134 | Dr. William Julius Shergy |  | Theresa Ann Causey  Dr. Kun Chen  Dr. Jesus Hernandez  Dr. Robert Edward Hunt  Dr. Robert Macon Phillips Jr. | Rheumatology Associates of North Alabama, P.C.  Suites 600 & 620  201 Sivley Road  Huntsville, AL 35801  UNITED STATES | Quorum Institutional Review Board  Suite 1000  1601 Fifth Avenue  Seattle, WA 98101  UNITED STATES |
|  |  |  |  |  |  |
