# Supplementary material for: Changes in serum creatinine in patients with active rheumatoid arthritis treated with tofacitinib: results from clinical trials
Source: Arthritis Res Ther. 2014 Jul 25;16(4):R158. doi: 10.1186/ar4673 (PMC4220634; doi:10.1186/ar4673)
Supplement: Supplementary file 10 — Additional file 10: List of Investigators and Corresponding Ethics Committees or Institutional Review Boards for the Phase 3 A3921045 study. (DOC 328 KB) [file 13075_2013_4378_MOESM10_ESM.doc]

# 16.1.4 LIST OF INVESTIGATORS AND CORRESPONDING ETHICS COMMITTEES OR INSTITUTIONAL REVIEW BOARDS

## Brazil

**Coordinating Investigators:**

<None Entered>

| **Center** | **Principal Investigator** | **Co-Investigator(s)** | **Sub-Investigator(s)** | **Address(es)** | **Institutional Review Board or Ethics Committee Address(es)** |
| --- | --- | --- | --- | --- | --- |
|  |  |  |  |  |  |
| 1042 | Dr. Cristiano Augusto de Freitas Zerbini |  | Dr. Marta E. C. Bastos  Lina Oliveira de Carvalho  Dr. Wagner Ikehara  Dr. Monique Sayuri Konai  Dr. Luiz Carlos Latorre  Dr. Andrea Barranjard Vannucci Lomonte  Dr. Silvia Caroline Santana Moura  Dr. Maria Jose Nunes  Dr. Lenise B. Pieruccetti  Dr. Luiza Helena Coutinho Ribeiro  Dr. Raissa Gomes da Silva  Dr. Mariana G. Waisberg | CEPIC - Centro Paulista de Investigacao Clinica e Servicos Medicos Ltda  Rua Moreira e Costa 342  Sao Paulo, SP 04266-010  BRAZIL | Hospital Heliopolis  Comite de Etica em Pesquisa  Rua Conego Xavier, 276. 10. andar Sacoma  Sao Paulo, SP 04231-030  BRAZIL |
|  |  |  |  |  |  |
| 1043 | Dr. Antonio Carlos Ximenes |  | Dr. Rafael Navarrete Fernandez  Dr. Fabia M.G.P. Oliveira  Dr. Marcelo Pimenta  Dr. Bruno Nazeozeno Ribeiro | CIP - Centro Internacional de Pesquisas  Rua 9 B, 129 - 3 andar  Setor Oeste  Goiania, GO 74110-120  BRAZIL | Comite de Etica em Pesquisa do Hospital Geral de Goiania - CEPHGG  Avenida Anhhaguera, 6479 - Setor Oeste  Goiania, GO 74110-010  BRAZIL |
|  |  |  |  |  |  |
| 1044 | Dr. Joao Carlos Tavares Brenol |  | Dr. Claiton Viegas Brenol  Dr. Odirlei A. Monticielo  Dr. Tamara M. Mucenic  Dra Iloite Maria Scheibel  Dr. Ricardo Machado Xavier | Hospital de Clinicas de Porto Alegre  Servico de Reumatologia  Rua Ramiro Barcelos, 2350- 6º andar - sala 645 A  Bom Fim  Porto Alegre, RS 90035-903  BRAZIL  Hospital de Clinicas de Porto Alegre  Grupo de Pesquisa  Rua Ramiro Barcelos 2350 2 andar  sala 2202  Porto Alegre, RS 90035-003  BRAZIL | Comite de Etica em Pesquisa em Seres Humanos do Hospital de Clinicas de Porto Alegre- HCPA  Rua Ramiro Barcelos 2350, 2 andar  Bom Fim  Porto Alegre, RS 90035-903  BRAZIL |
|  |  |  |  |  |  |
| 1045 | Dr. Sebastiao C. Radominski |  | Dr. Vivian B. Coginotti  Dr. Sinara da Silva Freitas  Dr. Andreas Funke  Dr. Lucila Stange Rezende  Dr. Alexandre G. Tavares  Dr. David Cezar Titton | Centro de Estudos em Terapias Inovadoras  Rua Padre Camargo, 241  Alto da Gloria  Curitiba, PR 80060-240  BRAZIL | Comitê de Ética em Pesquisa em Seres Humanos do Hospital de Clínicas - UFPR  Rua General Carneiro, 181  Curitiba, PR 80060-900  BRAZIL |
|  |  |  |  |  |  |
| 1046 | Dr. Ana Claudia Cauceglia Melazzi |  | Dr. Carolina A. Cabizuca  Dr. Alessandra Saldanha Matheus Fernandes Da Costa  Dr. Maria De Fatima Dias De Castro  Dr. Juliana Branco Dias  Dr. Luiz Henrique de Gregorio  Dr. Angela Frazão S. Linhares Hahn  Dr. Paulo Gustavo Sampaio Lacativa  Dr. Flavia S. Lessa  Dr. Tarso Lameri Sant anna Mosci  Dr. Renata Alexandra Calixto Pinheiro  Dr. Luis Augusto Tavares Russo  Dr. Pedro Moitrel Schwarts  Dr. Priscila Geller Wolff | CCBR Brasil  Centro de Pesquisas e Analises Clinicas Ltda.  Rua Mena Barreto, 33  Rio de Janeiro, RJ 22271-100  BRAZIL | Comite de Etica em Pesquisa do Hospital Pro Cardiaco Pronto Socorro Cardiologico/PROCEP  Rua Dona Mariana, 219  Rio de Janeiro, RJ 22280-000  BRAZIL |
|  |  |  |  |  |  |
| 1125 | Dr. Mauro W. Keiserman |  | Dr. Briele Keiserman  Dr. Tatiana Karenini Muller  Dr. Maria Mercedes Picarelli  Dr. Aline Defaveri do Prado  Caroline Z. Xavier de Freitas | Hospital Sao Lucas da PUCRS  Av. Ipiranga, 6690 - 4o andar  Porto Alegre, RS 90610-000  BRAZIL | Comite de Etica em Pesquisa da Pontificia Universidade Catolica do Rio Grande do Sul  Av. ipiranga 6690-Conj.314 -3 andar  Jardim Botanico  Porto Alegre, RS 90610-000  BRAZIL |
|  |  |  |  |  |  |
| 1126 | Dr. Luciana Teixeira Pinto |  | Camila Cristhine Bucchi  Dr. Marise Lazaretti Castro  Patricia Muszkat  Dr. Camila Albero Schiavon  Jeane Jeong Hoon Yang | IMA Brasil - Instituto de Medicina Avancada  Praca Americo Jacomino, 55  Vila Madalena  Sao Paulo, SP 05437-010  BRAZIL | Comite de Etica em Pesquisa do Instituto de Infectologia Emilio Ribas  Av. Dr. Arnaldo, 165  Sao Paulo, SP 01246-900  BRAZIL |
|  |  |  |  |  |  |

## Bulgaria

**Coordinating Investigators:**

<None Entered>

| **Center** | **Principal Investigator** | **Co-Investigator(s)** | **Sub-Investigator(s)** | **Address(es)** | **Institutional Review Board or Ethics Committee Address(es)** |
| --- | --- | --- | --- | --- | --- |
|  |  |  |  |  |  |
| 1093 | Dr. Boycho Oparanov |  | Dr. Borislava Angelova Ilchova  Dr. Raycho Raychev  Dr. Ignat Zhutev | MBAL na Voennomeditsinska Akademia - Sofia  Klinika po Revmatologia i Kardiologia  MMA HAT Sofia  Ul. "Georgi Sofiyski" 3  Sofia, 1606  BULGARIA | Ethic Committee for Multicenter Trials  Etichna komisiya za mnogocentrovi izpitvaniya  ul. "Damyan Gruev" 8  Sofia, 1303  BULGARIA  Komisiya po etika pri MBAL na Voennomeditsinska Akademia - Sofia/Ethics Commettee at MMA HAT-Sofia  MMA HAT-Sofia  Ul. Georgi Sofiyski  3  Sofia, 1606  BULGARIA |
|  |  |  |  |  |  |
| 1094 | Dr. Daniela Bichovska |  | Dr. Ivan Bichovski  Emilia F. Fileva-Veleva | DKTs "Sveta Anna" Sofia  Konsultativen Kabinet po Revmatologia  Diagnostic Consultative Center "Sveta Anna" Sofia  Ul. "Dimitar Mollov" 1  Sofia, 1709  BULGARIA | Ethic Committee for Multicenter Trials  Etichna komisiya za mnogocentrovi izpitvaniya  ul. "Damyan Gruev" 8  Sofia, 1303  BULGARIA  Komisiya po etika pri DKTs"Sv. Anna"/ Ethics Committee at DCC "Sv. Anna"  DKTs "Sveta Anna"  Diagnostic Consultative Center "Sveta Anna"  Ul. Dimitar Mollov 1  Sofia, 1709  BULGARIA |
|  |  |  |  |  |  |
| 1095 * | Prof. Rasho Rashkov |  | Dr. Penka Bekyarova  Dr. Daniela Dimitrova  Dr. Kameliya Ivanova Garbeva-Popova  Dr. Natalia Marinova  Dr. Lubomir Marinov Marintchev  Dr. Simeon Monov  Assoc. Prof. Veneta Paskaleva-Peytcheva  Dr. Tsvetelina Dimitrova Yoneva | MBAL "Sveti Ivan Rilski" Sofia  Klinika po Revmatologia  MHAT "Sveti Ivan Rilski"  Ul. "Urvich" 13  Sofia, 1612  BULGARIA | Ethic Committee for Multicenter Trials  Etichna komisiya za mnogocentrovi izpitvaniya  ul. "Damyan Gruev" 8  Sofia, 1303  BULGARIA |
|  |  |  |  |  |  |
| 1096 | Dr. Kiril Yablanski |  | Ivan Gerchev  Dr. Virzhiniya Yordanova | UMBAL "D-r Georgi Stranski" Pleven  Klinika po Kardiologia i Revmatologia  MHAT "Dr. Georgi Stranski" Pleven  Ul. "Georgi Kochev" 8A  Pleven, 5800  BULGARIA | Ethic Committee for Multicenter Trials  Etichna komisiya za mnogocentrovi izpitvaniya  ul. "Damyan Gruev" 8  Sofia, 1303  BULGARIA  Komisiya po etika pri UMBAL"D-r Georgi Stranski"/ Ethics Committee at MHAT "Dr. Georgi Stranski"  UMBAL"D-r Georgi Stranski" Pleven  MHAT "Dr. Georgi Stranski" Pleven  Ul. Georgi Kochev  8A  Pleven, 5800  BULGARIA |
|  |  |  |  |  |  |
| 1097 | Anastas Batalov |  | Dr. Rositsa Karalilova  Dr. Aneta Nikolova  Dr. Dimitar Penev | MBAL "Kaspela" Plovdiv, Otdelenie po revmatologia  MHAT "Kaspela" Plovdiv  ul. Sofiya  64  Plovdiv, 4002  BULGARIA | Ethic Committee for Multicenter Trials  Etichna komisiya za mnogocentrovi izpitvaniya  ul. "Damyan Gruev" 8  Sofia, 1303  BULGARIA  Komisiya po etika kam MBAL "Kaspela"/ Ethics committee at MHAT "Kaspela"  MBAL 'Kaspela' Plovdiv  MHAT 'Kaspela' Plovdiv  Ul. Sofiya 64  Plovdiv, 4002  BULGARIA |
|  |  |  |  |  |  |
| 1122 | Dr. Ivan Goranov |  | Katya Kuchmova  Mariana Panayotova | MBAL - Plovdiv  Revmatologichno Otdelenie  MHAT - Plovdiv  bul. "Bulgaria" 234  Plovdiv, 4000  BULGARIA | Ethic Committee for Multicenter Trials  Etichna komisiya za mnogocentrovi izpitvaniya  ul. "Damyan Gruev" 8  Sofia, 1303  BULGARIA  Komisiya po etika pri MBAL-Plovdiv/Ethics Committee at MHAT-Plovdiv  MBAL-Plovdiv  MHAT-Plovdiv  bul. Bulgaria 234  Plovdiv, 4000  BULGARIA |
|  |  |  |  |  |  |

## Chile

**Coordinating Investigators:**

<None Entered>

| **Center** | **Principal Investigator** | **Co-Investigator(s)** | **Sub-Investigator(s)** | **Address(es)** | **Institutional Review Board or Ethics Committee Address(es)** |
| --- | --- | --- | --- | --- | --- |
|  |  |  |  |  |  |
| 1072 | Dr. Marta Aliste |  | Dr. Julio Cruz  Dr. Hector Gatica  Dr. Sandra Pino  Dr. Francisca Sabugo  Dr. Lilian Soto  Dr. Pamela Wurmann | Consulta Privada Dra. Marta Aliste  Guardia Vieja 255, Oficina 1409  Providencia  Santiago, RM 7510186  CHILE | Comite Etico Cientifico  Servicio de Salud Metropolitano Oriente  Av. Salvador 364  Providencia, Santiago, RM 7500922  CHILE |
|  |  |  |  |  |  |
| 1073 | Dr. Francisco Ballesteros |  | Dr. Maria Eugenia Alvarez  Dr. Loreto Ovalle  Dr. Paula Pozo | Centro de Diagnostico y Tratamiento San Borja Arriaran  Seccion Reumatologia  Amazonas 619  Santiago, RM 8360156  CHILE | Comite Etico Cientifico  Servicio de Salud Metropolitano Oriente  Av. Salvador 364  Providencia, Santiago, RM 7500922  CHILE |
|  |  |  |  |  |  |
| 1074 | Dr. Gloria Holuigue |  | Juan Manuel Contreras  Luis Orellana | Clínica Santa María, Sección Reumatología  Fernando Manterola 0540  Providencia, Santiago, RM 7530206  CHILE | Comite de Etica  Clinica Santa Maria  Avenida Santa Maria 0410  Santiago,  CHILE  Comite Etico Cientifico  Servicio de Salud Metropolitano Oriente  Av. Salvador 364  Providencia, Santiago, RM 7500922  CHILE |
|  |  |  |  |  |  |

## Colombia

**Coordinating Investigators:**

<None Entered>

| **Center** | **Principal Investigator** | **Co-Investigator(s)** | **Sub-Investigator(s)** | **Address(es)** | **Institutional Review Board or Ethics Committee Address(es)** |
| --- | --- | --- | --- | --- | --- |
|  |  |  |  |  |  |
| 1092 | Dr. William Jose Otero Escalante MD |  | Dr. Marcial Martinez  Dr. Gerardo Ramirez MD | SERVIMED E.U  Calle 51# 34-17 Consultorio 208-208A Centro Comercial Cabecera. Etapa I  Bucaramanga, Santander  COLOMBIA | Comite de ética en Investigación de Servimed E.U  Calle 51 No. 34-17. Centro comercial Cabecera Etapa I.  Bucaramanga, Santander  COLOMBIA |
|  |  |  |  |  |  |
| 1135 | Dr. Juan J. Jaller Raad |  | Dr. Javier Cuartas  Dr. Anubys Maiguel  Dr. Victor Andres Ulloque Lopez | Centro de Reumatologia y Ortopedia  Cra 49 C No 82-125  Barranquilla,  COLOMBIA | Comité de etica independiente centro de reumatologia y ortopedia  Cr. 49C No. 82-120  Barranquilla, Atlantico 0000  COLOMBIA |
|  |  |  |  |  |  |

## Czech Republic

**Coordinating Investigators:**

<None Entered>

| **Center** | **Principal Investigator** | **Co-Investigator(s)** | **Sub-Investigator(s)** | **Address(es)** | **Institutional Review Board or Ethics Committee Address(es)** |
| --- | --- | --- | --- | --- | --- |
|  |  |  |  |  |  |
| 1103 | Dr. Zuzana Urbanova |  | Dr. Zuzana Stejfova | Revmatologicka ambulance  Petra Rezka 3  Praha 4, 140 00  CZECH REPUBLIC | Eticka komise IKEM a FTNsP  Videnska 800  Praha 4 Krc, 140 59  CZECH REPUBLIC |
|  |  |  |  |  |  |
| 1104 | Dr. Zuzana Stejfova |  | Dr. Zuzana Urbanova | Revmatologicka ambulance  Nuselska poliklinika  Taborska 57  Praha 4, 140 00  CZECH REPUBLIC | Eticka komise IKEM a FTNsP  Videnska 800  Praha 4 Krc, 140 59  CZECH REPUBLIC |
|  |  |  |  |  |  |
| 1105 | Dr. Libor Novosad |  | Jana Jaluvkova | L.K.N. Arthrocentrum, s.r.o.  Revmatologicka ambulance  Na Valech 1  Hlucin, 748 01  CZECH REPUBLIC | Eticka komise pri Fak. Thomayerove nemocnici a IKEM  Videnska 800  Praha 4, 140 59  CZECH REPUBLIC |
|  |  |  |  |  |  |
| 1106 | Dr. Petr Vitek |  | Dr. Olga Januskova  Eva Vitkova | PV-Medical s.r.o.  Revmatologicka ambulance  Padelky I/3645  Zlin, 760 01  CZECH REPUBLIC | Eticka komise IKEM a FTNsP  Videnska 800  Praha 4 Krc, 140 59  CZECH REPUBLIC |
|  |  |  |  |  |  |
| 1107 | Dr. Zdenek Dvorak |  | Lucie Koubova  Dr. Martina Vaneckova | ARTHROMED, s. r. o. Revmatologicka ambulance  Rokycanova 2798  Pardubice, 530 02  CZECH REPUBLIC | Eticka komise IKEM a FTNsP  Videnska 800  Praha 4 Krc, 140 59  CZECH REPUBLIC |
|  |  |  |  |  |  |
| 1113 | Prof. Jiri Vencovsky |  | Dr. Sarka Forejtova  Dr. Katerina Jarosova  Dr. Dana Tegzova | Revmatologicky ustav  Na Slupi 4  Praha 2, 128 50  CZECH REPUBLIC | Eticka komise  Revmatologicky ustav  Na Slupi 4  Praha 2, 128 50  CZECH REPUBLIC  Eticka komise IKEM a FTNsP  Videnska 800  Praha 4 Krc, 140 59  CZECH REPUBLIC |
|  |  |  |  |  |  |
| 1119 | Dr. Zdenek Fojtik |  | Dr. Libor Cervinek  Dr. Monika Obrovska | FN Brno, Interni hematoonkologická klinika  Revmatologicka ambulance  Jihlavska 20  Brno, 625 00  CZECH REPUBLIC | Eticka komise FN Brno  Jihlavská 20  Brno, 625 00  CZECH REPUBLIC  Eticka komise IKEM a FTNsP  Videnska 800  Praha 4 Krc, 140 59  CZECH REPUBLIC |
|  |  |  |  |  |  |
| 1120 | Dr. Helena Stehlikova |  | Dr. Dana Pospisilova  Dr. Roman Zahora | Revmatologie  Pod Holym vrchem 349  Ceska Lipa, 470 01  CZECH REPUBLIC | Eticka komise IKEM a FTNsP  Videnska 800  Praha 4 Krc, 140 59  CZECH REPUBLIC |
|  |  |  |  |  |  |
| 1123 | Dr. Petr Kopsa |  | Dr. Lenka Zouharova | Fakultni Thomayerova nemocnice s poliklinikou  Revmatologicke a rehabilitacni oddeleni  Videnska 800  Praha 4, 140 59  CZECH REPUBLIC | Eticka komise IKEM a FTNsP  Videnska 800  Praha 4 Krc, 140 59  CZECH REPUBLIC |
|  |  |  |  |  |  |

## Dominican Republic

**Coordinating Investigators:**

<None Entered>

| **Center** | **Principal Investigator** | **Co-Investigator(s)** | **Sub-Investigator(s)** | **Address(es)** | **Institutional Review Board or Ethics Committee Address(es)** |
| --- | --- | --- | --- | --- | --- |
|  |  |  |  |  |  |
| 1020 | Patricia Alvarez-Felix |  | Rafael Alba-Ferriz  Iraquel Cordero  Roberto Munoz-Louis  Miriam Nova  Cinthia Rivas | Latin American Research  Avenida Maximo Gomez #60, suite 201, plaza paseo del treatro  Santo Domingo, Santo Domingo 00000  DOMINICAN REPUBLIC | CONABIOS  Universidad Católica Santo Domingo  Calle Santo Domingo No3, Ens. La Julia  Santo Domingo, Santo Domingo 2733  DOMINICAN REPUBLIC |
|  |  |  |  |  |  |

## Germany

**Coordinating Investigators:**

<None Entered>

| **Center** | **Principal Investigator** | **Co-Investigator(s)** | **Sub-Investigator(s)** | **Address(es)** | **Institutional Review Board or Ethics Committee Address(es)** |
| --- | --- | --- | --- | --- | --- |
|  |  |  |  |  |  |
| 1066 | Prof. Dr. med. Christoph Baerwald |  | Dr. Sybille Arnold  Dr. Olga Malysheva  Dr. Matthias Pierer  Prof. Dr. med. Ulf Wagner | Universitaetsklinik Leipzig, Klinik fuer Gastroenterologie und Rheumatologie, Sektion Rheumatologie  Liebigstr. 20  Leipzig, 04103  GERMANY | Ethik-Kommission an der Medizinischen Fakultaet der Universitaet Leipzig  Haertelstrasse 16-18  Leipzig, 04107  GERMANY |
|  |  |  |  |  |  |
| 1067 | Dr. med. Rieke Alten |  | Dr. med. Stefan Bieneck  Svitlana Djacenko  Dr. med. Christoph Pohl | Schlosspark-Klinik, Innere Medizin II, Rheumatologie  Heubnerweg 2  Berlin, 14059  GERMANY | Ethik-Kommission an der Medizinischen Fakultaet der Universitaet Leipzig  Haertelstrasse 16-18  Leipzig, 04107  GERMANY |
|  |  |  |  |  |  |
| 1068 | Prof. Dr. med. Juergen Wollenhaupt |  | Dr. med. Andrea Everding  Dr. med. Ulrike Schnoor | Schoen Klinik Hamburg - Eilbek, Abt. Rheumatologie und Klin. Immunologie  Dehnhaide 120  Hamburg, 22081  GERMANY | Ethik-Kommission an der Medizinischen Fakultaet der Universitaet Leipzig  Haertelstrasse 16-18  Leipzig, 04107  GERMANY |
|  |  |  |  |  |  |
| 1069 | Prof. Dr. med. Hendrik Schulze-Koops |  | Dr. med. Mathias Gruenke  Dr. med. Matthias Witt | Klinikum der Universitaet Muenchen, Campus Innenstadt  Pettenkoferstr. 8a  Muenchen, 80336  GERMANY | Ethik-Kommission an der Medizinischen Fakultaet der Universitaet Leipzig  Haertelstrasse 16-18  Leipzig, 04107  GERMANY |
|  |  |  |  |  |  |
| 1070 | Prof. Dr. med. Hubert Nuesslein |  |  | Arztpraxis, Internist - Rheumatologie  Kontumazgarten 4  Nuernberg, 90429  GERMANY | Ethik-Kommission an der Medizinischen Fakultaet der Universitaet Leipzig  Haertelstrasse 16-18  Leipzig, 04107  GERMANY |
|  |  |  |  |  |  |
| 1137 | Dr. med. Sylke Wagner |  | Dr. med. Thomas Linde | FAE Innere Medizin / Rheumatologie  Ludwig-Wucherer-Str. 10  Halle, 06108  GERMANY | Ethik-Kommission an der Medizinischen Fakultaet der Universitaet Leipzig  Haertelstrasse 16-18  Leipzig, 04107  GERMANY |
|  |  |  |  |  |  |

## India

**Coordinating Investigators:**

<None Entered>

| **Center** | **Principal Investigator** | **Co-Investigator(s)** | **Sub-Investigator(s)** | **Address(es)** | **Institutional Review Board or Ethics Committee Address(es)** |
| --- | --- | --- | --- | --- | --- |
|  |  |  |  |  |  |
| 1030 | Dr. Vineeta Shobha |  | Dr. Cecil Ross  Dr. Soumya Umesh | St. John's Medical College Hospital  Sarjapur Road  Bangalore, Karnataka 560 034  INDIA | Institutional Ethical Review Board  St. John's Medical College and Hospital  Sarjapur Road  Bangalore, Karnataka 560 034  INDIA |
|  |  |  |  |  |  |
| 1031 | Dr. Manoj Kumar Venkataiya Honnakere |  | Ms. Keerthana Loganathan  Dr. Suresh Manohar  Dr. Krishnegowda Ravi  Dr. Siddarahalli Somashekara | Bowring and Lady Curzon Hospitals  Shivaji Nagar  Bangalore, Karnataka 560 001  INDIA | Ethical Committee, Bangalore Medical College & Research Institute  K.R. Road,  Fort,  Bangalore, Karnataka 560 002  INDIA |
|  |  |  |  |  |  |
| 1032 | Dr. Srikantiah Chandrashekara |  | Mr. Raghunandan Bommanna  Dr. Jalaja Ganganna  Dr. Sneha Ramachandra Kulkarni  Mr. Sasi Kumar  Dr. Renuka Panchagnula  Dr. Radhika Sreeramappa | Chanre Rheumatology & Immunology Centre & Research  #149, 15th Main NHCL  Water Tank Road  4th Block, 3rd Stage, Basaveswaranagar  Bangalore, Karnataka 560 079  INDIA | Institutional Ethical Committee  Chanre Rheumatology & Immunology Centre & Research  #149, 15th Main NHCL, Water Tank Road, 4th Block, 3rd Stage  Basaveswaranagar  Bangalore, Karnataka 560 079  INDIA |
|  |  |  |  |  |  |
| 1033 * | Dr. Shrikant Wagh |  | Dr. Ashish Goyal  Dr. Nuzhat Shaikh  Dr. Ratnamala Swami | Jehangir Clinical Development Centre Pvt. Ltd.  Jehangir Hospital  32, Sassoon Road  Pune, Maharashtra 411 001  INDIA | Hirabai Cowasji Jehangir Medical Research Institute and Jehangir Clinical Development Center  Ethics Committee  Jehangir Hospital,  32, Sassoon Road,  Pune, Maharashtra 411 001  INDIA |
|  |  |  |  |  |  |
| 1035 | Dr. Sarath Chandra Mouli Veeravalli |  | Dr. Ravi Kumar Neela  Ms. Sunitha Rani Pagidipalli  Dr. Sreenivas Reddy Pesaru  Dr. Namrata Sridhar | Krishna Institute of Medical Sciences Ltd  1-8-31/1  Minister Road  Secunderabad, Andra Pradesh 500 003  INDIA | Institutional Ethics Committee  Krishna Institute of Medical Sciences Ltd.  1-8-31/1, Minister Road  Secunderabad, Andra Pradesh 500003  INDIA |
|  |  |  |  |  |  |
| 1038 | Dr. Arvind K. Chopra |  | Dr. Sharon Arthur  Dr. Vaijayanti Vardhan Lagu-Joshi  Dr. Sheetal S. Salvi | Arthritis Research and Care Foundation  Centre for Rheumatic Diseases  No. 11, Hermes Elegance  1988, Convent Street Camp  Pune, Maharashtra 411 001  INDIA | CRD Ethics Committee  11, Hermes Elegance  1988, Convent Street Camp  Pune, Maharashtra 411 001  INDIA |
|  |  |  |  |  |  |
| 1088 | Dr. Vikram I Shah |  | Dr. Govind Agrawal  Dr. Pankaj Doshi  Dr. Ankur Mahindroo  Dr. Utsav Mehta  Ms. Ankita Panchal  Dr. Ashish Saxena  Dr. Kinjal Shah  Dr. Reena Sharma  Dr. Daria Singh | Shalby Hospitals  Opp. Karnavati Club  S.G. Road  P.O. Ambawadi Vistar  Ahmedabad, Gujarat 380 015  INDIA | Ethics Committee Shalby Hospitals  Shalby Hospitals  Opp. Karnavati Club,  S. G. Road, P. O. Ambawadi Vistar,  Ahmedabad, Gujarat 380 015  INDIA |
|  |  |  |  |  |  |
| 1099 | Dr. Prabha Adhikari |  | Dr. Keshava Bhat  Dr. Sydney D'souza  Dr. Susan D'souza  Dr. Vishal Garg  Dr. Ramachandra K. Kamath  Dr. Deepak R. Madi  Dr. Basavaprabhu Nagabhushana  Dr. John Thomas Ramapuram  Dr. Shalini Rao  Dr. Satish B. Rao  Dr. Raghava Sharma  Dr. Pradeep Shenoy  Dr. Ashok K. Shenoy  Dr. Tarun Varma | Kasturba Medical College Hospital  Attavar  Mangalore, Karnataka 575 001  INDIA | Manipal University Ethics Cimmittee  Madhav Nagar  Manipal, karnataka 576 104  INDIA |
|  |  |  |  |  |  |
| 1114 | Dr. Jacob Chacko |  | Dr. Latheesh Latheef  Mr. Anjan Kumar Vuriya | Father Muller Medical College  Fr. Muller Road,  Kankanady  Mangalore, Karnataka 575002  INDIA | Institutional Ethics Committee  Father Muller Medical College  Father Muller Road  Kankanady  Mangalore, Karnataka 575 002  INDIA |
|  |  |  |  |  |  |
| 1128 | Dr. Uppuluri Ramakrishna Rao |  | Ms. Shashikala Arava  Dr. Firdaus Fatima  Mr. Venkata Subrahamanya Sarma Valiveti | Sri Deepti Rheumatology Centre  6-2-45/8,  A.C.Guards,  Hyderabad, Andhra Pradesh 500 004  INDIA | Ethics Committee  Sri Deepti Rheumatology Centre  6-2-45/8,  A. C. Guards,  Hyderabad, Andhra Pradesh 500 004  INDIA |
|  |  |  |  |  |  |
| 1130 | Dr. Jugal Kishore Kadel |  | Dr. Manik Dixit  Dr. Sindhu Joshi  Ms. Madhuri Mahajan | Mahavir Hospital & Research Center  10-1-1, Bhagwan Mahavir Marg  A.C. Guards  Hyderabad, Andhra Pradesh 500 004  INDIA | Institutional Ethical Committee for Bio Medical Research  Bhagwan Mahavir Medical Research Center, Mahavir Hospital & Research Center,  10-1-1, Bhagwan Mahavir Marg,  A.C. Guards,  Hyderabad, Andhra Pradesh 500 004  INDIA |
|  |  |  |  |  |  |

## Malaysia

**Coordinating Investigators:**

<None Entered>

| **Center** | **Principal Investigator** | **Co-Investigator(s)** | **Sub-Investigator(s)** | **Address(es)** | **Institutional Review Board or Ethics Committee Address(es)** |
| --- | --- | --- | --- | --- | --- |
|  |  |  |  |  |  |
| 1058 | Dr. Cheng Lay Teh |  | Dr. Wai Hoong Chan  Yin Yin Angela Chia  Dr. Jun Lee  Dr. Jin Shyan Wong | Sarawak General Hospital  Jalan Hospital  Kuching, Sarawak 93586  MALAYSIA | Medical Research & Ethics Committee  Ministry of Health, c/o NIH Secretariat, Institute for Health Management  Jalan Rumah Sakit  Bangsar  Kuala Lumpur, 59000  MALAYSIA |
|  |  |  |  |  |  |
| 1060 | Dr. Yun Yin Chong |  | Dr. Anna Farazilah Mohammad Salleh  Dr. Rachel Joshua Thundyil  Dr Teck Huat Wong | Queen Elizabeth Hospital  Kota Kinabalu, Sabah 88586  MALAYSIA | Medical Research & Ethics Committee  Ministry of Health, c/o NIH Secretariat, Institute for Health Management  Jalan Rumah Sakit  Bangsar  Kuala Lumpur, 59000  MALAYSIA |
|  |  |  |  |  |  |
| 1079 | Dr Heselynn Hussein |  | Dr. Fazirah Abdullah  Dr Eashwary Mageswaren  Dr. Liza Mohd Isa  Dr. Shamala Rajalingam | Hospital Putrajaya  Federal Government Administration Centre  Presint 7  Putrajaya, Wilayah Persekutuan 62250  MALAYSIA | Medical Research & Ethics Committee  Ministry of Health, c/o NIH Secretariat, Institute for Health Management  Jalan Rumah Sakit  Bangsar  Kuala Lumpur, 59000  MALAYSIA |
|  |  |  |  |  |  |
| 1080 | Dr. Sook Khuan Chow |  | Dr. Suresh V. Nainan George  Dr. Suren Thuraisingham | Sunway Medical Centre  No. 5, Jalan Lagoon Selatan  Bandar Sunway  Petaling Jaya, Selangor Darul Ehsan 46150  MALAYSIA | Medical Research & Ethics Committee  Ministry of Health, c/o NIH Secretariat, Institute for Health Management  Jalan Rumah Sakit  Bangsar  Kuala Lumpur, 59000  MALAYSIA |
|  |  |  |  |  |  |

## Mexico

**Coordinating Investigators:**

<None Entered>

| **Center** | **Principal Investigator** | **Co-Investigator(s)** | **Sub-Investigator(s)** | **Address(es)** | **Institutional Review Board or Ethics Committee Address(es)** |
| --- | --- | --- | --- | --- | --- |
|  |  |  |  |  |  |
| 1053 | Dr. Cesar Pacheco-Tena |  | Dr. Edgardo Munoz-Esteves  Dr. Hugo Parra-Ruiz  Dra. Adelfia Urenda-Quezada  Dr. Omar Villarreal Dominguez | Hospital Christus Muguerza del Parque  Calle 14 1610 A Colonia Centro  Chihuahua, Chihuahua 31000  MEXICO | Comite de Etica e Investigacion del Hospital Christus Muguerza del Parque  Calle Dr. Pedro Leal Rodriguez y de la Llave  Chihuahua, Chihuahua 31000  MEXICO |
|  |  |  |  |  |  |
| 1055 | Dr. Mario A. Garza-Elizondo |  | Dr. Jorge Antonio Esquivel-Valerio  Dra. Diana Elsa Flores-Alvarado  Dra. Jacqueline Rodriguez-Amado  Dra. Cassandra Michelle Skinner-Taylor  Dra. Brenda Vazquez | Hospital Universitario Jose Eleuterio Gonzalez  Gonzalitos 235 Norte  Colonia Mitras Centro  Monterrey, Nuevo Leon 64020  MEXICO | Comite de Etica Facultad de Medicina de la UANL y Hospital Universitario Dr. Jose Eleuterio Gonzalez  Av. Francisco I Madero Pte s/n y Dr. E Aguirre Pequeno  Col. Mitras Centro  Monterrey, Nuevo Leon 64460  MEXICO |
|  |  |  |  |  |  |
| 1056 | Dr. Diego Cesar-Ricardo Ramos-Remus |  | Sergio Duran-Barragan  Dr. Guillermo Hernandez-Rios  Dr. Adriana Sanchez-Ortiz | Unidad de Investigacion en Enfermedades Cronico Degenerativas  Colomos 2292  Colonia Providencia  Guadalajara, Jalisco 44620  MEXICO | Comite de Bioetica de la Unidad de Investigacion en Enfermedades Cronico-Degenerativas  Colomos 2292  Col. Providencia  Guadalajara, Jalisco 44620  MEXICO |
|  |  |  |  |  |  |
| 1057 | Dr. Jesus Ernesto Santana-Sahagun |  | Carla Yolanda Corti-Saldate  Francis B. Gabbai-Laval  Alma Guadalupe Lopez-Beltran  Dr. Maria Cristina Saldate-Alonso | Centro de Investigacion del Noroeste SC  Boulevard Sanchez Taboada 9250 Interior 28  Zona Rio  Tijuana, Baja California 22010  MEXICO | Comision de Investigacion y Etica Centro Medico Nova  Avenida Guadalupe Victoria 9308 Zona Rio  Tijuana, Baja California 22010  MEXICO |

## Philippines

**Coordinating Investigators:**

<None Entered>

| **Center** | **Principal Investigator** | **Co-Investigator(s)** | **Sub-Investigator(s)** | **Address(es)** | **Institutional Review Board or Ethics Committee Address(es)** |
| --- | --- | --- | --- | --- | --- |
|  |  |  |  |  |  |
| 1047 | Dr. Sandra T.G.V. Navarra |  | Dr. Ginger Alden I. Cabasan  Dr. Maria Cristina C. Tolin  Dr. Eugene A. Uy | University of Santo Tomas Hospital  6th Floor Hospital Research Center  Clinical Division Building  Espana Street  Manila, Phlippines 1008  PHILIPPINES | Institutional Review Board  3rd Floor, Clinical Division Building  University of Sto. Tomas Hospital  Espana Blvd.  Manila, 1008  PHILIPPINES |
|  |  |  |  |  |  |
| 1048 | Dr. Emmanuel C. Perez |  | Dr. Andrei Rhoneil M. Rodriguez  Loida Cruz Torres | University of Perpetual Help Rizal Dalta Medical Center  7th Floor  Research Room  Alabang-Zapote Road, Pamplona  Las Piñas City, 1742  PHILIPPINES | Institutional Ethics Review Board  Institutional Ethics Review Board  University of Perpetual Help System-DALTA 7th Floor Research Room  Alabang-Zapote Road  Las Piñas City, 1742  PHILIPPINES |
|  |  |  |  |  |  |
| 1049 | Dr. Auxencio A. Lucero Jr. |  | Dr. Jenny Rubio Bicol  Dr. Evelyn Salido | De La Salle Health Sciences Campus- Clinical Epidemiology Unit  2nd Floor Clinical Trial Room#2  The Angelo King Medical Research Center  Congressional Avenue Dasmarinas  Cavite, Phlippines 4114  PHILIPPINES | DLS Institutional Review Board  Room 6301  De La Salle Angelo King Medical Research Center  Congressional Avenue  Dasmariñas, Cavite 4114  PHILIPPINES |
|  |  |  |  |  |  |
| 1050 | Dr. Edgar B. Ramiterre |  | Maria Aurora Narisma | Southern Philippines Medical Center  Section of Rheumatology Department of Internal Medicine  Bajada, Davao City, Phlippines 8000  PHILIPPINES | Ethics Committee  Southern Philippines Medical Center  Bajada, Davao City, 8000  PHILIPPINES |
|  |  |  |  |  |  |

## Poland

**Coordinating Investigators:**

<None Entered>

| **Center** | **Principal Investigator** | **Co-Investigator(s)** | **Sub-Investigator(s)** | **Address(es)** | **Institutional Review Board or Ethics Committee Address(es)** |
| --- | --- | --- | --- | --- | --- |
|  |  |  |  |  |  |
| 1021 | Dr. Andrzej Sawicki (Previous PI)  Dr. Ines Pokrzywnicka-Gajek |  | Dr. Ewa Czernecka  Dr. Dorota Knychas  Dr. Andrzej Sawicki  Dr. Malgorzata Szymanska  Dr. Alina Walczak | Lecznica Specjalistow, Centrum Medyczne "Osteomed" NZOZ  Al. Krakowska 110/114  Warszawa, 02-256  POLAND | Komisja Bioetyczna przy Okregowej Izbie Lekarskiej w Warszawie  ul. Pulawska 18  Warszawa, 02-512  POLAND |
|  |  |  |  |  |  |
| 1022 | Dr. Zofia Ruzga |  | Dr. Ewa Jazwinska-Tarnawska  Ewa Krecipro-Nizinska  Dr. Anna Sidorowicz-Bialynicka | "SYNEXUS SCM" Sp. z o.o.  ul. Swobodna 8a  Wroclaw, 50-088  POLAND | Komisja Bioetyczna przy Okregowej Izbie Lekarskiej w Warszawie  ul. Pulawska 18  Warszawa, 02-512  POLAND |
|  |  |  |  |  |  |

## Russian Federation

**Coordinating Investigators:**

<None Entered>

| **Center** | **Principal Investigator** | **Co-Investigator(s)** | **Sub-Investigator(s)** | **Address(es)** | **Institutional Review Board or Ethics Committee Address(es)** |
| --- | --- | --- | --- | --- | --- |
|  |  |  |  |  |  |
| 1100 | Dr. Olga U. Stetsiouk |  | Dr. Irina V. Andreeva  Dr. Denis V. Ilyin  Dr. Natalya Yu. Khozyainova  Dr. Tatyana A. Korolyova | State Educational Institution of Higher professional education Smolensk State Medical Academy  Roszdrav, Clinical Research Centre of diagnostic medicine and drugs  Krupskoy str., 28  Smolensk, 214019  RUSSIAN FEDERATION | Ethics Committee at the Federal Service on Surveillance in Healthcare and Social Development  8, str. 2, Petrovskij bulvar  Moscow, 127051  RUSSIAN FEDERATION  Independent Ethics Committee of State Educational Institution of High Professional Education  "Smolensk State Medical Academy of Federal Agency of Healthcare and Social Development"  28, ul. Krupskoj, 214019  27, pr. Gagarina  Smolensk, 214018  RUSSIAN FEDERATION |
|  |  |  |  |  |  |
| 1127 | Dr. Irina Mihailovna Marusenko |  | Dr. Yanina A. Avdeeva  Dr. Svetlana N. Kondrichina  Dr. Nina V. Koryakova  Dr. Irina I. Polskaya  Dr. Natalia N. Vezikova | Republican Hospital n. a. V.A.Baranov  Pirogova str., 3  Petrozavodsk, 185019  RUSSIAN FEDERATION | Ethics Committee at the Federal Service on Surveillance in Healthcare and Social Development  8, str. 2, Petrovskij bulvar  Moscow, 127051  RUSSIAN FEDERATION |
|  |  |  |  |  |  |

## Ukraine

**Coordinating Investigators:**

<None Entered>

| **Center** | **Principal Investigator** | **Co-Investigator(s)** | **Sub-Investigator(s)** | **Address(es)** | **Institutional Review Board or Ethics Committee Address(es)** |
| --- | --- | --- | --- | --- | --- |
|  |  |  |  |  |  |
| 1082 | Vira Iosypivna Tseluyko |  | Dr. Ol'ha Victorivna Radchenko  Dr. Viktoriya Victorivna Yarosh | City Clinical Hospital # 8, Dept of Cardiology and Functional Diagnostics  266g, Saltivske Shosse  Kharkiv, 61178  UKRAINE | Central Ethics Committee Ministry of Health of Ukraine  5, Narodnogo Opolchennya Str.  Kyiv, 03680  UKRAINE  Committee for Ethics Issues of City Clinical Hospital #8  266g, Saltivske Shosse  Kharkiv, 61178  UKRAINE |
|  |  |  |  |  |  |
| 1083 | Prof. Mykola A. Stanislavchuk |  | Dr. Nabil Sh. Ali  Dr. Olena O. Savytska  Dr. Natalia V. Shcolina | Vinnitsa Regional Clinical Hospital n.a. Pirogov, Dept of Internal Medicine #1 of Vinnitsa NMU  46 Pirogova Street  Vinnitsa, 21018  UKRAINE | Bioethics Committee of Vinnitsa Regional Clinical Hospital n.a. Pirogov  46 Pirogova Street  Vinnitsa, 21018  UKRAINE  Central Ethics Committee Ministry of Health of Ukraine  5, Narodnogo Opolchennya Str.  Kyiv, 03680  UKRAINE |
|  |  |  |  |  |  |
| 1084 | Prof. Vladyslav V. Povoroznyuk |  | Dr. Nataliia V. Grygorieva  Dr. Tetyana A. Karasevska  Dr. Tetyana V. Orlyk | Institute of Gerontology, Department of Clinical Physiology and Pathology of Musculoskeletal System  67 Vyshgorodska Street  Kyiv, 04114  UKRAINE | Central Ethics Committee Ministry of Health of Ukraine  5, Narodnogo Opolchennya Str.  Kyiv, 03680  UKRAINE  Committee for Ethics Issues of Institute of Gerontology  67, Vyshgorodska Street  Kyiv, 04114  UKRAINE |
|  |  |  |  |  |  |
| 1085 | Dr. Halyna M. Hrytsenko |  | Nataliya O. Smoley | Municipal City Clinical Hospital #4  Department of Rheumatology  3 Sventsitskogo Street  Lviv, 79011  UKRAINE | Central Ethics Committee Ministry of Health of Ukraine  5, Narodnogo Opolchennya Str.  Kyiv, 03680  UKRAINE  Committee for Ethics Issues of Municipal City Clinical Hospital #4  3 Sventsitskogo Str  Lviv, 79011  UKRAINE |
|  |  |  |  |  |  |
| 1086 | Andriy Petrov |  | Dr. Ganna A. Alekseeva  Volodymyr Biloglazov  Dr. Galyna M. Koshukova | Republican Clinical Hospital  Dept of Internal Medicine #2 of SI "Crimean State Medical University n.a. S.I. Georgiyevskyj"  69, Kyivska Street  Simferopol, Crimea, 95017  UKRAINE | Central Ethics Committee Ministry of Health of Ukraine  5, Narodnogo Opolchennya Str.  Kyiv, 03680  UKRAINE  Committee for Ethics Issues of Republican Clinical Hospital  69, Kyivska Street  Simferopol, Crimea 95017  UKRAINE |
|  |  |  |  |  |  |

## United States

**Coordinating Investigators:**

<None Entered>

| **Center** | **Principal Investigator** | **Co-Investigator(s)** | **Sub-Investigator(s)** | **Address(es)** | **Institutional Review Board or Ethics Committee Address(es)** |
| --- | --- | --- | --- | --- | --- |
|  |  |  |  |  |  |
| 1002 | Dr. Robert Michael Griffin Jr. |  | Dr. Michael Allen Borofsky  Brent William Calhoon  Jane Crosby  Dr. Saurin M. Mehta  Dr. Peter Daniel Nicholas Jr.  Dr. Nancy Jane Walker  Dr. Jerome Stephen Weisberg | Clinical Research Center of Reading, LLP  2760 Century Boulevard  Wyomissing, PA 19610  UNITED STATES | Quorum Institutional Review Board  Suite 1000  1601 Fifth Avenue  Seattle, WA 98101  UNITED STATES |
|  |  |  |  |  |  |
| 1003 | Dr. Seppo E. Rapo |  | Dr. John F. Berry  Dr. David J. Brown  Dr. Benjamin Dichter Gordon  Dr. Kathleen A. Kerrigan  Dr. Herbert O. Mathewson  Dr. James Arthur McCarthy | Clinical Research Center of Cape Cod, Inc.  131 Attucks Lane  Hyannis, MA 02601  UNITED STATES | Quorum Institutional Review Board  Suite 1000  1601 Fifth Avenue  Seattle, WA 98101  UNITED STATES |
|  |  |  |  |  |  |
| 1004 | Dr. Charles L. Ludivico |  | Dr. Susan Marshall Durkin  Nancy Katherine McFadden  Maxine R. Paden  Erica L. Rau  Dr. Allen Jeffrey Samuels  Dr. Ranju Singh | East Penn Rheumatology Associates, PC  Suite 501 & 601  701 Ostrum Street  Bethlehem, PA 18015  UNITED STATES | Quorum Institutional Review Board  Suite 1000  1601 Fifth Avenue  Seattle, WA 98101  UNITED STATES |
|  |  |  |  |  |  |
| 1005 | Dr. David Hilton Sikes |  | Dr. Mark Sol Eisner  Dr. Natalie A. Faith  Nathan A. Meyer  Michelle L. Meyer  Marokhaya Samb  Dr. Amarilis Torres | Florida Medical Clinic  Clinical Research Division  38135 Market Square  Zephyr Hills, FL 33542  UNITED STATES | Quorum Institutional Review Board  Suite 1000  1601 Fifth Avenue  Seattle, WA 98101  UNITED STATES |
|  |  |  |  |  |  |
| 1006 | Dr. Joel Charles Silverfield |  | Dr. Michael Claude Burnette  Dr. Harris Hugh McIlwain  Anicette M. Richardson  Dr. Kimberly McIlwain Smith | Tampa Medical Group, PA  Suite 303  4700 North Habana Avenue  Tampa, FL 33614  UNITED STATES | Quorum Institutional Review Board  Suite 1000  1601 Fifth Avenue  Seattle, WA 98101  UNITED STATES |
|  |  |  |  |  |  |
| 1007 | Dr. Sanford Mayer Wolfe |  | Rebecca J. Hanshew | STAT Research, Inc.  West Medical Plaza - Suite 230  One Elizabeth Place  Dayton, OH 45417  UNITED STATES | Quorum Institutional Review Board  Suite 1000  1601 Fifth Avenue  Seattle, WA 98101  UNITED STATES |
|  |  |  |  |  |  |
| 1008 | Dr. Jane Herron Box |  | Dr. John Franklyn Babich  Dr. Patrick N. Box  Dr. Ashrito Kumar Dayal | Arthritis Clinic & Carolina Bone & Joint, PA  10460 Park Road  Charlotte, NC 28210  UNITED STATES | Quorum Institutional Review Board  Suite 1000  1601 Fifth Avenue  Seattle, WA 98101  UNITED STATES |
|  |  |  |  |  |  |
| 1009 | Dr. Shelly Pearl Kafka |  |  | Mountain State Clinical Research  Suite 303A  300 Davisson Run Road  Clarksburg, WV 26301  UNITED STATES  United Hospital Center  X-ray and ECG Only  Suite 107  300 Davisson Run Road  Clarksburg, WV 26301  UNITED STATES | Quorum Institutional Review Board  Suite 1000  1601 Fifth Avenue  Seattle, WA 98101  UNITED STATES |
|  |  |  |  |  |  |
| 1010 | Dr. Alan Jan Kivitz |  | Angela Braatz  Dr. Lori Ann Lavelle  Dr. Frederick Timothy Murphy  Dr. Marianne L. Shaw  Tamara L. Smith  Michael Joseph Zumer | Altoona Center for Clinical Research  175 Meadowbrook Lane  Duncansville, PA 16635  UNITED STATES | Quorum Institutional Review Board  Suite 1000  1601 Fifth Avenue  Seattle, WA 98101  UNITED STATES |
|  |  |  |  |  |  |
| 1011 | Dr. Philip Judson Mease |  | Nicole M. Furfaro  Lyne A. Schaefer-Alfonse  Sue Williams-Judge | Investigational Drug Service  Drug Shipment Only  747 Broadway  Seattle, WA 98122  UNITED STATES  Seattle Rheumatology Associates  Suite 1000  1101 Madison  Seattle, WA 98104  UNITED STATES  Swedish Medical Center  747 Broadway  Seattle, WA 98122  UNITED STATES | Western Institutional Review Board  3535 Seventh Avenue Southwest  Olympia, WA 98502  UNITED STATES |
|  |  |  |  |  |  |
| 1012 | Dr. Atul Kumar Singhal |  | Julia A. Dilliard  Angelia Hannah  Ms. Doris C. Harvey  Dr. Guillermo A. Quiceno  Sandra Rodriguez | Southwest Rheumatology, PA  Suite 615  18601 LBJ Freeway  Mesquite, TX 75150  UNITED STATES | Quorum Institutional Review Board  Suite 1000  1601 Fifth Avenue  Seattle, WA 98101  UNITED STATES |
|  |  |  |  |  |  |
| 1013 | Dr. Jeffrey Louis Kaine |  | Cheryl A. Costa  Dr. Yoel Drucker  Jill R. Garrett | Lovelace Scientific Resources  Suite C  411 Commercial Court  Venice, FL 34292  UNITED STATES  Venice Arthritis Center  Suite D  411 Commercial Court  Venice, FL 34292  UNITED STATES | Quorum Institutional Review Board  Suite 1000  1601 Fifth Avenue  Seattle, WA 98101  UNITED STATES |
|  |  |  |  |  |  |
| 1014 | Dr. Roy Mitchell Fleischmann |  | Jean A. Clark  Dr. Stanley Bruce Cohen  Dr. Thomas David Geppert  Dr. Imran Iqbal  Dr. Robert Neil Jenkins  Dr. Talat Jehan Kheshgi  Dr. Zoran Kurepa  Dr. Sharad Lakhanpal  Andrea S. Martin  Dr. Richard L. Stern  Dayna S. Swan-Flanders  Dr. Jack Bernstein Vine | Metroplex Clinical Research Center  Suite 810  8144 Walnut Hill Lane  Dallas, TX 75231  UNITED STATES | Quorum Institutional Review Board  Suite 1000  1601 Fifth Avenue  Seattle, WA 98101  UNITED STATES |
|  |  |  |  |  |  |
| 1015 | Dr. John Joseph Condemi |  | Terry F. Arnold  Dr. Peter Michael Grace Deane  Dr. Anatole K. Kleiner  Dr. Emmanuel Adolphus Quaidoo | AAIR Research Center  Suite 305  300 Meridian Centre  Rochester, NY 14618  UNITED STATES | Quorum Institutional Review Board  Suite 1000  1601 Fifth Avenue  Seattle, WA 98101  UNITED STATES |
|  |  |  |  |  |  |
| 1016 | Dr. Joel Marc Kremer |  | Christine J. Barr  Dr. Ludovico Frank Cavaliere  Justine S. Feder-Lailer  Dr. Neal Steven Greenstein  Dr. Dorota L. Hausner-Sypek  Jessica L. Johnson  Mari V. Kaymakcian  Justine V. Kehn  Kathleen Kessler  Iris B. Klein  Jessica A. Messemer  Teresa M. Michaels  Dr. Victoria M. Michaels  Rhonda L. Murphy  Dr. Norman Reid Romanoff  Dr. Lee Schulman Shapiro | The Center for Rheumatology, LLP  Suite 101  1367 Washington Avenue  Albany, NY 12206  UNITED STATES | Quorum Institutional Review Board  Suite 1000  1601 Fifth Avenue  Seattle, WA 98101  UNITED STATES |
|  |  |  |  |  |  |
| 1017 | Dr. Steven D. Mathews |  | Dr. Darlene M. Bartilucci  Dr. Alicia D. Campbell  Dr. Ramon B. Castello  Dr. Erin Gautier Doty  Dr. Susan Neims Greco  Dr. Keith R. Holden  Dr. Jeffry Alan Jacqmein  Dr. Michael Jay Koren  Dr. Alpa Patel  Dawn M. Robison  Dr. Neil Sager  Dr. Carolyn M. Tran  Dr. Francis P. Valenzuela | Jacksonville Center for Clinical Research  Suite 1  4085 University Boulevard South  Jacksonville, FL 32216  UNITED STATES | Quorum Institutional Review Board  Suite 1000  1601 Fifth Avenue  Seattle, WA 98101  UNITED STATES |
|  |  |  |  |  |  |
| 1018 | Dr. Michael James Fairfax |  | W. Richard Horn  Courtney G. McDaniel  Dr. Charles S. Mitchell  Julie J. Oppenheim | Arthrocare, Arthritis Care and Research PC  Suite 200  3921 East Baseline Road  Gilbert, AZ 85234  UNITED STATES | Quorum Institutional Review Board  Suite 1000  1601 Fifth Avenue  Seattle, WA 98101  UNITED STATES |
|  |  |  |  |  |  |
| 1024 | Dr. Neil J. Gonter |  | Dr. Ralph E. Marcus  Dr. Shari Schloss | Rheumatology Associates of North Jersey  1415 Queen Anne Road  Teaneck, NJ 07666  UNITED STATES | Quorum Institutional Review Board  Suite 1000  1601 Fifth Avenue  Seattle, WA 98101  UNITED STATES |
|  |  |  |  |  |  |
| 1025 | Dr. Nicholas A. Patrone |  | Jan Adams  Lisa J. Steed | Boice-Willis Clinic, PA  Suite 320  901 North Winstead Avenue  Rocky Mount, NC 27804  UNITED STATES | Quorum Institutional Review Board  Suite 1000  1601 Fifth Avenue  Seattle, WA 98101  UNITED STATES |
|  |  |  |  |  |  |
| 1026 | Dr. Geneva Louise Hill |  | DeEtte M. Burton  Dr. Josette J. Johnson  Dr. Jeffrey Geldert Lawson | Piedmont Arthritis Clinic, PA  Suite 400  3 St. Francis Drive  Greenville, SC 29601  UNITED STATES | Quorum Institutional Review Board  Suite 1000  1601 Fifth Avenue  Seattle, WA 98101  UNITED STATES |
|  |  |  |  |  |  |
| 1027 | Dr. Luis A. Toro |  | Jill R. Garrett | Lovelace Scientific Resources  Suite 560  5741 Bee Ridge Road  Sarasota, FL 34233  UNITED STATES  The Arthritis Specialty Centre  Suite 550  5741 Bee Ridge Road  Sarasota, FL 34233  UNITED STATES | Quorum Institutional Review Board  Suite 1000  1601 Fifth Avenue  Seattle, WA 98101  UNITED STATES |
|  |  |  |  |  |  |
| 1028 | Dr. Richard Roy Olson |  | Dr. David James Dansdill  Tami M. Kucia | Rockford Orthopedic Associates, Ltd.  324 Roxbury Road  Rockford, IL 61107  UNITED STATES | Quorum Institutional Review Board  Suite 1000  1601 Fifth Avenue  Seattle, WA 98101  UNITED STATES |
|  |  |  |  |  |  |
| 1036 | Dr. Nathan Wei |  |  | The Arthritis and Osteoporosis Center of Maryland  71 Thomas Johnson Drive  Frederick, MD 21702  UNITED STATES | Quorum Institutional Review Board  Suite 1000  1601 Fifth Avenue  Seattle, WA 98101  UNITED STATES |
|  |  |  |  |  |  |
| 1037 * | Dr. Douglas Camden Conaway |  | Nancy Eisenberger | Carolina Health Specialists  Suite 4  945 82nd Parkway  Myrtle Beach, SC 29572  UNITED STATES | Quorum Institutional Review Board  Suite 1000  1601 Fifth Avenue  Seattle, WA 98101  UNITED STATES |
|  |  |  |  |  |  |
| 1039 | Dr. Bridget Tyrell Walsh |  | Dr. Laurie Ann Bergstrom  Dr. Michael Joseph Maricic  Dr. Sabina R. Mian  Dr. Deborah Jane Power | Catalina Pointe Clinical Research  Suite 100  7520 North Oracle Road  Tucson, AZ 85704  UNITED STATES | Quorum Institutional Review Board  Suite 1000  1601 Fifth Avenue  Seattle, WA 98101  UNITED STATES |
|  |  |  |  |  |  |
| 1040 | Dr. Melody D. St. John |  | Laura J. Larrison  Dr. James W. Logan  Marilyn J. Morgan | St. Joseph's Mercy Clinic  100 McGowan Court  Hot Springs, AR 71913  UNITED STATES | St. Joseph's Mercy Health Center IRB  100 McAuley Court  Hot Springs, AR 71903  UNITED STATES |
|  |  |  |  |  |  |
| 1041 | Dr. Oscar Soto-Raices |  | Dr. Marelli Colon-Emeric | San Juan Arthritis & Research Center  El Monte Mall - Suite 2010  652 Avenue Munoz Rivera  San Juan, PR 00918  UNITED STATES | Quorum Institutional Review Board  Suite 1000  1601 Fifth Avenue  Seattle, WA 98101  UNITED STATES |
|  |  |  |  |  |  |
| 1052 | Dr. John Joseph Cush |  | Leilani D. Law | Baylor Research Institute  Arthritis Care and Research Center  Suite 550  9900 North Central Expressway  Dallas, TX 75231  UNITED STATES | Baylor Research Institute Institutional Review Board  White/Blue/Red  Suite 501  3310 Live Oak  Dallas, TX 75204  UNITED STATES |
|  |  |  |  |  |  |
| 1071 | Dr. Erdal Diri |  | Jerane A. Forsberg  Kristen A. Schoen | Trinity Health Center - Medical Arts  400 Burdick Expressway East  Minot, ND 58701  UNITED STATES | Trinity IRB  Institutional Review Board  One Burdick Expressway West  Minot, ND 58701  UNITED STATES |
|  |  |  |  |  |  |
| 1076 | Dr. Haydon Anthony Moorman  Dr. James D. Taborn (Previous PI) |  | Dr. Andrew Daugavietis  Deborah Hotchkiss  Lynn M. Kiewiet  Loria L. Ramos | Borgess Research Institute  Suites 003 and 004  1717 Shaffer Street  Kalamazoo, MI 49048  UNITED STATES  Borgess Rheumatology  Suite 124  1717 Shaffer Street  Kalamazoo, MI 49048  UNITED STATES | Quorum Institutional Review Board  Suite 1000  1601 Fifth Avenue  Seattle, WA 98101  UNITED STATES |
|  |  |  |  |  |  |
| 1077 * | Dr. Thomas Willard Littlejohn III |  | Dr. David Dutrow Collins  Dr. Robert John Holmes  Dr. Patricia Mills Klein  Dr. Steven Russell Klein  Kathy Chilton Simpson  Dr. Herbert Alva Soper  Dr. David Lee Spivey  Dr. Louis Rocco Valente II  Dr. Jonathan Paul Wilson  Dr. Steven Philip Wittmer | Brookview Hills Internal Medicine  Suite 207  3333 Brookview Hills Boulevard  Winston-Salem, NC 27103  UNITED STATES  Piedmont Medical Research Associates  Suite 306  1901 South Hawthorne Road  Winston-Salem, NC 27103  UNITED STATES  Preferred Pain Management  Suite C  245 Charlois Boulevard  Winston-Salem, NC 27103  UNITED STATES | Quorum Institutional Review Board  Suite 1000  1601 Fifth Avenue  Seattle, WA 98101  UNITED STATES |
|  |  |  |  |  |  |
| 1078 | Dr. James Craig VanDeWall |  | Dr. Ross A. Horsley  Ms. Josephine Raab  Dr. Victor R. Rodriguez | Southern Tier Arthritis and Rheumatism  415 North 8th Street  Olean, NY 14760  UNITED STATES | Quorum Institutional Review Board  Suite 1000  1601 Fifth Avenue  Seattle, WA 98101  UNITED STATES |
|  |  |  |  |  |  |
