# Supplementary material for: Changes in serum creatinine in patients with active rheumatoid arthritis treated with tofacitinib: results from clinical trials
Source: Arthritis Res Ther. 2014 Jul 25;16(4):R158. doi: 10.1186/ar4673 (PMC4220634; doi:10.1186/ar4673)
Supplement: Supplementary file 11 — Additional file 11: List of Investigators and Corresponding Ethics Committees or Institutional Review Boards for the Phase 3 A3921046 study. (DOC 362 KB) [file 13075_2013_4378_MOESM11_ESM.doc]

# 16.1.4 LIST OF INVESTIGATORS AND CORRESPONDING ETHICS COMMITTEES OR INSTITUTIONAL REVIEW BOARDS

## Australia

**Coordinating Investigators:**

<None Entered>

| **Center** | **Principal Investigator** | **Co-Investigator(s)** | **Sub-Investigator(s)** | **Address(es)** | **Institutional Review Board or Ethics Committee Address(es)** |
| --- | --- | --- | --- | --- | --- |
|  |  |  |  |  |  |
| 1065 | Dr. David H. Bossingham |  | Karen Cooke  Dr. Joshua P. Hanson  Kathryn Leonard | Rheumatology Research  5th Foor Block B Cairns Base Hospital  The Esplanade  Cairns, QLD 4870  AUSTRALIA | Cairns and Hinterland Health Service District Human Research Ethics Committee  4th Floor, Block A  Cairns Base Hospital  Cairns, QLD 4870  AUSTRALIA |
|  |  |  |  |  |  |
| 1066 | Dr. Nicola Cook  Madelynn Chan (Previous PI) |  | Ms. Maureen Blore  Dr. Elissa Campbell  Madelynn Chan  Priya Chowalloor  Peter Dias  Dr Andreea Harsanyi  Dr. Yasaman Khayyam  Ms. Margaret Rogers  Dr. Andrew Taylor | The Goatcher Clinical Research Unit  Royal Perth Hospital Shenton Park Campus  6 Selby Street  Shenton Park, WA 6008  AUSTRALIA | Royal Perth Hospital Ethics Committee, Royal Perth Hospital  Wellington Street Campus  Wellington Street  Perth, WA 6000  AUSTRALIA |
|  |  |  |  |  |  |
| 1067 | Assoc. Prof. Stephen Hall |  | Dr. Vivienne Beckett  Dr Jill Bell  Dr Malcolm Clark  Dr Jennifer Davey  Ms Stephanie Dolejs  Dr. Marie Feletar  Dr. Andrew Gibson  Gail Grant  Dr. James McDonald  Dr Nicole McKay  Dr Louise Murdoch | Emeritus Research  291 Wattletree Road  Malvern East, VIC 3145  AUSTRALIA | Cabrini Human Research Ethics Committee  183 Wattletree Rd,  Malvern, VIC , Australia 3144  AUSTRALIA |
|  |  |  |  |  |  |
| 1068 | Dr. David Nicholls (Previous PI)  Prof. Peter T. Nash  Prof. Peter T. Nash (Previous PI) |  | Dr. Frances Johnson  Dr. David Nicholls  Ms. Dale Shergold  Ms. Jan Smith  Dr. Susan Thackwray  Dr. Avgeania Voight | Rheumatology Research Unit Sunshine Coast  9-10 Maroochy Waters Shopping Centre  Denna Street  Maroochydore, QLD 4558  AUSTRALIA | Redcliffe-Caboolture Ethics Committee  Unit 1, Ground Floor, Redcliffe Hospital  Anzac Avenue  Redcliffe, Qld 4020  AUSTRALIA |
|  |  |  |  |  |  |
| 1069 | Dr. Maureen Rischmueller |  | Rachel Black  Dr. Simon Burnet  Dr. Sarah Downie-Doyle  Dr. Catherine Hill  Dr. Veera Katikireddi  Dr. Jem Ninan  Dr. Samuel Whittle | The Queen Elizabeth Hospital, Department of Rheumatology  28 Woodville Road  Woodville, SA 5011  AUSTRALIA | Central Northern Adelaide Health Service  Ethics of Human Research Committee  The Queen Elizabeth Hospital  28 Woodville Road  Woodville, SA 5011  AUSTRALIA |
|  |  |  |  |  |  |
| 1131 * | Dr. Mona Marabani |  | Dr. Melanie Wroth | Emeritus Research  33 South Parade  Campsie, NSW 2194  AUSTRALIA | Cabrini Human Research Ethics Committee  183 Wattletree Road  Malvern, VIC 3144  AUSTRALIA |
|  |  |  |  |  |  |

## Chile

**Coordinating Investigators:**

<None Entered>

| **Center** | **Principal Investigator** | **Co-Investigator(s)** | **Sub-Investigator(s)** | **Address(es)** | **Institutional Review Board or Ethics Committee Address(es)** |
| --- | --- | --- | --- | --- | --- |
|  |  |  |  |  |  |
| 1001 | Dr. Marta Aliste |  | Dr. Julio Cruz  Dr. Hector Gatica  Dr. Sandra Pino  Dr. Francisca Sabugo  Dr. Lilian Soto  Dr. Pamela Wurmann | Consulta Privada Dra. Marta Aliste  Guardia Vieja 255, Oficina 1409  Providencia  Santiago, RM 7510186  CHILE | Comite Etico Cientifico  Servicio de Salud Metropolitano Oriente  Avenida Salvador 364  Providencia, Santiago, RM 7500922  CHILE |
|  |  |  |  |  |  |
| 1002 | Dr. Francisco Ballesteros |  | Dr. Maria Eugenia Alvarez  Dr. Loreto Ovalle  Dr. Paula Pozo | Centro de Diagnostico y Tratamiento San Borja Arriaran  Seccion Reumatologia  Amazonas 619  Santiago, RM 8360156  CHILE | Comite Etico Cientifico  Servicio de Salud Metropolitano Oriente  Avenida Salvador 364  Providencia, Santiago, RM 7500922  CHILE |
|  |  |  |  |  |  |
| 1003 | Dr. Gloria Holuigue |  | Juan Manuel Contreras  Luis Orellana | Clínica Santa María, Sección Reumatología  Fernando Manterola 0540  Providencia, Santiago, RM 7530206  CHILE | Comite Etico Cientifico  Servicio de Salud Metropolitano Oriente  Avenida Salvador 364  Providencia, Santiago, RM 7500922  CHILE |
|  |  |  |  |  |  |
| 1004 | Dr. Renato Jimenez Calabresse |  | Dr. Cecilia Georgi  Dr. Ricardo Jerez  Dr. Luis Roca | Estudios Clinicos V Region  Alvarez 1806  Vina del Mar, V Region 2570017  CHILE | Comite Etico Cientifico del Servicio de Salud Vina-Quillota  Calle Limache 1307  2° Piso  Vina del Mar, V Region  CHILE |
|  |  |  |  |  |  |

## China

**Coordinating Investigators:**

<None Entered>

| **Center** | **Principal Investigator** | **Co-Investigator(s)** | **Sub-Investigator(s)** | **Address(es)** | **Institutional Review Board or Ethics Committee Address(es)** |
| --- | --- | --- | --- | --- | --- |
|  |  |  |  |  |  |
| 1101 | Dr. ZhanGuo LI |  | Yuan An  Dr. Benliang Ma  Sisi Pan | Peking University People's Hospital/Rheumatology and Immunology Department  No.11, Xizhimen South Street  Xicheng District  Beijing, 100044  CHINA | Ethics Committee of Peking University People's Hospital  No.11, Xizhimen South Street  Xicheng District  Beijing, 100044  CHINA |
|  |  |  |  |  |  |
| 1102 | Dr. Feng Huang |  | Hua Ma  Hong ZHANG  Jianglin ZHANG  Zheng Zhao | PLA General Hospital  28 Fu Xing Road HaiDian Qu  Beijing, 100853  CHINA | Ethics Committee of Peking University People's Hospital  No.11, Xizhimen South Street  Xicheng District  Beijing, 100044  CHINA |
|  |  |  |  |  |  |
| 1103 | Yi ZHENG |  | Xin DONG  Haiyun Li  Jian LI  Yongfeng Zhang | Beijing Chaoyang Hospital, Capital University of Medical Science  No. 8 Baijiazhuang  Chao Yang District  Beijing, 100020  CHINA | Ethics Committee of Beijing Chaoyang Hospital, Capital University of Medical Science  No. 8 Baijiazhuang  Chao Yang District  Beijing, 100020  CHINA |
|  |  |  |  |  |  |
| 1104 | Dr. Chunde Bao |  | Qing Dai  Dr. Hui Du  Dr. Qiang Guo | Rheumatology and Immunology Dept., Renji Hospital Shanghai Jiao Tong University School of Medicine  NO. 145 Middle Shandong Road  Shanghai, 200001  CHINA | Ethics Committee of Renji Hospital Shanghai Jiao Tong University School of Medicine  Independent Ethics Committee  No. 1630 Dongfang Road  Shanghai, 200127  CHINA |
|  |  |  |  |  |  |
| 1105 | Dongbao Zhao |  | Lianmei Ji  Dr. Yeqing Shi  Deshao YE | Rheumatology and Immunology Department, Shanghai Changhai Hospital  No. 168, Changhai Road  Shanghai, 200433  CHINA | Ethics Committee of Peking University People's Hospital  No.11, Xizhimen South Street  Xicheng District  Beijing, 100044  CHINA |
|  |  |  |  |  |  |
| 1106 | Huji Xu |  | Dr. Jun Bao  Dr. Ting LI  Jing ZHAO | Rheumatology and Immunology Department, Shanghai Changzheng Hospital  No. 415, Fengyang Road  Shanghai, 200003  CHINA | Ethics Committee of Peking University People's Hospital  No.11, Xizhimen South Street  Xicheng District  Beijing, 100044  CHINA |
|  |  |  |  |  |  |
| 1107 | Dr. JieRuo GU |  | Jianlin Huang  Ou Jin  Tianwang Li  Qiuxia Li  Qiguang Li  Zetao Liao  Yunfeng Pan  Yuqiong Wu | The Third Affiliated Hospital Of Sun Yat-sen University  Shipaigangding  Tianhe District  Guangzhou, Guangdong 510630  CHINA | EC of The Third Affiliated Hospital Of Sun Yat-sen University  The Third Affiliated Hospital Of Sun Yat-sen University  Shipaigangding  Tianhe District  Guangzhou, Guangdong 510630  CHINA |
|  |  |  |  |  |  |
| 1108 | Yi Tao |  | Ruilin Chen  Wenhui Huang  Chenghui Huang  Zelun Li  Zeying Lin  Ziguan Ye | The Second Affiliated Hospital of Guangzhou Medical College  No. 250, Changgang East Road, Haizhu District  Guangzhou, Guangdong 510260  CHINA | EC of The Second Affiliated Hospital of Guangzhou Medical College  No. 250, Changgang East Road, Haizhu District  Guangzhou, Guangdong 510260  CHINA |
|  |  |  |  |  |  |
| 1110 | Dr. Lu GONG |  | Wenwen Sun  Wei Wei  Na Zhang | Department of Infectious Diseases & Immunology, Tianjin Medical University General Hospital  No. 154, Anshan Road, Heping District  Tianjin, 300052  CHINA | Ethics Committee of Peking University People's Hospital  No.11, Xizhimen South Street  Xicheng District  Beijing, 100044  CHINA |
|  |  |  |  |  |  |
| 1111 | Xingfu Li |  | Huaxiang Liu  Feng Qiu  Xiao WANG | QiLu Hospital of Shandong University  107 Western Culture Road  Jinan, Shandong 250012  CHINA | Ethics Committee of QiLu Hospital of Shandong University  107 Western Culture Road  Jinan, Shandong 250012  CHINA |
|  |  |  |  |  |  |
| 1112 | Houheng Su |  | Guangwen LI  Hong MA  Shiping QU  Qian Xing | Qingdao Municipal Hospital  No. 1, Jiaozhou Road  Qingdao, Shandong 266011  CHINA | Ethics Committee of Peking University People's Hospital  No.11, Xizhimen South Street  Xicheng District  Beijing, 100044  CHINA |
|  |  |  |  |  |  |
| 1113 | Ping Zhu |  | Lina Chen  Nan Leng  Lu Wang  Ronghua Xie | Xijing Hospital, The Fourth Military Medical University  No. 15 Changlexi Road  Xi'an, Shanxi 710032  CHINA | IEC of Xijing Hospital, The Fourth Military Medical University  No. 15 Changlexi Road  Xi'an, Shanxi 710032  CHINA |
|  |  |  |  |  |  |
| 1114 | Dr. JianHua XU |  | Shanyu Chen  Li Lian  Shuang Liu  Zongwen Shuai  Fen Wang  Hui Xiao  Shengqian Xu | An Hui Medical University 1st Hospital/Rheumatology Department  No.218, Jixi Road  Hefei, Anhui 230022  CHINA | Ethics Committee of An Hui Medical University 1st Hospital  No.218, Jixi Road  Hefei, Anhui 230022  CHINA |
|  |  |  |  |  |  |
| 1115 | Dr. XiangPei LI |  | Xiaomei Li  Yan Ma  Min Zhang | Anhui Province Hospital/Rheumatology Department  No.4, Lujiang Road  Zhongshi District  Hefei, Anhui 230001  CHINA | Ethics Committee of Peking University People's Hospital  No.11, Xizhimen South Street  Xicheng District  Beijing, 100044  CHINA |
|  |  |  |  |  |  |
| 1116 | Huaxiang Wu |  | Wenjia Sun  Lihong Wen | The Second Affiliated Hospital of Zhejiang University School of Medicine  No. 80, Jiefang Road  Hangzhou, Zhejiang 310009  CHINA | Ethics Committee of Peking University People's Hospital  No.11, Xizhimen South Street  Xicheng District  Beijing, 100044  CHINA |
|  |  |  |  |  |  |
| 1117 | Miaojia Zhang |  | Yu Ding  Yanyan Wang  Guiqin Yuan | Jiangsu Province Hospital  No. 300, Guangzhou Road  Nanjing, Jiangsu 210029  CHINA | IEC of Jiangsu Province Hospital  No.300 Guangzhou Road  Gu Lou District  Nanjing, 210029  CHINA |
|  |  |  |  |  |  |
| 1118 | Zhiwei Chen |  | Yingsu Deng  Jian Wu  Keqin Zeng | the First Affiliated Hospital of Soochow University  Department of Rheumatology  No.188 Shizi street  suzhou, jiangsu 215006  CHINA | EC of the First Affiliated Hospital of Soochow University  No.188 Shizi Street,  Suzhou, Jiangsu 215006  CHINA |
|  |  |  |  |  |  |
| 1119 | Shaoxian Hu |  | Dr. PeiGen He  Xiaomei Lei  Wei Tu  Fei Yu  Yikai Yu | Tongji Hospital, Tongji Medical College, Huazhong University of Science and Technology  No.1095, Jiefang Road  Wuhan, Hubei 430030  CHINA | Ethics Committee of Peking University People's Hospital  No.11, Xizhimen South Street  Xicheng District  Beijing, 100044  CHINA |
|  |  |  |  |  |  |
| 1120 | Xiaoxia Zuo |  | Yisha Li  Sijia Liu  Hui Luo  Yanping Wang  Yanli Xie  Hongjun Zhao | Xiangya Hospital of Centre-south University  No. 87 Xiangya Road  Changsha, Hunan 410008  CHINA | Ethics Committee of Peking University People's Hospital  No.11, Xizhimen South Street  Xicheng District  Beijing, 100044  CHINA |
|  |  |  |  |  |  |
| 1121 | Yi Liu |  | Hui Lin  Chunyu Tan | Si Chuan Huaxi Hospital/Rheumatology Department  No.37, Wainanguoxuexiang  Chengdu, Sichuan 610041  CHINA | Ethics Committee of Peking University People's Hospital  No.11, Xizhimen South Street  Xicheng District  Beijing, 100044  CHINA |
|  |  |  |  |  |  |

## Colombia

**Coordinating Investigators:**

<None Entered>

| **Center** | **Principal Investigator** | **Co-Investigator(s)** | **Sub-Investigator(s)** | **Address(es)** | **Institutional Review Board or Ethics Committee Address(es)** |
| --- | --- | --- | --- | --- | --- |
|  |  |  |  |  |  |
| 1147 | Dr. William Jose Otero Escalante MD |  | Dr. Marcial Martinez  Dr. Gerardo Ramirez MD  Hernan Roberto Vera Quinche | SERVIMED E.U  Calle 51# 34-17 Consultorio 208-208A Centro Comercial Cabecera. Etapa I  Bucaramanga, Santander  COLOMBIA | Comite de ética en Investigación de Servimed E.U  Calle 51 No. 34-17 Consultorio 208-208A Centro comercial  Cabecera etapa I  Bucaramanga, Santander 0000  COLOMBIA |
|  |  |  |  |  |  |
| 1148 | Dr. Edwin Antonio Jauregui MD (Previous PI)  Dr. Maria Concepcion Maldonado |  | Dr. Maria Claudia Diaz  Dr. Aura Maria Dominguez  Dr. Edwin Antonio Jauregui MD  Dr. Jhon Jairo Medina  Dr. Yenny Soraida Valero | Riesgo de Fractura S.A.  Carrera 12 # 98-38  Bogota, Cundinamarca  COLOMBIA | Comite de etica de la investigación-Riesgo de Fractura S.A  Cr. 12 No.98-38  Bogota, Cundinarca  COLOMBIA |
|  |  |  |  |  |  |
| 1183 | Dr. Juan J. Jaller Raad |  | Dr. Javier Cuartas  Dr. Anubys Maiguel  Dr. Victor Andres Ulloque Lopez | Centro de Reumatologia y Ortopedia  Cra 49 C No 82-120  Barranquilla, Atlantico 0000  COLOMBIA | Comité de etica independiente centro de reumatologia y ortopedia  Cr. 49C No. 82-120  Barranquilla, Atlantico 0000  COLOMBIA |
|  |  |  |  |  |  |

## Croatia

**Coordinating Investigators:**

<None Entered>

| **Center** | **Principal Investigator** | **Co-Investigator(s)** | **Sub-Investigator(s)** | **Address(es)** | **Institutional Review Board or Ethics Committee Address(es)** |
| --- | --- | --- | --- | --- | --- |
|  |  |  |  |  |  |
| 1152 | Dr. Ksenija Mastrovic Radoncic |  | Dr. Marinko Artukovic  Dr. Maja Paar Puhovski | Institute for physical medicine, rehabilitation and rheumatology  General Hospital "Sveti Duh"  Sveti Duh 64  Zagreb, 10000  CROATIA | Central Ethics Committee  Agency for Medicinal Products and Medical Devices  Ksaverska c. 4  Zagreb, 10000  CROATIA |
|  |  |  |  |  |  |
| 1153 * | Dr. Tatjana Kehler |  | Dr. Anita Legovic  dr Drazen Massari | Thalassotherapia Opatija  Marsala Tita 188/1  Opatija, 51410  CROATIA | Central Ethics Committee  Agency for Medicinal Products and Medical Devices  Ksaverska c. 4  Zagreb, 10000  CROATIA |
|  |  |  |  |  |  |

## Denmark

**Coordinating Investigators:**

<None Entered>

| **Center** | **Principal Investigator** | **Co-Investigator(s)** | **Sub-Investigator(s)** | **Address(es)** | **Institutional Review Board or Ethics Committee Address(es)** |
| --- | --- | --- | --- | --- | --- |
|  |  |  |  |  |  |
| 1081 | Dr. Bente Danneskiold-Samsoe |  | Dr. Henning Bliddal  Dr. Henrik Gudbergsen  Dr. Anja Falk Riecke  Dr. Birgit Falk Riecke | H:S Frederiksberg Hospital  Parker Instituttet  Ndr. Fasanvej 57-59  Frederiksberg, 2000  DENMARK | De Videnskabsetiske Komitéer for Region Hovedstaden  Regionsgaarden  Kongens Vaenge 2  Hilleroed, 3400  DENMARK |
|  |  |  |  |  |  |

## Finland

**Coordinating Investigators:**

<None Entered>

| **Center** | **Principal Investigator** | **Co-Investigator(s)** | **Sub-Investigator(s)** | **Address(es)** | **Institutional Review Board or Ethics Committee Address(es)** |
| --- | --- | --- | --- | --- | --- |
|  |  |  |  |  |  |
| 1013 | Kari Eklund (Previous PI)  Leena Paimela |  | Marja-Terttu Brandt  Kari Eklund  Riitta Koivuniemi | Helsingin Reumakeskus Oy  Bulevardi 22 A  Helsinki, 00120  FINLAND | Helsingin ja Uudenmaan sairaanhoitopiiri  Sisatautien eettinen toimikunta  Biomedicum Helsinki 2 C  PL 705  HUS, 00029  FINLAND |
|  |  |  |  |  |  |
| 1014 | Pentti Jarvinen |  | Timo Ahtikari  Sirkka Koskinen | Kiljavan Laaketutkimus Oy  Donnerinkatu 5  Hyvinkaa, 05800  FINLAND | Helsingin ja Uudenmaan sairaanhoitopiiri  Sisatautien eettinen toimikunta  Biomedicum Helsinki 2 C  PL 705  HUS, 00029  FINLAND |
|  |  |  |  |  |  |
| 1080 | Pia Isomaki |  | Krista Karstila  Vappu Rantalaiho  Susanna Sihvonen | Tampereen yliopistollinen sairaala/Reumakeskus/Reumatologian poliklinikka  Teiskontie 35  Tampere, 33520  FINLAND | Helsingin ja Uudenmaan sairaanhoitopiiri  Sisatautien eettinen toimikunta  Biomedicum Helsinki 2 C  PL 705  HUS, 00029  FINLAND |
|  |  |  |  |  |  |

## Germany

**Coordinating Investigators:**

<None Entered>

| **Center** | **Principal Investigator** | **Co-Investigator(s)** | **Sub-Investigator(s)** | **Address(es)** | **Institutional Review Board or Ethics Committee Address(es)** |
| --- | --- | --- | --- | --- | --- |
|  |  |  |  |  |  |
| 1085 | Dr. med. Rieke Alten |  | Dr. med. Stefan Bieneck  Annalina Braun  Svitlana Djacenko  Dr. med. Christoph Pohl  Olaf Schroeder | Schlosspark-Klinik, Innere Medizin II, Rheumatologie  Heubnerweg 2  Berlin, 14059  GERMANY | Ethikkommission der Saechsischen Landesaerztekammer  Schuetzenhoehe 16  Dresden, 01099  GERMANY |
|  |  |  |  |  |  |
| 1086 | Prof. Dr. med. Christoph Baerwald (Previous PI)  Prof. Dr. med. Ulf Wagner |  | Dr. Sybille Arnold  Prof. Dr. med. Christoph Baerwald  Dr. Olga Malysheva  Dr. Matthias Pierer  Dr. Susette Unger | Universitaetsklinikum Leipzig AoeR, Department fuer Innere Medizin  Sektion Rheumatologie/Gerontologie, Studienambulanz Rheumatologie  Liebigstr. 20  Leipzig, 04103  GERMANY | Ethikkommission der Saechsischen Landesaerztekammer  Schuetzenhoehe 16  Dresden, 01099  GERMANY |
|  |  |  |  |  |  |
| 1088 | Prof. Dr. med. Hubert Nuesslein |  | Daniela Birsch | Arztpraxis, Internist - Rheumatologie  Kontumazgarten 4  Nuernberg, 90429  GERMANY | Ethikkommission der Saechsischen Landesaerztekammer  Schuetzenhoehe 16  Dresden, 01099  GERMANY |
|  |  |  |  |  |  |
| 1089 | Dr. Ulrich Schoo |  | Dr. Georg Huebner | Schwerpunktpraxis fuer Rheumatologie  Sprickmannstr. 36  Rheine, 48431  GERMANY | Ethikkommission der Saechsischen Landesaerztekammer  Schuetzenhoehe 16  Dresden, 01099  GERMANY |
|  |  |  |  |  |  |
| 1090 | Prof. Dr. med. Juergen Wollenhaupt |  | Dr. med. Andrea Everding  Dr. med. Ulrike Schnoor  Dr. med. Wolfgang Winter | Schoen Klinik Hamburg - Eilbek, Abt. fuer Rheumatologie  Dehnhaide 120  Hamburg, 22081  GERMANY | Ethikkommission der Saechsischen Landesaerztekammer  Schuetzenhoehe 16  Dresden, 01099  GERMANY |
|  |  |  |  |  |  |
| 1095 | Dr. Leonore Unger |  | Dr.med. Markus Enderlein  Dr. med. Franziska Herrmann  Dr.med. Marten Kayser  Dr. med. Susanne Neumann  Dr.med. Eva-Maria Wagner | Krankenhaus Friedrichstadt, I. Med. Klinik  Friedrichstrasse 41  Dresden, 01067  GERMANY | Ethikkommission der Saechsischen Landesaerztekammer  Schuetzenhoehe 16  Dresden, 01099  GERMANY |
|  |  |  |  |  |  |

## Greece

**Coordinating Investigators:**

<None Entered>

| **Center** | **Principal Investigator** | **Co-Investigator(s)** | **Sub-Investigator(s)** | **Address(es)** | **Institutional Review Board or Ethics Committee Address(es)** |
| --- | --- | --- | --- | --- | --- |
|  |  |  |  |  |  |
| 1047 * | Dr. Kyriaki Boki |  | Dr. Korina Lymperopoulou  Dr. Lamprini Pantazi  Dr. Vasiliki Tzavara | Sismanoglio Hospital, Rheumatology Department  1 Sismanogliou Street  Maroussi Athens, 15126  GREECE | National Ethics Committee  284 Mesogion Avenue  Athens, 15562  GREECE |
|  |  |  |  |  |  |

## Malaysia

**Coordinating Investigators:**

<None Entered>

| **Center** | **Principal Investigator** | **Co-Investigator(s)** | **Sub-Investigator(s)** | **Address(es)** | **Institutional Review Board or Ethics Committee Address(es)** |
| --- | --- | --- | --- | --- | --- |
|  |  |  |  |  |  |
| 1005 | Dr Heselynn Hussein |  | Dr. Fazirah Abdullah  Dr Eashwary Mageswaren  Dr. Liza Mohd Isa  Dr. Shamala Rajalingam | Hospital Putrajaya  Federal Government Administration Centre  Presint 7  Putrajaya, Wilayah Persekutuan 62250  MALAYSIA | Medical Research & Ethics Committee  Ministry of Health, c/o NIH Secretariat, Institute for Health Management  Jalan Rumah Sakit  Bangsar  Kuala Lumpur, 59000  MALAYSIA |
|  |  |  |  |  |  |
| 1008 | Dr. Suk Chyn Gun |  | Dr. Beryl Agnes D'souza  Dr. Bernard Prakash Devadasan  Dr. C.Gandhi K.Chembalingam Pillay  Dr. Ai Lee Lim  Dr. Asmah Mohd  Dr. Liza Mohd Isa  Dr. Nadiah Mohd Noor | Hospital Tuanku Ja'afar  Jalan Rasah  Seremban, Negeri Sembilan 70300  MALAYSIA | Medical Research & Ethics Committee  Ministry of Health, c/o NIH Secretariat, Institute for Health Management  Jalan Rumah Sakit  Bangsar  Kuala Lumpur, 59000  MALAYSIA |
|  |  |  |  |  |  |
| 1043 | Dr Swan Sim Yeap |  | Dr. Eng Seng Ng  Dr. Raveendran Ramachandran | Sime Darby Medical Centre Subang Jaya Sdn Bhd  No.1, Jalan SS12/1A  Subang Jaya, Selangor 47500  MALAYSIA | Independent Ethics Committee  Sime Darby Medical Centre Subang Jaya Sdn Bhd  No. 1, Jalan SS12/1A  Subang Jaya, Selangor 47500  MALAYSIA |
|  |  |  |  |  |  |
| 1046 | Dr. Azmillah Rosman |  | Dr. Hilmi Abdullah  Dr. Ramani Arumugam  Dr. Shereen Suyin Ch'ng  Dr. Hwee Cheng Chong  Dr. Asmahan Mohamed Ismail  Dr. Woon Pang Kuan  Dr. Ing Soo Lau  Dr. Habiba Mohd Yusoof  Dr. Mollyza Mohd Zain  Dr. Yew Chong Ong | Hospital Selayang  Lebuhraya Selayang-Kepong  Batu Caves, Selangor 68100  MALAYSIA | Medical Research & Ethics Committee  Ministry of Health, c/o NIH Secretariat, Institute for Health Management  Jalan Rumah Sakit  Bangsar  Kuala Lumpur, 59000  MALAYSIA |
|  |  |  |  |  |  |

## Mexico

**Coordinating Investigators:**

<None Entered>

| **Center** | **Principal Investigator** | **Co-Investigator(s)** | **Sub-Investigator(s)** | **Address(es)** | **Institutional Review Board or Ethics Committee Address(es)** |
| --- | --- | --- | --- | --- | --- |
|  |  |  |  |  |  |
| 1151 | Dr. Daniel Xavier Xibille-Friedmann |  | Sara Eugenia Hernandez-Gongora  Jaime Hector Ocampo-Velez | INOVAMED Hospital  Cuauhtemoc 203-109  Colonia Lomas de la Selva  Cuernavaca, Morelos 62270  MEXICO | Comité de Ética del Hospital INOVAMED  Cuauhtemoc 305  Colonia Lomas de la Selva  Cuernavaca, Morelos 62270  MEXICO |
|  |  |  |  |  |  |
| 1155 | Dr. Roman Cardona-Cabrera |  | Maria Isabel Segura-Esquivel | Hospital Angeles de Queretaro  Consultorio 120 B  Bernardino Del Razo 21  Colonia Ensueno  Mexico, Queretaro 76178  MEXICO | Comite Bioetico para la Investigacion Clinica S.C.  Puebla 422  Despacho 4  Col. Roma Sur  MEXICO, DISTRITO FEDERAL 06700  MEXICO |
|  |  |  |  |  |  |
| 1157 | Dr. Rafael Horacio Cornejo-Ballesteros |  | Dr. Marco Antonio Escobedo-Madrigal  Benigno Figueroa-Nunez  Rafael Garcia-Lopez  Noe Pina-Estrada  Jorge Villalpando-Espinoza | Clinica de Enfermedades Cronicas y Procedimientos Especiales SC  Fray Bernardino de Sahagun 101  Fraccionamiento Mirador de Punhuato  Morelia, Michoacan 58249  MEXICO | Comision de Etica e Investigacion CECYPE  Fray Bernardino de Sahagun 101  Fracc. Mirador del Punhuato  Morelia, Michoacan 58249  MEXICO |
|  |  |  |  |  |  |
| 1158 | Dr. Jose Arturo Covarrubias-Cobos |  | Dr. Francisco Avila-Zapata  Hugo Israel Segovia-Escalante | Centro Medico de las Americas  Consultorio 115  Calle 54 365 x 33 A Avenida Perez Ponce, Centro  Merida, Yucatan 97000  MEXICO | Comite de Etica del Centro Medico de las Americas  Calle 54 365 x 33A  Avenida Perez Ponce  Merida, Yucatan 97000  MEXICO |
|  |  |  |  |  |  |
| 1159 | Dra. Isaura Maria Rodriguez-Torres |  | Carlos De la Cueva-Rodriguez  Sergio Jesus Madinabeitia-Martinez  Dr. Myrna Isset Salas-Estrada | Unidad de Enfermedades Reumaticas y Cronico Degenerativas SC  Matamoros 798 West Torreon  Torreon, Coahuila 27000  MEXICO | Comite de Bioetica de la Facultad de Medicina UA de C  Morelos 900 Oriente  Colonia Centro  Torreon, Coahuila 27000  MEXICO |
|  |  |  |  |  |  |

## Poland

**Coordinating Investigators:**

<None Entered>

| **Center** | **Principal Investigator** | **Co-Investigator(s)** | **Sub-Investigator(s)** | **Address(es)** | **Institutional Review Board or Ethics Committee Address(es)** |
| --- | --- | --- | --- | --- | --- |
|  |  |  |  |  |  |
| 1071 | Dr. Stefan Daniluk |  | Prof. Janusz Badurski  Dr. Anna Jarmoc  Dr. Elzbieta Zofia Jeziernicka  Dr. Nonna Nowak | Niepubliczny Zaklad Opieki Zdrowotnej  Centrum Osteoporozy i Chorob Kostno-Stawowych J. Badurski Sp. J.  ul. Warynskiego 6/2  Bialystok, 15-461  POLAND | Komisja Bioetyczna przy Okregowej Izbie Lekarskiej  ul. Swietojanska 7  Bialystok, 15-082  POLAND |
|  |  |  |  |  |  |
| 1072 | Dr. Artur Racewicz |  | Dr. Malgorzata Fiedorczyk  Dr. Dorota Golaszewska  Dr. Krystyna Kuc  Dr. Sylwia Izabela Raczynska  Dr. Jerzy Supronik | NZOZ CENTRUM MEDYCZNE  ul. Pulaskiego 69  Bialystok, 15-337  POLAND | Komisja Bioetyczna przy Okregowej Izbie Lekarskiej  ul. Swietojanska 7  Bialystok, 15-082  POLAND |
|  |  |  |  |  |  |
| 1156 | Prof. Pawel Hrycaj |  | Dr. Katarzyna Cebrowska  Dr. Lidia Fornalska  Dr. Elzbieta Gigiel  Dr. Lukasz Hordecki  Dr. Kajetan Kisiel  Ewa Kloskowska  Dr. Michal Moskal  Dr. Anna Olewicz-Gawlik  Dr. Dorota Zapolska-Pytlik | Oddzial Reumatologiczny, Szpital im. Teodora Dunina  Samodzielny Publiczny Zespol Opieki Zdrowotnej w Koscianie  Ul. Szpitalna 7  Koscian, 64-000  POLAND | Komisja Bioetyczna przy Okregowej Izbie Lekarskiej  ul. Swietojanska 7  Bialystok, 15-082  POLAND |
|  |  |  |  |  |  |
| 1162 | Dr. Wieslawa Porawska |  | Dr. Kamilla Klama  Dr. Wlodzimierz Piotrowski | Poznanski Osrodek Medyczny  'Novamed'  Ul. Sniadeckich 7/2  Poznan, 60-773  POLAND | Komisja Bioetyczna przy Okregowej Izbie Lekarskiej  ul. Swietojanska 7  Bialystok, 15-082  POLAND |
|  |  |  |  |  |  |
| 1163 | Slawomir Jeka |  | Dr. Jolanta Augustynowicz-Koziell  Dr. Radoslaw Brukiewa  Dr. Dominik Chraniuk  Dr. Aneta Galan  Dorota Wisniewska  Rafal Wojciechowski | NZOZ "NASZ LEKARZ"  Praktyka Grupowa Lekarzy Rodzinnych z Przychodnia Specjalistyczna  Szczytna 20  Torun, 87-100  POLAND | Komisja Bioetyczna przy Okregowej Izbie Lekarskiej  ul. Swietojanska 7  Bialystok, 15-082  POLAND |
|  |  |  |  |  |  |

## Russian Federation

**Coordinating Investigators:**

<None Entered>

| **Center** | **Principal Investigator** | **Co-Investigator(s)** | **Sub-Investigator(s)** | **Address(es)** | **Institutional Review Board or Ethics Committee Address(es)** |
| --- | --- | --- | --- | --- | --- |
|  |  |  |  |  |  |
| 1142 | Prof. Leonid I. Dvoretsky |  | Dr. Svetlana E. Kolendo  Dr. Elena V. Sergeeva | Clinical Hospital #7  Kolomenskyi proezd, 4  Moscow, 115446  RUSSIAN FEDERATION | Ethics Committee at the Federal Service on Surveillance in Healthcare and Social Development  8, str. 2, Petrovskij bulvar  Moscow, 127051  RUSSIAN FEDERATION  Local Ethics Committee at the Clinical Hospital #7  Kolomenskyi proezd, 4  Moscow, 115446  RUSSIAN FEDERATION |
|  |  |  |  |  |  |
| 1143 | Prof. Grigory P. Aroutyunov |  | Dr. Ekaterina Yu. Ilyina  Dr. Elena A. Kolesnikova  Dr. Karine A. Lytkina  Dr. Alexandr V. Rozanov | Russian State Medical University, Moscow Faculty, City Clinical Hospital #4, Department of Therapy  building 13  25 Pavlovskaya ulitsa  Moscow, 115093  RUSSIAN FEDERATION | Ethics Committee at the Federal Service on Surveillance in Healthcare and Social Development  8, str. 2, Petrovskij bulvar  Moscow, 127051  RUSSIAN FEDERATION  Local Ethics Committee at the City Clinical Hospital #4  building 13  25 Pavlovskaya ulitsa  Moscow, 115093  RUSSIAN FEDERATION |
|  |  |  |  |  |  |
| 1150 | Dr. Irina Mihailovna Marusenko |  | Dr. Yanina A. Avdeeva  Dr. Svetlana N. Kondrichina  Dr. Nina V. Koryakova  Dr. Irina I. Polskaya  Dr. Natalia N. Vezikova | Republican Hospital n. a. V.A.Baranov  Pirogova str., 3  Petrozavodsk, 185019  RUSSIAN FEDERATION | Ethics Committee at the Federal Service on Surveillance in Healthcare and Social Development  8, str. 2, Petrovskij bulvar  Moscow, 127051  RUSSIAN FEDERATION  Ethics Committee at the Republican Hospital n.a. V.A. Baranov  Pirogova str., 3  Petrozavodsk, 185019  RUSSIAN FEDERATION |
|  |  |  |  |  |  |

## Slovakia

**Coordinating Investigators:**

<None Entered>

| **Center** | **Principal Investigator** | **Co-Investigator(s)** | **Sub-Investigator(s)** | **Address(es)** | **Institutional Review Board or Ethics Committee Address(es)** |
| --- | --- | --- | --- | --- | --- |
|  |  |  |  |  |  |
| 1124 | Dr. Pavol Polak |  | Dr. Renata Polakova | Nestatna reumatologicka ambulancia, MUDr. Pavol Polak, s.r.o.  Vojtecha Spanyola 43  Zilina, 010 01  SLOVAKIA | Eticka komisia Zilinskeho samospravneho kraja  Komenskeho 48  Zilina, 011 09  SLOVAKIA  Eticka komisia, Trenciansky samospravny kraj  K dolnej stanici 7282/20A  Trencin, 911 01  SLOVAKIA |
|  |  |  |  |  |  |
| 1125 | Dr. Zuzana Cizmarikova |  | Miroslava Vancova | Reumatologicka ambulancia, MUDr. Zuzana Cizmarikova, s.r.o.  Karpatska 3273/11  Poprad, 058 01  SLOVAKIA | Eticka komisia Presovskeho samospravneho kraja  Namestie mieru 2  Presov, 080 01  SLOVAKIA  Eticka komisia, Trenciansky samospravny kraj  K dolnej stanici 7282/20A  Trencin, 911 01  SLOVAKIA |
|  |  |  |  |  |  |
| 1126 | Dr. Viola Husarova |  | Dr. Judita Lennerova | Reumatologicka ambulancia, REUMEX, s.r.o.  Zeleznicna 686/23  Rimavska Sobota, 979 01  SLOVAKIA | Eticka komisia, Trenciansky samospravny kraj  K dolnej stanici 7282/20A  Trencin, 911 01  SLOVAKIA  Nezavisla Eticka komisia Banskobystrickeho samospravneho kraja  Namestie SNP 23  Banska Bystrica, 974 01  SLOVAKIA |
|  |  |  |  |  |  |
| 1127 | Dr. Lubomira Simova |  | Dr. Maria Kolkusova  Emilia Kostelanska | Nestatna reumatologicka ambulancia  Nemocnicna 986  Povazska Bystrica, 017 01  SLOVAKIA | Eticka komisia, Trenciansky samospravny kraj  K dolnej stanici 7282/20A  Trencin, 911 01  SLOVAKIA |
|  |  |  |  |  |  |
| 1128 | Dr. Milan Krpciar |  | Bibiana Krpciarova | Reumatologicka ambulancia, Nestatne zdravotnicke zariadenie  Sotinska 1588  Senica, 905 01  SLOVAKIA | Eticka komisia Trnavskeho samospravneho kraja  P.O.BOX 128, Starohajska 10  Trnava, 917 01  SLOVAKIA  Eticka komisia, Trenciansky samospravny kraj  K dolnej stanici 7282/20A  Trencin, 911 01  SLOVAKIA |
|  |  |  |  |  |  |
| 1129 | Dr. Peter Belica |  | Jana Barkociova  Dr. Terezia Belicova | Reumatologicka ambulancia, Ecclesia, s.r.o.  SNP 42/A  Nove Zamky, 94001  SLOVAKIA | Eticka komisia Nitrianskeho samospravneho kraja  Stefanikova tr. 69  Nitra, 949 01  SLOVAKIA  Eticka komisia, Trenciansky samospravny kraj  K dolnej stanici 7282/20A  Trencin, 911 01  SLOVAKIA |
|  |  |  |  |  |  |

## Spain

**Coordinating Investigators:**

<None Entered>

| **Center** | **Principal Investigator** | **Co-Investigator(s)** | **Sub-Investigator(s)** | **Address(es)** | **Institutional Review Board or Ethics Committee Address(es)** |
| --- | --- | --- | --- | --- | --- |
|  |  |  |  |  |  |
| 1020 | Juan Garcia Meijide |  | Myriam Liz Graña  Manuel Pombo Suarez | HOSPITAL NUESTRA SEÑORA DE LA ESPERANZA  AVENIDA DE LAS BURGAS, 2  SANTIAGO DE COMPOSTELA, A CORUÑA 15705  SPAIN | Comite Etico de Investigacion Clinica de Galicia  SUBDIRECCION GENERAL DE FARMACIA Y PRODUCTOS SANITARIOS  EDIFICIO ADMINISTRATIVO SAN LAZARO  C/ SAN LAZARO, S/N  SANTIAGO DE COMPOSTELA, A CORUÑA 15703  SPAIN |
|  |  |  |  |  |  |
| 1021 | Francisco J. Blanco Garcia |  | Jesus Carlos Fernandez Lopez  Mercedes Freire Gonzalez  Derikah Tatiana Gonzalez Perez  Natividad Oreiro Villar | COMPLEXO HOSPITALARIO UNIVERSITARIO A CORUÑA  LABORATORIO DE INVESTIGACION. EDIFICIO ANEXO AL HOSPITAL MATERNO-INFANTIL  C/ XUBIAS DE ARRIBA, 84  A CORUÑA, A CORUÑA 15006  SPAIN | Comite Etico de Investigacion Clinica de Galicia  SUBDIRECCION GENERAL DE FARMACIA Y PRODUCTOS SANITARIOS  EDIFICIO ADMINISTRATIVO SAN LAZARO  C/ SAN LAZARO, S/N  SANTIAGO DE COMPOSTELA, A CORUÑA 15703  SPAIN |
|  |  |  |  |  |  |
| 1022 | Emilio Martin Mola |  | Alejandro Balsa Criado  Miguel Bernad Pineda  Gema Bonilla Hernan  Diana Peiteado Lopez | HOSPITAL UNIVERSITARIO LA PAZ  SERVICIO DE REUMATOLOGIA  Pº DE LA CASTELLANA, 261  MADRID, MADRID 28046  SPAIN | Comite Etico de Investigacion Clinica de Galicia  SUBDIRECCION GENERAL DE FARMACIA Y PRODUCTOS SANITARIOS  EDIFICIO ADMINISTRATIVO SAN LAZARO  C/ SAN LAZARO, S/N  SANTIAGO DE COMPOSTELA, A CORUÑA 15703  SPAIN |
|  |  |  |  |  |  |
| 1024 | Alicia Garcia Lopez |  | Pilar Maiquez Asuero  Isabel Mª Moreno Gallego  Juan Bautista Povedano Gomez | HOSPITAL UNIVERSITARIO VIRGEN DEL ROCIO  CENTRO DE DIAGNOSTICO Y TRATAMIENTO. SERVICIO DE REUMATOLOGIA, 3ª PLANTA  AVDA. MANUEL SIUROT, S/N  SEVILLA, SEVILLA 41013  SPAIN | Comite Etico de Investigacion Clinica de Galicia  SUBDIRECCION GENERAL DE FARMACIA Y PRODUCTOS SANITARIOS  EDIFICIO ADMINISTRATIVO SAN LAZARO  C/ SAN LAZARO, S/N  SANTIAGO DE COMPOSTELA, A CORUÑA 15703  SPAIN |
|  |  |  |  |  |  |
| 1049 * | Antonio Fernandez Nebro |  | Maria Angeles Belmonte Lopez  Enrique Calero Secall  Virginia Coret Cagigal  Mª Victoria Irigoyen Oyarzabal  Francisco Gabriel Jimenez Nuñez  Maria America Lopez Lasanta  Antonio Ponce Vargas  Manuel Rodriguez Perez  Inmaculada Ureña Garnica | HOSPITAL CIVIL. HOSPITAL REGIONAL UNIVERSITARIO CARLOS HAYA  PABELLON 7, 2ª PLANTA, SERVICIO DE REUMATOLOGIA  PLAZA DEL HOSPITAL CIVIL S/N  MALAGA, MALAGA 29009  SPAIN | Comite Etico de Investigacion Clinica de Galicia  SUBDIRECCION GENERAL DE FARMACIA Y PRODUCTOS SANITARIOS  EDIFICIO ADMINISTRATIVO SAN LAZARO  C/ SAN LAZARO, S/N  SANTIAGO DE COMPOSTELA, A CORUÑA 15703  SPAIN |
|  |  |  |  |  |  |

## Sweden

**Coordinating Investigators:**

<None Entered>

| **Center** | **Principal Investigator** | **Co-Investigator(s)** | **Sub-Investigator(s)** | **Address(es)** | **Institutional Review Board or Ethics Committee Address(es)** |
| --- | --- | --- | --- | --- | --- |
|  |  |  |  |  |  |
| 1136 * | Anna Rudin  Maria Bokarewa (Previous PI) |  | Mats Dehlin  Helena Forsblad d Elia | Sahlgrenska sjukhuset, Reumatologkliniken  Grona straket 14  Goteborg, 413 46  SWEDEN | Regionala Etikprovningsnamnden i Goteborg  Box 401  Goteborg, 405 30  SWEDEN |
|  |  |  |  |  |  |
| 1140 | Ann Olofsson Sahlqvist |  | Johan Back  Eva Baecklund  Helena Bjorkesten  Dan Henrohn  Anne Ireneus | Akademiska sjukhuset, Reumatologmottagningen  Ingang 30  Uppsala, 751 85  SWEDEN | Regionala Etikprovningsnamnden i Goteborg  Box 401  Goteborg, 405 30  SWEDEN |
|  |  |  |  |  |  |
| 1174 | Dr. Jorgen Lysholm |  | Dr. Helena Hellstrom  Tomas Husmark  Anna Svard | Falu Lasarett, Kliniken for Reumatologi  Falun, 791 82  SWEDEN | Regionala Etikprovningsnamnden i Goteborg  Box 401  Goteborg, 405 30  SWEDEN |
|  |  |  |  |  |  |

## Thailand

**Coordinating Investigators:**

<None Entered>

| **Center** | **Principal Investigator** | **Co-Investigator(s)** | **Sub-Investigator(s)** | **Address(es)** | **Institutional Review Board or Ethics Committee Address(es)** |
| --- | --- | --- | --- | --- | --- |
|  |  |  |  |  |  |
| 1100 | Prof. Worawit Louthrenoo |  | Assist. Prof. Nuntana Kasitanon  Suparaporn Wangkaew | Division of Rheumatology, Department of Internal Medicine, Faculty of Medicine  Chiang Mai University  110 Intavaroros Road  Amphoe Muang, Chiang Mai 50200  THAILAND | Research Ethics Committee 2, Faculty of Medicine, Chiang Mai University  110 Intavaroros Rd, Amphoe Muang  Chiang Mai, 50200  THAILAND |
|  |  |  |  |  |  |
| 1139 | Assoc.Prof Siraphop Suwannaroj |  | Assist.Prof Chingching Foocharoen  Assist.Prof. Ajanee Mahakkanukrauh  Prof. Ratanavadee Nanagara | Division of Allergy-Immunology-Rheumatology, Department of Medicine, Faculty of Medicine  Srinagarind Hospital, Khonkaen University  123 Mitraphab Road  Muang District, Khonkaen 40002  THAILAND | The Khon Kaen University Ethics Committee for Human Research  17 Floor room 1733 Somdetprasrinakarintra Building  Faculty of Medicine, Khon Kaen University  Khon Kaen,  THAILAND |
|  |  |  |  |  |  |
| 1149 | Dr. Paijit Asavatanabodee |  | Dr. Peerawat Boonyateerana  Sumapa Chaiamnuay  Chokchai Kittiyanpanya  Pongthorn Narongroeknawin  Dr. Rattapol Pakchotanon  Suphawan Phukongchai  Tarinee Rojsakulkit | Rheumatic Disease Unit, Department of Medicine, Phramongkutklao Hospital  315  Rajavithi Road,  Rajathevee, Bangkok 10400  THAILAND | Institutional Review Board Royal Thai Army Medical Department  317 Rajavithi Road  Rajathevee  Bangkok, 10400  THAILAND |
|  |  |  |  |  |  |

## United Kingdom

**Coordinating Investigators:**

<None Entered>

| **Center** | **Principal Investigator** | **Co-Investigator(s)** | **Sub-Investigator(s)** | **Address(es)** | **Institutional Review Board or Ethics Committee Address(es)** |
| --- | --- | --- | --- | --- | --- |
|  |  |  |  |  |  |
| 1054 | Dr. Emmanuel George |  | Dr. Priyanka Chandratre  Peter Chapman  Dr. Yee Ho Chiu  Sarah Gibson  Nicola Jeffries  Vipin Tayal | Arrowe Park Hospital  Department of Rheumatology  Arrowe Park Road  Wirral, Merseyside CH49 5PE  UNITED KINGDOM | West Midlands Research Ethics Commitee  West Midlands Research Ethics Commitee  Prospect House  Fishing Line Road  Enfield, Redditch B97 6EW  UNITED KINGDOM |
|  |  |  |  |  |  |
| 1055 | Dr. T. Sheeran |  | Dr. Abdul Baker  Dr. George Hirsch  Deborah Lloyd  Jacqueline McPeake  Dr. Thomas Price  Samantha Roskell  Dr S. Venkatachalam  Ramin Yazdani | Cannock Rheumatology Dept., Cannock Chase Hospital  Brunswick Road  Cannock, Staffs WS11 2XY  UNITED KINGDOM | West Midlands Research Ethics Commitee  West Midlands Research Ethics Commitee  Prospect House  Fishing Line Road  Enfield, Redditch B97 6EW  UNITED KINGDOM |
|  |  |  |  |  |  |
| 1135 | Prof. John D. Isaacs |  | Gillian M. Bell  Dr. Claire Bracewell  Dorothy Carmen  Dr. Wan Fai Ng  Dr. Martin Rynne  Linda Smith  Dr. Josephine Vila  John K. Wilson | Royal Victoria Infirmary  Clinical Research Facility  4th Floor, Leazes Wing  Newcastle Upon Tyne, NE1 4LP  UNITED KINGDOM | West Midlands Research Ethics Commitee  West Midlands Research Ethics Commitee  Prospect House  Fishing Line Road  Enfield, Redditch B97 6EW  UNITED KINGDOM |
|  |  |  |  |  |  |
| 1138 * | Dr. Shirish Dubey |  | Dr. Arvind Sinha | Solihull Hospital,  Rheumatology Department  Lode Lane  Solihull, West Midlands B91 2JL  UNITED KINGDOM | West Midlands Research Ethics Committee  Osprey House  Albert Street  Redditch, Worcestershire B97 4DE  UNITED KINGDOM |
|  |  |  |  |  |  |

## United States

**Coordinating Investigators:**

<None Entered>

| **Center** | **Principal Investigator** | **Co-Investigator(s)** | **Sub-Investigator(s)** | **Address(es)** | **Institutional Review Board or Ethics Committee Address(es)** |
| --- | --- | --- | --- | --- | --- |
|  |  |  |  |  |  |
| 1009 | Dr. Ira Francis Fenton |  | Dr. Neil B. Perlman  Cecelia Yvette Thurman | Deerbrook Medical Associates  Suite 116  565 Lakeview Parkway  Vernon Hills, IL 60061  UNITED STATES | Quorum Institutional Review Board  Suite 1000  1601 Fifth Avenue  Seattle, WA 98101  UNITED STATES |
|  |  |  |  |  |  |
| 1010 * | Dr. Guillermo Jose Valenzuela |  | Dr. Michael Andrew McLean  Dr. Richard Anthony McLean  Shawn M. Saunders | Berma Research Group  Suite B  140 SW 84th Avenue  Plantation, FL 33324  UNITED STATES | Quorum Institutional Review Board  Suite 1000  1601 Fifth Avenue  Seattle, WA 98101  UNITED STATES |
|  |  |  |  |  |  |
| 1011 * | Dr. Beata Joanna Filip-Majewski |  | Dr. Leslie D. McCasland | NEA Clinic  311 East Matthews Avenue  Jonesboro, AR 72401  UNITED STATES  NEA Clinic  Clinical Research Center  Suite C  311 East Washington Avenue  Jonesboro, AR 72401  UNITED STATES | Quorum Institutional Review Board  Suite 1000  1601 Fifth Avenue  Seattle, WA 98101  UNITED STATES |
|  |  |  |  |  |  |
| 1016 | Dr. Moges Sisay |  | Dr. Richard Earnest Bell  Dr. Mujtaba F. Tapal | TSARR, LLC  (Tri-State Arthritis and Rheumatology Research, LLC)  3801 Bellemeade Avenue, Suite 320  Evansville, IN 47714  UNITED STATES | Quorum Institutional Review Board  Suite 1000  1601 Fifth Avenue  Seattle, WA 98101  UNITED STATES |
|  |  |  |  |  |  |
| 1017 | Dr. Farrukh Zaidi |  | Dr. Lisa Mauri Cohen  Dr. M. Sami Mughni | Florida Arthritis & Osteoporosis Center  8029 Washington Street  Port Richey, FL 34668  UNITED STATES  Suncoast Clinical Research, Inc.  5604 Gulf Drive  New Port Richey, FL 34652  UNITED STATES | Quorum Institutional Review Board  Suite 1000  1601 Fifth Avenue  Seattle, WA 98101  UNITED STATES |
|  |  |  |  |  |  |
| 1018 | Dr. Steven Charles Kimmel |  | Dr. Alan Richard Alberts  Dr. Elias Halpert  Dr. Kevin Elliot Stone | West Broward Rheumatology Associates, Inc.  Suite 300  7431 North University Drive  Tamarac, FL 33321  UNITED STATES | Quorum Institutional Review Board  Suite 1000  1601 Fifth Avenue  Seattle, WA 98101  UNITED STATES |
|  |  |  |  |  |  |
| 1019 | Dr. Basit A. Malik |  | Cynthia L. James  Vicky C. Rockhill  Kimberly L. Talbot | Professional Research Network of Kansas, LLC  Suite 400  345 Riverview Street  Wichita, KS 67203  UNITED STATES | Quorum Institutional Review Board  Suite 1000  1601 Fifth Avenue  Seattle, WA 98101  UNITED STATES |
|  |  |  |  |  |  |
| 1025 * | Dr. Michael Arthur Pick |  | Dr. Jason Paul Guthrie  Dr. Jeffrey R. Horvath  Dr. Mark Allen Stern | Springfield Clinic  800 North 1st Street  Springfield, IL 62702  UNITED STATES  Springfield Clinic Research Dept.  Administrative Only  Main Campus - West Building - Suite 4300  1025 South 6th Street  Springfield, IL 62703  UNITED STATES | Quorum Institutional Review Board  Suite 1000  1601 Fifth Avenue  Seattle, WA 98101  UNITED STATES |
|  |  |  |  |  |  |
| 1027 | Dr. Geoffrey Stephen Gladstein |  | Wiltora M. Stanley  Beth Carlson Tuohy  Margaret E. Ziegler | New England Research Associates, LLC  Suite 101  5520 Park Avenue  Trumbull, CT 06611  UNITED STATES | Quorum Institutional Review Board  Suite 1000  1601 Fifth Avenue  Seattle, WA 98101  UNITED STATES |
|  |  |  |  |  |  |
| 1028 | Dr. Frederick Dietz |  | Dr. Robin Renee Hovis  Kathryn M. Lemonds-Johnson | Rockford Health Physicians  2300 North Rockton Avenue  Rockford, IL 61103-3692  UNITED STATES | Quorum Institutional Review Board  Suite 1000  1601 Fifth Avenue  Seattle, WA 98101  UNITED STATES |
|  |  |  |  |  |  |
| 1029 | Dr. Michael Thomas Stack |  | Norma S. Anderson  Barbara G. Cohen  Nancy L. Golay  Dr. Richard M. Knapek | Diagnostic Rheumatology and Research PC  Suite B-1  1030 East County Line Road  Indianapolis, IN 46227  UNITED STATES | Quorum Institutional Review Board  Suite 1000  1601 Fifth Avenue  Seattle, WA 98101  UNITED STATES |
|  |  |  |  |  |  |
| 1031 | Dr. Joel Charles Silverfield |  | Dr. Michael Claude Burnette  Dr. Laura McIlwain Cruse  Dr. Bernard F. Germain  Dr. Harris Hugh McIlwain  Dr. Kimberly McIlwain Smith | Tampa Medical Group, P.A.  Suite 406  13801 Bruce B. Downs Boulevard  Tampa, FL 33613  UNITED STATES | Quorum Institutional Review Board  Suite 1000  1601 Fifth Avenue  Seattle, WA 98101  UNITED STATES |
|  |  |  |  |  |  |
| 1032 | Dr. Robert Michael Griffin Jr. |  | Dr. Michael Allen Borofsky  Brent William Calhoon  Jane Crosby  Dr. Saurin Mrugank Mehta  Dr. Peter Daniel Nicholas Jr.  Dr. Nancy Jane Walker  Dr. Jerome Stephen Weisberg | Clinical Research Center of Reading, LLP  2760 Century Boulevard  Wyomissing, PA 19610  UNITED STATES | Quorum Institutional Review Board  Suite 1000  1601 Fifth Avenue  Seattle, WA 98101  UNITED STATES |
|  |  |  |  |  |  |
| 1033 | Dr. Michael Wayne Grisanti |  | Dr. Karen Bir  Dr. Joseph Michael Grisanti  Dr. J. Cairn Marrale  Dr. Mary Margaret O'Neil  Dr. Ivan Sabio  Marcy Sheehan  Kathleen A. Ziomek | Buffalo Rheumatology  Suite 100  3055 Southwestern Boulevard  Orchard Park, NY 14127  UNITED STATES | Quorum Institutional Review Board  Suite 1000  1601 Fifth Avenue  Seattle, WA 98101  UNITED STATES |
|  |  |  |  |  |  |
| 1035 | Dr. Edward Joel Fudman |  | Dr. Stephanie Ann Booth  Dr. Brian Sam Sayers | Austin Rheumatology Research  Suite 702  1301 West 38th Street  Austin, TX 78705  UNITED STATES  Austin Rheumatology Research  Suite 110  1301 West 38th Street  Austin, TX 78705  UNITED STATES | Quorum Review IRB  Suite 1000  1601 Fifth Avenue  Seattle, WA 98101  UNITED STATES |
|  |  |  |  |  |  |
| 1036 | Dr. Jeffrey Stewart Neal |  | Dr. Kelly K. Cole  Dr. Rita M. Egan  Dr. Paul M. Goldfarb Jr. | Bluegrass Community Research, Inc.  330 Waller Avenue  Lexington, KY 40505  UNITED STATES | Quorum Institutional Review Board  Suite 1000  1601 Fifth Avenue  Seattle, WA 98101  UNITED STATES |
|  |  |  |  |  |  |
| 1040 | Dr. Jefrey Dale Lieberman |  | Nancy E. Green | Jeffrey D. Lieberman, MD, PC  2712 North Decatur Road  Decatur, GA 30033  UNITED STATES | Quorum Institutional Review Board  Suite 1000  1601 Fifth Avenue  Seattle, WA 98101  UNITED STATES |
|  |  |  |  |  |  |
| 1044 | Dr. Mark Christopher Genovese |  | Dr. Eliza F. Chakravarty  Dr. Lorinda Susan Chung  Dr. Amy Elliot  Dr. Lily Kao  Dr. Scott T. Kawamoto  Dr. Umaima Marvi  Dr. William Hewitt Robinson  Dr. Paul J. Utz | Stanford Health Services  Medical Specialty Clinics  Room A175  300 Pasteur Drive  Stanford, CA 94305  UNITED STATES  Stanford Investigational Pharmacy  Room H0301  300 Pasteur Drive  Stanford, CA 94305  UNITED STATES  Stanford University School of Medicine  Division of Immunology and Rheumatology - Suite 203  1000 Welch Road  Palo Alto, CA 94304  UNITED STATES | Stanford University Institutional Review Board  Administrative Panel on Human Subjects in Medical Research  1215 Welch Road  Stanford, CA 94305  UNITED STATES |
|  |  |  |  |  |  |
| 1045 | Dr. Joel Marc Kremer |  | Christine J. Barr  Dr. Ludovico Frank Cavaliere  Justine S. Feder-Lailer  Dr. Neal Steven Greenstein  Kimberly B. Griner  Dr. Dorota L. Hausner-Sypek  Jessica L. Johnson  Mari V. Kaymakcian  Justine V. Kehn  Kathleen A. Kessler  Iris B. Klein  Dr. Victoria M. Michaels  Teresa M. Michaels  Rhonda L. Murphy  Dr. Norman Reid Romanoff  Dr. Harbrinder S. Sandhu  Dr. Lee Schulman Shapiro  Nicole L. Shultes  Dr. Aixa Toledo-Garcia | The Center for Rheumatology  Suite 101  1367 Washington Avenue  Albany, NY 12206  UNITED STATES | Quorum Institutional Review Board  Suite 1000  1601 Fifth Avenue  Seattle, WA 98101  UNITED STATES |
|  |  |  |  |  |  |
| 1051 | Dr. Kenneth Alan Miller |  | Sydney S. Page  Dr. Richard R. Roseff | Clinical Research Center of Connecticut  Suite 205-206  27 Hospital Avenue  Danbury, CT 06810  UNITED STATES | Quorum Institutional Review Board  Suite 1000  1601 Fifth Avenue  Seattle, WA 98101  UNITED STATES |
|  |  |  |  |  |  |
| 1052 | Dr. Mohammed Yaseen Abubaker |  | Nancy E. Green | Marietta Rheumatology  Suite 100  670 North Avenue  Marietta, GA 30060  UNITED STATES | Quorum Institutional Review Board  Suite 1000  1601 Fifth Avenue  Seattle, WA 98101  UNITED STATES |
|  |  |  |  |  |  |
| 1056 | Dr. Shelly Pearl Kafka |  | Laura J. Stavrakis  Dr. Dominick R. Woofter | Mountain State Clinical Research  Suite 303A  300 Davison Road  Clarksburg, WV 26301  UNITED STATES | Quorum Institutional Review Board  Suite 1000  1601 Fifth Avenue  Seattle, WA 98101  UNITED STATES |
|  |  |  |  |  |  |
| 1057 | Dr. Charles L. Ludivico |  | Susan Marshall Durkin  Nancy Katherine McFadden  Maxine R. Paden  Erica L. Rau  Dr. Allen Jeffrey Samuels  Dr. Ranju Singh | East Penn Rheumatology Associates, PC  Suite 501 & 601  701 Ostrum Street  Bethlehem, PA 18015  UNITED STATES | Quorum Institutional Review Board  Suite 1000  1601 Fifth Avenue  Seattle, WA 98101  UNITED STATES |
|  |  |  |  |  |  |
| 1063 | Dr. Deborah Dyett Desir |  | Dr. C. Roxana Ciubotaru  Anita B. DeAngelo  Dr. Sonia Gordon-Dole  Dr. Victor Martin  Dr. Vivian C. Shih | Arthritis & Osteoporosis Center, PC  3018 Dixwell Avenue  Hamden, CT 06518  UNITED STATES | Quorum Institutional Review Board  Suite 1000  1601 Fifth Avenue  Seattle, WA 98101  UNITED STATES |
|  |  |  |  |  |  |
| 1064 | Dr. John Joseph Condemi |  | Terry F. Arnold  Jill S. Bernhardt  Dr. Peter Michael Grace Deane  Dr. Anatole K. Kleiner  Dr. Emmanuel Adolphus Quaidoo | AAIR Research Center  Suite 305  300 Meridian Centre  Rochester, NY 14618  UNITED STATES | Quorum Institutional Review Board  Suite 1000  1601 Fifth Avenue  Seattle, WA 98101  UNITED STATES |
|  |  |  |  |  |  |
| 1075 | Dr. Geneva Louise Hill |  | DeEtte M. Burton  Dr. Josette J. Johnson  Dr. Jeffrey Geldert Lawson | Piedmont Arthritis Clinic, PA  Suite 400  3 St. Francis Drive  Greenville, SC 29601  UNITED STATES | Quorum Institutional Review Board  Suite 1000  1601 Fifth Avenue  Seattle, WA 98101  UNITED STATES |
|  |  |  |  |  |  |
| 1091 | Dr. Melvin Albert Churchill Jr. |  | Dr. Rick Charles Chatwell  Julie A. Collier  Lisa Kastanek  Anne R. Lorenz  Kristina A. Tyndall  Dr. Robert Michael Valente | Physician Research Collaboration, LLC  Suite 120  3901 Pine Lake Road  Lincoln, NE 68516  UNITED STATES | Quorum Institutional Review Board  Suite 1000  1601 Fifth Avenue  Seattle, WA 98101  UNITED STATES |
|  |  |  |  |  |  |
| 1092 | Dr. William C. Gough III |  | Dr. Kristin Marie Gowin  Audrey W. Little  Dr. Christopher G. Meyer  Dr. Ellison Leon Smith  Dr. Jill Scott Vargo  Corinne H. Yelton | Asheville Rheumatology & Osteoporosis Research Associates, P.A.  Suite 304  445 Biltmore Center  Asheville, NC 28801  UNITED STATES | Quorum Institutional Review Board  Suite 1000  1601 Fifth Avenue  Seattle, WA 98101  UNITED STATES |
|  |  |  |  |  |  |
| 1094 | Dr. Stephen Allan Bookbinder |  | Christy M. Anstead  Arthur Elkins | Ocala Rheumatology Research Center  Suite 102  3210 Southwest 33rd Road  Ocala, FL 34474  UNITED STATES | Quorum Institutional Review Board  Suite 1000  1601 Fifth Avenue  Seattle, WA 98101  UNITED STATES |
|  |  |  |  |  |  |
| 1097 | Dr. Andrew Keith Solomon |  | Dr. Julie Lyn Carkin  Dr. Richard Alan Houdek Jimenez  Myla R. Morales-Tomas  Dr. Steven Scott Overman  Dr. Jeff Regan Peterson | The Seattle Arthritis Clinic  Suite 250  10330 Meridian Avenue North  Seattle, WA 98133  UNITED STATES | Quorum Institutional Review Board  Suite 1000  1601 Fifth Avenue  Seattle, WA 98101  UNITED STATES |
|  |  |  |  |  |  |
| 1098 * | Dr. Richard Thomas Meehan |  | Linda Bannister  Dr. Marc D. Cohen | National Jewish Health  1400 Jackson Street  Denver, CO 80206  UNITED STATES | Quorum Institutional Review Board  Suite 1000  1601 Fifth Avenue  Seattle, WA 98101  UNITED STATES |
|  |  |  |  |  |  |
| 1099 | Dr. John Irving Reed  Dr. Robert Alan Yood (Previous PI) |  | Dr. John Thomas Hosey  Marcia S. Kirkpatrick  Candace LeBlanc  Dr. Julie V. Levengood  Dr. Alice A. Williams  Dr. Robert Alan Yood | Fallon Clinic, Inc.  425 North Lake Avenue  Worcester, MA 01605  UNITED STATES  Fallon Clinic, Inc.  165 Mill Street  Leominster, MA 01453  UNITED STATES | Institutional Review Board  Research Review Committee/Saint Vincent Hospital Fallon Clinic/Fallon Community  Health Plan  123 Summer Street  Worcester, MA 01608  UNITED STATES |
|  |  |  |  |  |  |
| 1132 | Dr. Ruth Ditzian Kadanoff |  | Dr. Eugene A. Bacorro  Dr. Fariha Kausar  Dr. Rochella A. Ostrowski  Dr. John Arthur Robinson II  Dr. Troy K. Takagishi | Loyola Center for Health at Oakbrook Terrace North  1S260 Summit Ave.  Oakbrook Terrace, IL 60181  UNITED STATES  Loyola University Medical Center  2160 South First Avenue  Maywood, IL 60153  UNITED STATES | Loyola University Medical Center  Institutional Review Board for the Protection of Human Subjects  2160 South First Avenue  Maywood, IL 60153  UNITED STATES |
|  |  |  |  |  |  |
| 1133 | Dr. Robert Emil Ettlinger |  | Dr. George Howard Krick  Neil F. Moody Jr.  Teresa A. Unkrur | Tacoma Center for Arthritis Research, PS  Suite 204  1901 South Cedar Street  Tacoma, WA 98405-2308  UNITED STATES  Tacoma Center for Arthritis Research, PS  Suite 201  1901 South Cedar Street  Tacoma, WA 98405  UNITED STATES | Quorum Institutional Review Board  Suite 1000  1601 Fifth Avenue  Seattle, WA 98101  UNITED STATES |
|  |  |  |  |  |  |
| 1134 | Dr. Roy Mitchell Fleischmann |  | Jean A. Clark  Dr. Stanley Bruce Cohen  Dr. Thomas David Geppert  Dr. Imran Iqbal  Dr. Robert Neil Jenkins  Dr. Talat Jehan Kheshgi  Dr. Zoran Kurepa  Dr. Sharad Lakhanpal  Andrea S. Martin  Dr. Catalina Orozco  Dr. Richard L. Stern  Dayna S. Swan-Flanders  Dr. Jack Bernstein Vine  Andrea S. Wheeler | Metroplex Clinical Research Center  Suite 810  8144 Walnut Hill Lane  Dallas, TX 75231  UNITED STATES | Quorum Institutional Review Board  Suite 1000  1601 Fifth Avenue  Seattle, WA 98101  UNITED STATES |
|  |  |  |  |  |  |
| 1175 | Dr. Richard James Misischia |  | Dr. Robert J. Capps  Dr. Marcin T. Gornisiewicz  Nickie L. Hurst  Cathy C. Rhodes  Dr. Jay Henderson Warrick  Dr. Donna M. Winn  Dr. John Frederick Wolfe | Rheumatology Consultants, PLLC  Suite 200 - Colony Park  4707 Papermill Drive  Knoxville, TN 37909-1900  UNITED STATES | Quorum Institutional Review Board  Suite 1000  1601 Fifth Avenue  Seattle, WA 98101  UNITED STATES |
|  |  |  |  |  |  |
| 1178 * | Dr. Jody Kay Hargrove |  | Dr. Paul Harvey Waytz  Dr. David Charles Zoschke | Arthritis and Rheumatology Consultants, PA  Suite 215  7250 France Avenue South  Edina, MN 55435  UNITED STATES | Quorum Institutional Review Board  Suite 1000  1601 Fifth Avenue  Seattle, WA 98101  UNITED STATES |
|  |  |  |  |  |  |
| 1180 | Dr. William Julius Shergy |  | Dr. Kun Chen  Carol Ann Cholewa  Dr. Jesus Hernandez  Dr. Robert Edward Hunt  Dr. Robert Macon Phillips Jr. | Rheumatology Associates of North Alabama, PC  Suites 600 & 620  201 Sivley Road  Huntsville, AL 35801  UNITED STATES | Quorum Institutional Review Board  Suite 1000  1601 Fifth Avenue  Seattle, WA 98101  UNITED STATES |
|  |  |  |  |  |  |
| 1181 | Dr. Emily Jane Herron Box |  | Dr. John Franklyn Babich  Dr. Patrick N. Box  Dr. Ashrito Kumar Dayal | Box Arthritis & Rheumatology of the Carolinas, PLLC  Suite 100  10502 Park Road  Charlotte, NC 28210  UNITED STATES | Quorum Institutional Review Board  Suite 1000  1601 Fifth Avenue  Seattle, WA 98101  UNITED STATES |
|  |  |  |  |  |  |

## Venezuela

**Coordinating Investigators:**

<None Entered>

| **Center** | **Principal Investigator** | **Co-Investigator(s)** | **Sub-Investigator(s)** | **Address(es)** | **Institutional Review Board or Ethics Committee Address(es)** |
| --- | --- | --- | --- | --- | --- |
|  |  |  |  |  |  |
| 1038 | Dr. Sol Villegas de Morales |  | Maria Gabriela Perez  Oscar Urdaneta | Hospital Vargas de Caracas, Servicio de Reumatologia, Edificio Anexo  Esquina del Carmen  San Jose  Caracas, Distrito Capital 1010  VENEZUELA | Hospital Vargas de Caracas  Comité de Ética  san José del Avila  Caracas, Distrito Capital 1010  VENEZUELA |
|  |  |  |  |  |  |
| 1165 | Dr. Ernesto Hercules |  | Arabia Marelli  Genny Adriana Toldo | Clinica Atias  40273 Caracas  Avenida Roosevelt  Urbanizacion Los Rosales  Caracas, DC/ Municipio Libertados 1040-A  VENEZUELA | Clinica Atias  Comite de Etica  Avenida Roosevelt  Urbanizacion Los Rosales  Caracas, DC/ Municipio Municipio Libertados 1040-A  VENEZUELA |
|  |  |  |  |  |  |
