# Supplementary material for: Changes in serum creatinine in patients with active rheumatoid arthritis treated with tofacitinib: results from clinical trials
Source: Arthritis Res Ther. 2014 Jul 25;16(4):R158. doi: 10.1186/ar4673 (PMC4220634; doi:10.1186/ar4673)
Supplement: Supplementary file 12 — Additional file 12: List of Investigators and Corresponding Ethics Committees or Institutional Review Boards for the Phase 3 A3921064 study. (DOC 417 KB) [file 13075_2013_4378_MOESM12_ESM.doc]

# 16.1.4 LIST OF INVESTIGATORS AND CORRESPONDING ETHICS COMMITTEES OR INSTITUTIONAL REVIEW BOARDS

## Australia

**Coordinating Investigators:**

<None Entered>

| **Center** | **Principal Investigator** | **Co-Investigator(s)** | **Sub-Investigator(s)** | **Address(es)** | **Institutional Review Board or Ethics Committee Address(es)** |
| --- | --- | --- | --- | --- | --- |
|  |  |  |  |  |  |
| 1071 | Dr. David H. Bossingham |  | Karen Cooke  Dr. Joshua P. Hanson  Kathryn Leonard | Rheumatology Research  5th Floor Block B  Cairns Base Hospital  The Esplanade  Cairns, QLD 4870  AUSTRALIA | Cairns and Hinterland Health Service District Human Research Ethics Committee  4th Floor, Block A  Cairns Base Hospital  Cairns, QLD 4870  AUSTRALIA |
|  |  |  |  |  |  |
| 1072 | Assoc. Prof. Stephen Hall |  | Dr. Vivienne Beckett  Dr Jill Bell  Dr Malcolm Clark  Dr Jennifer Davey  Ms Stephanie Dolejs  Dr. Marie Feletar  Dr. Andrew Gibson  Gail Grant  Dr. James McDonald  Dr Nicole McKay  Dr Louise Murdoch  Dr. Veronique Sayag-Boukris | Emeritus Research  291 Wattletree Road  Malvern East, VIC 3145  AUSTRALIA | Cabrini Human Research Ethics Committee  183 Wattletree Road  Malvern, VIC 3144  AUSTRALIA |
|  |  |  |  |  |  |
| 1073 | Prof. Peter T. Nash |  | Dr. Frances Johnson  Dr. David Nicholls  Ms. Dale Shergold  Ms. Jan Smith  Dr. Susan Thackwray  Dr. Avgeania Voight | Rheumatology Research Unit Sunshine Coast  9-10 Maroochy Waters Shopping Centre  Denna Street  Maroochydore, QLD 4558  AUSTRALIA | Redcliffe-Caboolture Ethics Committee  Unit 1, Ground Floor, Redcliffe Hospital  Anzac Avenue  Redcliffe, Qld 4020  AUSTRALIA |
|  |  |  |  |  |  |
| 1074 | A/Prof. Leslie Schrieber |  | Dr. Mark Arnold  Ms. Lyndall Henderson | Royal North Shore Hospital  Department of Rheumatology  Pacific Highway  St Leonards, NSW 2065  AUSTRALIA | Northern Sydney and Central Coast Area Health Service  Human Research Ethics Committees  Royal North Shore Hospital  Pacific Hwy  St Leonards, NSW 2065  AUSTRALIA |
|  |  |  |  |  |  |

## Bosnia And Herzegovina

**Coordinating Investigators:**

<None Entered>

| **Center** | **Principal Investigator** | **Co-Investigator(s)** | **Sub-Investigator(s)** | **Address(es)** | **Institutional Review Board or Ethics Committee Address(es)** |
| --- | --- | --- | --- | --- | --- |
|  |  |  |  |  |  |
| 1150 | Assist.Prof. Sekib Sokolovic |  | Dr. Sida Kasumagic  Dr. Jasmina Krehic | University Clinical Center Sarajevo  Bolnicka 25  Sarajevo, 71000  BOSNIA AND HERZEGOVINA | Ethics Committee  University Clinical Center Sarajevo  Bolnicka 25  Sarajevo, 71000  BOSNIA AND HERZEGOVINA |
|  |  |  |  |  |  |

## Bulgaria

**Coordinating Investigators:**

<None Entered>

| **Center** | **Principal Investigator** | **Co-Investigator(s)** | **Sub-Investigator(s)** | **Address(es)** | **Institutional Review Board or Ethics Committee Address(es)** |
| --- | --- | --- | --- | --- | --- |
|  |  |  |  |  |  |
| 1006 | Dr. Boycho Oparanov |  | Dr. Borislava Angelova Ilchova  Dr. Raycho Raychev  Dr. Ignat Zhutev | MBAL na Voennomeditsinska Akademia - Sofia  Klinika po Revmatologia i Kardiologia  MMA HAT Sofia  Ul. "Georgi Sofiyski" 3  Sofia, 1606  BULGARIA | Ethics Committee for Multicenter Trials  ul. "Damyan Gruev" 8  Sofia, 1303  BULGARIA  Komisiya po etika pri MBAL na Voennomeditsinska Akademia - Sofia/Ethics Commettee at MMA HAT-Sofia  MMA HAT-Sofia  Ul. Georgi Sofiyski  3  Sofia, 1606  BULGARIA |
|  |  |  |  |  |  |
| 1007 | Dr. Kiril Yablanski |  | Ivan Gerchev  Dr. Virzhiniya Yordanova | UMBAL "D-r Georgi Stranski" Pleven  Klinika po Kardiologia i Revmatologia  MHAT "Dr. Georgi Stranski" Pleven  Ul. "Georgi Kochev" 8A  Pleven, 5800  BULGARIA | Ethics Committee for Multicenter Trials  ul. "Damyan Gruev" 8  Sofia, 1303  BULGARIA  Komisiya po etika pri UMBAL"D-r Georgi Stranski"/ Ethics Committee at MHAT "Dr. Georgi Stranski"  UMBAL"D-r Georgi Stranski" Pleven  MHAT "Dr. Georgi Stranski" Pleven  Ul. Georgi Kochev  8A  Pleven, 5800  BULGARIA |
|  |  |  |  |  |  |
| 1008 | Anastas Batalov |  | Dr. Rositsa Karalilova  Dr. Aneta Nikolova  Dr. Dimitar Penev | MBAL "Kaspela" Plovdiv, Otdelenie po revmatologia  MHAT "Kaspela" Plovdiv  ul. Sofiya  64  Plovdiv, 4002  BULGARIA | Ethics Committee for Multicenter Trials  ul. "Damyan Gruev" 8  Sofia, 1303  BULGARIA  Komisiya po etika kam MBAL "Kaspela"/ Ethics committee at MHAT "Kaspela"  MBAL 'Kaspela' Plovdiv  MHAT 'Kaspela' Plovdiv  Ul. Sofiya 64  Plovdiv, 4002  BULGARIA |
|  |  |  |  |  |  |
| 1075 | Dr. Daniela Bichovska |  | Dr. Ivan Bichovski  Emilia F. Fileva-Veleva | DKTs "Sveta Anna", Sofia; Konsultativen kabinet po Revmatologia  Diagnostic Consultative Center "Sveta Anna"  Ul. Dimitar Mollov 1  Sofia, 1709  BULGARIA | Ethics Committee for Multicenter Trials  ul. "Damyan Gruev" 8  Sofia, 1303  BULGARIA  Komisiya po etika pri DKTs"Sv. Anna"/ Ethics Committee at DCC "Sv. Anna"  DKTs "Sveta Anna"  Diagnostic Consultative Center "Sveta Anna"  Ul. Dimitar Mollov 1  Sofia, 1709  BULGARIA |
|  |  |  |  |  |  |
| 1130 | Dr. Ivan Goranov |  | Katya Kuchmova  Mariana Panayotova | Revmatologichno Otdelenie, MBAL - Plovdiv  MHAT Plovdiv  bul. Bulgaria 234  Plovdiv, 4000  BULGARIA | Ethics Committee for Multicenter Trials  ul. "Damyan Gruev" 8  Sofia, 1303  BULGARIA  Komisiya po etika pri MBAL-Plovdiv/Ethics Committee at MHAT-Plovdiv  MBAL-Plovdiv  MHAT-Plovdiv  bul. ¿Bulgaria¿ 234  Plovdiv, 4000  BULGARIA |
|  |  |  |  |  |  |
| 1175 | Nikolay G. Nikolov |  | Dr. Todor Hinov  Uzunov Ivan  Hristina P. Kostadinova  Asen V. Kutsarov  Dr. Borislav S. Nikolov  Ignat C. Petkov | DKTs Akta Medika EOOD, Konsultativen kabinet po Revmatologia  Diagnostic Consultative Center ¿Akta Medika¿ EOOD  ul. Nikola Petkov 60  Sevlievo, 5400  BULGARIA | Ethics Committee for Multicenter Trials  ul. "Damyan Gruev" 8  Sofia, 1303  BULGARIA  Komisiya po etika pri DKTs"Akta Medika"/ Ethics Committee at DCC "Akta Medika"  DKTs"Akta Medika" EOOD  Diagnostic Consultative Center "Akta Medika" EOOD  ul. "Nikola Petkov" 60  Sevlievo, 5400  BULGARIA |
|  |  |  |  |  |  |

## Canada

**Coordinating Investigators:**

<None Entered>

| **Center** | **Principal Investigator** | **Co-Investigator(s)** | **Sub-Investigator(s)** | **Address(es)** | **Institutional Review Board or Ethics Committee Address(es)** |
| --- | --- | --- | --- | --- | --- |
|  |  |  |  |  |  |
| 1009 | Dr. Andrew Chow |  | Dr. Ami Mody  Dr. Elaine Soucy | Credit Valley Rheumatology  Suite 209  2300 Eglinton Avenue West  Mississauga, ON L5M 2V8  CANADA | IRB Services  Suite 300  372 Hollandview Trail  Aurora, ON L4G 0A5  CANADA |
|  |  |  |  |  |  |
| 1011 * | Dr. Arthur Bookman |  | Dr. Robert Inman  Dr. Finbar Barry OShea | Toronto Western Hospital  399 Bathurst Street  Toronto, ON M5T 2S8  CANADA | University Health Network Research Ethics Board  8th Floor Room 8-23  700 University Avenue  Toronto, ON M5G 1Z5  CANADA |
|  |  |  |  |  |  |
| 1012 | Dr. Alice V. Klinkhoff |  | Dr. Kenneth Blocka  Dr. Andrew Chalmers  Bruce Clark  Gillian Parker | The Arthritis Research Centre of Canada  895 W 10th Avenue  Vancouver, BC V5Z 1L7  CANADA | IRB Services  Suite 300  372 Hollandview Trail  Aurora, ON L4G 0A5  CANADA |
|  |  |  |  |  |  |
| 1013 | Dr. Andre Damien Beaulieu |  | Sylvie L'Archer  Dr. Pierre Lachance  Ms. Johanne Rioux  Dr. Rene Therrien | Centre de rhumatologie St-Louis  Bureau 140  3165 chemin St-Louis  Quebec, QC G1W 4R4  CANADA | IRB Services  Suite 300  372 Hollandview Trail  Aurora, ON L4G 0A5  CANADA |
|  |  |  |  |  |  |
| 1014 | Dr. Janet Pope |  | Lindsey Carroll  Dr. Gina Rohekar  Dr. Sherry Rohekar | St. Joseph's Health Centre  Monsignor Roney Building, Level D2  268 Grosvenor Street  London, ON N6A 4V2  CANADA | Office of Research Ethics, University of Western Ontario  Support Services Building  Room 5150  1393 Western Road  London, ON N6A 5C1  CANADA |
|  |  |  |  |  |  |
| 1016 | Dr. Jean-Luc Tremblay |  | Dr. Michele Dessureault  Dr. Clode Lessard  Dr. Frederic Morin  Louise Rouleau  Ms. Carmen Trudel | Centre de Recherche Musculo-Squelettique  1119 Ste Marguerite  Trois-Rivieres, QC G8Z 1Y2  CANADA | IRB Services  Suite 300  372 Hollandview Trail  Aurora, ON L4G 0A5  CANADA |
|  |  |  |  |  |  |
| 1017 | Dr. Bindu Nair |  | Dr. Zohreh Sabbagh  Dr. Regina Taylor-Gjevre | Royal University Hospital  Room 367 Ellis Hall  103 Hospital Drive  Saskatoon, SK S7N 0W8  CANADA | University of Saskatchewan Biomedical Research Ethics Board  Ethics Office  P.O. Box 5000 RPO University  Saskatoon, SK S7N 0W8  CANADA  University of Saskatchewan BioMedical Research Ethics Board (Bio-REB)  Research Ethics Office, University of Saskatchewan  NRC, Plant Biotechnology Research Institute  1607 110 Gymnasium Place  Saskatoon, SK S7N 4J8  CANADA |
|  |  |  |  |  |  |
| 1045 * | Dr. Brett Hennenfent  Dr. Diane B. Wilson (Previous PI) |  | Dr. Diane B. Wilson | Office of Dr. Diane Wilson  PO Box 1617  78 Masons Beach Road  Lunenburg, NS B0J 2C0  CANADA | IRB Services  Suite 300  372 Hollandview Trail  Aurora, ON L4G 0A5  CANADA |
|  |  |  |  |  |  |

## Chile

**Coordinating Investigators:**

<None Entered>

| **Center** | **Principal Investigator** | **Co-Investigator(s)** | **Sub-Investigator(s)** | **Address(es)** | **Institutional Review Board or Ethics Committee Address(es)** |
| --- | --- | --- | --- | --- | --- |
|  |  |  |  |  |  |
| 1001 | Dr. Marta Aliste |  | Dr. Julio Cruz  Dr. Hector Gatica  Dr. Annaelise Goecke  Dr. Sandra Pino  Dr. Francisca Sabugo  Dr. Lilian Soto  Dr. Pamela Wurmann | Consulta Privada Dra. Marta Aliste  Guardia Vieja 255, Oficina 1409  Providencia  Santiago, RM 7510186  CHILE | Comite Etico Cientifico, Servicio de Salud Metropolitano Oriente  Avenida Salvador 364  Providencia  Santiago, RM 7500922  CHILE |
|  |  |  |  |  |  |
| 1002 | Dr. Francisco Ballesteros |  | Dr. Maria Eugenia Alvarez  Dr. Loreto Ovalle  Dr. Paula Pozo | Centro de Diagnostico y Tratamiento San Borja Arriaran  Seccion Reumatologia  Amazonas 619  Santiago, RM 8360156  CHILE | Comite Etico Cientifico  Servicio de Salud Metropolitano Oriente  Avenida Salvador 364  Providencia, Santiago, RM 7500922  CHILE |
|  |  |  |  |  |  |
| 1003 | Dr. Gloria Holuigue |  | Juan Manuel Contreras  Luis Orellana | Clínica Santa Maria  Seccion Reumatologia  Fernando Manterola 0540  Providencia, Santiago, RM 7530206  CHILE | Comite de Etica  Clinica Santa Maria  Avenida Bellavista 0415  2 Piso, Oficina 78  Providencia, Santiago  CHILE  Comite Etico Cientifico  Servicio de Salud Metropolitano Oriente  Avenida Salvador 364  Providencia, Santiago, RM 7500922  CHILE |
|  |  |  |  |  |  |
| 1174 | Dr. Ana Maria Flores |  | Diego Carvajal  Miguel Valenzuela | Hospital Regional de Rancagua  Alameda #611  Rancagua, VI Region 2841959  CHILE | Comite Etico Cientifico  Servicio de Salud Metropolitano Oriente  Avenida Salvador 364  Providencia, Santiago, RM 7500922  CHILE |
|  |  |  |  |  |  |

## Costa Rica

**Coordinating Investigators:**

<None Entered>

| **Center** | **Principal Investigator** | **Co-Investigator(s)** | **Sub-Investigator(s)** | **Address(es)** | **Institutional Review Board or Ethics Committee Address(es)** |
| --- | --- | --- | --- | --- | --- |
|  |  |  |  |  |  |
| 1136 | Dr. Raul Alpizar |  | Victor Cisneros-Perez  Dr. Allan Soto-Calvo | Centro de Reumatología y Osteoporosis, Cartago  de la esquina noroeste del Convento de los Padres Capuchinos  75 metros al este  Cartago, Cartago  COSTA RICA | UCIMED  Comite Etico Cientifico de la Universidad de Ciencias Medicas  400 mts Oeste del Ministerio de Agricultura y Ganaderia  Sabana Oeste  San Jose,  COSTA RICA |
|  |  |  |  |  |  |
| 1137 | Dr. Francisco Brenes-Silesky |  | Natalia Bustamante  Dr. Duncan Manley-Fredrich | Hospital Clinica Biblica  Calle central y 1, Av. 14 y 16  San Jose, San Jose  COSTA RICA | UCIMED  Comite Etico Cientifico de la Universidad de Ciencias Medicas  400 mts Oeste del Ministerio de Agricultura y Ganaderia  Sabana Oeste  San Jose,  COSTA RICA |
|  |  |  |  |  |  |
| 1145 | Dr. Daniel Alfaro-Vargas |  | Fernando Cerdas  Raul Sotomayor MD | Hospital Cima San Jose  De Multiplaza Escazu, 600 mts este, carretera Prospero Fernandez, contiguo a PriceMart  Torre 1, Piso 5  San Jose, San Jose 00  COSTA RICA | UCIMED  Comite Etico Cientifico de la Universidad de Ciencias Medicas  400 mts Oeste del Ministerio de Agricultura y Ganaderia  Sabana Oeste  San Jose,  COSTA RICA |
|  |  |  |  |  |  |

## Croatia

**Coordinating Investigators:**

<None Entered>

| **Center** | **Principal Investigator** | **Co-Investigator(s)** | **Sub-Investigator(s)** | **Address(es)** | **Institutional Review Board or Ethics Committee Address(es)** |
| --- | --- | --- | --- | --- | --- |
|  |  |  |  |  |  |
| 1146 | Assistant Prof.dr. Dusanka Martinovic Kaliterna |  | Dr. Daniela Marasovic Krstulovic  Dr. Mislav Radic | University Hospital Split, Department for Internal Medicine, Division of Clinical Rheumatology  Spinciceva1  Split, 21000  CROATIA | Central Ethics Committee  Agency for Medicinal Products and Medical Devices  Ksaverska c. 4  Zagreb, 10000  CROATIA |
|  |  |  |  |  |  |
| 1149 | Prof.dr Nada Cikes |  | Dr. Branimir Anic  Dr. Marko Baresic  Dr. Mislav Cerovec  Dr. Mirna Sentic | University Hospital Center Zagreb  Kispaticeva 12  Zagreb, 10000  CROATIA | Central Ethics Committee  Agency for Medicinal Products and Medical Devices  Ksaverska c. 4  Zagreb, 10000  CROATIA |
|  |  |  |  |  |  |
| 1155 | Dr. Visnja Prus |  | Dr. Drazen Bedekovic  Doc.dr. Jasminka Milas Ahic  Dr. Roberta Visevic | Deaprtment of Internal Medicine  University Hospital Osijek  J.Huttlera 4  Osijek, 31000  CROATIA | Central Ethics Committee  Agency for Medicinal Products and Medical Devices  Ksaverska c. 4  Zagreb, 10000  CROATIA |
|  |  |  |  |  |  |

## Czech Republic

**Coordinating Investigators:**

<None Entered>

| **Center** | **Principal Investigator** | **Co-Investigator(s)** | **Sub-Investigator(s)** | **Address(es)** | **Institutional Review Board or Ethics Committee Address(es)** |
| --- | --- | --- | --- | --- | --- |
|  |  |  |  |  |  |
| 1101 | Dr. Jan Rosa |  | Dr. Petr Kasalicky  Alena Slechticka | DC Mediscan  Sustova 1930  Praha 11 - Chodov, 148 00  CZECH REPUBLIC  MUDr. Dana Sinaglova  Soukroma laborator klinicke biochemie a hematologie  Sustova 1930  Praha 11, 14800  CZECH REPUBLIC  Sdruzene zdravotnicke zarizeni JM II  Radiodiagnosticke oddeleni  Sustova 1930  Praha 11, 14800  CZECH REPUBLIC  Sdruzene zdravotnicke zarizeni JM II  Lekarna  Sustova 1930  Praha 11, 14800  CZECH REPUBLIC | Eticka komise IKEM a FTNsP  Videnska 800  Praha 4 Krc, 140 59  CZECH REPUBLIC |
|  |  |  |  |  |  |
| 1102 | Dr. Petr Nemec |  | Dr. Jana Bohmova  Dr. Leona Prochazkova  Dr. Vlastimil Racek | Fakultni nemocnice u sv. Anny v Brne  II. Interni klinika  Pekarska 53  Brno, 656 91  CZECH REPUBLIC | Eticka komise Fakultni nemocnice u sv. Anny v Brne  Pekarska 53  Brno, 656 91  CZECH REPUBLIC  Eticka komise IKEM a FTNsP  Videnska 800  Praha 4 Krc, 140 59  CZECH REPUBLIC |
|  |  |  |  |  |  |
| 1103 | Dr. Petr Vitek |  | Dr. Olga Januskova | PV-Medical s.r.o.  Revmatologicka ambulance  Padelky I/3645  Zlin, 760 01  CZECH REPUBLIC | Eticka komise IKEM a FTNsP  Videnska 800  Praha 4 Krc, 140 59  CZECH REPUBLIC |
|  |  |  |  |  |  |
| 1104 | Dr. Sarka Forejtova |  | Dr. Katerina Jarosova  Dr. Dana Tegzova  Prof. Jiri Vencovsky | Revmatologicky ustav  Na Slupi 4  Praha 2, 128 50  CZECH REPUBLIC | Eticka komise IKEM a FTNsP  Videnska 800  Praha 4 Krc, 140 59  CZECH REPUBLIC |
|  |  |  |  |  |  |
| 1105 | Dr. Zdenek Dvorak |  | Lucie Koubova  Petra Stefanova  Dr. Martina Vaneckova | ARTHROMED, s. r. o. Revmatologicka ambulance  Rokycanova 2798  Pardubice, 530 02  CZECH REPUBLIC | Eticka komise IKEM a FTNsP  Videnska 800  Praha 4 Krc, 140 59  CZECH REPUBLIC |
|  |  |  |  |  |  |
| 1151 | Dr. Libor Novosad |  | Jana Jaluvkova | L.K.N. Arthrocentrum, s.r.o.  Na Valech 1  Hlucin, 748 01  CZECH REPUBLIC | Eticka komise IKEM a FTNsP  Videnska 800  Praha 4 Krc, 140 59  CZECH REPUBLIC |
|  |  |  |  |  |  |
| 1152 | Dr. Zuzana Urbanova |  | Dr. Zuzana Stejfova | Revmatologicka ambulance  Petra Rezka 3  Praha 4, 140 00  CZECH REPUBLIC | Eticka komise IKEM a FTNsP  Videnska 800  Praha 4 Krc, 140 59  CZECH REPUBLIC |
|  |  |  |  |  |  |
| 1153 | Dr. Zdenka Mosterova |  | Dr. Erik Moster  Dr. Rene Moster | D. L. Pharma s.r.o.  Cejl 5  Brno, 60200  CZECH REPUBLIC  Fakultni nemocnice u sv. Anny v Brne  Radiodiagnosticka klinika  Pekarska 53  Brno, 65691  CZECH REPUBLIC  Revmacentrum MUDr. Mostera, s.r.o.  Mosnova 8  Brno - Zidenice, 615 00  CZECH REPUBLIC | Eticka komise IKEM a FTNsP  Videnska 800  Praha 4 Krc, 140 59  CZECH REPUBLIC |
|  |  |  |  |  |  |

## Denmark

**Coordinating Investigators:**

<None Entered>

| **Center** | **Principal Investigator** | **Co-Investigator(s)** | **Sub-Investigator(s)** | **Address(es)** | **Institutional Review Board or Ethics Committee Address(es)** |
| --- | --- | --- | --- | --- | --- |
|  |  |  |  |  |  |
| 1080 | Dr. Bente Danneskiold-Samsoe |  | Dr. Henning Bliddal  Dr. Henrik Gudbergsen  Dr. Birgit Falk Riecke  Dr. Anja Falk Riecke | H:S Frederiksberg Hospital  Parker Instituttet  Ndr. Fasanvej 57-59  Frederiksberg, 2000  DENMARK | De Videnskabsetiske Komitéer for Region Hovedstaden  Regionsgaarden  Kongens Vaenge 2  Hilleroed, 3400  DENMARK |
|  |  |  |  |  |  |
| 1082 | Dr. Peter Mosborg Petersen |  | Dr. Martin Bak  Dr. Kaare Schoett Mai  Ms. Birthe Pedersen  Dr. Henrik Simonsen | Regionshospitalet Randers, Medicinsk Afdeling M  Skovlyvej 1  Randers NOE, 8930  DENMARK | De Videnskabsetiske Komitéer for Region Hovedstaden  Regionsgaarden  Kongens Vaenge 2  Hilleroed, 3400  DENMARK |
|  |  |  |  |  |  |

## Dominican Republic

**Coordinating Investigators:**

<None Entered>

| **Center** | **Principal Investigator** | **Co-Investigator(s)** | **Sub-Investigator(s)** | **Address(es)** | **Institutional Review Board or Ethics Committee Address(es)** |
| --- | --- | --- | --- | --- | --- |
|  |  |  |  |  |  |
| 1005 | Patricia Alvarez-Felix |  | Rafael Alba-Ferriz  Iraquel Cordero  Roberto Munoz-Louis  Miriam Nova  Cinthia Rivas | Latin American Research  Avenida Maximo Gomez #60, suite 201, plaza paseo del treatro  Santo Domingo, Santo Domingo 00000  DOMINICAN REPUBLIC | CONABIOS  Universidad Católica Santo Domingo  Calle Santo Domingo No3, Ens. La Julia  Santo Domingo, Santo Domingo 2733  DOMINICAN REPUBLIC |
|  |  |  |  |  |  |

## Finland

**Coordinating Investigators:**

<None Entered>

| **Center** | **Principal Investigator** | **Co-Investigator(s)** | **Sub-Investigator(s)** | **Address(es)** | **Institutional Review Board or Ethics Committee Address(es)** |
| --- | --- | --- | --- | --- | --- |
|  |  |  |  |  |  |
| 1024 | Pentti Jarvinen |  | Timo Ahtikari  Sirkka Koskinen | Kiljavan Laaketutkimus Oy  Donnerinkatu 5  Hyvinkaa, 05800  FINLAND | Helsingin ja Uudenmaan sairaanhoitopiiri  Sisatautien eettinen toimikunta  Biomedicum Helsinki 2 C  PL 705  HUS, 00029  FINLAND |
|  |  |  |  |  |  |

## Germany

**Coordinating Investigators:**

<None Entered>

| **Center** | **Principal Investigator** | **Co-Investigator(s)** | **Sub-Investigator(s)** | **Address(es)** | **Institutional Review Board or Ethics Committee Address(es)** |
| --- | --- | --- | --- | --- | --- |
|  |  |  |  |  |  |
| 1087 | Dr. med. Rieke Alten |  | Dr. med. Stefan Bieneck  Annalina Braun  Svitlana Djacenko  Dr. med. Christoph Pohl  Olaf Schroeder | Schlosspark-Klinik, Innere Medizin II, Rheumatologie  Heubnerweg 2  Berlin, 14059  GERMANY | Ethik-Kommission des Fachbereichs Medizin der Justus-Liebig-Universitaet Giessen  Gaffkystrasse 11 c  Giessen, 35385  GERMANY |
|  |  |  |  |  |  |
| 1093 | Prof. Dr. Juergen Braun |  | Dr. Friedrich Dybowski  Dr. Claas Fendler  Dr. Frank Heldmann  Dr. Ertan Saracbasi-Zender | St. Josefs-Krankenhaus, Rheumazentrum Ruhrgebiet  Landgrafenstr. 15  Herne, 44652  GERMANY | Ethik-Kommission des Fachbereichs Medizin der Justus-Liebig-Universitaet Giessen  Gaffkystrasse 11 c  Giessen, 35385  GERMANY |
|  |  |  |  |  |  |
| 1096 * | Dr. Anke Liebhaber |  | Dr. Karin Babinsky | Rheumapraxis Dres. Liebhaber und Babinsky  Pestalozzistr. 2A  Halle, 06128  GERMANY | Ethik-Kommission des Fachbereichs Medizin der Justus-Liebig-Universitaet Giessen  Gaffkystrasse 11 c  Giessen, 35385  GERMANY |
|  |  |  |  |  |  |
| 1097 | Prof. Dr. Hans-Peter Tony |  | Dr. Martin Feuchtenberger  Dr. Stefan Kleinert  Yvonne Kochler  Annette Kuhn  Dr. med. Silke Osiek  Dr. Eva Ostermeier  Gabriele Rothenbucher | Universitaetsklinikum Wuerzburg, Medizinische Klinik II, Rheumatologie/Immunologie, A3.-1.923  Oberduerrbacherstr. 6  Wuerzburg, 97080  GERMANY | Ethik-Kommission des Fachbereichs Medizin der Justus-Liebig-Universitaet Giessen  Gaffkystrasse 11 c  Giessen, 35385  GERMANY |
|  |  |  |  |  |  |
| 1098 | Dr. Siegfried Wassenberg |  | Peter Fretter  Dr. med. Markus Jost  Dorothea Longerich-Scheuss  Georg Raspe  Dr. med. Ralf Weier | Evangelisches Fachkrankenhaus Ratingen gGmbH  Rosenstr. 2  Ratingen, 40882  GERMANY | Ethik-Kommission des Fachbereichs Medizin der Justus-Liebig-Universitaet Giessen  Gaffkystrasse 11 c  Giessen, 35385  GERMANY |
|  |  |  |  |  |  |
| 1132 | Dr. med. Reiner Kurthen |  | Ayse Ece  Tom Kurthen  Bianca Lobenstein | Privat-Praxis, Rheumatologie (P515)  Karlsgraben 15  Aachen, 52064  GERMANY | Ethik-Kommission des Fachbereichs Medizin der Justus-Liebig-Universitaet Giessen  Gaffkystrasse 11 c  Giessen, 35385  GERMANY |
|  |  |  |  |  |  |
| 1135 | Prof. Dr. med. Harald Burkhardt |  | Dr. med. Frank Behrens  Dr. med. Carolin Brueck  Dr. med. Michaela Koehm  Dr. med. Nicola Reuschling | J.W.-Goethe-Universitaetsklinik, Medizinische Klinik II, Abteilung fuer Rheumatologie  Theodor-Stern-Kai 7  Frankfurt am Main, 60590  GERMANY | Ethik-Kommission des Fachbereichs Medizin der Justus-Liebig-Universitaet Giessen  Gaffkystrasse 11 c  Giessen, 35385  GERMANY |
|  |  |  |  |  |  |
| 1181 | Dr. med. Sylke Wagner |  | Dr. med. Thomas Linde | FAE Innere Medizin / Rheumatologie  Ludwig-Wucherer-Str. 10  Halle, 06108  GERMANY | Ethik-Kommission des Fachbereichs Medizin der Justus-Liebig-Universitaet Giessen  Gaffkystrasse 11 c  Giessen, 35385  GERMANY |
|  |  |  |  |  |  |

## Korea, Republic Of

**Coordinating Investigators:**

<None Entered>

| **Center** | **Principal Investigator** | **Co-Investigator(s)** | **Sub-Investigator(s)** | **Address(es)** | **Institutional Review Board or Ethics Committee Address(es)** |
| --- | --- | --- | --- | --- | --- |
|  |  |  |  |  |  |
| 1046 | Eun Bong Lee |  | In Ah Choi  Jin Hyun Kim  Joonwan Kim  Chul Kim  Eun Young Lee  Yeong-Wook Song  Ran Song  Young Im Yoon | Seoul National University Hospital, Rheumatology, Internal Medicine  28 Yongon-dong, Chongno-gu  Seoul, 110-744  KOREA, REPUBLIC OF | Seoul National University Hospital Institutional Review Board  28 Yongon-dong, Chongno-gu  Seoul, 110-744  KOREA, REPUBLIC OF |
|  |  |  |  |  |  |
| 1047 | Dae-Hyun Yoo |  | Dr. Sang-Cheol Bae  So Young Bang  Soo Kyung Cho  Chan-Bum Choi  Jae-Bum Jun  Kyong Hee Jung  Young Sam Kim  Tae-Hwan Kim  Eun Mi Kim  Kyeong A. Lee  So Yeon Park  Yoon-Kyoung Sung | Hanyang University Hospital, Department of Rheumatology  17 Hengdang-dong, Seongdong-gu  Seoul, 133-792  KOREA, REPUBLIC OF | Hanyang University Hospital IRB  17 Haengdang-dong, Seondong-gu  Seoul, 133-792  KOREA, REPUBLIC OF |
|  |  |  |  |  |  |
| 1048 | Dr. Shin-Seok Lee |  | Seong-Rye Seo | Chonnam National University Hospital  8, Hak-dong, Dong-gu  Gwangju, 501-757  KOREA, REPUBLIC OF | IRB of Chonnam National University Hospital  8, Hak-dong, Dong-gu  Gwangju, 501-757  KOREA, REPUBLIC OF |
|  |  |  |  |  |  |
| 1169 | Jung-Yoon Choe |  | Hyun-Young Jung  Dr. Seong-Kyu Kim  Hwa-Jeong Lee  Dr. Sung-Hoon Park | Daegu Catholic University Medical Center, Department of Rheumatology  3056-6 Daemyung-4 dong  Nam-gu  Daegu, 705-718  KOREA, REPUBLIC OF | IRB of Daegu Catholic University Medical Center  #202, Geumgang Villa, 991-8, Daemyung-10 dong Nam-gu,  Daegu, 705-812  KOREA, REPUBLIC OF |
|  |  |  |  |  |  |

## Mexico

**Coordinating Investigators:**

<None Entered>

| **Center** | **Principal Investigator** | **Co-Investigator(s)** | **Sub-Investigator(s)** | **Address(es)** | **Institutional Review Board or Ethics Committee Address(es)** |
| --- | --- | --- | --- | --- | --- |
|  |  |  |  |  |  |
| 1089 | Dr. Carlos Abud-Mendoza |  | Dr. Enrique Cuevas-Orta  Dr. Ricardo Moreno-Valdes  Dr. Juan Cruz Rizo-Rodriguez  Dr. Martin Saldana-Barnad  Dra. Eva N. Santillan-Guerrero | Hospital Central Dr. Ignacio Morones Prieto Unidad Regional de Reumatologia y Osteoporosis  Avenida Venustiano Carranza 2395  Zona Universitaria  San Luis Potosi, SLP 78240  MEXICO | Comite de Investigacion y Etica  Hospital Central Dr. Ignacio Morones Prieto  Avenida Venustiano Carrranza 2395  Zona Universitaria  San Luis Potosi, SLP 78240  MEXICO |
|  |  |  |  |  |  |
| 1090 | Dr. Diego Cesar-Ricardo Ramos-Remus |  | Dr. Francisco Javier Aceves-Avila  Sergio Duran-Barragan  Dr. Guillermo Hernandez-Rios  Dr. Adriana Sanchez-Ortiz | Unidad de Investigacion en Enfermedades Cronico Degenerativas  Colomos 2292, Providencia  Guadalajara, Jalisco 44620  MEXICO | Comite de Bioetica de la Unidad de Investigacion en Enfermedades Cronico-Degenerativas  Colomos 2292  Col. Providencia  Guadalajara, Jalisco 44620  MEXICO |
|  |  |  |  |  |  |
| 1142 | Dr. Rafael Horacio Cornejo-Ballesteros |  | Dr. Marco Antonio Escobedo-Madrigal  Benigno Figueroa-Nunez  Rafael Garcia-Lopez  Noe Pina-Estrada  Jorge Villalpando-Espinoza | Clinica de Enfermedades Cronicas y Procedimientos Especiales SC  Fray Bernardino de Sahagun 101  Fraccionamiento Mirador de Punhuato  Morelia, Michoacan 58249  MEXICO | Comision de Etica e Investigacion CECYPE  Comision de Etica e Investigacion  Fray Bernardino de Sahagun 101  Fraccionamiento Mirador del Punhuato  Morelia, Michoacan 58249  MEXICO |
|  |  |  |  |  |  |
| 1143 | Dr. Maria de Lourdes Sanchez-Gonzalez |  | Dr. Leonor Adriana Barile-Fabris  Dr. Beatriz De Cortina-Camou | Hospital Angeles del Pedregal  Periferico Sur 3697  Consultorio Sotano 33  Mexico, DF 10700  MEXICO | Comite de Bioetica del Instituto de Ciencias Biomedicas Angeles  Camino a Santa Teresa 1055  Torre Especialidades piso 14  Heroes de Padierna  Mexico, DF 10700  MEXICO |
|  |  |  |  |  |  |

## Philippines

**Coordinating Investigators:**

<None Entered>

| **Center** | **Principal Investigator** | **Co-Investigator(s)** | **Sub-Investigator(s)** | **Address(es)** | **Institutional Review Board or Ethics Committee Address(es)** |
| --- | --- | --- | --- | --- | --- |
|  |  |  |  |  |  |
| 1099 | Dr. Allan E. Lanzon |  | Rosalia Manaloto  Dr. Ma. Florence K. Ortiz-Dequina | Mary Mediatrix Medical Center  3rd Floor HB Calleja Bldg.  J.P. Laurel Highway  Lipa City, Batangas 4217  PHILIPPINES | Mary Mediatrix Medical Center  Institutional Review Board  Mary Mediatrix Medical Center  J.P. Laurel Highway  Lipa City, Batangas  PHILIPPINES |
|  |  |  |  |  |  |
| 1107 | Dr. Harold Michael P. Gomez |  | Dr. Gemalyn F. Pineda-Gueco | Angeles University Foundation Medical Center  3rd Floor Infection Control and Research Room  Angeles City, Pampanga 2009  PHILIPPINES | Institutional Review Board  College of Medicine  3rd Floor San Agustin Building  Angeles University Foundation Medical Center  McArthur Highway  Angeles City, Pampanga 2009  PHILIPPINES |
|  |  |  |  |  |  |
| 1140 | Josephine Abao Lim |  | Rhoel James Timothy O. Dejano  Dr. Ronald Eullaran | Chong Hua Medical Arts Center  Room 102  J. Llorente Extension  Cebu City, 6000  PHILIPPINES | Chong Hua Hospital  Institutional Review Board  Chong Hua Hospital  Fuente Osmeña Blvd.  Cebu City, 6000  PHILIPPINES |
|  |  |  |  |  |  |

## Poland

**Coordinating Investigators:**

<None Entered>

| **Center** | **Principal Investigator** | **Co-Investigator(s)** | **Sub-Investigator(s)** | **Address(es)** | **Institutional Review Board or Ethics Committee Address(es)** |
| --- | --- | --- | --- | --- | --- |
|  |  |  |  |  |  |
| 1054 | Dr. Jerzy Klimczak (Previous PI)  Dr. Joanna Badowska |  | Milosz Gawin  Dr. Jerzy Klimczak  Dr. Malgorzata Klimczak  Dr. Anna Wlodarczak | Niepubliczny Specjalistyczny Zaklad Opieki Zdrowotnej Medicus  ul. Bielska 37  Cieszyn, 43-400  POLAND | Komisja Bioetyczna przy Okregowej Izbie Lekarskiej w Gdansku  ul. Sniadeckich 33  Gdansk, 80-204  POLAND |
|  |  |  |  |  |  |
| 1055 | Dr. Elzbieta Langer-Bieda |  | Dr. Przemyslaw Borowy  Ass. Prof. Edward Czerwinski  Dr. Jolanta Osieleniec  Anetta Pawlus | Krakowskie Centrum Medyczne NZOZ  Kopernika 32  Krakow, 31-501  POLAND | Komisja Bioetyczna przy Okregowej Izbie Lekarskiej w Gdansku  ul. Sniadeckich 33  Gdansk, 80-204  POLAND |
|  |  |  |  |  |  |
| 1056 | Dr. Jaroslaw Marcinkiewicz |  | Dr. Iwonna Lajborek-Czyz  Dr. Donata Licznerska-Chabior  Dr. Bozena Poblocka  Dr. Ewa Wojtowicz  Dr. Romana Zak | Wojewodzki Zespol Reumatologiczny im. dr J. Titz-Kosko  ul. Grunwaldzka 1/3  Sopot, 81-759  POLAND | Komisja Bioetyczna przy Okregowej Izbie Lekarskiej w Gdansku  ul. Sniadeckich 33  Gdansk, 80-204  POLAND |
|  |  |  |  |  |  |
| 1100 | Dr. Artur Racewicz |  | Dr. Helena Denisiuk  Dr. Malgorzata Fiedorczyk  Dr. Dorota Golaszewska  Dr. Sylwia Kalinko  Dr. Anna Madrzak  Dr. Ewa Markiewicz  Dr. Jerzy Supronik | NZOZ CENTRUM MEDYCZNE  ul. Pulaskiego 69  Bialystok, 15-337  POLAND | Komisja Bioetyczna przy Okregowej Izbie Lekarskiej w Gdansku  ul. Sniadeckich 33  Gdansk, 80-204  POLAND |
|  |  |  |  |  |  |
| 1154 | Prof. Pawel Hrycaj |  | Dr. Katarzyna Cebrowska  Dr. Lidia Fornalska  Dr. Elzbieta Gigiel  Dr. Lukasz Hordecki  Dr. Kajetan Kisiel  Ewa Kloskowska  Dr. Michal Moskal  Dr. Anna Olewicz-Gawlik  Dr. Dorota Zapolska-Pytlik | Oddzial Reumatologiczny, Szpital im. Teodora Dunina  Samodzielny Publiczny Zespol Opieki Zdrowotnej w Koscianie  Ul. Szpitalna 7  Koscian, 64-000  POLAND | Komisja Bioetyczna przy Okregowej Izbie Lekarskiej w Gdansku  ul. Sniadeckich 33  Gdansk, 80-204  POLAND |
|  |  |  |  |  |  |
| 1167 | Maria Rell-Bakalarska |  | Dr. Elzbieta Eyman  Ewa Klimczak  Dr. Bozena Kowalczuk  Robert Rupinski  Iwona Slowinska  Dr. Ewa Walewska  Dr. Malgorzata Wieteska  Agnieszka Zielinska | Rheuma Medicus - Specjalistyczne Centrum Reumatologii i Osteoporozy  Ul. Pruszkowska 6  Warszawa, 02-118  POLAND | Komisja Bioetyczna przy Okregowej Izbie Lekarskiej w Gdansku  ul. Sniadeckich 33  Gdansk, 80-204  POLAND |
|  |  |  |  |  |  |
| 1168 | Slawomir Jeka |  | Dr. Radoslaw Brukiewa  Dr. Dominik Chraniuk  Dr. Szymon Kudlicki  Rafal Wojciechowski | NZOZ "NASZ LEKARZ"  Praktyka Grupowa Lekarzy Rodzinnych z Przychodnia Specjalistyczna  ul. Szczytna 20  Torun, 87-100  POLAND | Komisja Bioetyczna przy Okregowej Izbie Lekarskiej w Gdansku  ul. Sniadeckich 33  Gdansk, 80-204  POLAND |
|  |  |  |  |  |  |

## Slovakia

**Coordinating Investigators:**

<None Entered>

| **Center** | **Principal Investigator** | **Co-Investigator(s)** | **Sub-Investigator(s)** | **Address(es)** | **Institutional Review Board or Ethics Committee Address(es)** |
| --- | --- | --- | --- | --- | --- |
|  |  |  |  |  |  |
| 1117 | Dr. Pavol Polak |  | Dr. Renata Polakova | Nestatna reumatologicka ambulancia, MUDr. Pavol Polak, s.r.o.  Vojtecha Spanyola 43  Zilina, 010 01  SLOVAKIA | Eticka komisia  Zilinskeho samospravneho kraja  Komenskeho 48  Zilina, 011 09  SLOVAKIA  Eticka komisia, Trenciansky samospravny kraj  K dolnej stanici 7282/20A  Trencin, 911 01  SLOVAKIA |
|  |  |  |  |  |  |
| 1118 | Dr. Peter Belica |  | Jana Barkociova  Dr. Terezia Belicova | Reumatologicka ambulancia, Ecclesia, s.r.o.  SNP 42/A  Nove Zamky, 94001  SLOVAKIA | Eticka komisia Nitrianskeho samospravneho kraja  Stefanikova trieda 69  Nitra, 949 60  SLOVAKIA  Eticka komisia, Trenciansky samospravny kraj  K dolnej stanici 7282/20A  Trencin, 911 01  SLOVAKIA |
|  |  |  |  |  |  |
| 1119 | Dr. Agnesa Szolnokiova |  | Dr. Mariana Bartosova  Dr. Gizela Szolnokiova | AAGS, s.r.o. , nestatne zdravotnicke zariadenie  Reumatologicka ambulancia  Velkoblahovska 10  Dunajska Streda, 92901  SLOVAKIA | Eticka komisia  Trnavskeho samospravneho kraja  Starohajska 10  Trnava, 917 01  SLOVAKIA  Eticka komisia, Trenciansky samospravny kraj  K dolnej stanici 7282/20A  Trencin, 911 01  SLOVAKIA |
|  |  |  |  |  |  |
| 1120 | Dr. Maria Kolkusova |  | Emilia Kostelanska  Dr. Lubomira Simova | Reumatologicka Ambulancia  Nemocnicna 986  Povazska Dystrica, 017 01  SLOVAKIA | Eticka komisia, Trenciansky samospravny kraj  K dolnej stanici 7282/20A  Trencin, 911 01  SLOVAKIA |
|  |  |  |  |  |  |
| 1121 | MUDr. Danica Telepkova |  | Dr. Zuzana Semanova  Dr. Marieta Sencarova | Interna a reumatologicka ambulancia  MUDr. Danica Telepkova, s.r.o.  Strojarenska 13  Kosice, 040 01  SLOVAKIA | Eticka komisia Kosickeho samospravneho kraja  Namestie Maratonu mieru 1  Kosice, 042 66  SLOVAKIA  Eticka komisia, Trenciansky samospravny kraj  K dolnej stanici 7282/20A  Trencin, 911 01  SLOVAKIA |
|  |  |  |  |  |  |
| 1122 | Dr. Zuzana Bubanova |  | Dr. Eva Kokavcova | Reumatologicka ambulancia, Poliklinika Ruzinov  Ruzinovska 6  Bratislava, 82606  SLOVAKIA | Eticka komisia  Bratislavskeho samospravneho kraja  Sabinovska 16  P.O.Box 106  Bratislava, 820 05  SLOVAKIA  Eticka komisia, Trenciansky samospravny kraj  K dolnej stanici 7282/20A  Trencin, 911 01  SLOVAKIA |
|  |  |  |  |  |  |

## Spain

**Coordinating Investigators:**

<None Entered>

| **Center** | **Principal Investigator** | **Co-Investigator(s)** | **Sub-Investigator(s)** | **Address(es)** | **Institutional Review Board or Ethics Committee Address(es)** |
| --- | --- | --- | --- | --- | --- |
|  |  |  |  |  |  |
| 1029 | Juan Garcia Meijide |  | Myriam Liz Graña  Manuel Pombo Suarez | HOSPITAL NUESTRA SEÑORA DE LA ESPERANZA  AVENIDA DE LAS BURGAS, 2  SANTIAGO DE COMPOSTELA, A CORUÑA 15705  SPAIN | Comité Ético de Investigación de Galicia (SERGAS)  ETHICS COMMITTEE OF CLINIC INVESTIGATION  DIVISION DE FARMACIA Y PRODUCTOS  C/ SAN LAZARO, S/N  CONSELLERIA DE SANIDADE  SANTIAGO DE COMPOSTELA, A CORUÑA 15703  SPAIN |
|  |  |  |  |  |  |
| 1030 | Francisco J. Blanco Garcia |  | Jesus Carlos Fernandez Lopez  Mercedes Freire Gonzalez  Derikah Tatiana Gonzalez Perez  Natividad Oreiro Villar | COMPLEJO HOSPITALARIO UNIVERSITARIO A CORUÑA  LABORATORIO DE INVESTIGACION. EDIFICIO ANEXO AL HOSPITAL MATERNO INFANTIL  C/. XUBIAS DE ARRIBA, 84  A CORUÑA, A CORUÑA 15006  SPAIN | Comité Ético de Investigación de Galicia (SERGAS)  ETHICS COMMITTEE OF CLINIC INVESTIGATION  DIVISION DE FARMACIA Y PRODUCTOS  C/ SAN LAZARO, S/N  CONSELLERIA DE SANIDADE  SANTIAGO DE COMPOSTELA, A CORUÑA 15703  SPAIN |
|  |  |  |  |  |  |
| 1051 * | Jose Maria Pego Reigosa |  | Iñigo Hernandez Rodriguez  Ruben Ouviña Arribas | HOSPITAL DO MEIXOEIRO  CONSULTA DE REUMATOLOGIA, 1ª PLANTA  ALTO DO MEIXOEIRO S/N  VIGO, PONTEVEDRA 36200  SPAIN | Comité Ético de Investigación de Galicia (SERGAS)  ETHICS COMMITTEE OF CLINIC INVESTIGATION  DIVISION DE FARMACIA Y PRODUCTOS  C/ SAN LAZARO, S/N  CONSELLERIA DE SANIDADE  SANTIAGO DE COMPOSTELA, A CORUÑA 15703  SPAIN |
|  |  |  |  |  |  |
| 1058 | Desamparados Ybañez Garcia |  | Maria Teresa Contreras Martinez  Juan Jose Garcia Borras  Begoña Laiz Marro  Mª Luisa Muñoz Guillen  Rosa Negueroles Albuixech  Jose Luis Valero Sanz | Hospital Universitari i Politecnic La Fe de Valencia  Servicio de Reumatologia. Torre B. 6ª Planta.  Bulevar Sur, s/n  Valencia, Valencia 46026  SPAIN | Comité Ético de Investigación de Galicia (SERGAS)  ETHICS COMMITTEE OF CLINIC INVESTIGATION  DIVISION DE FARMACIA Y PRODUCTOS  C/ SAN LAZARO, S/N  CONSELLERIA DE SANIDADE  SANTIAGO DE COMPOSTELA, A CORUÑA 15703  SPAIN |
|  |  |  |  |  |  |
| 1066 | Federico Navarro Sarabia |  | Dr. Rafael Ariza Ariza  Maria Fernandez Alba  Maria Dolores Garcia Armario  Dra. Blanca Estela Hernandez Cruz  Manuel Maqueda Lopez  Maria Victoria Navarro Compan  Dolores Ruiz Montesinos | HOSPITAL UNIVERSITARIO VIRGEN MACARENA  SERVICIO DE REUMATOLOGIA. PLANTA SEMISOTANO  AVDA. DR. FEDRIANI Nº3  SEVILLA, SEVILLA 41009  SPAIN | Comité Ético de Investigación de Galicia (SERGAS)  ETHICS COMMITTEE OF CLINIC INVESTIGATION  DIVISION DE FARMACIA Y PRODUCTOS  C/ SAN LAZARO, S/N  CONSELLERIA DE SANIDADE  SANTIAGO DE COMPOSTELA, A CORUÑA 15703  SPAIN |
|  |  |  |  |  |  |
| 1077 | Isabel Mateo Bernardo |  | Alexia de Juanes Montmeterme  Maria Galindo Izquierdo  Javier Garcia Gonzalez  Rosa Maria Gonzalez Crespo  Beatriz Joven Ibañez | HOSPITAL 12 DE OCTUBRE  SERVICIO DE REUMATOLOGIA - PLANTA SEMISOTANO  AVDA. DE CORDOBA S/N  MADRID, MADRID 28041  SPAIN | Comité Ético de Investigación de Galicia (SERGAS)  ETHICS COMMITTEE OF CLINIC INVESTIGATION  DIVISION DE FARMACIA Y PRODUCTOS  C/ SAN LAZARO, S/N  CONSELLERIA DE SANIDADE  SANTIAGO DE COMPOSTELA, A CORUÑA 15703  SPAIN |
|  |  |  |  |  |  |

## Thailand

**Coordinating Investigators:**

<None Entered>

| **Center** | **Principal Investigator** | **Co-Investigator(s)** | **Sub-Investigator(s)** | **Address(es)** | **Institutional Review Board or Ethics Committee Address(es)** |
| --- | --- | --- | --- | --- | --- |
|  |  |  |  |  |  |
| 1109 | Prof. Worawit Louthrenoo |  | Assist. Prof. Nuntana Kasitanon  Suparaporn Wangkaew | Division of Rheumatology, Department of Internal Medicine, Faculty of Medicine  Chiang Mai University  110 Intavaroros Road  Amphoe Muang, Chiang Mai 50200  THAILAND | Research Ethics Committee 1  Research Ethics Committee 1, Faculty of Medicine, Chiang Mai University  110 Intavaroros Road  Amphoe Muang, Chiang Mai 50200  THAILAND |
|  |  |  |  |  |  |
| 1139 | Dr. Paijit Asavatanabodee |  | Dr. Peerawat Boonyateerana  Sumapa Chaiamnuay  Chokchai Kittiyanpanya  Pongthorn Narongroeknawin  Dr. Rattapol Pakchotanon  Suphawan Phukongchai  Tarinee Rojsakulkit | Rheumatology Unit, Department of Internal Medicine, Phramongkutklao Hospital  315  Rajavithi Road,  Rajathevee, Bangkok 10400  THAILAND | Institutional Review Board Royal Thai Army Medical Department  317 Rajavithi Road  Rajathevee  Bangkok, 10400  THAILAND |
|  |  |  |  |  |  |

## United Kingdom

**Coordinating Investigators:**

<None Entered>

| **Center** | **Principal Investigator** | **Co-Investigator(s)** | **Sub-Investigator(s)** | **Address(es)** | **Institutional Review Board or Ethics Committee Address(es)** |
| --- | --- | --- | --- | --- | --- |
|  |  |  |  |  |  |
| 1060 | Dr. Emmanuel George |  | Dr. Priyanka Chandratre  Peter Chapman  Dr. Yee Ho Chiu  Sarah Gibson  Nicola Jeffries  Vipin Tayal | Arrowe Park Hospital  Department of Rheumatology  Arrowe Park Road  Wirral, Merseyside CH49 5PE  UNITED KINGDOM | West Midlands Research Ethics Commitee  West Midlands Research Ethics Commitee  Prospect House  Fishing Line Road  Enfield, Redditch B97 6EW  UNITED KINGDOM |
|  |  |  |  |  |  |
| 1065 | Dr. T. Sheeran |  | Dr. Abdul Baker  Dr. George Hirsch  Deborah Lloyd  Jacqueline McPeake  Dr. Thomas Price  Samantha Roskell  Dr S. Venkatachalam  Ramin Yazdani | Cannock Rheumatology Dept., Cannock Chase Hospital  Brunswick Road  Cannock, Staffs WS11 2XY  UNITED KINGDOM | West Midlands Research Ethics Commitee  West Midlands Research Ethics Commitee  Prospect House  Fishing Line Road  Enfield, Redditch B97 6EW  UNITED KINGDOM |
|  |  |  |  |  |  |
| 1123 | Dr. Karen May Jane Douglas |  | Dr. George Hirsch  Prof. George D. Kitas  Stephanie Mole  Shirley Ann O¿Hare  Dr. Tracey Toms  Elizabeth Wells | Department of Rheumatology  Dudley Group of Hospitals NHS Trust  Esk House, Russells Hall Hospital  Dudley, West Midlands, DY1 2HQ  UNITED KINGDOM | West Midlands Research Ethics Commitee  West Midlands Research Ethics Commitee  Prospect House  Fishing Line Road  Enfield, Redditch B97 6EW  UNITED KINGDOM |
|  |  |  |  |  |  |

## United States

**Coordinating Investigators:**

<None Entered>

| **Center** | **Principal Investigator** | **Co-Investigator(s)** | **Sub-Investigator(s)** | **Address(es)** | **Institutional Review Board or Ethics Committee Address(es)** |
| --- | --- | --- | --- | --- | --- |
|  |  |  |  |  |  |
| 1020 | Dr. Guillermo Jose Valenzuela |  | Dr. Richard Anthony McLean  Shawn M. Saunders | Berma Research Group  Suite B  140 SW 84th Avenue  Plantation, FL 33324  UNITED STATES | Quorum Institutional Review Board  Suite 1000  1601 Fifth Avenue  Seattle, WA 98101  UNITED STATES |
|  |  |  |  |  |  |
| 1021 | Dr. Beata Joanna Filip-Majewski |  | Dr. Leslie D. McCasland  Barbara A. Wagoner | NEA Baptist Clinic  311 East Matthews Avenue  Jonesboro, AR 72401  UNITED STATES  NEA Baptist Clinic  Clinical Research Center  Suite C  416 East Washington Avenue  Jonesboro, AR 72401  UNITED STATES | Quorum Institutional Review Board  Suite 1000  1601 Fifth Avenue  Seattle, WA 98101  UNITED STATES |
|  |  |  |  |  |  |
| 1026 * | Dr. Moges Sisay |  | Dr. Richard Earnest Bell  Dr. Mujtaba F. Tapal | TSARR, LLC  (Tri-State Arthritis and Rheumatology Research, LLC)  3801 Bellemeade Avenue, Suite 320  Evansville, IN 47714  UNITED STATES | Quorum Institutional Review Board  Suite 1000  1601 Fifth Avenue  Seattle, WA 98101  UNITED STATES |
|  |  |  |  |  |  |
| 1028 * | Dr. Basit A. Malik |  | Kimberly L. Talbot | Professional Research Network of Kansas, LLC  Suite 400  345 Riverview Street  Wichita, KS 67203  UNITED STATES | Quorum Institutional Review Board  Suite 1000  1601 Fifth Avenue  Seattle, WA 98101  UNITED STATES |
|  |  |  |  |  |  |
| 1033 | Dr. Douglas Menzies Haselwood |  | Dr. Anupama S. Bhat  Dr. Chandan D.S. Cheema  Harold Jesse Ewing | Med Investigations  (Administrative Office)  Suite 260  6600 Mercy Court  Fair Oaks, CA 95628  UNITED STATES  Office of Douglas M. Haselwood, MD  Suite 1201  151 North Sunrise Avenue  Roseville, CA 95661  UNITED STATES | Quorum Institutional Review Board  Suite 1000  1601 Fifth Avenue  Seattle, WA 98101  UNITED STATES |
|  |  |  |  |  |  |
| 1037 | Dr. Joel Charles Silverfield |  | Dr. Michael Claude Burnette  Dr. Laura McIlwain Cruse  Dr. Bernard F. Germain  Dr. Harris Hugh McIlwain  Dr. Kimberly McIlwain Smith | Tampa Medical Group, P.A.  Suite 406  13801 Bruce B. Downs Boulevard  Tampa, FL 33613  UNITED STATES | Quorum Institutional Review Board  Suite 1000  1601 Fifth Avenue  Seattle, WA 98101  UNITED STATES |
|  |  |  |  |  |  |
| 1038 | Dr. Edward Joel Fudman |  | Dr. Stephanie Ann Booth  Dr. Brian Sam Sayers | Austin Rheumatology Research  Suite 702  1301 West 38th Street  Austin, TX 78705  UNITED STATES  Austin Rheumatology Research  Suite 110  1301 West 38th Street  Austin, TX 78705  UNITED STATES | Quorum Institutional Review Board  Suite 1000  1601 Fifth Avenue  Seattle, WA 98101  UNITED STATES |
|  |  |  |  |  |  |
| 1039 | Dr. Jeffrey Stewart Neal |  | Dr. Kelly K. Cole  Dr. Rita M. Egan  Dr. Paul M. Goldfarb Jr. | Bluegrass Community Research, Inc.  330 Waller Avenue  Lexington, KY 40504  UNITED STATES | Quorum Institutional Review Board  Suite 1000  1601 Fifth Avenue  Seattle, WA 98101  UNITED STATES |
|  |  |  |  |  |  |
| 1042 * | Dr. Jefrey Dale Lieberman |  | Nancy E. Green | Jeffrey D. Lieberman, MD, PC  2712 North Decatur Road  Decatur, GA 30033  UNITED STATES | Quorum Institutional Review Board  Suite 1000  1601 Fifth Avenue  Seattle, WA 98101  UNITED STATES |
|  |  |  |  |  |  |
| 1044 | Dr. Stephen Michael Lindsey |  | Lisa Alleman  Dr. Bobby J. Dupre  Sharon Holder  Dr. Sean E. Shannon | Ochsner Clinic Foundation, Baton Rouge  2nd Floor Research  9001 Summa Avenue  Baton Rouge, LA 70809  UNITED STATES | Ochsner Clinic Foundation Institutional Review Board  1514 Jefferson Highway  New Orleans, LA 70121  UNITED STATES |
|  |  |  |  |  |  |
| 1052 | Dr. Stuart Michael Weisman |  | Denise M. Munro  Jean Petrecz  Jennifer R. Roland | Boulder Medical Center, P.C.  2750 Broadway Street  Boulder, CO 80304  UNITED STATES | Quorum Institutional Review Board  Suite 1000  1601 Fifth Avenue  Seattle, WA 98101  UNITED STATES |
|  |  |  |  |  |  |
| 1053 | Dr. Mohammed Yaseen Abubaker |  | Nancy E. Green | Marietta Rheumatology  Suite 100  670 North Avenue  Marietta, GA 30060  UNITED STATES | Quorum Institutional Review Board  Suite 1000  1601 Fifth Avenue  Seattle, WA 98101  UNITED STATES |
|  |  |  |  |  |  |
| 1057 | Dr. Shelly Pearl Kafka |  | Laura J. Stavrakis  Dr. Dominick R. Woofter | Mountain State Clinical Research  Suite 303A  300 Davison Road  Clarksburg, WV 26301  UNITED STATES | Quorum Institutional Review Board  Suite 1000  1601 Fifth Avenue  Seattle, WA 98101  UNITED STATES |
|  |  |  |  |  |  |
| 1078 | Dr. Geneva Louise Hill |  | DeEtte M. Burton  Dr. Josette J. Johnson  Dr. Jeffrey Geldert Lawson | Piedmont Arthritis Clinic, PA  Suite 400  3 St. Francis Drive  Greenville, SC 29601  UNITED STATES | Quorum Institutional Review Board  Suite 1000  1601 Fifth Avenue  Seattle, WA 98101  UNITED STATES |
|  |  |  |  |  |  |
| 1084 | Dr. Philip Judson Mease |  | Nicole M. Furfaro  Lyne A. Schaefer-Alfonse  Susan Williams-Judge | Investigational Drug Service  747 Broadway  Seattle, WA 98122  UNITED STATES  Swedish Medical Center  747 Broadway  Seattle, WA 98122  UNITED STATES  Swedish Rheumatology Associates  Suite 1000  1101 Madison Street  Seattle, WA 98104  UNITED STATES | Western Institutional Review Board  3535 Seventh Avenue Southwest  Olympia, WA 98502  UNITED STATES |
|  |  |  |  |  |  |
| 1111 | Dr. Charles Allen Birbara |  | Dr. Sheela Kumar  Dr. Nassif Maalouli  Dr. Basheer Rahmoun | Clinical Pharmacology Study Group  26 Queen Street  Worcester, MA 01610  UNITED STATES | Quorum Institutional Review Board  Suite 1000  1601 Fifth Avenue  Seattle, WA 98101  UNITED STATES |
|  |  |  |  |  |  |
| 1113 | Dr. Michael Edmund Luggen |  | Laura Mary Alexander  Mary M. Bengel-Rogers  Dr. Kerrin D. Burte  Dr. Matthew Franklin Burton  Dr. Gregory Joseph DeLorenzo  Dr. David Greenblatt  Karie Kathern King  Peggy Ann Slattery | Cincinnati Rheumatic Disease Study Group, Inc.  at the Deaconess Arthritis Center  311 Straight Street  Cincinnati, OH 45219  UNITED STATES | Quorum Institutional Review Board  Suite 1000  1601 Fifth Avenue  Seattle, WA 98101  UNITED STATES |
|  |  |  |  |  |  |
| 1114 * | Dr. Menachem Kohen |  | Dr. Michael J. McCartney | ActivMed Practices and Research  Suite A  One Water Street  Haverhill, MA 01830  UNITED STATES | Quorum Institutional Review Board  Suite 1000  1601 Fifth Avenue  Seattle, WA 98101  UNITED STATES |
|  |  |  |  |  |  |
| 1115 | Dr. Ara Hagop Dikranian |  | Dr. Puja Chitkara  Dr. Michael Ira Keller  Timothy F. Lazarek  Jennifer Marconato  Dr. Smitha Chiniga Reddy | San Diego Arthritis Medical Clinic  Suite 300  3633 Camino Del Rio South  San Diego, CA 92108  UNITED STATES | Quorum Institutional Review Board  Suite 1000  1601 Fifth Avenue  Seattle, WA 98101  UNITED STATES |
|  |  |  |  |  |  |
| 1116 | Dr. Roy Mitchell Fleischmann |  | Jean A. Clark  Dr. Stanley Bruce Cohen  Dr. Thomas David Geppert  Dr. Imran Iqbal  Dr. Robert Neil Jenkins  Dr. Talat Jehan Kheshgi  Dr. Zoran Kurepa  Dr. Sharad Lakhanpal  Andrea S. Martin  Dr. Catalina Orozco  Dr. Richard L. Stern  Dayna S. Swan-Flanders  Dr. Jack Bernstein Vine  Andrea S. Wheeler | Metroplex Clinical Research Center  Suite 810  8144 Walnut Hill Lane  Dallas, TX 75231  UNITED STATES | Quorum Institutional Review Board  Suite 1000  1601 Fifth Avenue  Seattle, WA 98101  UNITED STATES |
|  |  |  |  |  |  |
| 1124 | Dr. Arnaldo Torres |  | Ingrid Ferro  Luis M. Garzon | BayCare Outpatient Imaging at Bardmoor  (X-Ray Only)  8787 Bryan Dairy Road  Largo, FL 33777  UNITED STATES  DMI Research, Inc.  6699 90th Avenue North  Pinellas Park, FL 33782  UNITED STATES  St. Petersburg Arthritis Center  6711 38th Avenue North  St. Petersburg, FL 33710  UNITED STATES | Quorum Institutional Review Board  Suite 1000  1601 Fifth Avenue  Seattle, WA 98101  UNITED STATES |
|  |  |  |  |  |  |
| 1131 | Dr. Michael James Fairfax |  | W. Richard Horn  Dr. Yelena A. Misaljevic | ArthroCare, Arthritis Care & Research P.C.  Suite 200  3921 East Baseline Road  Gilbert, AZ 85234  UNITED STATES | Quorum Institutional Review Board  Suite 1000  1601 Fifth Avenue  Seattle, WA 98101  UNITED STATES |
|  |  |  |  |  |  |
| 1158 | Dr. Jeffrey Alan Alper |  | Alisha Krysten Nicole Walker | Jeffrey Alper M.D. Research  689 Ninth Street North  Naples, FL 34102  UNITED STATES | Quorum Institutional Review Board  Suite 1000  1601 Fifth Avenue  Seattle, WA 98101  UNITED STATES |
|  |  |  |  |  |  |
| 1159 | Dr. James Michael Calmes |  | Dr. Naga S. Bushan  Dr. Jose Del Giudice  Dr. Jitendra Indrukumar Vasandani | Arthritis and Osteoporosis Associates, LLP  5220 80th Street  Lubbock, TX 79424  UNITED STATES | Quorum Institutional Review Board  Suite 1000  1601 Fifth Avenue  Seattle, WA 98101  UNITED STATES |
|  |  |  |  |  |  |
| 1160 | Dr. Larry Grant Willis (Previous PI)  Dr. William Martin Schnitz |  | Lindsey May Gillispie  Dr. Carl Patrick Griffin  Destiny S. Heinzig  Dr. Norman Kerr Imes  Dr. Robert Edward Ringrose | Lynn Health Science Institute  Suite 800  3555 North West 58th Street  Oklahoma City, OK 73112  UNITED STATES | Quorum Institutional Review Board  Suite 1000  1601 Fifth Avenue  Seattle, WA 98101  UNITED STATES |
|  |  |  |  |  |  |
| 1161 | Dr. Philip A. Waller |  | Barbara A. Slusher  Dr. Prashanth R. Sunkureddi  DeAnna Elena Wallace | Accurate Clinical Research, Inc.  12553 Gulf Freeway  Houston, TX 77034  UNITED STATES | Quorum Institutional Review Board  Suite 1000  1601 Fifth Avenue  Seattle, WA 98101  UNITED STATES |
|  |  |  |  |  |  |
| 1162 | Dr. Richard Roy Olson |  | Dr. David James Dansdill  Tami M. Kucia | Rockford Orthopedic Associates, Ltd.  324 Roxbury Road  Rockford, IL 61107  UNITED STATES | Quorum Institutional Review Board  Suite 1000  1601 Fifth Avenue  Seattle, WA 98101  UNITED STATES |
|  |  |  |  |  |  |
| 1163 | Dr. Ralph Edward Bennett |  | Scott F. Brown  Dr. Paul H. Caldron  Shawna Carbonniere  Dr. Sheetal K. Chhaya  Candace L. Coggin  Tiffany Anne Fotinos  Dr. Ramina Jajoo  Randy Jay  Maurisa O. Konya  Catherine Sue Lynn  Pamela L. Morris  Dr. Eric Alan Peters  Dora Quan  Dr. John Irwin Starr  Dr. Areena Swarup  Carol M. Swenson  Dr. John Robert Paul Tesser | Arizona Arthritis and Rheumatology Associates, PC  Suite F-150  10599 North Tatum Boulevard  Paradise Valley, AZ 85253  UNITED STATES  Arizona Arthritis and Rheumatology Associates, PC  Suite 505  9305 West Thomas Road  Phoenix, AZ 85037  UNITED STATES  Arizona Arthritis and Rheumatology Associates, PC  Suite 202  1500 South Dobson Road  Mesa, AZ 85202  UNITED STATES  Arizona Arthritis and Rheumatology Associates, PC  Suite 204  5601 West Eugie Avenue  Glendale, AZ 85304  UNITED STATES | Quorum Institutional Review Board  Suite 1000  1601 Fifth Avenue  Seattle, WA 98101  UNITED STATES |
|  |  |  |  |  |  |
| 1165 | Dr. Atul Kumar Singhal |  | Julia A. Dilliard  Angelia Y. Hannah  Sandra Jean Rodriguez | Southwest Rheumatology, PA  Suite 615  18601 LBJ Freeway  Mesquite, TX 75150  UNITED STATES | Quorum Institutional Review Board  Suite 1000  1601 Fifth Avenue  Seattle, WA 98101  UNITED STATES |
|  |  |  |  |  |  |
| 1171 * | Dr. Richard W. Martin |  | Dr. James Birmingham  Josh S. Brinks  Dr. Aaron Eggebeen  Dr. Andrew J. Head | West Michigan Rheumatology  Suite 100  1155 East Paris Avenue Southeast  Grand Rapids, MI 49546  UNITED STATES | Quorum Institutional Review Board  Suite 1000  1601 Fifth Avenue  Seattle, WA 98101  UNITED STATES |
|  |  |  |  |  |  |
| 1172 * | Dr. Mitchell Brian Lowenstein |  | Felicia M. Shonte' Jones  Catherine M. Preston  Dr. Karen Elizabeth Zagar | The Arthritis Center  Suite 2 and 3  32615 US Highway 19 North  Palm Harbor, FL 34684  UNITED STATES | Quorum Institutional Review Board  Suite 1000  1601 Fifth Avenue  Seattle, WA 98101  UNITED STATES |
|  |  |  |  |  |  |
