# Supplementary material for: Changes in serum creatinine in patients with active rheumatoid arthritis treated with tofacitinib: results from clinical trials
Source: Arthritis Res Ther. 2014 Jul 25;16(4):R158. doi: 10.1186/ar4673 (PMC4220634; doi:10.1186/ar4673)
Supplement: Supplementary file 13 — Additional file 13: Table S1: Acute renal failure cases. (DOC 54 KB) [file 13075_2013_4378_MOESM13_ESM.doc]

| **Study** | **Onset day** | **Baseline**  **SCr (mg/dL)** | **Max SCr (mg/dL)** | **Action** | **Outcome** | **Assessment** |
| --- | --- | --- | --- | --- | --- | --- |
| *Phase 3 – 5 mg BID* | | | | | | |
| ORAL Scan | 168 | 1.2 | 1.4 | Discontinued | Resolved | Unclear etiology |
| *Phase 3 – 10 mg BID* | | | | | | |
| ORAL Step^*^ | 171 | 1.6 | 2.2 | Discontinued | Resolved | Pre-renal  Diverticulitis, diarrhea, dehydration |
| ORAL Solo | 130 | 0.9 | 1.1 | No action | Resolved | Pre-renal  Aggressive diuresis, CHF, UTI |
| ORAL Sync | 357 | 0.9 | 2.2 | No action | Died | Pre-renal  Rhabdomyolysis, CHF |
| ORAL Solo | 107 | 1.3 | 1.4 | Discontinued | Died | Pre-renal  Multi-organ failure, cardiac arrest, dehydration, diarrhea |
| ORAL Standard^*^ | 247 | 1.1 | 2.1 | Discontinued | Resolved | Pre-renal  Infection, nephrolithiasis |
| *Phase 3 – Placebo* | | | | | | |
| ORAL Scan^*^ | 191 | 0.7 | NA | Discontinued | Died | Pre-renal  Sepsis, pyelonephritis, hydronephrosis |
| *LTE – 5 mg BID* | | | | | | |
| A3921024^*^ | 604 | 0.7 | NA | Temporarily discontinued | Resolved^a^ | Pre-renal  CHF, dehydration due to intensified diuretics and diarrhea |
| A3921024 | 485 | 1.1 | 1.2 | No action | Died | Pre-renal  Possible multi-organ dysfunction; increased LFTs; dyspnea |
| A3921024 | 64 | 0.6 | NA | No action | Resolved | Unclear etiology |
| A3921024^*^ | 90 | 0.9 | NA | Discontinued | Died | Pre-renal  Multi-organ failure, sepsis requiring inotropic support |
| A3921024^*^ | 958 | 0.9 | NA | Discontinued | Resolved | Pre-renal |
| A3921024^*^ | 182  (post-study) | 0.5 | NA | Discontinued | Ongoing – dialysis then off dialysis | Pre-renal  Sepsis, requiring inotropic support |
| A3921024^*^ | 904 | 1.1 | 4.2 | No action | Resolved | Pre-renal  Dehydration, gastroenteritis |
| A3921024^*^ | 227  (post-study) | 0.8 | NA | Post-study | Died | Pre-renal  Severe pneumonia post-op neurosurgery |
| A3921024^*^ | 85  (post-study) | 0.7 | 2.7 | Post-study | Improved | Unclear etiology – 2 months post‑study discontinuation for hip surgery |
| *LTE – 10 mg BID* | | | | | | |
| A3921024 | 478 | 0.9 | 1.5 | No action | Ongoing | Pre-renal  Worsening CHF |
| A3921024^*^ | 299  (post-study) | 0.7 | NA | Post-study | Resolved | Pre-renal  Dehydration, diarrhea, hypotension |
| A3921024 | 165  (post-study) | 0.7 | 1.5 | Post-study | Resolved | Post-op surgery for metastatic adenocarcinoma, lung abscess, osteomyelitis of rib, and mitral and tricuspid regurgitation |
| A3921024^*^ | 15 | 1.0 | 2.7 | No action | Died | Pre-renal  Septic shock |
| A3921024^*^ | 174 | 1.3 | 5.7 | No action | Resolved | Pre-renal  Severe dehydration, gastroenteritis |
| A3921024 | 117 | 0.8 | 1.5 | No action | Resolved | Pre-renal  Hypotension, adrenal insufficiency, dehydration from viral gastroenteritis |
| A3921024 | 21 | 0.9 | NA | No action | Resolved | Pre-renal  Dehydration, UTI, pneumonia |

*serious adverse event

^a^Died due to brain injury after resolution

MedDRA terms: acute renal failure, renal failure, acute renal insufficiency; terms are inclusive of azotemia and renal impairment events

BID = twice daily; CHF = congestive heart failure; LFTs = liver function tests; LTE = long-term extension; MedDRA = Medical Dictionary for Regulatory Activities; NA = not applicable; SCr = serum creatinine; UTI = urinary tract infection.

LTE study A3921024 [19]; ORAL Scan [5]; ORAL Standard [4]; ORAL Step [2]; ORAL Solo [3]; ORAL Sync [3].
